# Non-covalent double bond sensors for gas-phase infrared spectroscopy of unsaturated fatty acids

**DOI:** 10.1007/s00216-021-03334-3

**Published:** 2021-05-06

**Authors:** Carla Kirschbaum, Kim Greis, Maike Lettow, Sandy Gewinner, Wieland Schöllkopf, Gerard Meijer, Gert von Helden, Kevin Pagel

**Affiliations:** 1 Institut für Chemie und Biochemie, Freie Universität Berlin, 14195 Berlin, Germany; 2 Fritz-Haber-Institut der Max-Planck-Gesellschaft, 14195 Berlin, Germany

**Keywords:** IR spectroscopy, Ion mobility, Mass spectrometry, Fatty acids, Double bond isomers, Non-covalent interactions

## Abstract

The position and configuration of carbon-carbon double bonds in unsaturated fatty acids is crucial for their biological functions and influences health and disease. However, double bond isomers are not routinely distinguished by classical mass spectrometry workflows. Instead, they require sophisticated analytical approaches usually based on chemical derivatization and/or instrument modification. In this work, a novel strategy to investigate fatty acid double bond isomers (18:1) without prior chemical treatment or modification of the ion source was implemented by non-covalent adduct formation in the gas phase. Fatty acid adducts with sodium, pyridinium, trimethylammonium, dimethylammonium, and ammonium cations were characterized by a combination of cryogenic gas-phase infrared spectroscopy, ion mobility-mass spectrometry, and computational modeling. The results reveal subtle differences between double bond isomers and confirm three-dimensional geometries constrained by non-covalent ion-molecule interactions. Overall, this study on fatty acid adducts in the gas phase explores new avenues for the distinction of lipid double bond isomers and paves the way for further investigations of coordinating cations to increase resolution.

**Supplementary Information:**

The online version contains supplementary material available at 10.1007/s00216-021-03334-3.

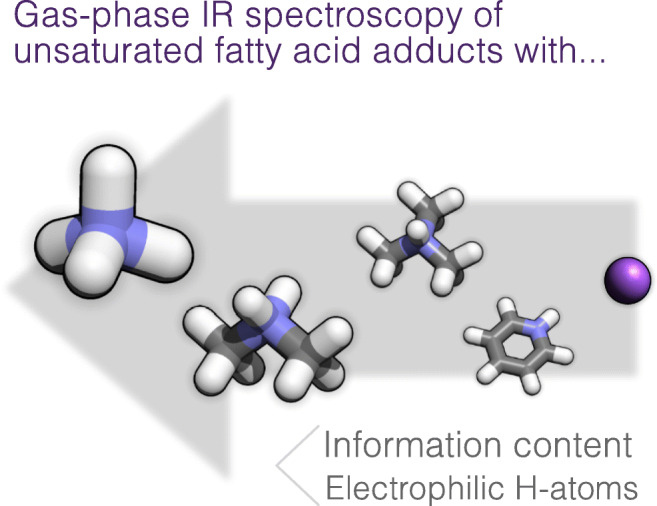

## Introduction

Lipids are essential biomolecules for all forms of life ranging from simple procaryotes to large multicellular organisms. Their molecular structures are as diverse as their dedicated functions, involving energy storage, signaling, assembly of biological membranes, and membrane trafficking [1–3]. Among the most basic lipids are fatty acids (FAs), which serve as building blocks for more complex lipid classes such as glycerolipids, glycerophospholipids, and sphingolipids [4]. Despite their relatively simple framework, FAs exhibit structural microheterogeneity that can have important effects on biophysical membrane properties and biological functionality [5, 6]. In particular, the number, position, and configuration (*trans*/*E* or *cis*/*Z*) of carbon-carbon double bonds (C=C) in unsaturated FAs can vary substantially between different tissues [7–9], and altered distributions of C=C isomers are potential biomarkers for disease diagnosis [10, 11]. For instance, it was recently demonstrated that cancer cells can employ an alternative FA desaturation pathway yielding a measurable increase in FAs with unusual C=C positions [12] and that cancer subtypes can further be differentiated on the basis of C=C location in FAs [13]. The dietary uptake of various unsaturated FAs such as *cis*-/*trans*-FAs or omega-3/omega-6 FAs can have beneficial or deleterious effects on human health [14–16]. Individual and even opposed effects of distinct C=C isomers were impressively demonstrated on the example of atherosclerosis, which is either positively or negatively influenced by two different C=C isomers of conjugated linoleic acid and which yielded contradictory findings when regarded as a single molecular species [17]. Other diseases that are promoted or inhibited by specific C=C isomers are cancer [18], type 2 diabetes [19], and cardiovascular disease [14, 15]. Determining C=C locations is also important in elucidating metabolic pathways. For instance, the C=C isomers oleic acid (18:1, 9*Z*) and *cis*-vaccenic acid (18:1, 11*Z*) are synthesized via substantially different routes, i.e., desaturation of stearic acid (18:0) or elongation of palmitoleic acid (16:1, 9*Z*) [20]. Taking together the pieces of evidence collected during the past decades, it has become evident that modern lipidomics must ensure structural profiling down to the level of C=C isomers to elucidate synthetic pathways and monitor altered lipid metabolism in health and disease [5].

The uncontested main tool used in lipid analysis is mass spectrometry (MS) in combination with soft ionization techniques such as electrospray ionization (ESI) and matrix-assisted laser desorption/ionization (MALDI) [21, 22]. Conventional MS workflows employing collision-induced dissociation (CID) allow for lipid analysis on the first two levels of structural complexity: identification of the lipid class and the overall FA composition, e.g., 18 carbons and one unsaturation (18:1) [7]. However, CID does not induce intrachain fragmentation indicative of C=C location. Therefore, the development of MS-compatible approaches that enable the determination of C=C locations has boomed in recent years and has given rise to a variety of techniques, including ozone-induced dissociation (OzID) [7, 23, 24], Paternò-Büchi reactions [8, 9, 25], epoxidation [26–28] and other oxidation reactions [29], charge inversion [30, 31], radical-directed dissociation [32, 33], and ultraviolet photodissociation [34, 35]. Most of those strategies require on- or offline chemical derivatization of the lipid and do not yield direct information about the C=C configuration.

Cryogenic gas-phase infrared (IR) spectroscopy was recently established as a powerful technique to resolve minute structural differences in sphingolipid and glycolipid isomers [36, 37]. Motivated by one of these previous studies, which showed that both C=C location and configuration in protonated deoxysphingolipids are distinguishable without chemical modification by gas-phase IR spectroscopy [36], we extended the application to FAs in this work. The distinction of sphingolipid isomers in the gas phase relies on a charge-olefin interaction between the protonated primary amine and the C=C bond [38], which induces characteristic N-H vibrations. Sphingolipids thus carry an intrinsic double bond sensor, which is their primary amino group. FAs lack a comparable, electrophilic functional group that could possibly interact with the C=C bond. The aim of this study is therefore to generate similar charge-olefin interactions in FAs by adduct formation with appropriate cations. Non-covalent complexes with sodium, pyridinium, trimethylammonium, dimethylammonium, and ammonium cations were investigated using a combined approach of cryogenic gas-phase IR spectroscopy, ion mobility-mass spectrometry (IM-MS), and quantum chemical calculations.

## Materials and methods

### Reagents and solvents

Oleic acid (9*Z*), elaidic acid (9*E*), *cis*-vaccenic acid (11*Z*) and *trans*-vaccenic acid (11*E*), trimethylammonium chloride, dimethylammonium chloride, ammonium acetate, pyridinium chloride, water, methanol, and acetonitrile were purchased from Sigma-Aldrich (Taufkirchen, Germany). Aqueous stock solutions (100 mM) of each salt were prepared. FAs were dissolved in methanol or acetonitrile (1 mm). Sodium and ammonium adducts were generated from 100 μm fatty acid solution in methanol containing 10 mm ammonium acetate. Trimethylammonium, dimethylammonium, and pyridinium adducts were generated from 500 μm solutions of FAs in acetonitrile containing 1 mm of the respective chloride salt.

### Cryogenic gas-phase IR spectroscopy in helium nanodroplets

IR spectra were measured using a home-built instrument described previously [39–41]. Charged lipid adducts are generated in positive ion mode by nano-ESI from 5 to 10 μL sample solutions using home-made emitters coated with Pd/Pt (needle voltage: 0.6–0.9 kV). After mass-to-charge (*m*/*z*) selection in a quadrupole, the ions are guided into a hexapole ion trap and thermalized by buffer gas cooling (90 K). A pulsed beam of superfluid helium droplets (10 Hz), generated by the expansion of pressurized helium through a cryo-cooled Even-Lavie valve (21 K), traverses the trap and picks up ions. The ions are rapidly cooled to the internal droplet temperature of 0.4 K by evaporation of helium atoms. The doped helium droplets travel towards the interaction region, where the droplet beam overlaps temporally and spatially with the pulsed beam (10 Hz) of the Fritz Haber Institute free-electron laser (FHI FEL) [42]. When the laser wavelength is resonant with a molecular vibration, the ions are released from the droplet and detected by MS. IR spectra are generated by scanning the wavenumber range of interest in steps of 2 cm^−1^ while monitoring the ion count on the time-of-flight detector. Each spectrum is averaged over two separate scans to ensure reproducibility.

### IM-MS: Determination of CCS

Collision cross sections (CCS) were measured on a home-built drift tube ion mobility-mass spectrometer described previously [43]. Non-covalent, charged lipid adducts are generated in positive ion mode by nano-ESI. The ions are accumulated in an electrodynamic entrance funnel and injected into the drift region at 10 Hz. The drift tube is 161.2 cm long and filled with helium at a pressure of 4.1–4.2 mbar. The adducts traverse the drift tube under the influence of a weak electric field, where they collide with helium atoms. Small and compact ions collide less often than larger, more extended ions and therefore spend less time in the drift region. The ions are transferred into high vacuum via a second ion funnel and two consecutive ion guides. After *m/z* selection in a quadrupole, the ions are detected using an electron multiplier detector (ETP Ion Detect, Australia) to record the arrival time distribution (ATD). CCSs were determined by measuring drift times of *m*/*z* selected ions at 11 different drift voltages from 2110 to 1110 V in steps of 100 V, while monitoring temperature and pressure in the drift tube. The resulting mobilities were converted into CCSs according to the Mason-Schamp equation [44] using a software developed in our group (https://github.com/jeriedel/CCS).

### Computational modeling and frequency calculation

Structures and IR spectra of non-covalent FA adducts were computed by sampling the conformational space and DFT optimization of selected structures followed by harmonic frequency calculations. The conformational space was sampled using CREST [45] with the semi-empirical method GFN2-xTB [46] and default settings. Twenty to 25 conformers below a threshold of 15 kJ mol^−1^ (relative to the lowest-energy conformer) were selected using principal component analysis (PCA) of all bond lengths excluding hydrogen atoms using the module sklearn.decomposition.PCA and clustered by k-means clustering [47]. The lowest-energy conformer of each cluster was submitted to DFT optimization at B3LYP+D3/6-311+G(d,p) [48, 49] level of theory followed by a harmonic frequency calculation in Gaussian 16 [50]. Because the harmonic approximation systematically overestimates vibrational frequencies, the computed IR spectra were scaled by single-parameter frequency scaling to enhance the agreement between experiment and theory. The harmonic frequencies were corrected by a scaling factor of 0.965, which has proven accuracy in numerous previous studies on various molecular classes including nucleotides [40], sugars [41, 51, 52], and lipids [36, 37] with comparable methods as used in this work. The harmonic free energies ΔF were calculated at 90 K, according to the temperature in the ion trap. CCSs were calculated for each DFT-optimized conformer at 298.15 K (25 °C) in helium using the software HPCCS [53], which is based on the trajectory method [54]. For the CCS calculation, DFT-computed Merz-Singh-Kollman charges [55] were used. The average CCSs of all computed conformers below a threshold of ΔF = 10 kJ mol^−1^ were calculated for comparison with the experimental CCSs and converted into boxplot diagrams using OriginPro 2020. XYZ coordinates, energetics, and CCSs of all computed conformers are available in the Supplementary Information (ESM).

## Results

### Non-covalent adduct formation of 18:1 fatty acids with cations

**Fig. 1:**
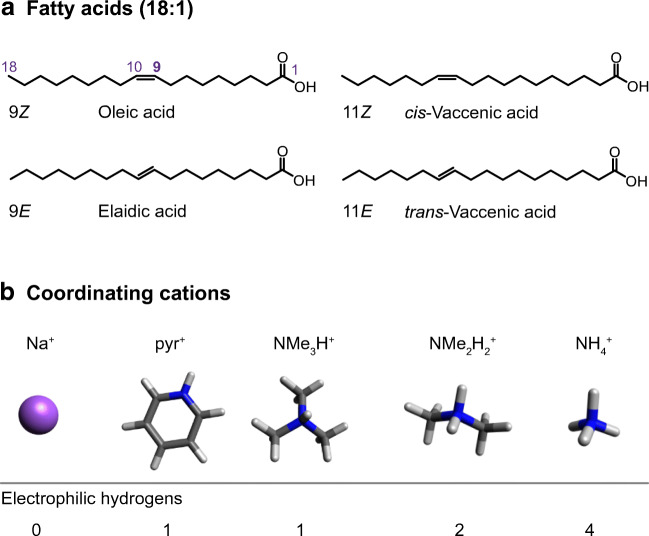
Overview of FA C=C bond isomers (18:1) and cations selected for the formation of non-covalent adducts. The numbering of lipid chains underlying the nomenclature of C=C bonds is exemplified for oleic acid. The cations are ordered according to the number of electrophilic hydrogens available for coordination to electron-rich functional groups

For the investigation of non-covalent FA adducts, a consistent set of four mono-unsaturated 18-carbon FAs (18:1) was selected: oleic acid (9*Z*), elaidic acid (9*E*), *cis*-vaccenic acid (11*Z*), and *trans*-vaccenic acid (11*E*) (Fig. 1). The most abundant C=C isomer of 18:1 FAs in mammalian cells is 9*Z*, followed by 11*Z* [10, 11]. The corresponding *trans*-isomers 9*E* and 11*E* are not naturally synthesized by mammalian enzymes but taken up from various food sources: elaidic acid belongs to the main *trans*-FAs formed during partial hydrogenation of oil in industrial food processing and is therefore elevated in margarine, fried, and bakery products [16]. *Trans*-vaccenic acid is naturally produced by bacterial fermentation in ruminants and therefore taken up with dairy products and meat [16]. Contrary to *cis*-FAs, where the C=C bond typically induces a bend in the lipid chain, *trans*-FAs tend to exhibit a linear hydrocarbon chain, similar to saturated FAs. Differences in the gas-phase geometries of C=C isomers are therefore expected between *cis*- and *trans*-FAs but also between the 9 and 11 C=C positions due to different lengths of the dangling lipid chains before and behind the C=C bond.

Five cations were selected for adduct formation: sodium, pyridinium, trimethylammonium, dimethylammonium, and ammonium. The cations differ by the number of electrophilic hydrogen atoms (0–4), size, and shape (Fig. 1). Depending on the number of electrophilic hydrogens, the cation can either only interact with the carboxyl group (1 H) or with both carboxyl group and C=C bond of the FA. The cation’s size and shape should further influence the overall size and geometry of the corresponding non-covalent complex. In general, the efficiency of adduct formation during the nano-ESI process decreases with increasing size of the coordinating cation, even though the proton affinity increases with the number of methyl groups from ammonium to trimethylammonium, which should facilitate ionization [56]. The observation of pyridinium, tri-, and dimethylammonium adducts requires dissolution of the fatty acid in pure acetonitrile. In contrast, protic solvents such as methanol promote the formation of ammonium and alkali metal adducts.

The non-covalent FA complexes were characterized using gas-phase IR spectroscopy in helium nanodroplets, combined with IM-MS and quantum chemical calculations. Gas-phase IR spectroscopy is directly sensitive to the structure and can reveal conformational changes and non-covalent interactions. The spectra are highly resolved due to cryogenic temperatures inside the helium droplets and the absence of interacting solvent molecules in the gas phase. IM-MS on the other hand probes the overall size and shape of the complex, which is expressed by the rotationally averaged CCS. Conformationally flexible, extended FA chains that only interact via the carboxylic acid moiety with a cation are expected to yield larger CCSs than compact adducts with a rigid geometry, in which the FA chain is wrapped around the cation. Computational modeling is an important complement to confirm the three-dimensional gas-phase structures in accordance with the experimental results. It has to be noted, however, that the distribution of conformers in the ion trap of the IR experiment (90 K) is not necessarily the same as in the drift tube of the IM-MS experiment (298 K).

### Sodium adducts [FA + Na]^+^

**Fig. 2:**
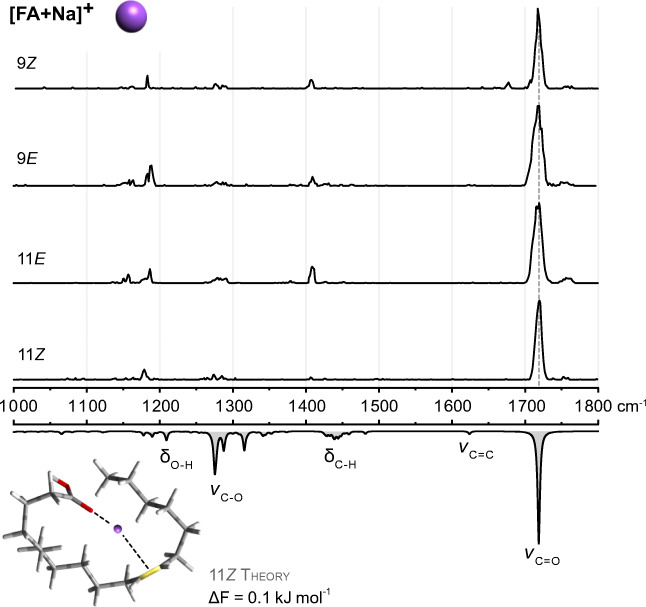
IR spectra of sodium adducts of 18:1 FA C=C isomers. Experimental spectra are stacked above the wavenumber scale and a computed spectrum of the 11*Z* isomer is shown as inverted gray trace at the bottom. The frequency of the intense carbonyl stretching vibration is not influenced by the C=C position and configuration. The region between 1100 and 1300 cm^−1^ displays weak, non-diagnostic bands derived from coupled vibrations of the lipid chain. Selected vibrational modes are annotated in the computed IR spectrum of sodiated FA 18:1 (11*Z*). Non-covalent interactions are depicted by dashed lines and the C=C bond is highlighted in yellow

Alkali metal adducts are usually the most abundant species generated during nano-ESI of FAs in positive ion mode. Accordingly, sodium adducts of 18:1 FAs are readily formed without the addition of sodium salts from methanolic solutions and observed at *m*/*z* 305 in positive ion mode by MS. IM-MS measurements yield almost identical CCSs of the [FA + Na]^+^ ions with a difference of only 1.5% between stereoisomers (ESM Table S1). As expected, the *E* configuration yields more extended lipid chains and therefore slightly increased CCSs compared to *Z* isomers. According to its small size, the impact of the sodium cation on the global size and shape of the adduct seems to be limited, and the observation of comparable CCSs is an indication of very similar three-dimensional geometries of the isomeric adducts. The geometry of the [11*Z* + Na]^+^ adduct was investigated as a representative example of sodiated FAs by conformational sampling and DFT optimization. All computed low-energy conformers display a common structural motif: the Na^+^ cation coordinates both the carbonyl oxygen of the carboxyl group and the C=C bond. However, the expected and desired coordination to the C=C bond does not lead to characteristic vibrations (Fig. 2). The predominant vibration is the intense carbonyl stretching (ν) vibration at the same frequency for all C=C isomers, in agreement with the identical CCSs. Other absorption bands derived from C-H bending (δ) vibrations of the hydrocarbon chain, O-H bending and C-O stretching vibrations, are comparably weak. The C=C stretching vibration itself is predicted between 1600 and 1650 cm^−1^ but not visible in the experimental spectra. In summary, the formation of sodium adducts does not lead to distinct geometries or distinguishable vibrational modes of C=C regio- and stereoisomers in FAs. Therefore, inspired by the previous study on sphingolipids [36], several amines were tested for adduct formation in anticipation of a more important influence on the lipid geometry and gain of spectroscopic information from intrinsic N-H bending vibrations.

### Pyridinium [FA + pyr]^+^ and trimethylammonium adducts [FA + NMe_3_H]^+^

**Fig. 3:**
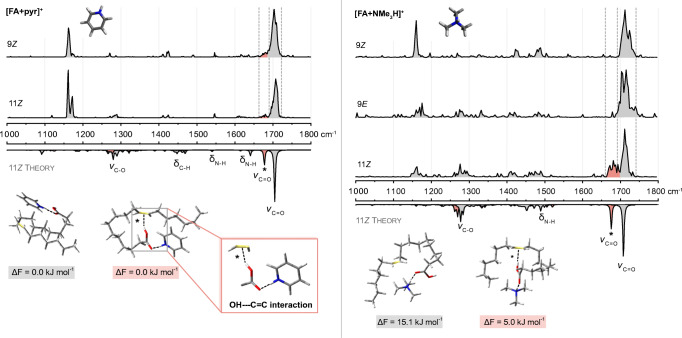
IR spectra of [FA + pyr]^+^ and [FA + NMe_3_H]^+^ adducts of 18:1 FA C=C isomers. Experimental spectra are stacked above the wavenumber scale and computed spectra of the 11*Z* isomer are shown as inverted traces at the bottom. The predominant structural motif confirmed by computational modeling is a cation-carbonyl oxygen interaction. A small fraction of conformers displays an additional interaction between the carboxylic acid proton and the C=C bond, which induces a significant redshift of the carbonyl stretching frequency. In the case of [FA + NMe_3_H]^+^ adducts, the redshift is only observed for the 11*Z* isomer but not for 9*Z* and 9*E*

Despite their significantly different shapes, pyridinium and trimethylammonium share two common characteristics: both contain a protonated tertiary amine and exhibit a similar mass. As a consequence, [FA + pyr]^+^ and [FA + NMe_3_H]^+^ adducts are observed upon nano-ESI of acetonitrile solutions at *m*/*z* 362 and 342, respectively. The single proton on the tertiary amines can interact with the carboxyl group or the C=C bond of the FA but not with both simultaneously. Sampling of the conformational space confirmed unambiguously that both pyridinium and trimethylammonium interact exclusively with the carbonyl oxygen and not with the less nucleophilic C=C bond (ESM Tables S3–S7). However, both theory and experimental IR spectra confirm that a small fraction of conformers displays an indirect hydrogen-C=C interaction: while the cation is coordinated to the carbonyl oxygen, the carboxyl proton interacts with the C=C bond (distance 2.1–2.5 Å). This interaction induces a significant redshift of the carbonyl stretching frequency of 20–30 cm^−1^ and can therefore be readily observed as an additional shoulder next to the dominant C=O band in the experimental spectra (Fig. 3). The pyridinium adducts of the 9*Z* and 11*Z* regioisomers display only a very weak side band besides the main carbonyl vibration. The main vibration frequency is slightly shifted between the isomers, but otherwise the spectra are not distinguishable. In the case of trimethylammonium adducts, however, a difference between conformer distributions can be observed. While the 9*Z* and 9*E* isomer only show a carbonyl stretching vibration at the expected frequency, the spectrum of the 11*Z* isomer also displays a pronounced, redshifted side band attributed to the OH--C=C interaction as observed for pyridinium adducts. Compared with the spectra of sodiated FAs, the relative intensity of lipid chain vibrations is increased for both cations, but the N-H bending frequencies and vibrations in the lower wavenumber region are not diagnostic for C=C isomers. Also, the region below 1200 cm^−1^ is not well modeled by the employed level of theory. CCSs are identical for isomeric pyridinium or trimethylammonium adducts within the accuracy of the measurement (ESM Table S1). The arrival time distributions (ATDs) of the adducts are broad compared to the ATDs of sodium adducts, which agrees with the assumption that the dangling lipid chain has a large conformational freedom if there is only one point of interaction with the cation.

In conclusion, the investigation of pyridinium and trimethylammonium adducts yielded interesting results that contradict the previous assumption that both cations only interact with the carbonyl oxygen and therefore cannot influence the overall conformation significantly. Instead, an interaction of the carboxyl OH with the C=C bond is observed, which gives rise to a characteristic shift of the C=O vibration. In addition, the broad ATDs show that a multitude of conformers are present at room temperature.

### Dimethylammonium adducts [FA + NMe_2_H_2_]^+^

**Fig. 4:**
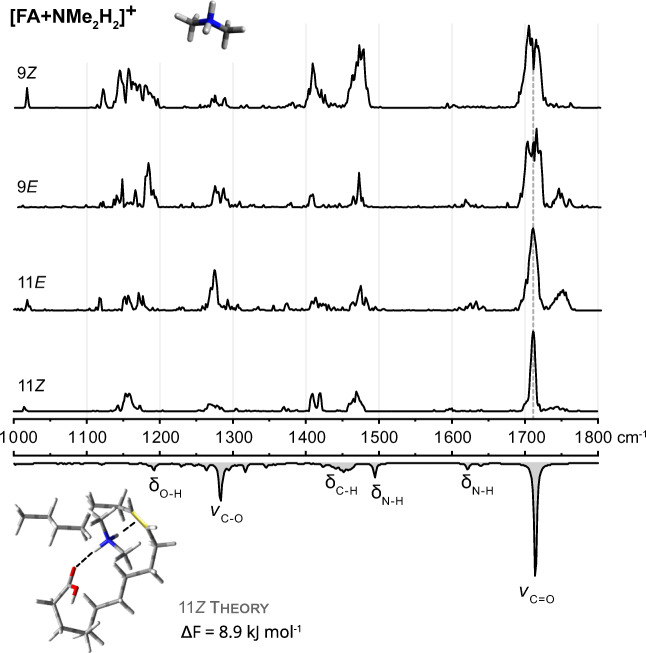
IR spectra of [FA + NMe_2_H_2_]^+^ adducts of 18:1 FA C=C isomers. Experimental spectra are stacked above the wavenumber scale and a computed spectrum of the 11*Z* isomer is shown as inverted gray trace at the bottom. The isomers yield very detailed but similar band patterns and an overall increased wealth of absorption bands due to N-H and CH_3_ bending vibrations of the dimethylammonium cation. C=C positions (9 and 11) can be distinguished by the shape of the carbonyl band. *E* and *Z* isomers can be distinguished by the intensity of blueshifted carbonyl bands, which are more pronounced in the spectra of *E* isomers. Computed structures display mainly a bridged motif, as shown for FA 18:1 (11*Z*)

Advancing from trimethyl- to dimethylammonium increases the number of electrophilic hydrogen atoms that can possibly interact with the lipid chain by one. In principle, the cation should be able to interact with two nucleophilic groups at the same time. This assumption was confirmed by theoretical modeling, which yielded a simultaneous interaction of the cation with the C=C bond and carbonyl oxygen as the main and energetically most favorable structural motif. In some computed conformers of 9*E* and 11*E*, however, dimethylammonium only interacts with the carbonyl oxygen (ESM Tables S9–S10), which does not induce a significant change of the C=O stretching frequency. To confirm the theoretical predictions, IR spectra of [FA + NMe_2_H_2_]^+^ adducts (*m*/*z* 328) were measured for all four C=C isomers (Fig. 4). The wealth of spectral features clearly increases compared to the previous spectra. In particular, intense C-H and N-H bending vibrations are present between 1400 and 1500 cm^−1^ and the fingerprint region below 1200 cm^−1^ exhibits distinct absorption patterns. The spectral region, which is most apparently affected by the C=C location and configuration, is the region of carbonyl stretching vibrations. The shape of the carbonyl band is different for the C=C positions 9 and 11. In addition, the *E* isomers display, contrary to the *Z* isomers, a visibly blueshifted side band adjacent to the main carbonyl band. Computational sampling of low-energy conformers does not lead to structures with such blueshifts. Instead, these structures are probably derived from higher-energy conformers due to incomplete lipid folding into a global minimum of the potential energy surface (PES). The difference between *E* and *Z* fits with the observation that some *E* isomers sampled by CREST are not completely folded into the bridged motif. It could be that *Z* isomers are more easily pre-folded due to the bend in the lipid chain, which facilitates wedging in the cation between the carbonyl oxygen and C=C bond. Differences between *E* and *Z* isomers were also confirmed by IM-MS. The CCSs of *E* isomers are 2–4% larger than those of *Z* isomers (ESM Table S1). In addition, the regioisomers 9*Z* and 11*Z* show a CCS difference of 3 Å^2^, which is in perfect agreement with theoretical predictions (ESM Fig. S7).

Overall, dimethylammonium adducts of C=C isomers are much easier to distinguish by IR spectroscopy and IM-MS than trimethylammonium adducts. Regioisomers can be discriminated by the different shapes of the carbonyl band, whereas stereoisomers differ by the intensity of a blueshifted side band and exhibit a significant CCS difference. However, the spectra and CCSs are too similar to allow an unambiguous identification of C=C isomers in isomeric mixtures.

### Ammonium adducts [FA + NH_4_]^+^

Ammonium adducts of 18:1 FAs (*m*/*z* 300) are readily formed from methanolic solutions containing MS-compatible ammonium acetate. The small ammonium cation is considerably less bulky than tri- and dimethylammonium, which leads to a significant decrease of CCS. Sampling of the conformational space yielded bridged motifs for all isomers, in which ammonium interacts with both the carbonyl oxygen and the C=C bond via two of the four available electrophilic hydrogens (ESM Tables S12–S15). Similar to [FA + Na]^+^ adducts, the CCSs of [FA + NH_4_]^+^ adducts are identical between regioisomers but slightly different between stereoisomers: the CCSs of *E* isomers are roughly 2% larger than the CCSs of the more compact *Z* isomers (ESM Table S1). Overall, the CCSs are very similar between C=C isomers because of the identical structural motif and small size of the cation.

**Fig. 5:**
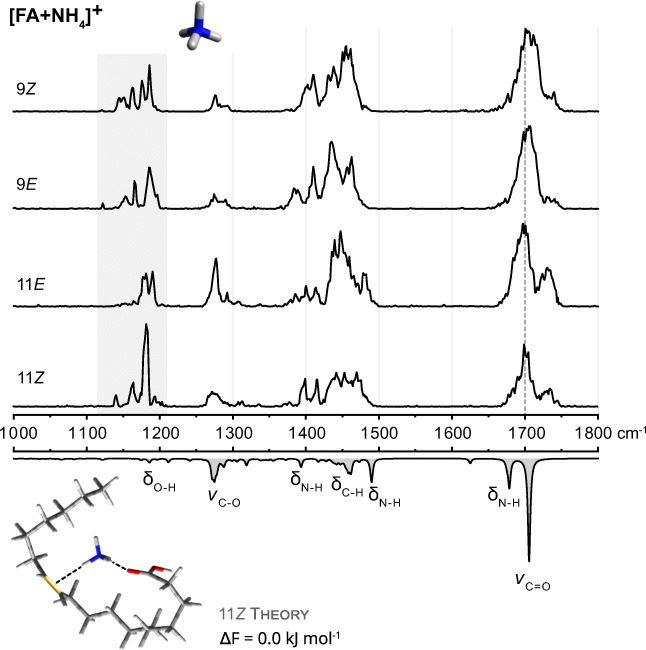
IR spectra of [FA + NH_4_]^+^ adducts of 18:1 FA C=C isomers. Experimental spectra are stacked above the wavenumber scale and a computed spectrum of the 11*Z* isomer is shown as inverted gray trace at the bottom. The coordination of ammonium cations induces a plethora of absorption bands between 1400 and 1500 cm^−1^. The carbonyl stretching frequency is slightly shifted depending on the C=C position. Additionally, the C=C isomers display distinct patterns in the region between 1100 and 1200 cm^−1^, which is highlighted in gray. The computed low-energy conformers exhibit a bridged motif, as depicted for FA 18:1 (11*Z*)

In contrast to the spectra of sodium adducts, the IR spectra of ammonium adducts show a multitude of vibrational modes (Fig. 5). The carbonyl stretching vibration is no longer the only strong band but many other spectral features, such as C-H and N-H bending vibrations are visible. The most diagnostic vibrations are located between 1100 and 1200 cm^−1^. The absorption band patterns in that region are diagnostic for the assignment of single isomers but the band positions are not distinct enough to deconvolute IR spectra of isomeric mixtures. The carbonyl bands are very broad, indicating that a multitude of conformers contributes to the spectra. This is in agreement with the conformational flexibility and absence of bulky methyl groups. The mean C=O stretching frequency is slightly shifted between position 9 and 11.

As already observed for dimethylammonium adducts, the isomers exhibit a blueshifted band adjacent to the main carbonyl band with varying intensity. Its origin was exemplarily explored for the [11*E* + NH_4_]^+^ adduct because the band is most intense in the corresponding experimental spectrum. As all conformers generated by CREST exhibit the same interaction motif and their C=O stretching frequencies coincide with the main experimental absorption band, other conformers were generated on an empirical basis to test the influence of different interaction motifs on the carbonyl band (ESM Fig. S10). If the ammonium cation interacts only with the carbonyl oxygen, the carbonyl band is redshifted, and even more so if the OH group simultaneously interacts with the C=C bond, as observed for trimethylammonium- and pyridinium adducts. Severely blueshifted carbonyl bands above 1800 cm^−1^ are obtained if the ammonium cation donates electron density into the C=O bond by interacting with the OH group. The experimentally observed, less drastic blueshift coincides with a conformer in which the ammonium cation interacts with the C=C bond but not with the carboxyl group. However, all empirically generated conformers are significantly higher in energy than the dominant bridged structural motif. Accordingly, no conformer with a blueshifted carbonyl stretching vibration was found for any kind of FA adduct during the automated conformer sampling. One explanation for the experimental observation of energetically unfavored conformers can be kinetic trapping of conformers in which the lipid chains are not yet ideally folded. Kinetically trapped conformers cannot reach the global minimum of the PES due to insufficient internal energy to overcome energetic barriers after cooling in the ion trap. This effect is most obvious in the 11*E* isomer: in the spectra of dimethylammonium, ammonium, and even sodium adducts of *trans*-vaccenic acid, blueshifted side bands are clearly visible. One possible explanation is that the lipid chains in the *E* isomer are not pre-folded as the hydrocarbon chains of the *Z* isomers, which are already naturally bent. In addition, the distance between the carbonyl oxygen and C=C bond, which must approach each other to form bridged structures, is larger than in oleic and elaidic acid. The relative intensity of the blueshifted band thus depends on both C=C location and configuration and consequently facilitates distinction of C=C isomers.

## Discussion

In the present study, we assessed the impact of on non-covalent adducts on the gas-phase structures of isomeric mono-unsaturated FAs. Our results demonstrate that coordinating cations induce specific three-dimensional conformations of the lipid chain. Those can result in distinct vibrational modes observable by IR spectroscopy, and different overall shapes translating into different CCSs. The geometry of the non-covalent complex depends on the nature of the cation, particularly on the number of available H-atoms that interact with the carboxyl group and the C=C bond via intermolecular charge-olefin interactions. Interestingly, the N-H bending vibrations of interacting amines are not diagnostic for C=C isomers, contrary to previous observations reported for sphingolipids [36]. However, C=C isomers can induce diagnostic variations in the carbonyl stretching vibrations. These include redshifts in [FA + NMe_3_H]^+^ adducts due to distinct structural motifs in low-energy conformers, different shapes and blueshifts of the carbonyl bands in [FA + NMe_2_H_2_]^+^ complexes, and frequency shifts in [FA + NH_4_]^+^ adducts depending on the C=C location. Overall, the spectral wealth and thus the information content increases from sodium adducts, where no characteristic absorptions are obtained, up to ammonium adducts that yield spectroscopic fingerprints with distinguishable band patterns for each C=C regio- and stereoisomer. However, none of the band positions is diagnostic enough to discriminate isomeric mixtures from biological sources and therefore further studies are needed to assess other types of coordinating cations that allow a more straightforward distinction of isomers.

## Supplementary information

ESM 1 (PDF 2462 kb)

ESM 2 (PDF 4252 kb)

## Data Availability

All data generated or analyzed during this study are included in this published article and its supplementary information files.

## Code availability

Custom code generated during the current study is available from the corresponding author on reasonable request.
