# Supplementary material for: Non-covalent double bond sensors for gas-phase infrared spectroscopy of unsaturated fatty acids
Source: Anal Bioanal Chem. 2021 May 6;413(14):3643–53. doi: 10.1007/s00216-021-03334-3 (PMC8141490; doi:10.1007/s00216-021-03334-3)
Supplement: Supplementary file 1 — (PDF 2462 kb) [file 216_2021_3334_MOESM1_ESM.pdf]

Supplementary Information

Non-Covalent Double Bond Sensors for Gas-Phase Infrared Spectroscopy of Unsaturated Fatty Acids

Carla Kirschbaum,[a,b] Kim Greis,[a,b] Maike Lettow,[a,b] Sandy Gewinner,[b] Wieland Schöllkopf,[b] Gerard Meijer,[b] Gert von Helden,[b] and Kevin Pagel[a,b]*

a. Institut für Chemie und Biochemie, Freie Universität Berlin, 14195 Berlin, Germany
b. Fritz-Haber-Institut der Max-Planck-Gesellschaft, 14195 Berlin, Germany

Correspondence to: kevin.pagel@fu-berlin.de

Table of Contents

Ion Mobility-Mass Spectrometry 3
Computed IR Spectra and Structures 4
Sodium Adducts $[FA+Na]^+$ 4
Pyridinium Adducts $[FA+pyr]^+$ 5
Trimethylammonium Adducts $[FA+NMe_3H]^+$ 8
Dimethylammonium Adducts $[FA+NMe_2H_2]^+$ 11
Ammonium Adducts $[FA+NH_4]^+$ 16
Computation of Carbonyl Stretching Vibrations in $[11E+NH_4]^+$ 20
References 21

Ion Mobility-Mass Spectrometry

Table S1: CCSs of non-covalent fatty acid adducts obtained from DT-IM-MS measurements (helium, 4.1–4.2 mbar). The values are given in Å^2 (± 1 %). Theoretically predicted CCSs are indicated in brackets behind the experimental values. Several combinations were not measured (n. m.)

Sample	**$[FA+Na]^+$**	**$[FA+pyr]^+$**	**$[FA+NMe_3H]^+$**	**$[FA+NMe_2H_2]^+$**	**$[FA+NH_4]^+$**
FA 18:1 (**9*Z***)	**120**	**135** (131)	**136** (134)	**133** (132)	**124** (123)
FA 18:1 (**11*Z***)	**120** (117)*	**136** (130)	**135** (130)	**130** (128)	**124** (123)
FA 18:1 (**9*E***)	**122**	n. m.	**136** (135)	**136** (135)	**127** (126)
FA 18:1 (**11*E***)	**122**	n. m.	n. m.	**135** (135)	**127** (125)

*Quantum chemical calculations of sodium adducts were only carried out for the 11*Z* isomer.

Computed IR Spectra and Structures

Sodium Adducts [FA+Na]$^+$

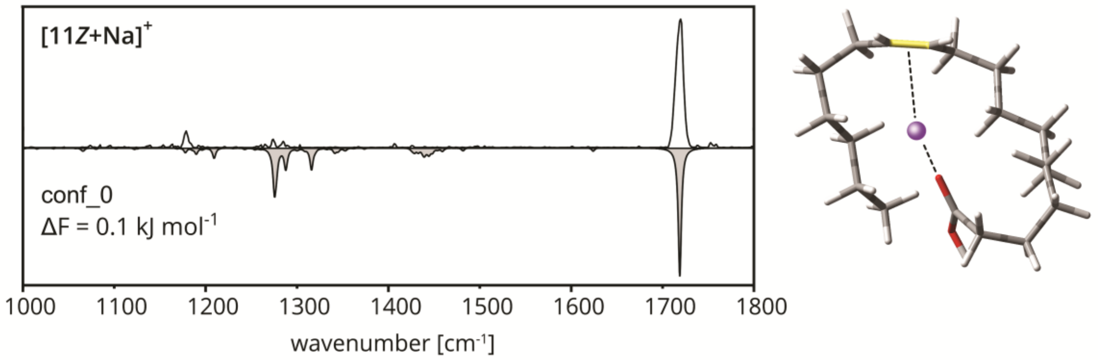

Figure S1: Experimental (top) and computed (bottom, inverted) IR spectra of the [11*Z*+Na]$^+$ adduct. The three-dimensional structure of the corresponding conformer is depicted on the right. Interactions between the sodium cation and the double bond and carbonyl-oxygen are visualized by dashed lines.

Table S2: List of computed conformers of the [11*Z*+Na]$^+$ adduct sampled by CREST [1] and re-optimized at B3LYP+D3/6-311+G(d,p) level of theory in Gaussian 16 [2]. The conformers are numbered according to the CREST conformational search (conf_XX) and ordered by ascending harmonic free energy ΔF at 90 K. The energetics are relative to the lowest-energy conformer and displayed in kJ mol^{-1}. Selected distances (d) are given in Å. CCSs were calculated in helium at 25 °C based on the trajectory method (TM) using HPCCS [3] (Input parameters: 1 10 60 750 1000 298.15 1).

conformer	$\Delta F_{90\,K}$ [kJ mol^{-1}]	d(Na-C=C) [Å]	d(Na-O=C) [Å]	d(Na-OH) [Å]	$^{TM}CCS_{He}$ [Å^2]
conf_21	0	2.91	2.16	4.12	116
**conf_0**	0.1	2.79	2.21	3.45	115.3
conf_97	0.8	2.76	2.17	4.07	115.5
conf_38	0.9	2.76	2.17	4.07	115.5
conf_16	1.7	2.88	2.18	3.61	116.2
conf_41	1.8	2.8	2.17	3.74	115.2
conf_22	2.4	2.8	2.19	3.72	116.5
conf_72	2.9	2.76	2.19	3.87	115.9
conf_2	5.3	2.84	2.3	2.65	115.8
conf_120	6.6	2.75	2.16	3.95	122.7
conf_36	7.3	2.73	2.18	3.69	116.9
conf_12	8.2	2.82	2.21	3.5	116.8
conf_100	8.7	2.76	2.29	2.68	123.5
conf_184	9.5	2.86	2.19	3.74	123.3
conf_139	9.7	2.65	2.2	3.75	114.3
conf_26	10	2.77	2.21	3.47	117.6
conf_77	10.1	2.9	2.21	3.47	113
conf_47	10.3	2.77	2.18	3.64	114.2
conf_25	10.8	2.83	2.2	3.71	117.4
conf_68	11.2	2.72	2.18	3.86	114.1
conf_128	12.1	2.73	2.2	3.44	118

Pyridinium Adducts [FA+pyr]$^+$

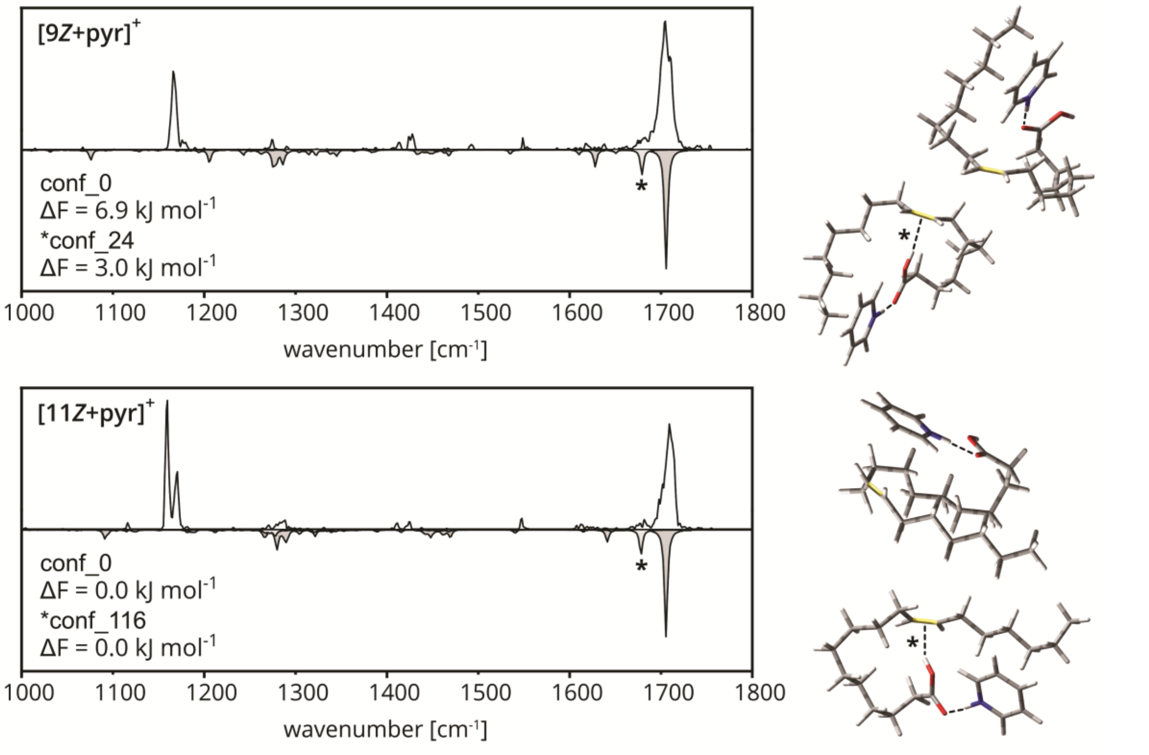

Figure S2: Experimental and computed IR spectra of non-covalent [FA+pyr]$^+$ adducts. The top and bottom panels show the spectra of [9*Z*+pyr]$^+$ and [11*Z*+pyr]$^+$ adducts, respectively. The three-dimensional structures of the corresponding conformers are depicted on the right, and their computed IR spectra are represented as inverted grey traces below the experimental spectra. Interactions between the pyridinium cation and the carbonyl-oxygen are visualized by dashed lines. Interactions between the hydroxyl group and the double bond (*) induce a significant redshift of the carbonyl stretching vibration.

Table S3: List of computed conformers of the [9*Z*+pyr]$^+$ adduct re-optimized at B3LYP+D3/6-311+G(d,p) level of theory in Gaussian 16. Interactions between the hydroxyl group and the double bond are marked by asterisks (*).

conformer	$\Delta F_{90\,K}$ [kJ mol^{-1}]	d(NH-O=C) [Å]	d(OH-C=C) [Å]	$^{TM}CCS_{He}$ [Å^2]
conf_1	0	1.64	7.68	129.8
**conf_24**	3	1.6	**2.16 ***	135.1
conf_46	4.5	1.64	7.38	130.1
**conf_0**	6.9	1.66	7.05	133.2
conf_45	9.6	1.66	7.01	125.5
conf_83	10.1	1.69	7.53	124.5
conf_81	10.2	1.67	6.92	133.7
conf_13	10.9	1.67	7.06	131.5
conf_67	12.4	1.65	8.86	140.2
conf_10	12.5	1.66	6.91	138.9
conf_20	12.8	1.63	3.05	131.2
conf_124	13.6	1.68	7.06	140.2
conf_14	13.9	1.66	6.74	134.1
conf_101	13.9	1.65	6.04	140.7
conf_8	14.8	1.63	3.01	135.8

conf_94	15.6	1.64	4.52	132.7
conf_51	16.3	1.63	4.64	136.6
conf_73	16.3	1.63	5.76	135.5
conf_39	16.4	1.66	6.99	135.1
conf_87	16.6	1.66	4.56	134.5
conf_119	18.3	1.65	6.61	141.8
conf_147	20.6	1.66	4.7	135.4
conf_103	20.8	1.66	4.42	139.2
conf_120	21.2	1.65	4.37	139.3

Table S4: List of computed conformers of the [11*Z*+pyr]$^+$ adduct re-optimized at B3LYP+D3/6-311+G(d,p) level of theory in Gaussian 16. Interactions between the hydroxyl group and the double bond are marked by asterisks (*).

conformer	ΔF $_{90\,K}$ [kJ mol^{-1}]	d(NH-O=C) [Å]	d(OH-C=C) [Å]	$^{TM}CCS_{He}$ [Å ^{2}]
**conf_0**	0	1.64	6.47	125.6
**conf_116**	0	1.62	**2.14 ***	137
conf_38	0.7	1.65	6.33	129.5
conf_1	2.4	1.64	6.4	126.1
conf_2	4.1	1.63	6.73	127.8
conf_4	4.7	1.65	7.46	126.4
conf_9	4.8	1.64	8.3	124.6
conf_15	5.7	1.64	6.63	124
conf_105	6.4	1.7	7.25	137.6
conf_31	8.3	1.64	6.34	125.1
conf_77	9.7	1.65	6.25	136
conf_107	10.1	1.65	6.11	137.7
conf_193	10.5	1.7	7.39	132.8
conf_21	11	1.66	4.61	134.3
conf_74	11.2	1.65	5.38	138.4
conf_88	12.1	1.66	6.47	141.4
conf_195	12.9	1.65	6.11	133.3
conf_191	13.3	1.62	5.15	129
conf_138	13.5	1.65	6.76	135.6
conf_219	16.8	1.66	7.08	143.3
conf_174	17.1	1.67	5.67	133.9

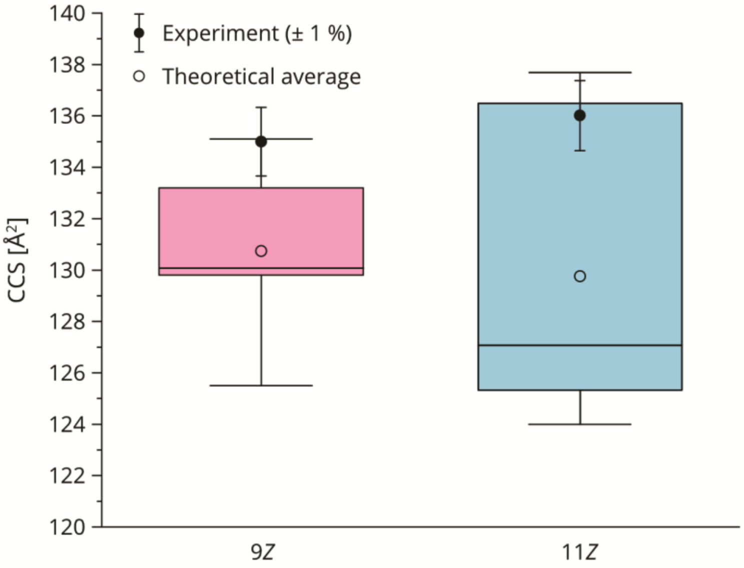

Figure S3: Experimental and computed CCSs of non-covalent $[FA+pyr]^+$ adducts in helium. The CCSs of the computed conformers from Tables S3–4 below a free energy of 10 kJ mol^{-1} are represented in boxplot diagrams. the average CCS is indicated as a circle. Filled circles with error bars of 1 % in the y-direction represent the experimentally determined CCS of each isomer. The computed CCSs are largely underestimated and do not agree well with the experimental values.

Trimethylammonium Adducts [FA+NMe_3H]$^+$

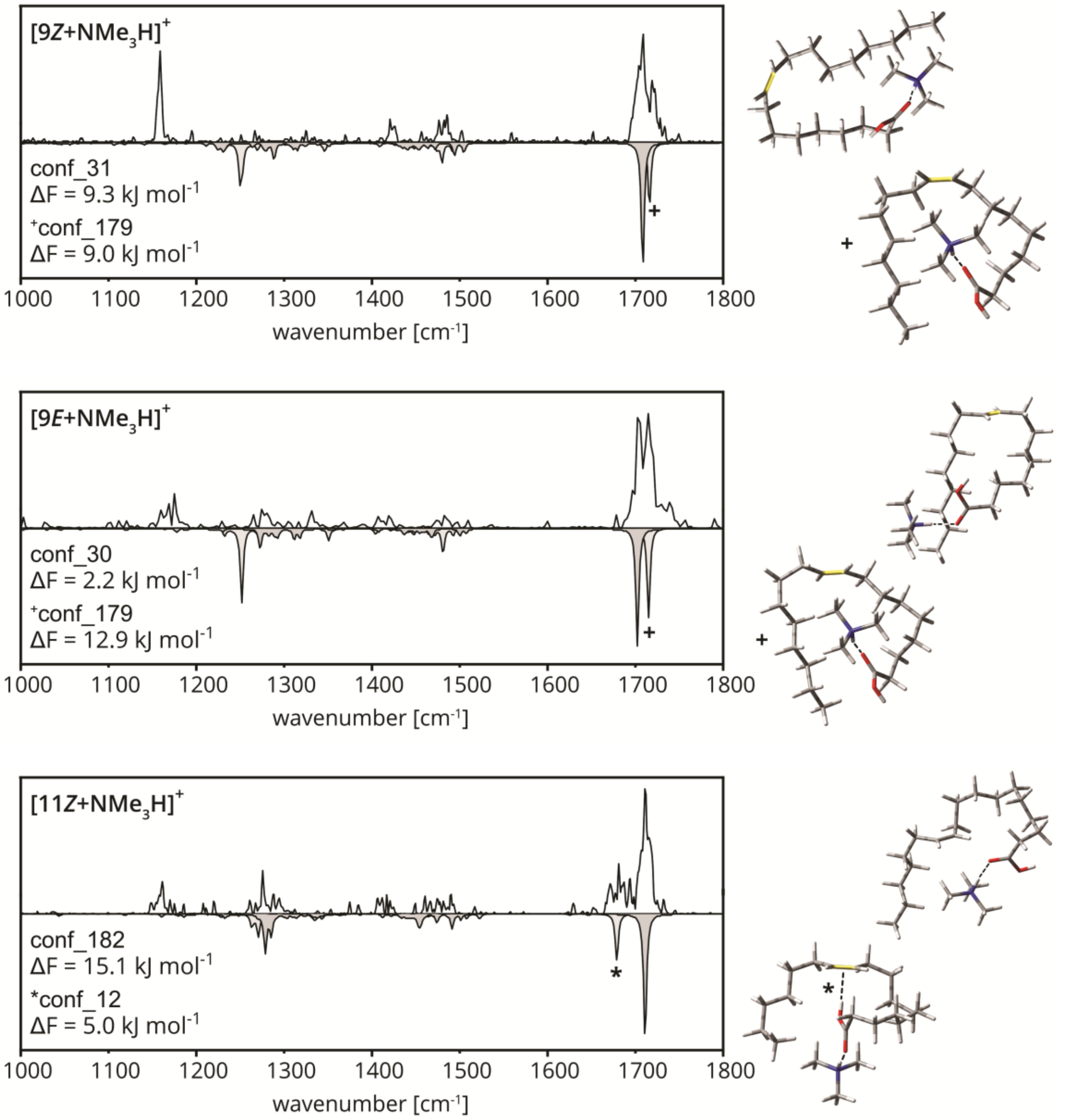

Figure S4: Experimental and computed IR spectra of non-covalent [FA+NMe_3H]$^+$ adducts. The spectra of [9*Z*+NMe_3H]$^+$, [9*E*+NMe_3H]$^+$ and [11*ZE*+NMe_3H]$^+$ adducts are shown, from the top to the bottom panel. The three-dimensional structures of the computed conformers are depicted on the right, and their computed IR spectra are represented as inverted grey traces below the experimental spectra. Interactions between the trimethylammonium cation and the carbonyl-oxygen are visualized by dashed lines. Interactions between the hydroxyl group and the double bond (*) induce a significant redshift of the carbonyl stretching vibration, which is experimentally only observed for the 11*Z* isomer.

Table S5: List of computed conformers of the $[9Z+NMe_3H]^+$ adduct re-optimized at B3LYP+D3/6-311+G(d,p) level of theory in Gaussian 16. The conformers derive from two different rounds of conformational sampling with CREST, indicated by 1 or 2. Interactions between the hydroxyl group and the double bond are marked by asterisks (*).

conformer	$\Delta F_{90\,K}$ [kJ mol^{-1}]	d(NH-O=C) [Å]	d(OH-C=C) [Å]	$^{TM}CCS_{He}$ [Å ^{2}]
conf_16_1	0	1.67	7.89	131.8
conf_48_1	2.2	1.64	**2.29 ***	135.3
conf_192_2	2.2	1.64	**2.3 ***	153.2
conf_0_1	2.4	1.65	**2.4 ***	130.5
conf_111_2	3.2	1.66	6.06	129.1
conf_8_1	4	1.65	**2.46 ***	130.4
conf_43_1	4.9	1.67	7.19	128.1
conf_168_1	5	1.64	**2.29 ***	150
conf_34_1	5.7	1.67	7.82	126
conf_186_2	7.5	1.66	8.46	132.2
conf_96_2	7.9	1.66	5.11	134.8
conf_6_2	8.7	1.67	2.93	127.3
conf_200_2	8.9	1.66	8.25	133.8
**conf_179_2**	9	1.71	7.85	129.7
conf_15_1	9.1	1.67	4.14	134.6
conf_173_2	9.2	1.69	6.43	144.2
**conf_31_1**	9.3	1.7	6.67	134.9
conf_56_1	9.8	1.69	4.56	132
conf_152_2	10.2	1.67	8.41	137
conf_70_1	10.6	1.73	8.75	139.4
conf_129_2	10.6	1.71	6.93	135.2
conf_63_1	10.7	1.67	6.35	136.5
conf_91_1	10.8	1.66	6.37	135.9
conf_134_1	10.8	1.68	9.44	135.1
conf_174_1	12.5	1.68	6.56	137.3
conf_123_2	12.7	1.67	6.92	131.9
conf_26_1	12.8	1.65	**2.4 ***	129.5
conf_122_1	13.6	1.68	4.95	129.3
conf_260_2	15.8	1.69	6.54	126.8
conf_249_2	16.4	1.69	7.2	142.8
conf_152_1	18.2	1.67	3.38	130.2
conf_110_1	18.9	1.67	4.69	132.1

Table S6: List of computed conformers of the $[9E+NMe_3H]^+$ adduct re-optimized at B3LYP+D3/6-311+G(d,p) level of theory in Gaussian 16. Interactions between the hydroxyl group and the double bond are marked by asterisks (*).

conformer	$\Delta F_{90\,K}$ [kJ mol^{-1}]	d(NH-O=C) [Å]	d(OH-C=C) [Å]	$^{TM}CCS_{He}$ [Å ^{2}]
conf_0	0	1.66	**2.45**	130.8
**conf_30**	2.2	1.68	5.31	134.7
conf_1	3.8	1.68	4.83	133.6
conf_2	4.3	1.66	**2.54**	132.5
conf_50	4.8	1.65	**2.13**	136.4
conf_55	6.6	1.7	6.84	142.1
conf_125	7.1	1.67	6.98	130.7
conf_29	7.5	1.66	5.25	132.4
conf_68	8.8	1.66	**2.51**	140
conf_25	9.2	1.69	4.49	130.9
conf_113	9.3	1.68	3.45	138.3
conf_163	9.3	1.69	6.85	139.4
conf_63	11.1	1.66	3.49	130.3
conf_140	11.3	1.68	4.25	130.5
conf_92	11.5	1.68	8.13	133.1
conf_97	12.4	1.69	6.56	140
conf_88	12.7	1.67	4.59	136.3
**conf_179**	12.9	1.69	8.06	129.6
conf_123	14.5	1.67	7.29	132.4
conf_149	15.7	1.66	7.82	134.6
conf_260	21	1.66	6.47	133.9

Table S7: List of computed conformers of the $[11Z+NMe_3H]^+$ adduct re-optimized at B3LYP+D3/6-311+G(d,p) level of theory in Gaussian 16. Interactions between the hydroxyl group and the double bond are marked by asterisks (*).

conformer	$\Delta F_{90\,K}$ [kJ mol^{-1}]	d(NH-O=C) [Å]	d(OH-C=C) [Å]	$^{TM}CCS_{He}$ [Å ^{2}]
conf_2	0	1.67	4.97	125.6
conf_4	2.9	1.69	8.39	127.6
conf_8	3.1	1.68	7.62	127.8
conf_0	4.5	1.68	5.71	127.4
conf_155	4.6	1.64	**2.27 ***	129.5
**conf_12**	5	1.65	**2.12 ***	129.9
conf_55	6.6	1.65	**2.19 ***	126.5
conf_138	7.6	1.64	**2.16 ***	142.3
conf_111	9.8	1.69	3.89	136.1
conf_17	9.9	1.67	6.63	126.9
conf_146	10.4	1.68	5.31	136.7
conf_26	10.6	1.69	4.22	129
conf_116	11.8	1.72	7.57	131.1
conf_91	12	1.69	9.58	135.8
conf_121	12	1.68	5.92	133.2
conf_71	12.6	1.69	5.23	129.6
conf_115	12.7	1.68	8.99	139.8
conf_84	14.3	1.67	7.59	136.3
**conf_182**	15.1	1.71	6.04	134.8

conf_160	16.7	1.64	**2.37 ***	133.2
conf_124	18.5	1.69	5.81	129.8

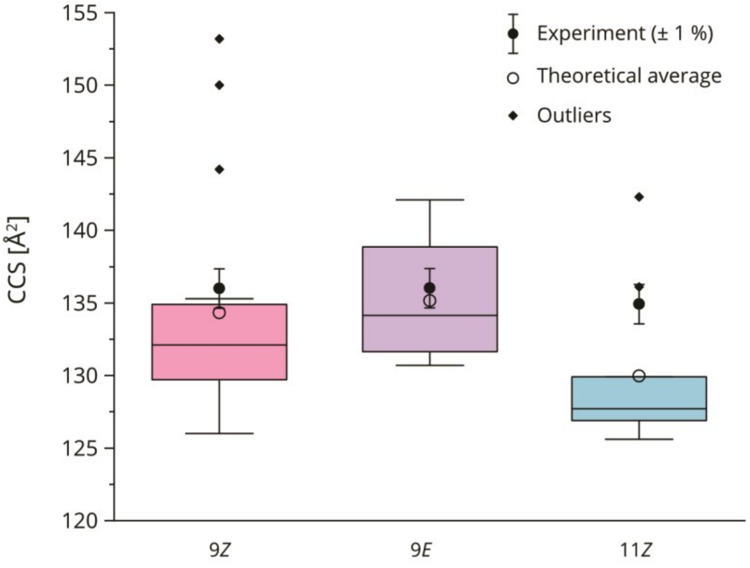

Figure S5: Experimental and computed CCSs of non-covalent $[FA+NMe_3H]^+$ adducts in helium. The CCSs of the computed conformers from Tables S5–7 below a free energy of 10 kJ mol^{-1} are represented in boxplot diagrams. the average CCS is indicated as a circle. Filled circles with error bars of 1 % in the y-direction represent the experimentally determined CCS of each isomer. The CCSs are systematically underestimated but overlap well with the experimental values except for the 11*Z* isomer, which is predicted to exhibit a 4–5 % smaller CCS than experimentally determined. The experimental CCS overlaps with an outlier value, meaning that single conformers with reasonable CCS were found, whereas the whole conformer distribution of $[11Z+NMe_3H]^+$ adducts is not well represented by the small theoretical sampling.

Dimethylammonium Adducts $[FA+NMe_2H_2]^+$

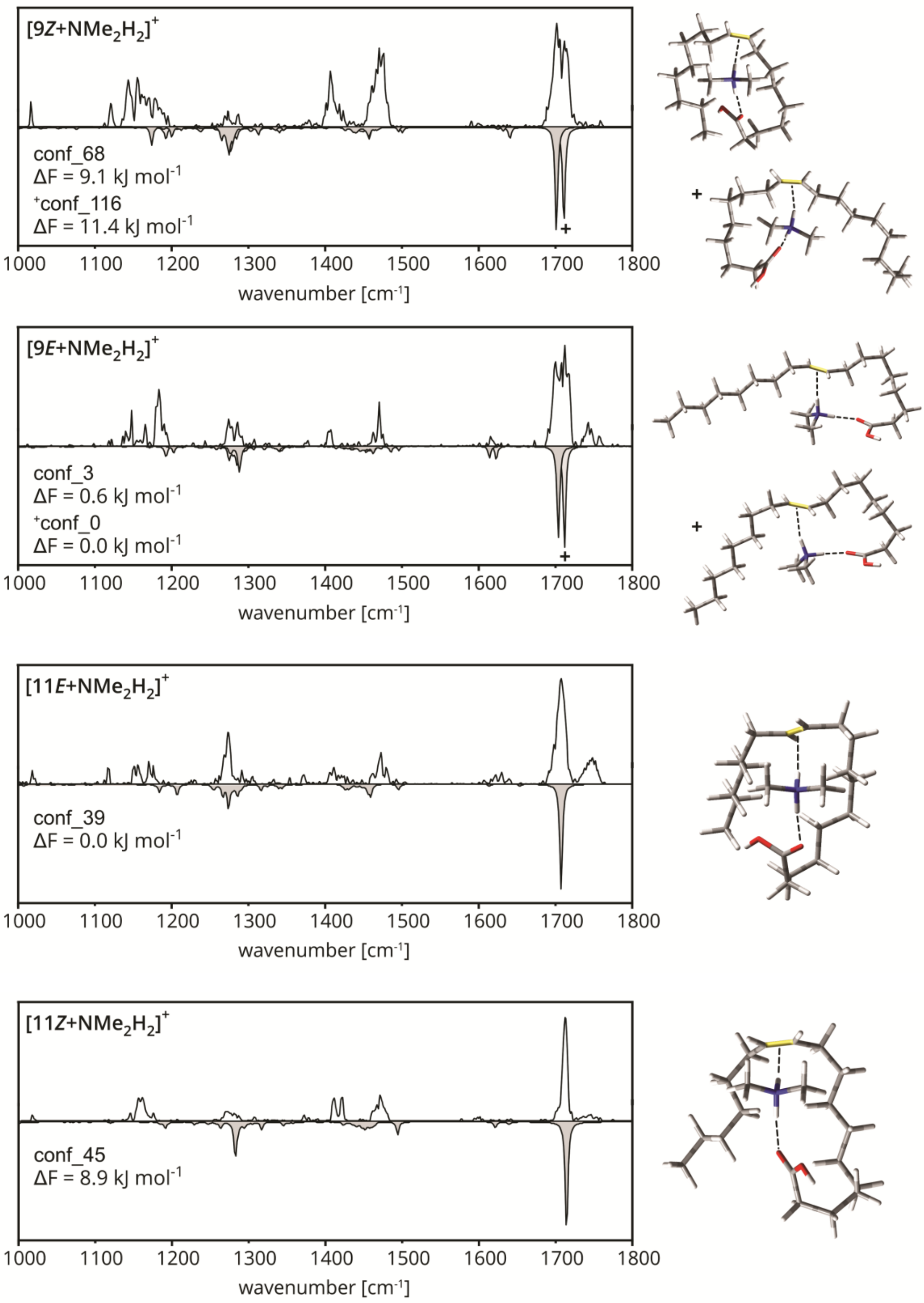

Figure S6: Experimental and computed IR spectra of non-covalent $[FA+NMe_2H_2]^+$ adducts. The spectra of $[9Z+NMe_2H_2]^+$, $[9E+NMe_2H_2]^+$, $[11E+NMe_2H_2]^+$ and $[11Z+NMe_2H_2]^+$ adducts are shown, from the top to the bottom panel. The three-dimensional structures of the computed conformers are depicted on the right, and their computed IR spectra are represented as inverted grey traces below the experimental spectra. Interactions between the dimethylammonium cation and the carbonyl-oxygen and the double bond are visualized by dashed lines.

Table S8: List of computed conformers of the [9*Z*+NMe_2H_2]$^+$ adduct re-optimized at B3LYP+D3/6-311+G(d,p) level of theory in Gaussian 16.

conformer	ΔF $_{90\,K}$ [kJ mol^{-1}]	d(NH-C=C) [Å]	d(NH-O=C) [Å]	$^{TM}CCS_{He}$ [Å^2]
conf_0	0	2.16	1.66	127.1
conf_102	1.9	2.22	1.69	139.4
conf_6	3	2.17	1.66	122.5
conf_10	3.3	2.17	1.67	130.1
conf_1	3.9	2.21	1.69	122.7
conf_22	4	2.25	1.7	126.7
conf_124	4.2	2.14	1.69	140.3
conf_51	4.4	2.23	1.69	135.1
conf_127	4.4	2.31	1.68	126.1
conf_37	4.6	2.15	1.67	127.5
conf_52	6.6	2.13	1.69	142.8
conf_78	7.5	2.15	1.73	143.1
conf_100	8.5	2.41	1.75	131.8
**conf_68**	9.1	2.2	1.71	125.3
conf_163	9.3	2.17	1.72	142.5
conf_158	11.3	2.15	1.67	132.5
**conf_116**	11.4	2.19	1.69	135.6
conf_167	11.6	2.29	1.73	125.9
conf_186	13.1	2.18	1.7	138.4

Table S9: List of computed conformers of the [9*E*+NMe_2H_2]$^+$ adduct re-optimized at B3LYP+D3/6-311+G(d,p) level of theory in Gaussian 16. The lack of interaction between dimethylammonium and the double bond is highlighted (†).

conformer	ΔF $_{90\,K}$ [kJ mol^{-1}]	d(NH-C=C) [Å]	d(NH-O=C) [Å]	$^{TM}CCS_{He}$ [Å^2]
**conf_0**	0	2.33	1.7	138
**conf_3**	0.6	2.17	1.7	142.7
conf_1	0.9	2.33	1.7	133.2
conf_45	2.4	2.12	1.69	127.2
conf_52	2.9	2.17	1.69	130.3
conf_128	3.5	2.2	1.69	137.7
conf_55	4.4	2.11	1.71	143.5
conf_22	4.5	2.17	1.69	143.5
conf_106	6	2.17	1.69	140.4
conf_4	7.4	**3.72 †**	1.7	130.3
conf_64	8.5	**5.28 †**	1.68	124.1
conf_53	8.7	**3.23 †**	1.69	130.7
conf_201	10.4	2.11	1.7	131.5
conf_357	10.4	2.18	1.71	128.6
conf_214	10.5	**7.04 †**	1.67	132
conf_76	10.6	**6.48 †**	1.68	128.5
conf_111	11.1	**5.81 †**	1.65	134.4
conf_260	11.8	2.11	1.7	138.8
conf_140	12.2	**5.31 †**	1.68	131.4
conf_226	13.9	**6.3 †**	1.66	130.7
conf_42	14.3	2.45	1.68	124.2
conf_101	14.8	**4.34 †**	1.65	130
conf_263	18.2	**6.81 †**	1.68	127.5
conf_158	18.6	**5.35 †**	1.66	133.3

Table S10: List of computed conformers of the $[11E+NMe_2H_2]^+$ adduct re-optimized at B3LYP+D3/6-311+G(d,p) level of theory in Gaussian 16. The lack of interaction between dimethylammonium and the double bond is highlighted (†).

conformer	$\Delta F_{90\,K}$ [kJ mol^{-1}]	d(NH-C=C) [Å]	d(NH-O=C) [Å]	$^{TM}CCS_{He}$ [Å^2]
conf_1	0	2.19	1.69	131.8
**conf_39**	0	2.25	1.7	123.2
conf_141	1.3	2.14	1.71	141
conf_37	2.1	2.12	1.7	138.9
conf_129	2.1	2.1	1.71	141.5
conf_3	3.4	2.28	1.68	121.7
conf_225	3.5	2.13	1.7	138.8
conf_60	3.9	2.11	1.71	139.4
conf_125	4.2	2.16	1.72	134.7
conf_0	4.5	2.29	1.69	128.2
conf_143	4.8	2.1	1.71	139.3
conf_4	5	2.29	1.69	132.4
conf_54	5.7	2.2	1.69	131.9
conf_10	6.1	2.18	1.7	138.5
conf_79	6.5	2.24	1.69	138.6
conf_62	8.9	2.22	1.69	136.2
conf_64	11.2	2.35	1.68	127.6
conf_360	11.4	**4.18 †**	1.67	125.5
conf_311	14.7	**6.61 †**	1.68	131
conf_372	15.5	**4.06 †**	1.67	128.1
conf_351	17.3	**4.59 †**	1.66	133.2
conf_322	18.7	**4.43 †**	1.67	131.7
conf_427	19	**6.06 †**	1.65	135.2

Table S11: List of computed conformers of the $[11Z+NMe_2H_2]^+$ adduct re-optimized at B3LYP+D3/6-311+G(d,p) level of theory in Gaussian 16.

conformer	$\Delta F_{90\,K}$ [kJ mol^{-1}]	d(NH-C=C) [Å]	d(NH-O=C) [Å]	$^{TM}CCS_{He}$ [Å^2]
conf_5	0	2.21	1.68	124.1
conf_6	0.8	2.16	1.69	125.5
conf_2	1.3	2.18	1.69	123.3
conf_8	4	2.23	1.72	123.4
conf_27	5.2	2.19	1.71	127.5
conf_1	5.8	2.19	1.67	121.7
conf_36	6.4	2.27	1.71	133
conf_154	7.3	2.17	1.71	137.2
conf_60	7.5	2.13	1.69	128.7
conf_0	8.9	2.18	1.67	123.1
**conf_45**	8.9	2.2	1.69	123.8
conf_95	9.1	2.11	1.7	138.5
conf_24	9.3	2.29	1.7	128.2
conf_56	9.4	2.22	1.72	131.6
conf_58	11.1	2.15	1.72	130.4
conf_31	11.8	2.34	1.72	120
conf_44	11.8	2.23	1.71	136
conf_268	12.6	2.11	1.69	133.6
conf_115	12.8	2.14	1.7	125.9

conf_72	14.3	2.18	1.73	127.8
conf_90	14.9	2.23	1.69	132.8
conf_171	15.7	2.25	1.7	129.3
conf_166	16.1	2.2	1.71	131.2
conf_130	16.6	2.15	1.71	137.3
conf_249	21.8	2.24	1.76	132.8

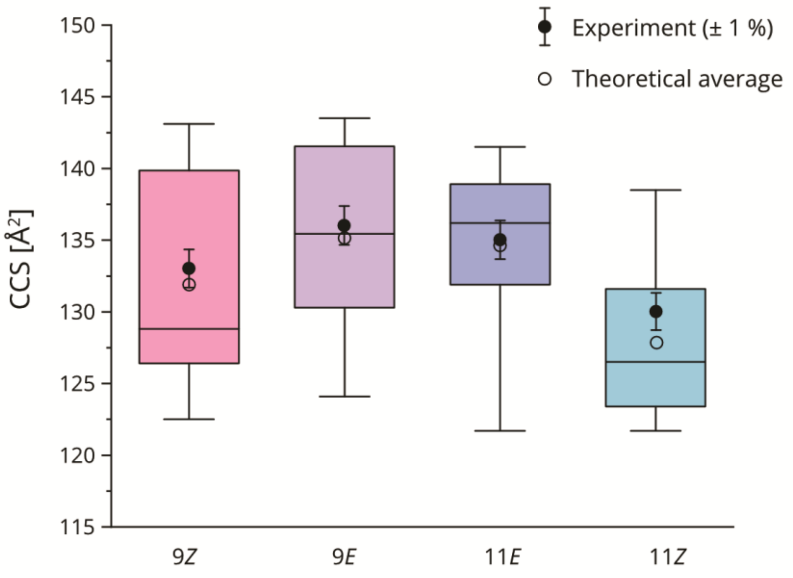

Figure S7: Experimental and computed CCSs of non-covalent $[FA+NMe_2H_2]^+$ adducts in helium. The CCSs of the computed conformers from Tables S8–11 below a free energy of 10 kJ mol^{-1} are represented in boxplot diagrams. the average CCS is indicated as a circle. Filled circles with error bars of 1 % in the y-direction represent the experimentally determined CCS of each isomer. The CCSs are systematically slightly underestimated but overlap well with the experimental values.

Ammonium Adducts $[FA+NH_4]^+$

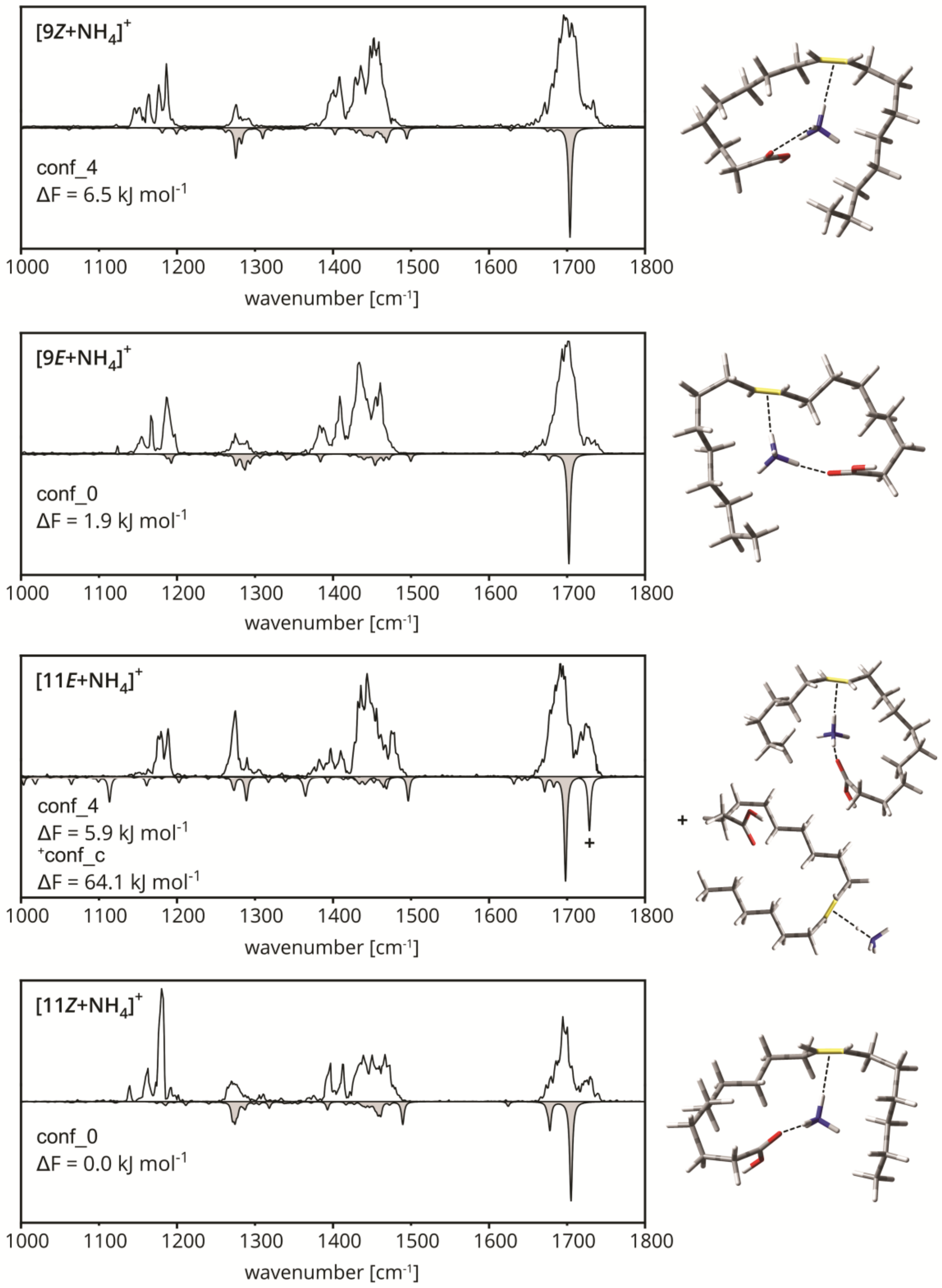

Figure S8: Experimental and computed IR spectra of non-covalent $[FA+NH_4]^+$ adducts. The spectra of $[9Z+NH_4]^+$, $[9E+NH_4]^+$, $[11E+NH_4]^+$ and $[11Z+NH_4]^+$ adducts are shown, from the top to the bottom panel. The three-dimensional structures of the computed conformers are depicted on the right, and their computed IR spectra are represented as inverted grey traces below the experimental spectra. Interactions between the ammonium cation and the carbonyl-oxygen and the double bond are visualized by dashed lines. The blueshifted carbonyl stretching vibrations (most prominent for 11*E*) can be modeled only with energetically unfavored conformers.

Table S12: List of computed conformers of the $[9Z+NH_4]^+$ adduct re-optimized at B3LYP+D3/6-311+G(d,p) level of theory in Gaussian 16.

conformer	ΔF $_{90\,K}$ [kJ mol^{-1}]	d(NH-C=C) [Å]	d(NH-O=C) [Å]	d(NH-OH) [Å]	$^{TM}CCS_{He}$ [Å ^{2}]
conf_0	0	2.2	1.64	3.86	123.2
conf_2	0.3	2.2	1.65	3.34	120.2
conf_11	0.7	2.15	1.64	3.78	116.6
conf_19	1.2	2.24	1.65	4.1	120.3
conf_1	1.3	2.21	1.66	3.24	119.5
conf_37	1.9	2.17	1.63	3.93	122.3
conf_21	2.2	2.16	1.67	3.26	123.4
conf_5	2.7	2.2	1.64	3.82	124.3
conf_9	3.7	2.18	1.63	3.64	117.3
conf_69	4.5	2.16	1.65	3.24	131.3
conf_59	4.9	2.16	1.61	4.25	121.5
conf_85	5.2	2.14	1.63	3.65	131
conf_83	5.8	2.16	1.61	4.6	116.8
**conf_4**	6.5	2.21	1.66	3.94	118
conf_164	6.7	2.19	1.64	4.01	125
conf_93	7.8	2.15	1.61	4.19	116.8
conf_209	8.1	2.11	1.62	3.65	133.2
conf_95	8.3	2.21	1.64	3.79	124.8
conf_207	8.5	2.16	1.6	2.82	116.3
conf_118	9.1	2.25	1.64	3.56	123.2
conf_196	9.5	2.15	1.63	3.42	129.5
conf_247	10.8	2.22	1.64	4.22	118.5

Table S13: List of computed conformers of the $[9E+NH_4]^+$ adduct re-optimized at B3LYP+D3/6-311+G(d,p) level of theory in Gaussian 16.

conformer	ΔF $_{90\,K}$ [kJ mol^{-1}]	d(NH-C=C) [Å]	d(NH-O=C) [Å]	d(NH-OH) [Å]	$^{TM}CCS_{He}$ [Å ^{2}]
conf_19	0	2.21	1.63	3.77	132.1
conf_9	0.1	2.11	1.64	3.68	121.3
conf_10	0.1	2.11	1.61	3.63	121.8
conf_23	0.2	2.18	1.64	4.03	126.1
conf_153	0.8	2.11	1.62	3.56	115.8
conf_76	1.2	2.23	1.64	3.74	125.4
**conf_0**	1.9	2.23	1.64	3.64	124.3
conf_14	3	2.12	1.65	3.53	125.8
conf_11	3.2	2.15	1.63	3.86	121
conf_13	4.3	2.17	1.63	3.85	123.1
conf_24	4.8	2.12	1.62	3.66	133.9
conf_40	4.9	2.18	1.64	4.18	127.1
conf_17	5	2.18	1.63	3.58	129.9
conf_54	6.4	2.12	1.62	3.65	131.8
conf_6	6.6	2.3	1.64	3.78	122.7
conf_88	7.5	2.11	1.62	3.66	133.7
conf_31	8.1	2.19	1.65	4.07	119.8
conf_121	8.7	2.11	1.62	3.81	132.9
conf_111	8.8	2.07	1.62	3.88	135.4
conf_171	8.9	5.6	1.51	3.42	121.4
conf_100	12.1	2.21	1.64	3.14	129.2

Table S14: List of computed conformers of the $[11E+NH_4]^+$ adduct re-optimized at B3LYP+D3/6-311+G(d,p) level of theory in Gaussian 16. The conformers a–e were empirically generated and exhibit unusual interactions.

conformer	$\Delta F_{90\,K}$ [kJ mol^{-1}]	d(NH-C=C) [Å]	d(NH-O=C) [Å]	d(NH-OH) [Å]	$^{TM}CCS_{He}$ [Å^2]
conf_3	0	2.14	1.63	3.72	123.2
conf_118	0.7	2.14	1.64	4.01	130.4
conf_2	1.5	2.18	1.63	3.77	123
conf_117	1.5	2.1	1.65	4.42	119.1
conf_36	2.3	2.11	1.63	3.67	126.5
conf_55	3.1	2.07	1.62	3.66	131.1
conf_18	3.8	2.19	1.63	3.25	125.6
conf_0	4.6	2.15	1.63	3.77	118.7
conf_64	5.1	2.07	1.61	3.68	128
conf_27	5.3	2.11	1.63	3.41	130.3
conf_44	5.3	2.09	1.62	3.28	133.4
conf_68	5.7	2.16	1.64	4.08	123.3
conf_50	5.8	2.14	1.6	3.34	117.3
**conf_4**	5.9	2.14	1.65	3.39	122.3
conf_7	6.4	2.2	1.67	3.33	120.2
conf_161	6.6	2.08	1.61	3.88	130.1
conf_172	7.1	2.13	1.64	3.63	125.3
conf_15	7.5	2.15	1.62	3.33	121.9
conf_192	7.7	2.08	1.63	3.96	129.8
conf_12	8.2	2.3	1.62	3.24	117
conf_92	8.8	2.12	1.63	3.7	124
conf_35	9.2	2.17	1.63	3.26	122.9
conf_224	10.5	2.14	1.62	4.13	131.7
conf_a	10.8	5.57	1.5	3.42	121.7
conf_194	10.9	2.09	1.61	3.8	128.6
conf_238	11.5	2.11	1.62	3.63	124.6
conf_133	11.9	2.15	1.63	3.89	119.2
conf_b	39.8	14.72	1.53	3.35	152.8
conf_d	50.2	2.08	3.96	1.76	121.4
**conf_c**	64.1	1.91	7.75	10.47	129.3
conf_e	89.8	14.2	3.82	1.63	153.5

Table S15: List of computed conformers of the $[11Z+NH_4]^+$ adduct re-optimized at B3LYP+D3/6-311+G(d,p) level of theory in Gaussian 16.

conformer	$\Delta F_{90\,K}$ [kJ mol^{-1}]	d(NH-C=C) [Å]	d(NH-O=C) [Å]	d(NH-OH) [Å]	$^{TM}CCS_{He}$ [Å^2]
**conf_0**	0	2.14	1.66	2.82	122.8
conf_6	1.1	2.11	1.65	2.85	126.5
conf_174	1.4	2.12	1.65	3.29	130
conf_177	1.4	2.12	1.65	3.29	130
conf_53	1.6	2.17	1.63	3.34	115.6
conf_4	3.1	2.11	1.65	3.98	123.7
conf_15	3.9	2.16	1.66	2.98	124
conf_102	4.1	2.11	1.6	3.55	117.1
conf_30	5.1	2.1	1.65	2.87	126.9
conf_132	5.5	2.13	1.64	3.78	121.5

conf_25	5.7	2.15	1.65	3.24	122.4
conf_40	6.6	2.2	1.67	2.59	114
conf_57	7.1	2.14	1.62	3.35	114.7
conf_96	7.5	2.08	1.63	2.89	129.4
conf_184	7.6	2.13	1.63	3.67	128.6
conf_81	7.7	2.11	1.63	3.69	122.6
conf_92	8	2.08	1.63	2.89	124.2
conf_8	8.2	2.12	1.66	4	118.3
conf_27	8.4	2.14	1.66	4.3	119.2
conf_46	9.3	2.12	1.62	3.83	119.1
conf_153	12.2	2.12	1.63	3.5	126

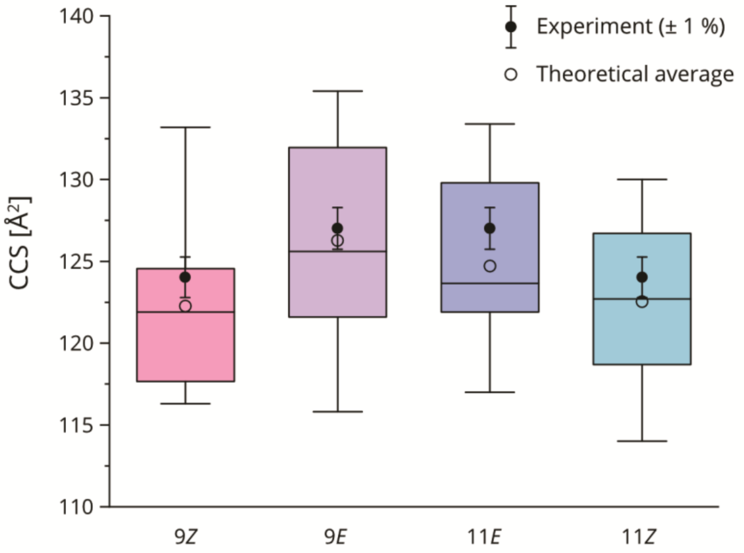

Figure S9: Experimental and computed CCSs of non-covalent $[FA+NH_4]^+$ adducts in helium. The CCSs of the computed conformers from Tables S12–15 below a free energy of 10 kJ mol^{-1} are represented in boxplot diagrams. the average CCS is indicated as a circle. Filled circles with error bars of 1 % in the y-direction represent the experimentally determined CCS of each isomer. The CCSs are systematically slightly underestimated but overlap well with the experimental values.

Computation of Carbonyl Stretching Vibrations in $[11E+NH_4]^+$

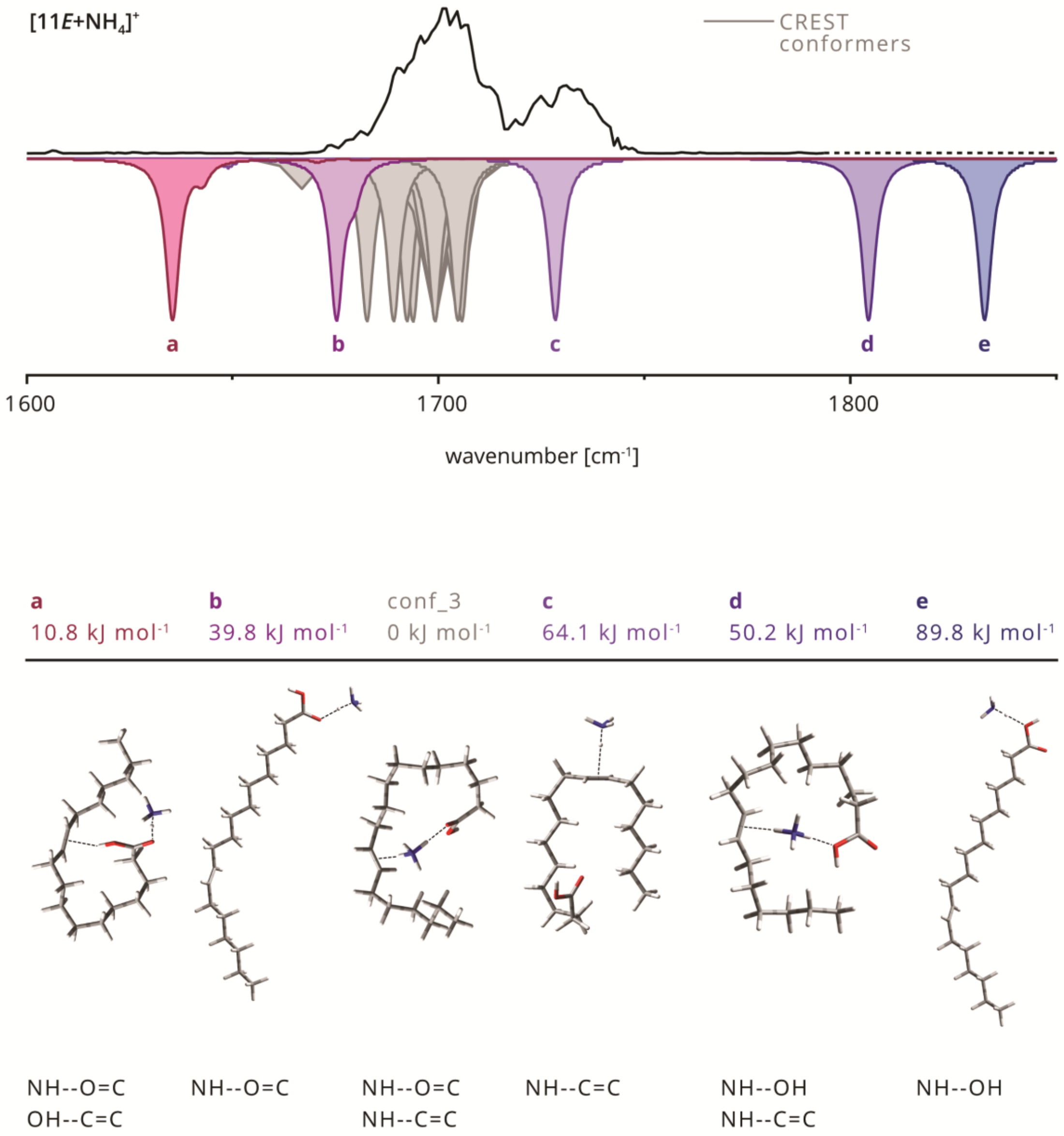

Figure S10: Empirical investigation of the influence of different structure motifs on the carbonyl stretching frequency. All conformers sampled by CREST (grey) coincide with the main absorption band in the experimental spectrum of $[11E+NH_4]^+$ (top). Interactions between the OH group and ammonium increase the electron density in the carbonyl bond and cause a significant blueshift (d,e). The exclusive coordination of ammonium to the carbonyl oxygen causes a redshift (b), which is increased by simultaneous interaction of the OH group with the C=C bond (a). The experimentally observed, blueshifted carbonyl band probably derives from coordination of ammonium to the C=C bond (c), even though the depicted conformer is energetically unfavored. The free energies at 90 K are given relative to the lowest-energy conformer (conf_3) obtained from the CREST search.

References

[1] Pracht P, Bohle F, Grimme S. Automated exploration of the low-energy chemical space with fast quantum chemical methods. Phys Chem Chem Phys. 2020;22:7169-92.

[2] Frisch MJ, Trucks GW, Schlegel HB, Scuseria GE, Robb MA, Cheeseman JR, Scalmani G, Barone V, Petersson GA, Nakatsuji H, Li X, Caricato M, Marenich AV, Bloino J, Janesko BG, Gomperts R, Mennucci B, Hratchian HP, Ortiz JV, Izmaylov AF, Sonnenberg JL, Williams, Ding F, Lipparini F, Egidi F, Goings J, Peng B, Petrone A, Henderson T, Ranasinghe D, Zakrzewski VG, Gao J, Rega N, Zheng G, Liang W, Hada M, Ehara M, Toyota K, Fukuda R, Hasegawa J, Ishida M, Nakajima T, Honda Y, Kitao O, Nakai H, Vreven T, Throssell K, Montgomery Jr. JA, Peralta JE, Ogliaro F, Bearpark MJ, Heyd JJ, Brothers EN, Kudin KN, Staroverov VN, Keith TA, Kobayashi R, Normand J, Raghavachari K, Rendell AP, Burant JC, Iyengar SS, Tomasi J, Cossi M, Millam JM, Klene M, Adamo C, Cammi R, Ochterski JW, Martin RL, Morokuma K, Farkas O, Foresman JB, Fox DJ, Gaussian 16, Rev. A.03, Inc., Wallingford, CT, 2016.

[3] Zanotto L, Heerdt G, Souza PCT, Araujo G, Skaf MS. High performance collision cross section calculation-HPCCS. J Comput Chem. 2018;39:1675-81.
