# Supplementary material for: Non-covalent double bond sensors for gas-phase infrared spectroscopy of unsaturated fatty acids
Source: Anal Bioanal Chem. 2021 May 6;413(14):3643–53. doi: 10.1007/s00216-021-03334-3 (PMC8141490; doi:10.1007/s00216-021-03334-3)
Supplement: Supplementary file 2 — (PDF 4252 kb) [file 216_2021_3334_MOESM2_ESM.pdf]

Supplementary Information_2

Non-Covalent Double Bond Sensors for Gas-Phase Infrared Spectroscopy of Unsaturated Fatty Acids

Carla Kirschbaum,[a,b] Kim Greis,[a,b] Maike Lettow,[a,b] Sandy Gewinner,[b] Wieland Schöllkopf,[b] Gerard Meijer,[b] Gert von Helden,[b] and Kevin Pagel[a,b]*

a. Institut für Chemie und Biochemie, Freie Universität Berlin, 14195 Berlin, Germany

b. Fritz-Haber-Institut der Max-Planck-Gesellschaft, 14195 Berlin, Germany

Correspondence to: kevin.pagel@fu-berlin.de

Contents: XYZ Files of Computed Conformers

11Z_Na 3

9Z_pyr 25

11Z_pyr 56

9Z_NMe3H 82

9E_NMe3H 124

11Z_NMe3H 152

9Z_NMe2H2 178

9E_NMe2H2 203

11E_NMe2H2 233

11Z_NMe2H2 263

9Z_NH4 295

9E_NH4 320

11E_NH4 344

11Z_NH4 380

11Z_Na

conf_21

C	-2.74067500	1.62378800	0.34673300
C	-3.67202200	0.45807400	0.68974500
C	-4.50334800	-0.06693100	-0.48719400
C	-3.66704700	-0.51653000	-1.71312000
C	-2.56089600	-1.45837300	-1.31620100
O	-1.38877700	-1.13378800	-1.24586500
O	-2.88987500	-2.70639800	-0.97166600
H	-3.83890500	-2.86408900	-1.06995900
H	-3.20417300	0.34118700	-2.19815400
H	-4.31935800	-1.00096600	-2.44610200
H	-5.19207100	0.70637400	-0.83775500
H	-5.12880400	-0.89710100	-0.14328300
H	-4.35903000	0.76171800	1.48558500
H	-3.07655300	-0.36188700	1.11139400
C	-1.93205600	2.11006200	1.55603800
C	-0.76647400	3.03950600	1.19222500
C	0.39838200	2.31414000	0.51098500
C	1.55374300	3.22534900	0.09225100
C	2.79399100	2.45991600	-0.39741200
C	2.51209100	1.54916200	-1.56317700
C	3.08498500	0.36606800	-1.83284300
C	4.15895400	-0.34630900	-1.05342200
C	3.67358600	-1.68649000	-0.46615500
C	2.85550700	-1.52256800	0.82446000
C	1.90920900	-2.69799400	1.10191800
C	1.05054800	-2.55448400	2.36441500
C	-0.00532900	-1.44908200	2.27190600
H	0.44511700	-0.45603300	2.17003400
H	-0.68853600	-1.62606500	1.43144800
H	-0.62309800	-1.41048100	3.17109900
H	1.70009700	-2.36920100	3.22590000
H	0.54844900	-3.50614800	2.55760700
H	1.24971200	-2.86948100	0.23500200
H	2.51106400	-3.60874500	1.17283800
H	2.31476300	-0.56358500	0.80469700
H	3.53659100	-1.40388400	1.67281500
H	3.07943900	-2.21262500	-1.22535800
H	4.52866700	-2.33562500	-0.26353700
H	4.55925100	0.28547300	-0.25709400
H	4.98945000	-0.53950200	-1.74004600
H	2.79398100	-0.13083500	-2.75916400
H	1.80439700	1.93943000	-2.29539700
H	3.23136800	1.89787700	0.43308800
NA	0.55904600	-0.49535300	-0.55093100
H	3.55450900	3.18863000	-0.70267100
H	1.20753500	3.90525400	-0.69381400
H	1.84139200	3.85522200	0.93884500
H	0.77993800	1.54604000	1.20024600
H	0.01524600	1.80700800	-0.38626300
H	-1.12319500	3.84479700	0.53990200
H	-0.39018800	3.52356400	2.09857400

H	-2.60361000	2.62195100	2.25150200
H	-1.53890800	1.24356200	2.10308300
H	-2.05938900	1.31674200	-0.45211500
H	-3.32434000	2.45797500	-0.05937400

conf_0

C	-3.42523000	1.32817300	-0.49187600
C	-4.65907200	0.46227400	-0.78041700
C	-4.55062500	-1.03030900	-0.43860300
C	-3.43696500	-1.78596400	-1.20465000
C	-2.05943600	-1.62080600	-0.62472400
O	-1.11005400	-1.13217200	-1.20824100
O	-1.86191500	-2.04953300	0.63448900
H	-2.67156900	-2.41357600	1.01849000
H	-3.39770600	-1.46521200	-2.24551000
H	-3.65717700	-2.85985100	-1.19949400
H	-5.49883500	-1.51013900	-0.69035000
H	-4.43209900	-1.16471900	0.64287700
H	-4.90949200	0.55205800	-1.84279700
H	-5.51562000	0.86824100	-0.23228400
C	-3.01739600	1.39195500	0.98561400
C	-1.88964700	2.39265400	1.26914800
C	-0.51097100	1.97635800	0.74080900
C	0.54323800	3.07607400	0.90242100
C	1.97554100	2.65238800	0.54890900
C	2.14667200	2.19392100	-0.88094800
C	3.12329600	1.40596900	-1.35898100
C	4.26966800	0.82328700	-0.57971600
C	4.58851900	-0.64353400	-0.92016600
C	3.43586000	-1.62732700	-0.68505300
C	2.86750900	-1.61602800	0.73933700
C	1.76573700	-2.65523700	0.98316200
C	1.14112900	-2.54867700	2.37440200
H	1.89785300	-2.67188700	3.15323800
H	0.66525900	-1.57411400	2.52706800
H	0.37732400	-3.31357300	2.52504500
H	2.19048300	-3.65322400	0.83992700
H	0.97497100	-2.57719600	0.22231600
H	3.68429200	-1.79243200	1.44654900
H	2.50318100	-0.61144600	1.01124800
H	3.78083600	-2.63987200	-0.91359700
H	2.64429900	-1.44213100	-1.43036000
H	4.90236400	-0.71184000	-1.96646800
H	5.44915600	-0.95577100	-0.32126900
H	4.10751000	0.94021500	0.49411500
H	5.15979700	1.42244100	-0.80767600
H	3.14563500	1.22297900	-2.43306900
H	1.44767200	2.62162600	-1.59962400
H	2.31531000	1.87939300	1.24858400
NA	0.81332600	-0.24955700	-0.58238300
H	2.64141100	3.50644400	0.72034700
H	0.25552200	3.93109300	0.28113900
H	0.53017800	3.43207600	1.93664000

H	-0.19225200	1.07417600	1.28984300
H	-0.60636300	1.71732900	-0.32258400
H	-2.15413800	3.36317200	0.83425800
H	-1.80887800	2.55428800	2.34853300
H	-3.89405600	1.67797500	1.57641700
H	-2.71644200	0.40369900	1.35226100
H	-2.58734700	0.99187400	-1.10969600
H	-3.64886200	2.34563800	-0.82990700

conf_97

C	-2.96244000	1.70548300	0.23171900
C	-4.03083400	0.78915800	0.84368500
C	-3.79432800	-0.72259100	0.70101100
C	-3.85880900	-1.24054300	-0.74267900
C	-2.60995800	-1.00399100	-1.55784200
O	-1.49412400	-0.93050000	-1.07970900
O	-2.73531100	-0.91584700	-2.88510000
H	-3.65962500	-0.98266900	-3.16072000
H	-4.72846100	-0.82450300	-1.26253200
H	-3.99537100	-2.32959600	-0.74015000
H	-4.56562500	-1.24495800	1.27161000
H	-2.83859900	-1.01789000	1.14103500
H	-5.00245100	1.03148900	0.39963500
H	-4.12413800	1.02044600	1.90984800
C	-1.61412800	1.66265300	0.95705000
C	-0.51228500	2.44305900	0.23275200
C	0.82193000	2.49034900	0.99058400
C	1.95346100	3.22646800	0.24033400
C	3.05894700	2.31080600	-0.31226500
C	2.62053700	1.36608000	-1.39890700
C	3.00328000	0.08828600	-1.56358300
C	3.94111600	-0.69789200	-0.68599600
C	3.58918300	-2.19060800	-0.55702300
C	2.18435000	-2.48798100	-0.01637800
C	1.86154000	-1.83211400	1.33206900
C	0.46211200	-2.16396500	1.86741200
C	0.12229500	-1.42137500	3.16064400
H	-0.87445800	-1.68482800	3.52105600
H	0.83600100	-1.66595300	3.95116600
H	0.14882900	-0.33575800	3.02067800
H	0.39758200	-3.24386700	2.03138700
H	-0.30478300	-1.95356700	1.10739100
H	2.60845100	-2.14752800	2.06765600
H	1.99177800	-0.73924900	1.27962500
H	2.06773600	-3.57074400	0.08749700
H	1.43624900	-2.21630900	-0.77825300
H	3.69660900	-2.67206700	-1.53416400
H	4.32918100	-2.66043700	0.09754500
H	4.01850900	-0.24036000	0.30281500
H	4.94589800	-0.62595700	-1.11997200
H	2.67143000	-0.42314000	-2.46810000
H	2.00322800	1.81146000	-2.17997800
H	3.52573200	1.76473400	0.51201400

NA	0.42374900	-0.16169600	-0.42440300
H	3.84844200	2.94649100	-0.73240800
H	1.52624900	3.81347400	-0.57987700
H	2.42479200	3.94843000	0.91027100
H	0.63970100	2.96996500	1.95576300
H	1.15925000	1.47591100	1.25777400
H	-0.37246400	2.05046400	-0.78766000
H	-0.85131800	3.47103400	0.07089500
H	-1.73462500	2.07184400	1.96581300
H	-1.31282100	0.61841500	1.11184000
H	-2.81806700	1.47244800	-0.82982800
H	-3.33441000	2.73452600	0.25170900

conf_38

C	-2.96222500	1.70515000	0.23617900
C	-4.03004600	0.78734600	0.84691300
C	-3.79359600	-0.72402400	0.70037400
C	-3.85937300	-1.23843400	-0.74456400
C	-2.61152300	-0.99897000	-1.56036900
O	-1.49503000	-0.92816900	-1.08344200
O	-2.73860200	-0.90489000	-2.88712100
H	-3.66331300	-0.97009600	-3.16175700
H	-4.72992000	-0.82169900	-1.26232900
H	-3.99510700	-2.32758500	-0.74451100
H	-4.56432200	-1.24785900	1.27039500
H	-2.83742500	-1.02032400	1.13875100
H	-5.00208200	1.03073500	0.40435900
H	-4.12234400	1.01606100	1.91371900
C	-1.61307600	1.66005600	0.95979300
C	-0.51195000	2.44237400	0.23648500
C	0.82314300	2.48706000	0.99287200
C	1.95394300	3.22530200	0.24349900
C	3.06125600	2.31133100	-0.30821600
C	2.62498800	1.36716700	-1.39618400
C	3.00792400	0.08945300	-1.56095900
C	3.94339500	-0.69769900	-0.68176200
C	3.59087500	-2.19053700	-0.55558900
C	2.18465500	-2.48837100	-0.01885600
C	1.85872800	-1.83443800	1.32975800
C	0.45817200	-2.16726100	1.86149600
C	0.11563500	-1.42718300	3.15546200
H	0.14319100	-0.34130400	3.01785100
H	-0.88218300	-1.69078800	3.51282700
H	0.82721000	-1.67390400	3.94723200
H	0.39333000	-3.24747400	2.02332800
H	-0.30707900	-1.95538800	1.10026600
H	2.60403900	-2.15083200	2.06655300
H	1.98893400	-0.74149200	1.27912100
H	2.06754500	-3.57126100	0.08313100
H	1.43845900	-2.21538800	-0.78215500
H	3.70072300	-2.67072100	-1.53308900
H	4.32912600	-2.66134000	0.10025400
H	4.01808200	-0.24135300	0.30780300

H	4.94935900	-0.62541800	-1.11292800
H	2.67809600	-0.42120600	-2.46665800
H	2.00938100	1.81316700	-2.17827300
H	3.52751700	1.76507800	0.51623100
NA	0.42459900	-0.16112600	-0.42965900
H	3.85060700	2.94808300	-0.72699500
H	1.52638900	3.81139700	-0.57718600
H	2.42342000	3.94822200	0.91369300
H	0.64223300	2.96353800	1.95984900
H	1.16049000	1.47167600	1.25629600
H	-0.37331900	2.05259400	-0.78519200
H	-0.85095500	3.47087400	0.07792400
H	-1.73233800	2.06634100	1.96987700
H	-1.31169900	0.61536100	1.11112300
H	-2.81915100	1.47501900	-0.82618500
H	-3.33398800	2.73420100	0.25942200

conf_16

C	3.30778700	1.26277500	-0.44162800
C	4.45553800	0.24220800	-0.41402800
C	4.06655500	-1.22092900	-0.15850300
C	3.65430400	-1.51483900	1.29495800
C	2.27612500	-1.02187900	1.64713800
O	1.30475900	-1.18517700	0.93157000
O	2.09408200	-0.39665300	2.81864500
H	2.92339000	-0.31081300	3.30798400
H	4.39905500	-1.11542200	1.99016800
H	3.62190700	-2.59916700	1.45297300
H	4.92589100	-1.85688900	-0.38271300
H	3.26253900	-1.54021200	-0.82587600
H	5.18953600	0.54472900	0.34172500
H	4.98023400	0.29234700	-1.37346400
C	2.39885600	1.14100300	-1.67105500
C	1.30003400	2.21198000	-1.73159300
C	0.08343700	1.91089600	-0.85026000
C	-0.89589300	3.08211200	-0.72707300
C	-2.25848000	2.70310900	-0.12319700
C	-2.15213300	2.03636800	1.22311400
C	-2.95247900	1.08458900	1.72739400
C	-4.16701800	0.45798600	1.09272400
C	-3.95564400	-1.03037700	0.74663400
C	-3.21250700	-1.24424800	-0.58169400
C	-2.51046900	-2.60464100	-0.68082800
C	-1.76672900	-2.85505200	-1.99828200
C	-0.56323100	-1.93435700	-2.21995800
H	-0.85952400	-0.88487200	-2.31446400
H	0.16761400	-2.02781000	-1.40691000
H	-0.03611400	-2.18837800	-3.14161300
H	-2.46809400	-2.74796300	-2.83204600
H	-1.42730700	-3.89429300	-2.01595500
H	-1.80815200	-2.73549800	0.16081200
H	-3.26059200	-3.38696500	-0.53152800
H	-2.51007200	-0.41553100	-0.75818000

H	-3.92084300	-1.14771200	-1.41025000
H	-3.41024700	-1.51337700	1.56917300
H	-4.91928900	-1.54280600	0.69797200
H	-4.47674700	1.00532600	0.19935500
H	-4.99135000	0.54356900	1.80745600
H	-2.74837200	0.74773000	2.74412400
H	-1.36239000	2.41810100	1.87054900
H	-2.80728400	2.06686600	-0.82403900
NA	-0.73164200	-0.48396900	0.57352700
H	-2.85616100	3.61619300	-0.01537100
H	-0.43187700	3.86838100	-0.12209800
H	-1.06289400	3.51625500	-1.71714600
H	-0.44121800	1.04938800	-1.29124800
H	0.43184300	1.62552300	0.15228100
H	1.72204400	3.18121200	-1.44264000
H	0.95366700	2.32794600	-2.76304200
H	3.02582200	1.22296800	-2.56458500
H	1.94140500	0.14552600	-1.71348900
H	2.71509700	1.20811600	0.47937900
H	3.74749600	2.26561400	-0.44098100

conf_41

C	-3.48447200	1.44944000	-0.00011100
C	-3.69926000	0.07053900	0.63382600
C	-4.36512400	-0.95263300	-0.29396300
C	-3.55550300	-1.27877000	-1.57525700
C	-2.16120500	-1.71412000	-1.22485300
O	-1.18237100	-0.99552700	-1.31550100
O	-1.99205800	-2.94357100	-0.71792000
H	-2.82363900	-3.43660300	-0.69217100
H	-3.47416200	-0.40147200	-2.21455200
H	-4.06176200	-2.06518200	-2.14304200
H	-5.34158700	-0.58218500	-0.61722400
H	-4.55774300	-1.87628900	0.26190200
H	-4.33242700	0.18320700	1.51973200
H	-2.74728200	-0.33134600	1.00069500
C	-2.93788600	2.49476400	0.98288000
C	-1.48669000	2.28081200	1.43653600
C	-0.44582100	2.51158300	0.33507600
C	0.99335700	2.42908300	0.85638800
C	2.09146500	2.77388600	-0.17070400
C	2.07227000	1.92122500	-1.41066100
C	2.98114700	1.00902200	-1.79192600
C	4.24032000	0.58247200	-1.08693300
C	4.15023000	-0.87378200	-0.58210700
C	3.32670200	-1.01704000	0.70856100
C	2.65799300	-2.38857600	0.86835600
C	1.78648600	-2.53902400	2.12216800
C	0.49358300	-1.71668400	2.08833500
H	0.68771900	-0.63806400	2.05038900
H	-0.13917800	-2.01154200	1.24127200
H	-0.10408500	-1.87844900	2.98733700
H	2.37221700	-2.26283000	3.00462600

H	1.52525800	-3.59337600	2.24523600
H	2.05370300	-2.62369000	-0.02427400
H	3.44092900	-3.15243600	0.87622000
H	2.58807200	-0.20491100	0.76582200
H	3.96927000	-0.83116700	1.57452300
H	3.71766400	-1.49646300	-1.37569700
H	5.15477800	-1.26816700	-0.41318900
H	4.48053200	1.24716300	-0.25404900
H	5.06719900	0.66043000	-1.79952100
H	2.81495300	0.51385000	-2.74937400
H	1.24338400	2.10592800	-2.09301800
H	3.06390900	2.71484900	0.32119800
NA	0.75707600	-0.41845700	-0.52253700
H	1.95920200	3.81841800	-0.47423100
H	1.09583200	3.11011300	1.70647500
H	1.18689100	1.43082800	1.27721900
H	-0.61169900	1.80276700	-0.48740800
H	-0.60408300	3.50190500	-0.10741100
H	-1.27560000	2.97202900	2.25890700
H	-1.36408300	1.27504800	1.85790700
H	-3.01521500	3.48692100	0.52569800
H	-3.58519400	2.51793900	1.86591900
H	-2.81744800	1.36956500	-0.86441100
H	-4.44427700	1.80583800	-0.38989200

conf_22

C	-3.40344000	0.83472400	-0.80126400
C	-4.46886400	-0.11650600	-0.23812500
C	-3.95270200	-1.27413200	0.63014300
C	-3.06061700	-2.27658900	-0.11936800
C	-1.66117000	-1.77890600	-0.37555500
O	-1.02782500	-1.10866300	0.41770900
O	-1.06545500	-2.12017700	-1.52790100
H	-1.65407800	-2.64329100	-2.08872400
H	-3.53295600	-2.58683700	-1.05705100
H	-2.93833100	-3.18364100	0.48533200
H	-4.81151800	-1.82628300	1.01837500
H	-3.40807800	-0.90350600	1.50148600
H	-5.04654300	-0.53384200	-1.07035500
H	-5.18028100	0.46341600	0.35865100
C	-2.65728200	1.64380900	0.26307200
C	-1.48953500	2.45723300	-0.30209400
C	-0.74787000	3.27042100	0.76521300
C	0.35294300	4.18980100	0.22443800
C	1.56682600	3.46888500	-0.38150000
C	2.34555400	2.65812600	0.62402900
C	3.12887900	1.59704200	0.37306400
C	3.39505900	0.96366600	-0.96930100
C	3.72813400	-0.53319700	-0.88694600
C	2.60302000	-1.38489900	-0.29596900
C	2.91275700	-2.88455500	-0.27289400
C	1.76958000	-3.75966400	0.25623000
C	1.38446700	-3.48386200	1.71264700

H	0.94127400	-2.49198100	1.84186900
H	0.64753300	-4.20799100	2.06823900
H	2.25546200	-3.55141100	2.37119100
H	2.06819000	-4.80781500	0.16152700
H	0.89362600	-3.63158300	-0.38934800
H	3.16620100	-3.20213900	-1.28961500
H	3.81055500	-3.05071300	0.33372600
H	1.68070900	-1.23098800	-0.87687500
H	2.42114500	-1.06133000	0.74017100
H	3.96596200	-0.89889900	-1.88980800
H	4.63442800	-0.66934500	-0.28682900
H	4.24100000	1.48796200	-1.43052700
H	2.55166000	1.12363000	-1.65224300
H	3.70184500	1.18761700	1.20448900
H	2.33320500	3.04258000	1.64334900
H	2.24299200	4.22630000	-0.79667200
NA	0.55422700	0.37832500	0.69687100
H	1.26936300	2.85635400	-1.23945500
H	-0.08034600	4.85165500	-0.53135100
H	0.70217700	4.83866900	1.03391100
H	-1.48189600	3.87916900	1.30130800
H	-0.33011100	2.60593300	1.54081300
H	-0.80345100	1.78714000	-0.84617500
H	-1.85956300	3.14336100	-1.07154100
H	-3.36034800	2.32519300	0.75413000
H	-2.29139500	0.97539100	1.04952500
H	-2.68263500	0.28031800	-1.41572000
H	-3.89049800	1.52766200	-1.49477400

conf_72

C	3.53344700	1.05195600	0.12829900
C	4.06526800	-0.24910000	0.74558400
C	4.47762200	-1.31373800	-0.28580700
C	3.35426300	-2.30910500	-0.66285200
C	2.11932900	-1.65115300	-1.21463300
O	1.06031900	-1.58902400	-0.61737700
O	2.19486900	-1.08403700	-2.42609800
H	3.07836000	-1.17960500	-2.80788200
H	3.04813000	-2.87121700	0.21915700
H	3.72989200	-3.02451000	-1.40246700
H	5.29617000	-1.91964600	0.10838800
H	4.87429000	-0.82668000	-1.18439600
H	3.32737100	-0.68031000	1.43070900
H	4.93459200	-0.00007600	1.36091700
C	3.08321400	2.09019800	1.16602000
C	1.80290400	1.72854300	1.93537500
C	0.55435700	1.58145300	1.05361600
C	0.13599400	2.86054500	0.32140300
C	-1.23436300	2.76502900	-0.36584300
C	-1.30473300	1.72371800	-1.45404900
C	-2.38347900	1.01689600	-1.82846100
C	-3.77043100	1.11065900	-1.25352000
C	-4.49248400	-0.24033600	-1.11018900

C	-3.81957500	-1.25239500	-0.17268600
C	-3.57084200	-0.72754500	1.24927500
C	-3.11239300	-1.79039600	2.25790500
C	-1.69882900	-2.33072200	2.02280800
H	-1.59040300	-2.81142300	1.04297600
H	-0.94331000	-1.54015200	2.13780900
H	-1.43727800	-3.09320000	2.75827900
H	-3.82236500	-2.62302700	2.24188900
H	-3.16072900	-1.36636600	3.26452400
H	-2.85461100	0.10957900	1.24435800
H	-4.50436000	-0.28345400	1.60830900
H	-2.89849000	-1.62787700	-0.64607400
H	-4.45563800	-2.14035900	-0.10505800
H	-5.50541300	-0.04853900	-0.74389100
H	-4.60640900	-0.69574000	-2.09916300
H	-4.36641000	1.73780400	-1.92789800
H	-3.75919900	1.63968500	-0.29804100
H	-2.27955100	0.35931100	-2.69221800
H	-0.39334500	1.59778300	-2.03939400
H	-1.46568300	3.73906800	-0.81410700
NA	-0.87670900	-0.71216000	-0.10797600
H	-2.01263700	2.59499100	0.38521300
H	0.11159600	3.68807200	1.03675100
H	0.89288900	3.12164000	-0.42481000
H	0.75977300	0.79810600	0.31419700
H	-0.27939700	1.24427200	1.68755000
H	1.95520000	0.79927600	2.49375100
H	1.61228000	2.50229600	2.68581800
H	3.89179700	2.24199200	1.88858100
H	2.94619900	3.05418600	0.66764800
H	4.33016600	1.49063300	-0.48191100
H	2.71321000	0.84469100	-0.56745400

conf_2

C	-3.44099900	-0.95235900	0.55787000
C	-4.46130000	0.19607300	0.54382000
C	-4.04440600	1.48707700	-0.17548000
C	-2.88318900	2.25481500	0.49527000
C	-1.53740600	1.60500700	0.37115700
O	-0.78103700	1.33645800	1.28025500
O	-1.10830200	1.28064100	-0.88010700
H	-1.76634800	1.48506900	-1.55899600
H	-3.07440900	2.39505400	1.55927300
H	-2.79512800	3.25068600	0.04421500
H	-4.89953800	2.16632200	-0.19408000
H	-3.81932500	1.28425500	-1.23008000
H	-4.72101800	0.44965900	1.57698000
H	-5.38569600	-0.16198900	0.07940700
C	-3.13078500	-1.55128200	-0.81905800
C	-2.18075400	-2.75530400	-0.77673800
C	-0.72490500	-2.42497600	-0.42261900
C	0.16521600	-3.67100100	-0.35773000
C	1.66923600	-3.38473500	-0.22503200

C	2.04129200	-2.62958800	1.02548600
C	3.03823500	-1.74115900	1.15976700
C	3.98797800	-1.30165100	0.06725900
C	3.48313800	-0.09549300	-0.74971800
C	3.27889200	1.18825800	0.06770800
C	2.50318200	2.27788500	-0.68113200
C	2.14337800	3.48447200	0.18936000
C	1.31695400	4.52953900	-0.56077800
H	0.37959400	4.09899700	-0.92948500
H	1.06631300	5.37517400	0.08300100
H	1.86099800	4.91957300	-1.42524100
H	3.06274600	3.94234200	0.56859600
H	1.58762900	3.14217900	1.07094900
H	3.08800700	2.60425000	-1.54762500
H	1.57703000	1.86314800	-1.10971700
H	4.25553000	1.57608200	0.37145400
H	2.77943600	0.97032000	1.02574600
H	4.18338600	0.11043400	-1.56372100
H	2.54897100	-0.38221400	-1.26159400
H	4.18238400	-2.13127900	-0.61594800
H	4.94880200	-1.04256800	0.52060300
H	3.21090200	-1.32669400	2.15048100
H	1.47311800	-2.89204900	1.91773100
H	2.02562500	-2.86059700	-1.11751000
NA	0.80897800	-0.04812500	0.37102400
H	2.19874800	-4.34549100	-0.21248000
H	-0.16125800	-4.29507800	0.48110500
H	0.00522100	-4.26588700	-1.26158700
H	-0.33167000	-1.73252900	-1.18473000
H	-0.70471600	-1.91662000	0.55260200
H	-2.56101700	-3.49037700	-0.05845700
H	-2.18825300	-3.25103600	-1.75241000
H	-4.07316300	-1.86643600	-1.27870200
H	-2.70955200	-0.79369800	-1.49002600
H	-2.52300500	-0.63184800	1.05998800
H	-3.84693400	-1.74804000	1.19134100

conf_120

C	-3.60510700	0.74833600	0.38040900
C	-3.46783500	2.10092300	-0.32418500
C	-2.87085700	3.21801000	0.53969200
C	-1.46146800	2.90256400	1.10152000
C	-0.52255300	2.44473000	0.01834200
O	-0.14494900	1.29296900	-0.10831300
O	-0.11189900	3.34351600	-0.88141200
H	-0.47378100	4.22147400	-0.69778800
H	-1.51086600	2.10836900	1.84414600
H	-1.04990200	3.79180500	1.58935200
H	-3.51879600	3.42151500	1.39641500
H	-2.83757100	4.14579300	-0.04145400
H	-4.45089300	2.42856000	-0.67559400
H	-2.85930400	1.97392200	-1.22926500
C	-4.16174100	-0.33433900	-0.54798400

C	-4.29962500	-1.71827300	0.10080400
C	-3.00185000	-2.31279300	0.66689500
C	-1.86259100	-2.42164000	-0.34979800
C	-0.63558800	-3.16076200	0.20739700
C	0.49582200	-3.27970600	-0.78146900
C	1.81274600	-3.17611500	-0.53691100
C	2.48468600	-2.92197800	0.78549400
C	3.65770400	-1.92952500	0.70615400
C	3.25229600	-0.54272400	0.20278600
C	4.34102000	0.52601600	0.30723800
C	3.89487900	1.88406400	-0.24086300
C	4.97292100	2.96206800	-0.12244800
H	5.25515600	3.12291200	0.92179000
H	5.87561800	2.67691500	-0.66960800
H	4.62759500	3.91658600	-0.52607900
H	2.99094600	2.20704000	0.29081000
H	3.60648300	1.77151900	-1.29409100
H	4.63679600	0.63210200	1.35683400
H	5.23279900	0.18632300	-0.23110400
H	2.37617500	-0.19425800	0.77338000
H	2.97304400	-0.62188500	-0.86135200
H	4.10059700	-1.83372800	1.70144000
H	4.44122900	-2.33393600	0.05682300
H	2.86483500	-3.88128200	1.15684800
H	1.76233000	-2.57986400	1.53319700
H	2.49063400	-3.35117600	-1.37191900
H	0.19499500	-3.54933500	-1.79377100
H	-0.95021400	-4.17277000	0.49064200
NA	0.67546100	-0.52983500	-0.92765200
H	-0.30393400	-2.68851300	1.13782200
H	-2.21572000	-2.93490200	-1.25136400
H	-1.58808200	-1.40645000	-0.67735600
H	-2.65966800	-1.72523700	1.52624500
H	-3.21827900	-3.31300100	1.05643700
H	-5.03656800	-1.66447200	0.90896600
H	-4.71262400	-2.41103100	-0.64031700
H	-5.14477200	-0.02225700	-0.91533600
H	-3.52431800	-0.40324800	-1.43829000
H	-2.62928500	0.43207700	0.76110200
H	-4.25709500	0.85361500	1.25597500

conf_36

C	-3.62526400	-0.22961200	-0.93453500
C	-4.30310400	-1.40358300	-0.21556900
C	-3.43673200	-2.17803000	0.78738900
C	-2.25224300	-2.92063300	0.14543300
C	-1.07531500	-2.03758000	-0.17594100
O	-0.66429100	-1.16881400	0.57141500
O	-0.41217900	-2.24478800	-1.32266600
H	-0.82743400	-2.94164400	-1.84861500
H	-2.57869400	-3.46789600	-0.74449600
H	-1.86263800	-3.66646000	0.84892700
H	-4.06048700	-2.92436400	1.28438300

H	-3.05639400	-1.52103400	1.57304100
H	-4.68380000	-2.10821000	-0.96371800
H	-5.18316900	-1.02765400	0.31607600
C	-3.23392100	0.93460800	-0.02079800
C	-2.64783400	2.12294900	-0.79078300
C	-2.24848800	3.30358000	0.10699400
C	-0.95015100	3.09683200	0.89853500
C	0.31802300	3.24382200	0.03889300
C	1.59379400	3.02471300	0.81489800
C	2.74075500	2.48964100	0.36736000
C	3.04271000	1.98647100	-1.01829500
C	3.82821100	0.66401300	-1.03651800
C	3.07594200	-0.50279000	-0.39369900
C	3.80030000	-1.84786700	-0.49351800
C	2.98588700	-3.03727600	0.03152500
C	2.59512900	-2.93593500	1.50909800
H	2.13282500	-3.86215700	1.85810800
H	3.46866300	-2.74626700	2.13975700
H	1.87035300	-2.13545000	1.69240100
H	3.57251500	-3.94832700	-0.11733400
H	2.08350200	-3.14958500	-0.57991400
H	4.06030800	-2.02812600	-1.54154200
H	4.74845600	-1.78147700	0.05216000
H	2.08921000	-0.60709500	-0.87270200
H	2.93035100	-0.27821900	0.67381500
H	4.06463700	0.41073300	-2.07372600
H	4.78707900	0.80082600	-0.52525100
H	3.63826800	2.75336400	-1.52808400
H	2.12742200	1.88398800	-1.61126400
H	3.57935400	2.45507400	1.06152200
H	1.58378000	3.40810200	1.83430300
H	0.32838800	4.26315900	-0.36584700
NA	0.74997000	0.45112200	0.90743000
H	0.27057300	2.59357900	-0.84212500
H	-0.89642500	3.82140600	1.71633800
H	-0.98596700	2.11335000	1.39557900
H	-2.13885700	4.20784500	-0.50048600
H	-3.06621500	3.50529200	0.80574500
H	-1.78749500	1.78923600	-1.38627400
H	-3.38917300	2.46761900	-1.51827500
H	-4.12205800	1.27127700	0.52675400
H	-2.52512800	0.58643200	0.73689000
H	-2.74228400	-0.57312100	-1.49002400
H	-4.31356400	0.14235200	-1.70066000

conf_12

C	-3.59368300	1.28261000	-0.41240900
C	-4.83859800	0.44146900	-0.72358000
C	-4.74415600	-1.06255400	-0.43179000
C	-3.62434200	-1.79647900	-1.20943700
C	-2.25568700	-1.68131800	-0.59569900
O	-1.28012500	-1.20889400	-1.14959800
O	-2.09984600	-2.14436300	0.65585700

H	-2.92932600	-2.49171300	1.01206500
H	-3.55948500	-1.43020100	-2.23385400
H	-3.85730000	-2.86671400	-1.25833400
H	-5.69235900	-1.52720200	-0.71045000
H	-4.64009800	-1.23354600	0.64607600
H	-5.09033900	0.56816800	-1.78189700
H	-5.68937400	0.83887900	-0.16043400
C	-3.18309400	1.30650100	1.06573500
C	-2.03980800	2.28521200	1.36468200
C	-0.67142900	1.86058400	0.81568000
C	0.38385700	2.96544100	0.93109200
C	1.80371000	2.54359200	0.53067600
C	1.91454500	2.04373600	-0.89316600
C	2.97033200	1.41808600	-1.43553600
C	4.29686500	1.14728400	-0.78096200
C	4.88306800	-0.26280400	-1.02075200
C	4.55586200	-1.28206400	0.07720800
C	3.06100000	-1.48939100	0.31760100
C	2.71433000	-2.54965400	1.36376600
C	1.20717900	-2.66902100	1.59153500
H	0.68092100	-2.95868200	0.67517800
H	0.96773700	-3.42398100	2.34254500
H	0.77942800	-1.72463000	1.95206100
H	3.21298200	-2.29851700	2.30508200
H	3.11951400	-3.51522500	1.04665100
H	2.63912100	-0.52757700	0.64647300
H	2.59752100	-1.76963500	-0.64403800
H	5.01952400	-0.96187600	1.01703600
H	5.01254000	-2.24337600	-0.17852600
H	4.54064000	-0.64034000	-1.99164500
H	5.97005300	-0.18574800	-1.09618000
H	4.25860400	1.36330000	0.29064100
H	4.99110000	1.88394600	-1.20419200
H	2.91982000	1.16325300	-2.49379300
H	1.08374000	2.29108600	-1.55450700
H	2.17329800	1.78402100	1.23263700
NA	0.67547200	-0.38377000	-0.54611800
H	2.47369400	3.40078000	0.66135900
H	0.07156900	3.81231000	0.31040700
H	0.40439800	3.33315500	1.96111500
H	-0.33844800	0.96792200	1.37094500
H	-0.79180400	1.58063300	-0.23911300
H	-2.29449900	3.26718700	0.94996300
H	-1.95044800	2.42535900	2.44629400
H	-4.05413000	1.59286400	1.66460700
H	-2.89597300	0.30622300	1.40989900
H	-2.76054000	0.94902900	-1.03850800
H	-3.80220200	2.31099000	-0.72620200

conf_100

C	-3.13028800	-0.89040500	-1.44184400
C	-3.83834200	-2.20959500	-1.10058600
C	-3.85186500	-2.62307200	0.37788100

C	-2.46250700	-2.95750100	0.96695400
C	-1.57433000	-1.77655900	1.22489200
O	-0.44854800	-1.61781300	0.80208900
O	-2.05954800	-0.78067000	2.01723900
H	-2.96885000	-0.94567100	2.30349400
H	-1.91501000	-3.63163100	0.30805800
H	-2.58898100	-3.47029400	1.92820100
H	-4.46103100	-3.52374000	0.48092300
H	-4.35918700	-1.86317000	0.98486700
H	-3.37710200	-3.01825100	-1.67727000
H	-4.87722500	-2.15023400	-1.44071200
C	-3.78759900	0.35821500	-0.84290500
C	-3.13216400	1.67671100	-1.27133300
C	-1.71402600	1.89954300	-0.73107900
C	-1.15104100	3.27603900	-1.09916400
C	0.32478300	3.48726200	-0.72625700
C	0.60108200	3.37555500	0.75019700
C	1.70136300	2.86139400	1.32255100
C	2.89168100	2.27315200	0.60434600
C	2.81500100	0.74385600	0.43795000
C	4.08107400	0.11513600	-0.14701400
C	3.96387600	-1.39841000	-0.33607900
C	5.22678100	-2.03475200	-0.92234900
C	5.09859300	-3.54722500	-1.10867200
H	6.01235800	-3.97430600	-1.52771300
H	4.90520300	-4.04752700	-0.15497100
H	4.27562800	-3.79321800	-1.78665200
H	6.07725600	-1.81536900	-0.26695100
H	5.45326300	-1.56298900	-1.88535300
H	3.73716400	-1.86947100	0.62911400
H	3.11094500	-1.61895900	-0.99110600
H	4.92661800	0.34172100	0.51171700
H	4.30309100	0.59149400	-1.10871700
H	2.61851900	0.27374700	1.41473800
H	1.97883400	0.49475900	-0.23544300
H	3.79471300	2.51593200	1.17134000
H	3.01191200	2.73000500	-0.38043600
H	1.77272800	2.90214700	2.40901400
H	-0.15409900	3.81331600	1.40303100
H	0.62000000	4.49198800	-1.05288600
NA	0.12415700	0.56020900	1.21764900
H	0.95198700	2.79139600	-1.29189000
H	-1.25986800	3.42487600	-2.17727800
H	-1.76172300	4.04924700	-0.62037500
H	-1.75689900	1.80488300	0.36578700
H	-1.04454000	1.11746300	-1.11815100
H	-3.10586800	1.73117100	-2.36528100
H	-3.75979200	2.51010000	-0.93950200
H	-4.83978500	0.37623300	-1.14544600
H	-3.79154700	0.30873200	0.25222200
H	-2.07506000	-0.94861600	-1.15802600
H	-3.12705300	-0.78625600	-2.53204900

conf_184

C	-3.94057000	0.15034500	0.05004000
C	-4.18201300	-1.21026300	-0.61934600
C	-4.08923000	-2.41247200	0.33500800
C	-2.67252200	-3.02511200	0.45337900
C	-1.62918100	-2.05516500	0.93367200
O	-0.73733100	-1.61216200	0.23235900
O	-1.69419000	-1.63867600	2.20598900
H	-2.44133700	-2.03769500	2.67299800
H	-2.34560700	-3.38980200	-0.52011200
H	-2.69743000	-3.87817600	1.14014000
H	-4.73667800	-3.21873400	-0.01611400
H	-4.47046100	-2.13393800	1.32461900
H	-3.48328400	-1.36010600	-1.44928900
H	-5.17938700	-1.19353000	-1.06789800
C	-3.92454000	1.32591900	-0.93837400
C	-2.68309200	1.38764000	-1.84223400
C	-1.37205900	1.67837700	-1.09689600
C	-1.27627700	3.08255800	-0.49199500
C	0.12341800	3.43458100	0.03476500
C	0.59226800	2.56327100	1.17131500
C	1.84940300	2.16413700	1.42295300
C	3.08280000	2.48149200	0.61757800
C	4.10603500	1.33541600	0.56054200
C	3.54415200	0.03687300	-0.02370200
C	4.58518100	-1.03898400	-0.33458100
C	3.96326400	-2.32887900	-0.87580900
C	4.99890000	-3.40784000	-1.19556100
H	5.56886100	-3.68082700	-0.30328400
H	4.52425800	-4.31383100	-1.57855200
H	5.70899700	-3.05858800	-1.95001800
H	3.38729400	-2.09835200	-1.78209700
H	3.24606100	-2.71920200	-0.14147100
H	5.30318900	-0.64299700	-1.06077200
H	5.15510000	-1.25866000	0.57495000
H	3.00575400	0.27210700	-0.95938100
H	2.83441100	-0.39453800	0.70070300
H	4.95937400	1.65858100	-0.04207000
H	4.49493800	1.13488200	1.56432800
H	3.56728400	3.35743100	1.06562900
H	2.81341300	2.78245400	-0.39991300
H	2.02473500	1.61038700	2.34549000
H	-0.17230300	2.30503800	1.90421100
H	0.10730400	4.47133500	0.39282900
NA	0.75796100	-0.06149500	-0.13925700
H	0.84228700	3.42019300	-0.79077600
H	-1.55829100	3.81673300	-1.25241800
H	-2.00291700	3.18494800	0.31994700
H	-1.27069300	0.93830300	-0.29319700
H	-0.53699300	1.54138700	-1.80206400
H	-2.57995800	0.44684500	-2.39187900
H	-2.83111600	2.16342400	-2.59993900
H	-4.81544200	1.26624500	-1.57195300
H	-4.01769200	2.26356700	-0.38278900
H	-4.73556400	0.31642200	0.78463300

H	-3.00912800	0.14046700	0.62603800

conf_139

C	-3.83792700	-0.36610900	-0.69806700
C	-4.18772500	-1.72346500	-0.07753700
C	-3.02112900	-2.48486500	0.56724000
C	-1.87907000	-2.81211700	-0.41227200
C	-0.88480300	-1.69484200	-0.59816500
O	-0.44523900	-1.02757900	0.32078900
O	-0.40499200	-1.45747600	-1.82737500
H	-0.82683600	-2.02486000	-2.48674000
H	-2.27921400	-3.13982800	-1.37665800
H	-1.28701100	-3.64694900	-0.01778200
H	-3.39809400	-3.42860700	0.96721800
H	-2.60811100	-1.93319900	1.41493500
H	-4.63573600	-2.36156700	-0.84760900
H	-4.96250000	-1.58043700	0.68256300
C	-3.35771400	0.69215800	0.29768800
C	-3.04220100	2.02965500	-0.37811700
C	-2.44698400	3.07802300	0.56989700
C	-1.01057100	2.79868200	1.03248000
C	0.02922900	2.85888800	-0.10089400
C	1.44829700	2.90035300	0.40101800
C	2.52487300	2.30733900	-0.13775000
C	2.58397400	1.44418000	-1.37731300
C	3.85336400	0.57716700	-1.43001600
C	3.98996200	-0.42949300	-0.28001500
C	3.06024600	-1.64585500	-0.38818300
C	3.12754000	-2.58879700	0.82254600
C	2.48771100	-2.03305000	2.10043100
H	2.64746000	-2.69907700	2.95010500
H	2.92247800	-1.06801200	2.39209600
H	1.39964700	-1.94164700	1.98888200
H	4.17539100	-2.83308800	1.02271300
H	2.63814100	-3.53440500	0.57187700
H	2.02054000	-1.33965900	-0.56470700
H	3.33657900	-2.20173900	-1.28930800
H	3.84665100	0.09333700	0.67643700
H	5.02060700	-0.79418000	-0.24370800
H	3.88550100	0.04453500	-2.38505900
H	4.72073100	1.24442200	-1.42287000
H	2.57430800	2.10218100	-2.25419000
H	1.68357800	0.82629900	-1.48785400
H	3.48757200	2.51898600	0.32381600
H	1.61319100	3.54763700	1.26090700
H	-0.14565100	3.78007300	-0.67222900
NA	1.13316000	0.28973000	1.10702700
H	-0.11684700	2.04390900	-0.81693800
H	-0.73757000	3.53628400	1.79386900
H	-0.97288300	1.82168000	1.53807600
H	-2.45995000	4.05813100	0.08113400
H	-3.09066000	3.16936100	1.45100100
H	-2.36432100	1.86114000	-1.22372200

H	-3.96244600	2.43158600	-0.81371200
H	-4.13008800	0.84674700	1.06031600
H	-2.47485100	0.32569500	0.82783400
H	-3.08121500	-0.49140900	-1.48496700
H	-4.72374500	0.01297000	-1.21816100

conf_26

C	-3.87846800	-0.01573700	-0.39344200
C	-4.31532600	-1.48666100	-0.40098400
C	-3.25800700	-2.51309100	0.02561200
C	-2.08946500	-2.65134200	-0.96912500
C	-1.02496100	-1.59840500	-0.82054800
O	-0.53686000	-1.28107800	0.24871100
O	-0.54111100	-1.00694100	-1.92650000
H	-0.99759200	-1.31580100	-2.72075000
H	-2.46446900	-2.68466300	-1.99660300
H	-1.57087100	-3.60036900	-0.78960000
H	-3.73407200	-3.49290200	0.10474000
H	-2.85527100	-2.28311600	1.01487900
H	-4.67195900	-1.75172800	-1.40313300
H	-5.17976100	-1.59476500	0.26186300
C	-3.59616500	0.54725100	1.00462500
C	-3.47895500	2.07862800	1.04211000
C	-2.32742900	2.67531800	0.22240200
C	-0.93946100	2.30326900	0.74956900
C	0.20501500	2.88759300	-0.09473300
C	1.56495300	2.69685800	0.52301000
C	2.68182700	2.23593700	-0.06438500
C	2.85420300	1.79661500	-1.49355100
C	3.77488300	0.57965700	-1.70165900
C	3.25864300	-0.73982500	-1.10128800
C	3.66851900	-0.99103800	0.35813200
C	3.07108800	-2.26864900	0.96333800
C	3.41311800	-2.45276400	2.44309800
H	3.03437900	-1.62490100	3.05403600
H	2.98718200	-3.37638700	2.83946500
H	4.49482200	-2.49295300	2.59179600
H	3.43297300	-3.12734100	0.38979200
H	1.97825600	-2.29373300	0.82850100
H	4.75971600	-1.05421900	0.40902400
H	3.43673300	-0.12045700	0.99195100
H	3.63261500	-1.57907200	-1.69481200
H	2.16512500	-0.78567300	-1.22130300
H	3.90592100	0.45159800	-2.77881100
H	4.77061400	0.79867000	-1.30070600
H	3.28557700	2.64163300	-2.04485300
H	1.88426200	1.60098600	-1.96074400
H	3.59442300	2.24187400	0.52985700
H	1.65111600	3.04576900	1.55233600
H	0.03528100	3.96650800	-0.19866800
NA	1.13801000	-0.01716100	0.94514600
H	0.16565500	2.47870700	-1.10773000
H	-0.84354600	2.64715900	1.78657800

H	-0.87067500	1.20777900	0.77437900
H	-2.41064000	2.36917000	-0.82632200
H	-2.41865300	3.76632800	0.22564000
H	-4.42045000	2.50743700	0.68318500
H	-3.37706300	2.40266300	2.08392300
H	-4.41660600	0.25359700	1.66835400
H	-2.69600500	0.08495800	1.42309900
H	-3.00693800	0.12903800	-1.04442100
H	-4.67849800	0.57544700	-0.85232400

conf_77

C	3.17582400	-0.25945500	1.16897000
C	4.40037600	0.23776500	0.38659600
C	4.11302100	1.07569300	-0.86768300
C	3.54911300	0.26714700	-2.04911600
C	2.09423400	-0.09671700	-1.91607600
O	1.23255200	0.67784300	-1.54400900
O	1.71257500	-1.33747300	-2.26644300
H	2.46662700	-1.87254900	-2.54873100
H	4.15571600	-0.62752700	-2.22083700
H	3.60919800	0.86967200	-2.96334800
H	5.04744500	1.52785200	-1.20772200
H	3.43303400	1.90143500	-0.64639900
H	5.02059900	-0.61962700	0.10051400
H	5.01740200	0.83949000	1.06142900
C	2.37797100	0.86726000	1.83332400
C	1.23961800	0.40401700	2.75239900
C	0.10615000	-0.35189900	2.03166900
C	0.17178300	-1.87851200	2.11198300
C	-0.99668000	-2.57149300	1.39208400
C	-0.99030700	-2.36438400	-0.09732100
C	-2.03748600	-2.12464600	-0.90049900
C	-3.48700500	-1.96320400	-0.52653900
C	-4.02546500	-0.56646400	-0.89354000
C	-3.55313000	0.53850300	0.06582200
C	-3.53003400	1.93504900	-0.57104800
C	-2.97806500	3.04979900	0.32637200
C	-1.47175200	2.95068500	0.58782600
H	-1.20120500	2.04026900	1.13245300
H	-0.90015800	3.00264400	-0.35009400
H	-1.11686900	3.78328700	1.19760200
H	-3.51454300	3.04330400	1.28044300
H	-3.19167100	4.01571400	-0.13856300
H	-2.95717500	1.91480900	-1.51587700
H	-4.54775400	2.18625100	-0.88311500
H	-2.57243500	0.27125600	0.48593000
H	-4.20424500	0.56165800	0.94473000
H	-3.71631100	-0.33004200	-1.92116900
H	-5.11764200	-0.58154000	-0.91509400
H	-3.64841300	-2.15618300	0.53636700
H	-4.06402000	-2.71879000	-1.06966400
H	-1.84085500	-2.08198700	-1.97395600
H	-0.01797700	-2.48414200	-0.57455200

H	-1.94440300	-2.24572200	1.82925400
NA	-0.89758700	0.54803100	-0.97822600
H	-0.92934500	-3.65013900	1.57986500
H	1.12033000	-2.23743600	1.69991200
H	0.16340900	-2.17580400	3.16453000
H	-0.86536000	-0.03195500	2.42400500
H	0.13166800	-0.05629500	0.97512200
H	1.64395400	-0.22508700	3.55285600
H	0.82496600	1.28729000	3.24602100
H	3.07105000	1.48051300	2.41948000
H	1.96118300	1.52962700	1.06570100
H	2.52300600	-0.85652500	0.52108700
H	3.52423400	-0.94988300	1.94459300

conf_47

C	2.11074300	-0.73044500	-1.45506200
C	3.52479600	-0.14409000	-1.34982200
C	4.15145800	-0.09158500	0.05051300
C	3.36969300	0.76776700	1.07639700
C	2.21916300	0.05291900	1.72963300
O	1.05031800	0.37619500	1.62043200
O	2.50056800	-1.01990700	2.48369400
H	3.45085900	-1.19825000	2.51307200
H	2.97681700	1.66874000	0.60592500
H	4.04827200	1.08228500	1.87748200
H	5.15049000	0.34040300	-0.03853900
H	4.30243200	-1.10582900	0.43698500
H	3.51352700	0.87211100	-1.75768100
H	4.19767300	-0.72087100	-1.99282100
C	1.98420500	-2.18799100	-0.99243700
C	0.64701200	-2.84064200	-1.36598100
C	-0.58179500	-2.21144900	-0.69989900
C	-1.90018400	-2.88461600	-1.10129500
C	-3.15781400	-2.03918800	-0.83134000
C	-3.40706600	-1.72233100	0.61725800
C	-3.70294100	-0.51801000	1.13400400
C	-3.79435900	0.78370900	0.37081800
C	-2.44575700	1.52287300	0.29987100
C	-2.47647500	2.84774100	-0.46454100
C	-1.12699600	3.57806200	-0.46422600
C	0.01929300	2.79638900	-1.11658100
C	1.32020400	3.59813100	-1.18129400
H	2.12117000	3.02241700	-1.65292700
H	1.19130400	4.51643900	-1.76007100
H	1.65413400	3.88702900	-0.17963600
H	-0.27830500	2.49547100	-2.12838000
H	0.20632800	1.87284400	-0.55907500
H	-1.24755700	4.53295800	-0.98575400
H	-0.85073400	3.82998400	0.56768000
H	-3.24162000	3.49492600	-0.02450800
H	-2.79085600	2.65285600	-1.49634700
H	-2.08437300	1.73094900	1.32058000
H	-1.71819900	0.86674200	-0.19626200

H	-4.53097800	1.43066700	0.85413500
H	-4.15348900	0.60887000	-0.64579400
H	-3.92741500	-0.46532400	2.19934500
H	-3.40545000	-2.57945500	1.29095000
H	-4.02677200	-2.59530000	-1.20353900
NA	-0.98039500	-0.41265000	1.60758800
H	-3.10546800	-1.12133300	-1.42252400
H	-1.86864900	-3.10040600	-2.17311300
H	-1.98483900	-3.85343200	-0.59802500
H	-0.44404500	-2.29635200	0.39051000
H	-0.62705900	-1.14544700	-0.95936900
H	0.51565500	-2.79030100	-2.45266500
H	0.67876600	-3.90470300	-1.11037600
H	2.79150600	-2.77302700	-1.44604800
H	2.12918600	-2.26611400	0.09153500
H	1.40932800	-0.09194600	-0.91135200
H	1.80652600	-0.67221700	-2.50595800

conf_25

C	-3.24854500	-0.01439700	1.02701100
C	-4.00500500	0.05912700	-0.30525300
C	-4.10906000	-1.27507500	-1.06003800
C	-2.86288300	-1.64017000	-1.90485400
C	-1.61214000	-1.81147200	-1.08901900
O	-0.67861500	-1.02882300	-1.09810200
O	-1.53391000	-2.87435200	-0.27613400
H	-2.32584800	-3.42653900	-0.33362300
H	-2.66699100	-0.85594200	-2.63593400
H	-3.05388600	-2.56917700	-2.45263300
H	-4.94653700	-1.23466100	-1.76000700
H	-4.34492400	-2.08336600	-0.35762900
H	-3.54654200	0.80264300	-0.96615700
H	-5.01764900	0.42183000	-0.10363700
C	-3.15760300	1.32455100	1.77309800
C	-2.47175800	2.46349600	1.00566200
C	-1.06070400	2.14088500	0.50146000
C	-0.35066200	3.35254600	-0.10942300
C	1.02444000	3.04065400	-0.71805000
C	2.03962900	2.56248000	0.29242800
C	3.10848200	1.78289200	0.06673700
C	3.55177300	1.20376600	-1.25209900
C	4.23248600	-0.17523000	-1.17972800
C	3.31963600	-1.34320500	-0.77244600
C	3.10755000	-1.51732000	0.74023200
C	2.11238900	-2.62741800	1.10330100
C	1.84608100	-2.72733700	2.60614400
H	1.42324200	-1.79768300	3.00435200
H	1.14442600	-3.53245000	2.83273100
H	2.76935300	-2.92416800	3.15664100
H	2.50109500	-3.58014700	0.73091600
H	1.16113800	-2.49233000	0.56807900
H	4.07386900	-1.73899200	1.20348700
H	2.81109100	-0.56988000	1.21670400

H	3.74166900	-2.27762000	-1.15311900
H	2.35690800	-1.25217100	-1.30219800
H	4.64887200	-0.38660500	-2.16763900
H	5.08657400	-0.12542100	-0.49538400
H	4.27003000	1.90871600	-1.68974700
H	2.72012600	1.16631400	-1.96586800
H	3.78517300	1.60836900	0.90102900
H	1.92110400	2.96963200	1.29560200
H	1.41312900	3.95382400	-1.18485000
NA	0.83539900	-0.05996200	0.16875900
H	0.91514500	2.32588400	-1.54381600
H	-0.99030700	3.78119500	-0.88656400
H	-0.23802100	4.12705200	0.65673100
H	-0.47023500	1.75635100	1.34992900
H	-1.12995800	1.34977000	-0.25733600
H	-3.08533400	2.76644700	0.15101400
H	-2.41975300	3.33936500	1.65999400
H	-4.16608300	1.64950000	2.04867600
H	-2.62587100	1.15941800	2.71716600
H	-3.74991900	-0.74079900	1.67567800
H	-2.23892800	-0.40600700	0.87397100

conf_68

C	-2.89159000	0.26159700	1.14822900
C	-3.80371100	0.61883900	-0.03167000
C	-4.31275200	-0.58397400	-0.84025600
C	-3.30680400	-1.15230600	-1.87101000
C	-2.04632800	-1.69246200	-1.25284600
O	-0.95989400	-1.15100900	-1.34285100
O	-2.13343600	-2.82005900	-0.53532900
H	-3.03541700	-3.16880900	-0.53084600
H	-3.01293100	-0.37185600	-2.57287100
H	-3.78734700	-1.95294900	-2.44342400
H	-5.19644000	-0.28952900	-1.41088700
H	-4.64672600	-1.37442800	-0.15767600
H	-3.29748000	1.31391000	-0.71069200
H	-4.67190100	1.15935800	0.35855900
C	-2.48498200	1.46269800	2.01259700
C	-1.71501300	2.57749700	1.29176300
C	-0.40195700	2.13710800	0.63238400
C	0.43617100	3.31758800	0.12865800
C	1.85525200	2.94887900	-0.33323700
C	1.90506900	2.06608100	-1.55148300
C	2.73633100	1.03778600	-1.78438200
C	3.78660600	0.48630900	-0.85598000
C	3.80574300	-1.06454700	-0.78423100
C	4.02923000	-1.59967500	0.63791900
C	2.82178900	-1.40557800	1.56876300
C	1.66890800	-2.39137600	1.32096600
C	0.39956000	-2.05579700	2.10961500
H	0.02485800	-1.04459900	1.89910300
H	-0.40776700	-2.75374200	1.88361800
H	0.59152100	-2.08856800	3.18439700

H	2.00665700	-3.39480000	1.59351100
H	1.43721300	-2.47985200	0.24889300
H	3.13791400	-1.50689000	2.61049300
H	2.46290700	-0.36445400	1.50012300
H	4.90444200	-1.10192800	1.06591300
H	4.27432100	-2.66525500	0.59483400
H	2.87500300	-1.48428900	-1.19420600
H	4.58228500	-1.45036600	-1.44778000
H	3.66458400	0.90703600	0.14534700
H	4.76924600	0.83253900	-1.19316300
H	2.68057200	0.55777000	-2.76065100
H	1.22223900	2.34749900	-2.35228100
H	2.40929300	2.50542400	0.50029100
NA	0.82031600	-0.33242000	-0.39389100
H	2.38641300	3.87706200	-0.57709000
H	-0.09784200	3.81581100	-0.68789200
H	0.51982700	4.05393400	0.93297900
H	0.18586500	1.56309900	1.36737200
H	-0.64459100	1.47544000	-0.21119500
H	-2.34900200	3.05014000	0.53450100
H	-1.48815800	3.36099300	2.02144500
H	-3.38484500	1.89782800	2.45943200
H	-1.87879200	1.09881300	2.85024700
H	-3.40977900	-0.46219500	1.78689400
H	-1.99209300	-0.24897100	0.79615000

conf_128

C	-3.23555000	-0.65776600	-0.55846000
C	-4.37732300	-0.01491900	0.23565900
C	-4.37262400	1.51856700	0.27012500
C	-3.21872700	2.14546300	1.08259400
C	-1.84847800	1.96187300	0.49048900
O	-0.90330300	1.47430700	1.08367300
O	-1.65015900	2.36910400	-0.77354500
H	-2.46536600	2.70773700	-1.16953400
H	-3.19078600	1.73147900	2.09059400
H	-3.38413500	3.22544600	1.17694900
H	-5.30227100	1.86994500	0.72359900
H	-4.38354600	1.91233600	-0.75526200
H	-4.36557000	-0.39373700	1.26452600
H	-5.33014500	-0.33978000	-0.19425000
C	-3.36072600	-2.18254200	-0.65099800
C	-2.22095100	-2.87410900	-1.42267400
C	-1.02679000	-3.33984800	-0.57847400
C	-0.19077800	-2.24092300	0.08816000
C	1.06162800	-2.80659100	0.78504900
C	1.82129500	-1.79372600	1.60028400
C	3.05807700	-1.32086700	1.37385600
C	3.98659900	-1.68928300	0.25198600
C	4.62077900	-0.48932000	-0.47787500
C	3.60875800	0.44222300	-1.17402600
C	3.27911900	1.71830900	-0.38721700
C	2.21084000	2.60171100	-1.04677600

C	1.80081900	3.80305000	-0.19370400
H	1.05972600	4.41735300	-0.70818300
H	1.36567000	3.49246500	0.76225300
H	2.66418700	4.43443900	0.02926800
H	1.31458500	2.01699700	-1.31228700
H	2.59530400	2.94431200	-2.01189700
H	3.00448100	1.47570300	0.65299300
H	4.19495300	2.30564200	-0.27220000
H	2.69011300	-0.12451900	-1.39884500
H	3.99015000	0.74329200	-2.15325500
H	5.22590800	0.09049400	0.22731700
H	5.31948200	-0.88760800	-1.21663800
H	3.47886000	-2.32395600	-0.47826900
H	4.79642400	-2.29571000	0.67545100
H	3.47235800	-0.62985200	2.10798300
H	1.31525300	-1.45912600	2.50743900
H	1.70600000	-3.27364800	0.03827600
NA	0.95474300	0.48660500	0.44032300
H	0.74089600	-3.60893000	1.45890100
H	0.11410400	-1.52441800	-0.69390100
H	-0.80177300	-1.69542700	0.81750500
H	-1.38453200	-4.02138500	0.20179000
H	-0.36197100	-3.93247000	-1.21645000
H	-2.62348300	-3.75960100	-1.92193700
H	-1.86953400	-2.21545400	-2.22720800
H	-3.42989600	-2.60691800	0.35821400
H	-4.31344900	-2.41447400	-1.13707400
H	-3.21749900	-0.24251600	-1.57396400
H	-2.27478000	-0.40585600	-0.10448400

9Z_pyr

conf_1

C	2.45510800	3.26891500	0.63769400
C	0.96812400	2.95506400	0.42249200
C	0.37282800	3.62223100	-0.82215600
C	-1.08699000	3.23898300	-1.07602200
C	-1.25925000	1.80938900	-1.51733300
O	-0.34821900	1.07993100	-1.85713700
O	-2.50984900	1.30748700	-1.55169900
H	-3.15719100	1.93537700	-1.20106300
H	-1.52486400	3.86387600	-1.86484900
H	-1.70082100	3.40131000	-0.18171400
H	0.95442500	3.36394100	-1.70907800
H	0.42707700	4.70951500	-0.71639500
H	0.40526800	3.27917000	1.30530400
H	0.83384400	1.87228700	0.35653300
C	3.38052800	2.83121600	-0.51000800
C	3.29717800	1.34680700	-0.88883000
C	3.79635000	0.39385800	0.20803400
C	3.72828400	-1.04628700	-0.21770200
C	3.36164300	-2.09691900	0.52338900
C	2.94853400	-2.10082900	1.97058100
C	1.42916400	-2.25698700	2.17653500

C	0.64589500	-0.98550800	1.84814500
C	-0.87268900	-1.16213400	1.90284500
C	-1.62931900	0.16287600	1.78690000
C	-3.13906700	0.00610000	1.60639000
C	-3.90100400	1.33307200	1.65625700
C	-5.38545300	1.19360700	1.31685300
H	-5.90447100	2.15313900	1.37446900
H	-5.88063700	0.50848100	2.01013700
H	-5.52827700	0.79390100	0.30704700
H	-3.42824600	2.05222600	0.97182400
H	-3.78945300	1.77385200	2.65247800
H	-3.53544200	-0.65946800	2.38202100
H	-3.33952500	-0.49297700	0.64901700
H	-1.22099300	0.73625500	0.94692100
H	-1.42700800	0.76564400	2.67976000
H	-1.19233800	-1.84360400	1.10344900
H	-1.15293500	-1.65549400	2.84038500
H	0.93513900	-0.61468500	0.85745600
H	0.94192100	-0.19963300	2.55318300
H	1.06062600	-3.09233900	1.56757400
H	1.23449800	-2.53503000	3.21725800
H	3.28600300	-1.19083000	2.47325400
H	3.45933500	-2.93202300	2.46693400
H	3.39036500	-3.07881900	0.05107800
H	4.04525300	-1.23703100	-1.24288300
H	4.83949700	0.64925000	0.43807400
H	3.23372100	0.54661900	1.13108200
H	2.26798700	1.08801700	-1.16114700
H	3.89671400	1.18294900	-1.79097600
H	3.16762400	3.43197700	-1.39972000
H	4.41273400	3.07137200	-0.23306800
H	2.57761000	4.34651500	0.79184800
H	2.77561600	2.79655400	1.57151900
H	-3.15778800	-3.80460500	-1.65369200
C	-2.15202900	-3.41448200	-1.58052200
C	-1.92270400	-2.06733900	-1.78243300
N	-0.67131500	-1.58285100	-1.69190100
C	0.38994100	-2.35176000	-1.39680500
C	0.21174500	-3.70679400	-1.18539400
C	-1.07029400	-4.24381000	-1.28181100
H	-1.22869800	-5.30301400	-1.11994900
H	1.06632500	-4.32343400	-0.94460300
H	1.34883600	-1.85641700	-1.30946600
H	-0.53238900	-0.55326600	-1.83055100
H	-2.69472800	-1.34456100	-2.00803300

conf_24

C	-4.79673800	-1.35838000	1.34389600
C	-3.70496500	-2.15641200	2.07608100
C	-2.31350100	-2.20374700	1.43202100
C	-1.69473100	-0.80546600	1.20249600
C	-0.39777000	-0.93734700	0.45865000
O	0.59063900	-1.46550300	0.94955000

O	-0.32592400	-0.51345800	-0.80580300
H	-1.13564400	-0.03479500	-1.10228700
H	-1.48053500	-0.33904600	2.16736300
H	-2.37774000	-0.15560400	0.65783000
H	-2.35912700	-2.74367300	0.48305900
H	-1.63689700	-2.77190600	2.07463300
H	-4.04532600	-3.19064400	2.19427900
H	-3.60785900	-1.75280900	3.09036300
C	-5.14861300	-1.81975600	-0.08284000
C	-4.20780000	-1.35245400	-1.20602200
C	-4.19250300	0.17362100	-1.40788700
C	-2.98541500	0.65466200	-2.16191100
C	-2.27755900	1.76437000	-1.90081600
C	-2.56446000	2.78382300	-0.82799900
C	-1.34590000	3.61916000	-0.41617700
C	-0.23639700	2.80710400	0.25384800
C	0.94947300	3.66520300	0.70288600
C	2.05188900	2.88276300	1.42624800
C	2.74507300	1.83315300	0.55027200
C	3.95159700	1.15931300	1.21582500
C	3.59793200	0.34397100	2.46362400
H	2.78837700	-0.36565700	2.26354000
H	3.26238900	0.98694400	3.27973700
H	4.46262200	-0.21572400	2.83033700
H	4.69065000	1.92374000	1.47897500
H	4.44798800	0.51280600	0.48245000
H	3.07136400	2.31264700	-0.38110300
H	2.01895100	1.06397300	0.26396100
H	1.62564800	2.40243200	2.31413600
H	2.80612200	3.58791600	1.79282700
H	1.37767600	4.17237600	-0.17067500
H	0.58319700	4.45746000	1.36447400
H	0.11240700	2.03458000	-0.43786100
H	-0.64953200	2.28063700	1.12577000
H	-0.94134400	4.12681200	-1.30003000
H	-1.67619000	4.41046900	0.26394500
H	-2.99752700	2.30377300	0.05638400
H	-3.34365000	3.45853100	-1.20411000
H	-1.45689100	2.01081800	-2.57357200
H	-2.70174300	0.06024200	-3.02965600
H	-5.09558200	0.46854600	-1.95687500
H	-4.25817800	0.68980000	-0.44794700
H	-3.19095400	-1.70351500	-1.01788400
H	-4.51009000	-1.83027900	-2.14317700
H	-5.20474500	-2.91392300	-0.09830600
H	-6.15777600	-1.46640200	-0.32063500
H	-5.70132800	-1.43186100	1.95440100
H	-4.54467700	-0.29249600	1.34655000
H	4.14528100	-0.55648100	-3.30399100
C	4.09465600	-1.05657500	-2.34664700
C	2.91344700	-1.04586900	-1.62809800
N	2.84980300	-1.66439100	-0.43790700
C	3.89901700	-2.30389700	0.10382700
C	5.10330400	-2.34779700	-0.57367800
C	5.20131600	-1.71544200	-1.81259800

H	6.13687200	-1.73421100	-2.35804100
H	5.94696800	-2.86268200	-0.13521200
H	3.73866300	-2.75484900	1.07322700
H	1.93991000	-1.60949800	0.10242400
H	2.00381000	-0.56003300	-1.95394600

conf_46

C	-1.56681400	3.89871400	0.22863300
C	-0.24049200	3.13478100	0.11006800
C	0.42826500	3.29229700	-1.26007200
C	1.65480500	2.39758900	-1.44117100
C	1.30821600	0.94386600	-1.63775100
O	0.17774500	0.53565400	-1.83422000
O	2.30881600	0.05324100	-1.61872800
H	3.16089900	0.46183100	-1.39686700
H	2.33767800	2.47959100	-0.58733900
H	2.23558800	2.69907100	-2.32197500
H	0.73660300	4.33281600	-1.39423100
H	-0.28071700	3.06923800	-2.05964200
H	-0.40656500	2.07339000	0.32119700
H	0.44805400	3.49504800	0.88266600
C	-2.70078700	3.37434200	-0.66934100
C	-3.07033700	1.90062000	-0.44801000
C	-3.60410500	1.60602900	0.96577400
C	-3.93095600	0.15507200	1.17867200
C	-3.20889000	-0.72546800	1.87979900
C	-1.90618300	-0.45331200	2.58088000
C	-0.70246900	-0.95288000	1.76681600
C	0.65707200	-0.55146200	2.33887900
C	1.82020200	-1.19563000	1.57532300
C	3.14678000	-0.44846200	1.71691300
C	4.31080500	-1.14379000	1.00093000
C	5.54955700	-0.25814900	0.80969000
C	5.36552800	0.83048400	-0.25242600
H	6.25851100	1.45015800	-0.35696000
H	4.54290000	1.50956400	-0.00120900
H	5.18155700	0.38256500	-1.23872100
H	6.40056200	-0.88341000	0.52484600
H	5.81727500	0.20674100	1.76458800
H	3.97460100	-1.50396400	0.01907200
H	4.58728000	-2.03916800	1.56666600
H	3.39642000	-0.32404000	2.77657000
H	3.01088000	0.56814100	1.32666300
H	1.94209200	-2.23516000	1.90218400
H	1.57079300	-1.24108700	0.50853300
H	0.71652700	-0.81414100	3.40035100
H	0.74527400	0.54106000	2.29145900
H	-0.75695300	-2.04485300	1.66508400
H	-0.77829500	-0.54096100	0.75543400
H	-1.90495500	-0.94266600	3.56061200
H	-1.78430800	0.61613500	2.76883900
H	-3.57230500	-1.74974200	1.94294700
H	-4.84800900	-0.19599800	0.70772900

H	-2.87450600	1.93852700	1.70555300
H	-4.50578200	2.20699700	1.12963900
H	-3.82818700	1.61230800	-1.18579000
H	-2.19419500	1.27217300	-0.64171500
H	-3.58851700	3.99324400	-0.50105500
H	-2.43557000	3.51567000	-1.72180800
H	-1.88924500	3.88158000	1.27365100
H	-1.39317400	4.95387400	-0.00917500
H	0.00902300	-5.13030000	-1.22122400
C	-0.61796800	-4.24956800	-1.23816300
C	-0.06548600	-3.02033200	-1.54059300
N	-0.84556100	-1.92546200	-1.56454800
C	-2.15892700	-1.96119300	-1.28472500
C	-2.76054000	-3.16733900	-0.97594100
C	-1.98305000	-4.32293200	-0.95610000
H	-2.43539200	-5.27725000	-0.71555100
H	-3.81660500	-3.19198300	-0.74648700
H	-2.68221300	-1.01586300	-1.29346500
H	-0.39772300	-0.99607600	-1.75264800
H	0.98178800	-2.86564300	-1.76149900

conf_0

C	-3.70569300	-1.22772000	0.80964400
C	-4.06699500	-1.24130500	-0.68077600
C	-3.52864400	-2.45688900	-1.45139200
C	-2.08419900	-2.29237500	-1.98453100
C	-1.06637500	-2.03639900	-0.91137500
O	-0.52952500	-0.96006300	-0.72867300
O	-0.73334200	-3.05293600	-0.09266600
H	-1.23047400	-3.85508500	-0.30410900
H	-2.03862500	-1.44660100	-2.67039200
H	-1.79188800	-3.19124000	-2.53799500
H	-4.15112000	-2.64670100	-2.32861900
H	-3.60567800	-3.35649700	-0.82900800
H	-3.71732600	-0.32504500	-1.16821200
H	-5.15772800	-1.23086800	-0.76763900
C	-4.26224700	-0.01894600	1.57447300
C	-3.72299600	1.34775500	1.13082900
C	-2.18940800	1.46857800	1.21617900
C	-1.72370600	2.88531300	1.03188300
C	-1.06034700	3.39792500	-0.01027600
C	-0.56936200	2.68525600	-1.24077500
C	0.83653900	3.15212000	-1.65570200
C	1.39100500	2.44562400	-2.89969700
C	1.68941000	0.94984600	-2.72102100
C	2.80884200	0.65634000	-1.71805700
C	3.06986500	-0.83591300	-1.50851200
C	4.18084500	-1.12213400	-0.49568500
C	4.37628700	-2.61301000	-0.21744200
H	5.16752500	-2.78740500	0.51596700
H	4.64811500	-3.15028100	-1.12998900
H	3.45740200	-3.07235900	0.16420000
H	3.96155900	-0.59517800	0.44187200

H	5.11968500	-0.69249600	-0.86071100
H	3.32743500	-1.30064200	-2.46747600
H	2.14139700	-1.32460200	-1.18471100
H	2.57284800	1.11222100	-0.74905600
H	3.73235800	1.14335600	-2.05331400
H	0.78171700	0.41964100	-2.41550600
H	1.97174100	0.53074400	-3.69296300
H	2.30906600	2.95432300	-3.21359200
H	0.67781300	2.57241200	-3.72194400
H	0.80026700	4.22952100	-1.84696200
H	1.52916300	3.02906400	-0.81455600
H	-1.25617200	2.89670900	-2.07145300
H	-0.59458400	1.60153700	-1.10592700
H	-0.85211800	4.46619700	0.01006900
H	-2.01373100	3.56937300	1.82704800
H	-1.87312500	1.10827300	2.20586600
H	-1.73515600	0.81157600	0.47277600
H	-4.03642600	1.57636300	0.10747600
H	-4.17660800	2.11936600	1.76063200
H	-5.35402700	-0.01344000	1.48607000
H	-4.04468600	-0.15312300	2.64015300
H	-4.09807900	-2.14199700	1.26911900
H	-2.62070300	-1.26694700	0.94573600
H	2.20117000	2.35683600	3.02743300
C	2.06892900	1.32027600	2.74938500
C	1.22152100	1.00017600	1.70449200
N	1.05592800	-0.28835600	1.35885500
C	1.67918900	-1.29916000	1.98793000
C	2.53482100	-1.03104700	3.03866600
C	2.73311700	0.29544300	3.42154000
H	3.40266100	0.52854400	4.24042300
H	3.03913800	-1.84513000	3.54043900
H	1.46718100	-2.29219500	1.61671000
H	0.43198700	-0.53063500	0.55566300
H	0.65707800	1.73637700	1.14654200

conf_45

C	-2.22044900	0.45019700	1.83717700
C	-3.42604900	1.37164100	1.62426900
C	-4.67455800	0.69328400	1.04414200
C	-4.59057100	0.34259200	-0.45601600
C	-3.56500900	-0.69321500	-0.82391100
O	-2.72973200	-0.54805000	-1.69534500
O	-3.58122400	-1.86350200	-0.15308500
H	-4.25543100	-1.85860500	0.54077600
H	-4.36570700	1.23214500	-1.04377900
H	-5.56120900	-0.03698500	-0.79762500
H	-5.53186700	1.36093000	1.15699600
H	-4.92392100	-0.19611900	1.63957500
H	-3.14032600	2.20817700	0.97706500
H	-3.69895900	1.81288400	2.58781300
C	-1.02615600	1.16536700	2.47667500
C	0.10787600	0.22787400	2.90957600

C	0.70870200	-0.59691400	1.75600700
C	1.90622100	-1.41268900	2.16857000
C	3.17995400	-1.01361100	2.10024200
C	3.64855200	0.31387700	1.57336800
C	4.23823500	0.21634900	0.15439900
C	4.51961600	1.57452500	-0.50559900
C	3.30445700	2.51181700	-0.59136900
C	2.07334300	1.89550200	-1.26187600
C	0.84127900	2.80192800	-1.23042000
C	-0.42629200	2.11852900	-1.74800800
C	-1.65631400	3.02558700	-1.73402200
H	-1.50241900	3.90726000	-2.36161000
H	-1.87144300	3.37859200	-0.72092500
H	-2.53832600	2.50029700	-2.10795500
H	-0.62823000	1.22923600	-1.13832400
H	-0.25596600	1.76277500	-2.77264500
H	1.03403700	3.70949700	-1.81340900
H	0.66964100	3.13591600	-0.19934300
H	1.82037100	0.95435200	-0.76164000
H	2.31473800	1.63494100	-2.30172300
H	3.02610600	2.84719200	0.41352700
H	3.59513900	3.41724200	-1.13390800
H	4.91058200	1.39598500	-1.51403700
H	5.31856600	2.08499900	0.04238300
H	5.16215200	-0.37062100	0.18414300
H	3.53996700	-0.35230800	-0.46634700
H	4.40686300	0.72707000	2.24740900
H	2.82026700	1.02394500	1.57724100
H	3.95306800	-1.69605400	2.44614500
H	1.69603900	-2.39013500	2.59770600
H	-0.06051800	-1.26965500	1.36337900
H	0.97055600	0.08316700	0.94039500
H	0.90664600	0.81858300	3.36684600
H	-0.25221600	-0.45214200	3.69011900
H	-0.63536500	1.90983600	1.77196300
H	-1.37117800	1.72509600	3.35242200
H	-2.51981100	-0.39198600	2.47552900
H	-1.90024200	0.01924600	0.88458300
H	0.86193200	-4.42346600	0.23473100
C	0.82895500	-3.58217100	-0.44327800
C	-0.38210300	-2.98917400	-0.74095800
N	-0.42225000	-1.93276800	-1.57304600
C	0.67933800	-1.41661200	-2.14447800
C	1.91388600	-1.97859200	-1.88344000
C	1.99124500	-3.06423900	-1.01305900
H	2.95271600	-3.50138200	-0.77617000
H	2.79808100	-1.55948200	-2.34173700
H	0.53271300	-0.55736900	-2.78278400
H	-1.34575200	-1.46544500	-1.73085600
H	-1.33284700	-3.30692000	-0.33510500

conf_83

C	-1.53767300	0.62448500	-1.71174300

C	-2.89477200	0.11837400	-2.20635600
C	-4.10267900	0.79748800	-1.54380600
C	-4.29655700	0.47422000	-0.04905200
C	-3.24573800	1.08804000	0.83954300
O	-2.90282300	2.25325300	0.78051100
O	-2.64378700	0.30260100	1.75526700
H	-2.91399800	-0.62333100	1.66521500
H	-4.34965000	-0.60662600	0.10649800
H	-5.25126300	0.89278800	0.28738500
H	-5.01482200	0.48648600	-2.05837600
H	-4.03221700	1.88264400	-1.65869800
H	-2.95958600	-0.96793400	-2.07037300
H	-2.96268800	0.29064200	-3.28513600
C	-0.32125200	0.05884100	-2.44787600
C	0.96856500	0.68814600	-1.91760200
C	2.27097300	0.06836400	-2.44784900
C	3.46653400	0.64401100	-1.74092900
C	4.36249100	-0.00720200	-0.99279400
C	4.43759400	-1.48834300	-0.72459700
C	3.76318400	-1.92305700	0.59372900
C	2.23952000	-1.83857400	0.52169200
C	1.50691300	-2.26009600	1.79617900
C	-0.01637000	-2.10676300	1.68024100
C	-0.68774900	-3.12489800	0.75301000
C	-2.17435200	-2.83998200	0.52528800
C	-2.87433300	-3.88253400	-0.34706500
H	-3.92929500	-3.64060000	-0.49957700
H	-2.82429300	-4.87328900	0.11118700
H	-2.40078100	-3.94781300	-1.33062500
H	-2.26427800	-1.85757300	0.04567100
H	-2.68349100	-2.78356900	1.49978700
H	-0.56842800	-4.13003200	1.17228000
H	-0.18612100	-3.13722300	-0.22003900
H	-0.24019600	-1.09727900	1.31154900
H	-0.47092800	-2.18268200	2.67527800
H	1.75426100	-3.29768500	2.04709000
H	1.86940600	-1.65855100	2.63933400
H	1.89876700	-2.45538700	-0.31639900
H	1.95200300	-0.81228100	0.27565500
H	4.06183800	-2.95145000	0.82169800
H	4.13658300	-1.30496600	1.42040700
H	5.49240600	-1.77557500	-0.69231000
H	3.99188400	-2.05242100	-1.54895100
H	5.15094200	0.58425600	-0.52998200
H	3.58747300	1.72137100	-1.84989200
H	2.23403600	-1.01579000	-2.32567300
H	2.34689700	0.26153800	-3.52459900
H	0.96403500	1.76178900	-2.14253800
H	0.96776100	0.60598100	-0.82539700
H	-0.28328900	-1.02872400	-2.31342200
H	-0.41403800	0.23582800	-3.52481300
H	-1.52447900	1.71914500	-1.78205700
H	-1.41040200	0.38605300	-0.64976800
H	2.23656100	0.73824800	2.56737400
C	1.74457200	1.50734300	1.98933800

C	0.36561800	1.59165700	1.99913400
N	-0.25096100	2.53461900	1.26404300
C	0.42298700	3.42285700	0.51186400
C	1.80287700	3.38342400	0.47371600
C	2.47115900	2.40697000	1.21153100
H	3.55053200	2.34226200	1.17529000
H	2.34145700	4.09490800	-0.13665200
H	-0.17450400	4.13188200	-0.04431500
H	-1.29685300	2.53290700	1.22681800
H	-0.28070300	0.92626800	2.55297400

conf_81

C	-0.52019600	-1.95860500	1.81243200
C	-1.76364900	-1.85188500	0.92393300
C	-1.78404300	-2.81945800	-0.26830300
C	-1.05159700	-2.30020000	-1.52829000
C	0.41049000	-2.02647300	-1.32676300
O	0.91129300	-0.91816100	-1.36892900
O	1.22324400	-3.07466900	-1.08258800
H	0.72487900	-3.90206800	-1.03151400
H	-1.50575200	-1.36835400	-1.86297800
H	-1.15185900	-3.03161100	-2.33809700
H	-2.81543200	-3.00327800	-0.57710500
H	-1.38673300	-3.79617900	0.03500700
H	-1.88169300	-0.82967300	0.55473900
H	-2.64336000	-2.04551000	1.54371800
C	-0.51284700	-0.95731500	2.97542600
C	-0.42146700	0.51947200	2.56582700
C	0.80897500	0.83534800	1.69610000
C	1.09147800	2.30697200	1.59694900
C	1.04666000	3.07646800	0.50409400
C	0.68073400	2.68356500	-0.90015100
C	-0.56240200	3.44947000	-1.40036100
C	-1.86781600	3.03658500	-0.71106300
C	-2.40346700	1.67533400	-1.16503600
C	-3.71323700	1.28291700	-0.47577100
C	-4.39543700	0.06585800	-1.10777100
C	-5.72371200	-0.32901100	-0.44916900
C	-5.58053900	-0.87812600	0.97275700
H	-4.95444100	-1.77631400	0.98406000
H	-6.55327800	-1.15120100	1.38829900
H	-5.12896600	-0.14838500	1.64968100
H	-6.21856100	-1.08077300	-1.07283300
H	-6.38878700	0.54194500	-0.44009500
H	-3.71479400	-0.79448400	-1.08279100
H	-4.57313100	0.28039500	-2.16775900
H	-4.40680400	2.13125900	-0.51733200
H	-3.52235400	1.10380100	0.58834400
H	-2.56132900	1.70350200	-2.25062100
H	-1.64875800	0.89863100	-0.99000200
H	-2.63099700	3.79389700	-0.91801300
H	-1.72607400	3.03762400	0.37595100
H	-0.39244500	4.52082800	-1.25120800

H	-0.65994500	3.30100000	-2.48114300
H	0.51378500	1.60826600	-0.99007700
H	1.51734300	2.93056400	-1.56796200
H	1.26381200	4.13561600	0.63292100
H	1.34317400	2.78821400	2.54026200
H	1.67999500	0.33798600	2.14855800
H	0.67367500	0.40059900	0.70490900
H	-1.32235100	0.83408000	2.02997100
H	-0.38522000	1.12909300	3.47405100
H	-1.41436800	-1.10504800	3.57961800
H	0.33427600	-1.18992600	3.63053400
H	-0.46898600	-2.97417900	2.22100100
H	0.39286800	-1.82777800	1.22311000
H	6.34660900	-2.03670600	-0.52285400
C	5.64728200	-1.22219800	-0.39358500
C	4.32457500	-1.38994400	-0.75560400
N	3.45379600	-0.37668000	-0.59680600
C	3.80755800	0.81619000	-0.08643100
C	5.12041400	1.03170900	0.29299000
C	6.04947300	0.00489100	0.13538500
H	7.08187800	0.15734000	0.42550400
H	5.40285600	1.99031900	0.70612900
H	3.02498900	1.55686700	0.02322600
H	2.46227100	-0.54560100	-0.88785400
H	3.91775600	-2.30473300	-1.16516200

conf_13

C	-3.93552300	0.52054500	-0.88803600
C	-3.99638400	1.42351300	0.34944900
C	-3.36244300	2.80884900	0.15701600
C	-1.83202300	2.84803700	0.37849300
C	-1.04531000	1.99133500	-0.57251700
O	-0.46680400	0.97121700	-0.24396600
O	-0.97530400	2.37045800	-1.85864200
H	-1.48470600	3.17694800	-2.01761700
H	-1.59493100	2.50340000	1.38479300
H	-1.47365400	3.87857100	0.28622100
H	-3.78800100	3.51361300	0.87502100
H	-3.61696200	3.20194400	-0.83484300
H	-3.52099200	0.93227800	1.20513000
H	-5.04710500	1.55682600	0.62480000
C	-4.55651000	-0.86635300	-0.67281500
C	-3.85503600	-1.74374400	0.37288100
C	-2.36659200	-2.00082700	0.07007600
C	-1.78379800	-3.05444800	0.96958200
C	-0.82520000	-2.91078900	1.89143800
C	-0.03858200	-1.67573300	2.23705300
C	1.45495300	-1.98886700	2.44127500
C	2.33209300	-0.75939700	2.70230100
C	2.56318900	0.11506600	1.46600900
C	3.47674800	1.31315900	1.73662200
C	3.84395000	2.11836800	0.48301100
C	2.66071900	2.74431700	-0.26959300

C	1.83600200	3.72368700	0.56860500
H	2.47036000	4.50391500	0.99805500
H	1.32756600	3.22073600	1.39518700
H	1.07709200	4.22231000	-0.04137600
H	1.99933800	1.95510900	-0.64127800
H	3.04695900	3.26443500	-1.15280400
H	4.39694100	1.46710000	-0.20567900
H	4.53908000	2.91631600	0.76574900
H	4.40215900	0.95722100	2.20202100
H	3.00429800	1.96735700	2.47748900
H	3.01388000	-0.50345600	0.67615500
H	1.60261200	0.46712800	1.08016100
H	3.30368200	-1.09083500	3.08372100
H	1.88383300	-0.15430200	3.49972800
H	1.54688800	-2.68643100	3.27987800
H	1.84304100	-2.52645100	1.56598500
H	-0.42664300	-1.25606900	3.17462700
H	-0.17564900	-0.89426200	1.48668600
H	-0.56942800	-3.78947300	2.48098800
H	-2.24129000	-4.03734100	0.87448000
H	-2.28757300	-2.33160800	-0.97627100
H	-1.81088400	-1.06523200	0.15453100
H	-3.94038300	-1.30320600	1.37101700
H	-4.37725000	-2.70419100	0.42542700
H	-5.60678700	-0.74666200	-0.38569000
H	-4.56192600	-1.39765100	-1.63118100
H	-4.46513000	1.01846100	-1.70799500
H	-2.90325000	0.39924500	-1.22919100
H	3.63980300	-0.89679600	-3.65796300
C	2.89864200	-1.26709800	-2.96321900
C	1.98506600	-0.39351000	-2.40647900
N	1.06272000	-0.85078700	-1.54108900
C	0.98414000	-2.14212200	-1.17646800
C	1.87577900	-3.05643600	-1.70768700
C	2.84278600	-2.61548600	-2.60862900
H	3.55158700	-3.31681300	-3.03168600
H	1.81078300	-4.09410200	-1.41048100
H	0.21667700	-2.40930800	-0.46153300
H	0.41348800	-0.15568800	-1.10784800
H	1.96029600	0.66667400	-2.61614600

conf_67

C	-4.26110100	2.41574400	0.00886500
C	-3.01611400	3.05703500	0.63387200
C	-1.70006500	2.57411500	0.01823000
C	-0.49035100	3.26691500	0.64859400
C	0.82834400	2.77984200	0.11467900
O	0.96228800	2.13688100	-0.90746400
O	1.94088600	3.07349900	0.82141600
H	1.73063700	3.57770800	1.61942400
H	-0.49569000	3.13770900	1.73784700
H	-0.52799400	4.35051200	0.47315500
H	-1.69879700	2.75690900	-1.05986700

H	-1.59543100	1.49506500	0.15003500
H	-3.00897300	2.84771600	1.71124600
H	-3.08362100	4.14643900	0.53234200
C	-4.32487100	0.89323200	0.16052900
C	-5.66652700	0.30053200	-0.27318200
C	-5.70511700	-1.23582800	-0.17818500
C	-4.83687100	-1.91036400	-1.20283100
C	-3.78549300	-2.70704100	-0.99686800
C	-3.18418000	-3.12582800	0.31615500
C	-1.65626100	-2.94443900	0.35297200
C	-1.22456700	-1.48325200	0.20984800
C	0.29072200	-1.26868300	0.14421800
C	1.04900300	-1.63344200	1.42434200
C	2.51840400	-1.20931800	1.38759800
C	3.32142400	-1.61434300	2.62483600
C	4.78142000	-1.16225700	2.56211300
H	5.33244500	-1.45631400	3.45795800
H	4.85408400	-0.07263500	2.47365700
H	5.29319300	-1.60374400	1.70051700
H	3.27907100	-2.70252500	2.74183100
H	2.84505300	-1.19411600	3.51760000
H	2.57092900	-0.11826400	1.27182600
H	2.99601300	-1.63914400	0.49626200
H	0.98900200	-2.71202400	1.60044700
H	0.55838000	-1.15806900	2.28306600
H	0.69938700	-1.85191500	-0.69293600
H	0.48094100	-0.21619000	-0.09360500
H	-1.63543400	-0.90432400	1.04707800
H	-1.68193800	-1.07042800	-0.69483900
H	-1.27368600	-3.36176600	1.28932800
H	-1.20492700	-3.53359300	-0.45599300
H	-3.40969000	-4.18506900	0.49057100
H	-3.63536100	-2.57499500	1.14571700
H	-3.29340600	-3.12481800	-1.87479900
H	-5.12700500	-1.72184100	-2.23548300
H	-5.43333400	-1.54373900	0.83510500
H	-6.73834700	-1.56785100	-0.33269500
H	-6.46568900	0.71817200	0.34857100
H	-5.88641800	0.60538600	-1.30361500
H	-3.52740400	0.42169800	-0.42055700
H	-4.14021200	0.62543100	1.20971800
H	-5.14701400	2.86116300	0.47389700
H	-4.31203600	2.67863800	-1.05475700
H	6.33321500	0.38860100	-0.42086500
C	5.41905200	0.16812900	-0.95404600
C	4.31738300	0.98641600	-0.79241000
N	3.17876100	0.71059100	-1.44952700
C	3.04810900	-0.34949500	-2.26434800
C	4.12101500	-1.19799900	-2.46352100
C	5.31941700	-0.93470100	-1.80106400
H	6.17068600	-1.58972900	-1.94034400
H	4.01559900	-2.04993600	-3.12098900
H	2.08008700	-0.48338800	-2.72646100
H	2.34494700	1.32372500	-1.27867600
H	4.29536400	1.85454200	-0.14801200

conf_10

C	-4.61267700	-0.58638100	0.75995700
C	-4.68018200	-1.49398800	-0.47379000
C	-4.11011800	-2.90370500	-0.26028200
C	-2.57726700	-3.00259000	-0.41745500
C	-1.77030500	-2.21860900	0.58159200
O	-0.97527400	-1.34959700	0.26768500
O	-1.91435200	-2.51021300	1.88127900
H	-2.59659900	-3.18227800	2.01815300
H	-2.27887900	-2.65484600	-1.40628300
H	-2.26796700	-4.05100500	-0.32961900
H	-4.53519100	-3.58831200	-0.99780200
H	-4.42784000	-3.29136500	0.71643300
H	-4.16365800	-1.02879500	-1.32027300
H	-5.72847000	-1.58470300	-0.77435700
C	-5.16916700	0.82400500	0.52030300
C	-4.36684900	1.68180900	-0.46751000
C	-2.90588600	1.90998000	-0.03708200
C	-2.19564700	2.90594700	-0.91146000
C	-1.22803300	2.66042200	-1.80105400
C	-0.58296000	1.33969500	-2.10876000
C	0.94772400	1.38307500	-1.95930300
C	1.59939800	0.00109300	-2.03015600
C	3.09346300	0.01800100	-1.70208500
C	3.70573600	-1.38227300	-1.60817700
C	5.19707200	-1.40252800	-1.25392900
C	5.52264100	-0.88387900	0.15097800
C	7.00706300	-1.00292000	0.50114400
H	7.62046300	-0.43123600	-0.20081300
H	7.33839600	-2.04403400	0.45691600
H	7.21770100	-0.62920000	1.50675100
H	4.92846800	-1.44957100	0.88304700
H	5.21465800	0.16424800	0.23825800
H	5.75463700	-0.81366800	-1.99214000
H	5.56932100	-2.42903600	-1.34170400
H	3.55674500	-1.89775800	-2.56307200
H	3.15131300	-1.97060100	-0.86268200
H	3.63232900	0.60068700	-2.45838500
H	3.24088500	0.55204800	-0.75595900
H	1.44521400	-0.42891200	-3.02653700
H	1.08292400	-0.67416600	-1.33659600
H	1.37522100	2.03954300	-2.72500200
H	1.20061400	1.85084500	-1.00054000
H	-0.82485800	1.05369800	-3.14018300
H	-0.98663400	0.54748900	-1.47423800
H	-0.85052800	3.50358700	-2.37656300
H	-2.55528800	3.92934400	-0.82671700
H	-2.91712300	2.27734800	0.99984700
H	-2.37773700	0.95356400	-0.03250200
H	-4.37310600	1.23594400	-1.46692400
H	-4.86147500	2.65256200	-0.57043400
H	-6.20195400	0.74394200	0.16442100

H	-5.21848300	1.34815000	1.48129300
H	-5.18407100	-1.05696500	1.56813400
H	-3.58553800	-0.50724600	1.12893700
H	1.00258200	3.65615900	2.04904000
C	1.28978500	2.61611900	1.97983100
C	0.37836200	1.68875900	1.50983600
N	0.73257600	0.39517500	1.41615200
C	1.95961700	-0.04652400	1.74322300
C	2.90687700	0.84005200	2.21695700
C	2.56531500	2.18653400	2.34114000
H	3.29385900	2.89846600	2.70924600
H	3.89371300	0.48017700	2.47090800
H	2.14476800	-1.10224600	1.60351200
H	0.03737600	-0.29443400	1.05002400
H	-0.62057500	1.94226000	1.18609900

conf_20

C	3.21345600	0.32849400	-0.56370100
C	3.10244100	1.85237400	-0.63834200
C	1.70843600	2.37549800	-0.99997500
C	1.17261500	1.89923400	-2.38644500
C	0.06840100	0.89283900	-2.24185500
O	-1.10801900	1.14846800	-2.43858800
O	0.36818300	-0.35013800	-1.82786200
H	1.30395200	-0.45259700	-1.58438900
H	1.98331400	1.47115100	-2.98330200
H	0.75393700	2.73401900	-2.94491300
H	1.72677500	3.46629400	-1.00790500
H	0.99947200	2.09704900	-0.21557400
H	3.83376200	2.23432300	-1.35940900
H	3.38131900	2.27555900	0.33221400
C	4.56812200	-0.15952700	-0.04192700
C	4.79457500	-1.67168700	-0.15339500
C	3.80745200	-2.54758200	0.64038400
C	2.48121200	-2.78451600	-0.03093800
C	1.27724200	-2.83317600	0.54618900
C	0.94989500	-2.63104600	2.00122800
C	-0.39402000	-1.92623700	2.23456900
C	-0.43750700	-0.49655400	1.69362000
C	-1.77615600	0.20603400	1.93905800
C	-1.88802300	1.58814400	1.28409300
C	-0.93173100	2.64053200	1.85141600
C	-1.03664500	3.99182300	1.14033600
C	-0.02366400	5.01650800	1.65208600
H	-0.12210000	5.97175500	1.13118900
H	1.00230400	4.66215700	1.50907200
H	-0.15936100	5.20553300	2.72065600
H	-2.05238600	4.38534600	1.26064000
H	-0.89715000	3.84302700	0.06227100
H	0.10178100	2.28203700	1.78315000
H	-1.13078900	2.77259400	2.92153300
H	-2.91569500	1.95585300	1.38976500
H	-1.70717300	1.49684800	0.20547500

H	-2.58552000	-0.43603500	1.56910500
H	-1.94561800	0.30019500	3.01783300
H	-0.22700100	-0.50896700	0.61863000
H	0.37402400	0.07747100	2.15346000
H	-1.19583900	-2.51679800	1.77162700
H	-0.61044700	-1.91549700	3.30763100
H	1.74636000	-2.07066100	2.49947200
H	0.92094600	-3.61114300	2.49406300
H	0.42963300	-3.09892100	-0.08561000
H	2.54183900	-3.01213400	-1.09499600
H	4.27361800	-3.53023700	0.78639300
H	3.67117400	-2.13427300	1.64416400
H	4.77346200	-1.96928400	-1.20932600
H	5.80730000	-1.88660800	0.19825900
H	5.36385300	0.35549000	-0.59117600
H	4.67223700	0.14763700	1.00520600
H	2.41894500	-0.06762300	0.07787200
H	3.08003600	-0.10049900	-1.57096100
H	-5.96664800	-0.69039900	-0.18713100
C	-4.97074200	-1.03121400	-0.43448200
C	-4.09202800	-0.17527100	-1.07128500
N	-2.85386200	-0.59684200	-1.37922800
C	-2.41326300	-1.83102200	-1.08531300
C	-3.25342300	-2.72747800	-0.45133300
C	-4.54652400	-2.32323100	-0.12422100
H	-5.22007500	-3.00953400	0.37417100
H	-2.89738900	-3.72108300	-0.21684300
H	-1.39040700	-2.04303700	-1.36260400
H	-2.18642500	0.07233800	-1.84761000
H	-4.33502300	0.84228900	-1.34450800

conf_124

C	4.89906200	-1.75410500	1.60488300
C	5.21160700	-0.37816200	0.99929400
C	5.27940100	-0.37323700	-0.53337400
C	5.23178200	1.04365800	-1.11441300
C	3.87008100	1.67313200	-0.95053800
O	2.83228500	1.04145900	-0.93873500
O	3.79593500	3.01357000	-0.83370800
H	4.67512100	3.41596900	-0.83850400
H	5.43144600	1.02899400	-2.19330300
H	5.99744300	1.68298800	-0.66185500
H	4.44999700	-0.93988300	-0.95926800
H	6.19914700	-0.85978100	-0.86772600
H	6.16557600	-0.01800000	1.39897200
H	4.45603700	0.34080300	1.33594800
C	3.51728600	-2.31751300	1.24031200
C	2.34215500	-1.45029000	1.70400600
C	0.98383300	-1.96163200	1.20659600
C	0.85306600	-1.87650900	-0.29775700
C	-0.01524300	-2.54257000	-1.06476000
C	-1.04589900	-3.53471300	-0.60844500
C	-2.44709400	-3.26375200	-1.18080800

C	-3.06532400	-1.94671600	-0.70589300
C	-4.48894200	-1.73540000	-1.22923100
C	-5.15746600	-0.43490800	-0.76602900
C	-5.45292300	-0.38486600	0.73860400
C	-6.28157700	0.82900900	1.17829200
C	-5.53935300	2.16413300	1.07343100
H	-6.17040500	2.99486000	1.39777600
H	-5.22636900	2.37312400	0.04578200
H	-4.65078400	2.15834300	1.71547600
H	-6.60227700	0.68491300	2.21459600
H	-7.19737900	0.87388700	0.57877900
H	-4.51951500	-0.40982300	1.31651900
H	-5.99292400	-1.29828600	1.01147300
H	-6.10322200	-0.31464100	-1.30581600
H	-4.53566900	0.41785200	-1.06808400
H	-4.46637900	-1.75583100	-2.32442700
H	-5.11214600	-2.58394000	-0.92284500
H	-2.43526300	-1.11200300	-1.04004200
H	-3.05796000	-1.92754200	0.39045000
H	-2.40059400	-3.27014200	-2.27632600
H	-3.10683700	-4.09032300	-0.89816400
H	-1.09748200	-3.56420400	0.48335500
H	-0.72908000	-4.53430500	-0.93183500
H	0.02550500	-2.37780400	-2.14094400
H	1.55954700	-1.21181900	-0.79204500
H	0.83154300	-2.99114200	1.54430700
H	0.18760500	-1.37451400	1.68625300
H	2.33755500	-1.40009900	2.79798600
H	2.47308000	-0.42312100	1.34691000
H	3.44984900	-2.47334600	0.15881900
H	3.41668500	-3.31318600	1.68526400
H	5.66911700	-2.46722500	1.29096300
H	4.98033800	-1.67990400	2.69465300
H	-2.79080200	1.06406600	0.35757900
C	-1.93383700	1.70632600	0.21394800
C	-0.71856400	1.15181300	-0.14484200
N	0.34689000	1.95375200	-0.32481200
C	0.29076700	3.28991100	-0.17329700
C	-0.90188100	3.88961100	0.18187600
C	-2.02653000	3.08702300	0.37825200
H	-2.97282300	3.53320500	0.65709400
H	-0.94769000	4.96324100	0.30209400
H	1.21525100	3.82515200	-0.34514000
H	1.26984400	1.54458100	-0.59053300
H	-0.55552900	0.09093200	-0.30160100

conf_14

C	-3.24039700	-1.09189800	0.66806400
C	-3.96155600	-1.86223000	-0.44412500
C	-3.19990200	-3.07093700	-1.00109000
C	-1.85695100	-2.72814900	-1.68107900
C	-0.75051000	-2.32818500	-0.74394300
O	-0.09251200	-1.30922700	-0.85188500

O	-0.45486500	-3.15937200	0.27056400
H	-1.05619200	-3.91674100	0.29265800
H	-1.98180000	-1.91574800	-2.39725800
H	-1.49556200	-3.60099500	-2.23807900
H	-3.81999200	-3.57013300	-1.74922200
H	-3.05247800	-3.81414600	-0.20546200
H	-4.19162100	-1.18581900	-1.27375700
H	-4.92634500	-2.21624400	-0.06586400
C	-4.11529000	-0.03618400	1.35834800
C	-4.70039300	1.02980700	0.42186500
C	-3.65727000	1.81466800	-0.38806600
C	-2.70557100	2.61161900	0.46081800
C	-1.43250200	2.88592200	0.16224100
C	-0.69025900	2.43149700	-1.07411600
C	0.67316500	3.11566400	-1.22813900
C	1.44561400	2.68563000	-2.48150200
C	1.95362400	1.23660900	-2.47008400
C	3.07824000	0.98229900	-1.46172000
C	3.56262800	-0.46888200	-1.44578200
C	4.76643900	-0.70157400	-0.52935800
C	5.15916700	-2.17461800	-0.40762500
H	6.02382500	-2.31042400	0.24640800
H	5.41393800	-2.59419900	-1.38451900
H	4.33787600	-2.78043200	-0.00606600
H	4.55620900	-0.28716800	0.46528500
H	5.61844300	-0.12679900	-0.90721800
H	3.82597500	-0.77548000	-2.46469800
H	2.73368500	-1.12783400	-1.15252400
H	2.75338500	1.26326100	-0.45167800
H	3.92272000	1.64234500	-1.69343800
H	1.12675800	0.54429300	-2.27948400
H	2.32541600	0.98914100	-3.47018700
H	2.29984800	3.35718200	-2.62065000
H	0.80059200	2.83281500	-3.35493600
H	0.51472600	4.19855500	-1.26138700
H	1.27842400	2.93300700	-0.33102700
H	-1.29360000	2.64193900	-1.96449200
H	-0.56526500	1.34088700	-1.06695900
H	-0.87965000	3.53590900	0.83916100
H	-3.12560900	3.04082000	1.36869500
H	-3.10277200	1.14555000	-1.05308300
H	-4.19231700	2.50952300	-1.04862200
H	-5.41011000	0.56753800	-0.27040000
H	-5.28595000	1.73450000	1.02154700
H	-4.93872400	-0.53825200	1.87731800
H	-3.51889400	0.45395200	2.13588200
H	-2.88540900	-1.79863400	1.42811100
H	-2.35256700	-0.59888300	0.26087200
H	0.64269600	2.21316800	3.38160900
C	1.11819100	1.33256100	2.97209800
C	0.58961400	0.73848900	1.84133200
N	1.18218700	-0.35234800	1.32529100
C	2.27603900	-0.91258600	1.86806700
C	2.84029500	-0.35853300	3.00130500
C	2.25605000	0.77904400	3.55751800

H	2.68602400	1.23110600	4.44293800
H	3.72350200	-0.80837700	3.43332700
H	2.66819700	-1.78612800	1.36749000
H	0.76674600	-0.77713600	0.46199800
H	-0.29121400	1.10911000	1.33109000

conf_101

C	-4.94288900	1.48274700	-0.56682400
C	-3.82230600	1.84054300	0.41052500
C	-3.22404600	3.22575700	0.16162900
C	-2.04934400	3.55883900	1.11367200
C	-0.95815100	2.53192900	0.98410500
O	-0.78461600	1.62595400	1.77896900
O	-0.17584100	2.58000300	-0.10510600
H	-0.39473400	3.33566000	-0.66712500
H	-2.38718600	3.54746400	2.14972400
H	-1.65306900	4.55477000	0.89310300
H	-3.98658300	3.99722800	0.29580200
H	-2.89326500	3.30237400	-0.88096800
H	-3.02950100	1.08920400	0.33513000
H	-4.19407300	1.78452100	1.44057700
C	-5.55489700	0.09589800	-0.31757200
C	-4.55051100	-1.06603400	-0.30225000
C	-3.69212800	-1.16851600	-1.57355700
C	-2.79418100	-2.37123000	-1.58247600
C	-1.46325300	-2.36362200	-1.69842200
C	-0.57788300	-1.14604700	-1.82364000
C	0.90448200	-1.46085800	-1.59377900
C	1.78177600	-0.21459400	-1.48304900
C	3.25023700	-0.52614700	-1.18944000
C	4.10664200	0.72316000	-0.97488700
C	5.57086700	0.41339800	-0.65783900
C	6.42760300	1.66386900	-0.44489100
C	7.88836100	1.34172600	-0.12584200
H	8.35219700	0.77281800	-0.93698600
H	7.97090200	0.74373700	0.78702500
H	8.47534000	2.25171100	0.01921700
H	5.99727500	2.26241600	0.36738400
H	6.37753200	2.28967600	-1.34344600
H	5.99884900	-0.18391900	-1.47215200
H	5.62429200	-0.21767200	0.23967300
H	3.67749600	1.32308900	-0.16054900
H	4.05500600	1.35396000	-1.87046400
H	3.31554600	-1.16587600	-0.29840400
H	3.66901200	-1.12026800	-2.00992400
H	1.38370300	0.44254900	-0.69978100
H	1.71197600	0.36467900	-2.41141600
H	1.01267300	-2.06072300	-0.68290400
H	1.26719500	-2.09955900	-2.40672800
H	-0.89783000	-0.36749100	-1.11962000
H	-0.69516500	-0.69815500	-2.81815500
H	-0.95006700	-3.32267800	-1.73154300
H	-3.29718700	-3.33365000	-1.51074400

H	-4.36351000	-1.22609100	-2.44027100
H	-3.10394300	-0.25767800	-1.70785400
H	-3.89318700	-0.97632100	0.57187100
H	-5.09944300	-2.00308500	-0.16362400
H	-6.09172000	0.10634500	0.63723000
H	-6.30806000	-0.09634000	-1.08874200
H	-5.73681500	2.23400500	-0.50247200
H	-4.55720400	1.53995400	-1.59094400
H	2.91902900	-2.71638500	2.39565500
C	1.89107100	-2.47936700	2.15882300
C	1.50326600	-1.15883800	2.04443800
N	0.22509800	-0.86121300	1.74973100
C	-0.70914600	-1.80367100	1.54008900
C	-0.37179100	-3.14045800	1.64043200
C	0.94007000	-3.48130400	1.96156200
H	1.22480100	-4.52261600	2.04852900
H	-1.12629400	-3.89280200	1.45946800
H	-1.70019900	-1.45792300	1.28439400
H	-0.06329700	0.14255500	1.68975600
H	2.17438300	-0.32171900	2.17643300

conf_8

C	4.53035600	0.53591500	-0.10526500
C	4.75434300	1.76259600	-0.99117700
C	3.62885600	2.80238400	-0.93962500
C	2.26991500	2.30074900	-1.49700300
C	1.18871000	2.06108000	-0.48041800
O	0.03971100	2.44564000	-0.62673500
O	1.47689800	1.37728600	0.63688300
H	2.37384400	1.00171200	0.63221600
H	2.40938500	1.35265200	-2.03233500
H	1.84920800	3.00045500	-2.21724500
H	3.92535000	3.67256500	-1.52764200
H	3.50701300	3.16732000	0.08589600
H	4.89361400	1.43841400	-2.02923700
H	5.68951600	2.24739400	-0.69510200
C	5.66071600	-0.49261400	-0.21198900
C	5.56352200	-1.67263400	0.76149400
C	4.26602200	-2.49902000	0.65946000
C	3.12118800	-1.97086100	1.47925700
C	1.83631800	-1.89283600	1.12343100
C	1.23501100	-2.25839000	-0.20970500
C	-0.10349100	-1.55648400	-0.46222100
C	-0.72676000	-1.87371000	-1.82185100
C	-2.01341100	-1.08920800	-2.10848600
C	-3.18437500	-1.43480800	-1.18366200
C	-4.47190700	-0.68532500	-1.53037200
C	-5.66024300	-1.06920700	-0.64621400
C	-6.90574400	-0.22218900	-0.90987200
H	-7.23364600	-0.31770400	-1.94846700
H	-6.71074500	0.84114400	-0.73042600
H	-7.73986100	-0.52149300	-0.27084300
H	-5.37095000	-0.98535500	0.40892600

H	-5.89515400	-2.12743700	-0.80300500
H	-4.73045700	-0.86878700	-2.57955400
H	-4.29497200	0.39675300	-1.45824200
H	-2.91908000	-1.22395700	-0.14022500
H	-3.37110800	-2.51447700	-1.23079600
H	-1.80128900	-0.01284600	-2.04646000
H	-2.31940900	-1.27469700	-3.14335400
H	-0.93115500	-2.94870900	-1.88926800
H	0.00542900	-1.65427800	-2.60739600
H	-0.79708300	-1.83343600	0.33992100
H	0.03961200	-0.47384800	-0.38410100
H	1.07984900	-3.34392500	-0.26014800
H	1.93371800	-2.02540900	-1.02149100
H	1.12741100	-1.56016700	1.88043700
H	3.38234700	-1.68470700	2.49690300
H	3.97820100	-2.62030400	-0.38976200
H	4.47848600	-3.51139500	1.02542400
H	6.41647000	-2.33060600	0.57504500
H	5.67967000	-1.31178900	1.79041200
H	5.68741400	-0.87469200	-1.23943300
H	6.61662900	0.01756800	-0.05222700
H	4.43373800	0.85327500	0.94358700
H	3.59577200	0.03205400	-0.38634100
H	-2.19195300	-0.53444700	3.60396900
C	-2.48490500	0.13146400	2.80399500
C	-1.51938100	0.68226700	1.98224300
N	-1.88305500	1.50827700	0.98682600
C	-3.16436000	1.83173500	0.74315100
C	-4.16825300	1.30919800	1.53596100
C	-3.82339900	0.44942400	2.57754400
H	-4.59569300	0.02628100	3.20804500
H	-5.19875500	1.56487700	1.33342800
H	-3.34170700	2.50191000	-0.08642900
H	-1.12503600	1.90636200	0.37063100
H	-0.45848200	0.49523000	2.07366300

conf_94

C	3.63578500	-1.86914000	-0.30585500
C	4.93266500	-1.38450400	-0.96478200
C	4.87022300	-0.00759900	-1.63967400
C	4.60945800	1.14701900	-0.66083200
C	3.15933600	1.43753500	-0.38112800
O	2.25330800	1.23393200	-1.16711300
O	2.85457700	2.04077900	0.78444600
H	3.63358900	2.12585500	1.35057500
H	4.99784800	2.08533500	-1.07876700
H	5.14586400	0.98693400	0.28079800
H	4.10992500	0.01458500	-2.42355400
H	5.82739800	0.18348400	-2.13005800
H	5.24346800	-2.11748300	-1.71646800
H	5.73144000	-1.36608100	-0.21411700
C	2.50564800	-2.15631000	-1.30026100
C	1.28464600	-2.86997600	-0.70607300

C	0.49220500	-2.07287200	0.34463900
C	1.12030900	-2.02801100	1.71302500
C	1.06887700	-1.03349500	2.60219600
C	0.36912100	0.28606300	2.44012700
C	-0.96091600	0.35776600	3.22130900
C	-2.03488200	-0.61103600	2.71336000
C	-2.59131600	-0.25036200	1.33387700
C	-3.57193600	-1.28317700	0.77719600
C	-4.08717000	-0.93508600	-0.62006000
C	-5.07288800	-1.95542900	-1.19242600
C	-5.56424100	-1.58829500	-2.59345500
H	-6.07688800	-0.62086700	-2.59150800
H	-6.26598900	-2.33091800	-2.97942700
H	-4.73059200	-1.52441400	-3.30068900
H	-5.92846700	-2.04725800	-0.51434800
H	-4.59554900	-2.94153800	-1.21598600
H	-3.23242400	-0.84163100	-1.30506000
H	-4.56917400	0.05206200	-0.59311500
H	-4.42013600	-1.38874300	1.46340200
H	-3.08292000	-2.26425500	0.74720900
H	-3.08849500	0.72627300	1.39315300
H	-1.76760400	-0.13489100	0.62020800
H	-2.86089500	-0.63895600	3.43141800
H	-1.61945500	-1.62370700	2.68703500
H	-0.75632000	0.15191000	4.27664900
H	-1.34599700	1.38497000	3.18079500
H	0.18446100	0.47973000	1.38210100
H	1.02515700	1.08848600	2.79564500
H	1.53936500	-1.19521700	3.56967800
H	1.63563200	-2.93969200	2.00801800
H	0.30275400	-1.06142400	-0.03035300
H	-0.49515300	-2.54202500	0.44615200
H	0.60629500	-3.12323100	-1.52706500
H	1.59553600	-3.82557200	-0.26759200
H	2.17869000	-1.22332200	-1.77090200
H	2.90685200	-2.78082500	-2.10705700
H	3.86010300	-2.78665000	0.24873100
H	3.29915800	-1.15474600	0.45367000
H	-2.08975000	4.02068300	1.18169800
C	-1.85728800	3.36684100	0.35258300
C	-0.57278200	2.88041900	0.20373200
N	-0.28290800	2.06498200	-0.82489800
C	-1.20103900	1.68385800	-1.72946600
C	-2.50098700	2.14058500	-1.62449600
C	-2.83058600	2.99387400	-0.57327100
H	-3.84451100	3.36058100	-0.47138000
H	-3.23935300	1.82513900	-2.34784000
H	-0.85900600	1.01896100	-2.51056500
H	0.70466100	1.71844400	-0.92209800
H	0.24430900	3.10545600	0.87503400

conf_51

C	-3.87608200	2.66165000	-0.30414700

C	-2.41250100	3.10258900	-0.18398200
C	-1.72720200	2.62871500	1.10430300
C	-0.22872800	2.93577300	1.15155900
C	0.63907600	2.19529300	0.16555800
O	1.76730200	2.55664100	-0.12367600
O	0.18505300	1.06007900	-0.38444900
H	-0.71631100	0.85610700	-0.09242000
H	0.18734000	2.68909000	2.13627400
H	-0.02905500	3.99914000	1.00064400
H	-1.89262500	1.55498700	1.26463400
H	-2.20067700	3.10830500	1.96535600
H	-2.34634600	4.19434400	-0.23331700
H	-1.86646900	2.72877200	-1.06009800
C	-4.05365900	1.14023700	-0.28135600
C	-5.42942200	0.67212600	-0.75686300
C	-5.61907700	-0.85151200	-0.63246000
C	-4.60460200	-1.62144400	-1.42937600
C	-3.70864900	-2.50056300	-0.97285700
C	-3.52975400	-2.95375700	0.45233100
C	-2.35596400	-2.28028400	1.18963400
C	-0.99987900	-2.45055000	0.50097400
C	0.17828700	-1.97240700	1.35376000
C	1.50865800	-2.00921300	0.59724900
C	2.74808600	-1.69890600	1.44475200
C	2.73613400	-0.32824600	2.12903600
C	4.05208000	-0.00869400	2.83985000
H	4.89599000	-0.02908800	2.14164700
H	4.26495400	-0.74416600	3.62013500
H	4.02899800	0.97694900	3.31124900
H	1.91742000	-0.28601200	2.85391900
H	2.52035700	0.44894400	1.38754000
H	3.64004400	-1.77058700	0.80821800
H	2.86816800	-2.47545500	2.20899800
H	1.44775500	-1.29942700	-0.23553600
H	1.63060600	-3.00064800	0.14610300
H	-0.01878000	-0.95359500	1.70967600
H	0.24951700	-2.59373800	2.25439200
H	-1.00409700	-1.91289700	-0.45586800
H	-0.85021400	-3.50733900	0.24831900
H	-2.57388000	-1.21112100	1.30502500
H	-2.29944500	-2.69419400	2.20278000
H	-3.36092900	-4.03684000	0.45502500
H	-4.44387600	-2.78694300	1.02593100
H	-3.03937200	-2.95508100	-1.69978100
H	-4.60940700	-1.41367300	-2.49869700
H	-5.57999900	-1.13226400	0.42317500
H	-6.62421800	-1.10637200	-0.98750300
H	-6.21058800	1.17676100	-0.17853200
H	-5.57230900	0.97502100	-1.80076400
H	-3.28845000	0.67282600	-0.91543200
H	-3.88658800	0.75551900	0.73149100
H	-4.46863900	3.11597800	0.49804700
H	-4.27819000	3.05868200	-1.24182400
H	3.64817900	-1.85213900	-3.17273000
C	3.98525800	-1.08725600	-2.48689900

C	3.09823600	-0.12288700	-2.04642600
N	3.51369900	0.82762400	-1.19303000
C	4.77380900	0.88589200	-0.72968200
C	5.70092100	-0.05399300	-1.13856700
C	5.30096800	-1.05138300	-2.02740500
H	6.01146300	-1.79888500	-2.35836500
H	6.71413000	-0.00558200	-0.76413500
H	4.99412500	1.68727900	-0.03820300
H	2.81189100	1.53121900	-0.84285100
H	2.05883000	-0.07808300	-2.34021600

conf_73

C	-2.96226000	1.81189400	0.55453100
C	-1.49124200	2.11122600	0.26274300
C	-1.29240100	2.76782100	-1.10593900
C	0.15117600	3.21779900	-1.37491400
C	1.17876300	2.11826100	-1.34345300
O	2.28078500	2.24245000	-0.83712800
O	0.88021100	0.95082400	-1.92974000
H	-0.03456900	0.94119000	-2.24743600
H	0.47059600	3.96613500	-0.64914700
H	0.22677500	3.68214200	-2.36623200
H	-1.93165200	3.65089300	-1.18872600
H	-1.64636200	2.09165000	-1.89769800
H	-0.91377400	1.17968300	0.31527800
H	-1.08172000	2.76594600	1.04132700
C	-3.17140900	1.05410700	1.86874900
C	-4.63794600	0.76233500	2.22011400
C	-5.29648400	-0.38603300	1.42766700
C	-5.57541100	-0.08129200	-0.01755500
C	-5.16497900	-0.77453500	-1.08222300
C	-4.28947800	-2.00019200	-1.06262900
C	-2.78955700	-1.66069600	-1.10788000
C	-1.89667500	-2.90310300	-1.14158900
C	-0.39603600	-2.59920000	-1.06231800
C	0.06130200	-2.06161200	0.29752000
C	1.56459500	-1.77977400	0.34601100
C	2.08520200	-1.32650800	1.71595700
C	1.54102100	0.02826400	2.17843600
H	1.74508500	0.81424400	1.44418400
H	1.98891300	0.33163900	3.12823900
H	0.45966500	-0.00179600	2.32708700
H	3.18080000	-1.28754400	1.68853600
H	1.83907900	-2.08970900	2.46205700
H	1.79209700	-1.01635600	-0.40734400
H	2.10222600	-2.68670300	0.04343000
H	-0.19678400	-2.78959700	1.07633100
H	-0.49012200	-1.14864000	0.54059800
H	0.16659400	-3.51302200	-1.28474800
H	-0.12599500	-1.88042800	-1.84830800
H	-2.10445200	-3.46470100	-2.05860000
H	-2.17049100	-3.56607100	-0.31166700
H	-2.58378800	-1.03849600	-1.98983700

H	-2.55114600	-1.04645700	-0.23509000
H	-4.53784200	-2.63736200	-1.91767100
H	-4.48377700	-2.59977700	-0.16867500
H	-5.45701000	-0.41413500	-2.06599700
H	-6.18979500	0.80078100	-0.19094200
H	-4.67936100	-1.28372700	1.52462100
H	-6.25177200	-0.61622000	1.91573000
H	-4.69255600	0.50449200	3.28208900
H	-5.23100300	1.67683700	2.09959900
H	-2.61686800	0.10676200	1.83660500
H	-2.72456900	1.63707900	2.68284600
H	-3.51965500	2.75549200	0.59179300
H	-3.38711000	1.24057300	-0.27299000
H	6.49725600	0.39067500	2.17306300
C	5.95789600	0.09728900	1.28305000
C	4.92643700	0.88902200	0.81436800
N	4.25421300	0.52724000	-0.29149200
C	4.53583500	-0.59325500	-0.97654900
C	5.55650600	-1.42190200	-0.54993800
C	6.27515100	-1.07187200	0.59254900
H	7.07758800	-1.70856900	0.94442500
H	5.78006000	-2.32435500	-1.10193500
H	3.92330400	-0.79266700	-1.84456100
H	3.45859000	1.13918200	-0.60471700
H	4.60603500	1.80581100	1.28961200

conf_39

C	3.26872100	0.86070900	0.63266000
C	4.16236100	1.64881700	-0.33335800
C	3.58923400	2.99375500	-0.79839800
C	2.24214800	2.90369600	-1.54828400
C	1.05495300	2.57775400	-0.68185000
O	0.28191800	1.66155400	-0.89313600
O	0.82978100	3.35389700	0.39239500
H	1.51364300	4.03117200	0.48830300
H	2.28436500	2.14932200	-2.33415500
H	2.02841600	3.86517700	-2.03014100
H	4.30300800	3.47090500	-1.47383700
H	3.50566200	3.67280800	0.06093700
H	4.38190600	1.03766800	-1.21488200
H	5.12670600	1.84436100	0.14726500
C	3.98230000	-0.29906300	1.33992800
C	4.59941100	-1.35182500	0.40906400
C	3.61430500	-2.01239300	-0.56581800
C	2.48526900	-2.74444200	0.11743200
C	1.32177800	-3.10093100	-0.43696700
C	0.90037200	-2.83961000	-1.85773100
C	-0.19178700	-1.76382900	-2.00956600
C	-1.51875300	-2.10718400	-1.32899800
C	-2.59213400	-1.03550900	-1.53203500
C	-3.88419700	-1.31940300	-0.76048400
C	-5.01087500	-0.31213600	-1.02130700
C	-4.75611400	1.09056600	-0.46011500

C	-5.89122200	2.07003100	-0.76400100
H	-6.83886300	1.71266000	-0.35139600
H	-6.02519500	2.18874000	-1.84269800
H	-5.69574900	3.05961900	-0.34269200
H	-3.81779300	1.48888100	-0.86429400
H	-4.62536900	1.01285100	0.62757700
H	-5.94219100	-0.69510600	-0.58997600
H	-5.18503600	-0.24060400	-2.10158900
H	-3.66780500	-1.35427600	0.31687400
H	-4.23580400	-2.32226500	-1.02593900
H	-2.18645200	-0.05734400	-1.24982100
H	-2.82258800	-0.95736500	-2.60100400
H	-1.36318100	-2.26360300	-0.25465900
H	-1.88727900	-3.06579400	-1.71292900
H	0.18546500	-0.80823600	-1.62806200
H	-0.37523600	-1.60490800	-3.07774200
H	0.52881700	-3.77825500	-2.28563400
H	1.76608500	-2.55242900	-2.45836400
H	0.62159200	-3.66779700	0.17327000
H	2.67632800	-3.05251200	1.14447400
H	3.21588600	-1.26861900	-1.26286600
H	4.17441400	-2.72363100	-1.18518800
H	5.41580000	-0.90397700	-0.16516400
H	5.06143500	-2.12930200	1.02655700
H	4.77175200	0.10763100	1.98086100
H	3.26852400	-0.78519000	2.01510500
H	2.87561200	1.53864100	1.40031500
H	2.40425900	0.46859500	0.08837500
H	-0.22215300	-2.30048000	2.94558200
C	-0.73275600	-1.39150500	2.65878800
C	-0.27808700	-0.67326100	1.56835900
N	-0.91326400	0.45381000	1.20406400
C	-1.99009400	0.92601800	1.85492200
C	-2.48492300	0.24267000	2.94879600
C	-1.84692200	-0.92902200	3.35624900
H	-2.21989800	-1.47945100	4.21129700
H	-3.35478800	0.62114200	3.46753800
H	-2.42363700	1.83712100	1.46881000
H	-0.54665800	0.98141300	0.37783400
H	0.56734300	-0.98126700	0.96581700

conf_87

C	3.68878800	1.30366100	-1.47734300
C	5.05080200	0.65569100	-1.75658100
C	5.46621600	-0.49310000	-0.82865800
C	4.58607700	-1.75529200	-0.95215500
C	3.26466600	-1.72402100	-0.23635700
O	2.19875800	-2.02788500	-0.73826200
O	3.26812400	-1.39290400	1.07101800
H	4.15051500	-1.12446700	1.36339500
H	5.12449000	-2.61629700	-0.53619300
H	4.38009200	-1.98211900	-1.99827400
H	5.51564900	-0.14504400	0.21125300

H	6.48716300	-0.79033300	-1.07849100
H	5.82487300	1.42819800	-1.70473200
H	5.06467300	0.28135200	-2.78598300
C	3.60020300	2.04505300	-0.13750500
C	2.38084600	2.97108400	-0.02701000
C	1.00939900	2.27540600	-0.14252300
C	0.75487000	1.27533400	0.94869200
C	-0.16418500	1.35472400	1.91518800
C	-1.16777200	2.45055300	2.14385800
C	-2.60061800	2.07278200	1.71936700
C	-2.77237500	1.93496500	0.20607800
C	-4.21713500	1.65419600	-0.21783400
C	-4.39801200	1.50308100	-1.73585100
C	-3.87186100	0.18374900	-2.31987000
C	-4.76656200	-1.02553300	-2.02631200
C	-4.18756400	-2.34578800	-2.53717000
H	-4.86059400	-3.18364100	-2.34063100
H	-3.22706500	-2.57728200	-2.06300400
H	-4.01378900	-2.30538100	-3.61592700
H	-5.74515000	-0.85595100	-2.48753800
H	-4.96728700	-1.09978100	-0.95014300
H	-2.85441600	-0.00462800	-1.95130300
H	-3.77306000	0.28289800	-3.40590100
H	-3.89890900	2.34379500	-2.22967800
H	-5.46041500	1.59351100	-1.98574400
H	-4.59035100	0.76018800	0.29841000
H	-4.84797500	2.47650300	0.13527200
H	-2.10126700	1.15355300	-0.16633100
H	-2.44102000	2.86274600	-0.27573900
H	-2.89464400	1.13753400	2.21375700
H	-3.28879900	2.83926500	2.09057200
H	-0.87237900	3.36339500	1.62067700
H	-1.17169400	2.69569100	3.21118200
H	-0.20683100	0.54055200	2.63755900
H	1.41812900	0.41447500	0.95859700
H	0.23017100	3.03882400	-0.14861600
H	0.94245400	1.77034400	-1.11402800
H	2.42138500	3.50353400	0.92821200
H	2.44829300	3.73195900	-0.81218000
H	3.59673500	1.33657300	0.69790800
H	4.50279500	2.65327600	-0.01186500
H	3.49246000	2.01999500	-2.28257000
H	2.89178600	0.55622400	-1.55709400
H	-1.09398300	-2.54532400	3.83989500
C	-1.15662200	-2.29468100	2.78992400
C	-0.01424000	-2.30875000	2.01303600
N	-0.09032900	-1.99914500	0.70776000
C	-1.24213100	-1.65426800	0.11109400
C	-2.41388400	-1.62352100	0.84249600
C	-2.37027100	-1.94900300	2.19573700
H	-3.27723300	-1.92834600	2.78735000
H	-3.33621800	-1.34291500	0.35623900
H	-1.18787200	-1.40936800	-0.94001100
H	0.79401300	-2.00271100	0.14426400
H	0.97070600	-2.54841100	2.38924100

conf_119

C	-4.43710400	0.00959800	-0.54283900
C	-5.49458000	-1.01518800	-0.96454400
C	-5.25033900	-2.44995100	-0.48127100
C	-3.89980300	-3.05303400	-0.90650500
C	-2.67831300	-2.55551200	-0.17971200
O	-1.59438300	-2.38461800	-0.70828200
O	-2.76921200	-2.33822300	1.14473800
H	-3.67648200	-2.46795700	1.45589700
H	-3.71844400	-2.90374900	-1.97167800
H	-3.91220200	-4.13782800	-0.73972500
H	-6.03591500	-3.09882200	-0.87540600
H	-5.37381300	-2.50092100	0.61108100
H	-5.57010900	-1.01872300	-2.05771800
H	-6.47460000	-0.69636200	-0.59512700
C	-4.86477700	1.45140100	-0.83792500
C	-3.73589200	2.48025900	-0.71520800
C	-2.99223600	2.45677800	0.63476200
C	-2.06415700	3.63343200	0.79013500
C	-0.83074700	3.73914700	0.29019500
C	-0.08258000	2.69942300	-0.49547000
C	1.40014100	2.61653300	-0.11212100
C	2.16221500	1.53453400	-0.87668300
C	3.63059800	1.41479800	-0.46736800
C	4.36799200	0.27116900	-1.16556800
C	5.78044800	0.03548400	-0.62877400
C	6.50956900	-1.12475600	-1.31048700
C	7.91692600	-1.35648400	-0.75843400
H	7.89049800	-1.58451700	0.31181700
H	8.54174200	-0.46865300	-0.89100500
H	8.41204000	-2.18905500	-1.26353300
H	6.56440400	-0.93024800	-2.38746600
H	5.91529600	-2.04114400	-1.19939000
H	5.73235600	-0.15336200	0.45329900
H	6.37052400	0.95248500	-0.74284000
H	4.41341200	0.47134000	-2.24224100
H	3.78460400	-0.65436300	-1.06400900
H	4.14585400	2.35992900	-0.67188300
H	3.69238700	1.27734500	0.62015900
H	2.09720900	1.73284900	-1.95281700
H	1.66536900	0.56684200	-0.72637400
H	1.87118000	3.58974300	-0.28925400
H	1.48658800	2.43635600	0.96625000
H	-0.15093800	2.93670800	-1.56555300
H	-0.55402600	1.71592800	-0.38221500
H	-0.30061000	4.67762700	0.43848500
H	-2.47535200	4.49494100	1.30942300
H	-3.72305900	2.45913600	1.45069500
H	-2.43513400	1.51635500	0.71836000
H	-3.00523200	2.32393900	-1.51674800
H	-4.14806100	3.48114300	-0.87416400
H	-5.27681400	1.50435600	-1.85158600

H	-5.68412500	1.72127000	-0.16129100
H	-4.23178200	-0.08918800	0.52932900
H	-3.49292200	-0.19430600	-1.06388200
H	0.88796400	0.86459100	3.14874800
C	1.13029000	0.06105800	2.46735400
C	0.14067300	-0.47931000	1.66897500
N	0.43867800	-1.47339300	0.81475900
C	1.67904500	-1.97717100	0.69367200
C	2.70432600	-1.47489200	1.47187500
C	2.42562100	-0.44507800	2.36885600
H	3.21618000	-0.03530500	2.98499300
H	3.70281400	-1.87561400	1.36709800
H	1.80885800	-2.76771000	-0.03255500
H	-0.33592800	-1.85957200	0.21736300
H	-0.88961100	-0.15242300	1.68300600

conf_147

C	-4.53775400	0.91914500	-1.72510300
C	-5.93763200	0.92525300	-1.09948800
C	-6.05888300	0.26040800	0.28055100
C	-5.55350200	-1.20477500	0.32998800
C	-4.07622300	-1.35270200	0.57639000
O	-3.28785300	-1.86664000	-0.19376000
O	-3.59464600	-0.89734300	1.75153500
H	-4.29139900	-0.49631800	2.28925500
H	-5.78616200	-1.72711500	-0.59766700
H	-6.06133000	-1.73761300	1.14218800
H	-7.11180900	0.25534900	0.56992400
H	-5.54803000	0.86475700	1.03852800
H	-6.62905100	0.42201000	-1.78348400
H	-6.29655700	1.95572300	-1.00847000
C	-3.46574100	1.64461900	-0.90677000
C	-2.06084800	1.46202000	-1.48748200
C	-0.94451100	2.08828700	-0.63979400
C	-0.74133900	1.41891300	0.69649500
C	0.36345100	1.46878700	1.44579500
C	1.64031100	2.19047000	1.11926600
C	2.88438900	1.46844300	1.65134700
C	4.20711900	2.18142300	1.34967100
C	4.51724900	2.36857100	-0.14434400
C	4.46677100	1.08623300	-0.98665700
C	5.40589800	-0.02417500	-0.50799500
C	5.45932200	-1.22859400	-1.45185900
C	6.33470400	-2.36812100	-0.92804100
H	5.96881300	-2.74280700	0.03396600
H	7.36342200	-2.03109000	-0.77491200
H	6.36125800	-3.20887200	-1.62526100
H	5.82683000	-0.90097000	-2.43024800
H	4.44203500	-1.60259000	-1.63200000
H	5.11041900	-0.36050200	0.49410500
H	6.41779500	0.38189700	-0.39298800
H	4.71722400	1.34059100	-2.02248100
H	3.43749800	0.70449500	-1.01561100

H	3.82677400	3.10052500	-0.57580100
H	5.51447900	2.81195100	-0.23541700
H	5.02009100	1.62228900	1.82334700
H	4.20122400	3.16539000	1.83112100
H	2.78553800	1.34580600	2.73598300
H	2.91145900	0.45618900	1.23565900
H	1.60646100	3.19700900	1.55854800
H	1.72141600	2.34270300	0.03978100
H	0.34337500	0.97054200	2.41426900
H	-1.59628100	0.88237400	1.10101200
H	-0.00792500	2.08267800	-1.20636500
H	-1.18126300	3.14874700	-0.48088900
H	-1.87064600	0.38861400	-1.61222000
H	-2.03194800	1.89225200	-2.49370800
H	-3.70220200	2.71367300	-0.85355800
H	-3.47538300	1.28983400	0.12828300
H	-4.21991700	-0.11403800	-1.90177900
H	-4.60188900	1.37978000	-2.71600600
H	1.78439000	-2.31232900	2.40949200
C	1.33693700	-2.13344500	1.44172800
C	-0.03845800	-2.12040800	1.31458200
N	-0.59704800	-1.89784100	0.11401300
C	0.13132200	-1.66289100	-0.98995300
C	1.51054600	-1.66050800	-0.91739200
C	2.12014200	-1.90338300	0.31193300
H	3.20008600	-1.90858300	0.38551800
H	2.09598100	-1.47101000	-1.80600200
H	-0.42179600	-1.48577800	-1.90137700
H	-1.64191400	-1.88394100	0.03957800
H	-0.72136100	-2.27627100	2.13818200

conf_103

C	4.84272700	-1.39219400	-0.01202200
C	3.82308700	-2.52449300	-0.19940000
C	3.03755000	-2.86378900	1.08201700
C	1.68140700	-3.52544500	0.82353900
C	0.63741000	-2.65801000	0.16280600
O	-0.37481800	-3.10193200	-0.34909300
O	0.80306800	-1.32422200	0.15656000
H	1.64745800	-1.05753500	0.55156300
H	1.22639300	-3.86593400	1.76119900
H	1.77940700	-4.41734600	0.19970000
H	2.89825600	-1.97147600	1.70631300
H	3.63270000	-3.53563700	1.70455900
H	4.32518700	-3.43047500	-0.54955900
H	3.13456800	-2.23833300	-1.00516800
C	4.18346600	-0.01895400	0.14808900
C	5.17248300	1.13834800	0.30526600
C	4.47814000	2.50246100	0.50045700
C	3.71945300	2.96362000	-0.71517800
C	2.41466700	2.81301700	-0.95730200
C	1.40742900	2.13590400	-0.07128000
C	0.04438500	2.83513200	-0.00508200

C	-0.94644800	2.08138500	0.88506600
C	-2.36823500	2.64581800	0.85607600
C	-3.36785700	1.85496800	1.70998400
C	-3.63973300	0.43764300	1.19164400
C	-4.73334600	-0.31922400	1.95589500
C	-4.37704200	-0.62112200	3.41363100
H	-5.15676700	-1.21686400	3.89346900
H	-4.25765300	0.29313400	3.99920900
H	-3.43995200	-1.18380300	3.48021700
H	-4.94045900	-1.26274300	1.43664100
H	-5.66396700	0.25852200	1.91738000
H	-2.71636100	-0.15392500	1.22191600
H	-3.93171400	0.51012700	0.13670000
H	-4.31725100	2.40067300	1.74679000
H	-3.00094900	1.81175000	2.74062600
H	-2.34488400	3.68618800	1.19671600
H	-2.72715800	2.67765900	-0.18178300
H	-0.58004600	2.08855400	1.91891100
H	-0.95885100	1.02660100	0.58825600
H	0.17155900	3.85972700	0.35865500
H	-0.36415700	2.92755400	-1.02077200
H	1.80243900	2.01829800	0.94187000
H	1.24401500	1.11584300	-0.44702700
H	2.02512800	3.19365000	-1.90007000
H	4.31899900	3.45095100	-1.48055300
H	3.81835600	2.44085700	1.37178500
H	5.24027100	3.24865300	0.74291100
H	5.82276000	0.94682900	1.16538100
H	5.82359100	1.17989700	-0.57500800
H	3.54689400	0.17518800	-0.72272000
H	3.52688100	-0.01558500	1.03083900
H	5.47904200	-1.60633100	0.85451700
H	5.50788100	-1.36555100	-0.88043900
H	-1.95399300	1.73317700	-2.79285900
C	-2.35946000	0.76658100	-2.52902600
C	-1.56501600	-0.14873100	-1.86448400
N	-2.06565500	-1.35053500	-1.53147800
C	-3.32950800	-1.71246500	-1.81124600
C	-4.16496800	-0.83411600	-2.47519800
C	-3.67207500	0.41797100	-2.84140300
H	-4.31086900	1.12056300	-3.36230400
H	-5.18131600	-1.12751000	-2.69886600
H	-3.62912800	-2.70110400	-1.49170200
H	-1.43347400	-2.03206600	-1.03884800
H	-0.54035600	0.03497800	-1.57493900

conf_120

C	4.11631900	1.05621000	0.29958900
C	5.52049600	0.73181200	-0.21366200
C	5.61571000	-0.57050100	-1.01859800
C	5.07236900	-1.81346100	-0.28385400
C	3.57436400	-1.96842200	-0.24664300
O	2.94062000	-2.29713300	0.73966100

O	2.89539400	-1.79685200	-1.39684400
H	3.47675000	-1.47306300	-2.09927000
H	5.42449200	-1.84454200	0.74753000
H	5.44529500	-2.72086300	-0.77559100
H	6.66101800	-0.76639900	-1.26774400
H	5.11306900	-0.44137600	-1.98809500
H	6.20951200	0.67242400	0.63661700
H	5.88409300	1.55085800	-0.84270600
C	4.01765200	2.44078400	0.95114400
C	2.75188300	2.67099600	1.78777100
C	1.42149600	2.66931300	1.01526400
C	1.00009300	1.31649100	0.50601400
C	0.34129600	1.03977200	-0.62079700
C	-0.17668900	2.00269400	-1.64952200
C	-1.71633400	1.98145700	-1.71736400
C	-2.38159400	2.55846100	-0.46354300
C	-3.89844600	2.33840700	-0.40367100
C	-4.29231100	0.88360500	-0.13213300
C	-5.80095500	0.65193100	-0.04301700
C	-6.17567800	-0.80711300	0.23107500
C	-7.68459900	-1.03678000	0.33116600
H	-7.92005600	-2.08595700	0.52562000
H	-8.18616700	-0.74831100	-0.59675000
H	-8.11762300	-0.44186100	1.14010200
H	-5.69734100	-1.13246200	1.16523000
H	-5.76649500	-1.43929000	-0.56907700
H	-6.27412700	0.97570800	-0.97748300
H	-6.22066400	1.28671900	0.74627800
H	-3.82231100	0.56669600	0.80928300
H	-3.88000800	0.23686900	-0.91663100
H	-4.32280800	2.96897800	0.38486100
H	-4.35831100	2.67265400	-1.34138700
H	-2.16551900	3.63134300	-0.41807800
H	-1.92142900	2.11713500	0.42873800
H	-2.04716900	2.54573700	-2.59523400
H	-2.04304300	0.94732200	-1.87780000
H	0.22213300	1.72656000	-2.63308600
H	0.16537300	3.01997200	-1.44509400
H	0.10899400	-0.00204300	-0.83133800
H	1.27221100	0.48418400	1.15346800
H	0.64097800	3.04132000	1.69202400
H	1.47259900	3.39032600	0.19478100
H	2.70526600	1.91478800	2.58233500
H	2.84747300	3.63495700	2.29573900
H	4.88593600	2.58341400	1.60443000
H	4.09144300	3.21285300	0.17623300
H	3.39344600	0.98397600	-0.51881700
H	3.82091100	0.30550600	1.04112900
H	-2.13365400	-2.95946000	-1.65850200
C	-1.68795400	-2.63572900	-0.72805100
C	-0.31630500	-2.69034300	-0.57022300
N	0.24137100	-2.29118200	0.58611900
C	-0.48496600	-1.81740200	1.61290800
C	-1.86006900	-1.73621800	1.50373800
C	-2.46908500	-2.15262800	0.32135600

H	-3.54462200	-2.09159100	0.21730400
H	-2.44134500	-1.34843500	2.32856500
H	0.06556000	-1.51526600	2.49275000
H	1.28951800	-2.31938500	0.66531500
H	0.36875300	-3.02985500	-1.33470400

11Z_pyr
conf_0

C	-1.91248600	-1.51168400	-0.93944400
C	-3.29144200	-1.66269600	-0.28387000
C	-3.29953100	-2.12565500	1.17949300
C	-2.76023000	-1.08504900	2.18017700
C	-1.27929000	-0.82626700	2.14560800
O	-0.78046600	0.28367100	2.15765000
O	-0.45135900	-1.88844100	2.14888700
H	-0.94707600	-2.71651300	2.08049100
H	-3.25328100	-0.12307000	2.04005800
H	-2.98213900	-1.41310900	3.20352100
H	-2.77319900	-3.08360300	1.28487500
H	-4.32914700	-2.33982700	1.47522500
H	-3.82300300	-0.70771700	-0.34215900
H	-3.88210300	-2.37462800	-0.86920100
C	-1.17497400	-2.83187000	-1.18077500
C	0.19333200	-2.66602600	-1.85420100
C	1.28568000	-2.09070000	-0.94358000
C	2.65488400	-2.00054700	-1.63174900
C	2.78827000	-0.80304000	-2.59551600
C	3.02907400	0.49746900	-1.88069500
C	2.19827500	1.53504100	-1.75200700
C	0.78873000	1.65605900	-2.25183800
C	-0.20497300	1.95015000	-1.11810400
C	-1.64679000	2.11434600	-1.59939200
C	-2.64906400	2.26246300	-0.45370700
C	-4.10681400	2.30011100	-0.91633100
C	-5.09928300	2.38291200	0.24424300
H	-6.13154700	2.41532600	-0.11161700
H	-4.92718400	3.27839000	0.84840100
H	-5.00608000	1.51604700	0.90767900
H	-4.25328000	3.15390900	-1.58688400
H	-4.31690800	1.40688300	-1.51748500
H	-2.51260200	1.43233200	0.24910600
H	-2.42473900	3.17272800	0.11627400
H	-1.92111100	1.24491800	-2.20768700
H	-1.71387900	2.98167900	-2.26605900
H	-0.16184500	1.13510300	-0.38696400
H	0.10614800	2.85839500	-0.58539900
H	0.73492400	2.46927400	-2.98634000
H	0.47976800	0.74705700	-2.77354500
H	2.57108400	2.41513600	-1.22953300
H	4.02042200	0.59224500	-1.43993000
H	1.90674200	-0.74277500	-3.23677000
H	3.63712500	-0.98459800	-3.26296000
H	3.44977200	-1.93314500	-0.88016100

H	2.83732500	-2.93027700	-2.18008900
H	0.99555300	-1.09786700	-0.58190800
H	1.36427200	-2.73491600	-0.05946500
H	0.53038500	-3.64351600	-2.21457000
H	0.07781000	-2.03853600	-2.74513200
H	-1.80735500	-3.46870000	-1.80833500
H	-1.03939300	-3.38338700	-0.24159200
H	-2.05184600	-1.02059900	-1.90730900
H	-1.28840600	-0.82418200	-0.36140300
H	4.67320600	-1.10217800	1.60822200
C	4.03022100	-0.23391400	1.64666100
C	4.54620500	1.05675000	1.54261600
C	3.68835500	2.15403600	1.58686100
C	2.33230200	1.93481800	1.73899500
N	1.86713800	0.68006000	1.85044800
C	2.66722400	-0.39760400	1.80264600
H	2.17490900	-1.35578100	1.89005200
H	0.83607200	0.52579900	1.97540900
H	1.59557600	2.72485300	1.77734300
H	4.06146800	3.16524600	1.50120500
H	5.61214900	1.20672900	1.42266600

conf_116

C	-4.49369200	-0.89444200	-0.25157200
C	-3.82056100	-2.24854500	-0.48670200
C	-3.17729300	-2.40018700	-1.87005800
C	-2.04095400	-1.38719900	-2.15208100
C	-0.95878200	-1.49697500	-1.11443300
O	-0.26442800	-2.49474200	-0.98313600
O	-0.75513000	-0.48111400	-0.26830700
H	-1.20540400	0.35379100	-0.53242100
H	-1.58605300	-1.61908800	-3.11808400
H	-2.42490900	-0.36728800	-2.19631200
H	-3.92953300	-2.27214000	-2.65353500
H	-2.77013200	-3.40783500	-1.97961900
H	-3.05891200	-2.41227200	0.28701100
H	-4.55186800	-3.05119200	-0.35246000
C	-4.91522100	-0.67529300	1.20355800
C	-5.47179000	0.72869800	1.49174700
C	-4.58522700	1.88173700	0.99546000
C	-3.14520600	1.84323800	1.52111000
C	-2.19988800	2.84260600	0.82617700
C	-1.91814800	2.50427900	-0.61209800
C	-0.72031400	2.41752700	-1.21161200
C	0.63466400	2.62543200	-0.59104900
C	1.70527700	1.66570400	-1.13040200
C	3.06980100	1.85214400	-0.46548000
C	4.10837900	0.83025300	-0.93618400
C	5.46428800	0.93372400	-0.22675800
C	6.22924200	2.22488900	-0.52917600
H	5.69238700	3.10831700	-0.17561800
H	6.38874900	2.34000300	-1.60523000
H	7.20940300	2.22305200	-0.04704900

H	5.31219200	0.84898700	0.85707400
H	6.08107400	0.07633300	-0.51806700
H	3.70145300	-0.17886500	-0.79188700
H	4.25926300	0.93774100	-2.01690700
H	2.95690200	1.77473200	0.62467600
H	3.42878200	2.86793400	-0.65700500
H	1.37147800	0.63310900	-0.98542500
H	1.80510300	1.80401000	-2.21315300
H	0.95461700	3.65603500	-0.79301000
H	0.57756600	2.54035200	0.49814700
H	-0.71002900	2.21990800	-2.28314700
H	-2.79418000	2.37101500	-1.24290100
H	-1.26738400	2.92050400	1.38937200
H	-2.65616400	3.84053200	0.85640300
H	-3.15209700	2.05159300	2.59601900
H	-2.72799400	0.83634100	1.41422700
H	-4.58431600	1.88067500	-0.09869000
H	-5.04164900	2.83348000	1.28647900
H	-6.45874500	0.82953700	1.02805900
H	-5.63118300	0.82737500	2.57087200
H	-4.05139200	-0.86731500	1.85050900
H	-5.66604300	-1.42112300	1.48404900
H	-3.80615500	-0.09452500	-0.54064900
H	-5.36192400	-0.79312900	-0.91367400
H	3.06709000	-0.08834500	2.88104000
C	3.06143000	-0.89675100	2.16325200
C	4.18976800	-1.68725000	1.95318700
C	4.15148400	-2.71723800	1.01434200
C	2.98206100	-2.93476300	0.30976200
N	1.90994600	-2.15618600	0.53472500
C	1.91931100	-1.15225000	1.42793700
H	1.00255400	-0.58462400	1.50507900
H	1.02725400	-2.32152200	-0.02676400
H	2.86500500	-3.71010100	-0.43515200
H	5.01362200	-3.34277000	0.82850400
H	5.09663200	-1.49909500	2.51413400

conf_38

C	1.98946300	2.23922500	0.35083900
C	3.11042000	2.28296300	1.39535300
C	2.93578800	1.37448900	2.62110100
C	2.94082700	-0.12767800	2.29947400
C	1.59586400	-0.72558300	1.98523900
O	0.52944200	-0.25536000	2.33168000
O	1.57503800	-1.90618100	1.33400400
H	2.45193100	-2.14541800	1.00281100
H	3.63984800	-0.35559000	1.48704600
H	3.29585200	-0.69631500	3.16923100
H	2.01844500	1.61691000	3.16189200
H	3.76085500	1.56760200	3.31036000
H	4.06125400	2.03309700	0.90999400
H	3.21764900	3.31232600	1.75253000
C	0.62608400	2.72558900	0.84899000

C	-0.41210400	2.82449700	-0.27371800
C	-1.84660400	2.93103200	0.24819900
C	-2.90208800	3.20699400	-0.82889700
C	-2.90050300	2.21090100	-2.00444800
C	-3.12343400	0.78646400	-1.58448900
C	-2.35151500	-0.27376600	-1.84178100
C	-1.05881700	-0.29821900	-2.60592100
C	0.12054700	-0.84920100	-1.78835300
C	1.44458000	-0.78390800	-2.55334100
C	2.64858200	-1.28737400	-1.75604500
C	3.97203000	-1.23461100	-2.52125700
C	5.16050700	-1.69356200	-1.67511300
H	5.02809400	-2.72498900	-1.33190600
H	5.28569800	-1.05724500	-0.79187900
H	6.09493300	-1.65409400	-2.23878400
H	3.89547800	-1.85682300	-3.41926600
H	4.14300700	-0.21081200	-2.87175300
H	2.74794800	-0.67919300	-0.84770200
H	2.46197300	-2.32095700	-1.43151500
H	1.62893400	0.25297000	-2.85820200
H	1.35649700	-1.36201700	-3.48036100
H	0.20658600	-0.27909800	-0.85635000
H	-0.08584500	-1.88757800	-1.49868800
H	-1.18365100	-0.92095800	-3.50071700
H	-0.80536700	0.70067700	-2.96659600
H	-2.69129300	-1.24526200	-1.48632100
H	-4.04337200	0.61672500	-1.02558400
H	-1.97252100	2.30815200	-2.56953400
H	-3.70460500	2.49813300	-2.69234700
H	-3.89448300	3.21458400	-0.36356000
H	-2.74892100	4.21288600	-1.23324300
H	-2.09156900	1.99481200	0.76626200
H	-1.90194400	3.72076400	1.00554400
H	-0.18343500	3.68713200	-0.91073600
H	-0.32793600	1.94290900	-0.91613000
H	0.73980400	3.70484400	1.32850000
H	0.25591800	2.04749400	1.62304100
H	2.29757400	2.85163600	-0.50380000
H	1.88277200	1.22228100	-0.04869000
H	-5.05581900	-0.85314500	1.34293500
C	-4.11569300	-1.37850900	1.24795700
C	-4.05516500	-2.67335600	0.73739000
C	-2.82361900	-3.31834300	0.62113000
C	-1.68171800	-2.65469500	1.02696100
N	-1.77421000	-1.40906700	1.52327400
C	-2.94263800	-0.75750000	1.63518500
H	-2.89829100	0.24609800	2.03363400
H	-0.88824300	-0.93680200	1.83026300
H	-0.68385600	-3.06755100	0.97511700
H	-2.74858400	-4.32063500	0.22246600
H	-4.96244900	-3.17684100	0.42692500

conf_1

C	-1.92035400	-1.71931400	-0.99191200
C	-3.31011100	-1.90551400	-0.36860400
C	-3.34151700	-2.23706200	1.12961600
C	-2.86255300	-1.09310100	2.04504400
C	-1.38485300	-0.81488000	2.05335000
O	-0.89895400	0.29992700	1.99246500
O	-0.54597300	-1.85850500	2.18936000
H	-1.02848100	-2.69701900	2.17085000
H	-3.36048200	-0.15843800	1.78956100
H	-3.12516100	-1.32441200	3.08515900
H	-2.78703500	-3.16311500	1.33203500
H	-4.37109300	-2.46140500	1.41776800
H	-3.90166500	-0.99895600	-0.52702100
H	-3.83171100	-2.70462100	-0.90511000
C	-1.11652300	-3.01790500	-1.11300100
C	0.27934200	-2.83960200	-1.72111700
C	1.29959900	-2.18283400	-0.78316000
C	2.69818500	-2.07071800	-1.40401500
C	2.82788500	-0.92454100	-2.42738000
C	2.93308700	0.42659800	-1.77791100
C	2.03056900	1.41049400	-1.77685400
C	0.66550500	1.41971500	-2.40234100
C	-0.40757500	1.99463800	-1.46556000
C	-1.76529300	2.17313000	-2.15137500
C	-2.81244600	2.89845500	-1.29850500
C	-3.26235300	2.12490700	-0.05641800
C	-4.33564000	2.85954400	0.74852200
H	-4.64673400	2.28699800	1.62728200
H	-5.22651600	3.04576900	0.14216200
H	-3.96852000	3.82805800	1.10039300
H	-3.64898900	1.15028200	-0.37780500
H	-2.40296900	1.91686300	0.58797700
H	-2.41595000	3.87532900	-0.99351400
H	-3.69006500	3.11017500	-1.91904600
H	-2.15326600	1.19190400	-2.45187700
H	-1.61292300	2.73559400	-3.07903500
H	-0.50818900	1.34799800	-0.58697800
H	-0.06976100	2.97176800	-1.09451800
H	0.69753400	2.03885600	-3.30847800
H	0.37443200	0.41730900	-2.72625900
H	2.30994700	2.34669200	-1.29474500
H	3.88008900	0.61421800	-1.27409100
H	1.99397200	-0.95722500	-3.13121800
H	3.73300500	-1.08648300	-3.02214000
H	3.44826400	-1.92337000	-0.61848400
H	2.94977500	-3.02034100	-1.88712500
H	0.95417500	-1.18665600	-0.48414800
H	1.35418300	-2.78265700	0.13321200
H	0.66660900	-3.82162900	-2.01205800
H	0.19308200	-2.26192700	-2.64820900
H	-1.68976500	-3.71676900	-1.73114700
H	-1.01338500	-3.50494500	-0.13493800
H	-2.05037000	-1.30298600	-1.99616000
H	-1.35244700	-0.96071200	-0.44416500
H	4.62514100	-0.95555800	1.92252400

C	3.95150300	-0.11417800	1.83775000
C	4.42881200	1.18180900	1.64719400
C	3.53298700	2.24297700	1.53240500
C	2.17786500	1.98336600	1.61512700
N	1.74973800	0.72656100	1.81456000
C	2.58740700	-0.31773500	1.91968500
H	2.12364000	-1.28338200	2.06386200
H	0.71748000	0.54567500	1.88567000
H	1.41324600	2.74282200	1.53033100
H	3.87625200	3.25630000	1.37610900
H	5.49474200	1.36320200	1.58362400

conf_2

C	-2.33802600	0.74441500	1.51562500
C	-3.76792500	0.97413600	1.00946400
C	-3.92057200	1.95386700	-0.16181300
C	-3.29141500	1.46397700	-1.48168500
C	-1.79018000	1.50652700	-1.56060400
O	-1.10112500	0.59023400	-1.97092500
O	-1.17176600	2.64655600	-1.20376400
H	-1.80218500	3.28626900	-0.84378800
H	-3.59296100	0.43875300	-1.69545500
H	-3.65063200	2.08627200	-2.31096800
H	-3.53931500	2.94637200	0.11259600
H	-4.98469600	2.10699800	-0.35548400
H	-4.20000200	0.01560200	0.70486900
H	-4.38439600	1.33497200	1.83912400
C	-1.73018100	1.94384600	2.24850800
C	-0.28333100	1.72945300	2.71038800
C	0.75099900	1.74688900	1.57712300
C	2.19917500	1.69720000	2.08312000
C	2.66612300	0.29698900	2.52110600
C	2.95372100	-0.60287400	1.35336900
C	2.52349700	-1.84821200	1.13831500
C	1.63051100	-2.68639000	2.00775100
C	0.56507500	-3.45286900	1.20947600
C	-0.49512300	-2.55099700	0.57671700
C	-1.53520800	-3.32101600	-0.23926800
C	-2.64792600	-2.43529600	-0.80410700
C	-3.67539500	-3.21175400	-1.62905700
H	-4.46289700	-2.55729800	-2.01318800
H	-4.15606600	-3.99008100	-1.02970100
H	-3.20409100	-3.70041400	-2.48691100
H	-3.15274500	-1.93195400	0.02883200
H	-2.20102500	-1.64500400	-1.41667400
H	-1.03640000	-3.84758300	-1.06316200
H	-1.97988000	-4.10188400	0.38926100
H	-0.01068500	-1.79852100	-0.05973100
H	-0.99930200	-1.98705400	1.37005500
H	1.05740400	-4.04789800	0.42967900
H	0.07259900	-4.17193200	1.87172500
H	2.25559300	-3.41255700	2.54228200
H	1.14864300	-2.07536500	2.77612600

H	2.88970200	-2.35973600	0.24786000
H	3.64059800	-0.17673300	0.62395900
H	1.93633300	-0.15307300	3.19730800
H	3.58958200	0.40574100	3.10269600
H	2.87939600	2.05737700	1.30186400
H	2.30636700	2.39585400	2.91898800
H	0.57546000	0.90995900	0.89124900
H	0.60310200	2.66436300	0.99571200
H	-0.02197300	2.51953700	3.42228000
H	-0.21854600	0.78670600	3.26522800
H	-2.35562700	2.16729700	3.11921400
H	-1.76552100	2.84257600	1.61983500
H	-2.35480000	-0.10440000	2.20695200
H	-1.69011600	0.42435800	0.69451400
H	4.16995800	2.63194500	-1.61644100
C	3.63081000	1.70955300	-1.78243300
C	4.29878600	0.51882200	-2.06773800
C	3.57445000	-0.65526800	-2.26410400
C	2.19523700	-0.61038200	-2.17936900
N	1.57995400	0.55500300	-1.92179700
C	2.25073100	1.70043600	-1.71540600
H	1.64623800	2.57146400	-1.50320400
H	0.52929100	0.58298300	-1.89995200
H	1.55561500	-1.47136500	-2.31408600
H	4.06842100	-1.59341100	-2.47581000
H	5.38000600	0.50520800	-2.12983100

conf_4

C	2.78176700	1.42525200	-1.23439200
C	4.27734800	1.48446700	-0.89875200
C	4.68128700	0.99669700	0.49978400
C	4.40921200	-0.49624700	0.74353600
C	2.99070200	-0.81295500	1.13219500
O	2.28628200	-0.07867500	1.79823500
O	2.47995900	-2.00560600	0.76718800
H	3.07921200	-2.49292100	0.18505300
H	4.70476200	-1.09350600	-0.12557000
H	5.01724500	-0.85198900	1.58504700
H	4.18222400	1.57681900	1.27911900
H	5.75342400	1.16116000	0.62896300
H	4.83406400	0.90441600	-1.64412100
H	4.62005800	2.51859600	-1.00695400
C	1.89721800	2.33922700	-0.38277300
C	0.44654800	2.39970700	-0.87123500
C	-0.47245600	3.15938300	0.09347200
C	-1.78352900	3.65125500	-0.53087600
C	-2.68509100	2.55587900	-1.11437800
C	-3.22494900	1.59713500	-0.08200200
C	-3.77387500	0.40465800	-0.32229600
C	-3.94849900	-0.25609700	-1.66069200
C	-3.06817600	-1.50771800	-1.83581900
C	-1.57696900	-1.18128400	-1.90500000
C	-0.67565200	-2.40841000	-2.04219300

C	0.80428100	-2.03974400	-2.16322700
C	1.73700900	-3.25070500	-2.19860800
H	1.51524700	-3.89871100	-3.05036200
H	1.63093400	-3.86342200	-1.29627300
H	2.78581200	-2.94940700	-2.29426600
H	0.95006700	-1.43826900	-3.06670800
H	1.07474900	-1.38890700	-1.32500200
H	-0.82041000	-3.07292200	-1.17828800
H	-0.97941800	-2.99494600	-2.91666500
H	-1.28885000	-0.61132900	-1.01309400
H	-1.39932000	-0.51130700	-2.75472700
H	-3.25856000	-2.20557200	-1.00978400
H	-3.36829100	-2.03080600	-2.74947500
H	-4.99912300	-0.54642600	-1.77118500
H	-3.73733200	0.44801600	-2.46948900
H	-4.16332600	-0.15970900	0.52254900
H	-3.20764800	1.95262000	0.94754700
H	-2.15027200	2.00904700	-1.89610900
H	-3.52937900	3.04201600	-1.61835700
H	-2.34978300	4.21441800	0.21971000
H	-1.54255900	4.36425400	-1.32623200
H	-0.68750700	2.52213900	0.96178300
H	0.06523400	4.02912300	0.48690800
H	0.42202100	2.88311000	-1.85528000
H	0.06587500	1.38355500	-1.02731400
H	2.31563100	3.35279300	-0.39135000
H	1.91782100	2.01188500	0.66062700
H	2.66076300	1.69682900	-2.28881100
H	2.41430000	0.39278200	-1.16525800
H	-3.04246600	0.48527500	3.73670800
C	-2.41927600	-0.16612000	3.13986200
C	-2.90924900	-1.36147900	2.61660700
C	-2.08355900	-2.17277200	1.84069600
C	-0.77875400	-1.77800200	1.61829600
N	-0.33122200	-0.62559600	2.14595600
C	-1.10767100	0.18513800	2.88500500
H	-0.64466800	1.08965500	3.25424500
H	0.67821500	-0.37914000	1.99868200
H	-0.06049300	-2.34321400	1.04107800
H	-2.44257000	-3.09897200	1.41459900
H	-3.93305500	-1.65720800	2.80972000

conf_9

C	-2.21063500	-1.53225500	-1.39487900
C	-3.42658000	-0.72604000	-1.86857200
C	-4.68605800	-0.91398400	-1.00677100
C	-4.79111300	0.05934400	0.19328400
C	-3.60480800	0.00424600	1.11160300
O	-2.77627800	0.88961400	1.20506700
O	-3.42959000	-1.10563700	1.85717000
H	-4.12976300	-1.75319700	1.69675000
H	-4.86271700	1.08513800	-0.16715000
H	-5.69624400	-0.15945100	0.76992000

H	-4.74784600	-1.95327700	-0.66215800
H	-5.58216200	-0.74704700	-1.60826800
H	-3.17080900	0.33902600	-1.91285300
H	-3.65687000	-1.02317700	-2.89562800
C	-0.94668400	-1.22942000	-2.20330300
C	0.23689500	-2.15597400	-1.90220100
C	0.69822500	-2.16260000	-0.44002500
C	1.92525500	-3.05307300	-0.20092800
C	3.24944500	-2.46152200	-0.72223000
C	3.76327400	-1.34738800	0.14356600
C	4.08768700	-0.10040600	-0.20519700
C	4.02895100	0.54011200	-1.56266300
C	3.19558600	1.83421300	-1.57144700
C	1.69336900	1.58103700	-1.44223800
C	0.86498900	2.85427900	-1.26639000
C	-0.64533800	2.60191700	-1.22929500
C	-1.46114500	3.84142900	-0.86209200
H	-2.53217300	3.62672300	-0.84986900
H	-1.28890200	4.65251100	-1.57464400
H	-1.19040500	4.21930400	0.13003400
H	-0.96610000	2.22921500	-2.20817300
H	-0.87139800	1.79424200	-0.52299200
H	1.17808300	3.36503000	-0.34444700
H	1.09180500	3.55795500	-2.07528300
H	1.51322300	0.90904700	-0.59530200
H	1.34723000	1.03938500	-2.33016800
H	3.53395600	2.48470700	-0.75424200
H	3.39121200	2.38539800	-2.49650000
H	5.05292300	0.78085700	-1.87155100
H	3.63747200	-0.15423200	-2.31067100
H	4.47661200	0.55291200	0.57632800
H	3.91165800	-1.63033700	1.18569300
H	3.13817100	-2.13684800	-1.75845300
H	4.00131200	-3.25930200	-0.73344600
H	2.03377200	-3.25483200	0.87164900
H	1.75388000	-4.02567600	-0.67360300
H	0.92193700	-1.13766900	-0.12149600
H	-0.12223700	-2.51741900	0.19365100
H	-0.02500100	-3.18123000	-2.19055300
H	1.07340700	-1.86618000	-2.54563200
H	-0.65328000	-0.19079000	-2.02413100
H	-1.18238800	-1.29951300	-3.27096500
H	-2.01210400	-1.33800300	-0.33732600
H	-2.44597500	-2.60180700	-1.46620000
H	1.68610000	-1.79299400	3.44458800
C	1.38608300	-0.83017300	3.05558400
C	2.30303000	0.20813000	2.90050300
C	1.88562800	1.43634200	2.39142600
C	0.56008800	1.59648600	2.04020700
N	-0.29991200	0.57487000	2.19545600
C	0.07216100	-0.61990500	2.68621600
H	-0.70601600	-1.36723000	2.75795100
H	-1.29515600	0.70154900	1.89099900
H	0.15331000	2.50753900	1.62577500
H	2.57805500	2.25472400	2.25368100

H	3.33992400	0.06024800	3.17365600

conf_15

C	2.38259500	-1.07251300	1.21896100
C	3.85092300	-0.76371100	0.89134500
C	4.29005000	-1.02690200	-0.55612900
C	3.71323900	-0.03786000	-1.58932500
C	2.23863200	-0.14962600	-1.86015700
O	1.47015900	0.79399100	-1.85578100
O	1.74381200	-1.36345300	-2.16765800
H	2.42347200	-2.04740600	-2.08865300
H	3.90187600	0.99046000	-1.28354000
H	4.21248500	-0.18743600	-2.55474700
H	4.07367000	-2.06415800	-0.84248700
H	5.37742700	-0.94054200	-0.61589000
H	4.06525100	0.28304300	1.12852600
H	4.48774800	-1.35978900	1.55277400
C	2.04927200	-2.56756800	1.20558000
C	0.58234200	-2.89485700	1.50717900
C	-0.39348900	-2.52055000	0.38390800
C	-1.81520000	-3.04583900	0.62065000
C	-2.59947800	-2.26597900	1.69314400
C	-3.07535600	-0.92867000	1.20311700
C	-2.76765000	0.27664200	1.68598300
C	-1.86578000	0.60079000	2.84876900
C	-1.21390900	1.98335700	2.71559200
C	-0.20087300	2.07984600	1.57226500
C	0.21671200	3.52155200	1.26936100
C	1.26308800	3.66934500	0.15787200
C	2.63565800	3.10317100	0.52598900
H	3.35635800	3.26484400	-0.27980000
H	2.58577200	2.03010500	0.72036600
H	3.02934400	3.58658500	1.42492000
H	0.90958700	3.17552100	-0.75568500
H	1.36926300	4.73049100	-0.08824300
H	-0.67910900	4.09554100	1.00106400
H	0.60072500	3.98495300	2.18613100
H	-0.63066700	1.62696600	0.67017000
H	0.67486000	1.47174500	1.81988300
H	-2.00390600	2.73018700	2.56674700
H	-0.72172900	2.24892300	3.65615200
H	-2.45959600	0.58026400	3.77092300
H	-1.09485500	-0.16707400	2.96686400
H	-3.25604100	1.13952400	1.23387300
H	-3.78320100	-0.97595100	0.37742200
H	-1.99911000	-2.16125400	2.59902700
H	-3.47806100	-2.85556400	1.97900300
H	-2.38442500	-3.01598400	-0.31628600
H	-1.76002300	-4.10146900	0.90546900
H	-0.42368000	-1.43237300	0.25590200
H	-0.00988300	-2.93602100	-0.55551500
H	0.49623600	-3.97106200	1.69110700
H	0.28939800	-2.40315500	2.44139100

H	2.68618200	-3.06641300	1.94376900
H	2.31310800	-3.01385100	0.23831100
H	2.16682200	-0.68158600	2.21899600
H	1.71374700	-0.52347600	0.54928400
H	-3.48565000	-1.74628000	-2.78433400
C	-3.07776200	-0.78703700	-2.49741500
C	-3.90370900	0.30730100	-2.24584300
C	-3.34604800	1.52702700	-1.86642800
C	-1.97192000	1.62768300	-1.75765800
N	-1.20204100	0.55771800	-2.01708200
C	-1.71034200	-0.63402400	-2.37175300
H	-0.99143200	-1.42389700	-2.53959300
H	-0.15728100	0.64678200	-1.94761800
H	-1.45550300	2.53318900	-1.47093600
H	-3.96434300	2.38937700	-1.65881900
H	-4.97828200	0.20846500	-2.33886700

conf_105

C	-5.07739900	-1.18876200	-0.29105600
C	-4.16979600	-2.06884400	0.57958900
C	-3.45094100	-3.17210700	-0.20740500
C	-2.31198200	-2.63792800	-1.10840500
C	-1.22514100	-1.96724400	-0.31395100
O	-0.94699200	-0.78588500	-0.37178000
O	-0.51506600	-2.73839800	0.54012600
H	-0.83429500	-3.65135500	0.53852700
H	-1.86679100	-3.46121600	-1.67764300
H	-2.68722600	-1.90736800	-1.82277500
H	-4.15994600	-3.70015300	-0.85074500
H	-3.05560300	-3.92707100	0.48257100
H	-3.42516600	-1.45216700	1.09497500
H	-4.77075800	-2.52963500	1.36909500
C	-5.52224100	0.11644900	0.38545200
C	-4.38141600	1.09417000	0.70896800
C	-3.57015000	1.53413000	-0.51204900
C	-2.35399200	2.39021000	-0.15847700
C	-1.46685500	2.67976100	-1.37947000
C	-0.23591700	3.47923200	-1.06038900
C	1.03260400	3.09705700	-1.24428700
C	1.50448800	1.77216600	-1.78592900
C	2.87374300	1.34982600	-1.23535700
C	3.23067000	-0.10071700	-1.56032200
C	4.56959600	-0.54792100	-0.97211000
C	4.86811500	-2.03302500	-1.18935200
C	6.20644400	-2.47005600	-0.59199000
H	6.39182800	-3.53356900	-0.75925600
H	6.23574800	-2.29292900	0.48817300
H	7.03581100	-1.91442200	-1.03839900
H	4.05711900	-2.63159200	-0.75328700
H	4.85682400	-2.24924600	-2.26341700
H	5.37654300	0.05316100	-1.40697600
H	4.58446700	-0.33124900	0.10479500
H	3.24358400	-0.24057400	-2.64738600

H	2.43243100	-0.75920500	-1.18939000
H	3.64671500	2.01830100	-1.63001500
H	2.89535900	1.49028400	-0.14846800
H	0.76689400	0.99075900	-1.57698200
H	1.57020200	1.82454100	-2.88031100
H	1.81846400	3.81096900	-1.00355800
H	-0.41364200	4.47799000	-0.66532600
H	-2.06147400	3.23607900	-2.11480500
H	-1.20099500	1.73323400	-1.85552000
H	-2.66965300	3.32981600	0.30933200
H	-1.76861900	1.85157100	0.59781100
H	-3.20832700	0.65593000	-1.05407400
H	-4.21737900	2.08127600	-1.20759000
H	-4.80475300	1.97600000	1.20142600
H	-3.70299400	0.64347500	1.44377400
H	-6.06157500	-0.12103100	1.30911400
H	-6.24452100	0.61659200	-0.26830700
H	-4.57756300	-0.94912800	-1.23430300
H	-5.96388000	-1.76905600	-0.56648800
H	3.62376200	-1.02158600	2.81980400
C	2.87944700	-0.28977000	2.53834700
C	2.93872200	1.02470400	3.00297600
C	1.96768200	1.94342500	2.61217100
C	0.95471400	1.53123000	1.76510400
N	0.91922200	0.25490600	1.34499000
C	1.84344800	-0.65524100	1.70218200
H	1.70884300	-1.64915400	1.29958000
H	0.16686000	-0.06198400	0.69395800
H	0.18574100	2.18733900	1.38566100
H	1.99485200	2.97098200	2.94738700
H	3.74196600	1.33085000	3.66189600

conf_31

C	2.96433700	0.21289100	0.94643000
C	3.93116900	1.30525600	0.47092600
C	4.13693000	1.41408300	-1.04604500
C	2.90406900	1.92210100	-1.81865200
C	1.74749900	0.96829900	-1.93750300
O	0.58755000	1.27584800	-1.73297800
O	2.00243000	-0.28913600	-2.34221900
H	2.95408800	-0.44356000	-2.42312900
H	2.51555900	2.83545100	-1.36758800
H	3.19392000	2.16981300	-2.84760300
H	4.49423500	0.46061400	-1.45663000
H	4.94497100	2.12262000	-1.24215300
H	3.58266700	2.27702000	0.83726000
H	4.90792900	1.13868800	0.93627100
C	3.46967100	-1.21766800	0.73348200
C	2.49737600	-2.29569000	1.22778500
C	1.24367200	-2.46220900	0.36007300
C	0.26541500	-3.50893000	0.90933000
C	-0.55902400	-3.00973800	2.11387000
C	-1.63623600	-2.03421600	1.72662600

C	-1.66194600	-0.71720900	1.94722500
C	-0.59616400	0.11865400	2.60095800
C	-0.39662100	1.47809800	1.90693000
C	-1.51221300	2.48731400	2.20175100
C	-1.38286200	3.79854300	1.41391200
C	-1.72979500	3.68885400	-0.07868600
C	-3.22306800	3.46635500	-0.34163800
H	-3.43449200	3.37536600	-1.41153200
H	-3.81357900	4.30586400	0.03467200
H	-3.60296200	2.56955700	0.15987500
H	-1.42319400	4.61106700	-0.58187900
H	-1.13506000	2.89335300	-0.54132300
H	-2.03046200	4.55658500	1.86758500
H	-0.35826700	4.17302700	1.51966000
H	-1.49754700	2.70963600	3.27387400
H	-2.49240600	2.03678000	2.01080900
H	0.55467400	1.91019900	2.23553000
H	-0.29716200	1.32128400	0.82730100
H	-0.86592400	0.29973600	3.64980000
H	0.34839600	-0.42777900	2.61957000
H	-2.55521200	-0.17606900	1.64156300
H	-2.49762000	-2.47826200	1.23025300
H	0.10505900	-2.57852400	2.86552100
H	-1.03572500	-3.87195000	2.59150500
H	-0.42658500	-3.82611000	0.12089200
H	0.82457000	-4.40446700	1.19935700
H	0.72349100	-1.50359700	0.25819400
H	1.56341600	-2.75205400	-0.64810700
H	3.01888300	-3.25765700	1.26588200
H	2.21172300	-2.06679400	2.26046500
H	4.42472800	-1.32789100	1.25785100
H	3.68985900	-1.40139300	-0.32569400
H	2.79281900	0.35744000	2.01812500
H	1.98248400	0.34680100	0.48345000
H	-2.08280700	-3.71716000	-2.28909900
C	-2.25984300	-2.68605700	-2.01641100
C	-3.52358200	-2.24492800	-1.62546700
C	-3.71587500	-0.91071100	-1.27268200
C	-2.63917500	-0.04530400	-1.32269100
N	-1.43776700	-0.49823400	-1.71538900
C	-1.21815700	-1.77921100	-2.05309400
H	-0.20545900	-2.02789200	-2.33744100
H	-0.63244300	0.17334900	-1.74610900
H	-2.69836000	1.00214600	-1.06424300
H	-4.68340400	-0.54291900	-0.95993800
H	-4.35372700	-2.93985800	-1.59207300

conf_77

C	-4.48603200	0.64509100	1.19078000
C	-5.64896800	-0.33447400	0.98834800
C	-5.53665800	-1.27678600	-0.21766500
C	-4.42324600	-2.33130900	-0.09699900
C	-3.00040900	-1.88356600	-0.30269500

O	-2.05508000	-2.34591800	0.31204700
O	-2.74407300	-0.98837200	-1.27013300
H	-3.56469600	-0.64252400	-1.64941000
H	-4.46031100	-2.82659700	0.87411600
H	-4.57854800	-3.11380300	-0.85149400
H	-5.44410300	-0.70471300	-1.15119900
H	-6.47749700	-1.82182800	-0.32455600
H	-5.76276500	-0.94867200	1.88804500
H	-6.57823000	0.23593500	0.89152300
C	-4.39790800	1.74697600	0.12879000
C	-3.29212200	2.77555800	0.39350500
C	-1.86798400	2.22910000	0.25629500
C	-0.79952800	3.26994000	0.60184000
C	0.63818100	2.74540200	0.46960800
C	1.09149200	2.58821800	-0.95798700
C	2.33127000	2.76404000	-1.42707300
C	3.55953600	3.12914600	-0.63816400
C	4.43120400	1.90941700	-0.27378700
C	3.77935100	0.99052500	0.76067400
C	4.57315900	-0.28400100	1.04445600
C	3.84193700	-1.25741600	1.97134500
C	4.60454600	-2.56297700	2.19850800
H	4.05533000	-3.24275700	2.85482200
H	5.57806400	-2.37430100	2.65861400
H	4.78920200	-3.08404500	1.25292800
H	3.65456100	-0.76848000	2.93349200
H	2.85099200	-1.47621100	1.55159300
H	4.80193700	-0.79029600	0.09684100
H	5.54572700	-0.02685100	1.47936700
H	2.77566000	0.71840900	0.41839000
H	3.63374000	1.54483900	1.69519000
H	4.65638900	1.33942900	-1.18512200
H	5.39548800	2.26196400	0.10603500
H	4.15960800	3.82655300	-1.23008200
H	3.28687100	3.65388800	0.28177700
H	2.48813100	2.63361300	-2.49709500
H	0.31505600	2.33714300	-1.67963000
H	0.70492900	1.78013700	0.99271200
H	1.32309000	3.40935400	1.00187600
H	-0.92387000	4.15571400	-0.03095800
H	-0.95967700	3.60366800	1.63217700
H	-1.73227100	1.36193400	0.91394000
H	-1.72774100	1.86359900	-0.76904400
H	-3.41397400	3.61711000	-0.29643100
H	-3.42452400	3.18735400	1.40112200
H	-5.36308800	2.26303300	0.08730300
H	-4.24937800	1.31985700	-0.87185700
H	-4.61255600	1.12424600	2.16720200
H	-3.53828300	0.09999200	1.26202900
H	2.50823300	0.04253600	-2.15065100
C	2.17523200	-0.87379500	-1.68543100
C	2.99335000	-1.99773700	-1.59894900
C	2.53407500	-3.14492500	-0.95188700
C	1.25890500	-3.14587800	-0.42138000
N	0.48627200	-2.04972500	-0.53331700

C	0.90707700	-0.92617700	-1.13851700
H	0.21294200	-0.09795600	-1.16102200
H	-0.49556900	-2.08886300	-0.16242200
H	0.82116800	-3.99089100	0.09203500
H	3.15306000	-4.02627500	-0.85866500
H	3.98746100	-1.97908500	-2.02727600

conf_107

C	-1.42323300	-2.76895000	0.34487500
C	-2.41572800	-3.72130700	-0.33478600
C	-3.83834800	-3.18337800	-0.53895300
C	-3.93959700	-2.03574900	-1.56229400
C	-3.41251600	-0.69575500	-1.12800200
O	-2.71534900	0.02250100	-1.82052900
O	-3.78250300	-0.23521700	0.08245500
H	-4.28251900	-0.90401700	0.57124000
H	-3.42378500	-2.29575000	-2.48697200
H	-4.99300100	-1.86762900	-1.82022700
H	-4.28502900	-2.89668300	0.42251200
H	-4.47148700	-3.99316200	-0.90874400
H	-2.02042900	-4.01237100	-1.31395900
H	-2.47972900	-4.64331400	0.25192100
C	-1.70053200	-2.51940700	1.83135900
C	-0.70403500	-1.55775800	2.49174400
C	-0.90927800	-0.08472900	2.11727400
C	0.16268700	0.84450600	2.70181300
C	1.50343300	0.79258700	1.94017000
C	1.44644800	1.47494400	0.60118500
C	1.40498900	0.89870200	-0.60289700
C	1.40630000	-0.56946300	-0.91976000
C	2.72683000	-1.04008700	-1.55932400
C	3.91336600	-0.99011300	-0.59530800
C	5.23044500	-1.42487200	-1.24333000
C	6.42196600	-1.46431400	-0.27788300
C	6.80168700	-0.09907100	0.30255500
H	7.69641500	-0.17229500	0.92555900
H	6.00382300	0.31627200	0.92406200
H	7.01104700	0.62066800	-0.49542800
H	6.20009900	-2.16135900	0.53927900
H	7.28588100	-1.88105800	-0.80550800
H	5.09823500	-2.41871300	-1.68678500
H	5.46257400	-0.74759500	-2.07528700
H	3.70418000	-1.63964900	0.26530300
H	4.00974100	0.02445000	-0.19750900
H	2.59560300	-2.06393300	-1.92602300
H	2.94344600	-0.42407900	-2.44069700
H	1.21208000	-1.15890900	-0.02115000
H	0.58240200	-0.77980000	-1.61291100
H	1.42114900	1.55160900	-1.47521400
H	1.45643900	2.56333300	0.63999900
H	1.82877000	-0.24371800	1.83213700
H	2.26879400	1.28889900	2.54547200
H	-0.19812100	1.87941600	2.70296300

H	0.33232000	0.58242000	3.75124900
H	-0.92670300	0.02835500	1.02820200
H	-1.89812100	0.22269900	2.47889400
H	-0.78195600	-1.64965900	3.57987300
H	0.31359500	-1.87233400	2.23494800
H	-1.67115600	-3.48226800	2.35196600
H	-2.71673800	-2.13269300	1.98133400
H	-0.42229100	-3.20296800	0.25433600
H	-1.36904100	-1.82188500	-0.20097400
H	-1.82887600	4.16944000	1.92469200
C	-1.58389000	3.89267500	0.90868900
C	-0.76715300	4.69536300	0.11433700
C	-0.46630100	4.30601500	-1.19016500
C	-0.99195500	3.12147500	-1.66994600
N	-1.78239800	2.37378300	-0.88269100
C	-2.08508500	2.71865800	0.37873700
H	-2.71808300	2.02842800	0.91798100
H	-2.15904500	1.46593400	-1.25255300
H	-0.80821900	2.74240900	-2.66551600
H	0.16813700	4.90773700	-1.82618500
H	-0.36380100	5.61918700	0.51061200

conf_193

C	-3.59977400	1.15223900	1.07991800
C	-3.88856200	2.44042800	0.29530000
C	-2.82099500	2.83900500	-0.73622200
C	-1.50896400	3.35303900	-0.12327700
C	-0.62034600	2.28611600	0.45847700
O	-0.47525600	1.17459400	-0.00839800
O	0.10067700	2.60479600	1.55690400
H	-0.08956600	3.50544300	1.85137400
H	-0.90226800	3.83316700	-0.90151400
H	-1.71446600	4.12243000	0.62843900
H	-3.21799300	3.64171000	-1.36201200
H	-2.59225600	2.00861300	-1.40798900
H	-4.84152400	2.32276800	-0.23069900
H	-4.03277200	3.27370700	0.99280800
C	-3.67783000	-0.11511000	0.21607400
C	-2.86882500	-1.28549200	0.77913400
C	-3.08599100	-2.62152900	0.05231400
C	-2.95431900	-2.56648600	-1.47637700
C	-1.58756100	-2.10499800	-2.00035900
C	-0.46998400	-3.08157400	-1.73779300
C	0.83769800	-2.80263800	-1.77077300
C	1.43851500	-1.45008800	-2.05082400
C	2.87805700	-1.29488000	-1.54209200
C	3.50817200	0.06308400	-1.87479700
C	2.85185800	1.24971800	-1.16401700
C	3.47747000	2.60183800	-1.51139800
C	2.77640900	3.77303800	-0.82025100
H	1.72674300	3.83479700	-1.12808100
H	3.24731000	4.72719500	-1.06660200
H	2.80095800	3.66142600	0.26897000

H	4.53835200	2.59505600	-1.23807800
H	3.44365700	2.74566000	-2.59679300
H	1.78364200	1.28330400	-1.40103000
H	2.92198200	1.09722200	-0.07809400
H	3.46133600	0.22187100	-2.95871400
H	4.57262500	0.03757500	-1.61774500
H	2.91095900	-1.45635500	-0.45643400
H	3.48977800	-2.09088100	-1.97930200
H	1.43976200	-1.28070100	-3.13652500
H	0.79625400	-0.66321900	-1.64311200
H	1.54198200	-3.62105400	-1.63334300
H	-0.76826800	-4.11545800	-1.57424900
H	-1.33674200	-1.11632700	-1.59991600
H	-1.66676200	-1.96149000	-3.08586100
H	-3.16802200	-3.56136900	-1.88134300
H	-3.72790100	-1.90908500	-1.88252000
H	-2.38710300	-3.36536500	0.45527700
H	-4.08663500	-2.99381600	0.29575300
H	-1.81175500	-1.00176700	0.73838400
H	-3.10591100	-1.42133800	1.84069200
H	-3.31667100	0.10493000	-0.79170800
H	-4.72802000	-0.40675200	0.10267800
H	-2.61570700	1.21209500	1.55893000
H	-4.31389900	1.07228300	1.90512400
H	1.46081600	-3.84042700	2.09293300
C	1.59561800	-2.77088600	2.17652100
C	2.54417000	-2.22086900	3.03499500
C	2.69586700	-0.83502200	3.10712800
C	1.89293600	-0.03416000	2.31942500
N	0.98491800	-0.59708400	1.50133400
C	0.81500200	-1.92650500	1.40745000
H	0.08380600	-2.27717600	0.69359500
H	0.41307800	0.03859900	0.90106200
H	1.93123100	1.04562000	2.30871300
H	3.42559000	-0.38175900	3.76375500
H	3.16484700	-2.86627700	3.64446000

conf_21

C	-4.13300100	-1.34972300	0.35057100
C	-4.09676800	-2.88282700	0.31984900
C	-2.79238100	-3.52501800	-0.17399700
C	-1.55979100	-3.29262400	0.75431800
C	-0.54595900	-2.35926700	0.16022300
O	0.55941100	-2.69757000	-0.22403600
O	-0.88036400	-1.06395500	0.01464300
H	-1.78282300	-0.88246800	0.32138600
H	-1.88513400	-2.88969800	1.71951600
H	-1.03999200	-4.22859100	0.94887900
H	-2.56547300	-3.19482800	-1.19180900
H	-2.94637300	-4.60281200	-0.24440100
H	-4.31515100	-3.26653000	1.32209600
H	-4.90991000	-3.23386200	-0.32352900
C	-4.03784800	-0.68170500	-1.02823700

C	-4.15417200	0.84816200	-0.99932200
C	-3.00453400	1.56661300	-0.28489400
C	-3.11027300	3.09077200	-0.37210800
C	-2.04265600	3.83184000	0.44969200
C	-0.63983900	3.62542700	-0.04804000
C	0.42401200	3.20461300	0.64387700
C	0.47235100	2.78646700	2.08718100
C	0.89892700	1.31882900	2.28330000
C	2.34828100	1.02377100	1.88841700
C	2.71206800	-0.45764400	1.99462500
C	4.13355500	-0.77625200	1.52577000
C	4.43314600	-2.27585800	1.49880600
H	3.73291600	-2.81034900	0.84730500
H	4.33813200	-2.71333400	2.49624100
H	5.44732900	-2.47879700	1.14595300
H	4.28753400	-0.34927000	0.52611600
H	4.85324500	-0.26733900	2.17557600
H	2.58911500	-0.79268100	3.03121500
H	1.99802000	-1.05044100	1.40928200
H	3.02343000	1.61474300	2.51812100
H	2.53265500	1.36003300	0.86142400
H	0.75832000	1.04855000	3.33544900
H	0.22569400	0.67455600	1.70563400
H	1.18104500	3.43198900	2.62100000
H	-0.49796000	2.94144800	2.56146700
H	1.37919800	3.17885900	0.12393900
H	-0.48804400	3.90062300	-1.09220700
H	-2.13156700	3.54304200	1.49974300
H	-2.26213700	4.90552400	0.40801300
H	-3.04747600	3.39954900	-1.42239100
H	-4.09919200	3.40243100	-0.02058400
H	-2.97931500	1.28277400	0.77519400
H	-2.04695700	1.25033100	-0.71705900
H	-4.20991800	1.21268900	-2.03056600
H	-5.10256000	1.12908000	-0.52686400
H	-4.84081600	-1.08826500	-1.65162800
H	-3.10492900	-0.95928400	-1.53365200
H	-5.07005700	-1.03544300	0.82160800
H	-3.36349800	-0.96975700	1.04390200
H	1.83089500	2.46021800	-2.23567200
C	2.23829900	1.46111300	-2.17053000
C	3.47164600	1.13790600	-2.73224700
C	3.97005500	-0.16002400	-2.61580800
C	3.21676100	-1.10566800	-1.94725100
N	2.02644000	-0.76430400	-1.42318700
C	1.52469900	0.47943100	-1.50734600
H	0.56985700	0.64654800	-1.02886500
H	1.46493700	-1.50221300	-0.92736800
H	3.52671600	-2.13192500	-1.80680900
H	4.92785500	-0.43628600	-3.03458800
H	4.04546300	1.89351000	-3.25469800

conf_74

C	4.62284500	-0.48825000	-1.10295500
C	3.38317100	-0.99797600	-1.84861500
C	3.20733400	-2.52206200	-1.74697000
C	2.62221100	-3.00747700	-0.41501700
C	1.16569400	-2.69993600	-0.17736900
O	0.65695800	-2.66044300	0.92678800
O	0.36765500	-2.50178700	-1.24464700
H	0.88037600	-2.52812200	-2.06603000
H	2.70238400	-4.09871100	-0.33472300
H	3.16866700	-2.60332000	0.43852500
H	4.17864400	-3.00517000	-1.88262300
H	2.60518800	-2.90124400	-2.58646800
H	2.48402400	-0.48625900	-1.48421600
H	3.48059300	-0.72685400	-2.90471500
C	4.88255500	1.01254900	-1.29829200
C	3.93869600	1.94883500	-0.53117000
C	4.15222900	1.91630800	0.98622200
C	3.39162200	3.00536400	1.75078400
C	1.85839600	2.90870300	1.63988000
C	1.33317800	1.59923500	2.14956500
C	0.52359000	0.74605800	1.51842300
C	-0.09886600	0.92642800	0.16140100
C	-1.54065600	1.45784200	0.23670300
C	-2.25437900	1.47111500	-1.11512300
C	-3.70212900	1.95723400	-1.03479800
C	-4.46195500	1.84266800	-2.35809100
C	-5.91167200	2.31993100	-2.26119600
H	-6.42958500	2.22423000	-3.21817200
H	-5.96030800	3.37028200	-1.96073900
H	-6.47194500	1.73987100	-1.52041100
H	-3.93545800	2.41891800	-3.12692700
H	-4.43951500	0.79885300	-2.69865800
H	-4.23566200	1.38732600	-0.26202100
H	-3.71658400	2.99921900	-0.69472900
H	-2.23333400	0.46065100	-1.54656300
H	-1.69676800	2.10141600	-1.81755800
H	-2.11589100	0.85333300	0.94859000
H	-1.52279400	2.46704700	0.66044000
H	0.49614200	1.60771800	-0.45225900
H	-0.10477600	-0.03213000	-0.37062700
H	0.25030800	-0.16410500	2.04763200
H	1.67262000	1.32696200	3.14784600
H	1.54066500	3.08413900	0.60959800
H	1.42046000	3.71916200	2.23524100
H	3.67102600	2.95697300	2.80899400
H	3.70846000	3.99102400	1.39378600
H	3.86829500	0.93564700	1.38321200
H	5.22241500	2.03205100	1.19121800
H	4.10138900	2.97235200	-0.88745600
H	2.89674900	1.70917400	-0.77053800
H	4.83029400	1.23833100	-2.36952800
H	5.91079000	1.23324700	-0.99311500
H	4.54723800	-0.71738600	-0.03457600
H	5.49104300	-1.04514900	-1.47212100
H	-4.15483800	-1.02317000	3.46967800

C	-3.75597000	-1.26826500	2.49505800
C	-4.53986300	-1.17925500	1.34503900
C	-3.99210300	-1.49624900	0.10348900
C	-2.67113400	-1.89738700	0.03969000
N	-1.94138000	-1.97613700	1.16511800
C	-2.43980600	-1.67278500	2.37591100
H	-1.76050700	-1.76123500	3.21238900
H	-0.93321500	-2.26484400	1.08262700
H	-2.15537700	-2.15311200	-0.87524100
H	-4.57456500	-1.42368200	-0.80433600
H	-5.57181700	-0.85840400	1.41621500

conf_88

C	5.06748200	1.07097700	-0.89892200
C	6.30974400	0.17234400	-0.94432400
C	6.28472500	-1.06280500	-0.03443100
C	5.22991000	-2.11424000	-0.42575700
C	3.79511100	-1.80958300	-0.08956900
O	2.85637600	-2.05223500	-0.82541400
O	3.52514700	-1.30437800	1.12931800
H	4.34101400	-1.09950400	1.60773300
H	5.26761700	-2.32237200	-1.49549700
H	5.44812500	-3.05953600	0.08796600
H	6.17908200	-0.76616700	1.01808000
H	7.25848500	-1.55546100	-0.08507900
H	6.46699500	-0.16931700	-1.97299200
H	7.18973000	0.77004200	-0.68587500
C	4.88779700	1.84185500	0.41388700
C	3.67054400	2.77469200	0.41560600
C	2.31801800	2.05600900	0.45814600
C	1.13552800	3.02178000	0.34559700
C	-0.23866200	2.34436500	0.41259600
C	-0.53529300	1.44638600	-0.76323500
C	-1.74995300	1.00217000	-1.09494900
C	-3.01552800	1.29888100	-0.33289800
C	-4.23784000	0.53448400	-0.84687100
C	-5.49530500	0.76683900	-0.00935400
C	-6.71521000	-0.00546300	-0.51512900
C	-7.97432700	0.22532700	0.32413600
C	-9.18642800	-0.55315800	-0.18970800
H	-10.06938000	-0.36863700	0.42669200
H	-8.99569200	-1.63080800	-0.18338500
H	-9.43209500	-0.26651600	-1.21651800
H	-7.77115000	-0.05539100	1.36471300
H	-8.20470100	1.29680800	0.33760300
H	-6.91688000	0.27788700	-1.55541600
H	-6.48546400	-1.07943900	-0.53321400
H	-5.72486400	1.83889900	0.00825400
H	-5.29527200	0.48648100	1.03400600
H	-4.42992800	0.82292900	-1.88657700
H	-4.01464400	-0.53984600	-0.87451900
H	-2.86852900	1.08502900	0.73432800
H	-3.22366800	2.37617100	-0.37569500

H	-1.85562600	0.40063300	-1.99532400
H	0.30180400	1.18342200	-1.40533200
H	-1.01627200	3.11223100	0.48273500
H	-0.31224600	1.77815200	1.35296900
H	1.20446900	3.76422300	1.14727000
H	1.21353600	3.57936000	-0.59468900
H	2.26626400	1.31443000	-0.34613400
H	2.24147800	1.49536200	1.40002700
H	3.73578000	3.45075800	1.27443300
H	3.71132700	3.41211900	-0.47565100
H	5.79236300	2.43276000	0.59259100
H	4.80866700	1.15494800	1.26611600
H	5.14783700	1.80023800	-1.71173400
H	4.16972300	0.48696000	-1.12976300
H	-1.30097600	-0.57764900	2.70624400
C	-1.14661200	-1.10977100	1.77797700
C	-2.20099800	-1.73085100	1.11428700
C	-1.96878400	-2.39711100	-0.08813500
C	-0.68327900	-2.43831000	-0.59180500
N	0.31486300	-1.83622500	0.07596400
C	0.11884800	-1.17077400	1.22562700
H	0.99704000	-0.71775500	1.66361100
H	1.29107200	-1.88476000	-0.30418000
H	-0.41492900	-2.93647300	-1.51320100
H	-2.77090200	-2.88018800	-0.62832300
H	-3.20234800	-1.68781900	1.52412300

conf_195

C	4.25362100	1.09984100	-0.95382600
C	5.47540200	0.27422300	-1.37862000
C	5.71505400	-1.02847400	-0.60332400
C	4.67918400	-2.13347700	-0.88449700
C	3.30648800	-1.94620900	-0.29800000
O	2.27205300	-2.16810800	-0.90035100
O	3.21529200	-1.56790800	0.99035800
H	4.08692500	-1.36119700	1.35611700
H	4.54860400	-2.27674500	-1.95743900
H	5.04226700	-3.08608700	-0.47778300
H	5.79850300	-0.82851100	0.47342000
H	6.68995200	-1.43216900	-0.88624500
H	5.39059700	0.02543300	-2.44185900
H	6.37018500	0.89779100	-1.28458400
C	4.39484500	1.78213800	0.41240700
C	3.14316100	2.56555300	0.82950900
C	1.99965100	1.67258700	1.32518800
C	0.67106000	2.40992300	1.53181200
C	0.00006000	2.89466400	0.22917300
C	-0.25694100	1.78550500	-0.75379400
C	-1.42378900	1.18088500	-0.99621100
C	-2.75618200	1.46664200	-0.36933300
C	-3.85159000	1.74249300	-1.41102800
C	-5.25976900	1.86087100	-0.81155400
C	-5.74106400	0.63114400	-0.02656100

C	-5.71394500	-0.67847600	-0.81978300
C	-6.25593600	-1.86852500	-0.02585100
H	-6.21726800	-2.79521800	-0.60508300
H	-7.29736700	-1.70703500	0.26410200
H	-5.69053000	-2.02549700	0.90019800
H	-6.29914700	-0.55635700	-1.73799200
H	-4.68972100	-0.89094300	-1.14810800
H	-5.14416600	0.51300100	0.88708600
H	-6.76442100	0.81615700	0.31710300
H	-5.96583700	2.06056100	-1.62470400
H	-5.29961500	2.73618700	-0.15365200
H	-3.60809500	2.66534300	-1.94586800
H	-3.83482800	0.94766200	-2.16487400
H	-3.05457200	0.59578200	0.22426000
H	-2.69407600	2.30118400	0.33411100
H	-1.43823200	0.40303700	-1.75960200
H	0.60570600	1.45064900	-1.32572200
H	0.63523400	3.64801200	-0.24719900
H	-0.93078500	3.40363500	0.48699300
H	-0.03363400	1.75052200	2.05181200
H	0.83049000	3.26889300	2.19239300
H	1.84816500	0.84831500	0.62129500
H	2.30732700	1.21280500	2.27256300
H	3.39977900	3.27664700	1.62104800
H	2.80928700	3.16730100	-0.02247500
H	5.25301000	2.46050300	0.36906700
H	4.63500500	1.05053600	1.19498000
H	4.09228000	1.87909100	-1.70574300
H	3.34753700	0.48451100	-0.98431900
H	-1.51529600	-0.54642400	2.97947700
C	-1.45393600	-1.09847300	2.05200100
C	-2.58417500	-1.66748200	1.46985300
C	-2.47303000	-2.36166600	0.26515700
C	-1.22953500	-2.47469600	-0.32537500
N	-0.15480700	-1.92455100	0.26661100
C	-0.23322400	-1.23976700	1.41888800
H	0.69559000	-0.83363400	1.79306300
H	0.78528000	-2.01558400	-0.19006800
H	-1.05194800	-2.99347800	-1.25739200
H	-3.33851900	-2.80317500	-0.20839500
H	-3.55123200	-1.56686700	1.94631700

conf_191

C	-3.14489500	-0.00797300	0.50296700
C	-4.00524300	-1.16072100	-0.03318800
C	-3.95594800	-1.39828000	-1.54897100
C	-2.69619800	-2.13648400	-2.03231300
C	-1.38893300	-1.39976600	-1.93412200
O	-0.36034900	-1.91947700	-1.53623300
O	-1.33271300	-0.13294100	-2.36732100
H	-2.21378100	0.19683200	-2.59511800
H	-2.56926200	-3.07352600	-1.48966900
H	-2.80669400	-2.39555400	-3.09334900

H	-4.09729000	-0.45515500	-2.09281400
H	-4.80657300	-2.01851100	-1.84112400
H	-3.72605100	-2.09151000	0.47301700
H	-5.04515000	-0.96462800	0.24563800
C	-3.58332800	1.37659100	0.00348600
C	-3.07929500	2.53083800	0.87895100
C	-1.56245500	2.75938600	0.86624700
C	-1.03742800	3.43442000	-0.40377500
C	0.45512900	3.81344500	-0.30584700
C	1.40969900	2.67094600	-0.52456300
C	2.07044600	1.96638100	0.39635400
C	1.99113600	2.06903700	1.89363700
C	1.92332200	0.68016600	2.55569000
C	0.69130000	-0.13517300	2.15687900
C	0.68040700	-1.55860600	2.71514900
C	-0.50440600	-2.39343000	2.22031000
C	-0.51093200	-3.81884200	2.77413500
H	-1.36763000	-4.38863000	2.40582100
H	-0.55884300	-3.81698700	3.86658200
H	0.39553200	-4.36077800	2.48606000
H	-1.43542900	-1.88658000	2.49838300
H	-0.49338100	-2.42316500	1.12369400
H	1.61935200	-2.06774400	2.45260700
H	0.66984700	-1.52297400	3.81058400
H	0.61603400	-0.17004700	1.06277100
H	-0.21031500	0.38708400	2.49449400
H	2.83031200	0.11950700	2.29132800
H	1.94901700	0.79622100	3.64358800
H	2.88103800	2.58888800	2.26978200
H	1.13021300	2.66815800	2.19722400
H	2.76911800	1.21096900	0.04397500
H	1.59194700	2.42506200	-1.57023900
H	0.63677900	4.28426900	0.66424400
H	0.67349500	4.57626500	-1.06008800
H	-1.19437700	2.79158500	-1.27796200
H	-1.61686300	4.34685100	-0.58100000
H	-1.30681100	3.39908700	1.71832200
H	-1.03559500	1.81575700	1.02787200
H	-3.57824600	3.45711700	0.57380600
H	-3.40276900	2.33864000	1.90802400
H	-4.67842000	1.40993900	-0.01400600
H	-3.26453800	1.53914700	-1.03366300
H	-3.22460600	-0.02019300	1.59516900
H	-2.08394900	-0.17661300	0.29277700
H	4.53872200	0.69131700	-2.89079500
C	4.08225600	0.02253000	-2.17436100
C	4.82186500	-0.54736400	-1.13839900
C	4.19938200	-1.39688700	-0.22507900
C	2.85053000	-1.66208300	-0.37257900
N	2.16626300	-1.10207300	-1.38313800
C	2.73635000	-0.27547100	-2.27490100
H	2.08667800	0.12564500	-3.04030300
H	1.14164400	-1.32418100	-1.47139100
H	2.28605400	-2.30147900	0.29140700
H	4.74748900	-1.84688100	0.59110800

H	5.87737000	-0.32530500	-1.04060000

conf_138

C	4.41431700	1.16559200	-1.26895700
C	5.85391700	0.69518400	-1.02273900
C	6.09124100	-0.14454900	0.23907600
C	5.41104900	-1.52774900	0.21338900
C	3.92282200	-1.54720700	0.42658800
O	3.13950800	-2.18905400	-0.24759400
O	3.43127100	-0.85223000	1.47283400
H	4.12728800	-0.33682900	1.90434100
H	5.60520800	-2.03664900	-0.73096300
H	5.83058500	-2.15661100	1.00895000
H	5.80725100	0.42048900	1.13700400
H	7.16397200	-0.32030900	0.34721200
H	6.19132200	0.11104200	-1.88570400
H	6.50817100	1.57171200	-0.97631500
C	3.90148400	2.20544000	-0.26591800
C	2.51282400	2.75664400	-0.61401600
C	1.35342200	1.78240000	-0.37673900
C	0.02751800	2.31526200	-0.92877700
C	-1.20262900	1.45961000	-0.56851300
C	-1.62974100	1.61574200	0.86413900
C	-2.79516200	2.08976000	1.31522600
C	-3.97081800	2.56925800	0.51414200
C	-5.29608200	1.88625500	0.89997200
C	-5.32368600	0.36744800	0.69085100
C	-5.00973600	-0.07670300	-0.74110400
C	-5.33482400	-1.54768100	-1.01414400
C	-4.97031700	-1.98756900	-2.43339100
H	-5.18623900	-3.04618800	-2.59796500
H	-3.90813600	-1.81962900	-2.64517600
H	-5.53463500	-1.41640000	-3.17529500
H	-4.82145500	-2.18575500	-0.28223800
H	-6.40292300	-1.71784300	-0.84326500
H	-5.57754200	0.54379400	-1.44520000
H	-3.95211200	0.10790100	-0.96451700
H	-6.31546700	-0.00267400	0.97222400
H	-4.61687000	-0.10446800	1.38522000
H	-5.52030100	2.10603100	1.94946700
H	-6.10077900	2.34156400	0.31320300
H	-4.08522200	3.64602700	0.68873500
H	-3.79112200	2.45506000	-0.55629900
H	-2.92004900	2.16702700	2.39481500
H	-0.87925300	1.35565200	1.60739700
H	-0.96463800	0.40630800	-0.77328800
H	-2.02564300	1.71725900	-1.23652500
H	-0.13733900	3.33643300	-0.56939500
H	0.10750200	2.38131500	-2.01911100
H	1.56716200	0.81514100	-0.84892000
H	1.26916700	1.58940700	0.69979800
H	2.32706700	3.66282800	-0.02841600
H	2.51142600	3.07056000	-1.66481600

H	4.61630500	3.03471400	-0.23455800
H	3.87569400	1.79209700	0.74999100
H	4.37593700	1.61190000	-2.26823900
H	3.73603800	0.30682700	-1.31501100
H	-1.42340300	-1.08074400	2.83443400
C	-1.15989800	-1.48552600	1.86783900
C	-2.13889000	-1.89322600	0.96461000
C	-1.77058000	-2.40068600	-0.27992900
C	-0.42729100	-2.48703700	-0.59476600
N	0.49391200	-2.07831900	0.29460900
C	0.16794900	-1.58509000	1.50287000
H	0.99737000	-1.27690300	2.12404400
H	1.51324700	-2.13552400	0.05216200
H	-0.05363600	-2.86630400	-1.53599800
H	-2.51358000	-2.71822800	-0.99774900
H	-3.18531100	-1.80495000	1.22353700

conf_219

C	4.24379000	-2.19174500	0.57741100
C	5.75819900	-2.03203100	0.76425100
C	6.37285400	-0.71079500	0.28476700
C	5.89124700	0.53002200	1.06527900
C	4.52014100	1.04000600	0.71869800
O	3.65622100	1.31082200	1.53025900
O	4.24988300	1.26181800	-0.58551600
H	4.98298500	0.97191200	-1.14640400
H	5.90195900	0.34009200	2.13862000
H	6.57562000	1.36725700	0.87902400
H	6.21526000	-0.58483000	-0.79444100
H	7.45689800	-0.76483900	0.40774200
H	5.99974100	-2.15139200	1.82597200
H	6.26677200	-2.85043800	0.24439500
C	3.78144000	-2.26515300	-0.88315400
C	2.30310500	-2.64535300	-1.03615300
C	1.30572800	-1.56027300	-0.61438200
C	-0.13535500	-2.08070700	-0.58478900
C	-1.19432000	-0.99398000	-0.34423800
C	-1.46266200	-0.15349400	-1.56553400
C	-2.60002300	0.48764800	-1.84994700
C	-3.83933900	0.51670300	-1.00311400
C	-4.97248100	-0.33975600	-1.59995300
C	-6.28722900	-0.26336300	-0.81613000
C	-6.21025500	-0.82478100	0.60672200
C	-7.56344900	-0.84151900	1.32244700
C	-7.48072200	-1.39300300	2.74662000
H	-8.45993200	-1.39632500	3.23132100
H	-6.80664600	-0.79133300	3.36476600
H	-7.10500900	-2.42078900	2.75010200
H	-7.97135500	0.17591500	1.34485600
H	-8.27094500	-1.43980200	0.73723400
H	-5.80828800	-1.84589500	0.57009700
H	-5.50263500	-0.23905100	1.20642800
H	-7.05435100	-0.81392900	-1.37136800

H	-6.62958900	0.77872400	-0.77931800
H	-5.14609600	-0.02132600	-2.63341400
H	-4.63372500	-1.38061700	-1.65361200
H	-3.61686000	0.18205900	0.01240200
H	-4.20100600	1.55101900	-0.91946500
H	-2.66648700	1.01163900	-2.80204400
H	-0.65143700	-0.09307500	-2.28854400
H	-0.86235200	-0.35799000	0.49077700
H	-2.12350900	-1.45890400	-0.00606500
H	-0.36055200	-2.59457600	-1.52603800
H	-0.21367000	-2.83820800	0.20142800
H	1.55963500	-1.17668800	0.38171500
H	1.39123600	-0.70939600	-1.30291400
H	2.10484200	-2.91097300	-2.07979400
H	2.11232700	-3.55390600	-0.45244900
H	4.39285700	-3.01129100	-1.40234600
H	3.96005400	-1.31390500	-1.39865200
H	3.94594400	-3.12107700	1.07457800
H	3.71117000	-1.39819300	1.11175500
H	-0.03559600	3.36383000	-2.18880600
C	-0.00237500	3.00966500	-1.16804600
C	-1.15776000	2.93621200	-0.38954700
C	-1.08282300	2.47556300	0.92385300
C	0.14213400	2.07550500	1.42166300
N	1.23419000	2.13762800	0.64081800
C	1.19406300	2.58999000	-0.62715300
H	2.13554100	2.58351800	-1.15836200
H	2.15909700	1.81957300	1.01663200
H	0.28857200	1.70024700	2.42524300
H	-1.96258700	2.41217800	1.54880000
H	-2.10950400	3.24409800	-0.80095100

conf_174

C	-4.64484600	0.80869700	0.86378500
C	-5.56203600	-0.25863400	0.25134200
C	-4.86877400	-1.36862200	-0.55305700
C	-3.97914300	-2.32244900	0.29893400
C	-2.50814300	-2.20206900	0.02185800
O	-1.78638000	-3.12795600	-0.30126900
O	-1.94441300	-0.98375800	0.12406700
H	-2.58364200	-0.30939300	0.40063800
H	-4.12952000	-2.12266600	1.36669400
H	-4.24062200	-3.36550200	0.13178700
H	-4.28738000	-0.93762300	-1.37385800
H	-5.63674800	-1.97821000	-1.03149400
H	-6.14760400	-0.72669500	1.04961100
H	-6.28452500	0.23845300	-0.40360800
C	-3.98443400	1.75091200	-0.15572200
C	-3.12541000	2.85290200	0.48536500
C	-1.80480500	2.36379000	1.10226800
C	-0.71530100	2.01020600	0.07683100
C	0.01589200	3.23977800	-0.48854000
C	0.99905500	2.87755500	-1.56548200

C	2.33274200	2.93679800	-1.49928200
C	3.18585400	3.37242500	-0.34042500
C	3.97858400	2.20641100	0.28349200
C	3.09754900	1.22157800	1.05386500
C	3.83551800	-0.03434400	1.51777100
C	2.91700600	-1.07962200	2.15458000
C	3.65223600	-2.35371200	2.57273000
H	4.14719000	-2.82351900	1.71636800
H	2.97136000	-3.08773000	3.01144900
H	4.42482600	-2.13567700	3.31471200
H	2.41797600	-0.64026500	3.02541700
H	2.11504000	-1.33033000	1.44808600
H	4.35583800	-0.48641500	0.66274900
H	4.62333300	0.24015400	2.22851100
H	2.25128100	0.92959900	0.42359100
H	2.65892900	1.72959300	1.92114500
H	4.51846900	1.67393500	-0.51081600
H	4.74637400	2.60983600	0.95098300
H	3.89420500	4.12734600	-0.69757300
H	2.58416000	3.85364500	0.43447500
H	2.89218500	2.64541000	-2.38844100
H	0.55435800	2.53846000	-2.50073700
H	0.51038300	3.76319900	0.33266000
H	-0.71852700	3.93870800	-0.90450100
H	0.02473300	1.35486300	0.54579100
H	-1.14978500	1.44152500	-0.75445300
H	-1.41325300	3.13677800	1.77114800
H	-1.99585700	1.50086600	1.75102900
H	-2.90998700	3.61809200	-0.26660900
H	-3.72064300	3.35129100	1.25730500
H	-4.78192400	2.21760200	-0.74218200
H	-3.38690600	1.19098900	-0.88682100
H	-5.23744100	1.41969100	1.55164900
H	-3.89796300	0.33314300	1.51981500
H	2.42953200	0.22664300	-1.93485600
C	2.26690600	-0.80369400	-1.65033300
C	3.30080900	-1.73710100	-1.62783500
C	3.04794300	-3.04718800	-1.22111700
C	1.76198400	-3.39723000	-0.85671100
N	0.78326800	-2.47552600	-0.89308300
C	1.00063500	-1.20254600	-1.26738800
H	0.14700200	-0.54120500	-1.22616100
H	-0.19389700	-2.75202200	-0.62434700
H	1.47790700	-4.38951700	-0.53436000
H	3.83394500	-3.78882700	-1.18707200
H	4.30175200	-1.44486600	-1.92009500

9Z_NMe3H

conf_16

C	1.96002600	3.94719900	-0.62406300
C	0.67008700	3.66655300	0.15909900
C	-0.42289300	2.98431800	-0.66938100
C	-1.68088700	2.66402600	0.13962900

C	-1.48485800	1.56963200	1.15521200
O	-0.47491000	0.90108100	1.26226000
O	-2.51313800	1.29187000	1.97819200
H	-3.27218900	1.86554600	1.80339400
H	-2.05427400	3.55487100	0.65994900
H	-2.49386500	2.32853400	-0.51652300
H	-0.04897000	2.05592000	-1.10654300
H	-0.71045600	3.63051700	-1.50391100
H	0.90446900	3.04655800	1.02869200
H	0.28181500	4.61419200	0.54966800
C	2.61463600	2.71349500	-1.26300000
C	2.86040400	1.55025900	-0.29785800
C	3.65253000	0.40764100	-0.95313400
C	3.75716100	-0.80177300	-0.07002100
C	3.45196900	-2.06405800	-0.38755700
C	2.97778800	-2.57947100	-1.71952200
C	1.48230600	-2.94444800	-1.75224500
C	0.55045400	-1.73637700	-1.63846200
C	-0.93124500	-2.10392000	-1.75763000
C	-1.87070200	-0.93495300	-1.45855100
C	-3.35209100	-1.25625700	-1.65846100
C	-4.27969400	-0.09693700	-1.28442100
C	-5.75974800	-0.40748900	-1.51271100
H	-5.95251400	-0.63740600	-2.56401500
H	-6.07596600	-1.27224200	-0.92217900
H	-6.39619400	0.43664800	-1.23599800
H	-3.99850800	0.78912200	-1.86773600
H	-4.11941500	0.15941200	-0.22811800
H	-3.52604200	-1.53443300	-2.70413000
H	-3.62026400	-2.13839400	-1.06303600
H	-1.59534700	-0.08004500	-2.08924100
H	-1.70756500	-0.61138400	-0.42423700
H	-1.16423300	-2.93653200	-1.08022600
H	-1.12758400	-2.48477700	-2.76593400
H	0.80264000	-1.01174200	-2.42174200
H	0.73397000	-1.21126100	-0.69237600
H	1.27028900	-3.47260500	-2.68765700
H	1.26271900	-3.66008000	-0.94808400
H	3.55963400	-3.47389700	-1.96509400
H	3.18368900	-1.85503000	-2.51104600
H	3.58519800	-2.82667000	0.37949400
H	4.13830900	-0.60651500	0.93238800
H	4.66011400	0.77063500	-1.19248600
H	3.18491200	0.14753300	-1.90539100
H	3.40315700	1.91014000	0.58555900
H	1.90411000	1.15980200	0.06456500
H	3.56731500	3.02042400	-1.70759300
H	2.00165300	2.35513800	-2.09832800
H	1.75571600	4.68424200	-1.40841400
H	2.67604500	4.41931700	0.05709100
N	0.03090900	-1.33401900	2.71489400
C	-0.71031300	-1.22197600	4.00578700
H	-0.49953500	-2.09863900	4.61735100
H	-1.77579500	-1.15582600	3.79346900
H	-0.38413300	-0.31998000	4.52041200

C	1.51252700	-1.32628800	2.91089200
H	1.78934900	-0.41705400	3.44164200
H	1.79878500	-2.20175200	3.49346900
H	1.99399200	-1.34531900	1.93413400
C	-0.41221800	-2.51838300	1.92070300
H	-1.47790500	-2.42729500	1.71829600
H	0.14080400	-2.53707200	0.98518500
H	-0.21517400	-3.42462800	2.49267100
H	-0.20615500	-0.47821700	2.15890900

conf_48

C	4.84911800	0.29482600	-1.29012300
C	4.26219600	1.54470200	-1.96065600
C	2.93892200	2.03032600	-1.35221400
C	1.85253700	0.92683800	-1.33180800
C	0.69274000	1.37181400	-0.49766900
O	-0.24147800	2.03213300	-0.92803400
O	0.71325400	1.08276600	0.80972400
H	1.41280800	0.43338800	1.04220200
H	1.49919300	0.72679800	-2.34408900
H	2.26209000	0.00552400	-0.91653600
H	2.55971300	2.88409500	-1.91860500
H	3.11130600	2.38521500	-0.33238900
H	4.98059000	2.36841100	-1.89617100
H	4.11877700	1.34508200	-3.02772200
C	5.07124200	0.43143900	0.22100700
C	5.39557800	-0.89514400	0.91664400
C	4.20116400	-1.86037200	0.99307200
C	3.06679900	-1.36529700	1.84668700
C	1.76410100	-1.62626800	1.66546700
C	1.16265200	-2.42152000	0.53190700
C	-0.36363100	-2.30365300	0.44076500
C	-0.92778400	-2.97148900	-0.81600200
C	-2.45223900	-3.12409400	-0.83324800
C	-3.24157100	-1.81476800	-0.72337400
C	-4.74016800	-2.02009200	-0.97240200
C	-5.60053100	-0.76071200	-0.81150400
C	-5.75332000	-0.29229300	0.63851000
H	-6.23927600	-1.06237700	1.24361400
H	-4.78841800	-0.07942300	1.10711000
H	-6.36613500	0.61108600	0.70484400
H	-6.59610300	-0.95643300	-1.22050900
H	-5.18199000	0.04417500	-1.43037300
H	-4.86940600	-2.41343200	-1.98612400
H	-5.11452500	-2.79775000	-0.29566100
H	-2.85010500	-1.08978800	-1.45138700
H	-3.08436900	-1.38004600	0.27090500
H	-2.75767500	-3.79073200	-0.01789200
H	-2.74006000	-3.63029900	-1.76088300
H	-0.47883800	-3.96545900	-0.92020900
H	-0.60811600	-2.40181800	-1.69831200
H	-0.81001700	-2.75795300	1.33373100
H	-0.65006000	-1.24612200	0.46138900

H	1.42705600	-3.47949000	0.65066600
H	1.61141700	-2.12339000	-0.42372000
H	1.07033200	-1.30619200	2.44203500
H	3.35636100	-0.81676200	2.74185700
H	4.54809200	-2.80528000	1.43170100
H	3.84558800	-2.12132900	-0.00746000
H	6.22210000	-1.39151400	0.39855600
H	5.74790100	-0.69400000	1.93384300
H	4.18463300	0.86115200	0.70125600
H	5.88008900	1.14755800	0.39841900
H	4.20208100	-0.56398500	-1.49751400
H	5.80361700	0.05562000	-1.76897400
N	-2.34844300	2.62013700	0.64856400
C	-2.40681400	1.62527700	1.75974000
H	-2.48594600	0.62885100	1.33023400
H	-3.27562800	1.83449400	2.38285800
H	-1.48995000	1.69823200	2.33998200
C	-3.52706000	2.50933800	-0.26121300
H	-3.40419900	3.21572100	-1.08014500
H	-3.57223300	1.49544400	-0.65192400
H	-4.43592100	2.73135800	0.29670600
C	-2.16507300	4.00993000	1.15890000
H	-1.26128400	4.04429500	1.76478500
H	-2.06545200	4.68559300	0.31124700
H	-3.02947700	4.28964100	1.76070500
H	-1.49682200	2.38896200	0.06958400

conf_192_2

C	-1.85067000	3.68203900	1.19184500
C	-2.86843600	3.07412500	2.16782000
C	-3.39883400	1.69468200	1.75472800
C	-2.26917000	0.64743400	1.58147800
C	-2.79747700	-0.53811100	0.83678200
O	-3.44020300	-1.43772500	1.35828300
O	-2.61539500	-0.58079900	-0.49133700
H	-2.01579600	0.12892300	-0.81308400
H	-1.43129000	1.07875500	1.03420200
H	-1.91273000	0.31655600	2.55791900
H	-3.95649300	1.78516600	0.81830400
H	-4.10494500	1.32477600	2.50133000
H	-2.41796300	3.00781600	3.16373000
H	-3.72795800	3.74557800	2.26359000
C	-2.35109400	3.78765300	-0.25263800
C	-1.28636200	4.26149800	-1.24682700
C	-0.13045400	3.26496200	-1.44524400
C	-0.55480600	1.94266200	-2.01809100
C	-0.12833200	0.72434200	-1.65160300
C	0.86281200	0.40348800	-0.56598200
C	2.28505500	0.19313300	-1.11652600
C	3.29484900	-0.17572100	-0.02868600
C	4.71385300	-0.37590300	-0.56512400
C	5.72678600	-0.74092300	0.52220500
C	7.14619900	-0.94117300	-0.01264800

C	8.16098100	-1.30535200	1.07390300
C	9.57646600	-1.50305500	0.52876400
H	9.94252800	-0.59278400	0.04425000
H	10.27925600	-1.76150500	1.32477700
H	9.60410400	-2.30655200	-0.21361300
H	7.83204900	-2.21990400	1.58176600
H	8.16832800	-0.51856400	1.83756100
H	7.13821400	-1.72778000	-0.77788000
H	7.47448300	-0.02599000	-0.52136400
H	5.39888800	-1.65659100	1.03065000
H	5.73581100	0.04554800	1.28732400
H	5.04106400	0.53985100	-1.07262200
H	4.70444300	-1.16149800	-1.33092400
H	2.96567500	-1.09226900	0.47767300
H	3.30477100	0.60951900	0.73774700
H	2.26441900	-0.59063000	-1.88269700
H	2.60389300	1.10925300	-1.62515000
H	0.55445600	-0.51096900	-0.04338600
H	0.88864300	1.19824500	0.18521600
H	-0.43573900	-0.11992200	-2.26900800
H	-1.22670300	2.00623600	-2.87344500
H	0.42537600	3.12706100	-0.51575400
H	0.58230600	3.70819100	-2.15230300
H	-1.76009200	4.44892800	-2.21638300
H	-0.87202000	5.21951900	-0.91735900
H	-3.20734200	4.46991800	-0.28411100
H	-2.72966300	2.81774100	-0.59503700
H	-1.57795500	4.68028700	1.54859900
H	-0.92307800	3.10131200	1.22450700
N	-4.42371900	-3.38440800	-0.22568300
C	-5.31841200	-2.70334000	-1.20767100
H	-5.71722400	-3.44075700	-1.90382400
H	-4.73720600	-1.95299500	-1.74001300
H	-6.13193900	-2.22324800	-0.66653700
C	-5.16724400	-4.35360900	0.63188300
H	-5.57807000	-5.14658200	0.00725500
H	-4.48067300	-4.77292400	1.36515000
H	-5.97062000	-3.82620200	1.14305600
C	-3.25051200	-4.01628800	-0.89823000
H	-2.70603200	-3.24379200	-1.43759800
H	-2.60746200	-4.45866400	-0.13930400
H	-3.60044200	-4.78588100	-1.58579100
H	-4.03965000	-2.62939800	0.40597800

conf_0

C	3.78002500	-0.86293700	2.38960800
C	2.26533400	-1.11131600	2.43542000
C	1.45148800	0.15235700	2.13703300
C	-0.06174400	-0.04732400	2.19795200
C	-0.68279500	-0.92852100	1.14766400
O	-1.76196400	-1.47732200	1.31148800
O	-0.08895000	-1.08146100	-0.04286600
H	0.77379400	-0.63172800	-0.13671900

H	-0.38005000	-0.44929000	3.16199000
H	-0.57414300	0.91650300	2.08784800
H	1.72811000	0.57121100	1.16460100
H	1.71301500	0.92813300	2.86271500
H	1.99740100	-1.90666000	1.72902100
H	1.98828600	-1.48439100	3.42678400
C	4.29493800	-0.46435900	1.00080000
C	4.32604200	-1.61774800	-0.01614700
C	4.11582900	-1.13301200	-1.46449700
C	2.66402000	-0.94671000	-1.80692200
C	2.01991300	0.19737800	-2.06784100
C	2.58890700	1.59486200	-2.06025500
C	1.50234800	2.67791500	-2.00378400
C	0.60446400	2.59260100	-0.76727000
C	-0.48129300	3.67171600	-0.72578300
C	-1.49291100	3.49269900	0.41345600
C	-2.45687100	2.31710900	0.21808100
C	-3.45948300	2.15687100	1.36357200
C	-4.40822800	0.97075100	1.17886200
H	-3.85146900	0.02867100	1.18066700
H	-5.14292500	0.91680100	1.98545800
H	-4.96166800	1.05504600	0.23687800
H	-4.04075800	3.07971500	1.45951700
H	-2.91815100	2.04260700	2.31009400
H	-3.00356600	2.46092600	-0.72319300
H	-1.89336400	1.38346400	0.09726400
H	-2.08291500	4.40885100	0.51866800
H	-0.95534400	3.37298200	1.36352200
H	-1.01738500	3.68430100	-1.68297000
H	-0.00209000	4.65170500	-0.63503900
H	1.22012200	2.66690500	0.13818600
H	0.13137400	1.60503200	-0.73910200
H	1.97617600	3.66348800	-2.04288800
H	0.87871600	2.60409900	-2.90324800
H	3.18835600	1.74305900	-2.96641600
H	3.28145800	1.71910600	-1.22164900
H	0.97905200	0.12349900	-2.38167900
H	2.09788800	-1.87413100	-1.89136400
H	4.52306500	-1.87722100	-2.15759000
H	4.68061500	-0.21239500	-1.63271300
H	5.28125100	-2.14357600	0.06322800
H	3.55542100	-2.36034600	0.22008800
H	5.30186500	-0.04734000	1.09188300
H	3.67946300	0.35016900	0.60705800
H	4.02663600	-0.07594600	3.11055200
H	4.30356300	-1.76254600	2.72908400
N	-3.02537500	-2.33450000	-0.91999600
C	-2.24967300	-3.48868000	-1.46221500
H	-1.22332800	-3.16620900	-1.62597000
H	-2.69979300	-3.81718100	-2.39859800
H	-2.26924600	-4.29787300	-0.73423700
C	-2.99311300	-1.16474300	-1.84796100
H	-3.47260900	-1.43955700	-2.78707200
H	-3.52159500	-0.33536500	-1.38294600
H	-1.95445300	-0.88929200	-2.01755900

C	-4.42152100	-2.71603000	-0.55393900
H	-4.38534500	-3.52610600	0.17228200
H	-4.91620400	-1.85155200	-0.11549700
H	-4.95191600	-3.03929400	-1.44935700
H	-2.53642500	-2.03379300	-0.03559700

conf_111_2

C	-1.08075600	-0.69443300	1.99044400
C	-2.16112500	-1.76566400	2.15158500
C	-1.88979800	-3.07226500	1.39688300
C	-1.72265300	-2.89207100	-0.12313900
C	-0.35718900	-2.40219600	-0.52801400
O	0.67886400	-2.85204400	-0.06702300
O	-0.26128900	-1.46140100	-1.47889300
H	-1.12656200	-1.08542800	-1.70642300
H	-1.83098800	-3.86365000	-0.61937600
H	-2.50253600	-2.24272600	-0.52866100
H	-2.72206000	-3.76028500	1.56158200
H	-0.99456300	-3.56243600	1.78870600
H	-2.27809400	-2.00223500	3.21411700
H	-3.12417600	-1.35276000	1.82728900
C	-1.31957500	0.53843200	2.87004300
C	-0.50226400	1.76616000	2.45177600
C	1.01115800	1.50858300	2.34428200
C	1.78316900	2.76679700	2.06572200
C	2.36802900	3.12780300	0.92117600
C	2.40975200	2.36611000	-0.37337600
C	1.73250700	3.12067400	-1.53566000
C	0.21424800	3.27061100	-1.38678700
C	-0.54395600	1.94342700	-1.46771400
C	-2.06407800	2.10067900	-1.38864800
C	-2.81031500	0.76519000	-1.39524300
C	-4.33510100	0.88727300	-1.35028600
C	-5.03922000	-0.46905900	-1.29392900
H	-4.78588700	-1.08463100	-2.16337400
H	-4.75644100	-1.02421300	-0.39315100
H	-6.12510200	-0.35553100	-1.27886800
H	-4.67673200	1.44678500	-2.22718600
H	-4.61627100	1.48523800	-0.47665100
H	-2.53559300	0.20275000	-2.30427500
H	-2.48552100	0.17588300	-0.52784600
H	-2.41276200	2.71688800	-2.22463000
H	-2.32669200	2.64832600	-0.47613900
H	-0.21468700	1.28030200	-0.66010600
H	-0.28040400	1.43773000	-2.40688100
H	-0.15324500	3.93834300	-2.17280900
H	-0.01170000	3.76209900	-0.43431000
H	2.18905900	4.11187700	-1.61999300
H	1.95349000	2.60220700	-2.47674600
H	1.93889500	1.38643300	-0.25491000
H	3.45824100	2.18750200	-0.64570800
H	2.86460300	4.09557500	0.89175000
H	1.83504900	3.46557900	2.89767500

H	1.19255100	0.76148800	1.56695800
H	1.36372600	1.07027500	3.28658100
H	-0.86042900	2.13116900	1.48333600
H	-0.67820800	2.57724000	3.16520500
H	-1.09953500	0.28191500	3.91250800
H	-2.38268900	0.80153400	2.83950200
H	-0.10134600	-1.12973200	2.21613200
H	-1.03624200	-0.36594700	0.94514500
N	3.16593500	-1.95885600	-0.67204800
C	3.65796800	-1.21699600	0.52802700
H	4.69179100	-0.91510600	0.36267400
H	3.59321600	-1.87250500	1.39451800
H	3.03679300	-0.33788300	0.68217600
C	3.13309900	-1.09332500	-1.88912200
H	2.73194200	-1.67209500	-2.71871800
H	2.48942700	-0.23899700	-1.69555400
H	4.14510200	-0.75987200	-2.11672600
C	3.94524900	-3.21178500	-0.90314300
H	3.51067100	-3.74539800	-1.74645700
H	4.98244200	-2.95500900	-1.11685700
H	3.88836100	-3.83049800	-0.00947900
H	2.17606500	-2.25432600	-0.47162100

conf_8

C	-3.67430800	0.03170900	-1.65630700
C	-2.39373700	-0.71811400	-2.02572300
C	-1.21327300	0.24208700	-2.20405300
C	0.13840900	-0.43600600	-2.42049100
C	0.77931100	-1.11577300	-1.23702900
O	1.86361700	-1.67181400	-1.32620100
O	0.19593600	-1.09349700	-0.03298800
H	-0.67882900	-0.66102600	-0.01020900
H	0.08513100	-1.19186700	-3.21084400
H	0.88903800	0.28491000	-2.76147600
H	-1.15344500	0.92267200	-1.34794800
H	-1.40601000	0.88701000	-3.06558200
H	-2.17155800	-1.46140500	-1.25227700
H	-2.54437200	-1.28840800	-2.94925400
C	-4.86656800	-0.87059600	-1.32799200
C	-4.68415900	-1.78496500	-0.10673400
C	-4.28723000	-1.04554300	1.19121200
C	-2.81310700	-1.01364200	1.48683100
C	-2.08911300	0.03539300	1.89557300
C	-2.57090100	1.45183700	2.08964400
C	-1.42780200	2.47310900	2.16861100
C	-0.57030700	2.52942900	0.90359000
C	0.56293100	3.55666700	0.96537000
C	1.51359800	3.49818700	-0.23784900
C	2.41988200	2.26263300	-0.25624400
C	3.36087500	2.21056200	-1.46222600
C	4.29952100	1.00174900	-1.44800200
H	3.73594900	0.06405600	-1.47910800
H	4.97221300	1.00728500	-2.30866100

H	4.92445200	1.00656600	-0.54752300
H	3.95292300	3.13107100	-1.49389200
H	2.76845900	2.20325700	-2.38484200
H	3.01592500	2.25229000	0.66662100
H	1.81108300	1.35035100	-0.23434400
H	2.14647800	4.39153000	-0.24150800
H	0.92993000	3.53720300	-1.16635500
H	1.13927700	3.40974000	1.88772500
H	0.12881400	4.55901400	1.03704900
H	-1.21091800	2.75571800	0.04191000
H	-0.14556500	1.53698300	0.71985800
H	-1.84615200	3.46397600	2.37042200
H	-0.78897000	2.23154800	3.02736500
H	-3.15959400	1.50636900	3.01317900
H	-3.25835800	1.73109700	1.28361500
H	-1.05614400	-0.14942600	2.18874500
H	-2.32162400	-1.98583500	1.44774900
H	-4.75570700	-1.56213600	2.03822900
H	-4.70740200	-0.03617100	1.19368000
H	-5.62851900	-2.30981800	0.05800100
H	-3.94666800	-2.56709900	-0.31934500
H	-5.10944700	-1.48809800	-2.19999600
H	-5.74096200	-0.23414000	-1.15501900
H	-3.46560500	0.68369400	-0.80136000
H	-3.94686900	0.69735200	-2.48233800
N	3.18693200	-2.23731300	0.95824000
C	2.50401900	-3.39485700	1.60690400
H	2.99837400	-3.62226300	2.55098900
H	2.55853000	-4.25381900	0.94033400
H	1.46290800	-3.12867500	1.77966400
C	3.09313600	-1.00288800	1.79328300
H	3.55998900	-0.18115000	1.25514900
H	2.04183400	-0.77870900	1.96037600
H	3.60278500	-1.17243000	2.74138600
C	4.59824800	-2.55120600	0.58617700
H	5.17031500	-2.76037600	1.48984500
H	4.60381300	-3.41989900	-0.06975400
H	5.01959200	-1.69441700	0.06394800
H	2.66341400	-2.04148100	0.06437800

conf_43

C	0.42494300	-2.00008200	-2.27241300
C	-0.92903700	-1.27995800	-2.25745600
C	-2.15935400	-2.18591800	-2.13405100
C	-2.16719700	-3.06338300	-0.85911000
C	-1.78131000	-2.29906300	0.37753000
O	-0.81490900	-2.56969600	1.06844000
O	-2.52712400	-1.24136600	0.73993900
H	-3.22745300	-1.03814800	0.10157900
H	-3.16355300	-3.49189300	-0.70721900
H	-1.46418800	-3.88958100	-0.94486600
H	-2.22801300	-2.86104800	-2.99092300
H	-3.06547600	-1.57127800	-2.16735500

H	-0.93267600	-0.56006000	-1.42967900
H	-1.03004200	-0.67525500	-3.16366800
C	1.57487100	-1.01695500	-2.05148100
C	2.96599500	-1.64747000	-2.03054600
C	4.05189700	-0.64650200	-1.58964500
C	3.93458400	-0.26582700	-0.14000100
C	3.73082100	0.94532800	0.38761700
C	3.57636100	2.26113800	-0.32241500
C	2.29819900	3.02007400	0.07937300
C	1.01841000	2.37179200	-0.44879600
C	-0.26801500	3.06661600	-0.00083700
C	-1.53090000	2.41873100	-0.57490600
C	-2.82870700	3.06614500	-0.08196100
C	-4.08716900	2.61608100	-0.83432200
C	-4.37526500	1.11366500	-0.74651700
H	-3.58086900	0.54039900	-1.23697800
H	-4.45581900	0.79814800	0.30064200
H	-5.31499500	0.86127100	-1.24266900
H	-3.99584900	2.90070800	-1.88832500
H	-4.94950500	3.16450700	-0.44385100
H	-2.95214100	2.86435700	0.99055000
H	-2.74010100	4.15381700	-0.17522800
H	-1.53079900	1.35059000	-0.32630500
H	-1.49074500	2.47241600	-1.66998200
H	-0.23594100	4.12053100	-0.29906500
H	-0.32313700	3.07482100	1.09659600
H	0.99479200	1.31878000	-0.14335200
H	1.05385800	2.34913900	-1.54440900
H	2.25170800	3.09636000	1.17384400
H	2.36075000	4.04769800	-0.29228600
H	3.59433800	2.12668900	-1.40666600
H	4.44266000	2.88599100	-0.07475400
H	3.70948300	1.02223200	1.47540700
H	4.05834500	-1.10065600	0.55052000
H	4.01575100	0.23658900	-2.23229700
H	5.03593400	-1.10262700	-1.74598900
H	2.97113300	-2.50862200	-1.35038200
H	3.21470800	-2.03749700	-3.02244300
H	1.40921200	-0.50733700	-1.09687600
H	1.54421600	-0.23244400	-2.81738800
H	0.46400300	-2.75770300	-1.48266000
H	0.55090100	-2.53561200	-3.21990300
N	0.18007700	-0.90440400	2.96733900
C	-0.37311000	-1.35321700	4.27879100
H	0.05183300	-0.74294000	5.07519800
H	-0.11393800	-2.39952100	4.43051600
H	-1.45580500	-1.24117100	4.25907200
C	-0.21189100	0.50112600	2.65019900
H	0.20588800	0.77004300	1.68356000
H	-1.29741400	0.56163600	2.61034600
H	0.17962800	1.16362700	3.42148800
C	1.65913300	-1.09797900	2.88468700
H	2.14201100	-0.48551500	3.64587500
H	1.88387800	-2.14981600	3.05224500
H	1.99810800	-0.79749800	1.89458800

H	-0.24850300	-1.52083600	2.23265100

conf_168

C	1.01145100	3.79338700	-0.52546300
C	2.46036600	3.83433100	-1.03233800
C	3.27921500	2.57527800	-0.71727600
C	2.63530000	1.28524400	-1.28627700
C	3.27104900	0.08837000	-0.65160800
O	4.34293500	-0.38047700	-1.00594400
O	2.66361100	-0.45843400	0.41175800
H	1.76910000	-0.08199200	0.56995600
H	1.56326900	1.27654800	-1.08986200
H	2.79158000	1.23365400	-2.36472200
H	3.39229000	2.47654700	0.36599400
H	4.28730200	2.67087200	-1.12652300
H	2.45902600	4.00664200	-2.11374200
H	2.97964900	4.68859600	-0.58584700
C	0.87769500	3.50859900	0.97428800
C	-0.56787100	3.31441600	1.44290500
C	-1.23907500	2.04843600	0.88297100
C	-0.58318000	0.76780800	1.31497100
C	-0.36558400	-0.32861500	0.57297200
C	-0.70011700	-0.53166400	-0.88039200
C	-1.97785700	-1.37177700	-1.08334300
C	-3.24918800	-0.67874500	-0.59091200
C	-4.51382000	-1.50915000	-0.81809100
C	-5.78677200	-0.81458000	-0.32928700
C	-7.05061400	-1.64998600	-0.55210200
C	-8.35205600	-0.94984700	-0.14039800
C	-8.45860800	-0.65818600	1.35910800
H	-9.43017400	-0.22346400	1.60684000
H	-8.34664200	-1.57626300	1.94499700
H	-7.69145100	0.04516700	1.69356000
H	-9.19717300	-1.57689100	-0.44279700
H	-8.45102000	-0.01421700	-0.70397100
H	-7.11232100	-1.91962300	-1.61298700
H	-6.95985100	-2.59519000	-0.00143300
H	-5.89583000	0.14782700	-0.84601300
H	-5.67721000	-0.57981500	0.73534900
H	-4.40944100	-2.47475100	-0.30749300
H	-4.61215100	-1.73859400	-1.88629500
H	-3.35357600	0.28697200	-1.10168200
H	-3.15327900	-0.45266300	0.47688400
H	-2.07567300	-1.60036400	-2.14994400
H	-1.86271100	-2.33445200	-0.57078400
H	0.13338200	-1.04672700	-1.37386400
H	-0.82248600	0.42858500	-1.38953800
H	-0.00226900	-1.21690600	1.09096700
H	-0.33154300	0.71603700	2.37396700
H	-1.31747600	2.09947800	-0.20440800
H	-2.27318800	2.01889700	1.24900800
H	-0.58695400	3.26903200	2.53707900
H	-1.16844000	4.18512200	1.16143200

H	1.33570600	4.33014000	1.53540000
H	1.44649800	2.61079800	1.24225400
H	0.53535300	4.75306200	-0.75002300
H	0.44672800	3.05089300	-1.09867100
N	5.35248200	-2.44781100	0.39939800
C	5.51984300	-1.95222000	1.79761300
H	6.23168300	-1.12855900	1.79479600
H	5.88920300	-2.76256700	2.42560300
H	4.55390600	-1.60259000	2.15667300
C	6.65417700	-2.84178600	-0.21546200
H	6.48012700	-3.13568200	-1.24900500
H	7.32907100	-1.98823300	-0.18676100
H	7.07983300	-3.67457200	0.34400500
C	4.34186800	-3.54363000	0.32255600
H	4.68469500	-4.39242600	0.91372700
H	3.39686800	-3.16774900	0.70962000
H	4.22247600	-3.83830800	-0.71864100
H	4.96603800	-1.64041600	-0.16200800

conf_34

C	-0.87994000	4.29616200	-0.63618500
C	-1.99515500	3.30457900	-0.28035700
C	-2.16770200	2.16400400	-1.28793700
C	-3.21080700	1.14055600	-0.83295100
C	-2.76759100	0.34487700	0.36777200
O	-1.60276400	0.15999900	0.66527400
O	-3.71213500	-0.23405300	1.12795900
H	-4.60095400	-0.02265100	0.81098600
H	-4.17083700	1.62390000	-0.62067600
H	-3.39986400	0.40075900	-1.62159200
H	-1.22155400	1.64377700	-1.44890300
H	-2.47677000	2.56547500	-2.25702100
H	-1.79569700	2.88435800	0.71115700
H	-2.94112100	3.85139000	-0.19618300
C	0.52102900	3.67929200	-0.76441400
C	0.94069400	2.81723200	0.42948900
C	2.42248100	2.41735800	0.37871200
C	2.77314700	1.40285900	1.42957000
C	3.53544200	0.31533100	1.27961400
C	4.27693200	-0.10796300	0.03954900
C	3.62353100	-1.26694500	-0.73665900
C	2.34929500	-0.87628900	-1.48997700
C	1.80709500	-2.01564300	-2.35869300
C	0.51912400	-1.66758400	-3.11873400
C	-0.75084100	-1.64154900	-2.25662200
C	-1.21416500	-3.03041600	-1.80298500
C	-2.51139200	-3.00425800	-0.99299800
H	-2.40593900	-2.40504600	-0.08370400
H	-3.32840900	-2.56748300	-1.57582300
H	-2.82422500	-4.00916100	-0.69836300
H	-0.42601800	-3.51750700	-1.21688800
H	-1.35367300	-3.66386600	-2.68529700
H	-0.59962300	-0.99610400	-1.38427700

H	-1.56034000	-1.18113800	-2.83625100
H	0.37259100	-2.38778900	-3.93075800
H	0.65011200	-0.69211100	-3.60024100
H	1.65182300	-2.90969500	-1.74233100
H	2.58064000	-2.29234200	-3.08236100
H	2.56862800	-0.01784800	-2.13621400
H	1.58455800	-0.52745100	-0.78636400
H	4.35124400	-1.65254100	-1.45817200
H	3.41437700	-2.10025500	-0.05237400
H	5.28303800	-0.41638600	0.34156400
H	4.40954400	0.74034400	-0.63656000
H	3.68885900	-0.32098600	2.15078300
H	2.36705700	1.60957300	2.42017400
H	3.03532700	3.31725400	0.51822600
H	2.66152800	2.03359900	-0.61554800
H	0.74581800	3.35864800	1.36417400
H	0.32724800	1.91101000	0.46395500
H	1.24334200	4.49185900	-0.89635300
H	0.58563900	3.07506700	-1.67679600
H	-1.13199000	4.80723500	-1.57171300
H	-0.85664600	5.07163600	0.13684300
N	-0.38243100	-1.53605800	2.40356600
C	-1.36354600	-2.54176600	2.90759600
H	-2.18513300	-2.01873700	3.39364300
H	-0.86486300	-3.20236400	3.61605000
H	-1.74282900	-3.11477200	2.06380200
C	0.15820100	-0.68138400	3.50275000
H	0.82076000	0.06214500	3.06453100
H	-0.67247400	-0.19292000	4.00925400
H	0.70780700	-1.30830700	4.20433500
C	0.71542400	-2.17062700	1.61261500
H	1.38847200	-1.38947700	1.26620700
H	1.25152400	-2.87267300	2.25078600
H	0.27855300	-2.69181100	0.76346200
H	-0.90083900	-0.89871300	1.75506700

conf_186_2

C	3.47363800	1.77068200	-0.41313400
C	2.18771500	2.47592200	-0.86108300
C	0.99336200	1.52697300	-0.98140500
C	-0.27693600	2.24895800	-1.42857800
C	-1.46758500	1.33905600	-1.55132300
O	-1.40625700	0.12436600	-1.54215700
O	-2.68230100	1.90534000	-1.69708700
H	-2.62897800	2.87028600	-1.65867900
H	-0.52867000	3.06414900	-0.74049900
H	-0.12889200	2.71624500	-2.41155800
H	0.81075600	1.04601900	-0.01584400
H	1.20967500	0.72404500	-1.68810300
H	1.94294500	3.27028000	-0.14636700
H	2.36238400	2.97046100	-1.82472100
C	4.04084000	0.79399900	-1.44988800
C	5.36305500	0.14398900	-1.02590200

C	5.22616800	-0.86184700	0.13424600
C	4.43264300	-2.07995100	-0.24483500
C	3.28708400	-2.51214700	0.28726800
C	2.49367400	-1.87234500	1.39202200
C	0.98576900	-1.86112000	1.10452200
C	0.17924500	-1.13887700	2.18468600
C	-1.33081700	-1.18639500	1.94084200
C	-2.17188000	-0.45287700	2.99613100
C	-1.87312800	1.04562600	3.14192300
C	-2.02634300	1.84228300	1.84375500
C	-1.79123100	3.34146100	2.03249700
H	-0.77853100	3.53777000	2.39577100
H	-1.92287700	3.89944800	1.09969100
H	-2.48920000	3.76145300	2.76130500
H	-3.02964400	1.67388600	1.43092700
H	-1.31803000	1.45352500	1.10539700
H	-2.54392400	1.46415000	3.89958900
H	-0.85907800	1.18715200	3.53023700
H	-2.02487200	-0.93908400	3.96606700
H	-3.23750100	-0.57734100	2.76103500
H	-1.64567300	-2.23778700	1.91447600
H	-1.53217600	-0.77440000	0.94447400
H	0.51356600	-0.09661900	2.23797700
H	0.40017200	-1.57606200	3.16555400
H	0.80384300	-1.38653200	0.13320700
H	0.63221300	-2.89611000	1.01232300
H	2.83637600	-0.84977300	1.57149900
H	2.66327900	-2.41864500	2.32953200
H	2.86886700	-3.43950600	-0.10157200
H	4.86657100	-2.67243100	-1.04872200
H	4.80195000	-0.37159500	1.01308100
H	6.23222200	-1.18450000	0.42637300
H	5.79925000	-0.37271700	-1.88748800
H	6.07767800	0.92377100	-0.74061500
H	4.19645800	1.33668600	-2.38961300
H	3.31505700	0.00234700	-1.66371400
H	4.23174200	2.52964500	-0.19257900
H	3.28071600	1.25034700	0.53162700
N	-3.53695500	-1.55051800	-1.45099100
C	-4.21230600	-1.47471500	-2.78052700
H	-5.07424700	-2.14120200	-2.78407800
H	-3.50496500	-1.77493700	-3.55158300
H	-4.53020200	-0.44778800	-2.95168800
C	-4.43084600	-1.08149300	-0.35003300
H	-5.30587200	-1.72878700	-0.29969900
H	-3.88026400	-1.11855900	0.58710200
H	-4.73083500	-0.05655900	-0.56033100
C	-2.98371500	-2.91154500	-1.17975100
H	-2.29869100	-3.17747400	-1.98274100
H	-2.44782700	-2.88645900	-0.23344200
H	-3.80347800	-3.62795900	-1.13290900
H	-2.72459100	-0.88667400	-1.48841500

conf_96_2

C	-3.64966200	-1.57576700	1.38251700
C	-2.28068200	-2.26220400	1.43567600
C	-1.25558300	-1.65669600	0.46996000
C	0.10985700	-2.33964600	0.58454200
C	1.20784800	-1.78826600	-0.28477100
O	2.39218200	-1.96631200	-0.05717700
O	0.87644300	-1.07585500	-1.37175100
H	-0.08342900	-0.95518600	-1.43343600
H	0.48595300	-2.30928900	1.60959100
H	0.02668800	-3.40360300	0.32915400
H	-1.15579100	-0.58669000	0.68067100
H	-1.64318700	-1.75080500	-0.55263000
H	-1.88087900	-2.19575100	2.45340000
H	-2.39559400	-3.33039000	1.21673900
C	-4.40230500	-1.77636300	0.06223300
C	-5.79081000	-1.12605600	0.04543800
C	-5.75903000	0.41490400	0.00543600
C	-5.22143400	0.94948500	-1.29148900
C	-4.11562500	1.67142700	-1.48618500
C	-3.11734500	2.11424400	-0.45207600
C	-1.67193600	2.01142900	-0.95658600
C	-0.62863800	2.37239500	0.10151700
C	0.79454700	2.45523700	-0.46006900
C	1.88193300	2.65443500	0.60392300
C	2.12640200	1.42299600	1.48067900
C	3.26475700	1.59413000	2.48864900
C	3.51436000	0.33700800	3.32383500
H	4.34594200	0.47396800	4.01862200
H	2.63065900	0.07875400	3.91421500
H	3.74368700	-0.52625100	2.68966600
H	3.03814900	2.43682100	3.14988400
H	4.18288900	1.87999200	1.95753200
H	1.21146800	1.16212900	2.02385000
H	2.34389200	0.55862600	0.83898200
H	1.62085100	3.50828300	1.23915500
H	2.82352300	2.93464600	0.11335900
H	0.83521200	3.28026400	-1.17923700
H	1.01128800	1.54305900	-1.03087400
H	-0.67504600	1.64022900	0.91621500
H	-0.88859200	3.33658400	0.55332200
H	-1.49203500	0.99008200	-1.32071700
H	-1.55006000	2.66287400	-1.82979300
H	-3.22830200	1.53491400	0.46873300
H	-3.30973800	3.15999500	-0.17782500
H	-3.89996400	1.99924100	-2.50188400
H	-5.82612400	0.71478400	-2.16578300
H	-5.19748200	0.80544200	0.85698400
H	-6.78450800	0.78127200	0.12820900
H	-6.34572900	-1.48817900	-0.82631800
H	-6.35483000	-1.45087100	0.92647600
H	-4.50841300	-2.85225100	-0.11858100
H	-3.82113000	-1.37505500	-0.77544600
H	-4.26488800	-1.96513500	2.20031500
H	-3.51777700	-0.50739900	1.58570300

N	4.43267300	-0.69567900	-1.30983700
C	3.93618400	0.53844300	-1.98886900
H	3.51504200	1.20524100	-1.24021200
H	3.16405200	0.25784800	-2.70219600
H	4.76759300	1.02344800	-2.49953200
C	4.99473200	-1.67673000	-2.28527900
H	5.27867200	-2.58094000	-1.74995700
H	4.23140800	-1.91140300	-3.02503100
H	5.86601100	-1.23985900	-2.77238000
C	5.40135400	-0.37759400	-0.21814600
H	6.28118800	0.10010200	-0.64841600
H	4.92034700	0.29099500	0.49247800
H	5.68296800	-1.30264200	0.28135400
H	3.60476600	-1.16166300	-0.85968400

conf_6_2

C	-3.84127100	1.76088100	1.44936800
C	-2.65970800	1.33335000	2.33094200
C	-1.32248700	1.89912600	1.82793000
C	-0.08191400	1.31779200	2.51515300
C	0.43166100	-0.00469900	2.00650100
O	1.55634600	-0.40745000	2.25338500
O	-0.34485100	-0.78943800	1.24251600
H	-1.20594500	-0.39889000	1.02542300
H	0.77011100	1.99730400	2.44646900
H	-0.26095300	1.18032300	3.58865200
H	-1.32292500	2.97838500	1.99862400
H	-1.22996100	1.78446600	0.74277500
H	-2.81120900	1.67115300	3.36062100
H	-2.63041200	0.23654700	2.39188000
C	-3.92391900	0.96546200	0.13840800
C	-4.55265700	-0.42237700	0.31036400
C	-4.26992600	-1.35884600	-0.87871000
C	-2.87006500	-1.90576700	-0.85598600
C	-1.87735300	-1.65345400	-1.71379100
C	-1.89929500	-0.75071200	-2.91798200
C	-0.56041900	-0.02882500	-3.13744600
C	-0.16937500	0.89648200	-1.98397000
C	1.21489000	1.52797500	-2.13578100
C	1.63284900	2.35714700	-0.91756300
C	3.04093400	2.96039400	-1.01872700
C	4.18855700	1.94194900	-0.94690400
C	4.34454600	1.28554400	0.42863900
H	4.55644100	2.03539200	1.19600200
H	3.44020600	0.75848800	0.74666700
H	5.17634700	0.57431400	0.43429400
H	5.12353300	2.44994400	-1.20032400
H	4.05496300	1.17439600	-1.71849700
H	3.11563200	3.51971000	-1.95735600
H	3.17609300	3.69430000	-0.21635800
H	1.56412600	1.73504000	-0.01631300
H	0.90793900	3.16671900	-0.77789100
H	1.23597700	2.16204700	-3.02919400

H	1.94913500	0.73469700	-2.31952700
H	-0.19701600	0.32742600	-1.04829800
H	-0.92457000	1.68511600	-1.87939500
H	0.22823600	-0.77930500	-3.28347500
H	-0.60561300	0.54380100	-4.06866700
H	-2.70540700	-0.01704900	-2.83406900
H	-2.12279400	-1.35025800	-3.80899200
H	-0.93783500	-2.18533000	-1.56052700
H	-2.67206500	-2.61055500	-0.04854400
H	-4.47494500	-0.83617900	-1.81632100
H	-4.96454800	-2.20468700	-0.83926400
H	-4.18658800	-0.89798100	1.22886300
H	-5.63274900	-0.30928000	0.44193900
H	-2.92201800	0.86352500	-0.29715900
H	-4.50585300	1.52444200	-0.60054800
H	-4.77690700	1.63832500	2.00326400
H	-3.75188800	2.83127800	1.23647200
N	2.43980100	-2.42103200	0.65702100
C	1.53611600	-3.59646400	0.83344000
H	1.82962300	-4.38173200	0.13745700
H	0.51471800	-3.27592000	0.63971100
H	1.62150100	-3.95447200	1.85799300
C	3.85545100	-2.73783800	1.00984600
H	4.23239400	-3.50124200	0.32970700
H	4.44756900	-1.82911400	0.92150300
H	3.88946200	-3.10100200	2.03546100
C	2.32405200	-1.84141100	-0.71481800
H	2.96675800	-0.96666500	-0.78037100
H	2.63029900	-2.58893000	-1.44599900
H	1.28920600	-1.55150400	-0.88090200
H	2.10735700	-1.67896700	1.32438000

conf_200_2

C	-3.42646000	-1.86757100	0.77393700
C	-2.16120100	-2.73164600	0.68548200
C	-1.00807200	-2.01091700	-0.01519900
C	0.25330500	-2.86327800	-0.13289900
C	1.35732300	-2.17252100	-0.89099000
O	1.27558200	-1.03207200	-1.30685300
O	2.49012300	-2.85195400	-1.11319100
H	2.45039900	-3.74139100	-0.73537700
H	0.64675500	-3.12782900	0.85729300
H	0.03823300	-3.81424400	-0.63656900
H	-0.76727900	-1.09428500	0.52918700
H	-1.31100400	-1.70065900	-1.01706700
H	-1.84732200	-3.02207000	1.69463800
H	-2.39311900	-3.66263000	0.15352300
C	-4.07361600	-1.58314200	-0.58624300
C	-5.35099800	-0.73971100	-0.50917700
C	-5.13006600	0.69558200	0.00575900
C	-4.19341100	1.49139100	-0.85840600
C	-3.09361200	2.14971200	-0.48271600
C	-2.51686300	2.24398200	0.90144800

C	-0.99711000	2.02885300	0.94009200
C	-0.44329300	2.02831300	2.36746600
C	1.08006400	1.90007500	2.46628500
C	1.63969200	0.56786900	1.95892800
C	3.14825500	0.42746300	2.17529300
C	3.74428000	-0.83959900	1.55889200
C	5.24535800	-0.98698600	1.81160400
H	5.46064800	-1.03423300	2.88232300
H	5.64413500	-1.89436200	1.35145000
H	5.80013600	-0.13556300	1.40469000
H	3.55297200	-0.84598100	0.47893100
H	3.21805400	-1.71416500	1.96183200
H	3.66404500	1.30620300	1.76522800
H	3.36064000	0.44243900	3.25013400
H	1.40776200	0.44062800	0.89517700
H	1.12460000	-0.25520500	2.47077400
H	1.38070600	2.03139700	3.51108100
H	1.55037600	2.72818500	1.91732800
H	-0.90985700	1.21090300	2.93102600
H	-0.75482700	2.95322100	2.86439100
H	-0.75968400	1.08476700	0.43792200
H	-0.50704200	2.82633300	0.36303900
H	-2.99587100	1.52211400	1.56754500
H	-2.73659500	3.23409900	1.32208600
H	-2.55508900	2.70920900	-1.24756300
H	-4.47316100	1.53873600	-1.90997100
H	-4.78756900	0.67629200	1.04212700
H	-6.10157500	1.20410000	0.02113500
H	-5.80202700	-0.69108700	-1.50628400
H	-6.08340400	-1.23751700	0.13533400
H	-4.30775700	-2.53860800	-1.06986900
H	-3.36033400	-1.07058000	-1.23978300
H	-4.15679200	-2.37012900	1.41687500
H	-3.17429800	-0.92624300	1.27471200
N	2.37508300	1.23280100	-2.24467700
C	3.13886200	1.94753600	-1.17843300
H	4.00842300	1.35141900	-0.90842800
H	2.49576400	2.07094700	-0.31060800
H	3.45227600	2.91999800	-1.55689600
C	1.09408200	1.93315200	-2.56942600
H	0.49783200	2.00644500	-1.66226500
H	1.31939500	2.92539000	-2.95925700
H	0.55560600	1.34908700	-3.31305100
C	3.20795700	0.99510700	-3.46124000
H	2.62663700	0.41679300	-4.17701800
H	3.48978000	1.95392400	-3.89514000
H	4.09807700	0.43790800	-3.17436600
H	2.10330100	0.29686400	-1.86993000

conf_179_2

C	-4.61578000	1.01428600	-0.36534200
C	-3.47970100	2.04590600	-0.34561600
C	-2.64753000	2.08793900	-1.63153100

C	-1.45024400	3.03982500	-1.52139000
C	-0.46790000	2.57379200	-0.47684100
O	-0.25778500	1.40490800	-0.22262700
O	0.21576100	3.50452900	0.21944400
H	-0.01927900	4.39885600	-0.06323200
H	-0.90066600	3.08279500	-2.46959100
H	-1.77820200	4.06068500	-1.29899300
H	-3.27016800	2.40782100	-2.47133400
H	-2.27027500	1.09360100	-1.87618300
H	-3.90474100	3.03870700	-0.15934800
H	-2.82100100	1.83118600	0.50361700
C	-4.17709300	-0.43051700	-0.64194200
C	-3.09360700	-0.97322400	0.29644300
C	-2.75678200	-2.44054400	-0.01078300
C	-1.70446300	-3.03251400	0.88207400
C	-0.57666900	-3.62880700	0.48157300
C	-0.12798900	-3.81652800	-0.94729200
C	1.35095100	-4.20063300	-1.09806600
C	2.35517100	-3.18114200	-0.54553200
C	2.21620300	-1.77052200	-1.12507900
C	3.37832300	-0.84720600	-0.74496600
C	3.13859800	0.61768800	-1.11259500
C	4.27620400	1.55743500	-0.70786100
C	3.96586500	3.02674400	-0.99919700
H	3.06955800	3.35361800	-0.46082000
H	4.79083100	3.67889800	-0.70349500
H	3.78639000	3.18393800	-2.06688800
H	5.19320900	1.26271500	-1.22863700
H	4.49329500	1.43264100	0.36164400
H	2.97225700	0.69677300	-2.19371800
H	2.20328800	0.95834200	-0.65085700
H	4.29621100	-1.20315100	-1.22554100
H	3.57687400	-0.92277600	0.33421900
H	1.26983300	-1.32365900	-0.79577700
H	2.15019300	-1.82763200	-2.21800800
H	3.36699800	-3.55120100	-0.74316100
H	2.26798300	-3.13336900	0.54763000
H	1.52057400	-5.16508000	-0.60714700
H	1.55820400	-4.36039300	-2.16133000
H	-0.33861600	-2.91399300	-1.52925900
H	-0.73246000	-4.60813500	-1.40660900
H	0.06306900	-4.07150800	1.24341400
H	-1.92644100	-3.01301900	1.94888500
H	-2.45844900	-2.52608700	-1.05871400
H	-3.67339700	-3.03586900	0.09197500
H	-2.18090600	-0.37479400	0.20070200
H	-3.42999400	-0.88230600	1.33816100
H	-3.82487200	-0.52118900	-1.67558900
H	-5.05909000	-1.07593800	-0.57538100
H	-5.13355600	1.05571400	0.59911600
H	-5.35502900	1.30731300	-1.11849000
N	0.76726400	0.59489800	2.17673200
C	-0.44588800	0.92549800	2.98268600
H	-0.68102100	1.97933600	2.84564700
H	-0.24234900	0.72115000	4.03317100

H	-1.27277400	0.31040100	2.63236500
C	1.93029500	1.46002100	2.53720700
H	2.76688700	1.20491700	1.89017700
H	1.65171900	2.50015000	2.38069200
H	2.19359100	1.28650300	3.58000000
C	1.09818500	-0.85962400	2.26004600
H	1.28643900	-1.11798700	3.30192500
H	0.26345200	-1.43750100	1.86578200
H	1.98476200	-1.04911400	1.66187800
H	0.51612400	0.82161500	1.18947400

conf_15

C	-3.62450500	-2.59688300	-0.55935600
C	-2.24119400	-3.25384500	-0.47378200
C	-1.24385400	-2.74563000	-1.53282400
C	0.22170200	-2.90694300	-1.12777900
C	0.70187100	-2.03583500	0.00738100
O	1.80030200	-2.17073800	0.51971600
O	-0.07341300	-1.03884200	0.45748500
H	-0.93983900	-1.01344200	0.02228200
H	0.45213400	-3.93742200	-0.84292300
H	0.89161500	-2.67533900	-1.96386600
H	-1.43502700	-1.69553600	-1.78415900
H	-1.40366700	-3.28370900	-2.46989700
H	-1.84460600	-3.08340100	0.53569400
H	-2.33308800	-4.33921100	-0.57092300
C	-3.59805600	-1.09485600	-0.25519000
C	-4.98608900	-0.47582000	-0.07613000
C	-4.94462800	1.05461500	0.08218500
C	-4.14211100	1.49793700	1.27287000
C	-3.06923900	2.29364000	1.28637100
C	-2.39541100	2.94777100	0.11385000
C	-0.90600100	2.58669400	0.00323600
C	-0.21212100	3.27771700	-1.17417500
C	1.29272900	3.00390000	-1.27508200
C	1.65781600	1.55245800	-1.60275400
C	3.16642200	1.32514500	-1.72592500
C	3.55514300	-0.13118300	-1.99243000
C	5.06454800	-0.33681900	-2.13268400
H	5.46478000	0.24683700	-2.96577600
H	5.59809800	-0.01836700	-1.23042400
H	5.31217300	-1.38568900	-2.31152800
H	3.05610600	-0.47069700	-2.90749900
H	3.17097200	-0.76932200	-1.18709500
H	3.56263800	1.95802400	-2.52782200
H	3.66613800	1.67671200	-0.81096500
H	1.17313800	1.26465000	-2.54395600
H	1.24829700	0.88075100	-0.83954400
H	1.77600200	3.30041300	-0.33361100
H	1.72288400	3.65351900	-2.04454600
H	-0.36998400	4.35798500	-1.08779800
H	-0.69840300	2.97516400	-2.10977100
H	-0.39871500	2.86550600	0.93686600

H	-0.81044600	1.49939900	-0.08429400
H	-2.48032500	4.03719600	0.21608500
H	-2.89789300	2.69079300	-0.82168400
H	-2.62400600	2.51969600	2.25454400
H	-4.49827400	1.11799800	2.22949500
H	-5.97219900	1.41930600	0.19339800
H	-4.55901600	1.50045200	-0.83823100
H	-5.61137800	-0.72672300	-0.93920200
H	-5.46985800	-0.92516500	0.79855600
H	-3.08482300	-0.55051500	-1.05894300
H	-3.02333300	-0.91767500	0.66471000
H	-4.05906600	-2.76562300	-1.55107700
H	-4.28995300	-3.09302000	0.15403900
N	2.78346000	-0.33423900	2.26420000
C	2.57089400	-0.93280000	3.61526000
H	3.00945500	-0.28067400	4.37001400
H	3.04499300	-1.91218500	3.64461100
H	1.50095600	-1.03637200	3.78704300
C	2.07367300	0.97247300	2.12101600
H	2.45507300	1.66780700	2.86824800
H	2.25186800	1.36098900	1.12184700
H	1.00734800	0.80881800	2.25944100
C	4.23373400	-0.22056000	1.92583500
H	4.68832900	-1.20718900	1.99350200
H	4.32609700	0.15611000	0.90955900
H	4.71278500	0.46322500	2.62607600
H	2.36074400	-1.00481800	1.57316400

conf_173_2

C	1.14518700	2.93483100	-0.61970600
C	2.10375300	3.22529700	0.53963300
C	3.48501700	3.72858800	0.10235100
C	4.23197400	2.77230800	-0.85394900
C	4.32403700	1.36497700	-0.33036700
O	3.89348100	0.38864700	-0.91510800
O	4.91216800	1.17400500	0.86552900
H	5.19081100	2.01274800	1.25931300
H	3.73695600	2.71760100	-1.82157300
H	5.25032300	3.13895400	-1.02558900
H	4.09870300	3.93100400	0.98885000
H	3.39019800	4.68787100	-0.41330800
H	1.65784200	3.97577000	1.20017700
H	2.22569500	2.32557900	1.15461200
C	-0.26617100	2.53270100	-0.16751000
C	-0.33055300	1.32250200	0.77397400
C	0.33566300	0.05342100	0.21468200
C	0.21125200	-1.10208600	1.16719200
C	-0.32835000	-2.30196500	0.92756300
C	-0.93825200	-2.79089300	-0.35704400
C	-2.48087900	-2.80033600	-0.31450900
C	-3.09188600	-1.40088400	-0.23092300
C	-4.62122600	-1.40435400	-0.21010300
C	-5.22275200	0.00001500	-0.12220800

C	-6.75236500	0.00869700	-0.09870100
C	-7.35254100	1.41402700	-0.01044800
C	-8.88203600	1.41171300	0.01279400
H	-9.28876600	0.94884400	-0.89141900
H	-9.26185100	0.84939800	0.87119000
H	-9.28308300	2.42640300	0.07613300
H	-6.99937000	2.01015000	-0.86052000
H	-6.97263600	1.91162500	0.89001000
H	-7.13160800	-0.49034800	-0.99930800
H	-7.10525700	-0.58904700	0.75110300
H	-4.87078600	0.59822000	-0.97262600
H	-4.84450400	0.49941900	0.77919900
H	-4.97026300	-2.00246100	0.64059900
H	-4.99803500	-1.90552900	-1.11005200
H	-2.74419100	-0.80444600	-1.08489300
H	-2.72157000	-0.89593100	0.66775900
H	-2.84997400	-3.30852400	-1.21153900
H	-2.81553100	-3.39973100	0.54005000
H	-0.58931500	-3.81261100	-0.54893200
H	-0.61368400	-2.18141400	-1.20605800
H	-0.38658900	-3.00193200	1.75942500
H	0.56034800	-0.89976300	2.17993100
H	1.39715100	0.26038200	0.02800800
H	-0.10488000	-0.18903200	-0.75538100
H	0.12665200	1.57036200	1.73846100
H	-1.37950700	1.10126700	0.98912900
H	-0.87009500	2.32594200	-1.05749600
H	-0.74066800	3.38497900	0.33057700
H	1.07025400	3.82553200	-1.25355200
H	1.55712400	2.14528100	-1.25623100
N	3.90782100	-2.23242200	-0.14354600
C	2.83348400	-2.91365600	-0.92904900
H	2.88824600	-3.98669300	-0.74701100
H	2.98783700	-2.70421200	-1.98597500
H	1.86998400	-2.53017900	-0.60104100
C	3.68932500	-2.36945400	1.32836700
H	2.68231500	-2.02809500	1.56044800
H	3.80041200	-3.41700200	1.60709400
H	4.42730100	-1.76027400	1.84733900
C	5.27063700	-2.68860800	-0.54747600
H	6.01104300	-2.11986800	0.01245400
H	5.37143700	-3.75120800	-0.32911400
H	5.39882400	-2.51198100	-1.61392700
H	3.86286400	-1.21236000	-0.37226700

conf_31

C	-3.09557000	-1.60293900	-1.01786800
C	-1.66068800	-2.09554500	-0.80831900
C	-0.73156500	-1.76832300	-1.98154200
C	0.62991000	-2.50366700	-1.97116700
C	1.51100100	-2.14384700	-0.80796800
O	2.52682900	-1.47969400	-0.89348500
O	1.15659000	-2.58626700	0.41812800

H	0.30630700	-3.04864900	0.39231300
H	0.45683600	-3.58575900	-1.95346100
H	1.19088200	-2.26787300	-2.87463100
H	-0.55908500	-0.68956200	-2.03137600
H	-1.22274700	-2.04684200	-2.91772200
H	-1.28068000	-1.63954900	0.11348900
H	-1.68733000	-3.18463400	-0.65032300
C	-3.98805000	-1.86874700	0.19665500
C	-5.41610000	-1.33043400	0.05153500
C	-5.51452300	0.20654500	0.03231400
C	-5.01948500	0.83179900	1.30661500
C	-4.19188000	1.86993700	1.44813600
C	-3.54586600	2.69295900	0.36932000
C	-2.01356200	2.76265800	0.49768200
C	-1.34068200	1.40568300	0.28085600
C	0.18516400	1.41693400	0.41991500
C	0.90914200	2.22081500	-0.66425200
C	2.43314700	2.12175000	-0.58316200
C	3.16350600	2.93673900	-1.65309100
C	4.68450200	2.79300100	-1.58294500
H	5.17975600	3.39387100	-2.34858600
H	5.06960100	3.12245700	-0.61169500
H	4.98995500	1.75244800	-1.73706800
H	2.88883000	3.99200000	-1.55122500
H	2.81223800	2.62338200	-2.64237600
H	2.76371500	2.46143500	0.40935200
H	2.72411200	1.06791100	-0.68001300
H	0.61585400	3.27324200	-0.60440400
H	0.58112400	1.86979800	-1.65085900
H	0.55053700	0.38194500	0.39495100
H	0.45356600	1.81236300	1.40877300
H	-1.60232600	1.04125600	-0.71962500
H	-1.76713000	0.68717100	0.98823700
H	-1.63480400	3.49067700	-0.22558000
H	-1.74900100	3.14503900	1.49151700
H	-3.94368200	3.71329400	0.42513100
H	-3.81040200	2.31809000	-0.62334900
H	-3.95991600	2.19135100	2.46280500
H	-5.41060700	0.37699200	2.21586000
H	-6.56897500	0.47516700	-0.10598100
H	-4.98725400	0.61175200	-0.83448800
H	-5.86853400	-1.73133900	-0.86202400
H	-6.02005300	-1.70818000	0.88336400
H	-3.52732600	-1.42169600	1.08557100
H	-4.02973500	-2.94906200	0.37751500
H	-3.07204000	-0.53188800	-1.23647000
H	-3.52109400	-2.09071000	-1.90241800
N	3.94825300	-0.95127300	1.39390000
C	5.13105900	-0.09516400	1.07744400
H	4.78069500	0.86601300	0.70971600
H	5.72466100	-0.58748100	0.30925200
H	5.72492700	0.03824900	1.98125300
C	4.35330000	-2.31739100	1.84258300
H	4.90779200	-2.23727300	2.77692100
H	3.45494200	-2.91473600	1.98427200

H	4.97926600	-2.76764100	1.07416200
C	3.03489300	-0.29811700	2.37942100
H	2.73549100	0.67104000	1.98841600
H	2.15787700	-0.92910300	2.50779400
H	3.56056700	-0.17988100	3.32645300
H	3.40215600	-1.08635400	0.50911900

conf_56

C	3.99528100	-1.18841000	-1.81994000
C	2.47325600	-1.31844600	-1.66881400
C	2.03177400	-2.68653700	-1.13691400
C	0.51703700	-2.82692200	-0.96260700
C	-0.12177700	-2.01011000	0.13236700
O	-1.30816500	-1.73509800	0.14739000
O	0.62889900	-1.60740400	1.17070000
H	1.56373400	-1.82953900	1.04189900
H	-0.01661900	-2.58055200	-1.88299900
H	0.25331200	-3.86661800	-0.73147900
H	2.54641600	-2.92623000	-0.19638400
H	2.36239600	-3.46448700	-1.83059000
H	2.09512800	-0.51809100	-1.02144200
H	2.00227000	-1.15807500	-2.64423600
C	4.79257300	-1.33135100	-0.51133800
C	4.34561800	-0.39568300	0.61884100
C	4.50301100	1.09881300	0.28646900
C	3.89386400	1.98597100	1.33326700
C	2.80345700	2.74541000	1.19576000
C	1.94612300	2.88747800	-0.03026600
C	0.54964500	2.27269100	0.15570600
C	-0.33079300	2.37744400	-1.09070900
C	-1.72824000	1.78631100	-0.89597600
C	-2.62138000	1.88751900	-2.13616900
C	-4.02062400	1.28893600	-1.94622400
C	-4.04667000	-0.24029900	-1.84871700
C	-5.44821700	-0.79639000	-1.59110400
H	-5.44372400	-1.88763200	-1.52622600
H	-5.86852000	-0.40424100	-0.65818300
H	-6.13707300	-0.51622700	-2.39249100
H	-3.36308200	-0.58619100	-1.06681300
H	-3.65475200	-0.65941000	-2.78250000
H	-4.48175100	1.72505200	-1.04838700
H	-4.66001500	1.59564300	-2.78048600
H	-2.12663100	1.39182400	-2.98020800
H	-2.71425500	2.94235500	-2.41488400
H	-2.22337900	2.31248100	-0.06667300
H	-1.62991400	0.73650400	-0.59675400
H	0.16314900	1.86699500	-1.92656800
H	-0.41883600	3.42878100	-1.38718000
H	0.66188600	1.21964800	0.44317400
H	0.05368800	2.77176900	0.99855400
H	2.42287300	2.42920100	-0.90005300
H	1.83014800	3.95126500	-0.26917200
H	2.47764900	3.32444800	2.05802500

H	4.39466400	1.98486500	2.29923600
H	4.06061100	1.30612500	-0.68868200
H	5.57245700	1.32082100	0.19204900
H	3.29234400	-0.57676300	0.87220000
H	4.91143300	-0.62713400	1.52673600
H	5.84937300	-1.14748400	-0.72972000
H	4.74364600	-2.36718200	-0.15853800
H	4.34811900	-1.94445000	-2.52943300
H	4.21524500	-0.22259800	-2.28247900
N	-2.50619800	-0.50389100	2.26904900
C	-1.63722000	0.59340500	2.79104800
H	-0.64396000	0.19049300	2.97830400
H	-2.07135300	0.98364600	3.71106300
H	-1.57531200	1.37610200	2.03967200
C	-2.60862500	-1.63347000	3.24034800
H	-3.08900800	-1.27976600	4.15198800
H	-3.19860500	-2.43039300	2.79129100
H	-1.60580900	-1.99467600	3.46103100
C	-3.85155800	-0.00380400	1.85476500
H	-3.72031800	0.77817100	1.11097900
H	-4.41494800	-0.82749400	1.42102600
H	-4.36976800	0.38818800	2.72953000
H	-2.03460900	-0.89723000	1.41899200

conf_152_2

C	2.96190000	-1.78522300	-0.71185900
C	1.98552500	-1.35085000	-1.80872200
C	0.52474900	-1.76083900	-1.57205500
C	-0.04676600	-1.22194500	-0.25543300
C	-1.52445900	-1.40838400	-0.04404200
O	-2.16717200	-0.78781600	0.78386800
O	-2.17746700	-2.31620400	-0.79381600
H	-1.57770700	-2.73807000	-1.42693900
H	0.44200300	-1.69495600	0.60353100
H	0.14391800	-0.15362100	-0.14907800
H	0.46960200	-2.85875900	-1.58265200
H	-0.07570700	-1.39212800	-2.41205500
H	2.03397500	-0.26372400	-1.93222100
H	2.30344800	-1.77912400	-2.76433400
C	4.42303100	-1.57136300	-1.12234400
C	5.44154400	-1.98324900	-0.05309900
C	5.48688600	-1.05458900	1.17764000
C	5.97440700	0.32487200	0.83450400
C	5.31025100	1.47870100	0.93363200
C	3.89950100	1.67664100	1.40938300
C	2.94579700	2.07261200	0.27061400
C	1.50022500	2.24308900	0.73815900
C	0.51078900	2.49167700	-0.40247700
C	-0.94772100	2.52690800	0.06273200
C	-1.95701900	2.50363100	-1.08615800
C	-3.41112200	2.47593600	-0.61085400
C	-4.42241700	2.41529800	-1.75698900
H	-4.32650500	3.28741400	-2.40856300

H	-4.26520900	1.52956800	-2.38223200
H	-5.45469000	2.39895800	-1.39336700
H	-3.60789900	3.35964200	0.00609300
H	-3.53518100	1.60989800	0.05313700
H	-1.80333000	3.37292600	-1.73594400
H	-1.76917200	1.62240300	-1.71569300
H	-1.11191500	3.41915600	0.67791100
H	-1.14029400	1.66961500	0.71926800
H	0.63038700	1.70518900	-1.16033900
H	0.76093700	3.42755900	-0.91424400
H	1.43981800	3.06353000	1.46261000
H	1.19567900	1.34165400	1.28760600
H	3.29683600	2.99779000	-0.20008800
H	2.99096600	1.30235400	-0.50731000
H	3.88226200	2.46219500	2.17463100
H	3.52126900	0.77094300	1.89193800
H	5.82411500	2.38543700	0.62077100
H	6.99080500	0.36238300	0.44621000
H	6.17012100	-1.49730500	1.91151000
H	4.50648400	-1.01892900	1.65854500
H	5.23031300	-3.00678500	0.27735100
H	6.43778400	-2.00800200	-0.50671600
H	4.57795800	-0.51829000	-1.38200900
H	4.61376600	-2.14561400	-2.03557000
H	2.75424400	-1.23293900	0.21073700
H	2.80677700	-2.84758500	-0.48055200
N	-4.86007700	-1.10399100	1.03172000
C	-5.44930600	-1.00507400	-0.33770400
H	-6.51969100	-1.20061600	-0.28070000
H	-4.96317200	-1.74018700	-0.97609700
H	-5.27109400	-0.00454200	-0.72515500
C	-5.38840300	-0.04607500	1.94431200
H	-6.46064400	-0.18981100	2.07490100
H	-4.88039100	-0.12410500	2.90383500
H	-5.19077300	0.92826100	1.50199100
C	-5.02906700	-2.47219600	1.60606200
H	-4.54141600	-2.50782800	2.57863900
H	-6.09166100	-2.68826600	1.71226700
H	-4.56393700	-3.19034200	0.93331100
H	-3.82538600	-0.95271600	0.93080500

conf_70

C	4.34833100	-1.36932400	-0.84230700
C	4.86593400	0.06690300	-0.68497700
C	3.75295100	1.06965500	-0.37095100
C	4.26077400	2.49210100	-0.16173200
C	3.16584000	3.48522000	0.11108400
O	1.97045200	3.22948500	0.16952700
O	3.62422200	4.72043500	0.29151300
H	2.87887900	5.31842200	0.46165500
H	4.96772200	2.54795100	0.67296400
H	4.81642300	2.85178800	-1.03455100
H	3.21185300	0.75159600	0.52620200

H	3.03637300	1.06611700	-1.19894900
H	5.62431500	0.09674200	0.10592400
H	5.36970000	0.37692000	-1.60632400
C	3.95672500	-2.04073400	0.48026300
C	3.33702300	-3.43427100	0.30045000
C	1.86465400	-3.41767200	-0.13737500
C	0.92830500	-2.96909600	0.95974500
C	-0.40338200	-3.06349600	0.92830200
C	-1.21709400	-3.61909900	-0.21305800
C	-2.70212300	-3.23865600	-0.15154300
C	-2.95257400	-1.73833400	-0.31053300
C	-4.42134800	-1.32573400	-0.21044400
C	-4.62028400	0.18722600	-0.32357600
C	-6.08327700	0.62712400	-0.22614700
C	-6.30134000	2.13154900	-0.43166000
C	-5.62710900	3.01407800	0.62294700
H	-4.53611400	2.93219800	0.58795400
H	-5.87896200	4.06680400	0.47435300
H	-5.94768900	2.73532800	1.63165300
H	-7.37698300	2.33227300	-0.42700500
H	-5.94531900	2.41458800	-1.42980800
H	-6.66687100	0.07737500	-0.97265100
H	-6.48217800	0.33232800	0.75220900
H	-4.20548900	0.53714200	-1.27964600
H	-4.04063100	0.67782500	0.46917900
H	-4.83247100	-1.67626100	0.74336300
H	-4.99553600	-1.83101900	-0.99511500
H	-2.55587800	-1.41151200	-1.28213400
H	-2.38408600	-1.19824100	0.45909700
H	-3.24668500	-3.77900800	-0.93178200
H	-3.11984700	-3.57629900	0.80408600
H	-1.12990100	-4.71318800	-0.20629400
H	-0.79182900	-3.30124700	-1.17358700
H	-0.96121800	-2.77302200	1.81701700
H	1.39387400	-2.60137900	1.87210800
H	1.57538000	-4.42168000	-0.46518000
H	1.74617200	-2.78639300	-1.02978500
H	3.92761900	-3.99440400	-0.43127700
H	3.41007700	-3.99083500	1.23995600
H	3.26144800	-1.40519100	1.04348900
H	4.85362300	-2.12001200	1.10298600
H	3.50045400	-1.37106700	-1.53940500
H	5.12319600	-1.97756200	-1.31846400
N	0.12179900	1.20288800	0.06560600
C	-1.07176000	2.03507000	-0.28261200
H	-1.14901500	2.85117700	0.43284600
H	-1.96331500	1.41162100	-0.24589300
H	-0.93584800	2.43786500	-1.28454500
C	0.03882800	0.68098400	1.46380500
H	0.93830900	0.10841700	1.67740400
H	-0.04391900	1.52587500	2.14494200
H	-0.83003300	0.03204500	1.54862700
C	0.33021900	0.10233000	-0.92471100
H	-0.54383500	-0.54324600	-0.92089200
H	0.47394800	0.54413600	-1.90930400

H	1.20507400	-0.47178100	-0.63493300
H	0.94348100	1.84228400	0.03210700

conf_129_2

C	3.22754300	0.49794200	-1.91759300
C	4.57548800	0.67857400	-1.20705400
C	5.22562500	-0.62847700	-0.73041100
C	4.74294000	-1.10343300	0.65910400
C	3.28300200	-1.44897500	0.74704100
O	2.48524400	-0.88735100	1.47256800
O	2.82998700	-2.46702300	-0.01707600
H	3.52893600	-2.81775400	-0.58662600
H	5.30802100	-1.99400000	0.95864800
H	4.92945800	-0.32951000	1.40327700
H	6.30637000	-0.49567400	-0.64519300
H	5.08450400	-1.41544800	-1.48261600
H	5.26021900	1.18144000	-1.89603200
H	4.46732300	1.34932400	-0.34852100
C	2.50295500	1.82163700	-2.20242600
C	1.93752800	2.52079900	-0.95809100
C	0.78007500	1.75106900	-0.29513400
C	0.32638700	2.38392800	0.99108000
C	-0.72635800	3.18940300	1.16335800
C	-1.70755700	3.64993500	0.12464300
C	-3.17564100	3.47188000	0.55247000
C	-3.59717700	2.02050800	0.80777100
C	-3.47129500	1.10443400	-0.41214100
C	-4.03937500	-0.29645100	-0.17834100
C	-3.89415600	-1.22866400	-1.38245900
C	-4.36335000	-2.66003600	-1.11098400
C	-4.19434500	-3.58809700	-2.31458700
H	-4.53732600	-4.60102100	-2.09135500
H	-4.76774800	-3.22519100	-3.17195500
H	-3.14578200	-3.65030900	-2.62381900
H	-5.41461600	-2.64135100	-0.80373800
H	-3.81272700	-3.06901200	-0.25172400
H	-4.45952100	-0.81742700	-2.22642500
H	-2.84691900	-1.24537000	-1.71505900
H	-5.09834100	-0.21854600	0.09228900
H	-3.54908000	-0.74648300	0.69619100
H	-2.41845900	1.02193500	-0.70934900
H	-3.98594800	1.56311700	-1.26490000
H	-4.63769100	2.01444300	1.14981400
H	-3.00525800	1.60918200	1.63516200
H	-3.35298100	4.05887800	1.46007900
H	-3.81956900	3.90270300	-0.22137500
H	-1.52736500	3.15556300	-0.83260500
H	-1.53554600	4.71864900	-0.05312200
H	-0.89135000	3.59480200	2.16070000
H	0.97308600	2.20269000	1.84810500
H	1.11735100	0.73137400	-0.08239100
H	-0.04509700	1.67347400	-1.00994500
H	2.72795800	2.68629200	-0.21862400

H	1.57527300	3.51508200	-1.23393800
H	1.68444800	1.63644900	-2.90695000
H	3.19394100	2.50078100	-2.71309800
H	3.40262900	-0.02783200	-2.86266400
H	2.56748400	-0.15313300	-1.33602800
N	-0.15772400	-1.64021200	1.58968400
C	-0.94495500	-0.67784500	2.41977200
H	-0.49220000	-0.62005600	3.40820300
H	-1.97105100	-1.03453100	2.49692400
H	-0.91878600	0.30190400	1.94460000
C	-0.07527300	-2.98969400	2.22349700
H	-1.07751700	-3.41012300	2.29755200
H	0.55552300	-3.62619200	1.60562000
H	0.36133600	-2.88649900	3.21527000
C	-0.67651200	-1.72706700	0.19107500
H	-1.66270200	-2.18620800	0.20490400
H	-0.74139900	-0.72308700	-0.22001400
H	0.01543900	-2.32795600	-0.39605900
H	0.82461300	-1.28711200	1.53153000

conf_63

C	1.93378200	2.69764400	0.91453600
C	0.43820600	2.56877100	0.60018700
C	0.07171900	3.04562300	-0.80958800
C	-1.44018000	3.07178200	-1.07321900
C	-2.13044800	1.73472600	-1.01897300
O	-3.24322500	1.56910700	-0.55090800
O	-1.50498000	0.67461700	-1.55168700
H	-0.61215300	0.90691000	-1.84653700
H	-1.95268300	3.71943300	-0.36109600
H	-1.64349700	3.47637100	-2.07296600
H	0.58747400	2.44402300	-1.57020200
H	0.44798100	4.06154600	-0.95839100
H	0.12249000	1.52738200	0.73953300
H	-0.13524300	3.16102800	1.32177700
C	2.83315500	1.71735800	0.15542200
C	4.32232400	2.01836700	0.34022400
C	5.24178100	0.96748100	-0.30715500
C	5.27397400	-0.32797800	0.45353500
C	4.91898600	-1.54319500	0.03122800
C	4.36987300	-1.94015300	-1.31095600
C	3.11903700	-2.82655900	-1.18357700
C	1.92668100	-2.10267700	-0.55513700
C	0.75863200	-3.03639500	-0.23044100
C	-0.49965700	-2.31802200	0.27172700
C	-0.29474900	-1.48738600	1.54177900
C	-1.58302000	-0.84301200	2.05894700
C	-1.37420900	0.02044200	3.30406400
H	-0.67008900	0.83263100	3.10315700
H	-2.30919900	0.46983800	3.64957600
H	-0.96673300	-0.57259300	4.12686600
H	-2.30931800	-1.63605900	2.28351900
H	-2.02021700	-0.22964600	1.26024600

H	0.13258800	-2.12098600	2.32792300
H	0.44325100	-0.70034500	1.35578600
H	-1.27664700	-3.07021700	0.46279200
H	-0.88304300	-1.66551500	-0.52303200
H	1.08456400	-3.76258800	0.52365600
H	0.50188200	-3.62016200	-1.12204500
H	1.58821500	-1.30725000	-1.23497900
H	2.26263600	-1.60060700	0.35640400
H	2.83959000	-3.21030900	-2.17068800
H	3.36803800	-3.70325600	-0.57339300
H	5.13790500	-2.50046900	-1.85804200
H	4.13999700	-1.05983100	-1.91822200
H	5.03963900	-2.36973600	0.73046000
H	5.65323500	-0.24324300	1.47078600
H	6.26051600	1.37096400	-0.33870700
H	4.94368100	0.81018500	-1.34761300
H	4.54244700	3.00347000	-0.08544300
H	4.55168300	2.08781000	1.41032100
H	2.60914400	1.74307700	-0.91846800
H	2.62109100	0.69605300	0.48838800
H	2.25000300	3.72650900	0.70304600
H	2.08392600	2.55227300	1.98909500
N	-4.84993400	-0.60703600	-0.35940100
C	-4.17956100	-1.88543900	-0.74274600
H	-3.80088100	-1.79139400	-1.75880300
H	-4.90296400	-2.69822600	-0.68459700
H	-3.35142500	-2.06567300	-0.06221700
C	-5.96147700	-0.26056600	-1.29506700
H	-6.73374400	-1.02670400	-1.23331700
H	-6.36952200	0.70789100	-1.01197100
H	-5.56526100	-0.21075600	-2.30768700
C	-5.30912600	-0.62382700	1.06247600
H	-6.06175400	-1.40223200	1.18503300
H	-4.45286600	-0.82320300	1.70276600
H	-5.73256600	0.34919700	1.30445300
H	-4.14133200	0.16194000	-0.44420000

conf_91

C	-1.84817200	-1.78245900	0.82292900
C	-0.35735000	-1.98662100	0.54312600
C	-0.09968800	-2.47286500	-0.88553400
C	1.36363300	-2.84474400	-1.16559200
C	2.35562100	-1.72584100	-0.99632600
O	3.45133500	-1.86718400	-0.48012200
O	2.03939300	-0.51049000	-1.46800700
H	1.12514200	-0.47220900	-1.78693200
H	1.69402500	-3.65640700	-0.51683400
H	1.47319500	-3.19752200	-2.19890900
H	-0.45752100	-1.72254500	-1.60468800
H	-0.70644200	-3.36007500	-1.08564100
H	0.18155900	-1.04738900	0.72316100
H	0.05466800	-2.71323200	1.25320800
C	-2.12140000	-1.10847100	2.17032900

C	-3.58800500	-1.12552100	2.62244100
C	-4.57697100	-0.33138100	1.75028600
C	-4.91083000	-0.97844400	0.43177500
C	-5.15533600	-0.37946700	-0.73588900
C	-5.14679600	1.09249400	-1.03466300
C	-4.22786900	1.46425100	-2.21316900
C	-2.74460500	1.14929100	-1.98383100
C	-2.13650700	1.87864600	-0.78388600
C	-0.62553600	1.68544500	-0.65297400
C	-0.03484800	2.37057200	0.58093100
C	1.48825300	2.26263500	0.68531600
C	2.05101200	2.96585200	1.92218500
H	3.14424400	2.92563600	1.96041200
H	1.76866900	4.02170500	1.93256300
H	1.66773500	2.51460600	2.84176400
H	1.93211900	2.69491800	-0.22068300
H	1.77115000	1.20185000	0.69149900
H	-0.31483100	3.43025300	0.56951200
H	-0.49053000	1.94660900	1.48360000
H	-0.12961600	2.06578100	-1.55575700
H	-0.41208400	0.61022000	-0.60157200
H	-2.61507200	1.53629100	0.13850500
H	-2.35396000	2.95070300	-0.86235200
H	-2.18527800	1.41739500	-2.88841900
H	-2.62190200	0.06742500	-1.85041700
H	-4.56923900	0.93600400	-3.11011200
H	-4.33961000	2.53342200	-2.42469600
H	-4.87796400	1.67079300	-0.14808400
H	-6.16744800	1.39984100	-1.29308800
H	-5.41184800	-1.01543500	-1.58206000
H	-4.99972800	-2.06300600	0.46653300
H	-4.20382200	0.68705700	1.60516000
H	-5.50810900	-0.22722300	2.32208700
H	-3.92978800	-2.16547600	2.69252800
H	-3.63258000	-0.72477200	3.63975200
H	-1.76643900	-0.07061500	2.13060400
H	-1.52112900	-1.60490900	2.94262700
H	-2.29684300	-1.19446300	0.01889700
H	-2.34746000	-2.75777800	0.79353900
N	5.23315500	0.10329900	0.04529000
C	6.57798600	-0.47939600	-0.24075800
H	6.64285200	-0.70933000	-1.30271000
H	6.69597800	-1.39243800	0.33989800
H	7.34734100	0.24206900	0.03313200
C	5.04311000	0.37208800	1.50219300
H	5.18886800	-0.55619500	2.05149900
H	5.76806900	1.11808200	1.82670800
H	4.03218700	0.74132900	1.66063200
C	4.96191700	1.31694900	-0.78194500
H	5.06979900	1.05568600	-1.83301000
H	3.94423400	1.64880000	-0.59060400
H	5.67377800	2.09748200	-0.51483600
H	4.52001300	-0.62272000	-0.22007700

conf_134

C	-0.60255100	2.47680500	0.69235000
C	0.82090300	2.60310400	0.14635500
C	1.86968700	2.82181000	1.23757300
C	3.30776000	2.96977100	0.68926600
C	3.72362900	1.71707300	-0.02895700
O	4.00858300	0.67917700	0.53881200
O	3.73627800	1.71676400	-1.37782700
H	3.47496300	2.57766500	-1.73158100
H	3.37414200	3.83898400	0.02845300
H	4.00656000	3.11737200	1.51470800
H	1.85209300	1.99199400	1.94901100
H	1.63890600	3.72898800	1.80190300
H	1.05648800	1.69223300	-0.41975500
H	0.86070000	3.43301900	-0.57176800
C	-1.64388300	2.26594100	-0.40830400
C	-3.04536300	1.97834500	0.13015200
C	-4.07391300	1.73382700	-0.98623100
C	-5.41141500	1.31494800	-0.44613700
C	-5.99019200	0.11829700	-0.57129500
C	-5.46301500	-1.09032800	-1.29219700
C	-5.14660200	-2.26110900	-0.33865700
C	-4.02639700	-1.97980900	0.66950700
C	-2.64342100	-1.78405200	0.04080500
C	-1.54160300	-1.59008000	1.08491100
C	-0.15727700	-1.35429400	0.47427000
C	0.95000900	-1.10624000	1.50683700
C	1.26125600	-2.31606400	2.39200100
H	1.48939400	-3.20278500	1.78888100
H	2.11284300	-2.12113300	3.04933800
H	0.41409600	-2.57733800	3.02849000
H	0.66378700	-0.26097900	2.14224300
H	1.86467600	-0.78254000	0.99576800
H	0.10736000	-2.22266800	-0.14634400
H	-0.21493800	-0.49708700	-0.20649500
H	-1.51531800	-2.46621900	1.74104000
H	-1.79571600	-0.73782800	1.72722000
H	-2.65589600	-0.91954800	-0.63099200
H	-2.40257900	-2.65367900	-0.58438000
H	-3.97515800	-2.81407300	1.37836500
H	-4.28435900	-1.09214500	1.25787200
H	-6.05772200	-2.52244000	0.21014500
H	-4.88516900	-3.14186600	-0.93651200
H	-4.57925400	-0.84274400	-1.88412600
H	-6.22182900	-1.43060700	-2.00606000
H	-6.95493500	-0.02512400	-0.08772700
H	-5.93858100	2.07528900	0.12689000
H	-3.68184900	0.97957600	-1.67356400
H	-4.18691800	2.65482400	-1.57149800
H	-3.01028900	1.09697600	0.77838200
H	-3.37973300	2.80901500	0.76220700
H	-1.33124000	1.42772300	-1.04459400
H	-1.67159100	3.14744400	-1.06031200
H	-0.64271700	1.63775100	1.39614900

H	-0.85425800	3.37307700	1.27052500
N	4.31235900	-1.64979100	-0.84465600
C	3.10472800	-1.79517200	-1.71241400
H	2.22236300	-1.85971000	-1.07937900
H	3.20363300	-2.69913100	-2.31258000
H	3.03428000	-0.92027400	-2.35581000
C	4.42036200	-2.75804200	0.15120200
H	3.51371900	-2.77882100	0.75188700
H	4.54417900	-3.70268300	-0.37767100
H	5.28114900	-2.57186200	0.79091600
C	5.55811600	-1.50056600	-1.65485900
H	5.71117800	-2.40231100	-2.24688000
H	5.44693500	-0.63574100	-2.30627200
H	6.39939500	-1.34892600	-0.98089000
H	4.19691200	-0.75564300	-0.30756900

conf_174

C	3.59588500	-1.22419800	0.81766000
C	3.26330700	-2.50514000	0.03955100
C	2.95625000	-2.29034300	-1.44943900
C	1.78703700	-1.31861900	-1.71024900
C	0.50585800	-1.74752900	-1.05216800
O	-0.16261600	-1.05631800	-0.30713600
O	0.06124400	-2.99847800	-1.30740400
H	0.67692600	-3.47637000	-1.88111700
H	2.02124900	-0.31730800	-1.35418100
H	1.59631500	-1.24564700	-2.78745700
H	2.76403400	-3.26034500	-1.92633800
H	3.83076000	-1.88865900	-1.96434600
H	4.10245100	-3.20362400	0.11664500
H	2.41171500	-3.00685300	0.51749000
C	4.84088800	-0.49712800	0.30145200
C	5.22148700	0.74166800	1.11987000
C	4.16908900	1.86714300	1.09112900
C	3.87071900	2.34395400	-0.30202300
C	2.67660000	2.47789400	-0.88587900
C	1.32696300	2.21550600	-0.28139000
C	0.52829600	3.51055700	-0.04959300
C	-0.86108200	3.27938700	0.55566000
C	-1.80005700	2.45120800	-0.32615700
C	-3.21611300	2.33045300	0.23992300
C	-4.13272900	1.45259400	-0.61490000
C	-5.51737500	1.21513900	-0.00796500
C	-6.41009900	0.32319200	-0.87217500
H	-6.56574400	0.76295100	-1.86102800
H	-5.96332700	-0.66625500	-1.02272500
H	-7.39174700	0.17520400	-0.41650700
H	-6.00800400	2.18134300	0.14915400
H	-5.41076900	0.77366800	0.99197600
H	-4.24496900	1.90504100	-1.60680700
H	-3.63945500	0.48544100	-0.79805400
H	-3.66107900	3.32671500	0.33938500
H	-3.16502300	1.93148500	1.26278800

H	-1.38610700	1.44574100	-0.46533000
H	-1.84925500	2.90186200	-1.32532200
H	-1.32444100	4.25189200	0.75497500
H	-0.75425900	2.78832100	1.53214000
H	1.10774400	4.16552500	0.60803500
H	0.42622100	4.04545100	-1.00126800
H	1.41548900	1.67653600	0.66643400
H	0.75474100	1.56597800	-0.95202200
H	2.65580200	2.86457800	-1.90343300
H	4.74793500	2.61590300	-0.88730000
H	4.55966000	2.70936600	1.67490200
H	3.25672900	1.54720500	1.59949100
H	5.40712200	0.45366300	2.16034400
H	6.16840200	1.13612300	0.73663500
H	4.69041700	-0.19226700	-0.73943200
H	5.68427400	-1.19695500	0.29985200
H	2.73505800	-0.54797400	0.80304700
H	3.74892900	-1.49089700	1.86922500
N	-2.30965100	-2.15032700	0.97734000
C	-1.70585000	-3.31048400	1.69820900
H	-1.24814800	-3.97261100	0.96602900
H	-2.48566200	-3.83481000	2.24949100
H	-0.94781700	-2.93961000	2.38591900
C	-3.31845700	-2.59428900	-0.03088800
H	-4.12998600	-3.11157000	0.47997100
H	-3.70399200	-1.71888500	-0.54934300
H	-2.83003300	-3.26132500	-0.73866400
C	-2.87114400	-1.13795100	1.92114200
H	-3.25094600	-0.29695900	1.34725800
H	-3.67593300	-1.59433900	2.49674700
H	-2.07803200	-0.80163700	2.58648800
H	-1.52555300	-1.69252000	0.45057000

conf_123_2

C	-2.42058500	-1.46309600	1.17834800
C	-1.24569300	-2.36225200	1.57636000
C	-0.59968600	-1.98828900	2.91990800
C	0.12552800	-0.63672800	2.94746100
C	1.38196600	-0.51161200	2.12221200
O	1.89103500	0.56264500	1.85471700
O	1.99296300	-1.62254900	1.68490200
H	1.49350000	-2.41468300	1.93067200
H	-0.52683700	0.18024600	2.63355900
H	0.43044300	-0.39126800	3.97171800
H	0.08150100	-2.78504500	3.25186000
H	-1.37598800	-1.95786200	3.68933600
H	-1.59971200	-3.39577600	1.64425600
H	-0.49772900	-2.35720700	0.77349300
C	-3.05448700	-1.88786300	-0.15078100
C	-4.36672700	-1.17967100	-0.51202200
C	-4.26145800	0.32005200	-0.83748600
C	-4.07495300	1.21811700	0.35847900
C	-3.44909900	2.39683200	0.39812400

C	-2.75309400	3.10102700	-0.73270800
C	-1.26221400	3.36044400	-0.44019800
C	-0.44497300	2.07724700	-0.25840100
C	-0.23947900	1.27151900	-1.54449900
C	0.36266900	-0.10963100	-1.28887700
C	0.64835200	-0.92429800	-2.54963500
C	1.12755400	-2.34846400	-2.25355600
C	1.46403800	-3.14638900	-3.51379900
H	2.26154500	-2.66363600	-4.08682000
H	1.79359900	-4.15943300	-3.27147800
H	0.59252600	-3.22838800	-4.16903700
H	0.35091700	-2.87235000	-1.68399000
H	2.00565000	-2.31152900	-1.59491900
H	-0.25852300	-0.97114100	-3.16349000
H	1.39479300	-0.40483200	-3.16599200
H	-0.32379300	-0.67107200	-0.64794800
H	1.28417800	-0.00908100	-0.70299800
H	0.39351400	1.83934800	-2.23999200
H	-1.19538700	1.14049500	-2.06053400
H	-0.95126600	1.44505300	0.47915200
H	0.52950500	2.32072700	0.17867500
H	-0.83469100	3.96293700	-1.24986200
H	-1.18454200	3.96558100	0.46970900
H	-3.24327700	4.06748900	-0.89774400
H	-2.86569700	2.54568500	-1.66620900
H	-3.44018500	2.93198600	1.34655500
H	-4.55805600	0.87490100	1.27153400
H	-5.19198300	0.61431900	-1.33957000
H	-3.46567600	0.47849500	-1.57240100
H	-4.79083700	-1.68642100	-1.38399000
H	-5.09039400	-1.32155400	0.29978700
H	-3.24759400	-2.96631100	-0.11383600
H	-2.33119600	-1.73957400	-0.96298700
H	-3.17824400	-1.50227200	1.97003700
H	-2.10444200	-0.41865300	1.11536800
N	3.93718500	1.03973400	0.13786100
C	3.49619300	2.15579800	-0.75180600
H	3.18545800	2.99554800	-0.13328100
H	2.65703900	1.81431600	-1.35358900
H	4.32655100	2.44907300	-1.39364800
C	4.30134700	-0.18085300	-0.64230500
H	5.17793400	0.03481500	-1.25246800
H	4.51379700	-0.98984200	0.05375500
H	3.46115000	-0.45704300	-1.27537900
C	5.04168100	1.46188800	1.05077200
H	4.70150100	2.30859500	1.64403800
H	5.29032000	0.62999100	1.70722600
H	5.91075800	1.74332100	0.45691900
H	3.12630600	0.79049000	0.75898800

conf_26

C	3.40602300	-2.34437900	-0.30910700
C	2.20243100	-3.12762500	-0.86228400

C	1.64434000	-2.60568400	-2.21028900
C	0.23689700	-1.95432100	-2.15331100
C	0.22981100	-0.68504300	-1.35518200
O	-0.13696500	-0.59277700	-0.18827200
O	0.71811300	0.35371500	-2.01589100
H	0.90042100	1.09931100	-1.40497500
H	-0.08711800	-1.72420200	-3.17048500
H	-0.47931100	-2.64622000	-1.70668200
H	1.56298200	-3.43344100	-2.91722700
H	2.34108800	-1.89333700	-2.65985900
H	2.49614600	-4.17290500	-0.98523200
H	1.40214200	-3.13086300	-0.11099700
C	3.06483700	-0.89800000	0.03806000
C	4.20994400	-0.01897300	0.53324800
C	3.71221900	1.40138400	0.85960200
C	3.09529100	2.10492200	-0.31869500
C	1.98565400	2.85457100	-0.31157600
C	1.12116300	3.16259800	0.89125400
C	-0.05322200	4.10113800	0.57262600
C	-1.09372000	3.53099800	-0.40025700
C	-1.86430500	2.32584400	0.14427200
C	-2.85722400	1.73706500	-0.86081700
C	-3.53727400	0.45957200	-0.36475900
C	-4.55749000	-0.12644200	-1.34303700
C	-5.15706000	-1.44819300	-0.86041800
H	-5.65011700	-1.32904800	0.10989600
H	-5.90175300	-1.83204400	-1.56090600
H	-4.38378800	-2.21800900	-0.75522700
H	-4.07819900	-0.27451500	-2.31750400
H	-5.35801900	0.60245300	-1.50640700
H	-2.76122300	-0.29265400	-0.16987500
H	-4.03539300	0.65875800	0.59448600
H	-2.33360600	1.51939800	-1.80000800
H	-3.61955600	2.48480500	-1.10673600
H	-2.39875800	2.62414500	1.05596100
H	-1.15874800	1.54252300	0.43565500
H	-0.60911400	3.25043800	-1.34392500
H	-1.80360100	4.32256500	-0.66128100
H	-0.55140300	4.37489100	1.50894500
H	0.34975300	5.03208400	0.16043900
H	1.73890800	3.62762800	1.66717900
H	0.74850100	2.23046300	1.33395500
H	1.71972100	3.36370800	-1.23649600
H	3.64736900	2.02193800	-1.25373100
H	4.55899600	2.00337900	1.21269900
H	3.00111100	1.36004700	1.69104300
H	4.67549900	-0.45416500	1.42344000
H	4.99242800	0.03384800	-0.23226800
H	2.26273200	-0.88524900	0.78600400
H	2.65750300	-0.41632500	-0.85124700
H	3.79308000	-2.86143500	0.57592900
H	4.21366200	-2.36418200	-1.04981300
N	-1.08175800	-1.65425100	2.08511600
C	-1.46504500	-0.41206600	2.82383500
H	-0.60408900	0.25240600	2.85785300

H	-1.77630300	-0.68076900	3.83292200
H	-2.27832700	0.07382300	2.29063600
C	0.08282000	-2.33714300	2.72385100
H	0.92019400	-1.64286400	2.75724400
H	-0.19157300	-2.64551700	3.73214400
H	0.35291800	-3.20645100	2.12724600
C	-2.24423800	-2.57711000	1.91711200
H	-2.58111200	-2.90903900	2.89872500
H	-3.04357700	-2.04554400	1.40522900
H	-1.93016500	-3.43391300	1.32326700
H	-0.76827500	-1.35234700	1.13270600

conf_122

C	-3.12188100	-2.41852600	0.29031200
C	-3.90423400	-1.22887800	0.85517000
C	-4.71532800	-0.45805800	-0.19718300
C	-3.87033200	0.28181300	-1.24555700
C	-2.94997300	1.35366500	-0.71953300
O	-1.91952300	1.68484100	-1.27719100
O	-3.28851100	1.99906200	0.40966200
H	-4.11228500	1.64644300	0.77676100
H	-3.24439700	-0.40297700	-1.81784200
H	-4.52338700	0.77938200	-1.97322500
H	-5.40285500	0.24457100	0.29574800
H	-5.37052700	-1.15006200	-0.73329700
H	-4.59221300	-1.58859600	1.62716200
H	-3.21715800	-0.54442900	1.36495300
C	-2.30981400	-3.17772300	1.35176000
C	-1.20822200	-2.38689500	2.07581400
C	0.01942500	-2.01165800	1.22349800
C	-0.23714700	-0.95012200	0.19033100
C	0.05266800	-0.97874300	-1.11401100
C	0.70567500	-2.09042300	-1.88302400
C	1.92633000	-1.62482700	-2.70080400
C	3.03794000	-0.97175900	-1.87092400
C	3.59302600	-1.85746900	-0.75301700
C	4.79567700	-1.25163900	-0.01650800
C	4.53939200	0.10465700	0.65616000
C	3.38177800	0.10449300	1.65957900
C	3.29484900	1.40361200	2.46315800
H	2.44138300	1.40328300	3.14775700
H	4.19571700	1.55390800	3.06347800
H	3.20385000	2.27397600	1.80391600
H	3.50222300	-0.73796700	2.35021500
H	2.43723500	-0.07316200	1.13325300
H	5.45526200	0.41282200	1.17237300
H	4.35720700	0.87297700	-0.10594500
H	5.62864400	-1.14226000	-0.71972100
H	5.13282600	-1.96230900	0.74620800
H	3.89383800	-2.82142500	-1.17881700
H	2.80107500	-2.08116500	-0.03195100
H	2.66008900	-0.03882200	-1.43949000
H	3.85526500	-0.68456900	-2.54216300

H	2.33254300	-2.48960600	-3.23533800
H	1.59186700	-0.91927200	-3.47026700
H	-0.02971500	-2.50732900	-2.58297900
H	0.99229300	-2.90763500	-1.22021500
H	-0.23884400	-0.11724200	-1.71215800
H	-0.74528000	-0.06468900	0.57307600
H	0.42326100	-2.91351100	0.75802400
H	0.79771000	-1.64812500	1.90665000
H	-1.62415200	-1.48053000	2.53161500
H	-0.85503900	-2.99762000	2.91188200
H	-1.85513400	-4.05573000	0.88011700
H	-3.00284400	-3.56719800	2.10609100
H	-3.83193300	-3.11680200	-0.16677900
H	-2.45564200	-2.08670700	-0.50799400
N	0.11602800	3.09829300	-0.14346800
C	0.06030100	2.88821700	1.33450500
H	0.84242100	3.47849800	1.80917300
H	0.22007200	1.83235800	1.54093900
H	-0.92081200	3.19491400	1.69206400
C	-0.09127600	4.53152800	-0.50633100
H	-1.04399700	4.86272000	-0.09674800
H	0.72205200	5.12672000	-0.09246800
H	-0.10565300	4.62056000	-1.59109000
C	1.37226500	2.54464900	-0.73399000
H	2.22618500	3.08540400	-0.32861400
H	1.43748300	1.48908500	-0.48054000
H	1.33150100	2.66152300	-1.81538900
H	-0.68477300	2.56582100	-0.56034400

conf_260_2

C	2.42914900	-0.55602600	1.37499100
C	1.87353800	-1.33924600	2.56915700
C	0.35943600	-1.24734600	2.78330000
C	-0.13158200	0.14513400	3.21147300
C	-0.21277600	1.13527100	2.08207400
O	-0.61373800	0.86071000	0.96940900
O	0.13482600	2.41776300	2.32857800
H	0.45057100	2.52691000	3.23571000
H	-1.15506400	0.07113800	3.59918000
H	0.48606600	0.53801500	4.02563200
H	0.06766300	-1.95007600	3.56734100
H	-0.17604900	-1.54579000	1.87963300
H	2.12916900	-2.39533400	2.43523900
H	2.38843600	-1.02300000	3.48524600
C	3.94261000	-0.74020700	1.23261800
C	4.56165200	-0.12291900	-0.02633900
C	4.11323600	-0.80414800	-1.34465900
C	3.08259300	-0.04703300	-2.13267700
C	1.87317600	-0.46026000	-2.52284400
C	1.18538600	-1.75832900	-2.20897100
C	-0.26009400	-1.54562100	-1.73451400
C	-1.01188900	-2.84894200	-1.44787100
C	-2.47882400	-2.63111000	-1.05323500

C	-2.66076000	-2.03691200	0.34808200
C	-4.11145900	-1.68091000	0.70271800
C	-4.73899700	-0.57465800	-0.15886100
C	-3.97897700	0.75459700	-0.10679500
H	-4.48684000	1.51894000	-0.70289200
H	-3.90374700	1.12505500	0.92098600
H	-2.96045800	0.63742500	-0.48604200
H	-5.76762600	-0.41123400	0.17639900
H	-4.81338500	-0.90785000	-1.19874500
H	-4.73319800	-2.57977500	0.62840000
H	-4.15146200	-1.37087500	1.75423200
H	-2.03387200	-1.14894000	0.46094100
H	-2.29037800	-2.76315400	1.08128500
H	-3.01577100	-3.58421200	-1.10448500
H	-2.94679100	-1.98205100	-1.80270700
H	-0.49479100	-3.40416800	-0.65520100
H	-0.96784800	-3.48167300	-2.34084800
H	-0.25481200	-0.90912200	-0.84416200
H	-0.80977500	-0.99564000	-2.51060400
H	1.74138400	-2.32182100	-1.45530400
H	1.17080300	-2.38592500	-3.10927500
H	1.30717500	0.20620200	-3.17437500
H	3.41133100	0.93546600	-2.47148200
H	3.77345800	-1.82117500	-1.13467100
H	4.98828100	-0.91336200	-1.99491800
H	4.33660800	0.94994000	-0.06620800
H	5.64846200	-0.19813500	0.06152300
H	4.17227300	-1.81253500	1.24188700
H	4.43463700	-0.32082200	2.11767000
H	1.91751200	-0.87615200	0.46302200
H	2.22119100	0.51727600	1.47835700
N	-0.57709700	2.72616000	-1.02046400
C	0.88549700	3.02901800	-1.08885700
H	1.05200300	3.81978100	-1.81969000
H	1.41340800	2.12139600	-1.37870400
H	1.21546300	3.35020200	-0.10287000
C	-1.36644400	3.88546700	-0.50820600
H	-2.41191600	3.59040800	-0.43653700
H	-0.98890700	4.15197300	0.47690500
H	-1.25855500	4.72344600	-1.19608100
C	-1.10193000	2.22951100	-2.32672300
H	-0.53795000	1.34563400	-2.61294200
H	-2.15278300	1.97442700	-2.20888200
H	-0.98546600	3.01235000	-3.07573700
H	-0.67274200	1.96394700	-0.31003600

conf_249_2

C	-4.39509700	0.10810900	-0.55396600
C	-5.15756000	-0.75019400	-1.56683900
C	-4.98083400	-2.26473200	-1.40361800
C	-3.52487300	-2.75754900	-1.51180700
C	-2.62854300	-2.44752400	-0.34246600
O	-1.47745600	-2.06572500	-0.44231700

O	-3.11729000	-2.64805700	0.89731200
H	-4.04789500	-2.91199500	0.86536600
H	-3.51383900	-3.85023500	-1.61247300
H	-3.03970500	-2.35357900	-2.40100100
H	-5.55502300	-2.77813300	-2.17822900
H	-5.43432900	-2.59188600	-0.45642600
H	-6.22660400	-0.52391000	-1.50167500
H	-4.84863800	-0.46616300	-2.57925100
C	-4.74467200	1.59748900	-0.64107800
C	-3.83995400	2.48307300	0.22405900
C	-2.44322100	2.70096500	-0.37331300
C	-1.54157000	3.51520600	0.51786100
C	-0.20695800	3.55616200	0.47766000
C	0.68733200	2.79168100	-0.45858700
C	2.00846500	2.35912400	0.19039200
C	2.88324800	1.50443200	-0.72747100
C	4.17121700	1.01381800	-0.06349300
C	5.02461200	0.12382000	-0.96926600
C	6.30687200	-0.37458100	-0.29990600
C	7.16144500	-1.26461100	-1.20580300
C	8.43985100	-1.75787400	-0.52620900
H	9.07103800	-0.91891600	-0.21853500
H	8.21037600	-2.34466000	0.36870300
H	9.02965900	-2.38906000	-1.19522300
H	7.41979900	-0.70864400	-2.11442900
H	6.56448900	-2.12401800	-1.53444400
H	6.90284900	0.48641000	0.02698200
H	6.05021400	-0.92892200	0.61276100
H	5.28225300	0.67827000	-1.87951200
H	4.42890800	-0.73763800	-1.29961900
H	3.92503500	0.46346700	0.85555400
H	4.76222900	1.87856800	0.25988200
H	3.13320500	2.07933200	-1.62639400
H	2.30598300	0.64093000	-1.08902800
H	2.56564800	3.24975900	0.50067200
H	1.79917000	1.81050600	1.11803300
H	0.91559000	3.41771500	-1.33126300
H	0.16553900	1.91662600	-0.86589700
H	0.30218200	4.22920000	1.16438300
H	-2.04674400	4.16088300	1.23298700
H	-2.55746600	3.21314400	-1.33784600
H	-1.97524500	1.73929500	-0.61444200
H	-4.31229600	3.46019700	0.36381300
H	-3.75065500	2.04647700	1.22768000
H	-5.78867100	1.73099400	-0.34078500
H	-4.68212900	1.92813100	-1.68467600
H	-4.60794700	-0.23965800	0.46546000
H	-3.31785500	-0.01388800	-0.70950300
N	-0.14546200	-1.16972800	1.77288800
C	-0.44623700	-2.05406700	2.93742300
H	0.03356100	-1.64712100	3.82691500
H	-0.06338100	-3.05184500	2.73013600
H	-1.52475100	-2.09731100	3.07166300
C	-0.69484000	0.20755000	1.96763800
H	-0.49149500	0.80418700	1.08332100

H	-1.76948500	0.13501700	2.12373900
H	-0.21964400	0.66438000	2.83446000
C	1.31285400	-1.14891600	1.45030800
H	1.64736400	-2.16907600	1.27067600
H	1.46773300	-0.54701800	0.55846000
H	1.85863800	-0.71795200	2.28861000
H	-0.64069300	-1.57178900	0.94111200

conf_152

C	3.39385800	-0.96130800	-1.75445700
C	2.19665500	-1.81701700	-2.19432100
C	1.49055300	-2.47529200	-0.99593400
C	0.05760900	-2.94627000	-1.26371300
C	-1.01581300	-1.88824300	-1.20410800
O	-2.20231000	-2.15824100	-1.11413600
O	-0.68018300	-0.59109600	-1.24163900
H	0.27746100	-0.44902600	-1.28951500
H	-0.02585800	-3.41256300	-2.25285600
H	-0.25414200	-3.71371500	-0.55169900
H	1.49259000	-1.80301500	-0.13306400
H	2.07933900	-3.33807700	-0.67754200
H	1.50150700	-1.19632000	-2.77605200
H	2.51703600	-2.60214600	-2.88475300
C	2.98501300	0.43164300	-1.24849100
C	2.70900200	1.42410200	-2.38313600
C	2.14109300	2.76988600	-1.90340300
C	0.69256700	2.70238800	-1.50812800
C	0.13716600	3.11495000	-0.36560500
C	0.82278700	3.73777200	0.82111500
C	0.18005100	3.42285900	2.18390300
C	0.38051100	1.98991400	2.69573300
C	-0.41344200	0.92478200	1.93343200
C	-0.28013300	-0.49554600	2.49434200
C	1.16306100	-1.00843100	2.54750500
C	1.26697500	-2.52038700	2.75536400
C	2.71153400	-3.02082200	2.78515900
H	3.27127700	-2.55660300	3.60175400
H	2.75841200	-4.10331900	2.92465800
H	3.23213600	-2.78064200	1.85258000
H	0.71513400	-3.03372900	1.95713700
H	0.76257200	-2.79225800	3.68931900
H	1.68228900	-0.73171800	1.62039800
H	1.70768600	-0.49856300	3.34820900
H	-0.87398800	-1.18070800	1.87280000
H	-0.71749800	-0.54472500	3.49858200
H	-0.10366000	0.92284900	0.88555200
H	-1.47190600	1.22060000	1.93959400
H	0.09660500	1.94916100	3.75378500
H	1.44846000	1.75000000	2.65874700
H	0.60341700	4.11217500	2.91991900
H	-0.89285100	3.65208100	2.14010100
H	0.80506200	4.82715200	0.68774400
H	1.88021300	3.45792900	0.84552800

H	-0.94711800	3.05264000	-0.28261900
H	0.02943100	2.31261800	-2.28065900
H	2.23213400	3.49472100	-2.72138500
H	2.75436400	3.15817900	-1.08615600
H	3.64506100	1.59861100	-2.92245900
H	2.01853500	0.98686900	-3.11572800
H	3.77608700	0.84042200	-0.61218900
H	2.10627600	0.35963700	-0.58989200
H	3.94376600	-1.49921100	-0.97533600
H	4.09057500	-0.84136300	-2.58919400
N	-3.97318200	-0.19517000	-0.50096200
C	-4.14719600	-0.37664600	0.97153500
H	-3.18572700	-0.22495300	1.45771800
H	-4.50055000	-1.38862500	1.16050700
H	-4.87211300	0.34925600	1.33887100
C	-5.23756500	-0.46584500	-1.24715400
H	-5.57223900	-1.47578700	-1.01719700
H	-5.99443400	0.25793800	-0.94590000
H	-5.04159900	-0.37848700	-2.31432100
C	-3.40510600	1.14723900	-0.82795700
H	-2.44431800	1.24917100	-0.32991500
H	-3.26342600	1.21539300	-1.90478000
H	-4.09759000	1.91816300	-0.49098400
H	-3.27125300	-0.91750100	-0.80646100

conf_110

C	1.85696800	3.22090300	0.43843200
C	0.82756200	4.35659800	0.39164000
C	-0.51026000	4.00395300	-0.27266600
C	-1.33801200	2.93778200	0.50719400
C	-1.53964100	1.65811700	-0.25021600
O	-2.63185900	1.22561400	-0.57816600
O	-0.45703300	0.94316100	-0.59331000
H	0.36487100	1.35580800	-0.28556600
H	-0.83807400	2.68937400	1.45005900
H	-2.32857700	3.31123100	0.75820400
H	-0.34382200	3.67779200	-1.30342400
H	-1.11760800	4.90719900	-0.34599200
H	0.62933100	4.70587800	1.41053900
H	1.26477600	5.20578100	-0.14286300
C	2.40153100	2.79915200	-0.93413900
C	3.53993800	1.77413900	-0.86921000
C	3.15313900	0.42533700	-0.24115500
C	4.30618000	-0.53948300	-0.23141400
C	4.30268100	-1.81920000	-0.60866100
C	3.13721800	-2.63720100	-1.08739900
C	2.66032500	-3.63710600	-0.01068300
C	2.02120100	-2.96956000	1.21307500
C	0.61204300	-2.43057200	0.95196000
C	0.04302200	-1.60854300	2.11050600
C	-1.43919400	-1.27218100	1.93704800
C	-1.98973000	-0.29220600	2.97487400
C	-3.47802500	0.00974300	2.78855000

H	-3.84275200	0.70832000	3.54491600
H	-3.67355100	0.45237700	1.80682300
H	-4.07587800	-0.90327000	2.87336100
H	-1.41443200	0.64103000	2.92781700
H	-1.81817200	-0.69648000	3.97793500
H	-1.59215000	-0.85130800	0.93637300
H	-2.02101400	-2.20296300	1.97831500
H	0.62163300	-0.68130500	2.20976100
H	0.18188800	-2.15115800	3.05223500
H	-0.05436100	-3.27986400	0.74622600
H	0.61160000	-1.80869900	0.04849100
H	2.66891700	-2.15652700	1.55859300
H	1.97146400	-3.69001800	2.03593400
H	1.94823300	-4.33960400	-0.45977700
H	3.51852400	-4.23582700	0.30999500
H	3.44027100	-3.20131700	-1.97578300
H	2.30651200	-1.99622500	-1.39642600
H	5.23897500	-2.36667600	-0.52300500
H	5.24378400	-0.12085200	0.12878400
H	2.30009100	-0.00368900	-0.77837600
H	2.82498800	0.58546700	0.79475700
H	3.91603200	1.59419400	-1.88071600
H	4.37548000	2.20235200	-0.30406400
H	2.76233600	3.69687300	-1.44678400
H	1.60130900	2.40686600	-1.57632500
H	2.70061200	3.53988100	1.05880000
H	1.43810300	2.36014400	0.98328000
N	-3.19847200	-1.16568700	-1.72597700
C	-1.97803800	-1.98665900	-1.98511600
H	-2.26827800	-2.90449600	-2.49570300
H	-1.50019500	-2.21719000	-1.03610200
H	-1.29423500	-1.41080700	-2.60572400
C	-3.87771900	-0.76514000	-2.99432300
H	-4.23009500	-1.65784800	-3.51018400
H	-4.71650100	-0.11501400	-2.75268000
H	-3.16599300	-0.22880200	-3.61934700
C	-4.13840900	-1.84794200	-0.78618400
H	-3.60915800	-2.06767300	0.13796400
H	-4.97321000	-1.18062200	-0.58037000
H	-4.49694600	-2.76850100	-1.24601700
H	-2.89096000	-0.27379900	-1.26294700

9E_NMe3H

conf_0

C	4.40046300	-1.22473200	1.60326300
C	2.92603200	-1.42099400	1.98519100
C	2.16583700	-0.10526700	2.19097200
C	0.65914100	-0.29984100	2.38295600
C	-0.05958600	-0.91904200	1.21466300
O	-1.02440500	-1.65580600	1.33672100
O	0.34071800	-0.62718600	-0.03262300
H	1.12972400	-0.05442000	-0.07737400
H	0.43625500	-0.92388500	3.24967100

H	0.16606500	0.66507500	2.55669400
H	2.55766900	0.41517400	3.06909800
H	2.34372100	0.57848900	1.35446900
H	2.42717000	-2.01922800	1.21350900
H	2.86472800	-2.01311800	2.90381200
C	4.61392800	-0.45155000	0.29475400
C	4.10951800	-1.16561900	-0.96795200
C	3.84053100	-0.18175200	-2.12753700
C	2.52393600	0.53456700	-1.98820800
C	2.35188900	1.77538000	-1.52242800
C	1.04655300	2.51190000	-1.42235400
C	0.78131700	3.07988700	-0.01851700
C	-0.42814100	4.02039500	0.05980800
C	-1.77373500	3.40177300	-0.34139500
C	-2.19065600	2.19663900	0.50574600
C	-3.60012100	1.69382500	0.18732300
C	-4.02938500	0.49486900	1.03671900
C	-5.41184700	-0.04401800	0.66401100
H	-5.69492000	-0.89803900	1.28550500
H	-6.17960900	0.72264300	0.79511000
H	-5.45454000	-0.35680500	-0.38587100
H	-3.27686200	-0.30005100	0.95700600
H	-4.02570700	0.78436700	2.09296900
H	-4.31804200	2.50931300	0.32966000
H	-3.65983700	1.43637000	-0.87949700
H	-1.47936400	1.37629100	0.35494600
H	-2.13477000	2.46241100	1.56901100
H	-2.54502500	4.17479100	-0.25974200
H	-1.75449200	3.11233300	-1.39872800
H	-0.23574900	4.89080400	-0.57670700
H	-0.50583600	4.40386600	1.08325300
H	0.65807000	2.25538600	0.69307800
H	1.67032500	3.62633300	0.31367800
H	0.22554000	1.86021500	-1.73871500
H	1.06426900	3.34914600	-2.13209400
H	3.23315400	2.33916300	-1.21707400
H	1.64579800	-0.02037200	-2.32200500
H	4.65759200	0.54417600	-2.18075900
H	3.84389500	-0.72680100	-3.07639700
H	3.18850200	-1.72254300	-0.76032400
H	4.84594100	-1.91179500	-1.28036400
H	5.68145000	-0.24448300	0.17388300
H	4.13673300	0.53015800	0.37107500
H	4.87924300	-2.20692100	1.53159900
H	4.91009900	-0.69628900	2.41615200
N	-2.20007400	-2.53808400	-0.94361000
C	-2.41606400	-1.37985400	-1.86061500
H	-2.80255200	-1.74460600	-2.81187200
H	-3.12867000	-0.69875000	-1.40228600
H	-1.46448000	-0.87262800	-2.00359000
C	-3.48233900	-3.21318600	-0.58367700
H	-4.15062300	-2.48021200	-0.13692100
H	-3.93225500	-3.63152900	-1.48377500
H	-3.27213800	-4.00550300	0.13253900
C	-1.19774800	-3.49595800	-1.49672300

H	-0.26648400	-2.95937500	-1.66796800
H	-1.57362900	-3.91055400	-2.43161900
H	-1.03641800	-4.29249500	-0.77250900
H	-1.78107400	-2.16010500	-0.05403100

conf_30

C	-3.67167300	-2.29354800	-0.13126700
C	-2.26260700	-2.89271200	-0.20275100
C	-1.19302200	-1.88429200	-0.63750600
C	0.19556700	-2.53490200	-0.72552400
C	1.30929500	-1.61770900	-1.14696500
O	2.42599300	-1.63360500	-0.66267300
O	1.06578500	-0.73511500	-2.13409900
H	0.13601900	-0.76395000	-2.40347400
H	0.48809900	-2.96744900	0.23164200
H	0.17587000	-3.35890200	-1.45089600
H	-1.49571300	-1.47464600	-1.61231400
H	-1.15746600	-1.04127600	0.05638600
H	-1.98870900	-3.30789500	0.77424400
H	-2.25825000	-3.73162200	-0.90749800
C	-3.87861300	-1.31944100	1.03332600
C	-5.28363000	-0.70708600	1.08086500
C	-5.59958200	0.23953700	-0.09538400
C	-4.70811400	1.44614300	-0.12609100
C	-3.82351000	1.73528800	-1.07943600
C	-2.92754700	2.94117600	-1.09480700
C	-1.43636400	2.59468700	-0.92637100
C	-1.10003600	2.01932300	0.44997900
C	0.38075900	1.68004900	0.62358400
C	0.70375600	0.99880500	1.95522200
C	2.18518000	0.65582100	2.12274200
C	2.52249700	-0.01866600	3.45427100
C	3.99992900	-0.39558700	3.57387400
H	4.64200500	0.48738000	3.48536300
H	4.21883600	-0.86150400	4.53696200
H	4.28889200	-1.10779400	2.79338200
H	2.24428000	0.64850600	4.27690900
H	1.90554800	-0.91718400	3.56740100
H	2.49067300	-0.01022400	1.30557000
H	2.78020600	1.57550000	2.02833300
H	0.38490200	1.64579800	2.78006800
H	0.10994300	0.08048900	2.04761400
H	0.69914000	1.03315800	-0.20367600
H	0.97312000	2.60018200	0.53283600
H	-1.70509200	1.12392600	0.62421900
H	-1.40053100	2.73532200	1.22378900
H	-1.14235900	1.87700800	-1.70548800
H	-0.83872600	3.49672100	-1.10094600
H	-3.22728200	3.63157400	-0.29950900
H	-3.05806600	3.47817800	-2.04119400
H	-3.72684300	1.05528100	-1.92728500
H	-4.80070500	2.12714400	0.72046300
H	-5.53008600	-0.29655500	-1.04653500

H	-6.64183300	0.56437400	-0.00150700
H	-5.39876100	-0.15360300	2.01891500
H	-6.03062000	-1.50802900	1.10325300
H	-3.14551700	-0.50830600	0.98218800
H	-3.68828600	-1.85120500	1.97253100
H	-4.39556900	-3.10990500	-0.03947900
H	-3.89375400	-1.79838200	-1.08300600
N	4.32299100	0.22977200	-1.27686600
C	4.86665600	-0.19951600	-2.59987000
H	5.64652600	0.49535100	-2.90973300
H	5.27685000	-1.20308100	-2.50243500
H	4.05523100	-0.20141400	-3.32542400
C	5.35983000	0.17639000	-0.20241300
H	5.75663000	-0.83574700	-0.14807600
H	6.15734900	0.87940900	-0.44142200
H	4.89564400	0.43909200	0.74544000
C	3.67372700	1.57292700	-1.35737300
H	4.41804400	2.31000300	-1.65724900
H	3.26577400	1.82253100	-0.38107300
H	2.86895800	1.52607200	-2.08816700
H	3.57547500	-0.46308800	-1.02750900

conf_1

C	2.39084200	2.55906000	-0.36311900
C	0.88250900	2.57065800	-0.63812100
C	0.51755900	2.56751000	-2.12813400
C	-0.95878900	2.24450000	-2.38155300
C	-1.39083900	0.85797100	-1.97389900
O	-2.51935100	0.58819100	-1.60283700
O	-0.50148900	-0.14427700	-2.05797400
H	0.37664700	0.18373200	-2.30326000
H	-1.61742500	2.94178900	-1.86110000
H	-1.19662600	2.33031300	-3.44924400
H	0.73628900	3.54496200	-2.56526100
H	1.15761100	1.87053700	-2.68588200
H	0.43783000	1.69766700	-0.14493200
H	0.42075300	3.44574800	-0.16991300
C	3.06389400	1.24072200	-0.75841200
C	4.52015100	1.12361900	-0.30182500
C	5.10372400	-0.28837700	-0.52084800
C	4.45560800	-1.30428600	0.37482600
C	3.59448100	-2.24233000	-0.01631400
C	2.84302300	-3.17063100	0.89473900
C	1.35718700	-2.76885200	1.01644900
C	1.15186000	-1.46886800	1.79819100
C	-0.26206600	-0.89790500	1.68923000
C	-0.43792600	0.44652000	2.39842300
C	-1.81356600	1.07824700	2.17808500
C	-1.94605900	2.48735200	2.75983400
C	-3.32423700	3.10441600	2.51818400
H	-4.11211800	2.50398100	2.98344400
H	-3.39022400	4.11242300	2.93364400
H	-3.54499100	3.17072400	1.44774800

H	-1.73843100	2.45572300	3.83494800
H	-1.17266900	3.12811800	2.31998500
H	-2.02969900	1.11748000	1.10176700
H	-2.58585500	0.43618200	2.62314200
H	-0.25780000	0.32184700	3.47183600
H	0.33592900	1.13728400	2.04335600
H	-0.50555300	-0.76961900	0.62545700
H	-0.98259700	-1.62253900	2.09443800
H	1.86068600	-0.71830400	1.43577000
H	1.40128800	-1.63632600	2.85209600
H	0.94303100	-2.65573700	0.00475500
H	0.79795500	-3.58093000	1.49532900
H	3.30032100	-3.17211200	1.88940900
H	2.90606200	-4.19580500	0.51505400
H	3.36333900	-2.32802000	-1.07897700
H	4.68386700	-1.21560400	1.43706600
H	4.97770300	-0.57905800	-1.56965600
H	6.18090800	-0.25793600	-0.32778600
H	4.58836900	1.37776500	0.76251100
H	5.13346800	1.85752400	-0.83444700
H	3.03452700	1.10511100	-1.84720000
H	2.49192600	0.41133100	-0.32695300
H	2.54778700	2.72599800	0.70749200
H	2.87142800	3.40048500	-0.87541700
N	-3.44537900	-1.85342900	-0.84056200
C	-2.39142500	-2.91091900	-0.89296000
H	-2.84079900	-3.87272800	-0.64743500
H	-1.97139300	-2.93689500	-1.89650700
H	-1.61096500	-2.66513700	-0.17704900
C	-4.52775700	-2.09904300	-1.84005300
H	-5.03582300	-3.03153400	-1.59617600
H	-5.23037900	-1.26847900	-1.80735500
H	-4.08238500	-2.16443300	-2.83111900
C	-3.99373700	-1.68309200	0.53858800
H	-4.48927200	-2.60511300	0.84151200
H	-4.70374900	-0.85818800	0.53355500
H	-3.17315000	-1.45451600	1.21396400
H	-2.99805100	-0.94265900	-1.11005100

conf_2

C	4.58311500	-1.00368200	1.88172700
C	3.10148800	-1.37204700	2.04914500
C	2.19198300	-0.15442300	2.25657700
C	0.70413100	-0.50032700	2.32971400
C	0.06614900	-1.01487300	1.06584500
O	-0.98503600	-1.63454100	1.06229800
O	0.63434400	-0.75150500	-0.11934400
H	1.45343900	-0.22756200	-0.05432200
H	0.49828900	-1.24431500	3.10261000
H	0.11545400	0.38440400	2.60051100
H	2.46798100	0.34805300	3.18786300
H	2.35984700	0.59571800	1.47477500
H	2.76460600	-1.94879300	1.17971300

H	2.99041100	-2.04105700	2.90861300
C	4.88259500	-0.15117700	0.64106600
C	4.70065300	-0.88452600	-0.69642200
C	4.40959000	0.08234300	-1.86306900
C	2.98621800	0.56517800	-1.86016700
C	2.57056900	1.79238400	-1.53462900
C	1.14683000	2.27119300	-1.56551900
C	0.74075500	3.07743900	-0.31867300
C	-0.77423200	3.31053000	-0.22780600
C	-1.51876900	2.09815100	0.34016700
C	-3.03902100	2.14765300	0.18689100
C	-3.74775900	1.01336400	0.93316700
C	-5.23531900	0.88368300	0.59888300
C	-5.94619800	-0.19122500	1.42310500
H	-5.90257200	0.04148400	2.49046400
H	-6.99875900	-0.27924700	1.14506200
H	-5.48330800	-1.17543600	1.29006500
H	-5.72557900	1.84947500	0.76066400
H	-5.35081900	0.67095800	-0.47253800
H	-3.23965800	0.06045200	0.72717000
H	-3.63031400	1.16669200	2.01228500
H	-3.41857900	3.10964100	0.54819400
H	-3.29552800	2.11311800	-0.88083100
H	-1.14820800	1.18710800	-0.14197600
H	-1.26861000	2.00165600	1.40426200
H	-1.16304500	3.55407300	-1.22411000
H	-0.98298500	4.18159300	0.40026300
H	1.08095800	2.56038900	0.58794100
H	1.27166700	4.03369300	-0.33330500
H	0.47674300	1.41591400	-1.70384100
H	1.00354800	2.91378300	-2.44339200
H	3.31609700	2.53428300	-1.24962300
H	2.24326600	-0.16962800	-2.17610200
H	5.09552600	0.93361400	-1.81357300
H	4.60635700	-0.42603900	-2.81265100
H	3.88206200	-1.61126700	-0.63361400
H	5.59931700	-1.46799200	-0.91581900
H	5.91075600	0.21766400	0.70074300
H	4.25310400	0.74428000	0.65244000
H	5.17417600	-1.92459400	1.84338200
H	4.91492800	-0.46599500	2.77655700
N	-2.20779200	-2.10747200	-1.31436500
C	-3.59972800	-2.57440000	-1.04231600
H	-4.07669200	-2.84623500	-1.98367200
H	-3.55736300	-3.43762600	-0.38061300
H	-4.15160500	-1.76973000	-0.56123700
C	-1.36701100	-3.17884200	-1.92648400
H	-0.35035400	-2.80369400	-2.02740200
H	-1.37501800	-4.04809700	-1.27135300
H	-1.77305900	-3.44142000	-2.90287500
C	-2.19105000	-0.86072600	-2.13567600
H	-2.78440100	-0.10079100	-1.63250100
H	-2.61096700	-1.07539600	-3.11799700
H	-1.16148600	-0.52216600	-2.22899600
H	-1.75948200	-1.88430000	-0.38747700

conf_50

C	4.94463100	-0.72306000	1.25872700
C	3.91804900	-1.86744100	1.15892900
C	2.77982800	-1.75267800	2.18072300
C	1.76308500	-0.63781700	1.83771900
C	0.92433700	-1.02052900	0.64852400
O	0.10047700	-1.92328500	0.69009700
O	1.08699200	-0.37428300	-0.51022800
H	1.67100200	0.41637500	-0.43806200
H	1.07414400	-0.50279700	2.67464900
H	2.26993000	0.31201200	1.65985400
H	2.23612200	-2.69652200	2.26531300
H	3.19104300	-1.53578500	3.17048100
H	3.49346300	-1.91300800	0.14928100
H	4.43007300	-2.82276600	1.30157900
C	5.63223500	-0.37742100	-0.06998000
C	4.69461600	0.31372000	-1.07084700
C	4.29934700	1.74973900	-0.64977700
C	2.90586800	2.12674700	-1.06403400
C	1.97514800	2.65176600	-0.25682000
C	0.58725100	3.05546200	-0.66918900
C	-0.50047100	2.53229800	0.28292200
C	-1.92168000	2.69746900	-0.25706800
C	-2.96683600	1.99547300	0.61299200
C	-4.37202500	1.99472300	0.00402800
C	-5.39466100	1.15954500	0.78461400
C	-5.14901300	-0.35278700	0.72479500
C	-6.21307100	-1.16202300	1.46877500
H	-7.20726900	-0.97692400	1.05327000
H	-6.02237300	-2.23711800	1.40810200
H	-6.24141000	-0.88996200	2.52737400
H	-5.12485100	-0.66241600	-0.32929100
H	-4.16313700	-0.58492400	1.14703100
H	-5.40808900	1.48208400	1.83256500
H	-6.39628800	1.36335800	0.39162000
H	-4.72656900	3.02798900	-0.06797500
H	-4.32217900	1.62493900	-1.02972500
H	-2.63523500	0.96461500	0.79250500
H	-3.00106400	2.46607800	1.60231700
H	-1.96603800	2.28593800	-1.27451600
H	-2.16792000	3.76033800	-0.35565100
H	-0.33201300	1.46781200	0.47376200
H	-0.40943900	3.03162900	1.25387300
H	0.39431700	2.70444000	-1.68913300
H	0.52778300	4.15009000	-0.70699200
H	2.23751700	2.83595400	0.78583500
H	2.65715600	1.97502000	-2.11562100
H	4.38986000	1.87334900	0.43295200
H	5.01013300	2.45661900	-1.09196800
H	3.79312300	-0.29637500	-1.19363300
H	5.16346400	0.34106400	-2.05807900
H	6.03238100	-1.29251300	-0.51936200

H	6.49365100	0.27047500	0.12244000
H	5.69921300	-0.99131900	2.00324100
H	4.46004000	0.17687600	1.65293500
N	-1.57518300	-2.18306100	-1.41702400
C	-2.01414200	-0.81010900	-1.80690100
H	-2.64309800	-0.87390900	-2.69441200
H	-2.57642100	-0.37443700	-0.98475300
H	-1.12927900	-0.21027000	-2.00700900
C	-2.72439200	-3.03476600	-0.98958800
H	-3.24643700	-2.53593000	-0.17547200
H	-3.39993800	-3.17578100	-1.83305800
H	-2.34214400	-3.99615800	-0.65116600
C	-0.76634100	-2.82783600	-2.49269700
H	0.09301300	-2.19428000	-2.70445000
H	-1.38067500	-2.94354800	-3.38522900
H	-0.42821500	-3.80160000	-2.14262700
H	-0.93140100	-2.07959500	-0.58765800

conf_55

C	4.47370400	0.54096000	0.17820600
C	5.70485100	-0.23176700	-0.29673200
C	5.75179000	-1.69095700	0.17468000
C	4.64537900	-2.59022900	-0.41497700
C	3.24225700	-2.33957500	0.07430400
O	2.27327800	-2.25102500	-0.65871000
O	3.03995300	-2.25224000	1.39932000
H	3.87620600	-2.29226500	1.88449300
H	4.62111300	-2.50688800	-1.50184400
H	4.86459400	-3.63895300	-0.17833000
H	6.70748300	-2.13420300	-0.11450600
H	5.74095700	-1.72748400	1.27334200
H	5.74887300	-0.21236900	-1.39203200
H	6.61001100	0.27388000	0.05358300
C	4.41170300	1.98900400	-0.31502600
C	3.17624600	2.74371400	0.19385100
C	1.84185800	2.15790300	-0.30045000
C	0.64275800	2.81476600	0.31996100
C	-0.34273300	3.42118400	-0.34234700
C	-1.52732800	4.09441500	0.28854100
C	-2.87439000	3.47591100	-0.12896700
C	-3.01962400	2.01493300	0.29698300
C	-4.35383400	1.37778600	-0.09449200
C	-4.43336700	-0.11085900	0.25267500
C	-5.77415500	-0.76433000	-0.08399900
C	-5.80637000	-2.26653700	0.20884000
C	-7.15480600	-2.91331400	-0.11177400
H	-7.14661200	-3.98453100	0.10326800
H	-7.95589400	-2.46144800	0.47984800
H	-7.41128700	-2.78629700	-1.16752200
H	-5.01765500	-2.76302800	-0.37297900
H	-5.56123800	-2.43367300	1.26488700
H	-6.56897600	-0.26667400	0.48350300
H	-6.00497900	-0.59553200	-1.14282900

H	-3.63898100	-0.64736000	-0.28777300
H	-4.22360400	-0.24817700	1.32202700
H	-5.17262900	1.90904700	0.40342300
H	-4.51964600	1.50682600	-1.17096300
H	-2.88543400	1.94157600	1.38453000
H	-2.19810500	1.44273100	-0.14713800
H	-3.68589700	4.06841700	0.30528500
H	-2.98801200	3.55078300	-1.21720200
H	-1.52715100	5.15310500	0.00507300
H	-1.42988300	4.06144500	1.37900000
H	-0.30273500	3.44802600	-1.43235700
H	0.60929800	2.80860000	1.41028300
H	1.78880000	2.23330500	-1.39258700
H	1.82742100	1.08536400	-0.06091300
H	3.23179100	3.79177200	-0.11349300
H	3.18097600	2.74562200	1.29063400
H	4.42376200	1.99954900	-1.41170900
H	5.31295400	2.52088300	0.00660000
H	4.43846100	0.53409700	1.27570900
H	3.57291600	0.02660900	-0.16412500
N	-0.31628400	-1.52139600	-0.19689700
C	-0.62660200	-0.68846800	-1.39956100
H	-0.06281700	0.23966300	-1.33724000
H	-0.33625200	-1.24522800	-2.28867000
H	-1.69308400	-0.47331800	-1.41800200
C	-0.53491600	-0.77461000	1.07994600
H	-0.00031600	0.17131100	1.03078600
H	-1.59948300	-0.58640900	1.20268900
H	-0.15720600	-1.37958000	1.90244400
C	-1.06558900	-2.81399500	-0.21195500
H	-2.13379500	-2.60687100	-0.16051600
H	-0.75769300	-3.40695000	0.64753400
H	-0.82758200	-3.34649900	-1.13094500
H	0.69846800	-1.76859900	-0.25810800

conf_125

C	-4.38867100	-1.74678700	-0.96151700
C	-2.97693500	-2.32104600	-1.15297900
C	-2.06626000	-1.45059600	-2.02517000
C	-0.64511600	-2.01574700	-2.13097200
C	0.10420700	-1.92025600	-0.82749100
O	-0.03561200	-1.00511100	-0.03788900
O	0.99998500	-2.87732100	-0.52948700
H	1.03136800	-3.55763900	-1.21590400
H	-0.65761100	-3.05178400	-2.48521900
H	-0.05475100	-1.44028700	-2.85535000
H	-2.48259100	-1.36974900	-3.03306500
H	-2.00174000	-0.43749200	-1.62420400
H	-2.50709200	-2.46924200	-0.17368600
H	-3.05859300	-3.31676000	-1.60357500
C	-4.43230300	-0.35414200	-0.31800900
C	-3.87504400	-0.29246800	1.10865200
C	-3.59470000	1.15006600	1.57490300

C	-2.42257900	1.74393100	0.84701300
C	-2.46373600	2.78503800	0.01593200
C	-1.31153000	3.34667200	-0.77308500
C	0.04639400	2.70920900	-0.48729300
C	1.17953100	3.23775900	-1.37039800
C	2.55514400	2.67122500	-0.99454700
C	2.69387700	1.16072300	-1.21085900
C	4.03018900	0.59047900	-0.73224300
C	4.17475700	-0.91375200	-0.97890600
C	5.47047900	-1.49755800	-0.41321400
H	5.54984100	-2.56946200	-0.60990000
H	6.34510900	-1.01373000	-0.85579000
H	5.53470100	-1.35175300	0.67063500
H	3.31277800	-1.44050400	-0.54920100
H	4.12881900	-1.10249300	-2.05756100
H	4.85449500	1.11880300	-1.22409900
H	4.15383400	0.79845700	0.34106100
H	1.87918300	0.63298800	-0.70271000
H	2.56799200	0.94097100	-2.27853100
H	3.33048200	3.18317800	-1.57358900
H	2.76358500	2.91148300	0.05740100
H	1.21313800	4.32998700	-1.29926900
H	0.96384700	3.00701300	-2.42078600
H	0.30439400	2.89436100	0.56301600
H	-0.03665000	1.62488300	-0.60336400
H	-1.25408400	4.42786200	-0.59476100
H	-1.54431200	3.24961400	-1.84252200
H	-3.41867400	3.28680400	-0.13302600
H	-1.48157400	1.21262000	0.97978200
H	-4.48405800	1.77068800	1.42727600
H	-3.39660200	1.14364200	2.65432400
H	-2.93822500	-0.85922600	1.17192900
H	-4.57531200	-0.77656800	1.79731300
H	-5.46984900	-0.00491800	-0.30491300
H	-3.88919200	0.35826400	-0.94570800
H	-4.97086000	-2.44972200	-0.35587000
H	-4.88644800	-1.70153000	-1.93637800
N	1.16979400	-0.56675500	2.35321500
C	1.46704500	0.88998800	2.49622900
H	1.87498100	1.07294500	3.48996900
H	2.18755000	1.17659400	1.73370400
H	0.54552100	1.45089800	2.35722600
C	2.40292000	-1.40024000	2.47988000
H	3.13583900	-1.05221800	1.75534600
H	2.79695500	-1.30305500	3.49094500
H	2.14739500	-2.43714500	2.27059600
C	0.09710400	-1.00618500	3.29557600
H	-0.79800000	-0.41690600	3.10421700
H	0.43593500	-0.85559000	4.32003200
H	-0.11225300	-2.06009000	3.12157700
H	0.78855100	-0.72533600	1.39012600

conf_29

C	2.56852100	2.08995400	0.40682400
C	1.04247100	2.19287900	0.46135300
C	0.46383200	2.85816700	-0.79131400
C	-1.05475700	3.05151300	-0.76495200
C	-1.90647400	1.80778300	-0.78121700
O	-3.06530800	1.79935700	-0.39963300
O	-1.40205000	0.67140100	-1.27703100
H	-0.46024400	0.75408100	-1.49052800
H	-1.36717300	3.62713000	0.10892300
H	-1.38133400	3.63050900	-1.63796800
H	0.91908000	3.84404000	-0.91914900
H	0.76634400	2.29879600	-1.68908500
H	0.61765000	1.19005100	0.58978700
H	0.73832400	2.76310800	1.34647400
C	3.15742700	1.25113000	1.54198900
C	4.69093000	1.22580100	1.56861300
C	5.34409900	0.74628700	0.25633400
C	4.82705300	-0.58466600	-0.20620700
C	4.25402300	-0.81752100	-1.38620900
C	3.70622500	-2.13738300	-1.84448900
C	2.20275400	-2.07623800	-2.18051600
C	1.31291800	-1.57578000	-1.03402400
C	1.40566100	-2.41994200	0.24148300
C	0.49995400	-1.94874600	1.38539100
C	-0.99914800	-2.12560700	1.12103800
C	-1.88346900	-1.74880000	2.31776300
C	-1.89242200	-0.25151100	2.64028300
H	-2.22908800	0.34071700	1.78274300
H	-2.55292600	-0.02792600	3.48224800
H	-0.89644500	0.10625900	2.90947600
H	-2.91037700	-2.08980300	2.13082100
H	-1.55255300	-2.30867300	3.19873100
H	-1.29154500	-1.52604800	0.25096700
H	-1.18277100	-3.17346200	0.85491900
H	0.75826600	-2.50631400	2.29256300
H	0.72012300	-0.89881900	1.60532600
H	1.17520800	-3.46529800	0.00065000
H	2.43923700	-2.40938300	0.59611900
H	0.27514900	-1.55653700	-1.38677500
H	1.59417500	-0.54378900	-0.78744800
H	2.06163400	-1.42405600	-3.05070400
H	1.86963600	-3.07332600	-2.48875200
H	3.89382300	-2.89817800	-1.08179000
H	4.24033200	-2.46609900	-2.74351300
H	4.15696700	0.00972900	-2.09111700
H	4.92995000	-1.41061400	0.49838100
H	5.19712900	1.48843400	-0.53428000
H	6.42622300	0.68367100	0.41939800
H	5.01445300	0.57497500	2.38796400
H	5.07136900	2.22583400	1.80330500
H	2.78393300	0.22559800	1.45031400
H	2.79166000	1.62912700	2.50358700
H	2.99889400	3.09840600	0.42660700
H	2.85981200	1.65078900	-0.55274200
N	-4.87203700	-0.21581500	-0.41308700

C	-5.40018000	-0.28576200	0.98268000
H	-6.21236800	-1.01119100	1.02160100
H	-4.59392500	-0.58754500	1.64714800
H	-5.76292900	0.69970500	1.26878200
C	-4.28132900	-1.51537800	-0.85199900
H	-3.84710300	-1.38580800	-1.84146800
H	-3.50418300	-1.80391500	-0.14870200
H	-5.06511200	-2.27180300	-0.88067700
C	-5.90704700	0.27453800	-1.37170100
H	-6.25426400	1.25235100	-1.04324500
H	-6.73716600	-0.43089600	-1.39527500
H	-5.45902900	0.35691300	-2.36033000
H	-4.10196800	0.49958500	-0.41510100

conf_68

C	-5.45410200	-0.77535800	-0.74531700
C	-4.16823900	-1.57896900	-0.99223100
C	-3.18940500	-0.88166800	-1.94620600
C	-1.88407500	-1.64833500	-2.17007400
C	-0.95285800	-1.76588000	-0.98949100
O	-0.04530200	-2.58221100	-0.94555800
O	-1.08515300	-0.92805000	0.04408300
H	-1.79800300	-0.27354700	-0.07178900
H	-2.06855200	-2.66839400	-2.51584200
H	-1.28815700	-1.17178700	-2.95811900
H	-3.67063400	-0.74840000	-2.91920000
H	-2.96721200	0.13530000	-1.60189800
H	-3.67695900	-1.79125600	-0.03540500
H	-4.43011400	-2.55521800	-1.41324500
C	-5.22964400	0.58220900	-0.06483800
C	-4.76916800	0.49661000	1.39926600
C	-3.94175200	1.72665100	1.83106000
C	-2.53347100	1.66250600	1.31160200
C	-2.02538900	2.38338600	0.30752200
C	-0.60920300	2.29595800	-0.18421600
C	0.24742300	3.52245200	0.17437100
C	1.65895300	3.46481100	-0.42547400
C	2.44004700	2.19982700	-0.05562000
C	3.89553500	2.20179300	-0.52605200
C	4.59406300	0.86187600	-0.28501000
C	6.06488200	0.83085600	-0.70391000
C	6.71754600	-0.53411400	-0.47889500
H	6.21465100	-1.31329100	-1.06209800
H	7.76822600	-0.53073400	-0.77673400
H	6.67577100	-0.82259600	0.57671700
H	6.14408300	1.10716100	-1.76101700
H	6.61301800	1.59742000	-0.14571200
H	4.52061900	0.59971700	0.77995600
H	4.05255100	0.07957100	-0.83758500
H	3.93298700	2.44011800	-1.59546500
H	4.44540600	3.00006900	-0.01515100
H	2.41150900	2.06470900	1.03476700
H	1.93760300	1.32641700	-0.49102100

H	2.22036700	4.34464100	-0.09428100
H	1.59467500	3.53691600	-1.51797600
H	-0.25532600	4.43292500	-0.16665300
H	0.31003200	3.59550000	1.26600200
H	-0.60600400	2.17822600	-1.27704800
H	-0.14033000	1.40191600	0.23218400
H	-2.66182700	3.12168600	-0.17879700
H	-1.88118100	0.94960700	1.82011700
H	-4.43240100	2.63998800	1.48089800
H	-3.91497400	1.78053900	2.92412200
H	-4.16582400	-0.40386200	1.56478600
H	-5.64199700	0.39228400	2.04980500
H	-6.15742300	1.16002300	-0.10732700
H	-4.50162600	1.16155700	-0.64108300
H	-6.13706100	-1.37954900	-0.13911000
H	-5.95795000	-0.61717100	-1.70504300
N	1.81341100	-2.58039600	1.03511700
C	1.82281700	-1.25573500	1.72505700
H	2.53435000	-1.28876000	2.54963300
H	2.11606300	-0.48886200	1.01218800
H	0.82045200	-1.04842800	2.09306900
C	3.12730400	-2.87910900	0.39031500
H	3.37566200	-2.06962200	-0.29265500
H	3.89440200	-2.95903200	1.16003900
H	3.04477100	-3.81614800	-0.15728300
C	1.38144300	-3.67793800	1.95024600
H	0.39784500	-3.43474400	2.34820300
H	2.10116100	-3.77231500	2.76289500
H	1.32972700	-4.60692800	1.38533700
H	1.09115400	-2.53177000	0.27018200

conf_25

C	0.73634400	3.72878800	-1.49101800
C	0.40604000	3.41155600	-0.02452000
C	-0.83446500	2.52679800	0.15177900
C	-0.93460900	1.90133200	1.56909500
C	-0.00823600	0.72468800	1.67887900
O	1.18903500	0.81086600	1.89653100
O	-0.51325100	-0.50109100	1.45999700
H	-1.46990300	-0.47783100	1.29546300
H	-0.64174600	2.63665300	2.32042600
H	-1.95985300	1.59027800	1.77704600
H	-1.73843100	3.11749000	-0.01649600
H	-0.85255600	1.72655400	-0.59445300
H	1.26525700	2.93237600	0.45116700
H	0.24418400	4.34532700	0.52342800
C	1.24256900	2.51913300	-2.29105200
C	2.61254300	2.00089400	-1.83072900
C	2.88903700	0.54787500	-2.25023100
C	1.87963800	-0.39628200	-1.65275500
C	1.29722800	-1.42650000	-2.26665500
C	0.21623500	-2.28233600	-1.67422900
C	-1.07115900	-2.24126900	-2.51623300

C	-2.23665200	-3.03360900	-1.91079700
C	-2.75947500	-2.50594000	-0.56641000
C	-3.26513200	-1.06066800	-0.62051200
C	-3.93412200	-0.58820700	0.67306600
C	-4.47997200	0.83979100	0.59610000
C	-5.05030900	1.34169800	1.92309800
H	-4.28776100	1.35119900	2.70973100
H	-5.44321400	2.35654500	1.83053400
H	-5.86550400	0.70024200	2.26857500
H	-3.68975200	1.51622100	0.24853600
H	-5.25634600	0.87527000	-0.17512400
H	-4.74688400	-1.27505700	0.93390000
H	-3.23916100	-0.66683100	1.52917300
H	-2.44376300	-0.37947100	-0.87802000
H	-3.98686700	-0.96501200	-1.43971200
H	-3.57859600	-3.15298900	-0.23552400
H	-1.98133300	-2.60100600	0.20170100
H	-1.93548700	-4.08018400	-1.78934800
H	-3.06424600	-3.03732500	-2.62821100
H	-1.36281000	-1.19718900	-2.66857500
H	-0.85261900	-2.63845800	-3.51263000
H	0.00982900	-1.94759600	-0.65385400
H	0.55006200	-3.32727400	-1.60901000
H	1.57631300	-1.64923900	-3.29599200
H	1.56626300	-0.16091000	-0.63442600
H	2.88513600	0.45011200	-3.34013700
H	3.90459100	0.28010000	-1.92776500
H	2.68186900	2.05207500	-0.73822400
H	3.40219700	2.65363400	-2.21432900
H	1.29838800	2.78408800	-3.35143000
H	0.51035100	1.70718300	-2.23193600
H	1.49228500	4.52070500	-1.51695500
H	-0.15215300	4.14188200	-1.98100000
N	2.95158600	-1.27280700	1.94218700
C	3.15947100	-1.41633000	3.41420600
H	3.92881200	-2.16602000	3.59601000
H	3.46741500	-0.45472300	3.82054600
H	2.22111900	-1.72491400	3.87152700
C	4.18440700	-0.78712800	1.25235500
H	4.47193700	0.17078500	1.68192900
H	4.98182700	-1.51642200	1.39256800
H	3.96565000	-0.66955900	0.19366100
C	2.43461700	-2.53379000	1.32909700
H	1.47661400	-2.77504200	1.78525900
H	3.15229500	-3.33376000	1.50906300
H	2.30574200	-2.37365200	0.26108200
H	2.21948700	-0.52976100	1.82909400

conf_113

C	-5.09604600	0.87988600	1.85694600
C	-4.28959400	1.94536900	1.09819500
C	-2.84254400	1.52841700	0.81543200
C	-2.07226300	2.61444500	0.05530900

C	-0.63316300	2.29642400	-0.24479800
O	0.24958700	3.13464100	-0.29652500
O	-0.29581500	1.01726400	-0.48984400
H	-1.05368700	0.41943000	-0.39231400
H	-2.55405700	2.80378400	-0.91313300
H	-2.07840700	3.56474200	0.59111700
H	-2.33400500	1.30004400	1.75900000
H	-2.86011200	0.59986200	0.23136000
H	-4.77974700	2.18361700	0.14852000
H	-4.28884400	2.87367000	1.67880000
C	-5.07179600	-0.51486900	1.21653800
C	-5.62857300	-0.57498100	-0.21142100
C	-5.16195800	-1.82769100	-0.97787400
C	-3.71004100	-1.75133300	-1.35496400
C	-2.74314400	-2.55104100	-0.90503100
C	-1.29614200	-2.47102200	-1.29616000
C	-0.34434900	-2.38611200	-0.09294800
C	1.12577400	-2.28667800	-0.50083700
C	2.08980500	-2.19277200	0.68274800
C	3.55700800	-2.08450300	0.26108400
C	4.52423000	-1.94718500	1.44013200
C	6.00333000	-1.87421000	1.03874800
C	6.38768100	-0.60558500	0.27154900
H	6.19244800	0.28926900	0.87407200
H	7.45150900	-0.60078600	0.02427700
H	5.83747100	-0.51614100	-0.67044200
H	6.61860100	-1.93970500	1.94107900
H	6.25466900	-2.75332000	0.43517300
H	4.26274000	-1.05849700	2.03164800
H	4.38012800	-2.80104500	2.11047800
H	3.82905600	-2.97141300	-0.32309300
H	3.67300900	-1.23562900	-0.42367000
H	1.81831400	-1.33097300	1.30835100
H	1.96291800	-3.07102000	1.32564300
H	1.25822700	-1.41213500	-1.15201900
H	1.38846400	-3.15672700	-1.11349000
H	-0.61253800	-1.52794400	0.53873600
H	-0.48905700	-3.26719600	0.54198100
H	-1.13935900	-1.61478900	-1.96492400
H	-1.02752400	-3.36051600	-1.88024700
H	-3.00491400	-3.34702900	-0.20850600
H	-3.44931100	-0.96503100	-2.06666800
H	-5.34472400	-2.72328000	-0.37538500
H	-5.76141300	-1.93154700	-1.88901800
H	-5.32255200	0.30810800	-0.78365900
H	-6.72187400	-0.54670200	-0.18014300
H	-5.64153200	-1.20313800	1.84869600
H	-4.04631200	-0.89895200	1.21964600
H	-6.12978200	1.22946800	1.94589200
H	-4.71134800	0.80426800	2.88012300
N	2.83105300	2.32772600	-0.60744400
C	2.92380500	1.44509500	-1.80829800
H	3.93274500	1.04034600	-1.87672700
H	2.20031700	0.64006500	-1.70075100
H	2.69488200	2.03363300	-2.69512800

C	3.08007700	1.56633100	0.65354600
H	2.36281900	0.75106200	0.71156700
H	4.09335100	1.17009300	0.63952800
H	2.94868800	2.23914300	1.49914600
C	3.71849600	3.52265400	-0.72108000
H	3.56543300	4.15810200	0.14931800
H	3.45894600	4.07012800	-1.62541700
H	4.75629500	3.19370900	-0.76703100
H	1.83896600	2.66783000	-0.55022300

conf_163

C	4.34519100	0.66964000	0.22732400
C	5.60948700	-0.05027400	-0.24383600
C	5.70010500	-1.51786200	0.19394400
C	4.63710100	-2.44159100	-0.43575300
C	3.21779600	-2.25465700	0.03506000
O	2.25821200	-2.18961900	-0.71269200
O	2.99109800	-2.20069200	1.35755900
H	3.82037600	-2.21866700	1.85583900
H	4.62750700	-2.33296100	-1.52062900
H	4.89048500	-3.48725700	-0.22053700
H	6.67591000	-1.91971200	-0.08851700
H	5.67240400	-1.58104500	1.29111400
H	5.67159200	-0.00276900	-1.33740200
H	6.49051700	0.47745800	0.13437200
C	4.25158200	2.13005100	-0.22329700
C	2.98897700	2.83686300	0.28749100
C	1.67850900	2.24115000	-0.25629200
C	0.45297900	2.83188700	0.37873500
C	-0.54001800	3.44646800	-0.26443400
C	-1.75260000	4.04667700	0.38698200
C	-3.07524700	3.41105900	-0.08098600
C	-3.15587100	1.91456900	0.21972700
C	-4.48120100	1.26003600	-0.17366000
C	-4.46415200	-0.26044900	0.00152200
C	-5.79879800	-0.94449300	-0.30311800
C	-5.72151800	-2.47644700	-0.32531100
C	-5.30139100	-3.10572700	1.00621400
H	-5.32697700	-4.19681900	0.95250900
H	-4.28737000	-2.81447100	1.29861400
H	-5.97250200	-2.79848000	1.81372100
H	-6.69905800	-2.87431400	-0.61364300
H	-5.02713300	-2.78867500	-1.11697800
H	-6.54204400	-0.62661400	0.43740600
H	-6.16446200	-0.59271400	-1.27411300
H	-3.69784500	-0.68567400	-0.66412100
H	-4.15287400	-0.49937100	1.02583000
H	-5.29312500	1.68649900	0.42599300
H	-4.71556400	1.50058400	-1.21747100
H	-2.97442200	1.75262200	1.29090200
H	-2.33390400	1.41394300	-0.30360200
H	-3.90778500	3.93114300	0.40348100
H	-3.19758600	3.57208300	-1.15873900

H	-1.78341800	5.11955100	0.16539400
H	-1.66621100	3.95315200	1.47486800
H	-0.48681700	3.53410500	-1.35060300
H	0.40398500	2.76509100	1.46646900
H	1.63835800	2.37013100	-1.34391300
H	1.69108700	1.15780800	-0.07106600
H	3.02628400	3.89705100	0.02186800
H	2.97195800	2.79652600	1.38341800
H	4.28168600	2.17485200	-1.31877700
H	5.13315500	2.67473700	0.12993000
H	4.28819900	0.62894000	1.32317100
H	3.46732300	0.13891700	-0.14874100
N	-0.36154900	-1.54049700	-0.31400600
C	-0.62723400	-0.68622200	-1.51231600
H	-0.06602200	0.24016900	-1.41252600
H	-0.30332800	-1.22676300	-2.39979200
H	-1.69220000	-0.47056500	-1.56631800
C	-0.63516200	-0.81810800	0.96610100
H	-0.10061600	0.12903400	0.95793200
H	-1.70406200	-0.63284700	1.04634400
H	-0.29220300	-1.43801500	1.79267300
C	-1.10412000	-2.83532900	-0.38264500
H	-2.17433300	-2.63329700	-0.37037700
H	-0.82897100	-3.44274700	0.47783500
H	-0.82707900	-3.35021000	-1.30074400
H	0.65696500	-1.77789200	-0.34068700

conf_63

C	-3.65730500	-2.46769700	-0.10726800
C	-2.40227800	-2.94383800	0.63607600
C	-1.14411400	-2.92325000	-0.24804600
C	0.16461600	-2.91583300	0.54067900
C	0.55088700	-1.61567000	1.20005600
O	1.54737500	-1.50667300	1.89610200
O	-0.18472900	-0.51365700	0.99648400
H	-0.95408800	-0.67546400	0.42889200
H	0.15814700	-3.67118200	1.33254100
H	1.01616400	-3.16982900	-0.09747200
H	-1.14958800	-3.80242800	-0.89660100
H	-1.16214600	-2.07392700	-0.94304600
H	-2.25369800	-2.32808700	1.53111800
H	-2.54727900	-3.96228400	1.00706200
C	-3.72863800	-0.94411500	-0.28658400
C	-4.04193800	-0.18788600	1.00813900
C	-4.11763400	1.33665600	0.82374800
C	-2.79029300	2.00552200	0.60165900
C	-2.46165800	2.72540600	-0.47107700
C	-1.20400800	3.52500300	-0.65137300
C	-0.39660600	3.16190000	-1.90868300
C	0.34719800	1.83123100	-1.79741000
C	1.10724600	1.45852100	-3.07440500
C	2.11702400	0.31867800	-2.90083000
C	1.49903200	-1.00722200	-2.44725800

C	2.49334300	-2.17374400	-2.38734500
C	3.59182300	-2.00779600	-1.33251100
H	4.22673700	-1.14421000	-1.54662900
H	3.16622300	-1.87465700	-0.33180500
H	4.24034100	-2.88609600	-1.29824800
H	1.94497200	-3.10291300	-2.19504400
H	2.95302700	-2.30192500	-3.37303300
H	1.03973600	-0.87291700	-1.46094800
H	0.68223800	-1.26609600	-3.13106400
H	2.63986200	0.15690000	-3.84973500
H	2.88726600	0.63444600	-2.18599000
H	0.38629400	1.19392400	-3.85665500
H	1.63925700	2.34340700	-3.44068500
H	-0.36105000	1.04016600	-1.53049700
H	1.05857300	1.89865300	-0.96111400
H	-1.06850700	3.13507800	-2.77428300
H	0.32810500	3.95747900	-2.11178300
H	-0.57107600	3.42988200	0.23982800
H	-1.48299200	4.58362300	-0.71442300
H	-3.18392500	2.80254300	-1.28394300
H	-2.07636800	1.93155600	1.42421700
H	-4.79005600	1.57100900	-0.00791700
H	-4.57413800	1.77003900	1.72145200
H	-3.30073000	-0.41730500	1.78368600
H	-5.00101900	-0.54669900	1.39540700
H	-4.50444800	-0.70707700	-1.02171300
H	-2.79885600	-0.55959100	-0.73145400
H	-4.54811700	-2.79797000	0.43557800
H	-3.69844400	-2.95836200	-1.08565500
N	2.57222000	0.95597600	2.34875100
C	3.37603500	0.91174100	3.60600500
H	2.70851000	0.71754700	4.44361700
H	4.10791900	0.10992700	3.52672100
H	3.88033800	1.86785700	3.74382300
C	1.48722400	1.98049600	2.41100500
H	0.86592200	1.78093700	3.28218400
H	1.93504100	2.97078000	2.48921200
H	0.88430900	1.90305500	1.50947700
C	3.43489400	1.13853400	1.14296000
H	3.94129200	2.10127200	1.20646500
H	2.80636200	1.10326200	0.25553700
H	4.16304800	0.33069500	1.10657700
H	2.11102000	0.01597600	2.23917000

conf_140

C	-3.92084700	-1.04108000	-0.08019600
C	-4.11887800	-1.84631600	-1.37127200
C	-2.89710500	-1.96739000	-2.29356700
C	-1.69871800	-2.71790200	-1.67615500
C	-0.87813200	-1.95581800	-0.67105500
O	-0.52245500	-2.40108400	0.40520700
O	-0.47134800	-0.71802200	-1.00041500
H	-0.82602500	-0.44762100	-1.85876800

H	-2.02483800	-3.63861200	-1.19190800
H	-1.00008100	-3.00448300	-2.47240800
H	-3.19218400	-2.51910500	-3.18897900
H	-2.59375400	-0.97812100	-2.66024300
H	-4.44924100	-2.85759900	-1.11114200
H	-4.93536300	-1.40061500	-1.94856300
C	-3.62861100	0.44738500	-0.28830000
C	-3.39255400	1.19280500	1.02804600
C	-2.97459900	2.66585000	0.85867200
C	-1.63734000	2.82659600	0.19273200
C	-0.55703500	3.35789200	0.76695100
C	0.80499400	3.51209400	0.14532000
C	0.96263800	2.95841800	-1.27266500
C	2.41214900	2.96826900	-1.78127900
C	3.34274900	1.95263900	-1.10133800
C	2.97029500	0.49092100	-1.36863300
C	3.88747200	-0.51462800	-0.66859000
C	3.43437600	-1.96791000	-0.83393800
C	4.36915000	-2.97515600	-0.16199600
H	5.37208800	-2.92500400	-0.59362200
H	4.47260800	-2.77676100	0.91053900
H	4.00837800	-3.99989400	-0.27808400
H	2.41601600	-2.07991400	-0.43613200
H	3.35852400	-2.19961000	-1.90217000
H	4.90917300	-0.40679600	-1.04931500
H	3.95228500	-0.27114900	0.40224300
H	1.93602100	0.30249100	-1.06084300
H	2.99384600	0.30592000	-2.44954800
H	4.36693100	2.12618400	-1.44731000
H	3.36636800	2.13122600	-0.01882700
H	2.82695100	3.97444800	-1.65856500
H	2.41036600	2.77183800	-2.85905100
H	0.56961200	1.93683900	-1.31029400
H	0.34018800	3.54771600	-1.95307700
H	1.54226000	3.04637000	0.81331100
H	1.07325800	4.57649800	0.14759600
H	-0.64844200	3.73601600	1.78552800
H	-1.56702200	2.47694800	-0.83340200
H	-3.74337500	3.18147700	0.26996700
H	-2.95482100	3.15144100	1.83873400
H	-2.61250500	0.67053800	1.59838900
H	-4.30200300	1.14448500	1.63613000
H	-4.46466200	0.91164400	-0.82388400
H	-2.75243400	0.57235600	-0.92998500
H	-3.12390300	-1.49120100	0.52434800
H	-4.82829700	-1.14024700	0.52431400
N	0.70226200	-0.95002700	2.35976200
C	1.06353500	0.42979900	1.91566000
H	1.47506500	0.97653200	2.76376900
H	1.80252900	0.35790900	1.12269400
H	0.17267800	0.93142400	1.54127000
C	1.90303500	-1.73553300	2.77322600
H	2.60365000	-1.76556400	1.94211100
H	2.36395800	-1.25548200	3.63600000
H	1.59134800	-2.74685900	3.02818200

C	-0.34261800	-0.93035700	3.42600400
H	-1.20717700	-0.38111800	3.05787100
H	0.06049500	-0.44131100	4.31229000
H	-0.62761600	-1.95474700	3.65870400
H	0.28000000	-1.44891500	1.53971600

conf_92

C	2.64350400	-2.91836000	0.09907900
C	1.62783900	-3.04814400	-1.04598400
C	0.37239400	-2.20747300	-0.80941400
C	-0.64717500	-2.32417600	-1.96468500
C	-1.85752700	-1.50257000	-1.63405500
O	-1.90326400	-0.29126700	-1.74185400
O	-2.93356500	-2.12709700	-1.11131900
H	-2.80065100	-3.08367200	-1.06948900
H	-0.21206100	-1.92862700	-2.88405700
H	-0.91450500	-3.37102100	-2.13656400
H	-0.09934500	-2.52160300	0.12700400
H	0.63284800	-1.15352100	-0.69412700
H	2.08817900	-2.75837900	-1.99609600
H	1.34099200	-4.10007900	-1.15231300
C	3.27223500	-1.52633000	0.25081000
C	4.21005800	-1.13987700	-0.89525500
C	4.77818300	0.28267600	-0.76082000
C	3.76864400	1.36518100	-1.01810700
C	3.48504000	2.37854000	-0.20044000
C	2.57916600	3.53114100	-0.53184700
C	1.53919500	3.91021600	0.53786000
C	0.37828600	2.92081300	0.71044900
C	0.71071300	1.65286700	1.50079800
C	-0.49484600	0.72630500	1.66761500
C	-0.19292700	-0.55120800	2.45156300
C	-1.39420300	-1.49151300	2.57549700
C	-1.05380200	-2.81379600	3.26465700
H	-0.66898600	-2.64169000	4.27327400
H	-1.92876700	-3.46301700	3.35031900
H	-0.28346800	-3.35914500	2.71042900
H	-2.19421300	-0.98608900	3.13091000
H	-1.80084500	-1.69350900	1.57528500
H	0.63551900	-1.08132500	1.96951900
H	0.16389600	-0.28762000	3.45352000
H	-1.30138100	1.27572900	2.17507300
H	-0.87369300	0.45579100	0.67222700
H	1.52025500	1.11225800	1.00507500
H	1.09083700	1.93430200	2.48968900
H	0.00226300	2.63960100	-0.28340500
H	-0.44450900	3.43953500	1.22096300
H	2.04060100	4.05445000	1.50228800
H	1.12536100	4.88622800	0.26536200
H	2.07257500	3.33763200	-1.48467500
H	3.21043500	4.41362600	-0.69790500
H	3.99067100	2.43128900	0.76411500
H	3.26702600	1.32155400	-1.98631800

H	5.22135800	0.41497300	0.23209000
H	5.59645900	0.39580800	-1.48248900
H	3.69070800	-1.21907000	-1.85756400
H	5.03694900	-1.85712200	-0.93532500
H	3.83728100	-1.49851900	1.18882800
H	2.49331700	-0.76465300	0.35527900
H	3.44018000	-3.65351200	-0.05531200
H	2.15107000	-3.19931000	1.03752200
N	-3.89028300	1.20179400	-0.61973200
C	-3.35859000	2.58609300	-0.43813300
H	-4.13983200	3.21470400	-0.01156300
H	-2.49893400	2.54704700	0.22670900
H	-3.05344600	2.97443100	-1.40813800
C	-4.25846900	0.57143000	0.68396900
H	-3.39047300	0.59163600	1.33859500
H	-5.08172300	1.12940900	1.12903100
H	-4.55335300	-0.45942500	0.49915700
C	-5.02311700	1.16080100	-1.59170300
H	-4.68819900	1.57747500	-2.53991900
H	-5.85421200	1.74545200	-1.19853900
H	-5.32602500	0.12454100	-1.73059100
H	-3.12115800	0.62209200	-1.03549000

conf_97

C	4.79241400	0.32601800	1.00301200
C	5.30720200	0.41748200	-0.43852700
C	5.52234200	-0.94035300	-1.12406500
C	4.25766500	-1.52261400	-1.79232400
C	3.12017400	-1.85147000	-0.86501300
O	2.00078700	-1.38376200	-0.96972400
O	3.34358400	-2.73760900	0.12221000
H	4.27095900	-3.01257300	0.14558400
H	3.86872400	-0.82238000	-2.53127900
H	4.51664500	-2.44802900	-2.32098200
H	6.26419500	-0.83989300	-1.91944500
H	5.95357700	-1.65635000	-0.41203800
H	4.62539400	1.01692800	-1.05090700
H	6.25718200	0.96001200	-0.42865300
C	4.49055800	1.68911000	1.64167900
C	3.33168600	2.46341900	0.99733400
C	2.00165900	1.69054400	1.00981800
C	0.84724700	2.49440200	0.48606400
C	0.21680800	2.25881600	-0.66587100
C	-0.89898600	3.08375400	-1.24076500
C	-2.18325000	2.27610100	-1.49918800
C	-2.86199000	1.78045700	-0.22111200
C	-4.05600100	0.85950600	-0.47785200
C	-4.63501000	0.25225800	0.80314900
C	-5.74173300	-0.78362800	0.57263400
C	-7.01207000	-0.22152300	-0.07368100
C	-8.12051800	-1.26765500	-0.20279600
H	-8.40659200	-1.66230400	0.77668600
H	-9.01545700	-0.84485700	-0.66511500

H	-7.79615500	-2.11215900	-0.81880100
H	-7.37133500	0.62384500	0.52511400
H	-6.77889700	0.18188700	-1.06437000
H	-5.35367500	-1.60003900	-0.05281100
H	-6.00843700	-1.23799300	1.53389600
H	-3.81981800	-0.21691500	1.37257900
H	-5.01616800	1.05562300	1.44459600
H	-4.83161100	1.41641700	-1.01185400
H	-3.75652800	0.05012600	-1.15854900
H	-3.18601500	2.64212600	0.37399600
H	-2.12651200	1.25965900	0.40367400
H	-2.88647700	2.89152900	-2.06875600
H	-1.94412000	1.42139600	-2.14718900
H	-0.56330100	3.51638100	-2.19059100
H	-1.11773600	3.92434500	-0.57440100
H	0.54399700	1.41070900	-1.27178900
H	0.54412100	3.35289800	1.08429600
H	2.09896200	0.78062300	0.41154000
H	1.80007400	1.37854000	2.04441100
H	3.57197500	2.73400800	-0.03549900
H	3.20180800	3.40968900	1.53186100
H	5.39422700	2.30708200	1.60983600
H	4.26496200	1.53485900	2.70280900
H	5.54723100	-0.18873800	1.60824100
H	3.89491100	-0.29820400	1.05452700
N	-0.26988600	-1.82797800	0.48803800
C	-1.34914600	-1.56051900	-0.51075400
H	-1.21782100	-2.23269200	-1.35663700
H	-2.31836400	-1.72563900	-0.04255600
H	-1.26787600	-0.52787800	-0.83960500
C	-0.35370100	-0.89243400	1.65033100
H	-0.31262000	0.12967300	1.27925100
H	-1.29232800	-1.06555500	2.17559600
H	0.48841100	-1.08335800	2.31287300
C	-0.25501300	-3.25546600	0.92413400
H	-1.18883100	-3.48147100	1.43777300
H	0.59042000	-3.40924900	1.59208200
H	-0.14815400	-3.89026200	0.04632600
H	0.63928400	-1.65224900	0.00087400

conf_88

C	3.19645700	-2.97037900	0.22362200
C	1.67052800	-3.03162300	0.37909100
C	1.12996200	-2.19970500	1.54949000
C	-0.39904600	-2.19918200	1.64902900
C	-1.13817700	-1.57408200	0.49261400
O	-2.28708600	-1.86481800	0.20369800
O	-0.53045600	-0.62706000	-0.23309500
H	0.37392100	-0.46001800	0.07359600
H	-0.79637300	-3.21009100	1.75895700
H	-0.72709400	-1.64489900	2.53742900
H	1.52972400	-2.58707300	2.49082100
H	1.50200400	-1.16822200	1.49540800

H	1.21594000	-2.68860300	-0.55958500
H	1.35059900	-4.07105500	0.50819200
C	3.72907900	-1.53996600	0.09473100
C	5.14485200	-1.44201300	-0.47827900
C	5.74325000	-0.02489600	-0.34268400
C	4.83720900	1.03739800	-0.89625700
C	4.16123900	1.90898900	-0.14945600
C	3.12614500	2.87518300	-0.64638500
C	1.71003800	2.40170700	-0.27163600
C	0.60697500	3.41695600	-0.58325800
C	-0.81029100	2.85901300	-0.39758800
C	-1.15222800	2.45053600	1.03844700
C	-2.52737700	1.79406500	1.16971600
C	-2.84237500	1.29368600	2.58134200
C	-4.16259800	0.52674400	2.67004200
H	-5.00199300	1.14572000	2.33726100
H	-4.13488200	-0.37476100	2.04890500
H	-4.37589100	0.21232800	3.69398900
H	-2.02299600	0.64923400	2.92396900
H	-2.86064800	2.14458200	3.27001900
H	-3.30503100	2.50070900	0.85148000
H	-2.57244700	0.94426000	0.47738400
H	-0.39815200	1.75175300	1.41674500
H	-1.09983700	3.32937700	1.69118100
H	-1.53889400	3.60350200	-0.73909000
H	-0.92582700	1.98467000	-1.05127300
H	0.71994700	3.75934400	-1.61776000
H	0.74092100	4.30312400	0.04770700
H	1.51073200	1.46796600	-0.81679900
H	1.69702100	2.15147900	0.79517000
H	3.19928400	2.98728000	-1.73282300
H	3.29556500	3.86718500	-0.21156000
H	4.31091000	1.89126700	0.93041100
H	4.69191000	1.04200200	-1.97708400
H	5.93114700	0.18115700	0.71639100
H	6.71439500	-0.00051200	-0.84796900
H	5.12871700	-1.74101300	-1.53273800
H	5.80410600	-2.15138700	0.03335200
H	3.71136400	-1.04535000	1.07269900
H	3.05533800	-0.95578300	-0.54445700
H	3.47359200	-3.54782700	-0.66448600
H	3.67738400	-3.46858400	1.07285600
N	-3.88736900	-0.79041400	-1.70829400
C	-5.10853500	-0.25846200	-1.03165200
H	-5.82700400	0.05461200	-1.78878500
H	-5.53560000	-1.04372100	-0.41083700
H	-4.82381700	0.58597600	-0.40851300
C	-4.19294800	-2.00070400	-2.52904900
H	-4.63261400	-2.75925900	-1.88426700
H	-4.88968700	-1.72995800	-3.32175200
H	-3.26583500	-2.37673900	-2.95776300
C	-3.19533200	0.26234000	-2.51031900
H	-2.96513100	1.10394700	-1.86123600
H	-3.84921000	0.57849200	-3.32258500
H	-2.27174700	-0.15220400	-2.90969000

H	-3.22284300	-1.10862100	-0.95901000

conf_179

C	3.11121800	3.28678100	-0.47934600
C	3.44529900	1.83470600	-0.11311100
C	3.32143800	0.84635300	-1.27720900
C	3.60156900	-0.59902300	-0.85275900
C	2.58188300	-1.11071900	0.13367000
O	1.44742800	-0.68303600	0.21370300
O	2.94861900	-2.09254900	0.97769400
H	3.86041100	-2.37269700	0.81861700
H	3.56532500	-1.27271800	-1.71746300
H	4.60526000	-0.69544200	-0.42422900
H	4.02384800	1.11494600	-2.07103300
H	2.32098600	0.89014200	-1.71150500
H	4.46675600	1.79654400	0.28269400
H	2.78800600	1.51339900	0.70177600
C	1.71376600	3.50697400	-1.07501900
C	0.55124900	2.96730100	-0.23394800
C	-0.81086900	3.37693100	-0.81638400
C	-1.99487700	2.79493300	-0.09859500
C	-2.97675100	2.11258600	-0.68978300
C	-4.22979100	1.60085500	-0.03169600
C	-4.80971900	0.30352000	-0.62361800
C	-4.02931000	-0.98619800	-0.33804800
C	-2.69093600	-1.11891800	-1.07129600
C	-1.94643500	-2.41951200	-0.76116900
C	-0.67282800	-2.59675400	-1.59066600
C	0.20748000	-3.75601700	-1.11910600
C	1.44472800	-3.96259800	-1.99386200
H	2.04519600	-3.04838900	-2.05111100
H	2.08273000	-4.76282700	-1.61051800
H	1.16408000	-4.22388300	-3.01762900
H	-0.38142100	-4.67980000	-1.10047800
H	0.51853500	-3.57227800	-0.08270600
H	-0.94957800	-2.75172600	-2.63994200
H	-0.09080800	-1.66871600	-1.56056400
H	-2.61169000	-3.27593600	-0.92034000
H	-1.68603900	-2.44962800	0.30777900
H	-2.04115400	-0.26910600	-0.83927300
H	-2.87561000	-1.06312000	-2.15060500
H	-4.65830200	-1.83999600	-0.61312100
H	-3.87940200	-1.07666800	0.74702000
H	-5.82417600	0.18328200	-0.23230500
H	-4.91907100	0.42640800	-1.70782600
H	-4.07274500	1.48818900	1.04854700
H	-4.99932500	2.37621600	-0.13708700
H	-2.91411600	1.95518200	-1.76629000
H	-2.06125300	2.99950400	0.97192300
H	-0.85445700	3.08720100	-1.87158300
H	-0.87719900	4.47234300	-0.79603300
H	0.60504700	1.87398700	-0.18011900
H	0.63582800	3.34437400	0.79500600

H	1.65912100	3.06109500	-2.07445300
H	1.57271800	4.58240700	-1.22550400
H	3.21775800	3.90007200	0.42225600
H	3.85782600	3.66004400	-1.18847700
N	-0.07313600	-0.57163800	2.45355100
C	0.78130500	0.43225900	3.15556200
H	1.78640100	0.02749900	3.25882000
H	0.35765500	0.63613100	4.13827800
H	0.80633300	1.34282900	2.55988700
C	-0.09750400	-1.88158200	3.17013300
H	-0.69342200	-2.58424400	2.59076900
H	0.92274400	-2.25080400	3.25719900
H	-0.53693900	-1.74117500	4.15712700
C	-1.44593400	-0.03910600	2.20239500
H	-1.90259100	0.22480700	3.15597200
H	-1.37598500	0.83410700	1.55632000
H	-2.03246700	-0.80617300	1.70635000
H	0.38889600	-0.73587000	1.53095100

conf_123

C	-0.48179500	1.74889200	-0.91511000
C	-0.72157600	2.90284000	0.06640100
C	-2.19609200	3.19923400	0.37048300
C	-2.87912800	2.18094300	1.30481100
C	-3.14309700	0.85096700	0.65608000
O	-3.71543000	0.71893300	-0.41077200
O	-2.73954900	-0.26689300	1.29069100
H	-2.25334300	-0.06158300	2.10382900
H	-2.29786500	2.05515300	2.22149800
H	-3.86274100	2.56495300	1.59622600
H	-2.27159700	4.17317300	0.85953900
H	-2.77118200	3.26893300	-0.55704400
H	-0.18626100	2.70975000	1.00450200
H	-0.27848400	3.81176600	-0.34723500
C	0.99596700	1.40759700	-1.14440100
C	1.87013400	2.58979700	-1.57339400
C	3.28735800	2.16572900	-2.00079600
C	3.98113000	1.33499600	-0.95945600
C	4.53891300	0.14555700	-1.17588700
C	5.18532000	-0.71875100	-0.13299300
C	4.66588000	-2.16956200	-0.16633700
C	3.13968600	-2.30501000	-0.10337000
C	2.50043600	-1.74214700	1.16742600
C	0.98502600	-1.96246700	1.19876000
C	0.29534200	-1.50032400	2.48714100
C	0.32576000	0.01591300	2.70432300
C	-0.47425800	0.46496900	3.92948600
H	-1.52533300	0.14053700	3.89230100
H	-0.06565700	0.02973900	4.84470400
H	-0.46635400	1.55126600	4.04752100
H	1.35911300	0.35432700	2.81608400
H	-0.04518600	0.51513200	1.80093400

H	-0.74833800	-1.84328100	2.47590700
H	0.75739200	-1.99781900	3.34740600
H	0.53706500	-1.44104800	0.34138300
H	0.78329400	-3.03023300	1.05257600
H	2.95171500	-2.21661400	2.04769300
H	2.72204800	-0.67360600	1.24277200
H	2.87801000	-3.36584800	-0.19408700
H	2.69783100	-1.80180200	-0.97203400
H	5.01819300	-2.64681800	-1.08741100
H	5.11904500	-2.72826500	0.65966700
H	5.03142200	-0.27615400	0.85606800
H	6.26996500	-0.74412100	-0.29201800
H	4.52062300	-0.26102100	-2.18783900
H	4.00429400	1.74999900	0.04928500
H	3.23854100	1.60231700	-2.93891600
H	3.86976000	3.07108900	-2.20906400
H	1.95058900	3.30423500	-0.74722500
H	1.39279800	3.12742800	-2.40087400
H	1.06388000	0.61976900	-1.90500400
H	1.41268900	0.96945300	-0.23225600
H	-0.95893400	2.00093500	-1.87040300
H	-0.97889600	0.84076700	-0.55666100
N	-3.43712900	-1.72236900	-1.57705300
C	-1.96167800	-1.95867000	-1.58055700
H	-1.75452800	-2.92199000	-2.04551600
H	-1.47945600	-1.16043500	-2.14182800
H	-1.60553000	-1.95143500	-0.55302800
C	-3.99298500	-1.64365800	-2.96062600
H	-3.47240700	-0.85664000	-3.50323900
H	-3.84959000	-2.60133300	-3.46024700
H	-5.05396600	-1.40790300	-2.90074500
C	-4.15253800	-2.73158900	-0.74026900
H	-3.75939300	-2.67889500	0.27287900
H	-3.99207300	-3.72414000	-1.16014100
H	-5.21508600	-2.49484300	-0.73623700
H	-3.59316400	-0.78617100	-1.12398000

conf_149

C	3.15765300	-1.60995000	-0.04945300
C	2.25072800	-2.61144400	0.67201200
C	0.75612800	-2.33554400	0.47224800
C	0.32041400	-2.45318900	-1.00896100
C	-1.08268100	-1.95563400	-1.18225900
O	-1.35419100	-0.82240100	-1.53928700
O	-2.09963000	-2.77822800	-0.87289400
H	-1.78057000	-3.64835500	-0.59716200
H	0.95723700	-1.83344500	-1.63762200
H	0.40779000	-3.49095000	-1.34666300
H	0.16319500	-3.02278900	1.08503700
H	0.53015200	-1.32507500	0.82208500
H	2.48953600	-3.63406200	0.35677000
H	2.45349200	-2.56926600	1.74749400
C	4.62443600	-1.71931200	0.37824800

C	5.59949300	-0.84373000	-0.42036500
C	5.49220000	0.67473200	-0.17961700
C	4.29144100	1.34119900	-0.78921800
C	3.45470400	2.16218900	-0.15586400
C	2.27465600	2.84333300	-0.79024500
C	0.91465500	2.39749200	-0.21861900
C	0.74112000	2.70304800	1.27301700
C	-0.66087100	2.41092500	1.82031800
C	-1.05494200	0.93272400	1.76212500
C	-2.40501600	0.62408700	2.41128300
C	-2.79551500	-0.85424500	2.32965100
C	-4.15171500	-1.15905900	2.96786700
H	-4.40441100	-2.21933200	2.89134700
H	-4.15434500	-0.89279500	4.02798700
H	-4.95750200	-0.58970800	2.49167100
H	-2.79791000	-1.17747500	1.28070900
H	-2.01916800	-1.45280000	2.81954300
H	-2.38355700	0.93408200	3.46197200
H	-3.19103000	1.23904500	1.94816200
H	-1.06395800	0.58877300	0.72134600
H	-0.27886200	0.34233900	2.26463100
H	-0.71617600	2.75007500	2.86026400
H	-1.39770200	3.01402200	1.27145500
H	0.97591000	3.75926100	1.44581400
H	1.47199200	2.12997900	1.85387700
H	0.11964500	2.90866300	-0.77848400
H	0.78800200	1.32566900	-0.40520300
H	2.28983400	2.65896500	-1.86906600
H	2.36617500	3.92900000	-0.65681500
H	3.62772800	2.36581400	0.89958100
H	4.12724800	1.14343400	-1.84951600
H	5.52402100	0.88179000	0.89586400
H	6.38990600	1.13861800	-0.60766300
H	5.48146200	-1.04958000	-1.49175100
H	6.61823100	-1.15122100	-0.16596000
H	4.94103000	-2.76478700	0.28334700
H	4.70852200	-1.47316800	1.44409100
H	2.79471000	-0.59588800	0.13598900
H	3.10070200	-1.76089000	-1.13408700
N	-3.62645200	0.62443700	-1.69804800
C	-3.99641500	0.57184200	-3.14387200
H	-3.13785600	0.88185000	-3.73666600
H	-4.27043500	-0.45026200	-3.39927900
H	-4.83779900	1.24047100	-3.32336700
C	-3.16590000	1.98682100	-1.29242600
H	-2.86166800	1.95679400	-0.24924300
H	-2.31530400	2.26644700	-1.91084000
H	-3.98242600	2.69550900	-1.42818600
C	-4.72939500	0.12524200	-0.82411100
H	-4.39583700	0.15000200	0.21024700
H	-4.96601400	-0.89851500	-1.10824500
H	-5.60347800	0.76295000	-0.95281100
H	-2.80584800	-0.01837900	-1.57743100

conf_260

C	2.65176500	-0.58426000	1.32425900
C	2.12483500	-0.60989200	2.76238500
C	0.95327900	0.33036900	3.06510400
C	1.26638900	1.81661200	2.81255300
C	1.04810300	2.23940300	1.38497500
O	0.10042500	1.87199400	0.71671800
O	1.91078500	3.11164500	0.82726700
H	2.63467400	3.32390300	1.43200000
H	0.58195000	2.44261200	3.39780800
H	2.28006400	2.06513300	3.14227200
H	0.67356400	0.21991000	4.11511600
H	0.07519500	0.05864400	2.47541000
H	1.80892100	-1.63007600	3.00386500
H	2.94966300	-0.38269900	3.44934600
C	3.81113400	-1.56917400	1.13161900
C	4.45140700	-1.57852300	-0.26398300
C	3.71479500	-2.41356400	-1.32718800
C	2.39255200	-1.86651300	-1.77899300
C	1.24941000	-2.54609100	-1.85412900
C	-0.04714000	-2.00070800	-2.37878700
C	-1.21612500	-2.08916700	-1.38229200
C	-0.92376200	-1.42633400	-0.03276900
C	-2.15443700	-1.29218300	0.87203800
C	-3.10210100	-0.16285900	0.45473800
C	-4.33508300	-0.00827600	1.35577800
C	-5.26312000	-1.23085400	1.40742300
C	-5.81182200	-1.65295900	0.04230400
H	-6.53073500	-2.46908300	0.14405200
H	-6.32376000	-0.82047700	-0.45135600
H	-5.01909900	-2.00002300	-0.62670100
H	-6.09862500	-0.99778500	2.07488900
H	-4.74095800	-2.07531300	1.86904100
H	-4.00293400	0.22941600	2.37333000
H	-4.91433000	0.85780200	1.01317000
H	-3.42741100	-0.31436100	-0.58057800
H	-2.53754200	0.77794700	0.47113000
H	-1.83380000	-1.11038100	1.90412700
H	-2.68991400	-2.24704600	0.88870100
H	-0.48371200	-0.43364300	-0.18934700
H	-0.15195900	-2.00912600	0.47851700
H	-2.10453400	-1.64359000	-1.84407100
H	-1.46787200	-3.14198200	-1.21384300
H	0.10705500	-0.95633400	-2.67972400
H	-0.32560900	-2.54031300	-3.29197500
H	1.23340000	-3.58512800	-1.52626500
H	2.40600700	-0.82711700	-2.11371000
H	3.58133000	-3.43651400	-0.95982800
H	4.37076100	-2.48656000	-2.20411300
H	4.56960200	-0.54778700	-0.62376400
H	5.46458400	-1.98133800	-0.17661100
H	3.46525900	-2.58097300	1.37499400
H	4.58475500	-1.33184800	1.87051600
H	1.84324700	-0.81070200	0.62567800

H	3.01436900	0.42159000	1.06889400
N	-0.35794000	2.53808600	-1.87273100
C	0.89483500	2.16755700	-2.59666500
H	0.79930500	2.44608800	-3.64562800
H	1.04468100	1.09377400	-2.50607300
H	1.72888000	2.69597200	-2.13857700
C	-0.58661000	4.01351100	-1.88579800
H	-1.48149200	4.23504400	-1.30688300
H	0.27507800	4.50314900	-1.43564400
H	-0.71543600	4.34767800	-2.91480500
C	-1.54307800	1.78438300	-2.38237400
H	-1.34779400	0.71903200	-2.28975000
H	-2.41122300	2.04661300	-1.78156100
H	-1.71078800	2.04934400	-3.42600300
H	-0.20829000	2.26795900	-0.87000600

11Z_NMe3H

conf_2

C	0.97220200	1.19878100	1.94056600
C	2.39667700	1.75991800	1.90196500
C	2.65352600	2.82124000	0.82542000
C	2.50903700	2.31099900	-0.61967600
C	1.07993100	2.11426500	-1.04503500
O	0.19694300	2.92696800	-0.83571600
O	0.75342900	0.99740100	-1.71884400
H	1.49705800	0.38083600	-1.78005400
H	2.91648800	3.05792300	-1.31070600
H	3.08525100	1.39496800	-0.76541600
H	1.98236900	3.67342100	0.96288100
H	3.67145200	3.20198900	0.93636100
H	2.62362400	2.20761000	2.87481900
H	3.10689200	0.93469700	1.77735500
C	0.73277200	0.26031800	3.12682500
C	-0.70363900	-0.26835000	3.22101700
C	-1.08942100	-1.25784700	2.11826200
C	-2.53729300	-1.73996800	2.22555500
C	-2.93450800	-2.75470700	1.13648500
C	-3.00739500	-2.15928500	-0.24238000
C	-2.16206600	-2.35116400	-1.25928300
C	-0.91395700	-3.18553400	-1.26366700
C	0.35806300	-2.33301100	-1.40009100
C	1.63683800	-3.16968200	-1.47399900
C	2.92471000	-2.34130100	-1.54717200
C	3.25981300	-1.56906200	-0.26531600
C	4.61786400	-0.86814300	-0.33487700
H	5.42278500	-1.59581800	-0.46464500
H	4.67379800	-0.17769700	-1.18356300
H	4.83035700	-0.30110900	0.57485600
H	3.25322100	-2.26554100	0.58036800
H	2.47246800	-0.83854800	-0.03921200
H	3.76319600	-3.00695100	-1.77624200
H	2.86744800	-1.64532800	-2.39837800

H	1.58035800	-3.82497700	-2.34939000
H	1.68810000	-3.83114800	-0.60087400
H	0.27819100	-1.71375600	-2.30375700
H	0.40274000	-1.64619100	-0.54928500
H	-0.84289300	-3.78683800	-0.35486100
H	-0.95705500	-3.89120000	-2.10176500
H	-2.38623200	-1.86026100	-2.20581700
H	-3.86828500	-1.51411000	-0.41691000
H	-3.91814600	-3.16646800	1.38556000
H	-2.23481100	-3.59339300	1.16161700
H	-3.21824600	-0.87901200	2.19105600
H	-2.68922100	-2.20102600	3.20673000
H	-0.41829700	-2.12446700	2.16348200
H	-0.92629800	-0.81318900	1.13054800
H	-1.40037600	0.58098600	3.21277200
H	-0.84157400	-0.75574000	4.19159700
H	0.97740000	0.79634600	4.04979900
H	1.43019000	-0.58431700	3.07055100
H	0.75496800	0.65869500	1.01225400
H	0.25811400	2.03058500	1.98964000
N	-2.42492900	2.27018700	-1.07028500
C	-2.63945000	1.34921700	-2.22592000
H	-3.69573000	1.08745600	-2.28163700
H	-2.03843500	0.45644700	-2.07111800
H	-2.32944100	1.85412200	-3.13907000
C	-3.14952900	3.56413400	-1.24005300
H	-2.82083200	4.03284600	-2.16590700
H	-4.22156300	3.37256800	-1.27752700
H	-2.91375000	4.21186400	-0.39758800
C	-2.75922100	1.60955000	0.22682700
H	-2.20356500	0.67794800	0.29890900
H	-3.82862800	1.40366100	0.25690200
H	-2.48180700	2.27570500	1.04148700
H	-1.39946400	2.49794400	-1.04007100

conf_4

C	2.66991100	-1.43925500	-2.03976800
C	3.71555900	-0.35072600	-2.31252400
C	3.79960900	0.77515500	-1.27272700
C	4.23141700	0.29865200	0.12546300
C	3.11690100	-0.28372900	0.95388500
O	1.99736400	0.18859400	1.00577700
O	3.38459100	-1.35316600	1.73012700
H	4.29117600	-1.66293200	1.60088200
H	5.06608600	-0.40582500	0.05205300
H	4.59278800	1.15510100	0.70774500
H	4.53507100	1.50871900	-1.61061600
H	2.84995100	1.30629400	-1.18563000
H	4.70361800	-0.81630100	-2.40415700
H	3.50617200	0.10298600	-3.28663700
C	1.22194800	-0.94698700	-2.07568000
C	0.19760700	-2.05912400	-1.83714500
C	-1.22973900	-1.52402400	-1.72928800

C	-2.30079000	-2.59754400	-1.53750200
C	-3.69484900	-2.00253600	-1.26098500
C	-3.80317100	-1.37888700	0.10247100
C	-4.06987200	-0.10756200	0.41668400
C	-4.34437500	1.04795700	-0.50455200
C	-3.44905100	2.26829500	-0.22114800
C	-1.99322000	2.06336100	-0.64130000
C	-1.05551800	3.19727200	-0.22741500
C	0.39429600	2.96033700	-0.65837200
C	1.34956000	4.06574800	-0.20675200
H	2.37415600	3.86965800	-0.53480400
H	1.05369600	5.03644200	-0.61407100
H	1.36085300	4.15704600	0.88412200
H	0.43040900	2.86583400	-1.75002900
H	0.73491100	1.99999500	-0.25679900
H	-1.41411100	4.14362600	-0.64870600
H	-1.09451800	3.32364200	0.86352600
H	-1.94840400	1.92993300	-1.72872500
H	-1.62185100	1.12069800	-0.22306000
H	-3.85463200	3.14088700	-0.74244700
H	-3.49651400	2.50835700	0.84934900
H	-4.23921800	0.75138100	-1.55104200
H	-5.39117600	1.34617100	-0.37169600
H	-4.14381500	0.13974500	1.47627100
H	-3.67420500	-2.08025900	0.92728300
H	-4.43966200	-2.80329700	-1.33098700
H	-3.94283800	-1.28172900	-2.04377800
H	-2.02105000	-3.26490300	-0.71083300
H	-2.34734400	-3.22985100	-2.42954800
H	-1.46791700	-0.93983100	-2.62597500
H	-1.28042000	-0.80872900	-0.90114900
H	0.45516600	-2.60042900	-0.91589600
H	0.25911100	-2.79897800	-2.64340500
H	1.02696000	-0.47950300	-3.04798100
H	1.07453700	-0.16240800	-1.32803300
H	2.86618600	-1.92494400	-1.07328300
H	2.79660900	-2.23192900	-2.78466000
N	-0.03120100	-0.67898900	2.61553100
C	-1.05379800	0.40481400	2.72826200
H	-1.52789700	0.54371000	1.75992100
H	-0.55796800	1.32282700	3.03839800
H	-1.79972600	0.11203300	3.46654900
C	0.65917200	-0.93092200	3.91568200
H	-0.06390800	-1.31191300	4.63594500
H	1.45374200	-1.65634000	3.75275800
H	1.08472200	0.00499100	4.27367100
C	-0.61068400	-1.93188500	2.04483600
H	0.19131200	-2.65462800	1.90596900
H	-1.07822200	-1.69878400	1.09169300
H	-1.35579000	-2.32516400	2.73558700
H	0.71124100	-0.35236000	1.95351500

conf_8

C	-1.70993500	2.08568100	-0.17831200
C	-3.01471400	2.13175000	-0.97994400
C	-3.80341700	0.81873800	-1.04898400
C	-4.20210400	0.25700900	0.32508000
C	-3.08801400	-0.48270800	1.01620100
O	-2.27782100	-1.18126500	0.43892400
O	-3.00767500	-0.40820000	2.36199200
H	-3.68412500	0.18175900	2.72132800
H	-4.59729400	1.04713100	0.97204000
H	-5.00453600	-0.48148900	0.20518900
H	-4.71917500	0.98442700	-1.62101700
H	-3.23433200	0.05447600	-1.58125600
H	-3.65963900	2.91520600	-0.56323000
H	-2.78923100	2.44359200	-2.00487800
C	-0.95850100	3.41980300	-0.22178700
C	0.25998200	3.49610400	0.70390000
C	1.40371300	2.54263700	0.34601200
C	2.64713100	2.77510200	1.20766800
C	3.84056600	1.87514800	0.84638900
C	3.63192700	0.43249000	1.20671300
C	3.74891000	-0.63941500	0.41834600
C	4.11113600	-0.68126700	-1.03904700
C	3.06877100	-1.41588400	-1.89848700
C	1.72004200	-0.69853600	-1.97259700
C	0.68209500	-1.43736200	-2.81825500
C	-0.68046600	-0.74269600	-2.85143100
C	-1.70772900	-1.47465100	-3.71584300
H	-2.66778200	-0.95193600	-3.73146100
H	-1.36372700	-1.55958600	-4.75028500
H	-1.88708700	-2.48796500	-3.34331100
H	-0.55149400	0.28178900	-3.22011100
H	-1.06013100	-0.65644400	-1.82778600
H	1.06212000	-1.54738600	-3.84044500
H	0.55595700	-2.46114300	-2.43905600
H	1.87055700	0.30839800	-2.37932000
H	1.32863000	-0.53883200	-0.95892000
H	3.46501800	-1.53941400	-2.91128300
H	2.92873500	-2.43331500	-1.50813100
H	4.25934000	0.32666500	-1.43299400
H	5.07348200	-1.19655900	-1.14193200
H	3.59901700	-1.61909000	0.87409300
H	3.39352200	0.26387500	2.25735400
H	4.72169300	2.23630700	1.39049500
H	4.07087000	1.98549700	-0.21587200
H	2.39308000	2.63530600	2.26638700
H	2.95525500	3.82088000	1.10750400
H	1.66828800	2.67564600	-0.71050000
H	1.07652300	1.50119300	0.44172700
H	-0.05752800	3.31174100	1.73897400
H	0.64438200	4.52160300	0.68807000
H	-1.65391900	4.22341500	0.04528500
H	-0.64540400	3.62011500	-1.25341700
H	-1.07391600	1.28104700	-0.55833200
H	-1.91987200	1.84827200	0.87442300
N	-0.18735900	-2.32118900	1.76326100

C	0.64376000	-3.11837100	0.81259900
H	0.01878100	-3.88495000	0.35798700
H	1.46573600	-3.58012000	1.35871400
H	1.03307000	-2.45314200	0.04643000
C	-0.81364300	-3.17685600	2.81428000
H	-1.46588100	-2.55470700	3.42400600
H	-1.39679600	-3.95846200	2.33023800
H	-0.03030400	-3.62094100	3.42756100
C	0.58066300	-1.18675800	2.35968700
H	-0.10279400	-0.58140300	2.95228300
H	1.01811100	-0.59315500	1.56005200
H	1.37389600	-1.58765100	2.98982500
H	-0.97665500	-1.89636000	1.22028800

conf_0

C	-1.15889500	-0.93733100	2.10563700
C	-2.59464000	-0.73151200	2.59826200
C	-3.57172200	-0.16666200	1.55979900
C	-3.77349900	-1.05011700	0.31360900
C	-2.61268100	-1.11883100	-0.64090700
O	-2.19824000	-2.14896800	-1.14369800
O	-2.01141900	0.02857800	-0.99475300
H	-2.36780100	0.79204900	-0.51694300
H	-4.01512300	-2.07530000	0.59579500
H	-4.62128200	-0.66984200	-0.26991500
H	-4.55225500	-0.03929800	2.02449800
H	-3.26232800	0.84589200	1.26943600
H	-2.98641000	-1.68458000	2.97167100
H	-2.58400800	-0.05185000	3.45652700
C	-0.20338900	-1.36997100	3.22270200
C	1.18054100	-1.80103700	2.72281800
C	1.96371900	-0.70239900	2.00005800
C	3.37106200	-1.13467600	1.58561100
C	4.14855500	-0.03976600	0.83298100
C	3.58984600	0.26164300	-0.52956300
C	3.08133400	1.41421400	-0.97358300
C	2.92883100	2.70740800	-0.22350100
C	1.51491100	3.30272700	-0.33391500
C	0.44354500	2.43005200	0.32350000
C	-0.96197700	3.03834800	0.31448100
C	-1.53252600	3.31425100	-1.08284200
C	-3.02776900	3.63919100	-1.06170500
H	-3.40813300	3.85387200	-2.06268800
H	-3.62000200	2.80856900	-0.65659000
H	-3.23225700	4.50960600	-0.43270800
H	-1.34756400	2.45094300	-1.73519800
H	-0.99141000	4.14718400	-1.53961600
H	-1.63800500	2.36559200	0.86201700
H	-0.96491100	3.96987400	0.89176600
H	0.73922900	2.22851000	1.35902800
H	0.41716700	1.45319900	-0.17490700
H	1.50991000	4.29356400	0.13264000
H	1.27534300	3.46051300	-1.39134100

H	3.18896700	2.58002800	0.82993100
H	3.64259700	3.43237000	-0.63303200
H	2.75884800	1.45814000	-2.01422600
H	3.64734500	-0.56781400	-1.23493200
H	5.18682600	-0.36929900	0.71216700
H	4.18423600	0.86241500	1.44777500
H	3.31512100	-2.03733300	0.96213500
H	3.93710400	-1.42343300	2.47686500
H	2.03835600	0.17566800	2.65351600
H	1.41609800	-0.35986000	1.11547100
H	1.06717100	-2.66856500	2.05785500
H	1.77013300	-2.15582200	3.57456500
H	-0.65517300	-2.20178300	3.77395600
H	-0.09424400	-0.55005800	3.94210500
H	-0.79313700	-0.01368500	1.64515300
H	-1.14110900	-1.70889600	1.32639400
N	0.24130500	-2.15510300	-2.34978300
C	0.14715100	-3.14655000	-3.46221300
H	1.13189600	-3.27756200	-3.90983700
H	-0.55510800	-2.77469800	-4.20618100
H	-0.21137100	-4.09236800	-3.06005500
C	1.13491900	-2.63295700	-1.25250900
H	0.74938500	-3.57766800	-0.87368700
H	2.14216500	-2.76743300	-1.64444400
H	1.14516500	-1.88859300	-0.46013600
C	0.64444900	-0.80240400	-2.83801700
H	1.63179200	-0.87028800	-3.29349500
H	0.67144100	-0.11811400	-1.99395100
H	-0.08583000	-0.46202800	-3.56962700
H	-0.72128000	-2.07471400	-1.93588600

conf_155

C	2.58419200	-2.58190200	0.65106000
C	1.74532500	-3.28614200	-0.44186800
C	0.35026700	-2.67978500	-0.63523200
C	0.42208900	-1.31212200	-1.32725400
C	-0.89305300	-0.59076200	-1.37038600
O	-1.97279600	-1.15795100	-1.44217200
O	-0.88740600	0.74662100	-1.34214200
H	-0.00190200	1.12431400	-1.16072600
H	1.16967700	-0.66906200	-0.86112000
H	0.74094500	-1.43831300	-2.36993000
H	-0.28247300	-3.34285500	-1.22872700
H	-0.13985800	-2.56812500	0.33769600
H	2.27547400	-3.26303500	-1.40012000
H	1.63464900	-4.34403000	-0.19248000
C	4.02515400	-2.25751100	0.23086600
C	4.14056100	-1.17307900	-0.84968600
C	3.73045800	0.22654600	-0.38282000
C	3.80712800	1.27793200	-1.49114800
C	3.26813900	2.65446300	-1.05656400
C	1.76771300	2.76104800	-1.06761600
C	0.94502800	2.90770000	-0.01990500

C	1.29827300	2.95925400	1.44036600
C	0.37548800	2.08693800	2.31021400
C	0.60068000	0.58644700	2.11004500
C	-0.35501000	-0.32201900	2.89052800
C	-1.81300600	-0.26018200	2.42145100
C	-2.71808600	-1.23786000	3.17334400
H	-3.76232700	-1.16033600	2.85433800
H	-2.39763300	-2.27175400	3.01785900
H	-2.69561600	-1.04361900	4.24875300
H	-1.84721400	-0.47954600	1.34624100
H	-2.19509000	0.75924900	2.54670300
H	-0.00536100	-1.35746000	2.80900500
H	-0.30989100	-0.07096400	3.95659800
H	1.63152800	0.34903000	2.39288300
H	0.52329300	0.34877000	1.04421500
H	0.53767700	2.33441000	3.36399700
H	-0.66582400	2.34971700	2.09116500
H	2.33938300	2.67050500	1.60190400
H	1.20916400	4.00028200	1.77430600
H	-0.11310500	3.07043000	-0.22793700
H	1.32504700	2.79506000	-2.06403700
H	3.64868000	3.41632000	-1.74549700
H	3.66890200	2.90587200	-0.07172000
H	3.25412700	0.93115600	-2.37366900
H	4.84779000	1.38622400	-1.81143600
H	4.37750900	0.53372000	0.44769900
H	2.71706100	0.21198800	0.02988600
H	3.54077000	-1.45304400	-1.72477600
H	5.17521600	-1.13452500	-1.20585800
H	4.50503300	-3.17474200	-0.12706800
H	4.59394600	-1.94156500	1.11229500
H	2.60872300	-3.21241800	1.54395500
H	2.08627300	-1.65962100	0.96568300
N	-4.29968800	0.14719300	-1.08153000
C	-5.12126800	-0.81452000	-0.28833500
H	-6.10347700	-0.38080600	-0.10175200
H	-4.61302900	-1.01275500	0.65335400
H	-5.22061000	-1.73993700	-0.85263300
C	-4.88545600	0.40241000	-2.43030100
H	-5.86329700	0.86971300	-2.31616300
H	-4.21815300	1.06195900	-2.98234300
H	-4.98285100	-0.54564600	-2.95624800
C	-4.05420700	1.41617800	-0.33451200
H	-3.37638300	2.03645800	-0.91689700
H	-3.59407200	1.17469800	0.62124600
H	-5.00310300	1.92876600	-0.17855600
H	-3.36262100	-0.31237100	-1.23833300

conf_55

C	2.47903900	-1.21339200	1.56896400
C	3.43827100	-1.83558300	0.54081000
C	2.71859200	-2.31695800	-0.73250800
C	2.64679900	-1.26289000	-1.86869400

C	2.14308400	0.07255100	-1.40370500
O	2.88382300	0.99284300	-1.09172000
O	0.82467700	0.27493600	-1.28677600
H	0.26058200	-0.49329700	-1.52434200
H	3.64653200	-1.09359800	-2.26897600
H	2.01182700	-1.63598300	-2.67728900
H	3.22924700	-3.18292400	-1.15865200
H	1.70882300	-2.65243200	-0.48251800
H	4.22261200	-1.12250400	0.27008100
H	3.94672100	-2.68406600	1.00712200
C	1.63268700	-2.26241100	2.31750300
C	0.21958700	-1.78495800	2.68089500
C	-0.72466500	-1.65152800	1.47632900
C	-1.09437000	-2.98760900	0.82492400
C	-1.95487200	-2.84129200	-0.44036800
C	-1.21150600	-2.27630600	-1.61954100
C	-1.62109600	-1.31786900	-2.46272300
C	-2.92748200	-0.57358100	-2.43921800
C	-2.79132100	0.94565300	-2.21902900
C	-2.37956100	1.35669200	-0.79988700
C	-3.39634400	1.02181300	0.29346900
C	-2.98175900	1.54380500	1.67188600
C	-3.96539100	1.16436200	2.77937100
H	-4.96348500	1.55868300	2.56999700
H	-4.05284400	0.07763100	2.87131000
H	-3.64972700	1.55595300	3.74934200
H	-2.88040200	2.63540100	1.62722800
H	-1.98776600	1.15072000	1.92069700
H	-4.37365500	1.44064000	0.02629400
H	-3.53660200	-0.06237600	0.35517700
H	-2.21070300	2.44109300	-0.79263500
H	-1.42250700	0.89106100	-0.54561100
H	-2.06711400	1.34510300	-2.93898400
H	-3.74990100	1.41646100	-2.45949600
H	-3.40784200	-0.73284500	-3.41117800
H	-3.60400800	-0.99882900	-1.69719700
H	-0.98032900	-1.08240900	-3.31356000
H	-0.26753500	-2.77596700	-1.84081900
H	-2.84093400	-2.24381900	-0.21615700
H	-2.31917400	-3.83556900	-0.72672700
H	-1.63773300	-3.59787300	1.55304100
H	-0.19269000	-3.55538600	0.57261200
H	-0.27817400	-0.98834900	0.72775200
H	-1.64651500	-1.15615000	1.79708500
H	0.28679400	-0.82002500	3.19829500
H	-0.22380400	-2.48080200	3.40078100
H	2.16225400	-2.56217100	3.22599200
H	1.55741100	-3.17235400	1.71490200
H	1.81564800	-0.50416400	1.06491700
H	3.05031500	-0.62472900	2.29369200
N	1.67693200	3.08273300	0.13915000
C	0.88556800	2.51115800	1.26921900
H	0.15729800	1.81390400	0.86393600
H	0.37856800	3.31885600	1.79622700
H	1.56272300	1.98953200	1.94299800

C	0.79401900	3.72824200	-0.87694600
H	0.25906300	4.55666100	-0.41330700
H	1.41133500	4.09359600	-1.69584700
H	0.09252200	2.98275200	-1.24401000
C	2.74982600	4.00285600	0.61730900
H	3.39217200	3.46280200	1.31065200
H	2.29490300	4.85771600	1.11713300
H	3.33409000	4.33702200	-0.23821800
H	2.15084800	2.27515000	-0.34617700

conf_138

C	2.31584400	2.46483600	0.61490800
C	1.17589700	2.44566100	1.64547900
C	1.31855000	1.39341300	2.75264500
C	0.96383300	-0.04082100	2.30626800
C	2.00071200	-0.67480500	1.42068100
O	3.18051600	-0.74956700	1.73262600
O	1.62815600	-1.19561500	0.24568900
H	0.66891900	-1.08370900	0.05130100
H	0.90204900	-0.68523100	3.18963600
H	-0.01362000	-0.05643700	1.82223100
H	2.33058700	1.39900400	3.16692300
H	0.64248100	1.64299000	3.57376900
H	1.12216400	3.43515800	2.10946700
H	0.21445100	2.30362200	1.14196700
C	2.01206200	3.30399000	-0.64313400
C	1.52028900	2.46729200	-1.83362400
C	0.24446900	1.66574300	-1.56960900
C	-0.16430900	0.76239600	-2.73312600
C	-1.37781100	-0.12657200	-2.40131800
C	-1.06934900	-1.23916200	-1.43782600
C	-1.52824000	-1.37473600	-0.18442200
C	-2.46757400	-0.43773500	0.52387500
C	-3.93846300	-0.86799500	0.37136600
C	-4.89985500	0.05843400	1.12030600
C	-6.37034900	-0.36901000	1.04147100
C	-6.97248100	-0.32085700	-0.36665900
C	-8.46412700	-0.66029200	-0.38132100
H	-8.64241600	-1.66592600	0.01128800
H	-9.03343400	0.04101700	0.23582500
H	-8.87327400	-0.62121400	-1.39383600
H	-6.43975600	-1.01499900	-1.02546700
H	-6.81930500	0.68091200	-0.78698700
H	-6.47569100	-1.38339500	1.44665900
H	-6.95909900	0.28138100	1.69804100
H	-4.79807600	1.07804400	0.72672900
H	-4.59768700	0.10508400	2.17333300
H	-4.18320300	-0.89327600	-0.69428200
H	-4.05636500	-1.89435300	0.73832100
H	-2.22427500	-0.40968300	1.59218500
H	-2.35079200	0.58326600	0.15002500
H	-1.28667500	-2.29509000	0.34698400
H	-0.44484000	-2.03677000	-1.84002400

H	-1.74306400	-0.58567200	-3.32638100
H	-2.19207400	0.49740000	-2.02540000
H	0.68287200	0.12747300	-3.02390800
H	-0.40001900	1.37560700	-3.60821200
H	-0.57782000	2.35362700	-1.33933400
H	0.37812800	1.04966900	-0.67686200
H	2.31934300	1.77178500	-2.12655400
H	1.36302500	3.12229300	-2.69703800
H	2.91151100	3.84385700	-0.95257200
H	1.27026200	4.07317400	-0.40077600
H	2.56000600	1.44697100	0.29373300
H	3.21553700	2.83897700	1.11362800
N	4.86725800	-1.48411800	-0.23167900
C	4.59143800	-0.49465300	-1.31516300
H	3.54161000	-0.56953600	-1.59047000
H	5.22721100	-0.71261600	-2.17289900
H	4.80049100	0.50496300	-0.93845100
C	4.52454700	-2.87354300	-0.65566700
H	3.47152700	-2.89909000	-0.92828200
H	5.14650000	-3.15486800	-1.50511300
H	4.70464200	-3.54946400	0.17853100
C	6.26610000	-1.38023100	0.27752000
H	6.43789300	-0.36366600	0.62679600
H	6.96147500	-1.62283700	-0.52578700
H	6.39216700	-2.07719300	1.10411900
H	4.21935600	-1.23908800	0.56647800

conf_111

C	-4.31583700	-1.99446800	-0.18947300
C	-3.93975400	-0.53272500	-0.44011900
C	-4.58554900	0.43354900	0.55387500
C	-4.19584200	1.90346100	0.30055700
C	-2.70935200	2.13385000	0.36327500
O	-2.04928400	2.60542900	-0.54393600
O	-2.07057700	1.79020900	1.49596500
H	-2.67862900	1.39572900	2.13689500
H	-4.52740800	2.22672900	-0.68586600
H	-4.66998000	2.55687500	1.04200400
H	-5.67485300	0.36324300	0.49777500
H	-4.32641200	0.13704400	1.57925500
H	-2.85046400	-0.42507200	-0.39045000
H	-4.22618400	-0.24727900	-1.45948500
C	-3.67617200	-2.97511900	-1.18380100
C	-2.14152200	-2.94016600	-1.23420000
C	-1.46678800	-3.26636800	0.10139400
C	0.06529000	-3.24954700	0.05237200
C	0.68508600	-1.87267700	-0.27558400
C	0.23270600	-0.78198800	0.65971400
C	0.83207100	-0.40658200	1.79438400
C	2.09992500	-0.96152400	2.37987600
C	3.27229300	0.04027100	2.32538000
C	3.78433600	0.31410700	0.90583800
C	4.56769800	-0.84271000	0.27990600

C	4.93113600	-0.59493300	-1.18606000
C	5.73240100	-1.73904600	-1.80879400
H	6.67396800	-1.89402700	-1.27467400
H	5.17231500	-2.67814000	-1.77060800
H	5.97344900	-1.53690600	-2.85513800
H	5.50155000	0.33863800	-1.26429200
H	4.00806200	-0.44069600	-1.76106100
H	5.48190800	-1.01484400	0.85964500
H	3.98906600	-1.77075100	0.34819500
H	4.42366200	1.20469800	0.91721100
H	2.93283300	0.55158900	0.25837600
H	2.95478900	0.98002700	2.79342000
H	4.09483600	-0.33903900	2.93994000
H	1.91143200	-1.21857000	3.42774000
H	2.38100300	-1.89234500	1.88413000
H	0.36009900	0.38506700	2.37600700
H	-0.70216700	-0.28813400	0.40548700
H	0.42461000	-1.59744900	-1.30338400
H	1.77292100	-1.96783600	-0.25184900
H	0.41813800	-3.97526200	-0.68846100
H	0.45359900	-3.57638300	1.02206200
H	-1.79649500	-4.25786900	0.43036300
H	-1.80215800	-2.56786000	0.87536200
H	-1.81123500	-1.95858800	-1.59209100
H	-1.80090400	-3.65789700	-1.98864200
H	-4.06816300	-2.77078800	-2.18646100
H	-4.00258200	-3.98915300	-0.93011600
H	-5.40484100	-2.09827700	-0.24315900
H	-4.03706700	-2.26694200	0.83441000
N	0.63410500	2.99683700	-0.84706500
C	1.34303500	3.09161700	0.46486700
H	2.39580600	3.30601300	0.28597200
H	1.23781300	2.14105500	0.98206800
H	0.88954600	3.89041700	1.04907700
C	0.67083500	4.28796100	-1.59608300
H	0.23740700	5.06855500	-0.97345300
H	1.70521600	4.53185500	-1.83627000
H	0.08812600	4.18124300	-2.50916300
C	1.14644200	1.86086800	-1.67259100
H	2.19232600	2.04182100	-1.91765500
H	0.55219800	1.79584400	-2.58236700
H	1.05162300	0.94471200	-1.09375100
H	-0.37323000	2.79900400	-0.64217600

conf_17

C	-1.06823900	-1.73591600	-1.23825200
C	-2.33315700	-2.60237400	-1.24031200
C	-3.57817300	-1.94949800	-0.62451200
C	-3.97237600	-0.59242100	-1.24015400
C	-3.07591700	0.56276400	-0.88405400
O	-2.62590400	1.35686700	-1.69061200
O	-2.77142800	0.75285800	0.41186200
H	-3.12374100	0.04933600	0.97712300

H	-4.00666000	-0.64863100	-2.32839700
H	-4.97736000	-0.31513200	-0.89881500
H	-4.43094900	-2.62152200	-0.74492100
H	-3.44381100	-1.85190700	0.46046100
H	-2.55892700	-2.90488700	-2.26869200
H	-2.13650300	-3.52786400	-0.68947100
C	0.21169200	-2.52710400	-1.51241800
C	1.45720700	-1.63937000	-1.55496300
C	2.75885200	-2.43681200	-1.66169300
C	4.03631900	-1.59642700	-1.77565000
C	4.38473200	-0.74818100	-0.53786600
C	3.62620800	0.54537200	-0.41844000
C	3.18593000	1.13867500	0.69386300
C	3.30659500	0.65743900	2.11249200
C	1.98130800	0.71870400	2.88961600
C	0.94604000	-0.30580600	2.42079500
C	-0.39787400	-0.18825600	3.14397800
C	-1.36552700	-1.32854100	2.81716000
C	-2.76292000	-1.12826900	3.40742500
H	-3.43741200	-1.94806300	3.14652900
H	-2.72525000	-1.07209800	4.49831100
H	-3.21815400	-0.18729000	3.07086100
H	-0.94868200	-2.27161200	3.18542500
H	-1.42863500	-1.44507100	1.72933400
H	-0.22982300	-0.16011600	4.22668300
H	-0.86700400	0.77223200	2.89086300
H	1.35276800	-1.31346400	2.56834900
H	0.79109800	-0.20854900	1.33848100
H	2.18221600	0.55926600	3.95368500
H	1.56383800	1.73192000	2.81334200
H	3.70408000	-0.36019100	2.14604700
H	4.04153200	1.28723100	2.62879100
H	2.71366300	2.11574400	0.59079400
H	3.48667900	1.07199400	-1.36237400
H	5.45105300	-0.49446400	-0.59493000
H	4.27152100	-1.35310000	0.36653400
H	3.97256100	-0.94387300	-2.65575300
H	4.87143000	-2.27612500	-1.96704200
H	2.69726000	-3.09765900	-2.53377100
H	2.84379800	-3.09600500	-0.78885400
H	1.48575600	-1.01700100	-0.65506900
H	1.38374700	-0.95112300	-2.40814100
H	0.11482100	-3.07477400	-2.45692100
H	0.33434100	-3.28567800	-0.73031400
H	-0.95886500	-1.23847300	-0.26773700
H	-1.16275500	-0.94199500	-1.98789500
N	-0.51112400	2.92975800	-1.02173700
C	-0.28463700	2.93423400	0.45420600
H	0.57693000	3.56125400	0.67957400
H	-0.10161000	1.91484200	0.78351400
H	-1.17447400	3.32390000	0.94426000
C	-0.81584100	4.29506000	-1.54354600
H	-1.68391600	4.68737000	-1.01674700
H	0.04550200	4.94189600	-1.37933000
H	-1.03366400	4.22360800	-2.60763200

C	0.62566900	2.29455800	-1.75456700
H	0.79958300	1.30104400	-1.34795500
H	1.51872300	2.90460900	-1.62475000
H	0.36587800	2.22777000	-2.80949600
H	-1.35941800	2.33957800	-1.21043800

conf_146

C	-0.00348700	0.56868100	2.43874600
C	0.55628700	1.84488500	3.07135300
C	1.21215400	2.82134300	2.08795700
C	0.22315600	3.54848600	1.15531300
C	-0.42399000	2.71583600	0.08293500
O	-1.61807700	2.72410600	-0.15982800
O	0.36754700	1.95573500	-0.69297100
H	1.28362700	1.95565500	-0.37843200
H	-0.58128300	4.00649800	1.73115200
H	0.74550300	4.35836600	0.63052800
H	1.74187600	3.59567100	2.64768100
H	1.98969700	2.30235000	1.50981900
H	-0.24030200	2.36869800	3.61287800
H	1.30072700	1.56660000	3.82384800
C	-0.65315200	-0.36578700	3.46427000
C	-1.07274200	-1.72636500	2.89567700
C	-2.14786500	-1.65880300	1.80446300
C	-2.59027300	-3.03892000	1.29826000
C	-1.53738400	-3.76107900	0.42807900
C	-1.44450100	-3.21402100	-0.97084200
C	-0.57237100	-2.31643000	-1.44034600
C	0.51554300	-1.61624100	-0.67874500
C	1.88263000	-1.65810600	-1.37379300
C	2.94311800	-0.83862300	-0.63591200
C	4.32672100	-0.88501700	-1.28469100
C	5.36665000	-0.02758700	-0.55905700
C	6.75081300	-0.08789400	-1.20690100
H	7.47066800	0.53409100	-0.66972100
H	7.13617800	-1.11145700	-1.21438300
H	6.71679000	0.26048900	-2.24345200
H	5.43573900	-0.35266300	0.48582900
H	5.02046800	1.01396900	-0.53309500
H	4.67265900	-1.92488200	-1.31642400
H	4.25072100	-0.55810700	-2.32925800
H	3.01578200	-1.18518600	0.40238300
H	2.61315100	0.21015300	-0.58009000
H	2.21469900	-2.69823600	-1.45682600
H	1.78498900	-1.28689300	-2.40159500
H	0.23301200	-0.56368900	-0.54467600
H	0.60422700	-2.02958300	0.32799500
H	-0.63615900	-2.06169300	-2.49849900
H	-2.18055700	-3.60840000	-1.66936800
H	-1.80858400	-4.81855000	0.36189200
H	-0.56437600	-3.72709600	0.92332100
H	-3.51815400	-2.94359700	0.72254200
H	-2.83266600	-3.67017700	2.15935300

H	-1.78460000	-1.06238900	0.96111900
H	-3.01918800	-1.12782700	2.20655200
H	-1.44783900	-2.34943500	3.71450900
H	-0.18247500	-2.23741800	2.51299600
H	-1.52524000	0.13323300	3.90406900
H	0.05035700	-0.53086900	4.28742100
H	0.80668200	0.03709700	1.92515200
H	-0.74439400	0.81882200	1.67329600
N	-2.90944300	1.23010300	-2.03412400
C	-1.95799900	0.86140900	-3.12435400
H	-1.16553800	0.24971600	-2.70035200
H	-1.53654500	1.77224900	-3.54556500
H	-2.49375300	0.30499900	-3.89280100
C	-3.96502700	2.17348600	-2.50969500
H	-3.48614300	3.06643400	-2.90737100
H	-4.55619400	1.68749000	-3.28527200
H	-4.59977600	2.44254700	-1.66746800
C	-3.49841100	0.02104400	-1.38145800
H	-4.14534300	-0.48618900	-2.09692200
H	-4.07509200	0.33808400	-0.51445000
H	-2.69550000	-0.64771200	-1.07688300
H	-2.35672300	1.74761300	-1.30773000

conf_26

C	4.05833700	-2.03183400	-0.02903800
C	2.70903700	-2.63058400	-0.45473000
C	1.64866900	-2.57134900	0.65487000
C	0.24914300	-3.01052100	0.21822400
C	-0.51910000	-2.07178500	-0.67674900
O	-1.59707500	-2.35539200	-1.16963600
O	-0.03421900	-0.84418300	-0.92508100
H	0.81195900	-0.67965800	-0.48289000
H	0.27482700	-3.97443700	-0.29847600
H	-0.40140900	-3.16206100	1.08613000
H	1.96270400	-3.22110800	1.47606200
H	1.59939200	-1.57078200	1.10234700
H	2.35137600	-2.12412700	-1.35958700
H	2.84721600	-3.67738800	-0.74237000
C	4.04328200	-0.50439400	0.13117200
C	3.98906000	0.26214300	-1.20003500
C	3.50550500	1.71149800	-1.05301500
C	1.99039500	1.80455500	-0.85640800
C	1.47230800	3.22613900	-0.58561300
C	-0.03028800	3.29752200	-0.58386600
C	-0.83810700	3.35025200	0.47879200
C	-0.45568900	3.35223100	1.93142900
C	-0.97347700	2.11033700	2.68303800
C	-0.26035200	0.81855000	2.27379200
C	-0.78780400	-0.44382700	2.96662400
C	-2.17066400	-0.89692800	2.48653400
C	-2.65053700	-2.17824300	3.16985300
H	-3.63046500	-2.49062200	2.80105100
H	-1.95312600	-3.00517000	3.00110600

H	-2.73148800	-2.03782400	4.25089400
H	-2.13949200	-1.05159800	1.40011600
H	-2.90161700	-0.10011100	2.65932200
H	-0.07710000	-1.26490700	2.81417800
H	-0.81838800	-0.27964800	4.04984400
H	0.80811500	0.93179900	2.49322400
H	-0.33901700	0.69734000	1.18758700
H	-0.84224300	2.26458300	3.75878700
H	-2.05275500	2.01717600	2.51706700
H	0.62830300	3.42177000	2.05231800
H	-0.88019800	4.24592900	2.40197700
H	-1.91112300	3.40663900	0.29594300
H	-0.49225000	3.30615500	-1.57076300
H	1.85911600	3.89675100	-1.36081900
H	1.88281500	3.58172900	0.36217700
H	1.68536000	1.17673600	-0.01034700
H	1.49320400	1.40158200	-1.74826700
H	3.78073300	2.28626500	-1.94300900
H	4.01850800	2.18776100	-0.20920600
H	3.33174500	-0.25291400	-1.91218700
H	4.98446900	0.24459100	-1.65334400
H	4.93772400	-0.19090500	0.67736200
H	3.20263500	-0.20949800	0.77039800
H	4.81879000	-2.30612400	-0.76700600
H	4.36715000	-2.49712500	0.91316000
N	-2.90549000	-0.18220300	-2.19202200
C	-3.05968600	0.75067500	-1.03490800
H	-3.61108600	1.63193800	-1.36187600
H	-2.07203500	1.03854100	-0.68136500
H	-3.60436100	0.24056900	-0.24275000
C	-4.22345100	-0.65670800	-2.70835400
H	-4.76734000	-1.13384500	-1.89509500
H	-4.78788000	0.19453300	-3.08794200
H	-4.05070600	-1.37713400	-3.50594300
C	-2.06424500	0.42110800	-3.26811100
H	-1.93438900	-0.30966400	-4.06458200
H	-1.09822000	0.68110900	-2.84151100
H	-2.56238600	1.31006100	-3.65400500
H	-2.39230300	-1.02791200	-1.83681300

conf_116

C	-3.25784700	1.89632100	-1.45646700
C	-1.73644900	1.89742500	-1.28166600
C	-1.08677500	3.25532200	-1.55064900
C	0.45185800	3.22271700	-1.40235700
C	0.85659700	2.66554800	-0.06516900
O	1.41335500	1.59727900	0.09052600
O	0.53308000	3.37763700	1.03465600
H	0.08638600	4.20344900	0.80208100
H	0.89662000	2.58108200	-2.16243900
H	0.86946700	4.22813300	-1.51945200
H	-1.31543300	3.59360500	-2.56451200
H	-1.51717700	4.00863100	-0.87941600

H	-1.49173900	1.57956500	-0.26152400
H	-1.29383500	1.14574500	-1.94585300
C	-3.90121800	0.57737300	-1.00626600
C	-3.94735200	0.41215900	0.51806300
C	-4.39561400	-0.97662000	0.99420700
C	-3.50155000	-2.13848600	0.53929100
C	-2.02115600	-1.98515400	0.93677700
C	-1.19210000	-3.17564700	0.53233800
C	-0.56270100	-3.33293400	-0.63629500
C	-0.51891200	-2.35856200	-1.77988300
C	0.90017700	-2.11759000	-2.32186900
C	1.85330200	-1.57487500	-1.25677900
C	3.22180200	-1.15248700	-1.79015700
C	4.16755600	-0.65439200	-0.69296900
C	5.52999500	-0.20693300	-1.22511700
H	5.42038700	0.61265100	-1.94115600
H	6.18521600	0.13813200	-0.42107700
H	6.03862900	-1.02743100	-1.73845200
H	4.31264300	-1.45537100	0.04451300
H	3.68862800	0.18186000	-0.16762300
H	3.68900500	-1.99446800	-2.31441200
H	3.09249400	-0.36132900	-2.53871300
H	1.98745200	-2.34105600	-0.48495700
H	1.38273200	-0.71265600	-0.77032700
H	1.30071000	-3.05199400	-2.73103200
H	0.84405100	-1.41509700	-3.16058900
H	-1.14421800	-2.74885900	-2.59240500
H	-0.96293900	-1.40211200	-1.48727800
H	-0.04152700	-4.27288800	-0.80993100
H	-1.17212000	-4.00362700	1.23724200
H	-1.96595500	-1.84942700	2.02490900
H	-1.62049500	-1.07505600	0.47916300
H	-3.55509200	-2.26136900	-0.54623300
H	-3.88625500	-3.07116400	0.96303900
H	-5.41642200	-1.16468500	0.64459800
H	-4.44684800	-0.97245400	2.08930500
H	-4.62843500	1.16412900	0.93124700
H	-2.96424300	0.63861200	0.94720500
H	-3.35210200	-0.25320100	-1.46293700
H	-4.92153400	0.51474100	-1.39720100
H	-3.49459600	2.08425100	-2.50860000
H	-3.69467100	2.72752200	-0.88917200
N	1.49892100	0.14988500	2.42928900
C	0.19731600	0.53240600	3.05617000
H	-0.61082200	0.10037400	2.46929200
H	0.11886700	1.61771900	3.05770100
H	0.16604600	0.14570600	4.07413000
C	2.66040400	0.73685500	3.16313400
H	3.57396300	0.47976300	2.63017800
H	2.54229000	1.81843400	3.19501900
H	2.68678600	0.32984100	4.17330900
C	1.62705000	-1.33217000	2.27827500
H	1.59704700	-1.78669900	3.26826800
H	2.57404100	-1.55057500	1.79100300
H	0.80829200	-1.70536400	1.66571400

H	1.50448600	0.59277600	1.48523000

conf_91

C	-4.47609100	1.08670800	0.63749100
C	-5.67642900	0.51690300	-0.12834100
C	-5.38016200	-0.69028400	-1.02959300
C	-4.88548100	-1.92860900	-0.26373400
C	-3.41841400	-1.90689900	0.07668600
O	-2.55478100	-1.46324200	-0.65545500
O	-3.02571700	-2.46869000	1.23881000
H	-3.78300600	-2.77356600	1.75667000
H	-5.48394900	-2.08894700	0.63908800
H	-5.01482500	-2.82264100	-0.88659700
H	-6.29769500	-0.97029700	-1.55185100
H	-4.64892600	-0.43979500	-1.80121700
H	-6.45697500	0.23390300	0.58712200
H	-6.11029800	1.30753200	-0.74894400
C	-3.41138500	1.73966000	-0.24782600
C	-2.16641600	2.15840300	0.53531700
C	-1.09323000	2.82709300	-0.32578000
C	0.20186900	3.11605800	0.43588000
C	1.31602700	3.70392000	-0.44632500
C	2.55635200	4.01711900	0.34180100
C	3.72028400	3.36355600	0.31314100
C	4.06733000	2.15969100	-0.51637300
C	4.11450800	0.86992000	0.32020000
C	4.50352700	-0.36703300	-0.49197400
C	4.42459900	-1.66957400	0.30668100
C	4.66431300	-2.92688600	-0.53203700
C	4.56299600	-4.21957900	0.27928900
H	4.73134600	-5.09958500	-0.34544200
H	5.30259700	-4.23716400	1.08428100
H	3.57601600	-4.32603700	0.74408700
H	5.65292100	-2.86355200	-0.99845000
H	3.94695000	-2.95153900	-1.36341500
H	5.14549900	-1.63661900	1.13132800
H	3.43865800	-1.74240000	0.79060000
H	5.51896300	-0.24240700	-0.88331800
H	3.86131100	-0.44171200	-1.38097700
H	4.81551500	0.99716800	1.15227300
H	3.13060400	0.72048900	0.78467400
H	3.35506000	2.02981300	-1.33683900
H	5.04715100	2.31257400	-0.98306800
H	4.50996500	3.71112900	0.97559400
H	2.46724100	4.85923000	1.02473900
H	0.94378400	4.62190000	-0.91592500
H	1.53455500	3.00945300	-1.26321900
H	0.57731400	2.19608300	0.90421500
H	-0.00749000	3.80092700	1.26489800
H	-1.48802000	3.76196100	-0.73846900
H	-0.87876100	2.19397500	-1.19795100
H	-1.74297900	1.26779100	1.02180000
H	-2.44914100	2.83234800	1.35217100

H	-3.84610800	2.61558900	-0.74269600
H	-3.11144500	1.05232500	-1.04486600
H	-4.01016000	0.30246000	1.25072100
H	-4.84204800	1.82588800	1.35747500
N	0.11442200	-1.49471300	-0.05320100
C	0.57306300	-2.88629900	-0.34447400
H	0.04728400	-3.57311000	0.31640900
H	1.64706000	-2.95078800	-0.17765500
H	0.34093500	-3.12102800	-1.38174400
C	0.32085800	-1.13496800	1.38196700
H	-0.05058300	-0.12558900	1.54566300
H	-0.23504300	-1.83827300	1.99893900
H	1.38404900	-1.18239800	1.61169100
C	0.74427800	-0.50110500	-0.97429400
H	0.49658500	-0.77125000	-1.99925000
H	1.82193800	-0.51524300	-0.83071000
H	0.34951200	0.48554000	-0.74690100
H	-0.91671400	-1.47243800	-0.23557600

conf_121

C	-3.24165600	-1.69760600	0.11208600
C	-4.74031500	-1.59277900	0.40391700
C	-5.44494000	-0.39846500	-0.25054600
C	-5.02179100	0.97663200	0.30259300
C	-3.63198500	1.43964800	-0.04379900
O	-2.85806100	1.92729900	0.76030800
O	-3.23711600	1.35433800	-1.32611700
H	-3.90553300	0.91229000	-1.86859400
H	-5.69999000	1.74925400	-0.08114900
H	-5.10037600	0.99543500	1.38991300
H	-5.31381300	-0.43980700	-1.34147300
H	-6.52243800	-0.48497500	-0.09234200
H	-5.23440400	-2.50883800	0.06551300
H	-4.89828900	-1.54541600	1.48781500
C	-2.58257600	-2.91021800	0.77575600
C	-1.10804100	-3.09227900	0.39726500
C	-0.21064200	-1.91176700	0.78582300
C	1.28659800	-2.16610200	0.57043700
C	1.69143600	-2.49997400	-0.88011300
C	1.27635600	-1.45614000	-1.87864000
C	2.07054500	-0.62504900	-2.56067900
C	3.56794500	-0.51797400	-2.50102100
C	4.04366500	0.81501500	-1.88762400
C	3.71779700	0.96924300	-0.39753600
C	4.51097800	0.04518900	0.52964400
C	4.19893900	0.26949100	2.01169500
C	4.96025200	-0.68149800	2.93597200
H	4.71127400	-1.72400000	2.71685100
H	6.04060600	-0.56660000	2.81213200
H	4.72450000	-0.49543300	3.98652800
H	3.12039200	0.14637300	2.17696400
H	4.43428600	1.30770100	2.27652100
H	4.30627900	-1.00098500	0.27844500

H	5.58361100	0.19428100	0.35977000
H	2.64909400	0.78061800	-0.24782500
H	3.90345500	2.00895200	-0.09831800
H	5.12484500	0.90802200	-2.03177100
H	3.59110100	1.64103300	-2.44990700
H	4.00126200	-1.35456000	-1.95075100
H	3.96089000	-0.58526900	-3.52121900
H	1.59511900	0.06614700	-3.25682200
H	0.20671600	-1.39482300	-2.07320200
H	1.24282200	-3.45755000	-1.16530900
H	2.77154200	-2.65228800	-0.91050300
H	1.60454200	-2.98978400	1.21843500
H	1.85207800	-1.28671700	0.89514000
H	-0.37889500	-1.67270300	1.84352200
H	-0.51348900	-1.02356100	0.21786000
H	-1.03803800	-3.27069200	-0.68154500
H	-0.72679300	-3.99862500	0.87998200
H	-3.13540300	-3.81444700	0.50006700
H	-2.67226700	-2.81589400	1.86478700
H	-2.73073200	-0.79460000	0.45797200
H	-3.08354000	-1.75054600	-0.97360100
N	-0.25327500	2.61390700	0.35067700
C	0.21598900	2.17744500	-0.99965300
H	-0.45993500	2.58435100	-1.74936200
H	0.21368900	1.09076800	-1.04640000
H	1.22895000	2.54234800	-1.15976000
C	0.50335900	1.94407400	1.45180300
H	1.55180900	2.23035900	1.39335500
H	0.07795600	2.25320300	2.40489500
H	0.40735600	0.86688200	1.33539000
C	-0.23123200	4.10019600	0.48940500
H	-0.83025800	4.53644600	-0.30801600
H	0.79811500	4.44992800	0.41747300
H	-0.65269200	4.37011900	1.45602100
H	-1.25513300	2.32066900	0.44353900

conf_71

C	0.94171800	-3.27151200	-1.45321400
C	1.73677600	-1.99878300	-1.12638000
C	2.15762500	-1.22658200	-2.38994900
C	2.40821300	0.26553100	-2.15783900
C	1.20136500	1.07493400	-1.75158600
O	1.27278800	2.14852900	-1.18136600
O	-0.02286700	0.61764300	-2.06535000
H	0.01208700	-0.27010400	-2.45370400
H	3.16840700	0.43553800	-1.39325300
H	2.78604800	0.74151400	-3.07107600
H	3.06925800	-1.66748300	-2.79953700
H	1.41450300	-1.35672600	-3.18866000
H	1.13274000	-1.36065900	-0.47253200
H	2.63175000	-2.24282600	-0.54868600
C	-0.51615700	-2.97951400	-1.83994900
C	-1.42706900	-2.68850000	-0.64003500

C	-2.74502100	-2.00265700	-1.02876400
C	-3.89291300	-2.22843200	-0.03680900
C	-3.58796800	-1.84387000	1.42155000
C	-3.37640300	-0.37430100	1.64486200
C	-2.31904100	0.21887900	2.20747900
C	-1.05279700	-0.43042400	2.68627600
C	0.20275100	0.20235100	2.06829600
C	1.49130300	-0.54184500	2.41997900
C	2.74758600	0.13127000	1.86650600
C	4.02483700	-0.68421100	2.07557000
C	5.26432200	-0.01507200	1.48011500
H	6.16213100	-0.61461900	1.64620200
H	5.15764400	0.12713600	0.39922400
H	5.43468800	0.96921900	1.92583700
H	3.89343700	-1.67828600	1.63123700
H	4.17569700	-0.85138400	3.14767300
H	2.61367000	0.31991100	0.79508000
H	2.86639200	1.11934300	2.32806500
H	1.42615200	-1.56717700	2.03566700
H	1.57406600	-0.63327200	3.50893100
H	0.08752500	0.23348600	0.97717200
H	0.28842900	1.24545400	2.39986000
H	-0.98694300	-0.34499800	3.77802600
H	-1.06093700	-1.49968800	2.46493700
H	-2.37089100	1.29681600	2.36343200
H	-4.21537800	0.25699700	1.35304600
H	-2.73642000	-2.42039900	1.78683000
H	-4.44341100	-2.15274100	2.03435000
H	-4.16607600	-3.28883200	-0.05688700
H	-4.77753500	-1.67973600	-0.37888300
H	-3.06907200	-2.37295100	-2.00789100
H	-2.56330400	-0.92754700	-1.15336800
H	-0.89771700	-2.07175900	0.09329300
H	-1.63719800	-3.63875600	-0.13623700
H	-0.93042200	-3.82277600	-2.40017100
H	-0.55708900	-2.13859200	-2.55081700
H	0.94886400	-3.94224200	-0.58867100
H	1.44614400	-3.81165600	-2.26106300
N	-1.03019400	3.46849600	-0.52916400
C	-0.87278000	3.91453700	0.88704400
H	-1.73106900	4.52455200	1.16745800
H	0.04435500	4.49454200	0.97285900
H	-0.81321800	3.03458700	1.52303600
C	-2.22674400	2.58978500	-0.70498800
H	-3.12273900	3.16498600	-0.47233600
H	-2.25487500	2.24354500	-1.73546100
H	-2.14417500	1.73569100	-0.03517800
C	-1.04629800	4.62383300	-1.47474500
H	-1.10709200	4.24250400	-2.49244100
H	-0.12748000	5.19401000	-1.34996600
H	-1.91034800	5.25166600	-1.25916800
H	-0.17845500	2.90236400	-0.76888400

conf_115

C	0.88196500	2.38293400	-0.45015000
C	-0.60005400	2.45350300	-0.07748200
C	-1.48310200	2.93676000	-1.22983800
C	-2.98412300	2.91326500	-0.89585800
C	-3.55131800	1.53570500	-0.67673400
O	-4.27510900	1.23256100	0.25525700
O	-3.27051100	0.58920200	-1.58748700
H	-2.65221100	0.91963300	-2.25490100
H	-3.19379500	3.49254900	0.00341900
H	-3.56230300	3.36165100	-1.71349500
H	-1.21535300	3.96187400	-1.49829700
H	-1.27524200	2.34577000	-2.13394400
H	-0.93360700	1.46549300	0.26240200
H	-0.73341100	3.12828100	0.77587100
C	1.78110600	1.97877700	0.71940000
C	3.26161100	1.89558500	0.34427300
C	4.15327900	1.51029400	1.52708900
C	5.65051300	1.48854100	1.19882500
C	6.07180500	0.38066800	0.21639100
C	5.84230900	-0.99721800	0.77025800
C	5.27712700	-2.04527400	0.16720900
C	4.72403800	-2.12721300	-1.22800200
C	3.29129300	-2.68548200	-1.26542900
C	2.25760500	-1.74020400	-0.65173200
C	0.85323300	-2.34270200	-0.58029600
C	-0.19107500	-1.35815500	-0.05240900
C	-1.59052000	-1.96054200	0.08010300
H	-1.94974800	-2.33204800	-0.88509000
H	-1.58997900	-2.80065700	0.78174700
H	-2.30185700	-1.20996600	0.43873900
H	-0.22967700	-0.49051600	-0.72066200
H	0.13220100	-0.97585600	0.92192400
H	0.55104000	-2.69225500	-1.57590700
H	0.87305700	-3.23198200	0.06153400
H	2.22715700	-0.81412100	-1.23987800
H	2.58213600	-1.44982300	0.35361900
H	3.01407100	-2.90250600	-2.30306100
H	3.26836100	-3.64484700	-0.73391900
H	4.75281000	-1.15181300	-1.72205000
H	5.36871700	-2.78845300	-1.82006700
H	5.21615200	-2.97525500	0.73130600
H	6.20576300	-1.14241800	1.78680400
H	7.14243200	0.50086600	0.00831200
H	5.56398900	0.50858900	-0.74250300
H	6.21616600	1.36256300	2.12860200
H	5.94641500	2.46107000	0.78974900
H	3.84985600	0.52612700	1.90332000
H	3.98113700	2.21985500	2.34454300
H	3.58956400	2.86346100	-0.05542500
H	3.38442100	1.17063700	-0.46709500
H	1.45371600	1.00933000	1.11245800
H	1.65420300	2.69891600	1.53700000
H	1.20474700	3.35747200	-0.83571200
H	1.01530900	1.67045600	-1.27412600

N	-5.55998800	-1.11348200	0.75477300
C	-5.02931900	-1.64547700	2.04640300
H	-3.98844800	-1.92941000	1.90701600
H	-5.61845600	-2.51259900	2.34365700
H	-5.10230300	-0.86586200	2.80247400
C	-5.40178700	-2.09754400	-0.35821600
H	-5.99675500	-2.98324700	-0.13743600
H	-5.74314800	-1.63741100	-1.28354000
H	-4.35014800	-2.35984500	-0.44625900
C	-6.97400500	-0.64932600	0.88879800
H	-7.02154800	0.10986400	1.66715300
H	-7.60595500	-1.49739700	1.15062400
H	-7.29480900	-0.22274200	-0.05977900
H	-4.99328000	-0.26453200	0.50967800

conf_84

C	2.16670500	3.17886500	-0.01717700
C	0.98507900	2.35598700	0.50447800
C	-0.06007500	3.23175200	1.20721500
C	-1.29372200	2.48131000	1.71795800
C	-2.24226600	1.92897200	0.68414000
O	-3.13310800	1.14485000	0.95589100
O	-2.12863900	2.33198200	-0.59383500
H	-1.38511400	2.94346000	-0.69958100
H	-1.01170200	1.63554800	2.35050400
H	-1.90776400	3.13145400	2.35196100
H	0.40784300	3.72097800	2.06554600
H	-0.36321800	4.06458700	0.55474600
H	0.53670400	1.80435100	-0.33214800
H	1.33330000	1.59235500	1.20639600
C	3.20392900	2.37194300	-0.80483900
C	3.96222900	1.32493100	0.01629400
C	5.08876000	0.66796500	-0.78621400
C	5.91955300	-0.35113400	0.00089800
C	5.13135600	-1.58906300	0.47134800
C	4.52501000	-2.34741500	-0.67414300
C	3.24505700	-2.67957500	-0.85502600
C	2.08800500	-2.39021500	0.05815900
C	1.01741500	-1.50686700	-0.60440500
C	-0.20571400	-1.25173400	0.28490300
C	-1.07842700	-2.48726200	0.52540400
C	-2.24726200	-2.23609500	1.48121000
C	-3.18399500	-3.43795200	1.61462900
H	-4.00675600	-3.23556400	2.30564200
H	-3.61368900	-3.72210800	0.64699800
H	-2.64867600	-4.31326200	1.99133100
H	-2.80749700	-1.35170400	1.15590000
H	-1.85131600	-1.97115700	2.46758300
H	-1.45674400	-2.85288600	-0.43997300
H	-0.46981700	-3.30375100	0.92487800
H	-0.82250900	-0.46706300	-0.17204900
H	0.12621400	-0.85121900	1.25117500
H	1.47834400	-0.55229200	-0.87768700

H	0.69479800	-1.96995800	-1.54531700
H	1.63704000	-3.34353200	0.35697600
H	2.42613600	-1.91020800	0.98042800
H	2.98833100	-3.22991600	-1.75861300
H	5.23262400	-2.64641800	-1.44593000
H	4.36964000	-1.29567500	1.19702400
H	5.82317100	-2.24988900	1.00731800
H	6.36760100	0.13598400	0.87408500
H	6.75364800	-0.68375400	-0.62637900
H	5.75690800	1.45181900	-1.15980800
H	4.66083200	0.18111700	-1.66995000
H	3.26863500	0.55698400	0.37358900
H	4.38319000	1.80174400	0.91110400
H	3.92794800	3.07109400	-1.23638300
H	2.71536000	1.88105300	-1.65664300
H	2.65826400	3.67487100	0.82825400
H	1.78169700	3.98220700	-0.65737300
N	-4.59951400	-0.05930200	-0.99424100
C	-3.61996300	-0.78146600	-1.86041000
H	-4.15267500	-1.25295600	-2.68569500
H	-3.11143600	-1.53396200	-1.26219200
H	-2.89704000	-0.06217000	-2.24044600
C	-5.29163900	1.03471000	-1.73885000
H	-5.85882500	0.60444700	-2.56360000
H	-5.96147700	1.55460300	-1.05616000
H	-4.54030200	1.72629100	-2.11515700
C	-5.56672500	-0.99609500	-0.34715100
H	-6.21124800	-0.42792000	0.32115600
H	-5.01006800	-1.73834300	0.22075200
H	-6.16322300	-1.48225300	-1.11871100
H	-4.04641500	0.40409400	-0.23084500

conf_182

C	-4.81434500	-0.82551300	0.13031400
C	-4.62723300	0.47920100	-0.65286000
C	-4.79066300	1.74380500	0.20145000
C	-3.64725000	1.98519400	1.19848200
C	-2.29214900	2.21316200	0.58008000
O	-1.24622600	1.84498700	1.07923900
O	-2.22935200	2.89741800	-0.57983200
H	-3.11663300	3.10921600	-0.90475900
H	-3.85606700	2.87590200	1.80440800
H	-3.53680600	1.15660100	1.89755900
H	-4.92206100	2.62392500	-0.44483700
H	-5.72165200	1.68351500	0.77162300
H	-3.64664500	0.48480800	-1.14508200
H	-5.36590300	0.50869400	-1.45994100
C	-4.66243900	-2.07743700	-0.74689900
C	-3.21366500	-2.42799600	-1.11752600
C	-2.42120400	-3.03721400	0.04438600
C	-0.99311100	-3.45749900	-0.32112900
C	-0.04146600	-2.29382900	-0.67396300
C	0.10170000	-1.29877400	0.44677800

C	1.03196200	-1.31946900	1.40727600
C	2.14325900	-2.31881700	1.57823500
C	3.53856000	-1.76685400	1.22353000
C	3.76246000	-1.61388600	-0.28422400
C	5.15062800	-1.08731800	-0.66897100
C	5.38361700	0.39267500	-0.35011600
C	6.78673200	0.87053500	-0.72774400
H	6.97779200	0.72495600	-1.79476400
H	6.92680700	1.93136600	-0.50426700
H	7.54919600	0.31269600	-0.17745300
H	5.21384000	0.57664500	0.71688000
H	4.63782700	0.98913200	-0.89370500
H	5.91625400	-1.68858200	-0.16448500
H	5.30704300	-1.23999300	-1.74257500
H	2.99190600	-0.96019900	-0.71202000
H	3.61338400	-2.59349700	-0.75129700
H	3.69376200	-0.81136600	1.73892300
H	4.29788300	-2.44756600	1.62194500
H	2.14924400	-2.63985900	2.62515200
H	1.95285700	-3.21557500	0.98310900
H	0.97474900	-0.55347100	2.18017900
H	-0.66128200	-0.52720100	0.51073600
H	-0.41025300	-1.78228100	-1.56997600
H	0.93232800	-2.71093800	-0.93961300
H	-1.02485800	-4.15251600	-1.16751600
H	-0.55948200	-4.00729700	0.51992900
H	-2.96056800	-3.92074400	0.40239300
H	-2.38865600	-2.34306200	0.89124200
H	-2.70230100	-1.53737900	-1.50206200
H	-3.22301000	-3.14502100	-1.94561500
H	-5.25205800	-1.93790600	-1.65969700
H	-5.10621500	-2.93339800	-0.22836000
H	-5.81448700	-0.81705500	0.57665800
H	-4.10907700	-0.87599500	0.96694600
N	1.16309800	2.39730300	-0.13838400
C	2.27050800	2.10616000	0.82143500
H	2.08245400	2.65008200	1.74558900
H	3.21482500	2.42220500	0.38198300
H	2.28682500	1.03649000	1.01285100
C	1.06590600	3.85467800	-0.44791600
H	0.19133600	4.01963200	-1.07353100
H	0.95991400	4.40506800	0.48543500
H	1.97121100	4.16971800	-0.96552200
C	1.27493200	1.57338900	-1.37994100
H	1.27533100	0.52375200	-1.09375300
H	2.20098100	1.82915400	-1.89340700
H	0.41868800	1.78903500	-2.01668900
H	0.26407800	2.13278300	0.32676800

conf_160

C	-1.80623200	-0.89727200	2.84345100
C	-0.42779800	-1.12553900	3.48008800
C	0.78361500	-0.93785400	2.55341400

C	1.02448200	0.51203700	2.09249800
C	0.09858700	1.06923100	1.04933700
O	-0.42912700	2.16951000	1.14027400
O	-0.12837900	0.37474400	-0.07091000
H	0.27421900	-0.51589100	-0.09932800
H	0.98779100	1.19684100	2.94020000
H	2.02842800	0.58939100	1.65956000
H	1.68223200	-1.24487200	3.09413700
H	0.72209300	-1.61217700	1.69254500
H	-0.31227900	-0.46005600	4.34221800
H	-0.39051000	-2.14526000	3.87720800
C	-2.09077200	-1.83212400	1.66869700
C	-3.46296600	-1.65169500	1.01297300
C	-3.51434800	-2.23719900	-0.40675900
C	-2.69719100	-1.41356300	-1.43488800
C	-1.57055200	-2.16654800	-2.17353600
C	-0.45332100	-2.65549000	-1.29556400
C	0.85207100	-2.38915600	-1.43949400
C	1.49630100	-1.54870500	-2.51834400
C	2.95691600	-1.19036100	-2.21442400
C	3.13679300	-0.33420300	-0.95932300
C	4.59187300	0.04184500	-0.67502800
C	4.77055100	0.85412600	0.61011600
C	6.22348600	1.25373300	0.87209500
H	6.32062100	1.82746400	1.79681900
H	6.86340800	0.37108000	0.95840900
H	6.61460700	1.86754500	0.05574100
H	4.39758800	0.26802500	1.46025100
H	4.14757400	1.75653500	0.55751000
H	5.19592900	-0.87093900	-0.61191200
H	4.99006800	0.61173300	-1.52292400
H	2.73858600	-0.87626500	-0.09125800
H	2.53899500	0.58179700	-1.05896000
H	3.53683600	-2.11429100	-2.10694700
H	3.38096500	-0.66287400	-3.07460400
H	1.45717900	-2.09988000	-3.46534700
H	0.92057600	-0.63054800	-2.68602500
H	1.54054800	-2.88908900	-0.76019700
H	-0.73895400	-3.33915400	-0.50119900
H	-1.17197200	-1.52828200	-2.96637400
H	-2.00998700	-3.03663200	-2.67806400
H	-2.25058400	-0.55217300	-0.92916000
H	-3.37540800	-1.01109300	-2.19394100
H	-4.55458700	-2.29787900	-0.73579600
H	-3.15442200	-3.27131000	-0.37867200
H	-3.71178000	-0.58263900	0.96687800
H	-4.23473200	-2.11047400	1.63823600
H	-1.98401100	-2.87329200	1.99573900
H	-1.32488400	-1.68623900	0.90751200
H	-1.90394700	0.14501100	2.51369600
H	-2.57079200	-1.03070800	3.61583000
N	-2.11576100	3.02145500	-0.78154600
C	-3.45635600	2.48013200	-0.40979600
H	-3.41598000	1.39344100	-0.44406000
H	-4.20137000	2.84954700	-1.11417200

H	-3.69979600	2.80885100	0.59897200
C	-1.67122400	2.52400200	-2.11680600
H	-2.36980800	2.86951600	-2.87849000
H	-0.67467000	2.91153500	-2.32073900
H	-1.64174400	1.43737800	-2.08976600
C	-2.07618900	4.51141900	-0.69813900
H	-1.06482500	4.84875000	-0.91769800
H	-2.35093000	4.81374400	0.31077500
H	-2.77621200	4.93169800	-1.41991400
H	-1.43003000	2.65701900	-0.06419200

conf_124

C	2.52228700	-3.41791100	-1.00136900
C	3.46701200	-2.23767100	-0.72539300
C	3.47942600	-1.78914700	0.74938400
C	3.84531600	-0.31581400	0.94466400
C	2.85094400	0.68784000	0.41521600
O	3.10972200	1.86714500	0.24979200
O	1.59810800	0.28665000	0.14054000
H	1.47350400	-0.66089500	0.30303500
H	3.95656200	-0.07726800	2.00902300
H	4.80511900	-0.07169000	0.48275500
H	2.51541900	-1.99283700	1.23236900
H	4.19318300	-2.39605600	1.31077500
H	4.48524700	-2.50526100	-1.01932000
H	3.19131000	-1.39768700	-1.37279300
C	1.03201300	-3.06831700	-0.86978200
C	0.50294200	-2.11434400	-1.94913000
C	-0.83101400	-1.46292700	-1.57961000
C	-1.35868900	-0.51762500	-2.66013800
C	-2.55499700	0.32941600	-2.19646700
C	-3.75162000	-0.50732200	-1.84217800
C	-4.47436100	-0.47104100	-0.72138800
C	-4.30373300	0.42060800	0.47587400
C	-4.23943500	-0.37005400	1.79635500
C	-2.93639900	-1.15094700	2.00939700
C	-1.74302500	-0.26507400	2.38110700
C	-0.47516300	-1.05390000	2.71880400
C	0.63945600	-0.18586200	3.30761100
H	0.33174300	0.24140000	4.26545200
H	1.55083500	-0.76354800	3.48748300
H	0.89081900	0.64803600	2.64546200
H	-0.12522800	-1.57495500	1.81859300
H	-0.72507000	-1.84556800	3.43275600
H	-1.52666400	0.43440200	1.56258300
H	-2.01676000	0.35329300	3.24515900
H	-2.70382500	-1.73405900	1.11090400
H	-3.08771100	-1.87614600	2.81595700
H	-4.38563000	0.31741300	2.63684400
H	-5.08481500	-1.06517500	1.82241600
H	-3.42336400	1.06150100	0.37266600
H	-5.16625300	1.09667200	0.52372000
H	-5.31552800	-1.15840600	-0.64988300

H	-4.05008000	-1.21732900	-2.61162100
H	-2.82916700	1.02092200	-3.00362000
H	-2.25627300	0.94850800	-1.34349400
H	-1.64462300	-1.09646000	-3.54525600
H	-0.54972800	0.14649700	-2.99057600
H	-0.71592700	-0.90065200	-0.64280400
H	-1.58008500	-2.23159600	-1.36594900
H	0.40066900	-2.66151000	-2.89238600
H	1.23292700	-1.32082700	-2.14717000
H	0.83693800	-2.64273500	0.12570100
H	0.44194000	-3.98920700	-0.88590900
H	2.76516200	-4.23807900	-0.31755900
H	2.71164800	-3.79637000	-2.01070000
N	0.94957100	3.38922600	-0.46232000
C	0.42790500	2.81360800	-1.73806500
H	1.19110600	2.91612500	-2.50757800
H	-0.47404300	3.34970800	-2.03128300
H	0.20567000	1.76229500	-1.57485500
C	1.38367400	4.80890300	-0.62181500
H	2.14033300	4.85952300	-1.40269300
H	0.52285600	5.41876100	-0.89451400
H	1.80492400	5.15590000	0.32001800
C	-0.03362000	3.22532400	0.65022000
H	-0.93971100	3.78150500	0.41194100
H	0.40799500	3.60636800	1.56937400
H	-0.25768700	2.16682300	0.76012400
H	1.79911600	2.82726500	-0.20134900

9Z_NMe2H2

conf_0

C	-1.44558000	2.47078200	-1.57307000
C	-0.11380600	2.64058400	-2.31913300
C	1.10717600	1.92524000	-1.72675400
C	1.59571400	2.51201000	-0.39236600
C	0.75255700	2.10732100	0.78545400
O	0.31579100	0.98070100	0.93158700
O	0.49319100	3.01513900	1.73987700
H	0.88031000	3.87393500	1.52272800
H	1.67744200	3.60171000	-0.45557500
H	2.59840800	2.12811600	-0.17156200
H	0.91301800	0.85991500	-1.59060800
H	1.93441300	2.00054500	-2.43639700
H	0.11233800	3.70961700	-2.40531000
H	-0.25038100	2.28198200	-3.34458600
C	-1.98785100	1.03638800	-1.55745700
C	-3.46545900	0.96616900	-1.15896900
C	-4.06226500	-0.45630600	-1.21127600
C	-3.76060800	-1.32154500	-0.01606800
C	-2.81182300	-2.26252000	0.09521200
C	-1.77065900	-2.63985900	-0.91641000
C	-0.34115600	-2.49248400	-0.36759800
C	0.73098700	-2.93333900	-1.36836600
C	2.16187900	-2.85637400	-0.82208600

C	2.65375800	-1.43325700	-0.54307600
C	4.09181600	-1.37802900	-0.02456800
C	4.57384400	0.04327200	0.27713800
C	6.01728000	0.09920500	0.77976800
H	6.13493700	-0.47967100	1.70034200
H	6.33239400	1.12480500	0.98811400
H	6.70566600	-0.31597500	0.03851600
H	3.90879600	0.49299300	1.02689500
H	4.48035600	0.65264900	-0.63131400
H	4.76326200	-1.83789300	-0.75926500
H	4.17538500	-1.98606700	0.88473700
H	1.99488900	-0.94093500	0.18038200
H	2.58290500	-0.84714700	-1.46852300
H	2.83931600	-3.33030800	-1.54019800
H	2.23087100	-3.45300900	0.09643300
H	0.51916700	-3.96237400	-1.67807500
H	0.65261600	-2.31945200	-2.27436100
H	-0.17074500	-1.44972100	-0.08086600
H	-0.24161600	-3.09434400	0.54569500
H	-1.87556500	-2.04256600	-1.82425500
H	-1.92752100	-3.68563400	-1.20751800
H	-2.79071000	-2.85097400	1.01375700
H	-4.44342000	-1.19706000	0.82499700
H	-3.72792000	-0.94417300	-2.13026400
H	-5.15029300	-0.37445200	-1.28733300
H	-4.03759300	1.60372800	-1.83992600
H	-3.61259400	1.39593600	-0.15907300
H	-1.88331700	0.61026000	-2.56248100
H	-1.38050300	0.40918700	-0.89871900
H	-2.18173900	3.11609000	-2.06406200
H	-1.37149100	2.85233900	-0.54624600
N	-1.73801500	-0.12964500	2.27986100
C	-1.21286100	-1.15211900	3.22713700
H	-0.65795500	-0.64951100	4.01763400
H	-0.55324900	-1.82316900	2.68066100
H	-2.04472900	-1.71065200	3.65398200
C	-2.65681100	0.86582300	2.89673500
H	-2.98729700	1.55998300	2.12633000
H	-2.12122700	1.40736300	3.67464100
H	-3.51527000	0.34985800	3.32421300
H	-2.22387700	-0.61041700	1.49992100
H	-0.93001500	0.36952000	1.84032700

conf_202

C	1.44576400	-2.47137100	-1.57242700
C	0.11420500	-2.64115400	-2.31884500
C	-1.10680800	-1.92546500	-1.72698300
C	-1.59590400	-2.51180000	-0.39258900
C	-0.75297800	-2.10706800	0.78540300
O	-0.31548600	-0.98070300	0.93118200
O	-0.49477200	-3.01459100	1.74044700
H	-0.88265400	-3.87313500	1.52365200
H	-1.67788700	-3.60149600	-0.45553600

H	-2.59857600	-2.12761400	-0.17222700
H	-0.91248600	-0.86015200	-1.59100500
H	-1.93387900	-2.00070900	-2.43683000
H	-0.11209600	-3.71016000	-2.40489900
H	0.25112800	-2.28278900	-3.34433900
C	1.98823000	-1.03704000	-1.55706400
C	3.46569300	-0.96684800	-1.15806200
C	4.06263100	0.45556300	-1.21071200
C	3.76065900	1.32126400	-0.01593600
C	2.81185300	2.26229000	0.09474300
C	1.77090800	2.63921700	-0.91726300
C	0.34134300	2.49257000	-0.36842600
C	-0.73068900	2.93254100	-1.36970900
C	-2.16162000	2.85590500	-0.82344300
C	-2.65366800	1.43290900	-0.54411200
C	-4.09153200	1.37807000	-0.02502600
C	-4.57361800	-0.04301300	0.27760100
C	-6.01697500	-0.09853400	0.78049200
H	-6.13446700	0.48100500	1.70067400
H	-6.33209700	-1.12397100	0.98963000
H	-6.70545600	0.31614600	0.03905000
H	-3.90847000	-0.49235700	1.02749000
H	-4.48033100	-0.65293500	-0.63051600
H	-4.76321100	1.83763000	-0.75969800
H	-4.17469200	1.98659700	0.88399000
H	-1.99460400	0.94049400	0.17911600
H	-2.58329900	0.84671200	-1.46953400
H	-2.83902100	3.32978200	-1.54161700
H	-2.23052100	3.45274600	0.09495300
H	-0.51888100	3.96132100	-1.68024700
H	-0.65217900	2.31790900	-2.27518700
H	0.17075600	1.45011200	-0.08064300
H	0.24181500	3.09538400	0.54422900
H	1.87573100	2.04125900	-1.82468000
H	1.92806400	3.68474000	-1.20909800
H	2.79053900	2.85110900	1.01305400
H	4.44322100	1.19708300	0.82538800
H	3.72860100	0.94311600	-2.12998600
H	5.15068000	0.37364600	-1.28641200
H	4.03801500	-1.60471400	-1.83857200
H	3.61246100	-1.39623300	-0.15794900
H	1.88412700	-0.61123200	-2.56227100
H	1.38070900	-0.40952100	-0.89878100
H	2.18202200	-3.11690500	-2.06298200
H	1.37131500	-2.85263600	-0.54552000
N	1.73765300	0.13056700	2.28052900
C	1.21221900	1.15359300	3.22705800
H	0.65702000	0.65144300	4.01763800
H	0.55286000	1.82438400	2.67996500
H	2.04399700	1.71228700	3.65386600
C	2.65611700	-0.86469800	2.89822200
H	2.12011200	-1.40602400	3.67598800
H	3.51432800	-0.34858800	3.32602300
H	2.98703600	-1.55907300	2.12819600
H	2.22386000	0.61088800	1.50047700

H	0.92992100	-0.36876100	1.84088400

conf_102

C	-4.61188800	1.18169200	-1.87576400
C	-3.93270100	2.12850700	-0.87630400
C	-2.57104700	2.66042000	-1.33709200
C	-1.84615300	3.44239200	-0.23656200
C	-1.42472900	2.55175100	0.90853000
O	-1.15532100	1.37321500	0.77435200
O	-1.33040200	3.09027700	2.13298500
H	-1.57382300	4.02605900	2.12760300
H	-2.46833000	4.26194000	0.13886500
H	-0.92775500	3.89895300	-0.62590000
H	-1.92382100	1.84069200	-1.65419600
H	-2.70085600	3.31449500	-2.20343900
H	-3.81564000	1.61157700	0.08307500
H	-4.60014500	2.97515200	-0.68215700
C	-3.80811400	-0.07847100	-2.23030800
C	-3.36584000	-0.90792000	-1.02090100
C	-2.62152200	-2.19021500	-1.42893200
C	-2.14629600	-2.99806000	-0.25587800
C	-0.88476300	-3.35726100	0.02630300
C	0.35390700	-3.03493600	-0.76256400
C	1.26830700	-2.02200400	-0.05332100
C	2.56901300	-1.73874500	-0.80592400
C	3.45479100	-0.70983000	-0.10023600
C	4.75828000	-0.41440800	-0.84519100
C	5.64029100	0.61734100	-0.13929400
C	6.94554900	0.91308700	-0.88221300
C	7.81893500	1.94647400	-0.16894800
H	8.74270700	2.13721200	-0.72038900
H	8.09482000	1.60500300	0.83340000
H	7.29325200	2.90032900	-0.06104100
H	6.71203200	1.26465900	-1.89408000
H	7.50762200	-0.01980000	-1.00618200
H	5.87379800	0.26372400	0.87317500
H	5.07726500	1.55150100	-0.01337800
H	4.52421200	-0.06090600	-1.85695100
H	5.32107400	-1.34717600	-0.97147800
H	3.69138500	-1.06730200	0.91057300
H	2.89244300	0.22474100	0.02810000
H	3.12396200	-2.67540700	-0.93232900
H	2.33137900	-1.38475200	-1.81608700
H	0.72321900	-1.07695900	0.07567100
H	1.51117200	-2.39582700	0.94996100
H	0.09885400	-2.65503500	-1.75366600
H	0.91511600	-3.96235200	-0.92161300
H	-0.72462100	-3.98176600	0.90592800
H	-2.93269600	-3.35084400	0.41162400
H	-1.78648300	-1.92532800	-2.08077900
H	-3.29707500	-2.81299600	-2.02789000
H	-4.24280200	-1.16815400	-0.41373100
H	-2.70603400	-0.30247000	-0.39161400

H	-4.41743900	-0.70367500	-2.89102100
H	-2.92181100	0.19371500	-2.81405300
H	-4.83310100	1.72849900	-2.79843900
H	-5.58086600	0.88332000	-1.46130000
N	-1.26272000	-0.93689200	2.21501700
C	-2.63552400	-0.98722600	2.79248400
H	-2.78863900	-1.95286100	3.27183500
H	-3.35519900	-0.85361200	1.98758100
H	-2.73943500	-0.18749100	3.52388300
C	-0.17531800	-1.12909000	3.21572900
H	-0.27393500	-2.11429300	3.66901900
H	-0.26199200	-0.35741600	3.97888200
H	0.78365600	-1.04489700	2.71009200
H	-1.13638100	-0.01753300	1.73807500
H	-1.19530600	-1.65339800	1.46889300

conf_6

C	-1.89018500	2.52231100	-1.11373400
C	-0.71542800	3.05381000	-1.94927000
C	0.66527900	2.44579000	-1.66928900
C	1.28233900	2.85790700	-0.32284500
C	0.65410900	2.17753400	0.86321200
O	0.33541600	1.00358900	0.85830300
O	0.46570500	2.88081600	1.98989900
H	0.74483500	3.80006700	1.88237400
H	1.25766300	3.94571200	-0.20363400
H	2.33719800	2.55862500	-0.29879800
H	0.63177400	1.35657500	-1.72226100
H	1.35559100	2.77107800	-2.45117000
H	-0.65492300	4.14179500	-1.83020200
H	-0.94764700	2.88308300	-3.00550400
C	-2.18993500	1.03218700	-1.31744200
C	-3.56981700	0.62532700	-0.79141800
C	-3.91172200	-0.86214100	-1.02501700
C	-3.34750900	-1.81456900	-0.00448400
C	-2.25776200	-2.58778500	-0.11594700
C	-1.28235800	-2.64595900	-1.25453200
C	0.15070200	-2.31419700	-0.80514100
C	1.18950700	-2.46390000	-1.92077700
C	2.61367000	-2.10816800	-1.47351400
C	2.83122300	-0.60567200	-1.26193200
C	4.21537800	-0.23786000	-0.70997800
C	4.50814100	-0.74956500	0.70808700
C	3.53786400	-0.22576400	1.77076700
H	2.51200800	-0.55091700	1.57818900
H	3.53587700	0.86980100	1.79465300
H	3.82025700	-0.57418200	2.76745500
H	5.52698800	-0.45681100	0.97945500
H	4.50133300	-1.84411500	0.71801000
H	4.32053800	0.85497000	-0.71597000
H	4.98603400	-0.61568200	-1.39067600
H	2.05930600	-0.21129800	-0.59614800
H	2.69007300	-0.09899800	-2.22383100

H	3.32900700	-2.46668000	-2.22094600
H	2.84005100	-2.65698500	-0.55213900
H	1.16732700	-3.49682200	-2.28363900
H	0.90377700	-1.83370400	-2.77206500
H	0.17523400	-1.29227700	-0.41334200
H	0.43055700	-2.97920900	0.02285100
H	-1.57897200	-1.96831200	-2.05760300
H	-1.29337300	-3.65861600	-1.67555600
H	-2.04758200	-3.28167100	0.69969600
H	-3.94440100	-1.91976700	0.90203900
H	-3.59077300	-1.14407400	-2.03086600
H	-4.99941000	-0.97691900	-1.00802500
H	-4.32614100	1.23706600	-1.29253000
H	-3.65700500	0.86426200	0.27695900
H	-2.15318700	0.80837800	-2.39047700
H	-1.40851800	0.42391200	-0.85381000
H	-2.77822000	3.09953500	-1.39260300
H	-1.73859100	2.72826700	-0.04633200
N	-1.25604000	-0.64327500	2.27052100
C	-0.42088000	-1.64280000	2.99314400
H	0.27080400	-2.09292900	2.28424500
H	-1.06481900	-2.40721200	3.42575500
H	0.13559200	-1.13535200	3.77933600
C	-2.25089400	0.06065600	3.12517600
H	-2.92684400	-0.66826800	3.56980900
H	-2.81247900	0.75698000	2.50509800
H	-1.72356400	0.60565200	3.90636800
H	-1.74668500	-1.11049700	1.48565600
H	-0.62571900	0.06037200	1.82051300

conf_10

C	-1.20010100	2.00438400	-2.03004400
C	0.30225900	2.15150400	-2.31759800
C	1.26766400	1.67401100	-1.22446800
C	1.26864100	2.54552300	0.04310300
C	0.06697300	2.32098300	0.92288500
O	-0.36392900	1.21206100	1.17837700
O	-0.54260100	3.38156300	1.47305300
H	-0.12515400	4.20923200	1.19897700
H	1.36961000	3.60380500	-0.21699500
H	2.13515300	2.28561600	0.66234200
H	1.06063600	0.64133700	-0.93992900
H	2.28379700	1.68495700	-1.62525500
H	0.52038900	3.20123300	-2.54526300
H	0.52941200	1.59554100	-3.23306300
C	-1.67714600	0.55412700	-1.89344900
C	-3.20322100	0.41974100	-1.91931200
C	-3.71044000	-1.03926400	-1.91556800
C	-3.71919100	-1.72798200	-0.57339600
C	-2.76418700	-2.51259000	-0.05201600
C	-1.41791800	-2.81077700	-0.64161700
C	-0.27367100	-2.29791000	0.25225700
C	1.09378200	-2.32215600	-0.43178800

C	2.19330300	-1.69442200	0.42842900
C	3.51945100	-1.51244700	-0.31176100
C	4.57769700	-0.78091000	0.51579300
C	5.90348700	-0.58245600	-0.22258600
C	6.94683800	0.16453000	0.60968100
H	6.59013400	1.16103500	0.88935400
H	7.88213000	0.28970100	0.05907200
H	7.17478000	-0.37605400	1.53310400
H	5.71834200	-0.03772300	-1.15635700
H	6.29997700	-1.56098400	-0.51615300
H	4.76001000	-1.33292100	1.44612400
H	4.18437800	0.19957500	0.81968200
H	3.34004700	-0.95563700	-1.24054600
H	3.90644900	-2.49151400	-0.61744900
H	2.35399000	-2.30623100	1.32421900
H	1.84594700	-0.71793800	0.78955000
H	1.36424400	-3.34999800	-0.69783100
H	1.02079100	-1.77337400	-1.37932400
H	-0.47890600	-1.26315700	0.54521800
H	-0.23770100	-2.88514600	1.17811900
H	-1.32869300	-2.36194900	-1.63235800
H	-1.31119200	-3.89329500	-0.77472500
H	-2.97092400	-2.99511300	0.90396400
H	-4.64381200	-1.62135600	-0.00550000
H	-3.11803200	-1.61505100	-2.63158800
H	-4.73646800	-1.04886400	-2.29317400
H	-3.57805200	0.90662200	-2.82498200
H	-3.64890500	0.97311700	-1.08190000
H	-1.26608000	-0.02838800	-2.72657100
H	-1.26844200	0.11034300	-0.98256300
H	-1.74202200	2.47441100	-2.85752500
H	-1.48767400	2.57668100	-1.13868000
N	-2.68436700	0.06150600	1.96242700
C	-2.39814200	-0.76126400	3.17142500
H	-3.27075200	-1.36786200	3.40936200
H	-2.16747600	-0.09809000	4.00344000
H	-1.54376500	-1.40088900	2.95961400
C	-3.83677800	0.99220300	2.11205200
H	-4.73698000	0.42003600	2.33140200
H	-3.96470500	1.54294500	1.18209200
H	-3.62824800	1.68577600	2.92490100
H	-2.85353300	-0.57344000	1.15996500
H	-1.82568200	0.60451700	1.71668100

conf_1

C	-1.49880700	2.85016400	-1.18786200
C	-0.11868200	3.42313700	-1.53651600
C	1.10160000	2.75543900	-0.89054000
C	1.12768700	2.81276200	0.65906900
C	0.33472600	1.71081400	1.29749900
O	-0.70694100	1.85257900	1.90940000
O	0.80299900	0.45365700	1.15387800
H	1.63943400	0.42410800	0.66410000

H	0.73305000	3.76306800	1.01784300
H	2.16128700	2.72357300	1.00832700
H	1.19473300	1.71855300	-1.23103900
H	1.99824800	3.26556600	-1.24877100
H	-0.10665200	4.48645700	-1.27461400
H	0.01798400	3.37836300	-2.62234800
C	-1.70476000	1.37444200	-1.55267000
C	-3.18621300	0.98288200	-1.55278500
C	-3.44943500	-0.48729400	-1.93749100
C	-3.28802200	-1.46990900	-0.81015400
C	-2.27979100	-2.32979100	-0.60992500
C	-1.03841600	-2.49208100	-1.43791300
C	0.23031500	-2.62376500	-0.58242100
C	1.49531800	-2.80905800	-1.42466000
C	2.75360100	-3.12650200	-0.60589000
C	3.20981600	-2.03480800	0.37187300
C	3.62349600	-0.72090300	-0.29981000
C	4.27107100	0.28531300	0.65946500
C	4.75268400	1.56008800	-0.03559700
H	3.93821200	2.06695800	-0.56199700
H	5.52012100	1.32971200	-0.77892100
H	5.18445800	2.26660300	0.67703300
H	5.11737500	-0.20014000	1.15550800
H	3.57695400	0.53846900	1.47411300
H	2.76304900	-0.25841900	-0.81014500
H	4.33096500	-0.93944100	-1.10791700
H	4.06759200	-2.41556600	0.93580600
H	2.42950800	-1.84551600	1.11978000
H	3.57638100	-3.33988700	-1.29660400
H	2.58258500	-4.05203700	-0.04488000
H	1.32848400	-3.62951000	-2.13072700
H	1.65691700	-1.91620100	-2.03993100
H	0.32651900	-1.73676700	0.05165300
H	0.11987800	-3.48023100	0.09488300
H	-0.92806700	-1.66051900	-2.13774300
H	-1.14202700	-3.39857000	-2.04810600
H	-2.37273200	-3.03523800	0.21710600
H	-4.13387200	-1.51817900	-0.12374500
H	-2.80923700	-0.75708400	-2.78072400
H	-4.47952400	-0.57480000	-2.29656000
H	-3.70982200	1.62817300	-2.26434600
H	-3.63263400	1.19929200	-0.57306900
H	-1.29768600	1.18775700	-2.55405800
H	-1.14274000	0.71977900	-0.87518500
H	-2.24079700	3.44430700	-1.73136800
H	-1.71824400	3.00308400	-0.12689900
N	-2.14756200	-0.46675300	2.10056800
C	-1.38989600	-1.47169200	2.89822400
H	-0.41664100	-1.61409800	2.43445300
H	-1.94271000	-2.40963300	2.91424600
H	-1.26634700	-1.09477800	3.91217000
C	-3.50501900	-0.15831600	2.63081400
H	-3.97667000	0.57470900	1.97892100
H	-3.40681400	0.25147500	3.63460300
H	-4.09882400	-1.07079000	2.65756800

H	-2.23600900	-0.81063200	1.12796800
H	-1.58886100	0.41990700	2.05715400

conf_22

C	-1.66053500	2.26221700	-1.88830200
C	-0.27515400	2.59614500	-2.45777800
C	0.94272700	2.13081400	-1.64936000
C	1.05069900	2.74415600	-0.22710600
C	0.25949400	1.98166600	0.79509500
O	-0.77184100	2.35738600	1.31983000
O	0.71510600	0.75898700	1.13421900
H	1.52447600	0.51154500	0.65863900
H	0.70166400	3.77656700	-0.22471400
H	2.09767500	2.74111300	0.09264300
H	0.96393600	1.03789100	-1.58990100
H	1.84529800	2.41589200	-2.19447100
H	-0.20519100	3.68084100	-2.59123800
H	-0.19081400	2.16293300	-3.46031900
C	-1.95176400	0.76576800	-1.72239200
C	-3.43259200	0.49458800	-1.43611900
C	-3.80496100	-0.99982800	-1.35210700
C	-3.41358500	-1.68048700	-0.06651200
C	-2.34039200	-2.45274500	0.15409400
C	-1.25635400	-2.79307300	-0.82508600
C	0.15326500	-2.52678100	-0.27401600
C	1.24049700	-2.74605900	-1.33043200
C	2.67750000	-2.59360300	-0.81681200
C	2.97597400	-1.22578100	-0.19407300
C	4.45677500	-0.94533200	0.06134100
C	4.70045300	0.43727400	0.67308200
C	6.17808600	0.73320700	0.93385100
H	6.31741100	1.72603100	1.36783000
H	6.75581900	0.68968700	0.00652400
H	6.60653200	0.00332200	1.62614500
H	4.14207300	0.51905800	1.61540800
H	4.29123900	1.20261500	-0.00118900
H	5.00907100	-1.02910900	-0.88135600
H	4.86431300	-1.71591700	0.72558200
H	2.44210300	-1.15391600	0.76317600
H	2.58199000	-0.44494800	-0.86358700
H	3.36588700	-2.76002600	-1.65157200
H	2.89402500	-3.37680400	-0.08147500
H	1.12203400	-3.74600600	-1.76092400
H	1.07798900	-2.04045200	-2.15543900
H	0.19405200	-1.49862800	0.09791400
H	0.33885300	-3.17695900	0.59009800
H	-1.39174600	-2.23766800	-1.75516600
H	-1.33790100	-3.85643500	-1.08305900
H	-2.24413300	-2.92490700	1.13226700
H	-4.13421100	-1.57782100	0.74562500
H	-3.37011100	-1.51992100	-2.20903300
H	-4.88969900	-1.09319200	-1.45808300
H	-4.02575300	0.95000400	-2.23492800

H	-3.73606800	1.01015400	-0.51498600
H	-1.67185900	0.23973000	-2.64329600
H	-1.32890900	0.33174400	-0.93066900
H	-2.40342400	2.68191200	-2.57445700
H	-1.81401500	2.77673200	-0.93510200
N	-2.22214200	0.22695800	2.26353200
C	-3.54993300	0.71002600	2.73651900
H	-4.03936400	1.24189500	1.92285400
H	-3.39918000	1.38378000	3.57821400
H	-4.15581700	-0.14069000	3.04461700
C	-1.44835200	-0.53425600	3.28481700
H	-1.27256900	0.11002100	4.14469900
H	-0.49907200	-0.82952600	2.84457900
H	-2.01900800	-1.41113300	3.58671800
H	-1.65473000	1.05080300	1.94755400
H	-2.35508500	-0.37740100	1.43454000

conf_124

C	3.17395100	1.79676000	2.75439100
C	3.61660600	2.19619200	1.33984900
C	2.56062700	2.97805700	0.54963500
C	2.93267000	3.13097100	-0.92891300
C	2.89053500	1.80794800	-1.65634200
O	2.13718400	0.90272400	-1.35239600
O	3.71704200	1.62462000	-2.69737700
H	4.27553400	2.39992600	-2.84562800
H	3.92086900	3.58938300	-1.04241700
H	2.22007000	3.78813800	-1.44240300
H	1.59106400	2.48017900	0.60385100
H	2.43102900	3.97252700	0.98445200
H	3.89570500	1.29351400	0.78389900
H	4.52878200	2.79839200	1.41020900
C	1.94154100	0.88222000	2.81728100
C	2.08314500	-0.42762400	2.03554900
C	0.83231700	-1.31382500	2.14901600
C	0.96368400	-2.63463700	1.44768900
C	0.14743000	-3.12942100	0.50382800
C	-1.08727900	-2.46404700	-0.04372100
C	-2.35340100	-2.86652500	0.73943500
C	-3.64306300	-2.26148300	0.17479900
C	-3.71067700	-0.73327200	0.25207100
C	-5.05543600	-0.16577500	-0.20803400
C	-5.12251700	1.36141800	-0.14704800
C	-6.46853500	1.93192900	-0.60073000
C	-6.52294400	3.45926000	-0.53845000
H	-6.35309100	3.81836400	0.48122100
H	-7.49393900	3.83835800	-0.86620000
H	-5.75750100	3.90769600	-1.17966200
H	-7.26556100	1.51063100	0.02269600
H	-6.67434300	1.59816500	-1.62458500
H	-4.32326400	1.78540600	-0.76953100
H	-4.91761000	1.69292500	0.87902700
H	-5.85593200	-0.58944500	0.41023900

H	-5.25653800	-0.49741700	-1.23438900
H	-2.91474800	-0.28795800	-0.35751300
H	-3.51889400	-0.41658000	1.28562100
H	-3.77197900	-2.58174000	-0.86681300
H	-4.49072700	-2.68152400	0.72652600
H	-2.43138400	-3.95857100	0.74463700
H	-2.22766600	-2.56223500	1.78438700
H	-1.22720600	-2.75130600	-1.09278100
H	-0.97993000	-1.37653100	-0.02449300
H	0.33773100	-4.14366600	0.15501500
H	1.78766100	-3.26187600	1.78586800
H	-0.03915600	-0.76418500	1.78501100
H	0.64288000	-1.51644700	3.21064600
H	2.95996600	-0.97938200	2.39726500
H	2.26200600	-0.20118300	0.97960300
H	1.73723700	0.64870200	3.86732300
H	1.05673900	1.41739400	2.45539100
H	2.97059600	2.70160100	3.33693500
H	4.01375200	1.29929800	3.25133300
N	2.14512600	-1.81792800	-1.55678400
C	3.55919200	-2.24368600	-1.36111700
H	3.60791200	-3.33132800	-1.34108300
H	3.91429800	-1.84002700	-0.41447500
H	4.16229800	-1.85643600	-2.18064500
C	1.51718600	-2.33213800	-2.80595100
H	2.09464200	-1.98485000	-3.66119500
H	0.49950900	-1.95163800	-2.86677500
H	1.50594700	-3.42072800	-2.78042400
H	2.11101200	-0.77505900	-1.55675300
H	1.59234300	-2.13767100	-0.73714000

conf_51

C	-3.48883800	0.53034600	-1.96505100
C	-4.98234300	0.73517500	-1.67723000
C	-5.39770400	0.71744600	-0.19908400
C	-4.79489300	1.86116700	0.63354300
C	-3.36366200	1.62964700	1.04301600
O	-2.92734300	0.54789200	1.38643400
O	-2.52667600	2.68454900	1.07036700
H	-2.96714100	3.49127100	0.77097700
H	-5.34913100	1.96280400	1.57454600
H	-4.89367200	2.81593600	0.10686000
H	-6.48483900	0.80706400	-0.14230000
H	-5.14086800	-0.23309800	0.27365600
H	-5.55071200	-0.04173400	-2.19867500
H	-5.30169100	1.68654800	-2.11771100
C	-2.95485300	-0.85613000	-1.59456300
C	-1.45920700	-1.00571600	-1.88540100
C	-0.88711500	-2.40676800	-1.59618000
C	-0.91391100	-2.79422800	-0.14443300
C	0.12784400	-3.20918200	0.59135300
C	1.55472900	-3.35241500	0.13168000
C	2.37931800	-2.07678500	0.37400100

C	3.84984000	-2.20859600	-0.02832100
C	4.68116600	-0.94999200	0.24847800
C	4.28171800	0.27174700	-0.58478700
C	5.15057800	1.50131400	-0.31287200
C	4.73336600	2.73274500	-1.12056000
C	5.61088800	3.95528600	-0.84685600
H	5.57518000	4.23904700	0.20942800
H	5.29003600	4.81828500	-1.43531300
H	6.65570000	3.75208700	-1.09831000
H	3.68639900	2.97477700	-0.89466400
H	4.76483600	2.48981800	-2.18904400
H	6.19687500	1.26020800	-0.53456700
H	5.11745800	1.74165500	0.75789500
H	3.23298200	0.53432200	-0.39598500
H	4.33760600	0.01455500	-1.64985700
H	5.73522900	-1.17518100	0.05667200
H	4.61827100	-0.70119700	1.31633900
H	4.28689500	-3.05609300	0.51026400
H	3.90867600	-2.45884200	-1.09422700
H	1.91618800	-1.25735700	-0.18715800
H	2.32793500	-1.80750900	1.43877300
H	1.59604500	-3.60483300	-0.93056900
H	2.01920300	-4.18638600	0.66598600
H	-0.06534400	-3.48519400	1.62731600
H	-1.89017700	-2.76784500	0.33585500
H	0.13088800	-2.46474500	-1.98562300
H	-1.47701500	-3.13999000	-2.16068700
H	-0.89710600	-0.25542300	-1.31195800
H	-1.27433800	-0.77104400	-2.93868000
H	-3.15201500	-1.05679200	-0.53892500
H	-3.50663100	-1.61391100	-2.16318100
H	-2.89191700	1.30050100	-1.45761800
H	-3.31970800	0.70070300	-3.03346100
N	-0.29621300	-0.12977700	1.71349100
C	0.58178000	0.97283400	1.23034800
H	0.33565000	1.87972700	1.77946700
H	0.39747000	1.12753200	0.16895100
H	1.62297400	0.70151200	1.38894100
C	-0.09046700	-0.48658100	3.14528300
H	-0.29559200	0.38878100	3.75923800
H	0.93814100	-0.81217800	3.28997000
H	-0.77647200	-1.29008100	3.40711100
H	-0.16450400	-0.97911300	1.13611700
H	-1.29554100	0.15244800	1.58111900

conf_127

C	0.81186700	2.50301900	-1.52416400
C	-0.65641300	2.09237800	-1.69921200
C	-1.69078200	3.12639200	-1.23911500
C	-1.56076300	3.52629600	0.25522500
C	-1.34901500	2.31577300	1.12456100
O	-0.27298300	2.02029700	1.61274800
O	-2.38404100	1.48859300	1.32409300

H	-3.18174800	1.79366000	0.86989700
H	-2.45646200	4.06621400	0.57623800
H	-0.70506200	4.18112900	0.40801900
H	-1.61116900	4.04386300	-1.82789200
H	-2.69454200	2.73063200	-1.42622500
H	-0.82926400	1.15656500	-1.15427000
H	-0.84686800	1.85378200	-2.74984200
C	1.74332800	1.29357300	-1.61262200
C	3.21957900	1.60561400	-1.37312900
C	4.08212300	0.33155800	-1.27929600
C	3.87527800	-0.43692000	-0.00388800
C	3.39309800	-1.67826500	0.14909200
C	2.93199900	-2.63464700	-0.91496800
C	1.52526400	-3.20693500	-0.66268500
C	0.40887300	-2.17876500	-0.84246700
C	-0.99541000	-2.71270300	-0.55777000
C	-2.06200400	-1.61549200	-0.59194900
C	-3.48857500	-2.12911800	-0.39180500
C	-4.53507200	-1.01184100	-0.34962800
C	-5.96813000	-1.53312500	-0.23142500
H	-6.69211800	-0.71542100	-0.19540900
H	-6.09525300	-2.13118900	0.67549900
H	-6.22518700	-2.16703600	-1.08450600
H	-4.44097700	-0.39692700	-1.25368200
H	-4.31574400	-0.35565000	0.50267800
H	-3.54397700	-2.70897600	0.53783100
H	-3.73802100	-2.82671500	-1.19987800
H	-2.00156500	-1.08599100	-1.55108900
H	-1.83861100	-0.86778700	0.18061400
H	-1.01379100	-3.20983300	0.42152900
H	-1.24241200	-3.49157300	-1.28752800
H	0.59852200	-1.31307600	-0.19615800
H	0.44561500	-1.78024900	-1.86264300
H	1.35630100	-4.04297000	-1.34790000
H	1.48029100	-3.63325300	0.34793600
H	3.64546200	-3.46650500	-0.94637900
H	2.96256300	-2.16449700	-1.90026000
H	3.39290600	-2.09725500	1.15775000
H	4.22250400	0.08137400	0.89068400
H	5.13906400	0.61599900	-1.32512900
H	3.89442100	-0.29864400	-2.15135500
H	3.33139100	2.19136800	-0.45145900
H	3.60064200	2.23632900	-2.18169400
H	1.41104900	0.55343000	-0.87703800
H	1.63050900	0.80619700	-2.58844300
H	0.95997000	2.97711900	-0.54952100
H	1.08316100	3.25114900	-2.27673500
N	1.31095900	-0.10094100	2.19739200
C	2.27007100	0.51290500	3.16071700
H	3.02485800	-0.22256100	3.43469600
H	1.72124000	0.83399100	4.04407400
H	2.73673000	1.37328900	2.68564800
C	0.58429300	-1.28618400	2.73639900
H	-0.10489200	-1.64978100	1.97803400
H	0.03199100	-0.98190000	3.62367100

H	1.30327500	-2.06301900	2.99072200
H	1.82974300	-0.37226500	1.34607700
H	0.61727900	0.63137200	1.92573900

conf_37

C	0.11861200	1.93690800	1.84141300
C	-1.16322700	2.46939700	1.18433300
C	-1.26580200	2.35631000	-0.34192000
C	-0.23839500	3.21165600	-1.10211500
C	1.12832200	2.58287300	-1.15699000
O	1.30209700	1.39348400	-1.34517600
O	2.21037400	3.36742700	-1.02138000
H	1.96056500	4.28901900	-0.87025400
H	-0.18584200	4.22151000	-0.68305800
H	-0.55017400	3.31857700	-2.14783200
H	-1.16779600	1.31987500	-0.67056900
H	-2.26198500	2.68259300	-0.64713400
H	-1.28796500	3.52239600	1.46192000
H	-2.01877200	1.94408200	1.62026100
C	0.30955800	0.41704300	1.76435000
C	1.41109600	-0.07610900	2.70964000
C	1.57210900	-1.61046300	2.75855200
C	2.37162400	-2.21331600	1.63314900
C	1.90324000	-2.81296500	0.52884200
C	0.46944600	-2.95536000	0.11498000
C	0.18403200	-2.29100900	-1.24404000
C	-1.27453600	-2.41604900	-1.69981500
C	-2.27644900	-1.74267900	-0.75680000
C	-3.69086100	-1.66559200	-1.33782900
C	-4.73299500	-1.08924300	-0.37209600
C	-4.46696600	0.35795800	0.05427300
C	-5.57370000	0.93264500	0.93980200
H	-5.35901900	1.96318000	1.23578700
H	-5.69275200	0.34185200	1.85291900
H	-6.53525500	0.93042700	0.41891200
H	-4.35287200	0.97885700	-0.84344600
H	-3.51318500	0.41359900	0.58969200
H	-4.79009400	-1.72309500	0.52192200
H	-5.72030600	-1.14225700	-0.84380000
H	-4.00443700	-2.67020800	-1.64291400
H	-3.67097100	-1.06184300	-2.25407700
H	-1.91712000	-0.73461400	-0.52275800
H	-2.31624100	-2.28069900	0.19731000
H	-1.36616700	-1.96813800	-2.69557200
H	-1.53332500	-3.47476000	-1.81683700
H	0.44204700	-1.22610800	-1.18818000
H	0.83797700	-2.73796300	-2.00302300
H	-0.18728000	-2.53483900	0.87605300
H	0.22472100	-4.02201400	0.03954700
H	2.62830200	-3.26910600	-0.14685100
H	3.45167000	-2.20987500	1.78367800
H	0.58033300	-2.06629400	2.80970800
H	2.07730000	-1.87487100	3.69206000

H	1.17755400	0.27338000	3.72008500
H	2.37085000	0.39128000	2.45144800
H	-0.63149800	-0.07124300	2.04326700
H	0.51470600	0.10972600	0.73502300
H	0.08876700	2.22607600	2.89732600
H	1.00478800	2.44273300	1.43523100
N	3.37764200	-0.24749900	-0.78257300
C	3.82259700	-0.95129400	-2.01753300
H	4.56263400	-1.70639800	-1.75616200
H	4.25869400	-0.22460300	-2.70101600
H	2.95653900	-1.42109800	-2.47935700
C	4.46784100	0.44778500	-0.04482100
H	5.22609800	-0.27705700	0.24764300
H	4.04450000	0.91946800	0.83991600
H	4.90511800	1.20630800	-0.69173400
H	2.91721400	-0.93014100	-0.15194300
H	2.63614100	0.44651600	-1.03497300

conf_52

C	-3.91938400	-2.33437200	0.80057200
C	-5.11140900	-2.80348900	-0.04375800
C	-5.69165400	-1.77667500	-1.02698000
C	-6.27752100	-0.52654100	-0.34926200
C	-5.24153400	0.49230200	0.05084700
O	-4.26403100	0.75493800	-0.62363500
O	-5.42440100	1.18010700	1.19178800
H	-6.22152400	0.88957400	1.65511500
H	-6.89434100	-0.80576500	0.51112600
H	-6.93732600	-0.00227800	-1.05125800
H	-4.94408100	-1.46008000	-1.75775600
H	-6.49507400	-2.25321500	-1.59309400
H	-5.91257700	-3.13262600	0.62764000
H	-4.81466700	-3.69050100	-0.61282000
C	-2.63480000	-2.07420200	0.00796100
C	-1.50552900	-1.54824300	0.89806500
C	-0.13892200	-1.40515200	0.20123200
C	-0.12508400	-0.46940500	-0.97427100
C	0.59132800	0.65870400	-1.09847700
C	1.54152100	1.24885500	-0.09314500
C	3.01265000	0.95786800	-0.44364300
C	3.98926800	1.60238800	0.54336100
C	5.46628400	1.38089000	0.19560400
C	5.91987600	-0.08082900	0.25598300
C	7.42028000	-0.25310900	0.00976400
C	7.88291400	-1.71125000	0.06107200
C	9.38443500	-1.87134200	-0.18306700
H	9.66727900	-1.48203400	-1.16577400
H	9.68691800	-2.92065400	-0.14130400
H	9.96551300	-1.32731000	0.56761400
H	7.32605700	-2.29222500	-0.68404700
H	7.62233000	-2.13733700	1.03734700
H	7.97849500	0.33001500	0.75277200
H	7.67841600	0.17392000	-0.96756100

H	5.36995100	-0.67689900	-0.48147300
H	5.66419600	-0.49706700	1.23931200
H	6.07878000	1.96953100	0.88733300
H	5.66725900	1.78369100	-0.80493600
H	3.79001500	2.68009400	0.58560800
H	3.79326700	1.21353600	1.55091800
H	3.15242200	-0.12639200	-0.46859500
H	3.22182000	1.31936200	-1.45723900
H	1.33375200	0.87518100	0.91375500
H	1.40667000	2.33836300	-0.05835200
H	0.54653700	1.17652600	-2.05632300
H	-0.72857800	-0.77370000	-1.82667900
H	0.16541000	-2.39519300	-0.15982400
H	0.61019800	-1.10716200	0.93731800
H	-1.38022800	-2.22010500	1.75352300
H	-1.80310700	-0.58027000	1.32352200
H	-2.31490300	-3.00834100	-0.46873500
H	-2.83371200	-1.36535700	-0.79999600
H	-3.71503000	-3.09697500	1.55929500
H	-4.18575300	-1.43367100	1.37010400
N	-1.98445000	2.14864400	-0.08892900
C	-1.91694300	3.29496000	-1.03779800
H	-2.70426300	4.00576900	-0.79225500
H	-2.06164600	2.91935300	-2.04915800
H	-0.94265900	3.77399700	-0.95413900
C	-1.80775500	2.52793500	1.34001800
H	-0.83934900	3.00894800	1.46654100
H	-1.85657800	1.62790600	1.94992200
H	-2.60696900	3.20911400	1.62729300
H	-1.26099700	1.45358900	-0.35753900
H	-2.90144100	1.66007200	-0.21439500

conf_78

C	-4.36423400	-1.85044200	1.40154400
C	-4.81228900	-3.01780500	0.51268000
C	-4.50086000	-2.86776500	-0.98466000
C	-5.18379300	-1.66252300	-1.65171700
C	-4.50060700	-0.33981600	-1.40998400
O	-3.29589000	-0.19047100	-1.35338700
O	-5.27592200	0.75895000	-1.29756900
H	-6.21478700	0.53324200	-1.34174500
H	-6.23673900	-1.60457300	-1.35653400
H	-5.17243500	-1.79186300	-2.74110300
H	-3.42528500	-2.80127800	-1.16177000
H	-4.84208500	-3.76706100	-1.50230200
H	-5.89143000	-3.16515800	0.63290600
H	-4.34135800	-3.94041900	0.86708800
C	-2.84441200	-1.66791700	1.47410300
C	-2.43147200	-0.29130200	1.99414400
C	-0.91212900	-0.06501800	1.94074200
C	-0.52724300	1.37531700	2.12914400
C	0.40337300	2.05731200	1.44353300
C	1.31853200	1.51513300	0.37930000

C	2.69046000	1.10875500	0.95228300
C	3.65433700	0.60291200	-0.12203600
C	5.01490500	0.18454700	0.43972300
C	5.98137400	-0.32446400	-0.63199000
C	7.34159000	-0.74476100	-0.07153100
C	8.30992800	-1.25484300	-1.14167100
C	9.66602700	-1.67297800	-0.57073000
H	10.33655600	-2.03231300	-1.35528900
H	9.55338000	-2.47568500	0.16437800
H	10.15857800	-0.83353800	-0.07068200
H	8.45490100	-0.47384600	-1.89753500
H	7.85395900	-2.10418200	-1.66432100
H	7.19637100	-1.52537600	0.68596500
H	7.79672900	0.10555500	0.45178900
H	6.12725200	0.45653300	-1.38885400
H	5.52688200	-1.17526900	-1.15534200
H	4.86939500	-0.59563500	1.19702600
H	5.46795800	1.03591900	0.96218500
H	3.20100800	-0.24906100	-0.64489900
H	3.79915500	1.38508000	-0.87792700
H	3.13168600	1.96683500	1.47118600
H	2.53830700	0.33497600	1.71228900
H	1.48012000	2.27810100	-0.39124300
H	0.87885700	0.64421500	-0.11856000
H	0.58617600	3.08909400	1.73793500
H	-1.05171100	1.89896800	2.92689500
H	-0.51780400	-0.44897500	0.99492800
H	-0.43580000	-0.66607100	2.72488700
H	-2.79279000	-0.13985500	3.01711700
H	-2.93825700	0.46857700	1.38559100
H	-2.41121800	-2.45186500	2.10473900
H	-2.40891500	-1.79647100	0.47969300
H	-4.76324900	-1.99979500	2.40955800
H	-4.82634400	-0.91764100	1.05073900
N	-2.05183000	2.18762300	-0.66269500
C	-1.30136200	2.62812200	-1.87309900
H	-0.59178800	1.85040700	-2.14726500
H	-0.77247100	3.55322400	-1.65000700
H	-2.00823800	2.78896900	-2.68541500
C	-3.08312000	3.16079600	-0.20169800
H	-3.83574000	3.27151500	-0.97941100
H	-2.60405700	4.11642600	0.00507600
H	-3.54611600	2.77371200	0.70389700
H	-1.38759000	2.02755700	0.11734800
H	-2.51779200	1.27626400	-0.87496600

conf_100

C	4.76488900	0.52166000	0.66886900
C	5.71375700	-0.00251800	-0.41604500
C	5.05860900	-0.86886000	-1.50323900
C	4.37536800	-2.13751500	-0.96709900
C	3.00197500	-1.91028600	-0.38578000
O	2.20062500	-1.10542900	-0.81644000

O	2.61746900	-2.68810600	0.64838000
H	3.32963800	-3.27869200	0.92850400
H	5.01648000	-2.64313600	-0.23727700
H	4.22092800	-2.84714500	-1.78951700
H	4.32766300	-0.29695200	-2.07884500
H	5.82984500	-1.18183900	-2.21058100
H	6.51271900	-0.58266500	0.05914400
H	6.20621000	0.84390300	-0.90559200
C	3.70896800	1.51026700	0.16061200
C	2.52742000	1.65675000	1.11819900
C	1.40226300	2.53820300	0.56522600
C	0.14573300	2.47065400	1.39466500
C	-1.09009400	2.80274800	1.00027600
C	-1.49347800	3.35095800	-0.34255500
C	-2.94518300	3.01976600	-0.71328200
C	-3.19491300	1.51784400	-0.84107700
C	-4.64500200	1.14622900	-1.16159200
C	-4.89187800	-0.36675600	-1.22609300
C	-4.81827200	-1.07463100	0.13117700
C	-4.92319900	-2.59835300	0.03429100
C	-4.81688800	-3.29445800	1.39177400
H	-4.89849900	-4.37939300	1.29419300
H	-5.60850900	-2.96132000	2.06858300
H	-3.85837000	-3.07620100	1.87640300
H	-4.13986600	-2.97544100	-0.63643200
H	-5.87307300	-2.86305500	-0.44211400
H	-5.61790700	-0.69463400	0.77761900
H	-3.88396500	-0.81553600	0.64863700
H	-4.17172100	-0.82087700	-1.92060100
H	-5.87820900	-0.55387400	-1.66189500
H	-4.92132900	1.60048900	-2.11863900
H	-5.30697600	1.59126500	-0.40942500
H	-2.54170100	1.11526600	-1.62754400
H	-2.89761200	1.03514300	0.09849900
H	-3.61391400	3.43543600	0.04921800
H	-3.20303200	3.51735400	-1.65283300
H	-0.81369100	2.99838800	-1.12721300
H	-1.37019600	4.44095300	-0.31714000
H	-1.88711700	2.74483300	1.73991900
H	0.28154900	2.17651900	2.43434800
H	1.19172000	2.25868600	-0.47482100
H	1.75065800	3.57730100	0.51498100
H	2.86308500	2.05509400	2.08222000
H	2.13381700	0.65472500	1.33435300
H	4.17450200	2.48652600	-0.01248400
H	3.32330000	1.17729800	-0.80639300
H	5.35664200	0.99370900	1.45912400
H	4.26383000	-0.32335000	1.16065400
N	-0.29503500	-0.59176400	0.25179900
C	-0.41999400	-1.17259800	1.62051500
H	-0.01549600	-2.18250400	1.60585500
H	-1.47052700	-1.18516900	1.90564900
H	0.14762300	-0.55456700	2.31274500
C	-1.08883500	-1.32082500	-0.78090800
H	-2.13923700	-1.30951800	-0.50538400

H	-0.72076000	-2.34405500	-0.82978000
H	-0.94630400	-0.83188500	-1.74183000
H	0.70247900	-0.64361600	-0.04928000
H	-0.56165000	0.40349500	0.29925900

conf_68

C	-0.13546200	-3.38092800	1.43882900
C	1.31507800	-3.83259800	1.22268100
C	2.14693200	-2.97439500	0.25563800
C	1.53928400	-2.81009700	-1.15921400
C	0.47591100	-1.75009300	-1.28626400
O	-0.66978500	-1.95925900	-1.64291700
O	0.81845800	-0.48161900	-1.00495800
H	1.74159900	-0.40033000	-0.71971800
H	1.10690300	-3.74820300	-1.50703600
H	2.33661000	-2.53572700	-1.85938900
H	2.35243900	-1.99285600	0.69395400
H	3.12494800	-3.44473300	0.13385200
H	1.30843300	-4.86273700	0.85171100
H	1.84103100	-3.85922300	2.18283800
C	-0.27602300	-1.96116300	2.00084200
C	-1.66725900	-1.35820800	1.78860900
C	-1.69263800	0.14960100	2.08712000
C	-2.94443500	0.84965900	1.64099500
C	-2.99340900	1.89296600	0.79753900
C	-1.79067300	2.53357800	0.14631600
C	-1.01029600	3.48063500	1.08191200
C	0.32781000	3.94487900	0.49126500
C	1.28785500	2.78927800	0.18946700
C	2.69279500	3.23139300	-0.23050100
C	3.53320000	2.09302200	-0.82350200
C	3.81575100	0.93222600	0.13700600
C	4.61521900	-0.19928000	-0.51416900
H	5.58115900	0.16472800	-0.87237600
H	4.09371700	-0.61370900	-1.38580500
H	4.80739500	-1.01652900	0.18485200
H	2.87391000	0.54108400	0.54983300
H	4.36037100	1.30896400	1.00937300
H	4.49093700	2.49247400	-1.17287400
H	3.02591700	1.70862100	-1.71951500
H	3.21434600	3.67180800	0.62700400
H	2.60876200	4.02755500	-0.97819000
H	0.87372400	2.16838700	-0.61234700
H	1.34995900	2.14533500	1.07525800
H	0.15277100	4.52226700	-0.42462900
H	0.80017400	4.63371900	1.19937800
H	-1.63677900	4.34491800	1.32066200
H	-0.82504500	2.96535700	2.02983400
H	-2.10361800	3.09547200	-0.73971900
H	-1.10474300	1.75556000	-0.20207700
H	-3.96132500	2.35141700	0.60820400
H	-3.87615200	0.49335000	2.07623600
H	-0.81910600	0.61216500	1.62285900

H	-1.57737100	0.29884700	3.16753400
H	-2.41408300	-1.88155800	2.39480100
H	-1.94985600	-1.52873200	0.74391800
H	-0.01954500	-1.95194800	3.06550500
H	0.44679500	-1.29584500	1.51858500
H	-0.62670100	-4.09117500	2.11058100
H	-0.67767600	-3.45064400	0.49017600
N	-2.86709500	-0.31917100	-1.53586200
C	-2.85985900	0.61620600	-2.69612800
H	-3.75847100	1.23052800	-2.66848300
H	-2.83370500	0.03442000	-3.61595000
H	-1.97553500	1.24620800	-2.62995400
C	-4.00622700	-1.28134700	-1.53745600
H	-4.94369800	-0.72740700	-1.52869400
H	-3.93474300	-1.90666900	-0.64960400
H	-3.94304300	-1.90036100	-2.43046500
H	-2.90051600	0.22651300	-0.65638600
H	-1.97123800	-0.86211300	-1.53561900

conf_163

C	-4.95416700	2.49585000	-0.84506500
C	-5.52956900	1.07901800	-0.71735500
C	-5.15660700	0.14843400	-1.87844300
C	-5.42056300	-1.32385800	-1.55387500
C	-4.43385700	-1.87568900	-0.55031300
O	-3.31734900	-1.42042000	-0.39058400
O	-4.79760100	-2.94241800	0.17353100
H	-5.70755500	-3.20665800	-0.01925900
H	-5.32014300	-1.94697500	-2.45174900
H	-6.44266300	-1.47062600	-1.18764000
H	-5.72686500	0.42169500	-2.76988500
H	-4.10094200	0.25516900	-2.13502000
H	-6.62067700	1.14094000	-0.64953700
H	-5.19594900	0.64033100	0.22955900
C	-3.42262300	2.58427300	-0.75322400
C	-2.84775400	2.01542200	0.54630400
C	-1.34174400	2.25937300	0.70304400
C	-0.78767800	1.67195500	1.97425500
C	0.46810800	1.25777100	2.19766500
C	1.61504000	1.27872100	1.22509100
C	2.63681300	0.16087400	1.47040800
C	3.73262200	0.09795700	0.40666400
C	4.74643700	-1.02255100	0.65519900
C	5.80292800	-1.17240400	-0.44592100
C	6.73672700	0.03194300	-0.59742500
C	7.84467900	-0.19381200	-1.62959400
C	8.77307800	1.01183000	-1.78381200
H	9.55420900	0.82201100	-2.52387600
H	9.26439900	1.25429300	-0.83669700
H	8.21845600	1.89867500	-2.10570900
H	7.39046600	-0.43432800	-2.59827400
H	8.43034300	-1.07426200	-1.34087700
H	7.19030800	0.26108200	0.37561300

H	6.16292300	0.92101900	-0.88399100
H	5.30393100	-1.36756500	-1.40411900
H	6.40621500	-2.06166100	-0.23251000
H	4.20648800	-1.97235700	0.76164400
H	5.24627200	-0.84843000	1.61583800
H	3.27538200	-0.04058800	-0.58299500
H	4.24348000	1.06510600	0.36765000
H	3.08948600	0.29902700	2.45855400
H	2.12504800	-0.81051800	1.51992900
H	2.12891000	2.24488000	1.30988200
H	1.25069600	1.23997100	0.19234100
H	0.71339200	0.91642900	3.20223900
H	-1.48209600	1.64078400	2.81247100
H	-1.15467100	3.34103200	0.70762100
H	-0.80265900	1.87969200	-0.17306400
H	-3.04616100	0.94000500	0.59444200
H	-3.36877100	2.46219400	1.40165600
H	-2.95988600	2.07476000	-1.60650000
H	-3.13269100	3.63663800	-0.84291600
H	-5.38866200	3.11887300	-0.05619400
H	-5.28121900	2.93544700	-1.79352100
N	-0.88462200	-1.39657800	0.82722000
C	-0.09779000	-1.50096300	-0.43665300
H	0.96383600	-1.44977100	-0.20700900
H	-0.38361800	-0.67891600	-1.08929300
H	-0.33514000	-2.44868400	-0.91658100
C	-0.59854200	-2.47497500	1.81583100
H	-1.21613200	-2.31439300	2.69781300
H	0.45460500	-2.43960600	2.08873700
H	-0.83812000	-3.43733100	1.36683300
H	-1.88967600	-1.42676800	0.56044900
H	-0.70581800	-0.47688100	1.27313500

conf_158

C	-3.48902400	0.64214300	-1.46697400
C	-3.04856700	2.00152500	-2.03049200
C	-1.93658800	2.74005100	-1.27324600
C	-2.35208800	3.22639400	0.12613600
C	-2.34552400	2.13368200	1.16213800
O	-1.46263100	1.29959300	1.23455000
O	-3.33657200	2.08900800	2.06577700
H	-3.98496400	2.78946000	1.91262900
H	-3.32716800	3.72223100	0.08876300
H	-1.63171200	3.97042300	0.48579700
H	-1.04084500	2.12155300	-1.17959600
H	-1.64681500	3.61945600	-1.85282900
H	-3.92290500	2.65952000	-2.09275600
H	-2.71430900	1.85284200	-3.06242500
C	-2.41886200	-0.45243500	-1.53961300
C	-2.97740400	-1.85080300	-1.25765800
C	-1.95396700	-2.98986700	-1.45438200
C	-0.97420100	-3.18467200	-0.32546500
C	0.26482700	-2.68128700	-0.22483700

C	0.94802600	-1.74315400	-1.17349000
C	1.34075000	-0.41671500	-0.49751700
C	1.95409000	0.60653800	-1.46123700
C	3.24808200	0.15264900	-2.15412100
C	4.37084500	-0.28955200	-1.20606300
C	4.82248000	0.78554900	-0.21431900
C	6.00163400	0.34538100	0.65700100
C	6.44232200	1.41713500	1.65520900
H	6.75144700	2.33132000	1.13989500
H	7.28534600	1.07708900	2.26150000
H	5.62743900	1.68199400	2.33681300
H	6.84477100	0.07628600	0.01088900
H	5.72935300	-0.57047400	1.19620600
H	3.98801800	1.07184200	0.43811000
H	5.09830700	1.69308600	-0.76615800
H	5.23048900	-0.59887900	-1.81008700
H	4.06434700	-1.18566100	-0.65159500
H	3.61078000	0.97922200	-2.77462400
H	3.02543000	-0.66396700	-2.84911200
H	1.21199300	0.85582000	-2.22910800
H	2.13984100	1.53269800	-0.90866200
H	0.45380700	0.03374800	-0.03997100
H	2.04318100	-0.62350100	0.31857300
H	0.31429100	-1.53540800	-2.03790400
H	1.85144500	-2.23078100	-1.55496000
H	0.86847800	-2.99079600	0.62959500
H	-1.30255700	-3.86794700	0.45825400
H	-1.42052600	-2.82085000	-2.39343800
H	-2.50033800	-3.92878200	-1.57910200
H	-3.81970100	-2.03040300	-1.93302400
H	-3.39701800	-1.89596800	-0.24367200
H	-1.98548700	-0.45043200	-2.54686600
H	-1.59775800	-0.22251800	-0.85589500
H	-4.36210100	0.31401700	-2.04088700
H	-3.84884900	0.74337900	-0.43462200
N	-1.19602900	-1.18903300	2.25212900
C	0.00234200	-1.21775400	3.13658200
H	-0.20019600	-0.62395400	4.02638200
H	0.84387000	-0.79424400	2.59192800
H	0.21863300	-2.24742400	3.41786800
C	-2.43181000	-1.74166500	2.87123800
H	-2.26168400	-2.77959300	3.15347500
H	-3.24192400	-1.68235200	2.14675700
H	-2.68164100	-1.15172900	3.75147300
H	-0.98285400	-1.71176800	1.38187400
H	-1.36825300	-0.20299500	1.94964800

conf_116

C	4.11669700	0.76262600	-1.74639300
C	4.48267500	1.50410800	-0.45612100
C	3.97944900	2.95115200	-0.38951300
C	2.43628600	3.08579100	-0.43203400
C	1.78951000	2.17301800	0.57065000

O	1.14902500	1.18099200	0.27921500
O	1.98211200	2.43489500	1.87853200
H	2.50813300	3.23635100	2.00653900
H	2.04591400	2.81570800	-1.41129100
H	2.14540600	4.12121400	-0.22872100
H	4.36651400	3.42817200	0.51772600
H	4.37878300	3.52943700	-1.22677400
H	5.57182800	1.51425600	-0.34923300
H	4.10735400	0.95144600	0.41397200
C	4.58025500	-0.70148300	-1.75509900
C	3.80885400	-1.63816000	-0.81417700
C	2.34483800	-1.85343800	-1.22081500
C	1.64944500	-2.93295300	-0.43429800
C	0.32241800	-3.08493500	-0.31298900
C	-0.72978100	-2.18150600	-0.90558300
C	-2.10583600	-2.34708500	-0.24835500
C	-3.09286100	-1.24426500	-0.62969700
C	-4.46236500	-1.40653100	0.03551600
C	-5.47611000	-0.31912900	-0.33982400
C	-5.10116900	1.08380600	0.15184700
C	-6.19277000	2.13795600	-0.07597600
C	-6.49380500	2.41279500	-1.55184800
H	-6.89965400	1.53225500	-2.05577600
H	-5.58760900	2.71470800	-2.08742400
H	-7.22584600	3.21665500	-1.65975000
H	-5.88614000	3.07193200	0.40706300
H	-7.11033000	1.81935800	0.43231600
H	-4.18013500	1.42007300	-0.34007300
H	-4.87794500	1.03013300	1.22557000
H	-6.45170100	-0.58808200	0.08045000
H	-5.60450000	-0.31287300	-1.42750600
H	-4.33645100	-1.42034700	1.12685500
H	-4.87060800	-2.38680200	-0.23437500
H	-2.66054200	-0.27149600	-0.36435900
H	-3.21774000	-1.22786400	-1.71915900
H	-2.51829300	-3.32588200	-0.51579600
H	-1.99559800	-2.36937800	0.84402200
H	-0.81855700	-2.38562200	-1.97991600
H	-0.41024500	-1.13267000	-0.84764000
H	-0.04344200	-3.96541900	0.21261900
H	2.29100800	-3.69212700	0.00955100
H	2.31991800	-2.14154500	-2.28106600
H	1.78354400	-0.91610200	-1.16327000
H	3.85818300	-1.26318600	0.21591000
H	4.31486100	-2.60897800	-0.80072800
H	4.49764700	-1.09416500	-2.77400900
H	5.64506800	-0.73871300	-1.50159800
H	4.57730500	1.28566000	-2.59104000
H	3.03675800	0.80380500	-1.91696400
N	0.66309400	-0.89928800	1.98669500
C	-0.61745000	-0.68019300	2.71639300
H	-1.40712100	-0.49430900	1.99168800
H	-0.50247700	0.18474500	3.36782900
H	-0.85351600	-1.56348800	3.30760300
C	1.84590500	-1.09007300	2.87151500

H	2.71364300	-1.29965800	2.24852200
H	1.66285700	-1.92768000	3.54279900
H	2.00833500	-0.17755400	3.44223900
H	0.83505400	-0.07699100	1.36300000
H	0.59447500	-1.71604900	1.35216500

conf_167

C	-0.04207400	2.11556400	-1.73853000
C	1.03593200	3.17207300	-1.45995500
C	1.07937000	3.69997800	-0.01826300
C	1.56021000	2.66510500	1.01477800
C	0.51731700	1.65788400	1.42355300
O	-0.64438600	1.93240800	1.66277600
O	0.89535400	0.37617900	1.57415900
H	1.80755700	0.22002100	1.28236500
H	1.83384400	3.18024700	1.94323800
H	2.46273400	2.15685900	0.66187800
H	1.76849100	4.54635000	0.02454100
H	0.10382000	4.07986200	0.29456000
H	0.88095200	4.02052900	-2.13415300
H	2.02108900	2.76368600	-1.71297400
C	-1.47680500	2.68063100	-1.74617000
C	-2.52735100	1.69089200	-1.23282800
C	-2.60155600	0.37429600	-2.02488000
C	-3.37177400	-0.69653600	-1.30436000
C	-2.96014100	-1.94834300	-1.04933200
C	-1.64352400	-2.55552500	-1.44577900
C	-1.03496100	-3.49201500	-0.38468400
C	0.48186100	-3.66121400	-0.55545900
C	1.25084500	-2.42225700	-0.08690300
C	2.74565200	-2.42339800	-0.40860400
C	3.44453300	-1.14083000	0.05205500
C	4.93772700	-1.07041500	-0.27529400
C	5.58273300	0.23825300	0.18288100
H	5.48814500	0.36909300	1.26572300
H	5.11471700	1.10096800	-0.30323900
H	6.64718000	0.26489200	-0.05883100
H	5.07098400	-1.19069300	-1.35567700
H	5.44676800	-1.91894300	0.19359200
H	3.32844900	-1.04192400	1.14389200
H	2.94631700	-0.27819600	-0.41800600
H	2.88613800	-2.53874700	-1.48923800
H	3.22782100	-3.28889900	0.05894000
H	1.11689200	-2.32078200	0.99790500
H	0.80264000	-1.52493800	-0.53059700
H	0.82530600	-4.53478500	0.00689000
H	0.70800400	-3.86542000	-1.60848300
H	-1.23196500	-3.09668800	0.61971100
H	-1.53575800	-4.46323200	-0.42759300
H	-0.93123900	-1.76665300	-1.69437700
H	-1.78414300	-3.12724100	-2.37135800
H	-3.65992400	-2.62173600	-0.55509800
H	-4.38038800	-0.42203100	-0.99631100

H	-1.59585800	0.02182300	-2.25590600
H	-3.08237600	0.56855900	-2.99136300
H	-3.51360900	2.16671200	-1.22069500
H	-2.27616500	1.47679900	-0.19041100
H	-1.52552500	3.57287700	-1.11583200
H	-1.73131200	3.01094200	-2.75839000
H	0.03227000	1.30671600	-1.00071200
H	0.17379600	1.64163700	-2.69995800
N	-2.38586000	-0.22044500	1.84632700
C	-3.71810900	0.31270800	2.25255200
H	-3.62267700	0.79150700	3.22539500
H	-4.43157300	-0.50803900	2.30642400
H	-4.04134100	1.04114300	1.51180700
C	-1.82594600	-1.23828100	2.78032000
H	-2.48280300	-2.10620500	2.80276300
H	-1.75486100	-0.79783300	3.77349200
H	-0.83502900	-1.51737000	2.43088000
H	-1.71102400	0.57845100	1.77642500
H	-2.47670700	-0.62632100	0.89841400

conf_186

C	4.02493200	-1.90194800	-1.09261500
C	5.53683100	-1.66222400	-1.19726600
C	6.10949800	-0.51316700	-0.35652700
C	5.59780300	0.88770900	-0.76151400
C	4.23052500	1.25811600	-0.25485000
O	3.31772800	1.65845500	-0.95169100
O	4.01823700	1.17315500	1.07402500
H	4.80098300	0.83731700	1.53285200
H	5.58144200	0.99439900	-1.84615400
H	6.28209400	1.65118000	-0.37119800
H	5.93866200	-0.69927500	0.71115000
H	7.19511600	-0.50473700	-0.47617300
H	5.79385500	-1.47539300	-2.24543400
H	6.06212600	-2.58078100	-0.91609800
C	3.54879400	-2.38626000	0.28224100
C	2.06735600	-2.78961700	0.31076500
C	1.06798100	-1.62987200	0.11604700
C	1.03363900	-0.68766700	1.28678100
C	-0.04252200	-0.33360800	2.00447200
C	-1.46675700	-0.75523900	1.76883100
C	-2.22600000	0.24039700	0.87355400
C	-3.70947100	-0.08710600	0.70294000
C	-4.42914500	0.89651300	-0.22470000
C	-5.93705600	0.64969800	-0.35675500
C	-6.29710800	-0.68635100	-1.01634700
C	-7.79557900	-0.85646900	-1.29923400
C	-8.66601600	-0.90057500	-0.04029700
H	-8.63250500	0.04170400	0.51250600
H	-8.33648400	-1.69591300	0.63612600
H	-9.71137700	-1.09310000	-0.29303100
H	-7.94118900	-1.78079300	-1.86773200
H	-8.13314700	-0.04162700	-1.95072100

H	-5.96156100	-1.51793300	-0.38555200
H	-5.74428300	-0.77277300	-1.96032000
H	-6.37590900	1.46266200	-0.94660700
H	-6.39434000	0.71279500	0.63651200
H	-3.96945500	0.85430400	-1.22156400
H	-4.26812200	1.91603800	0.14840700
H	-3.80541700	-1.10649200	0.31607700
H	-4.19559100	-0.08327400	1.68564000
H	-2.12651100	1.25279600	1.28959600
H	-1.74920600	0.25360200	-0.11554100
H	-1.98007300	-0.83060500	2.73196200
H	-1.51020300	-1.74785200	1.31365600
H	0.11478500	0.34430800	2.84242500
H	1.99370700	-0.27848100	1.59179400
H	0.07361800	-2.04060900	-0.06908300
H	1.34169400	-1.07771500	-0.79337200
H	1.84266100	-3.28437200	1.26030600
H	1.89176400	-3.53185400	-0.47470500
H	4.14729200	-3.25661800	0.57139000
H	3.74010100	-1.62724500	1.04848200
H	3.75581400	-2.66074200	-1.83520200
H	3.48385100	-0.99900400	-1.39357500
N	0.74408700	2.12522100	-0.11246500
C	0.69187700	3.24335100	0.87008900
H	1.04109100	4.15511200	0.38829500
H	1.33932100	2.99681100	1.70981500
H	-0.33336700	3.37158900	1.21343100
C	-0.08950000	2.33675100	-1.32900700
H	0.26924100	3.22269400	-1.85041700
H	-1.12929600	2.46751200	-1.03580100
H	0.00777600	1.46616400	-1.97512200
H	0.46534700	1.25274600	0.37131600
H	1.73888500	1.96942800	-0.39939300

9E_NMe2H2

conf_0

C	4.91647900	0.44875400	-1.31816900
C	5.28912400	-0.08156400	0.07020000
C	5.68489600	-1.56316100	0.09840700
C	4.58263000	-2.52222300	-0.40992700
C	3.25458800	-2.27084800	0.25200000
O	2.25708100	-1.90769800	-0.34256400
O	3.17469000	-2.42660000	1.58506800
H	4.02392600	-2.69782700	1.96044600
H	4.42891200	-2.41042700	-1.48148800
H	4.87813500	-3.56040700	-0.22405800
H	6.56923900	-1.73059500	-0.52208400
H	5.98026200	-1.84007800	1.11726100
H	6.12289000	0.50523900	0.46889700
H	4.45711300	0.07884500	0.76629600
C	4.56886200	1.94532000	-1.33379600
C	3.42076000	2.36020500	-0.40235000
C	2.09053000	1.63827500	-0.68447100

C	1.03171500	2.00852500	0.31599400
C	-0.10050900	2.66683600	0.04535900
C	-1.08884500	3.16569400	1.06122700
C	-2.55698900	2.96716200	0.65608900
C	-2.94392700	1.50419200	0.44179000
C	-4.42890300	1.29517000	0.13942800
C	-4.79742000	-0.16544200	-0.12891700
C	-6.29147100	-0.38784000	-0.37231000
C	-6.65121900	-1.84883400	-0.65332100
C	-8.14699500	-2.06072100	-0.89276000
H	-8.73254200	-1.75139100	-0.02208000
H	-8.49479300	-1.47694500	-1.75000400
H	-8.37480200	-3.11075300	-1.09088300
H	-6.08750400	-2.19693100	-1.52766500
H	-6.32376500	-2.46920800	0.19009400
H	-6.85492900	-0.03645200	0.50041100
H	-6.61579600	0.23443700	-1.21510400
H	-4.47916100	-0.78440800	0.72205300
H	-4.23919600	-0.52522500	-1.00433300
H	-5.02173900	1.67099000	0.98108600
H	-4.70967300	1.90453900	-0.72722900
H	-2.34967200	1.09591500	-0.38686700
H	-2.67761400	0.92941000	1.34048900
H	-3.19746700	3.40329800	1.42870600
H	-2.75831000	3.53068100	-0.26208700
H	-0.91361100	4.23909500	1.20413700
H	-0.89502300	2.69427600	2.03176600
H	-0.31071100	2.92812400	-0.99258700
H	1.26681000	1.79267400	1.36099800
H	2.26579600	0.55728100	-0.66205500
H	1.74748600	1.87746900	-1.69664500
H	3.25990500	3.43836400	-0.49303300
H	3.70566400	2.18893400	0.64161100
H	4.32085100	2.23485000	-2.36052700
H	5.45879500	2.52276200	-1.06173600
H	4.07631500	-0.12267700	-1.72474300
H	5.75506600	0.27759500	-2.00196300
N	-0.18481600	-0.91331600	0.40067800
C	-1.12222900	-1.59022100	-0.54173200
H	-1.00330400	-2.66705100	-0.43344500
H	-2.14320100	-1.29814000	-0.30898900
H	-0.86832200	-1.29394500	-1.55791400
C	-0.45575700	-1.19828900	1.83825000
H	-1.46695400	-0.87984000	2.08222800
H	0.26640100	-0.65428600	2.44418100
H	-0.34545700	-2.26767500	2.00915700
H	0.78282200	-1.23812800	0.17858900
H	-0.19522400	0.10927300	0.24839000

conf_3

C	-5.49524800	1.11765200	0.49254800
C	-5.59427600	0.35328700	-0.83161800
C	-6.24422700	-1.03168800	-0.71620200

C	-5.52289500	-1.99411900	0.25579600
C	-4.04516600	-2.09390100	-0.01260500
O	-3.18528700	-1.79841000	0.79578300
O	-3.65227900	-2.50819100	-1.23048900
H	-4.41066900	-2.69981600	-1.79965800
H	-5.63531000	-1.66863100	1.28819000
H	-5.95869600	-2.99617800	0.17723000
H	-7.27588300	-0.93601900	-0.36742800
H	-6.31070900	-1.48531000	-1.71237000
H	-6.17166800	0.94547000	-1.54878800
H	-4.59709000	0.24501700	-1.27496300
C	-4.89144900	2.52387000	0.35340300
C	-3.49511000	2.57704800	-0.28433000
C	-2.45213800	1.70051200	0.42845700
C	-1.11017900	1.74175700	-0.24703600
C	0.05246900	2.03843800	0.34575600
C	1.38524100	2.13066400	-0.33682600
C	2.43067700	1.16052900	0.23577500
C	3.82211900	1.33281200	-0.37526100
C	4.84398000	0.32899300	0.16241400
C	6.24264900	0.50752200	-0.43215200
C	7.26164500	-0.50205100	0.09997700
C	8.66264700	-0.32089800	-0.48960200
C	9.67326700	-1.33543200	0.04784000
H	10.66308300	-1.18252500	-0.38894600
H	9.77331100	-1.25438900	1.13448300
H	9.36355300	-2.35985100	-0.18086000
H	8.60677100	-0.40089400	-1.58164300
H	9.01358100	0.69545000	-0.27609800
H	7.31681100	-0.42051900	1.19300800
H	6.90995500	-1.52014000	-0.11222600
H	6.59583000	1.52441100	-0.22230800
H	6.18532000	0.42452400	-1.52458800
H	4.90121200	0.41878700	1.25457100
H	4.49374000	-0.69188400	-0.04274400
H	3.75466400	1.23724000	-1.46578600
H	4.17357000	2.35243100	-0.17990000
H	2.49126900	1.28900000	1.32347400
H	2.09674600	0.12769500	0.06537300
H	1.76508100	3.15310700	-0.22172800
H	1.26837300	1.96225100	-1.41237100
H	0.04726900	2.26203000	1.41354300
H	-1.11045500	1.55623500	-1.32300500
H	-2.82195700	0.66893900	0.44801600
H	-2.35268000	2.01097600	1.47417000
H	-3.14551600	3.61353400	-0.28906000
H	-3.55247300	2.27563100	-1.33622500
H	-4.84984100	2.98296100	1.34679000
H	-5.56603800	3.14820900	-0.24178700
H	-4.90695700	0.54187000	1.21402600
H	-6.49644900	1.20916100	0.92755400
N	-0.50211600	-1.28117800	0.53027800
C	0.14941200	-1.59651800	1.83338900
H	1.20522000	-1.33778900	1.77824600
H	-0.33681800	-1.01484900	2.61456400

H	0.03220800	-2.65930200	2.03815000
C	0.06496700	-2.01523300	-0.63590300
H	-0.05641400	-3.08454100	-0.47148700
H	1.12035300	-1.76944800	-0.73849400
H	-0.47858600	-1.71927100	-1.53134600
H	-1.52153200	-1.50183300	0.59667000
H	-0.43714700	-0.26014500	0.35852700

conf_1

C	4.76634600	0.60148100	-0.93554800
C	4.96555800	-0.12496100	0.39866500
C	5.32693600	-1.60958400	0.26517800
C	4.27477800	-2.45109900	-0.49532600
C	2.88267400	-2.24395300	0.03758000
O	1.97158700	-1.76408400	-0.61049100
O	2.64060500	-2.57955300	1.31686900
H	3.43285200	-2.93232500	1.74530500
H	4.25218200	-2.18932400	-1.55142100
H	4.52592500	-3.51471300	-0.42260800
H	6.27524400	-1.72185700	-0.26700400
H	5.49399100	-2.03302100	1.26253900
H	5.75869900	0.37272100	0.96587300
H	4.06177600	-0.03137700	1.01260400
C	4.45733200	2.09872800	-0.78212000
C	3.22116600	2.42820600	0.06714100
C	1.91556800	1.80016800	-0.45375000
C	0.75721100	2.08095700	0.46220900
C	-0.31607500	2.81801100	0.15652000
C	-1.40094000	3.22725300	1.11216900
C	-2.81715700	3.14346900	0.52367600
C	-3.21816800	1.73378600	0.09057400
C	-4.65956500	1.63687600	-0.41866400
C	-5.03389800	0.26364300	-0.98894500
C	-5.03657500	-0.87479900	0.03532900
C	-5.35791300	-2.24209100	-0.57248400
C	-5.31864500	-3.37717200	0.45155700
H	-5.55356600	-4.33998900	-0.00779700
H	-4.32815500	-3.46194200	0.91246000
H	-6.04061600	-3.20785200	1.25530900
H	-6.34666100	-2.20279500	-1.04171200
H	-4.65080400	-2.45358400	-1.38522600
H	-4.06403900	-0.93578300	0.54125900
H	-5.76123800	-0.64670100	0.82594400
H	-4.34979000	0.01754800	-1.81325400
H	-6.02776900	0.32513100	-1.44368400
H	-5.34659400	1.89530700	0.39542000
H	-4.80739600	2.39562700	-1.19451800
H	-2.53959200	1.39962700	-0.70645800
H	-3.07615800	1.05274900	0.93907100
H	-3.53018600	3.51222100	1.26739100
H	-2.89301700	3.82054900	-0.33485700
H	-1.21306700	4.26607200	1.41042000
H	-1.33405200	2.63056300	2.02928100

H	-0.39560100	3.21976000	-0.85450000
H	0.86240400	1.72184400	1.48871300
H	2.06211100	0.71934200	-0.55426900
H	1.69776900	2.17994200	-1.45765500
H	3.09542900	3.51406400	0.10738900
H	3.38260800	2.10886700	1.10270300
H	4.33364900	2.53312100	-1.77988100
H	5.32389300	2.59978600	-0.33776300
H	3.96511300	0.12283500	-1.50707000
H	5.67343200	0.49197100	-1.53997200
N	-0.51585200	-0.78448700	-0.01406000
C	-1.35324000	-1.28773900	-1.14163300
H	-2.38424200	-0.98007400	-0.99180200
H	-0.96703000	-0.87556500	-2.07206300
H	-1.28492800	-2.37403700	-1.16475200
C	-0.95563200	-1.25343000	1.33082000
H	-0.28937100	-0.83249000	2.08144900
H	-0.89809300	-2.34010100	1.35754300
H	-1.97774700	-0.92726300	1.50865600
H	0.46285700	-1.11369400	-0.17120900
H	-0.48533600	0.24885000	-0.02013200

conf_45

C	-1.27214900	-2.97057000	1.71043500
C	-2.11814300	-1.80533200	1.18540200
C	-2.95001300	-2.12745000	-0.06301800
C	-2.12975600	-2.65171700	-1.26033400
C	-0.89534500	-1.84997100	-1.57927400
O	0.19863300	-2.34798800	-1.77618200
O	-0.99478800	-0.51208300	-1.65489500
H	-1.88217200	-0.18683000	-1.43533600
H	-1.79539600	-3.67501700	-1.09794000
H	-2.75429600	-2.65968900	-2.16133800
H	-3.70025000	-2.88811900	0.16824900
H	-3.51804800	-1.23817700	-0.35891800
H	-2.79500800	-1.46235600	1.97465800
H	-1.46896000	-0.95151600	0.96502800
C	-0.42999500	-2.61394000	2.94749000
C	0.45488900	-1.36975900	2.78376000
C	1.48542300	-1.45674300	1.63546100
C	1.85023900	-0.09107800	1.13078500
C	3.05576400	0.48637700	1.18031300
C	3.39483700	1.88262000	0.72076000
C	2.22194000	2.71996100	0.19797400
C	1.27347200	3.21503600	1.30222900
C	-0.09124800	3.69068200	0.78765300
C	-0.98010400	2.56086300	0.25462900
C	-2.38633800	3.02014800	-0.13552700
C	-3.24869300	1.90215100	-0.72903700
C	-4.69258400	2.32308200	-1.00768600
H	-5.27573800	1.50676300	-1.44133800
H	-5.19032000	2.63632400	-0.08631000
H	-4.72602200	3.16398800	-1.70533100

H	-2.79001300	1.56855800	-1.67239900
H	-3.24670600	1.04925200	-0.03637000
H	-2.89068700	3.42866700	0.74753600
H	-2.31947200	3.84299500	-0.85652300
H	-1.06218200	1.77954800	1.02230600
H	-0.50687300	2.08889700	-0.61490300
H	-0.61859500	4.19605800	1.60335100
H	0.04931800	4.44467100	0.00360700
H	1.11613700	2.42469000	2.04301600
H	1.76610000	4.03316400	1.83669600
H	2.61683700	3.58556400	-0.34215500
H	1.66287800	2.13898000	-0.54376500
H	4.18088700	1.81630400	-0.04431700
H	3.87518100	2.40616300	1.55718500
H	3.88164800	-0.07950500	1.61087800
H	1.01143000	0.48060800	0.73990000
H	1.05273900	-2.03368700	0.81006900
H	2.37479800	-2.00200800	1.96521100
H	0.98679100	-1.17411000	3.71864900
H	-0.18139400	-0.49383600	2.62300200
H	0.19051700	-3.47696900	3.21080400
H	-1.09716900	-2.44750900	3.79966800
H	-0.61295000	-3.33870400	0.91791400
H	-1.92911000	-3.81000900	1.96156800
N	2.48812800	-0.84802400	-1.82480900
C	3.66955900	-1.73514700	-2.01200600
H	4.58045800	-1.14229600	-1.94210600
H	3.66047400	-2.49593100	-1.23335800
H	3.60731300	-2.20860000	-2.99039700
C	2.37708800	0.25613100	-2.81790000
H	3.24076200	0.91279100	-2.72750000
H	1.46169700	0.80921000	-2.61731000
H	2.33659000	-0.17161800	-3.81849600
H	1.61055400	-1.42078800	-1.85716000
H	2.51753000	-0.45126200	-0.86639300

conf_52

C	3.95680700	-0.12443000	-1.66632900
C	3.44255200	1.29198300	-1.38793700
C	4.31298200	2.10501400	-0.42221400
C	4.42916800	1.48943700	0.99574200
C	3.07085400	1.15271600	1.54230500
O	2.64017600	0.02018300	1.64794400
O	2.25458500	2.17169300	1.87392000
H	2.68758300	3.02748400	1.75010400
H	5.00632700	0.56705300	0.97464800
H	4.93732000	2.18912500	1.66644500
H	5.32759000	2.20098300	-0.81762900
H	3.91853000	3.12439800	-0.34952400
H	3.37779400	1.83899900	-2.33376900
H	2.41682200	1.24980400	-1.00163600
C	3.04468300	-0.92688600	-2.60515000
C	1.69549600	-1.35683600	-2.00812600

C	1.82753500	-2.39150900	-0.88761600
C	0.54070500	-2.99034200	-0.38296500
C	-0.70053500	-2.68233000	-0.77985100
C	-1.94998300	-3.38725000	-0.32862500
C	-2.97376900	-2.49061700	0.39198400
C	-3.42257900	-1.26415700	-0.40518200
C	-4.49147100	-0.43802200	0.31717600
C	-4.86644200	0.86671500	-0.39634900
C	-3.75340100	1.91938600	-0.41006100
C	-4.16417400	3.23433200	-1.07685500
C	-3.04275000	4.27426800	-1.08484000
H	-3.36119900	5.20261300	-1.56438300
H	-2.72591400	4.52049100	-0.06609200
H	-2.16662200	3.90533700	-1.62851500
H	-4.48439100	3.03058800	-2.10472000
H	-5.04003500	3.64135900	-0.55973900
H	-3.44283100	2.12668900	0.62393400
H	-2.86944500	1.52687400	-0.92841600
H	-5.74813000	1.29580900	0.09098800
H	-5.16791500	0.64307300	-1.42662400
H	-5.38729400	-1.05519600	0.44162700
H	-4.15163800	-0.20348200	1.33630000
H	-2.55710100	-0.63259800	-0.62999800
H	-3.81430800	-1.58708500	-1.37701100
H	-3.84857200	-3.09800900	0.64473100
H	-2.55742400	-2.16577400	1.35557200
H	-1.68381900	-4.23010900	0.31628200
H	-2.43489500	-3.81563800	-1.21435800
H	-0.83303400	-1.90781900	-1.53097100
H	0.65980700	-3.79706900	0.34182800
H	2.38303100	-1.96875300	-0.04109700
H	2.45335800	-3.22098000	-1.24222700
H	1.08696800	-1.78206300	-2.81193700
H	1.14451800	-0.47883800	-1.64821800
H	3.57723600	-1.82471600	-2.93569600
H	2.85895800	-0.33624300	-3.50862100
H	4.08888300	-0.67266800	-0.72891100
H	4.95189900	-0.05103800	-2.11786700
N	0.00516300	-0.70652400	1.80378800
C	-0.21424900	-1.38981600	3.10935300
H	0.45124000	-2.24924200	3.16975900
H	0.01100100	-0.69163100	3.91382300
H	-1.25065400	-1.71546400	3.17681200
C	-0.85825800	0.48802400	1.58443800
H	-0.61972900	1.23619800	2.33797100
H	-1.90312000	0.19676300	1.65459200
H	-0.65623100	0.88990900	0.59368800
H	1.00654900	-0.41053100	1.75189300
H	-0.13436700	-1.38602000	1.03216000

conf_128

C	-5.68042600	-0.91826800	-0.10509500
C	-5.39574900	0.53813200	0.28581100

C	-5.12530200	1.46102200	-0.91025000
C	-4.46072900	2.77612900	-0.49445200
C	-3.04277600	2.56071400	-0.02130700
O	-2.35014000	1.62985500	-0.38408000
O	-2.51295900	3.45318800	0.83118700
H	-3.15153000	4.14104400	1.06401600
H	-4.40159600	3.47051300	-1.34202000
H	-5.03864000	3.28405100	0.28553600
H	-4.47531600	0.97022300	-1.63646900
H	-6.06222200	1.68598600	-1.42584900
H	-6.24619400	0.93018400	0.85324200
H	-4.54183700	0.56267700	0.97222600
C	-4.49940400	-1.65958000	-0.74968800
C	-3.25425600	-1.75604400	0.13734900
C	-2.13897900	-2.59427000	-0.50242500
C	-0.93068500	-2.77582700	0.37308600
C	0.34455000	-2.78715400	-0.03668200
C	1.51489500	-3.15967400	0.83210900
C	2.73822900	-2.23185300	0.74668400
C	3.38838300	-2.17225500	-0.64093200
C	4.75167300	-1.46894900	-0.66381900
C	4.70425700	0.03042000	-0.35740900
C	6.07699000	0.70476700	-0.40026800
C	6.02606700	2.20552000	-0.10368900
C	7.40205900	2.87211800	-0.14820000
H	7.86210900	2.75842600	-1.13404200
H	7.33585100	3.94136700	0.06685200
H	8.07891200	2.42585000	0.58611600
H	5.35983400	2.69287200	-0.82663700
H	5.57531500	2.36225900	0.88441600
H	6.74212700	0.21462200	0.32076700
H	6.52715500	0.54464200	-1.38727800
H	4.26403200	0.19844100	0.63423700
H	4.04510200	0.52399000	-1.08511900
H	5.42029300	-1.96289300	0.05111800
H	5.20499000	-1.61060000	-1.65044600
H	3.51504200	-3.19818300	-1.00420800
H	2.71762400	-1.68134800	-1.35762100
H	2.46277100	-1.22590300	1.08561100
H	3.48284800	-2.58768900	1.46648800
H	1.83919700	-4.16667000	0.53837200
H	1.18045900	-3.24069800	1.87118500
H	0.55139200	-2.61752500	-1.09362800
H	-1.13544400	-2.99936600	1.42132200
H	-1.84672200	-2.16869700	-1.46925800
H	-2.54395100	-3.59045900	-0.72424100
H	-3.52806200	-2.20013600	1.10232200
H	-2.87859800	-0.75071100	0.35200700
H	-4.22657300	-1.18673200	-1.69959900
H	-4.82840700	-2.67214600	-1.00635400
H	-6.53389500	-0.94646900	-0.79108700
H	-5.99393900	-1.46457500	0.79091200
N	-0.05006900	0.42604500	0.47382300
C	1.02394500	1.01906600	-0.37290000
H	1.98872300	0.62389000	-0.06754300

H	0.82867500	0.76294200	-1.41271000
H	1.00566700	2.10079700	-0.25078000
C	0.11067400	0.68308200	1.93260400
H	1.06704800	0.28308600	2.26444400
H	-0.70198100	0.19025000	2.46379900
H	0.06980100	1.75695900	2.10659900
H	-0.95763100	0.83529900	0.16520100
H	-0.09763900	-0.59881400	0.32132100

conf_55

C	3.30878700	-2.86702800	0.64848300
C	3.98539000	-2.49588500	-0.67517500
C	5.47668700	-2.15750600	-0.55144400
C	5.78231300	-0.96451500	0.38269600
C	4.97713000	0.26360900	0.05012900
O	4.22393600	0.81552800	0.83021200
O	5.08013500	0.76703200	-1.19244200
H	5.68911200	0.24651000	-1.73473800
H	5.56882800	-1.21101800	1.42105000
H	6.84526100	-0.70640600	0.31886100
H	6.02817200	-3.01902500	-0.16568400
H	5.88956400	-1.96742400	-1.54960200
H	3.87784600	-3.32621400	-1.38046100
H	3.46389500	-1.64859700	-1.13640100
C	1.82478100	-3.23984700	0.50294500
C	0.94353200	-2.16577800	-0.15200800
C	0.99324700	-0.80380200	0.55906200
C	0.22460700	0.25745900	-0.17492500
C	-0.71075800	1.05652900	0.35148000
C	-1.50571600	2.08412900	-0.40160400
C	-3.00634600	1.73554200	-0.46697700
C	-3.29493300	0.46470400	-1.27027600
C	-4.78816700	0.15235500	-1.42546300
C	-5.50470900	-0.18162500	-0.11348400
C	-6.96467500	-0.59309400	-0.31531600
C	-7.69125100	-0.92364900	0.99066500
C	-9.14855200	-1.33636500	0.77664000
H	-9.64262400	-1.56611100	1.72395500
H	-9.71647300	-0.53768800	0.29002200
H	-9.21693600	-2.22463900	0.14141000
H	-7.15417300	-1.72795400	1.50775300
H	-7.65057100	-0.05269500	1.65586100
H	-7.49983700	0.21310400	-0.83265600
H	-7.00688000	-1.46261000	-0.98315900
H	-5.47079800	0.67921100	0.56425000
H	-4.96658800	-0.99311500	0.39427800
H	-5.28904200	1.00083200	-1.90795400
H	-4.89785700	-0.69406500	-2.11242600
H	-2.84565800	0.57010700	-2.26561400
H	-2.79491100	-0.38934900	-0.79888800
H	-3.39168900	1.63644000	0.55274900
H	-3.53899700	2.57984400	-0.91722300
H	-1.11041600	2.18795800	-1.41866400

H	-1.40108300	3.06094800	0.08903400
H	-0.97028500	0.92942000	1.40341200
H	0.44357500	0.35283900	-1.24047200
H	2.04131200	-0.49352100	0.64513600
H	0.61650300	-0.89898100	1.58297600
H	-0.09174700	-2.51813500	-0.17598000
H	1.23711800	-2.03089500	-1.19893500
H	1.42706200	-3.47594400	1.49559300
H	1.74113500	-4.15998400	-0.08477700
H	3.40674800	-2.04367500	1.36310700
H	3.83966100	-3.71477500	1.09494000
N	2.17862700	2.61715900	0.51042200
C	2.03060300	3.37920400	1.78194400
H	2.91246500	4.00054500	1.92893200
H	1.13996700	4.00321700	1.72496500
H	1.93789900	2.67122400	2.60363600
C	2.31207000	3.46550900	-0.70675400
H	2.42495100	2.81597200	-1.57296900
H	3.19335500	4.09671200	-0.60538200
H	1.42054800	4.08113200	-0.81495900
H	3.00748200	1.98440700	0.58156500
H	1.35691400	1.99213600	0.39463200

conf_22

C	4.82480800	-1.89709900	0.51858900
C	5.36785000	-2.43935400	-0.80984200
C	4.99394900	-1.64291700	-2.06836800
C	5.53125100	-0.20202400	-2.07671900
C	4.70853000	0.76118900	-1.26046100
O	3.49417300	0.72615000	-1.20175200
O	5.34353400	1.74190300	-0.59302400
H	6.30256300	1.67369700	-0.69375500
H	6.57855300	-0.17731000	-1.75842900
H	5.50484600	0.19506300	-3.09885400
H	5.40747300	-2.15524000	-2.93990100
H	3.91158400	-1.61307000	-2.21203300
H	6.46036100	-2.49869600	-0.74778100
H	5.01895500	-3.46864700	-0.94189700
C	3.30249800	-1.97460700	0.66816900
C	2.81720200	-1.34276100	1.97558800
C	1.30899000	-1.48948600	2.24363300
C	0.41312300	-0.81956200	1.24079200
C	-0.54247700	0.06992900	1.53914600
C	-1.53655700	0.63763300	0.56590900
C	-2.95783300	0.09821900	0.80881900
C	-3.99316200	0.70403400	-0.14037000
C	-5.40688000	0.16473700	0.08822300
C	-6.43945900	0.77109400	-0.86683000
C	-7.88086800	0.31543400	-0.60978600
C	-8.12853400	-1.17822300	-0.84490500
C	-9.59754000	-1.56742500	-0.66841700
H	-10.23631200	-1.02148400	-1.36910900
H	-9.75226900	-2.63572200	-0.83939900

H	-9.94608100	-1.33822100	0.34322200
H	-7.79775800	-1.44045200	-1.85753600
H	-7.51463100	-1.77131700	-0.15872700
H	-8.16414600	0.57160100	0.41920900
H	-8.55175400	0.88890000	-1.25958800
H	-6.16219400	0.52692700	-1.90036000
H	-6.39314300	1.86385800	-0.78836900
H	-5.39342400	-0.92503900	-0.01858100
H	-5.70976700	0.36802100	1.12318900
H	-3.99795000	1.79548800	-0.02458400
H	-3.69428500	0.50686600	-1.17775000
H	-2.94307600	-0.99134700	0.69946000
H	-3.24741600	0.30023100	1.84648300
H	-1.23282100	0.40420600	-0.46120800
H	-1.57188800	1.73182700	0.65340700
H	-0.68262000	0.34993200	2.58395700
H	0.51977700	-1.13305800	0.20218900
H	1.07604900	-1.11238700	3.24388600
H	1.06792300	-2.56015500	2.25438700
H	3.09097400	-0.27893600	1.98574600
H	3.35749900	-1.79457200	2.81396700
H	2.99421200	-3.02629800	0.63864000
H	2.82124100	-1.48580100	-0.18250700
H	5.15411800	-0.86002800	0.66968400
H	5.28783400	-2.46059800	1.33527100
N	1.77290500	2.06412600	0.43970000
C	2.44818200	2.74276400	1.58072700
H	1.70131600	3.24574600	2.19309500
H	2.97151300	1.99482500	2.17326200
H	3.16148600	3.46575900	1.18903500
C	0.99993200	2.98418800	-0.44237800
H	0.23736500	3.49226700	0.14493500
H	0.53283000	2.40041300	-1.23278200
H	1.68442700	3.71230200	-0.87489800
H	2.48839700	1.57404400	-0.14700700
H	1.15021500	1.31852300	0.80322600

conf_106

C	4.08780100	1.75214500	1.30778300
C	4.49676400	2.85089700	0.31826000
C	4.19821500	2.57268400	-1.16207300
C	4.94243000	1.35230000	-1.72939200
C	4.30373100	0.03225000	-1.38248700
O	3.10083500	-0.14360800	-1.34496500
O	5.10253400	-1.02373400	-1.14256500
H	6.03622700	-0.77680300	-1.18470400
H	4.93570600	1.39353900	-2.82546100
H	5.99330800	1.36242600	-1.42230300
H	3.12762000	2.43951400	-1.33276800
H	4.49848300	3.44460700	-1.74743400
H	3.99604000	3.78364300	0.59732100
H	5.57026400	3.04296100	0.42630800
C	2.57705500	1.53601700	1.43694100

C	2.23703500	0.37653200	2.37720100
C	0.73367400	0.17478700	2.63655300
C	-0.08180900	-0.17688200	1.42485700
C	-0.88242200	-1.24539200	1.32150600
C	-1.80927800	-1.52801600	0.17313300
C	-3.28621200	-1.33272500	0.56020100
C	-4.24017500	-1.64558200	-0.59464900
C	-5.72530000	-1.51917400	-0.22688700
C	-6.15990500	-0.13588700	0.27762800
C	-5.89418300	1.00860000	-0.70357000
C	-6.43757400	2.35539200	-0.21957500
C	-6.15373200	3.50092600	-1.19250700
H	-6.60441000	3.30751600	-2.17062400
H	-6.55492700	4.44817600	-0.82379000
H	-5.07745600	3.63215600	-1.34459700
H	-7.51816000	2.26950200	-0.05854000
H	-6.00239900	2.58804800	0.75989200
H	-4.81624800	1.10758700	-0.88340600
H	-6.34201400	0.76498700	-1.67567000
H	-5.67068600	0.08626100	1.23338900
H	-7.23239100	-0.17290500	0.49752700
H	-6.32203800	-1.77932400	-1.10816400
H	-5.96676800	-2.26794100	0.53640600
H	-4.01007900	-0.98646500	-1.43939200
H	-4.05244600	-2.66715700	-0.94651100
H	-3.52376800	-1.97182200	1.41882000
H	-3.42047200	-0.30095000	0.89724100
H	-1.68330400	-2.56274000	-0.17250300
H	-1.57057600	-0.87236900	-0.67252700
H	-0.94660000	-1.92969200	2.16838400
H	-0.05604500	0.52989600	0.59529700
H	0.33319700	1.10539200	3.05868400
H	0.59342200	-0.59460600	3.40161600
H	2.73026400	0.54029500	3.34094800
H	2.66888100	-0.55102500	1.97739600
H	2.14298900	1.35488000	0.45052200
H	2.11610400	2.45569200	1.81604500
H	4.49223700	2.00811700	2.29258000
H	4.57120400	0.80190000	1.04282400
N	1.66855500	-2.26989400	-0.40807800
C	2.48367200	-3.22616500	0.39172900
H	3.28386900	-3.61706500	-0.23423500
H	2.90849000	-2.69778100	1.24302800
H	1.84718200	-4.03866700	0.73885400
C	1.01244700	-2.88026900	-1.59931400
H	0.35908700	-3.68897500	-1.27728100
H	1.78331900	-3.26592400	-2.26471600
H	0.43220700	-2.11443200	-2.10970500
H	0.95331800	-1.83314100	0.20314200
H	2.27713500	-1.48395600	-0.73713500

conf_4

C	-3.85189400	-0.46740300	0.80101500

C	-4.89319600	0.03233500	-0.20187200
C	-4.87841400	1.54838400	-0.43575400
C	-3.66229300	2.06282400	-1.23219100
C	-2.32534300	2.02977200	-0.54056900
O	-1.29991400	1.63719000	-1.06761600
O	-2.24477600	2.51029500	0.71353900
H	-3.12024100	2.74307400	1.05441100
H	-3.54890700	1.50497800	-2.16212400
H	-3.82639000	3.11234000	-1.50828200
H	-5.76631500	1.83547300	-1.00384100
H	-4.97258600	2.07662600	0.52409000
H	-4.74811800	-0.47438300	-1.16331400
H	-5.89076600	-0.25445400	0.14436300
C	-3.81740000	-1.99204800	0.93546100
C	-2.84724400	-2.49276900	2.01317500
C	-1.38949000	-2.01771200	1.82555800
C	-0.76622500	-2.50314800	0.54662400
C	-0.61369100	-1.77594500	-0.56077400
C	-0.06125000	-2.26099700	-1.87041600
C	1.08187000	-1.38110900	-2.40395200
C	2.33606900	-1.41552200	-1.52842300
C	3.44523900	-0.47383500	-2.01093300
C	4.70552900	-0.47660300	-1.13713500
C	4.49714300	0.11214900	0.26051500
C	5.76095200	0.13897300	1.12299300
C	5.52108100	0.74602000	2.50596100
H	6.43465000	0.75350600	3.10395600
H	5.17263900	1.78177400	2.42922200
H	4.76931300	0.17707900	3.06301100
H	6.14096600	-0.88257700	1.22948600
H	6.54110600	0.70321100	0.60093900
H	4.12720000	1.14407000	0.15789100
H	3.72480500	-0.45477800	0.79519100
H	5.49447900	0.08859100	-1.64365100
H	5.07647500	-1.50355100	-1.04481300
H	3.71342900	-0.74879400	-3.03571700
H	3.06146700	0.55744000	-2.09090200
H	2.05891900	-1.20035700	-0.48818400
H	2.72865900	-2.43826400	-1.50048800
H	1.34200600	-1.70057100	-3.41768000
H	0.71809400	-0.34862500	-2.50195600
H	-0.86632500	-2.26683800	-2.61502400
H	0.27923000	-3.29657300	-1.77077100
H	-0.94262900	-0.73713000	-0.55623600
H	-0.46824000	-3.55077600	0.52722200
H	-0.80119500	-2.37341400	2.67827900
H	-1.36423800	-0.92399000	1.86362000
H	-2.86840100	-3.58688100	2.03341900
H	-3.19227400	-2.15946000	2.99817800
H	-3.54629700	-2.42585500	-0.03270800
H	-4.82222600	-2.35924800	1.16872400
H	-4.04541900	-0.01522600	1.78309300
H	-2.85837700	-0.12932400	0.49796900
N	1.17730200	1.47271000	0.09173100
C	1.68458400	2.87324900	0.00610400

H	2.66570000	2.92948600	0.47317500
H	1.74948400	3.15932600	-1.04213700
H	0.98247200	3.52353400	0.52478300
C	1.05589900	0.95490300	1.48698200
H	0.30849000	1.55098300	2.00737300
H	2.02178800	1.03571100	1.98140800
H	0.73981100	-0.08498700	1.43950300
H	0.23313000	1.44981200	-0.36196100
H	1.78948600	0.85110000	-0.44324100

conf_64

C	4.15673600	0.65821400	0.84505500
C	3.47502600	-0.65391200	0.43538700
C	3.59375400	-0.97892100	-1.05715200
C	2.75599400	-2.19564900	-1.47223100
C	1.26688500	-2.01876900	-1.32865600
O	0.50508500	-2.90219500	-0.98088100
O	0.73207500	-0.82232800	-1.63993900
H	1.41928600	-0.16939700	-1.83862900
H	2.93165300	-2.44101700	-2.52727800
H	3.02413500	-3.08115900	-0.89469100
H	3.32444000	-0.10621300	-1.66782000
H	4.63743000	-1.17913700	-1.31317000
H	3.90810600	-1.48101400	1.00692600
H	2.41964100	-0.61827000	0.72366400
C	3.57965200	1.91686800	0.18033000
C	2.08321900	2.12499300	0.42647100
C	1.52078800	3.36951400	-0.27785300
C	0.05400500	3.55258300	-0.02327900
C	-0.90562600	3.45232600	-0.94194700
C	-2.37434800	3.61015300	-0.67695100
C	-3.18796600	2.34126700	-0.99691200
C	-2.73750900	1.09133600	-0.22806600
C	-2.81596900	1.22188600	1.29555600
C	-2.46513200	-0.06326400	2.05433900
C	-0.98880000	-0.47379600	1.98526800
C	-0.67878800	-1.72558500	2.81145400
C	0.78155700	-2.16971200	2.72986600
H	0.97075100	-3.03125500	3.37400800
H	1.05283900	-2.45482600	1.70982800
H	1.45353000	-1.36658500	3.04535100
H	-0.94725400	-1.53325100	3.85542600
H	-1.32874000	-2.55008200	2.48963600
H	-0.66637900	-0.62868500	0.94698000
H	-0.37507700	0.35929600	2.34637500
H	-3.10409300	-0.88719200	1.69631500
H	-2.73491900	0.05967100	3.10793900
H	-2.14608900	2.01949900	1.62880600
H	-3.82900700	1.53526500	1.56945600
H	-3.39065400	0.26140700	-0.53993900
H	-1.71241300	0.84202500	-0.52755500
H	-4.24369600	2.53642300	-0.78130300
H	-3.12493500	2.13942300	-2.07247300

H	-2.77085400	4.42750800	-1.28968500
H	-2.53008100	3.90496700	0.36435700
H	-0.62355500	3.24104200	-1.97461000
H	-0.22781100	3.77214500	1.00648900
H	1.70183200	3.29464700	-1.35621800
H	2.07088400	4.25241300	0.06897000
H	1.89830700	2.19795500	1.50515400
H	1.50841200	1.25466600	0.08938100
H	3.77064200	1.89583000	-0.89957500
H	4.12628900	2.78898400	0.55257400
H	5.22738700	0.59392500	0.62314100
H	4.07745700	0.76099300	1.93234600
N	-2.14846600	-2.29185900	-0.83707400
C	-2.55302900	-1.98390600	-2.23960300
H	-2.38588400	-2.86959800	-2.85009400
H	-3.60566800	-1.70756300	-2.26062600
H	-1.93977500	-1.16065400	-2.59979300
C	-2.90569500	-3.41341500	-0.20973200
H	-2.74402000	-4.31429500	-0.79888100
H	-3.96572900	-3.16541700	-0.18776400
H	-2.53438700	-3.56164100	0.80213800
H	-1.12117400	-2.51861500	-0.83688600
H	-2.25738000	-1.44629900	-0.27024300

conf_53

C	-4.32318400	0.78033000	-0.19533700
C	-4.75097100	-0.56865600	0.39106400
C	-4.92128600	-1.68164000	-0.65287300
C	-3.69672000	-1.89545100	-1.56888000
C	-2.39288900	-2.06099900	-0.83376200
O	-1.38729700	-1.42322900	-1.08862400
O	-2.33534700	-2.96326000	0.15867400
H	-3.19262500	-3.39135600	0.29414300
H	-3.84547700	-2.79299100	-2.18071400
H	-3.56460700	-1.05948800	-2.25323300
H	-5.18588900	-2.61956700	-0.14824700
H	-5.76868700	-1.45687400	-1.30599900
H	-4.02367400	-0.89042300	1.14489800
H	-5.69842500	-0.45261700	0.92673100
C	-4.22048700	1.91338700	0.83654400
C	-3.33676900	1.61060600	2.05558400
C	-1.91319800	1.12142900	1.71964300
C	-1.09021200	2.12247500	0.95978900
C	-0.73053400	2.00970800	-0.31995100
C	0.05880700	3.01841900	-1.10469100
C	1.35928700	2.42705600	-1.67826000
C	2.39155100	2.07894700	-0.60243600
C	3.54750400	1.21166000	-1.10809900
C	4.45364700	0.68813800	0.00789700
C	5.57641000	-0.22243200	-0.49627900
C	6.44220100	-0.83076900	0.61417500
C	5.69713800	-1.82207000	1.51314900
H	4.88996500	-1.34108700	2.07411800

H	5.26523500	-2.63849800	0.92302800
H	6.37005200	-2.27272000	2.24580500
H	7.29401200	-1.34080400	0.15472300
H	6.86389000	-0.02605400	1.22667400
H	5.14726300	-1.03332400	-1.10151200
H	6.21299900	0.35146200	-1.17765600
H	3.84106800	0.15214200	0.74559500
H	4.88866900	1.53321000	0.55341800
H	3.14833800	0.36061900	-1.68001000
H	4.14187200	1.78077000	-1.83036500
H	2.78869400	3.00079700	-0.16433600
H	1.88672300	1.58154700	0.24065700
H	1.11047000	1.52810100	-2.25974000
H	1.80043400	3.12871300	-2.39187000
H	0.28632500	3.88665700	-0.47814400
H	-0.55460000	3.38243700	-1.93673000
H	-1.03952800	1.12137500	-0.87278900
H	-0.80920200	3.02308400	1.50504500
H	-1.96962800	0.18846700	1.15088900
H	-1.41597300	0.88473100	2.66891900
H	-3.81634200	0.85604700	2.68705200
H	-3.26708300	2.51368400	2.67024500
H	-5.22285900	2.16842100	1.19608100
H	-3.84161700	2.80441500	0.32724000
H	-5.03697700	1.07762700	-0.97177600
H	-3.35863700	0.67582800	-0.70009000
N	1.07529600	-1.08464200	0.04263200
C	1.03109600	-1.14179900	1.53325000
H	2.04343100	-1.05272300	1.92295100
H	0.41466900	-0.32064300	1.89034200
H	0.59574500	-2.09447600	1.82951900
C	1.96546500	-2.11000900	-0.57687100
H	2.98199700	-1.96741100	-0.21669900
H	1.92966100	-1.99739300	-1.65861000
H	1.60089400	-3.09648400	-0.29555500
H	0.10753300	-1.21378400	-0.33504000
H	1.37483500	-0.14700800	-0.23972500

conf_201

C	-0.98702900	-2.14424100	-0.84444800
C	-1.69935900	-3.19613400	0.01434200
C	-3.17090400	-3.43027800	-0.36122300
C	-4.16555900	-2.43560200	0.27913800
C	-3.96287000	-0.99470200	-0.10268200
O	-3.69739400	-0.11323600	0.69347300
O	-4.08789900	-0.66048400	-1.39922000
H	-4.27080700	-1.43603000	-1.94760400
H	-5.18888100	-2.71134000	-0.00111500
H	-4.09789600	-2.48617700	1.36564300
H	-3.27911000	-3.43839100	-1.45339000
H	-3.48196400	-4.42337500	-0.02952000
H	-1.15923000	-4.14231300	-0.08665700
H	-1.64170900	-2.92857700	1.07489500

C	0.47776600	-1.90501100	-0.45547800
C	0.69650500	-1.32175900	0.94881100
C	-0.11562700	-0.04565700	1.25655000
C	0.11189800	1.05362200	0.25818000
C	0.62445700	2.25997200	0.52945300
C	0.95574700	3.30251900	-0.49751100
C	2.47626600	3.48319700	-0.71593200
C	3.19401100	2.19332300	-1.14897400
C	3.76304600	1.36922700	0.01199700
C	4.23776800	-0.02137400	-0.41098300
C	4.84495200	-0.82528900	0.74204000
C	5.30894600	-2.23558300	0.35650100
C	4.17124600	-3.18003200	-0.04252300
H	3.43602100	-3.26731200	0.76450400
H	3.64764100	-2.83172700	-0.93668600
H	4.54925500	-4.18290100	-0.25544000
H	6.03126400	-2.16528100	-0.46500700
H	5.85194700	-2.67038800	1.20169200
H	5.69575000	-0.26766700	1.14920400
H	4.11403100	-0.89840300	1.55891800
H	4.97805600	0.07118600	-1.21526500
H	3.38960500	-0.56541400	-0.84267500
H	4.59456700	1.92129200	0.46586600
H	3.00933300	1.26088300	0.79859600
H	2.50074600	1.57220900	-1.73096100
H	4.01102300	2.44399200	-1.83217300
H	2.60314400	4.26028100	-1.47463700
H	2.93343400	3.87306500	0.20005000
H	0.53425000	4.27046500	-0.19857400
H	0.49769000	3.02941000	-1.45527600
H	0.89410900	2.49179300	1.55999400
H	-0.10782000	0.81881000	-0.78362300
H	0.14600300	0.31093200	2.25719600
H	-1.18071200	-0.30508900	1.28807600
H	1.76005600	-1.10271000	1.06985300
H	0.45126200	-2.07030500	1.70810700
H	0.93238400	-1.24110100	-1.19762300
H	1.02874300	-2.84751900	-0.52763400
H	-1.52703600	-1.19287800	-0.81050800
H	-1.02282400	-2.46872900	-1.89057000
N	-2.68707000	2.41236000	0.39063500
C	-3.01385200	3.03593500	-0.92191500
H	-2.70475200	2.35801600	-1.71556000
H	-4.08859400	3.20044600	-0.97953300
H	-2.48474000	3.98323400	-1.01210000
C	-3.07309100	3.22663800	1.57664400
H	-2.55478100	4.18373100	1.53997500
H	-2.79173700	2.68455700	2.47778300
H	-4.15007600	3.38582800	1.56374600
H	-3.14485800	1.47393100	0.44941900
H	-1.66684100	2.22018700	0.42821200

conf_357

C	-0.24532400	2.52243600	1.60286200
C	0.99417600	3.04768000	0.86103300
C	1.50839600	2.15637500	-0.28010100
C	0.78427200	2.35047800	-1.63210100
C	-0.64343400	1.87509100	-1.62189100
O	-0.95679700	0.70099000	-1.54734500
O	-1.63048300	2.78616900	-1.69283700
H	-1.27703700	3.68417500	-1.74554500
H	0.84145400	3.40003400	-1.93535500
H	1.29533500	1.75726700	-2.39451200
H	2.56219200	2.37404500	-0.46612500
H	1.46188400	1.10149500	-0.00403700
H	0.79516200	4.05241500	0.46885400
H	1.80058700	3.17353300	1.58996900
C	0.05582700	1.32109000	2.50597200
C	-1.16578500	0.76994400	3.25363900
C	-2.28721800	0.21228600	2.35051300
C	-1.79311800	-0.86566900	1.42814300
C	-2.12919500	-2.15906800	1.49333900
C	-1.57721700	-3.27392400	0.64093000
C	-0.49855500	-2.88207400	-0.37569000
C	0.86000600	-2.52878200	0.24446300
C	1.80127600	-1.84690400	-0.75076400
C	3.09861200	-1.34327100	-0.11644200
C	4.00549800	-0.61047500	-1.10919700
C	5.21642700	0.07880500	-0.46849900
C	6.19988000	-0.88421000	0.20169000
H	6.55413500	-1.63754800	-0.50866200
H	7.07394000	-0.35072400	0.58245300
H	5.74430900	-1.40996100	1.04452000
H	5.74395400	0.65112600	-1.23882900
H	4.86356700	0.81127900	0.26896700
H	4.35147300	-1.31727800	-1.87322900
H	3.41018800	0.14148100	-1.64313300
H	3.63562800	-2.18606300	0.33007700
H	2.85549800	-0.66811400	0.71550500
H	2.03693000	-2.53736100	-1.56962500
H	1.27318900	-1.00230300	-1.21123700
H	0.71633700	-1.87143000	1.10740600
H	1.32544500	-3.43952200	0.63691500
H	-0.36406500	-3.70763700	-1.08154700
H	-0.83833200	-2.02868700	-0.97512200
H	-2.41477700	-3.76837200	0.12969200
H	-1.18101900	-4.04436900	1.31470300
H	-2.85002600	-2.46600800	2.25087200
H	-1.05276500	-0.55447000	0.69698300
H	-2.71126800	1.03997800	1.76702200
H	-3.09477400	-0.17292200	2.97943800
H	-1.58961500	1.55637300	3.88710000
H	-0.83798600	-0.02953600	3.92468700
H	0.80454700	1.62327300	3.24544000
H	0.52208300	0.51921100	1.92454000
H	-1.03565300	2.26801900	0.88908800
H	-0.65537800	3.32971200	2.21924900
N	-3.40166900	-0.48566700	-1.16353100

C	-4.51678300	0.48290700	-0.96684800
H	-5.40563900	-0.05274600	-0.63728500
H	-4.21815900	1.20980300	-0.21426000
H	-4.71440100	0.99057200	-1.90936400
C	-3.67154100	-1.53705000	-2.18485900
H	-2.79879200	-2.18263500	-2.25847000
H	-3.86044000	-1.05791400	-3.14419700
H	-4.54203800	-2.11704200	-1.88217100
H	-2.52998500	0.03591000	-1.41424400
H	-3.16295300	-0.92864400	-0.25751000

conf_214

C	-2.93195500	1.11019500	1.47463300
C	-1.44687300	1.40273500	1.69673900
C	-1.10905900	2.88851200	1.54142500
C	0.39596700	3.18822700	1.58626200
C	1.19530300	2.56665300	0.47119900
O	2.31245200	2.10307500	0.61019500
O	0.65430300	2.54306700	-0.75987500
H	-0.24595400	2.89965100	-0.75070000
H	0.57305000	4.26940800	1.52625000
H	0.84178400	2.84765500	2.52148800
H	-1.55380900	3.27776700	0.61282900
H	-1.59140900	3.46334400	2.33596600
H	-1.14084400	1.06976800	2.69501900
H	-0.86141000	0.80647400	0.98460300
C	-3.29209400	-0.36124400	1.69066100
C	-4.73421800	-0.71815500	1.30926900
C	-5.08638500	-0.45153600	-0.16978600
C	-4.15464500	-1.13474900	-1.12811000
C	-3.35279200	-0.51586300	-1.99382200
C	-2.38780800	-1.18682100	-2.92953200
C	-0.91597700	-0.93937000	-2.54239300
C	-0.52035900	-1.63699100	-1.24051500
C	0.89251400	-1.30250200	-0.76217500
C	1.27784200	-1.99829200	0.54540600
C	2.68317500	-1.64499200	1.04153600
C	3.08677500	-2.32483900	2.35759000
C	3.18846900	-3.84916000	2.26808500
H	3.89224400	-4.15212400	1.48613300
H	3.54191600	-4.27087500	3.21136300
H	2.22388900	-4.31134100	2.04610200
H	4.05019200	-1.91795400	2.68212700
H	2.36373000	-2.04708000	3.13286100
H	3.41980200	-1.92618700	0.27001000
H	2.74850000	-0.55810000	1.18076300
H	1.19352200	-3.07979700	0.40654200
H	0.55006900	-1.73127500	1.32060400
H	1.60760400	-1.58713500	-1.55153400
H	0.96632100	-0.21439600	-0.63542000
H	-1.23209100	-1.36346800	-0.45657500
H	-0.61242400	-2.72146000	-1.36904500
H	-0.26684600	-1.28340800	-3.35625200

H	-0.74771200	0.14215400	-2.44494900
H	-2.55166100	-0.81915100	-3.94842200
H	-2.57859200	-2.26477500	-2.95006500
H	-3.37300600	0.57424600	-2.03505000
H	-4.13578100	-2.22411900	-1.08523800
H	-5.09402000	0.62374900	-0.37127400
H	-6.11007000	-0.80162600	-0.34322600
H	-5.43383400	-0.16120100	1.94180700
H	-4.90018400	-1.77803500	1.52913000
H	-3.12425100	-0.62237100	2.74125700
H	-2.60696900	-0.98445300	1.10640300
H	-3.19248100	1.40819200	0.45390400
H	-3.53588200	1.73731400	2.14135600
N	3.59162400	0.65399800	-1.30692200
C	5.05799000	0.71404000	-1.03942900
H	5.39156800	1.74343100	-1.15732600
H	5.23816500	0.38186500	-0.01869400
H	5.58150200	0.06894900	-1.74335400
C	3.18918400	1.07279900	-2.68090500
H	3.67038400	0.42468900	-3.41160700
H	3.50018900	2.10455000	-2.83450500
H	2.10671200	0.99861600	-2.76068100
H	3.25251800	-0.29492600	-1.12991000
H	3.08985400	1.25443800	-0.60362500

conf_76

C	-2.98882400	1.67738400	0.47727300
C	-1.71060400	2.35271700	0.98482400
C	-1.17622200	3.44280500	0.04472100
C	-0.57453800	2.91681400	-1.26637700
C	0.69253900	2.11447500	-1.12675500
O	1.01131900	1.20953900	-1.87616200
O	1.55012900	2.43994300	-0.14158200
H	1.18670000	3.14215900	0.41767800
H	-1.28140300	2.29560300	-1.81632100
H	-0.32119800	3.75449400	-1.92856200
H	-1.98933100	4.12623300	-0.21436500
H	-0.44729100	4.07715000	0.57059000
H	-1.91587500	2.80934600	1.95803600
H	-0.93854800	1.59266000	1.16578600
C	-3.57533300	0.68596700	1.48615800
C	-4.96503300	0.15881200	1.10781100
C	-5.03166800	-0.57171700	-0.24717700
C	-4.09791100	-1.74319000	-0.32502600
C	-3.12696000	-1.89515500	-1.22378900
C	-2.16756200	-3.04828000	-1.28596900
C	-0.71500700	-2.61414500	-1.01561300
C	-0.48466600	-2.13397000	0.41895400
C	0.90568300	-1.53525400	0.62556500
C	1.17804900	-1.04449100	2.04977300
C	2.51904700	-0.31692600	2.21181000
C	3.75841800	-1.19616100	2.00227800
C	5.07222300	-0.42840700	2.16127600

H	5.13828600	0.40995400	1.45857200
H	5.15657300	-0.00773100	3.16626200
H	5.93916900	-1.07278100	1.99865400
H	3.72902400	-2.02661200	2.71509400
H	3.73276500	-1.68562500	1.01658300
H	2.54805400	0.54249500	1.52553400
H	2.57693800	0.11467300	3.21640400
H	1.13564800	-1.89223400	2.74254600
H	0.36952600	-0.36841000	2.34895300
H	1.65748000	-2.28168600	0.33814600
H	1.00682000	-0.68920600	-0.06582600
H	-1.23956000	-1.38383800	0.67397600
H	-0.63538700	-2.96864300	1.11311700
H	-0.04190700	-3.45195500	-1.23360700
H	-0.45152900	-1.80828100	-1.71434500
H	-2.21690100	-3.51322100	-2.27712200
H	-2.46125600	-3.81628900	-0.56291200
H	-2.98859400	-1.12179100	-1.98085200
H	-4.23606900	-2.51623100	0.43148100
H	-4.81969900	0.12231200	-1.06655700
H	-6.06173000	-0.91586000	-0.39587400
H	-5.67653500	0.99173800	1.09276700
H	-5.30714900	-0.52120000	1.89526600
H	-3.64252600	1.17213900	2.46589500
H	-2.88510100	-0.15622500	1.60711600
H	-2.79297300	1.16081400	-0.46769800
H	-3.73366600	2.45278200	0.25852000
N	3.32654400	-0.15668400	-1.38108800
C	3.34910100	-1.35635200	-2.26871300
H	4.24650500	-1.93870600	-2.06564100
H	2.45919700	-1.95023100	-2.06992000
H	3.34641200	-1.02111600	-3.30422100
C	4.48712500	0.76431400	-1.55354400
H	4.35549900	1.61300300	-0.88517000
H	4.50883800	1.10577100	-2.58700800
H	5.40783800	0.23499000	-1.31465100
H	2.43795500	0.37868300	-1.55558800
H	3.28044600	-0.46363500	-0.40605500

conf_111

C	-5.27722400	-1.09191300	-1.38104300
C	-5.04078300	-2.02622600	-0.17964000
C	-3.63748200	-1.94223700	0.43217400
C	-2.50948300	-2.23193900	-0.57804500
C	-1.15326200	-1.85071100	-0.06496900
O	-0.20212100	-2.61224500	0.00799500
O	-0.95917700	-0.58339800	0.33727200
H	-1.73643000	-0.00662600	0.21963400
H	-2.47795700	-3.28674100	-0.84884900
H	-2.67489500	-1.66043500	-1.49770000
H	-3.50286900	-0.94603000	0.86330100
H	-3.55534200	-2.64598900	1.26504200
H	-5.75787400	-1.79835400	0.61397900

H	-5.24923900	-3.05684100	-0.48353000
C	-4.74338100	0.33669600	-1.21248800
C	-5.28381400	1.10224200	-0.00260400
C	-4.50425600	2.40197600	0.26204300
C	-3.10308200	2.18748100	0.76692800
C	-1.99616800	2.67935000	0.20139300
C	-0.60984700	2.60884700	0.77715400
C	0.46970100	2.24563400	-0.25266500
C	1.84261000	2.02787300	0.38462500
C	2.89992400	1.51807000	-0.59853800
C	4.25325300	1.21111500	0.04878200
C	5.27401900	0.60421600	-0.91554500
C	6.59113300	0.21107600	-0.24236000
C	7.60222800	-0.39852500	-1.21418500
H	8.52958800	-0.67034500	-0.70537500
H	7.20408000	-1.30185100	-1.68694700
H	7.85490600	0.30659600	-2.01081200
H	7.02691300	1.09414000	0.23768300
H	6.38690800	-0.50022500	0.56951500
H	4.83986400	-0.27824000	-1.40571400
H	5.47626600	1.31823700	-1.72177000
H	4.11542000	0.53256600	0.90519100
H	4.66043400	2.12990400	0.48398500
H	2.52156900	0.62149600	-1.11812400
H	3.03969600	2.25348900	-1.39749600
H	2.19350500	2.95805400	0.84443200
H	1.73601300	1.31955300	1.22025500
H	0.17405700	1.33755600	-0.78770700
H	0.53655400	3.03710100	-1.00694800
H	-0.59068700	1.89589300	1.60936300
H	-0.36080600	3.58602600	1.21096400
H	-2.09683700	3.23876200	-0.72851500
H	-3.01528300	1.65802600	1.71845300
H	-5.04670400	2.98967700	1.01176800
H	-4.47641700	3.00950100	-0.64843100
H	-5.24991700	0.48063500	0.89871100
H	-6.33967600	1.33968900	-0.16342800
H	-4.96939800	0.90618900	-2.12000100
H	-3.64784300	0.32017000	-1.15773000
H	-6.35256500	-1.05915200	-1.58140500
H	-4.82770100	-1.52689200	-2.28052800
N	2.18231800	-1.58226300	0.78175000
C	2.15868400	-1.49652300	2.27071200
H	1.31949000	-0.87050500	2.56775100
H	2.03290100	-2.50020300	2.67298600
H	3.09376600	-1.06509800	2.62372000
C	3.28043400	-2.43196700	0.23478300
H	3.15881800	-3.44344800	0.61797100
H	4.24102400	-2.02332800	0.54303800
H	3.20937700	-2.43910400	-0.85115800
H	1.25100600	-1.96106000	0.45705600
H	2.26122200	-0.63901600	0.39390600

conf_260

C	-4.50972800	-0.96559100	1.04513700
C	-4.84538000	-2.38564100	0.57079800
C	-3.76294700	-3.11404300	-0.23738500
C	-3.43644500	-2.45941400	-1.59860100
C	-2.52801200	-1.26112400	-1.55067300
O	-2.77108400	-0.18559500	-2.06397300
O	-1.34327300	-1.40658000	-0.92417500
H	-1.25259300	-2.29307700	-0.54769000
H	-2.93503200	-3.19214400	-2.24303200
H	-4.34954200	-2.15390400	-2.10953900
H	-2.85489700	-3.23907900	0.36581300
H	-4.11066600	-4.12844200	-0.44490600
H	-5.08958600	-3.00254400	1.44172100
H	-5.75474800	-2.34916100	-0.03856800
C	-3.39138200	-0.88801500	2.09159700
C	-3.19053400	0.52024200	2.67142100
C	-2.65215200	1.56379500	1.67162500
C	-1.22399100	1.31054800	1.27627300
C	-0.23097300	2.20344900	1.36908100
C	1.21663900	1.97203300	1.02722100
C	1.57686500	0.57041400	0.52979900
C	3.02691300	0.43739200	0.04741900
C	4.07789300	0.64184200	1.14393800
C	5.52428900	0.46854600	0.66247100
C	5.88211800	-0.95083100	0.21211300
C	7.35958300	-1.10923700	-0.15694000
C	7.71415800	-2.52572700	-0.61197300
H	8.77354600	-2.61025800	-0.86577800
H	7.50156900	-3.25684600	0.17399500
H	7.13679200	-2.81251200	-1.49655200
H	7.61098900	-0.39461400	-0.94959100
H	7.97662000	-0.83529300	0.70649900
H	5.63275400	-1.65716900	1.01486300
H	5.27113800	-1.24053500	-0.65098800
H	6.19844200	0.75914400	1.47550300
H	5.72009500	1.17112300	-0.15767400
H	3.97210400	1.64431500	1.57131500
H	3.88182600	-0.06302100	1.96221500
H	3.15136000	-0.55578700	-0.39351000
H	3.20992100	1.15612200	-0.76318700
H	1.39285800	-0.15360600	1.33200600
H	0.90492900	0.28180700	-0.28729700
H	1.80852900	2.21416000	1.91670400
H	1.53150600	2.71938700	0.28320900
H	-0.46602800	3.19644000	1.75276600
H	-0.99760100	0.31109300	0.91615100
H	-2.73822600	2.56386600	2.10589500
H	-3.29515000	1.55563100	0.78088500
H	-2.50606700	0.46986800	3.52348000
H	-4.14812100	0.87769400	3.06371200
H	-2.44628800	-1.25470600	1.67613200
H	-3.63671000	-1.56745900	2.91462600
H	-5.41780400	-0.53679900	1.48233400
H	-4.27772300	-0.33155900	0.18318500

N	-1.28884000	2.10639000	-1.74695600
C	-0.06066800	2.12878900	-2.58981400
H	0.54878400	1.26189400	-2.34248100
H	-0.35042200	2.09269300	-3.63880700
H	0.49633900	3.04265200	-2.38954600
C	-2.21495000	3.25040900	-1.97984900
H	-1.69589000	4.18324800	-1.76466300
H	-3.07425300	3.14265800	-1.32062900
H	-2.54226400	3.23649900	-3.01805700
H	-1.80525900	1.20946300	-1.90523300
H	-1.02627500	2.08441600	-0.74336000

conf_140

C	-3.15662200	1.21153900	0.71975300
C	-4.62887200	0.86394100	0.95617400
C	-5.26278100	-0.03726200	-0.11059500
C	-4.66872500	-1.45890500	-0.18876900
C	-3.26915200	-1.55450000	-0.73249700
O	-2.37004900	-2.18457800	-0.20812800
O	-2.99992200	-0.92301800	-1.89293700
H	-3.76738000	-0.42258200	-2.20428200
H	-4.66058500	-1.92939200	0.79467400
H	-5.29510400	-2.08167300	-0.83938600
H	-6.32849100	-0.14982300	0.10137700
H	-5.21797100	0.45880100	-1.09064100
H	-4.73564700	0.38285400	1.93525800
H	-5.21036300	1.78984000	1.00809700
C	-2.58659500	2.17425800	1.76572700
C	-1.07826600	2.40687600	1.62287800
C	-0.67263300	3.16440600	0.34797900
C	0.81000200	3.37758500	0.24746200
C	1.56608200	3.08274500	-0.80936400
C	3.04535100	3.31733200	-0.91853000
C	3.85055700	2.02940800	-1.16581800
C	3.79584400	1.04020900	-0.00039300
C	4.58623300	-0.24536900	-0.26380600
C	4.42613900	-1.31798400	0.82115700
C	3.04109400	-1.97113900	0.83107100
C	2.85253000	-3.06646200	1.88178600
C	1.46149500	-3.70157600	1.82761100
H	1.33932900	-4.47719700	2.58604800
H	0.67448800	-2.95823000	2.00349700
H	1.27930600	-4.17852100	0.85639500
H	3.61521300	-3.83869800	1.73980800
H	3.02448000	-2.64317800	2.87647700
H	2.28095400	-1.19353500	0.99358800
H	2.86978000	-2.40346100	-0.16854000
H	4.62623200	-0.88013300	1.80565400
H	5.17751300	-2.10044500	0.67389900
H	4.28657400	-0.66945100	-1.23289200
H	5.64611000	0.00686000	-0.36810400
H	4.18767600	1.52333800	0.90263900
H	2.74893200	0.81033800	0.22301600

H	3.48083600	1.54280300	-2.07908200
H	4.89303800	2.29411500	-1.36995600
H	3.40722800	3.80800300	-0.00922800
H	3.23622400	4.01101100	-1.74562700
H	1.08784900	2.65221900	-1.69181200
H	1.28604200	3.82458600	1.11983700
H	-1.18141400	4.13741600	0.35450500
H	-1.02603700	2.64202000	-0.54754500
H	-0.55738500	1.44005200	1.65786800
H	-0.71777200	2.96833100	2.49060300
H	-2.79108600	1.77115400	2.76342300
H	-3.11362600	3.13359400	1.70824700
H	-3.04363800	1.64071700	-0.28253400
H	-2.54923000	0.29981300	0.74351000
N	0.20619900	-1.77840900	-1.00246500
C	0.36046800	-0.30928000	-1.22208100
H	-0.36418500	0.00163900	-1.97148900
H	0.17140800	0.21297700	-0.28691300
H	1.37396900	-0.10050700	-1.55692600
C	0.47835400	-2.60278600	-2.21519700
H	1.49727400	-2.41993000	-2.55247300
H	-0.23106100	-2.31907900	-2.99046200
H	0.34944400	-3.65361600	-1.96196700
H	0.83824500	-2.06811400	-0.25133700
H	-0.77381500	-1.97351100	-0.67873700

conf_226

C	1.60658900	2.72621400	0.43351000
C	0.99678300	2.69146100	-0.97228200
C	0.04853800	3.85526700	-1.28605100
C	-1.14111700	3.99307500	-0.30339700
C	-1.85161600	2.68142700	-0.11349600
O	-1.81727600	2.03901700	0.92097000
O	-2.51850300	2.16955600	-1.15754900
H	-2.47253400	2.75355800	-1.92703700
H	-0.80008900	4.31902200	0.67728500
H	-1.84829800	4.74023500	-0.67751800
H	0.59482500	4.80189100	-1.26273300
H	-0.32640100	3.74989200	-2.31018300
H	1.79123100	2.69568200	-1.72281700
H	0.46809500	1.73817700	-1.10863500
C	2.57066700	1.56371200	0.70330000
C	3.91305100	1.67372800	-0.02416300
C	4.82986400	0.46181900	0.22246400
C	4.33903800	-0.79228700	-0.44107700
C	3.95568700	-1.90436300	0.18437500
C	3.49290800	-3.16432500	-0.48905900
C	2.02706600	-3.52069600	-0.18477500
C	1.02309100	-2.54112300	-0.79497800
C	-0.43067200	-2.90993700	-0.49822900
C	-1.45158300	-1.94810800	-1.11123000
C	-2.90551000	-2.30477600	-0.77969700
C	-3.92493600	-1.24998300	-1.21574800

C	-5.36183200	-1.61470600	-0.84059800
H	-5.66394900	-2.55751900	-1.30419600
H	-5.47384300	-1.73352600	0.24266400
H	-6.06634000	-0.84519900	-1.16351300
H	-3.66101700	-0.27756900	-0.78205400
H	-3.84966800	-1.11448300	-2.30003100
H	-3.15037700	-3.26796700	-1.23965100
H	-3.02043900	-2.49632500	0.30246300
H	-1.32995900	-1.93388300	-2.19909500
H	-1.23038700	-0.91682100	-0.79593300
H	-0.63284300	-3.92383200	-0.86070100
H	-0.57318300	-2.96382900	0.59229300
H	1.23454900	-1.52720300	-0.43310200
H	1.17032600	-2.50468000	-1.88097600
H	1.81987300	-4.52770200	-0.56219500
H	1.88350400	-3.56624500	0.90277200
H	4.12647600	-3.99678400	-0.16267600
H	3.62814500	-3.07526400	-1.57221800
H	3.98800000	-1.92957300	1.27450000
H	4.31084400	-0.77168800	-1.53105200
H	4.93801800	0.29575900	1.29991100
H	5.82934400	0.69837400	-0.15940100
H	4.42282500	2.58454200	0.30775400
H	3.75457700	1.78376500	-1.10266700
H	2.08866300	0.61808600	0.42442400
H	2.76645000	1.50262900	1.78018000
H	0.80522100	2.69947800	1.17822100
H	2.13092600	3.67820600	0.58096100
N	-1.86730400	-0.47478600	1.86886200
C	-0.47031200	-0.56855700	2.38825100
H	-0.30586500	0.25211100	3.08357800
H	-0.33603500	-1.52430600	2.89165700
H	0.22023000	-0.48612100	1.55156100
C	-2.92543000	-0.58457500	2.91505900
H	-2.79859300	0.23088300	3.62465100
H	-2.83167600	-1.54278700	3.42366000
H	-3.89928100	-0.50899100	2.43437700
H	-1.96881800	0.44593000	1.37946600
H	-2.01682800	-1.20061400	1.16353600

conf_42

C	1.89230000	-2.88352300	-1.11033300
C	0.62121100	-3.45617900	-1.75013900
C	-0.71645600	-2.89467700	-1.24845700
C	-0.93601700	-3.05202500	0.28102800
C	-0.38791200	-1.89606500	1.06441500
O	0.56986400	-1.93533800	1.81426500
O	-0.98994900	-0.70028800	0.89847600
H	-1.75517600	-0.74727400	0.30583800
H	-2.00724800	-3.11556600	0.49367800
H	-0.46669300	-3.96350400	0.65004800
H	-0.82133800	-1.84332900	-1.53727700
H	-1.52514300	-3.42474300	-1.75599700

H	0.66289000	-3.30691200	-2.83438900
H	0.61411400	-4.54013600	-1.59441900
C	2.13251500	-1.38945200	-1.35335500
C	3.47172000	-0.92260600	-0.77335300
C	3.86745600	0.51606800	-1.15893800
C	3.03736900	1.60699200	-0.53393100
C	2.04885600	2.25890400	-1.15108800
C	1.25613800	3.42350600	-0.62377500
C	-0.17731400	3.46502500	-1.17273000
C	-1.04207600	2.26799700	-0.77153200
C	-2.37101600	2.22301800	-1.53174700
C	-3.28717900	1.05046900	-1.16285100
C	-3.89967800	1.14021900	0.24125000
C	-4.95546400	0.06523900	0.53336000
C	-4.39895800	-1.35936600	0.60553000
H	-3.93017000	-1.66159600	-0.33740100
H	-3.66474800	-1.44925100	1.41428000
H	-5.18926500	-2.08459300	0.81072200
H	-5.44884000	0.29954600	1.48127900
H	-5.73373800	0.11218100	-0.23579400
H	-3.11758800	1.09835600	1.01030900
H	-4.36417900	2.12662700	0.34461000
H	-2.73238300	0.10917600	-1.29753800
H	-4.10529300	1.00000100	-1.88928300
H	-2.91406500	3.16170400	-1.37081400
H	-2.15284900	2.17966400	-2.60423200
H	-0.49070600	1.33793900	-0.95942200
H	-1.22798300	2.30488300	0.30822100
H	-0.66246900	4.39118900	-0.85036500
H	-0.12291300	3.51659300	-2.26649900
H	1.76523700	4.34745900	-0.92406200
H	1.25449600	3.42841500	0.47347600
H	1.81552100	1.96722800	-2.17526000
H	3.34022200	1.93794800	0.46254700
H	3.81347400	0.60691700	-2.24821500
H	4.91605000	0.67565200	-0.88866200
H	4.25886100	-1.59522900	-1.12860000
H	3.46059000	-1.03851600	0.31865500
H	1.31587700	-0.78628100	-0.93900400
H	2.12574800	-1.19491700	-2.43280400
H	2.74449200	-3.43735900	-1.51734900
H	1.89673300	-3.08607300	-0.03472600
N	1.57753600	0.57582800	2.19844800
C	0.55171000	1.45906500	2.82398900
H	-0.34743400	1.42207200	2.21529300
H	0.33850200	1.08861300	3.82539300
H	0.93691100	2.47594700	2.87734500
C	2.85948000	0.50695500	2.95638800
H	3.28329700	1.50628800	3.04309400
H	3.54807100	-0.14649000	2.42479600
H	2.65704700	0.10191700	3.94632800
H	1.18193800	-0.39365200	2.10536400
H	1.76797400	0.89592500	1.23738500

conf_101

C	2.03158000	-1.39767800	-1.12373000
C	2.77916000	-2.66984300	-0.71033300
C	1.97682600	-3.64716000	0.15511100
C	1.61668400	-3.11137000	1.55252100
C	0.56617400	-2.03726100	1.60923000
O	0.63639800	-1.05352900	2.32459200
O	-0.54766500	-2.19641200	0.87170400
H	-0.47867500	-2.97211300	0.29690500
H	1.23340900	-3.93092100	2.17387500
H	2.49984400	-2.71835900	2.05719900
H	1.07609200	-3.97416700	-0.38524600
H	2.56023600	-4.55881000	0.30368400
H	3.09959100	-3.19968900	-1.61328800
H	3.69580900	-2.40538500	-0.17303200
C	2.80448300	-0.53869500	-2.13291500
C	4.14060500	0.01799400	-1.62234900
C	4.01819100	0.95447100	-0.40542800
C	3.11994900	2.12460300	-0.67997800
C	2.14271900	2.57383500	0.10489900
C	1.24886500	3.73493800	-0.23040600
C	-0.24974700	3.39266300	-0.14904000
C	-0.63475200	2.12597000	-0.91830200
C	-2.13987400	1.83631700	-0.93009900
C	-2.47690700	0.41352700	-1.38151600
C	-3.97306300	0.09719500	-1.36691000
C	-4.29085600	-1.35195300	-1.74401500
C	-5.78851400	-1.66122600	-1.73041500
H	-5.98416900	-2.70098000	-2.00228600
H	-6.21967500	-1.48916200	-0.73935800
H	-6.32667900	-1.02596700	-2.43939600
H	-3.88089600	-1.56170400	-2.73853600
H	-3.76990200	-2.02665000	-1.05169900
H	-4.38501000	0.30878200	-0.37045300
H	-4.49441600	0.77431500	-2.05318200
H	-1.94479600	-0.30543200	-0.74242700
H	-2.08020200	0.25533200	-2.39097300
H	-2.57473000	2.01247700	0.06787300
H	-2.64723600	2.56223900	-1.57365500
H	-0.27456800	2.20425400	-1.94939900
H	-0.09034800	1.26656500	-0.50323600
H	-0.53294900	3.28659800	0.90962300
H	-0.83395700	4.24183200	-0.51717200
H	1.48404900	4.08439500	-1.24038200
H	1.45062400	4.57616700	0.44334700
H	1.96287000	2.07130400	1.05801500
H	3.29795000	2.64043300	-1.62402700
H	5.02233100	1.31717200	-0.15533500
H	3.66151200	0.41037200	0.47593500
H	4.61559500	0.57070400	-2.43963600
H	4.82634500	-0.79884800	-1.37862900
H	2.16484400	0.29510500	-2.43925900
H	2.98893300	-1.13262000	-3.03458400
H	1.80126200	-0.78588600	-0.24467400

H	1.06849400	-1.67323900	-1.57081600
N	-1.29517800	0.83990600	2.26978600
C	-2.62496900	0.17397200	2.39083800
H	-2.65318300	-0.37181400	3.33233700
H	-3.40982800	0.92795100	2.37190000
H	-2.74252400	-0.51543900	1.55803800
C	-0.98480500	1.79815200	3.37059000
H	-0.99419200	1.25591600	4.31438100
H	-1.73351100	2.58860600	3.38477200
H	0.00333200	2.22037700	3.19832700
H	-0.54783000	0.09740200	2.24696000
H	-1.24797400	1.32836700	1.37211900

conf_263

C	-2.91864600	1.53327500	-0.93632900
C	-1.49655800	2.04699900	-1.17201500
C	-1.17714100	3.32631700	-0.39510000
C	0.32116800	3.69566000	-0.43673400
C	1.18330200	2.68763800	0.27398200
O	2.05864000	2.02656400	-0.25438900
O	0.96546500	2.49795100	1.58845600
H	0.23121800	3.04067600	1.90793900
H	0.67537000	3.75887200	-1.46539800
H	0.48527200	4.67111300	0.03521800
H	-1.74343100	4.16777000	-0.80176700
H	-1.51391200	3.22036900	0.64464500
H	-0.78910400	1.25966000	-0.89139300
H	-1.33578000	2.22800700	-2.24090300
C	-3.20697300	0.24358300	-1.70844000
C	-4.59830600	-0.35325600	-1.46395600
C	-4.89006200	-0.72621300	0.00438400
C	-3.82715500	-1.59711300	0.60924900
C	-3.16432300	-1.32692400	1.73271600
C	-2.05491000	-2.15207600	2.31798500
C	-0.73850700	-1.35153600	2.41407300
C	-0.22161000	-0.86880100	1.05315500
C	0.42388500	-1.96102500	0.19749800
C	0.72299700	-1.51014300	-1.23724300
C	1.53759700	-2.53177100	-2.04460100
C	3.04053400	-2.53059300	-1.72847400
C	3.78655500	-1.33933100	-2.34124400
H	3.74346200	-1.38842300	-3.43168700
H	3.34565400	-0.37664000	-2.05997900
H	4.84405600	-1.33449100	-2.06150800
H	3.18662400	-2.57298400	-0.63648100
H	3.49446600	-3.45380500	-2.09848800
H	1.12706200	-3.53048800	-1.86454900
H	1.41290700	-2.33997100	-3.11522600
H	1.23516600	-0.53889300	-1.23070300
H	-0.22890100	-1.32492000	-1.74329800
H	1.33989700	-2.31727000	0.69083200
H	-0.23292500	-2.83544400	0.15482500
H	-1.05988600	-0.43450700	0.50230400

H	0.49006000	-0.04893600	1.19830100
H	-0.90917300	-0.48466300	3.06271900
H	0.02124800	-1.96562000	2.91330800
H	-1.90953700	-3.05595100	1.71872600
H	-2.32854400	-2.48716800	3.32471400
H	-3.41369800	-0.41525700	2.27771800
H	-3.58291200	-2.50877900	0.06262200
H	-5.85488400	-1.24562400	0.03578700
H	-5.00923300	0.17582900	0.61228600
H	-4.70199700	-1.25035800	-2.08378400
H	-5.36756000	0.34597900	-1.80856100
H	-2.44975200	-0.50235500	-1.44096400
H	-3.08469500	0.43193100	-2.78086200
H	-3.64566200	2.30412000	-1.21862300
H	-3.05510000	1.35741900	0.13628800
N	3.55772700	0.01273000	0.80179200
C	3.27929700	-0.32733900	2.22873800
H	3.97224200	-1.10191000	2.55339400
H	3.41278700	0.57048800	2.82908000
H	2.25382400	-0.67729200	2.31348800
C	4.95499500	0.47991900	0.55464600
H	5.65249900	-0.31346500	0.81800400
H	5.13753100	1.35803700	1.17137300
H	5.05563500	0.73765300	-0.49681400
H	3.36326600	-0.80029400	0.21093800
H	2.90266900	0.76874600	0.47767100

conf_158

C	-4.44902700	1.65146600	-0.38361700
C	-3.76628900	2.74020500	-1.22109200
C	-2.23080900	2.72802600	-1.17858100
C	-1.63789200	3.04948900	0.22317200
C	-0.83048200	1.94718600	0.84690800
O	0.28796200	2.10205300	1.30897500
O	-1.36587900	0.71717100	0.92404700
H	-2.22561500	0.64952400	0.47891600
H	-0.99118200	3.92432500	0.19333200
H	-2.44619000	3.27952700	0.92792600
H	-1.85065700	1.77005000	-1.54657500
H	-1.85290200	3.47451400	-1.87882100
H	-4.08403800	2.63915900	-2.26360200
H	-4.11584000	3.72349200	-0.88930000
C	-4.20973800	0.22398500	-0.88765400
C	-4.88552500	-0.85020000	-0.03209800
C	-4.44634000	-2.27853000	-0.40823500
C	-3.02184800	-2.55832500	-0.02370100
C	-2.01034900	-2.75426300	-0.86895200
C	-0.58725900	-3.00894100	-0.46514500
C	0.36582900	-1.88875300	-0.91013000
C	1.81760500	-2.12605500	-0.49099300
C	2.75229000	-0.96266800	-0.84156000
C	4.15525600	-1.09601700	-0.23853600
C	5.04353300	0.13904600	-0.42749100

C	5.38923900	0.45341200	-1.88665500
C	6.34504700	1.63976300	-2.02220500
H	6.57818200	1.84435800	-3.06944300
H	5.90992400	2.54860300	-1.59510300
H	7.28829300	1.44747600	-1.50287500
H	5.83800200	-0.43594300	-2.34428900
H	4.47379800	0.66039400	-2.45084000
H	4.55197900	1.01624400	0.01779900
H	5.97412800	-0.00502300	0.13246000
H	4.65028400	-1.97330000	-0.66920900
H	4.07247400	-1.31541900	0.83687200
H	2.82069300	-0.88060400	-1.92944800
H	2.30373200	-0.00831900	-0.52146400
H	1.85121600	-2.33173600	0.59190700
H	2.19490500	-3.04483600	-0.95176500
H	0.31699400	-1.77601300	-1.99890600
H	0.01146200	-0.94004900	-0.49047000
H	-0.24520400	-3.95739500	-0.89699600
H	-0.53323200	-3.12805600	0.62363500
H	-2.20803700	-2.72996800	-1.94032700
H	-2.81998100	-2.59193400	1.04827500
H	-4.58069600	-2.43317000	-1.48372500
H	-5.10352100	-2.99356800	0.09771500
H	-5.97134900	-0.76202400	-0.13282200
H	-4.66419000	-0.67510400	1.02901000
H	-3.13653000	-0.00382200	-0.95908000
H	-4.56877600	0.14559800	-1.92009000
H	-5.52618100	1.84193100	-0.36962300
H	-4.14036600	1.73367900	0.66968700
N	1.69640500	-0.01626600	2.24952000
C	2.87461500	0.49758900	3.00723900
H	3.45082900	-0.34200900	3.39262700
H	3.48818000	1.09399700	2.33473400
H	2.51654200	1.11521400	3.82890100
C	0.77842800	-0.88479000	3.04226200
H	0.41901100	-0.31783500	3.89934500
H	1.31763900	-1.76875900	3.37883400
H	-0.06034700	-1.16491400	2.40860300
H	1.14388500	0.79972800	1.87271100
H	2.02349300	-0.53769100	1.43304800

11E_NMe2H2
conf_1

C	-2.38568400	2.05898700	-0.91186200
C	-3.76146900	1.45262200	-1.20424100
C	-3.70984000	-0.00630200	-1.67322000
C	-3.11552300	-0.98347100	-0.65340500
C	-3.16723000	-2.43914600	-1.13100600
C	-2.48203300	-3.44049200	-0.19552300
C	-0.97577700	-3.38808800	-0.13409300
C	-0.16186800	-2.69992100	-0.94205400
C	1.33921300	-2.73037100	-0.90703800
C	1.96166100	-1.32648800	-0.95288900

C	3.48358900	-1.32783000	-0.80721600
C	4.09138100	0.07595500	-0.84638200
C	5.61401500	0.08793000	-0.69175300
C	6.20601900	1.49764000	-0.72770900
H	5.97994800	1.99620200	-1.67521100
H	5.80171700	2.11668200	0.07958200
H	7.29239900	1.47710600	-0.61608300
H	5.88455900	-0.40033900	0.25193600
H	6.06089800	-0.51873000	-1.48732800
H	3.82136700	0.55839400	-1.79461500
H	3.64119300	0.68824600	-0.05382500
H	3.92354800	-1.94067700	-1.60238400
H	3.76171000	-1.81765900	0.13563900
H	1.68561200	-0.84714100	-1.89933000
H	1.52604200	-0.68906100	-0.17385700
H	1.70394700	-3.29881400	-1.77143400
H	1.68517200	-3.27779600	-0.02235600
H	-0.59549000	-2.07047100	-1.71635800
H	-0.52051400	-4.04667600	0.60853300
H	-2.75767900	-4.46002900	-0.49251000
H	-2.88857900	-3.33088500	0.81897200
H	-2.73092100	-2.51413500	-2.13290800
H	-4.21496100	-2.73351700	-1.24243700
H	-3.66629300	-0.89571300	0.29295600
H	-2.07929100	-0.70048200	-0.44069700
H	-3.13113000	-0.06684600	-2.60330800
H	-4.72382000	-0.33335600	-1.92608700
H	-4.26170000	2.05346500	-1.97094900
H	-4.38537700	1.52554900	-0.30486500
C	-2.45289200	3.54199800	-0.53557000
C	-1.09989600	4.20010800	-0.23685700
C	-0.43957400	3.74659600	1.08283700
C	0.04091000	2.32061100	1.11001900
O	-0.26962500	1.51198900	1.96633300
O	0.88093300	1.92236100	0.14163900
H	1.03773100	2.62622400	-0.50301800
H	0.43344000	4.37766300	1.28890600
H	-1.13099100	3.86719100	1.91686500
H	-0.41811100	4.04972700	-1.08510500
H	-1.23435400	5.28181400	-0.16370000
H	-2.92643900	4.09264800	-1.35438600
H	-3.10866400	3.66952200	0.33388600
H	-1.91933700	1.48944800	-0.10424000
H	-1.74131200	1.93555900	-1.79226500
N	-0.19497300	-1.20585900	2.05732600
C	1.08168000	-1.68481600	2.65874600
H	1.02959300	-2.76319700	2.79980800
H	1.90059700	-1.43561700	1.98738400
H	1.22471600	-1.19008500	3.61789400
C	-1.39669200	-1.46991900	2.89957600
H	-2.27312900	-1.08727100	2.38128800
H	-1.27677200	-0.95774000	3.85242000
H	-1.49467100	-2.54254900	3.05840100
H	-0.13019200	-0.17296200	1.90051400
H	-0.33005600	-1.66110600	1.13704900

conf_39

C	1.35742700	-0.98687800	1.64551300
C	0.71658300	-1.03715800	3.03698900
C	-0.47095200	-0.08509100	3.21784300
C	-1.65528700	-0.35569700	2.28412100
C	-2.83379900	0.59903600	2.52920700
C	-3.84353600	0.67002100	1.37802300
C	-3.33915000	1.30897200	0.10514000
C	-2.17849100	1.95818400	-0.03188600
C	-1.67119700	2.64689900	-1.26426300
C	-0.22013700	2.25567600	-1.60867100
C	0.81136800	2.71729500	-0.57305800
C	2.23488200	2.25014400	-0.88106200
C	3.26797000	2.71412000	0.14800100
C	4.67598800	2.19253800	-0.14130500
H	5.39396600	2.54704600	0.60136500
H	4.70405400	1.09758200	-0.12797600
H	5.02582000	2.52172200	-1.12407200
H	3.27973000	3.80892600	0.17565900
H	2.95218400	2.38865900	1.14657000
H	2.24373800	1.15261800	-0.91394900
H	2.53139800	2.59376500	-1.87952000
H	0.53746700	2.35129800	0.42233700
H	0.79296700	3.81138300	-0.51046300
H	-0.14742800	1.16705100	-1.71855000
H	0.03809200	2.68098400	-2.58421800
H	-1.70742600	3.73164300	-1.10248500
H	-2.33498500	2.44400500	-2.11126800
H	-1.52493400	2.02215600	0.83210000
H	-4.02701300	1.28481700	-0.74245800
H	-4.72818900	1.23054000	1.70380900
H	-4.22224400	-0.33684500	1.15641600
H	-2.45384500	1.60543700	2.73429200
H	-3.35835600	0.29283300	3.43845200
H	-1.99153100	-1.39290600	2.40800800
H	-1.31491200	-0.26240800	1.24839700
H	-0.13123700	0.94745800	3.06558000
H	-0.81583500	-0.13832400	4.25577600
H	1.47718900	-0.80036100	3.78850600
H	0.39442200	-2.06504900	3.24344300
C	2.59111700	-1.88910600	1.53846900
C	3.22146700	-1.98376500	0.14343300
C	2.41730200	-2.83201700	-0.86973500
C	1.12891300	-2.21276100	-1.33211000
O	0.02496700	-2.69949200	-1.17244100
O	1.19904200	-1.03341500	-1.97998300
H	2.10414000	-0.69312300	-2.01689800
H	3.02945500	-3.00791000	-1.76190700
H	2.17311700	-3.80377000	-0.44088200
H	3.40587200	-0.97744500	-0.25284500
H	4.20519200	-2.45154400	0.22594800
H	3.35320500	-1.52434300	2.23417100

H	2.33305800	-2.89950300	1.87693200
H	0.61035900	-1.28426100	0.90618900
H	1.63948400	0.04618600	1.40581300
N	-2.21780100	-1.16922100	-1.53191000
C	-2.30073000	-0.73535200	-2.95549600
H	-1.44109800	-0.10696500	-3.17747700
H	-2.28785300	-1.61855900	-3.59184800
H	-3.22397400	-0.17879600	-3.10761000
C	-3.32585300	-2.07216200	-1.10725800
H	-3.17205600	-2.34363400	-0.06504900
H	-3.30543800	-2.96598000	-1.72824900
H	-4.27678500	-1.55544500	-1.22448400
H	-1.31297300	-1.67855700	-1.38896900
H	-2.21755700	-0.32996800	-0.92717800

conf_141

C	5.66976700	1.76047000	-0.40542000
C	4.27592200	2.38618100	-0.56768700
C	3.56902500	2.63894900	0.77006700
C	2.10069000	3.06004300	0.63506900
C	1.17044800	1.91466200	0.22416700
C	-0.30605300	2.33525400	0.15522700
C	-1.21656600	1.24623200	-0.33377600
C	-2.21142100	0.69202200	0.36997400
C	-3.18050800	-0.33299200	-0.14548200
C	-4.60590800	0.23069700	-0.28389600
C	-5.61065600	-0.82337500	-0.75716400
C	-7.02432900	-0.27820300	-0.99345500
C	-7.72577200	0.23967400	0.26646700
C	-9.16662900	0.67906700	-0.00060200
H	-9.76677700	-0.15138900	-0.38387700
H	-9.64764800	1.04575000	0.90937800
H	-9.20165100	1.48238200	-0.74258000
H	-7.71726600	-0.54787900	1.03030000
H	-7.16608400	1.08193400	0.68724200
H	-6.98316100	0.52322800	-1.74173800
H	-7.63554000	-1.07303800	-1.43526900
H	-5.65523300	-1.63641900	-0.02078300
H	-5.24178600	-1.27096600	-1.68786100
H	-4.91841300	0.64544400	0.67939000
H	-4.58596000	1.06799100	-0.99005000
H	-3.22273100	-1.19023400	0.54113700
H	-2.84491700	-0.71379000	-1.11705100
H	-2.39413200	1.05619800	1.38160500
H	-1.05826100	0.91414900	-1.36182900
H	-0.39215800	3.19240300	-0.52344300
H	-0.63878600	2.68249500	1.13857400
H	1.28257500	1.10200100	0.95483400
H	1.47992000	1.50182800	-0.74166900
H	2.01613300	3.87503700	-0.09341000
H	1.75243500	3.46773600	1.59045400
H	4.11878300	3.41600800	1.31104400
H	3.61993300	1.74159700	1.39919600

H	3.65856200	1.74300400	-1.20376500
H	4.36504600	3.33883400	-1.09957100
C	5.63126600	0.26608600	-0.05324100
C	5.28958200	-0.62058000	-1.25743700
C	4.90735900	-2.04634000	-0.85395400
C	3.54277700	-2.12331100	-0.21597500
O	2.65847700	-1.31033800	-0.39389400
O	3.27409600	-3.18549900	0.57258400
H	4.04618300	-3.76034900	0.66527400
H	4.86509200	-2.70320800	-1.73260000
H	5.65455600	-2.47994000	-0.17966400
H	4.46214200	-0.19216100	-1.82679400
H	6.14713900	-0.66729200	-1.93342000
H	6.59906900	-0.04609300	0.35286900
H	4.89892200	0.10311700	0.74616900
H	6.24543600	1.89194800	-1.32795400
H	6.21597500	2.30370300	0.37249500
N	0.13537300	-1.53083800	0.67684700
C	-0.43824100	-2.73895900	0.01938300
H	0.22196700	-3.58397800	0.20784400
H	-1.42859300	-2.93529200	0.42492000
H	-0.50688500	-2.55431800	-1.05115100
C	0.24302800	-1.64008300	2.15820200
H	0.90296300	-2.47101000	2.40138600
H	-0.74698200	-1.80885800	2.57885700
H	0.65710300	-0.71134100	2.54694900
H	1.08969600	-1.37149600	0.28079400
H	-0.43755000	-0.69814700	0.44351300

conf_37

C	-4.74579300	1.28193800	-0.78218700
C	-4.37962500	1.55928100	0.68165500
C	-3.56856000	2.84932100	0.87597100
C	-2.23350400	2.91462000	0.12346100
C	-1.19404300	1.89566800	0.59683000
C	0.13350400	2.00513100	-0.17137300
C	1.23751800	1.16384800	0.39956800
C	1.92228100	0.21996600	-0.25782700
C	3.09497300	-0.54211700	0.29024400
C	4.41836500	-0.13538200	-0.38281500
C	5.61315800	-0.93885800	0.13815000
C	6.93863000	-0.61281200	-0.56063800
C	7.44163100	0.81710700	-0.33687300
C	8.81210800	1.06443800	-0.97010300
H	8.78164600	0.89907500	-2.05127300
H	9.15167200	2.08892100	-0.79986100
H	9.56572600	0.38968500	-0.55358500
H	6.72252300	1.53537300	-0.74507800
H	7.49393400	1.01331700	0.74117200
H	7.70194800	-1.31388200	-0.20515300
H	6.83609500	-0.79750700	-1.63751200
H	5.40022500	-2.00795600	0.01694700
H	5.72140300	-0.76700400	1.21668600

H	4.32944000	-0.27407500	-1.46694600
H	4.57500700	0.93433200	-0.21946300
H	2.95218200	-1.61959700	0.13362400
H	3.17517800	-0.38036100	1.37159300
H	1.66983500	0.02858600	-1.30139600
H	1.51972700	1.38423200	1.43100800
H	0.45686800	3.05330800	-0.15201100
H	-0.02412600	1.74843300	-1.22371200
H	-1.59662600	0.88274500	0.48578800
H	-1.00967000	2.04870200	1.66822800
H	-1.81342700	3.91895300	0.24246800
H	-2.40160200	2.78679600	-0.95175300
H	-3.38237200	2.99665400	1.94611300
H	-4.18562600	3.69647600	0.55781200
H	-3.84483200	0.70363800	1.10487900
H	-5.30455700	1.65091900	1.26184300
C	-5.89312300	0.28159600	-0.97014300
C	-5.62059600	-1.15925400	-0.52031100
C	-4.52673500	-1.86455200	-1.34128600
C	-3.12426700	-1.60795000	-0.85770800
O	-2.81368900	-1.56156400	0.31731500
O	-2.14280300	-1.48680700	-1.77254400
H	-2.49545100	-1.52153400	-2.67188600
H	-4.65118800	-2.95160000	-1.26159100
H	-4.62059100	-1.61695500	-2.40350100
H	-5.34952300	-1.20057500	0.53717800
H	-6.54064300	-1.73774500	-0.62877500
H	-6.76729000	0.64834200	-0.42259600
H	-6.18548000	0.26586100	-2.02663400
H	-5.04501900	2.22442100	-1.25400000
H	-3.85793800	0.95387300	-1.33902600
N	-0.26918500	-1.53536600	1.33070100
C	-0.38656200	-1.18573900	2.77377000
H	-1.04145300	-1.90650700	3.26036300
H	0.60170700	-1.20971200	3.23056300
H	-0.81139200	-0.18734900	2.85491100
C	0.29731100	-2.88840600	1.07268700
H	0.36831500	-3.03629300	-0.00357400
H	-0.36433500	-3.63674200	1.50622400
H	1.28556300	-2.95743000	1.52337700
H	-1.21656300	-1.48195800	0.88991800
H	0.31692500	-0.82038500	0.85911800

conf_129

C	-5.30970200	-1.52713000	-0.43547600
C	-4.62143300	-2.82905200	-0.87826800
C	-3.11301100	-2.88841300	-0.59344500
C	-2.73768700	-2.72111000	0.88456700
C	-1.23909800	-2.49481100	1.12210600
C	-0.76453500	-1.10412400	0.65769600
C	0.68097300	-0.84922600	0.97340200
C	1.65302900	-0.68694200	0.06702500
C	3.11102100	-0.50846700	0.37231600

C	3.95302900	-1.70040000	-0.12086200
C	5.44944700	-1.56801300	0.18488100
C	6.14664400	-0.40755800	-0.53081400
C	7.65988000	-0.37983200	-0.29842000
C	8.35206000	0.78732300	-1.00374900
H	9.42964100	0.77929600	-0.82415300
H	8.19561100	0.74305900	-2.08585900
H	7.96450000	1.74843000	-0.65098600
H	7.85755600	-0.32881900	0.77868000
H	8.09211500	-1.32610200	-0.64271300
H	5.94797300	-0.47551100	-1.60853800
H	5.72469500	0.55003900	-0.20080400
H	5.94122400	-2.50439300	-0.09929100
H	5.59225900	-1.46846100	1.26811000
H	3.56352400	-2.61175500	0.34360800
H	3.81002400	-1.81797300	-1.20175100
H	3.25460800	-0.38148000	1.45109700
H	3.47945200	0.40467400	-0.11075100
H	1.38872300	-0.74533500	-0.99058500
H	0.95032700	-0.84309400	2.03047200
H	-1.39117500	-0.35072000	1.15606300
H	-0.92642500	-0.99020300	-0.41838400
H	-0.65348500	-3.26279100	0.60572000
H	-1.01839000	-2.60254700	2.18929100
H	-3.06318400	-3.60872200	1.43626900
H	-3.28531400	-1.87891800	1.32369600
H	-2.60809600	-2.12732900	-1.19689800
H	-2.72431900	-3.84811700	-0.94910100
H	-4.77659700	-2.96692600	-1.95369500
H	-5.12083600	-3.67134000	-0.38826800
C	-4.61301300	-0.26077800	-0.93742700
C	-5.30015300	1.03040900	-0.48820800
C	-4.47519700	2.28666500	-0.82296900
C	-3.10911600	2.28490200	-0.18653700
O	-2.06989200	2.48989400	-0.78266400
O	-3.03767300	2.04379200	1.13886000
H	-3.91372500	1.86471500	1.50880800
H	-4.32372700	2.38136100	-1.89816900
H	-4.99556000	3.19019600	-0.48368800
H	-6.27950600	1.12913300	-0.96274700
H	-5.50876300	0.98304600	0.59015700
H	-3.57824600	-0.25682100	-0.57812800
H	-4.55073100	-0.27506900	-2.03179000
H	-6.34673300	-1.53754300	-0.78690800
H	-5.36531500	-1.49003000	0.65849400
N	0.42716700	2.28033900	0.35708200
C	0.38047000	2.81310500	1.74728100
H	1.35572200	2.68441400	2.21422600
H	0.11933100	3.86958800	1.71348500
H	-0.37928400	2.26597900	2.30190800
C	1.39612100	2.97527600	-0.53636400
H	1.10594000	4.02042000	-0.63011200
H	1.37324600	2.49833300	-1.51465000
H	2.39503500	2.90096800	-0.10958800
H	0.67099300	1.27190100	0.39783100

H	-0.53110900	2.34164500	-0.05992200

conf_3

C	-2.49344800	1.05857000	1.11886600
C	-1.92473700	0.94025500	2.53439600
C	-0.71012100	1.83810800	2.79873800
C	0.46364300	1.60661400	1.83761800
C	1.78512800	2.17572900	2.36244600
C	2.98920900	1.96678600	1.42832100
C	3.15661600	0.52774600	1.02026600
C	3.71605200	0.07020400	-0.10326500
C	3.89459800	-1.38808800	-0.46380500
C	2.95622400	-2.36940300	0.25380400
C	1.47256700	-2.17048600	-0.07692800
C	0.52879900	-3.04112300	0.75486300
C	-0.94885100	-2.75451700	0.47473300
C	-1.89769200	-3.59265200	1.33171200
H	-1.74387600	-4.66163300	1.16097500
H	-1.73966900	-3.40275700	2.39744100
H	-2.94523800	-3.37211800	1.10793300
H	-1.13952300	-1.68876500	0.64813300
H	-1.15310400	-2.92699200	-0.58800100
H	0.74066800	-4.10020800	0.56878100
H	0.73255800	-2.87299500	1.81981600
H	1.30473400	-2.37282600	-1.14291400
H	1.19351300	-1.12480700	0.08998000
H	3.25087800	-3.38805500	-0.01531700
H	3.10307300	-2.29712600	1.33644900
H	4.93612900	-1.66815500	-0.26474500
H	3.77887600	-1.50627500	-1.54844000
H	4.14403700	0.79260000	-0.80177300
H	2.79124900	-0.19848500	1.74257800
H	3.89360900	2.29895900	1.95170600
H	2.90886300	2.60631500	0.54140600
H	2.00641700	1.70090400	3.32511200
H	1.67241500	3.24417100	2.57116900
H	0.23300700	2.04940900	0.85987500
H	0.57398100	0.52900900	1.66602500
H	-0.36606500	1.66040000	3.82302900
H	-1.01069000	2.89155100	2.75919400
H	-1.64410400	-0.10488900	2.71527900
H	-2.70932500	1.17580000	3.26098100
C	-3.74394700	0.20079300	0.90723200
C	-4.34730500	0.26403000	-0.50124800
C	-3.48891600	-0.39774600	-1.59820300
C	-2.19657100	0.29758700	-1.93248800
O	-1.12372400	-0.26938700	-2.02240900
O	-2.23484400	1.62018700	-2.17610600
H	-3.12334900	1.97573400	-2.03363500
H	-3.23456500	-1.42091200	-1.32180500
H	-4.06305700	-0.44709400	-2.53166300
H	-5.31104500	-0.25054000	-0.50283600
H	-4.58034500	1.30626800	-0.76159700

H	-3.51231500	-0.84333700	1.14576700
H	-4.51154300	0.51212500	1.62250900
H	-2.72796100	2.10978800	0.90512900
H	-1.71346100	0.76361600	0.41177100
N	1.35307600	0.85540800	-2.03388000
C	2.00560900	0.23550200	-3.22221000
H	1.98384600	-0.84545600	-3.10567800
H	1.45028200	0.52189700	-4.11366900
H	3.03397400	0.58566300	-3.29352100
C	1.26744200	2.34117800	-2.10425700
H	0.66946000	2.61663500	-2.97089300
H	2.27082400	2.75494100	-2.19141700
H	0.78975400	2.70738700	-1.19845900
H	0.38589100	0.45895800	-1.96069000
H	1.87241500	0.58251800	-1.18225000

conf_225

C	4.16251300	-0.08563300	-2.01062100
C	4.93965300	-1.08479800	-1.14037900
C	4.12814700	-1.73459200	-0.01040500
C	2.92619800	-2.55420200	-0.49077700
C	2.13454000	-3.21967000	0.64266800
C	1.39672500	-2.24325000	1.58581200
C	0.33097800	-1.45160600	0.88259900
C	-0.98734600	-1.61586900	1.05025800
C	-2.05563900	-0.91585300	0.25993500
C	-2.76651700	-1.86996800	-0.71890700
C	-3.83674100	-1.18539400	-1.57694200
C	-5.02242300	-0.62043000	-0.78962200
C	-6.12559300	-0.05292600	-1.68704600
C	-7.30147900	0.52385200	-0.89742700
H	-6.97747000	1.34160600	-0.24572500
H	-8.07396800	0.91682200	-1.56244300
H	-7.76458000	-0.24001600	-0.26575000
H	-5.70003600	0.72362400	-2.33391200
H	-6.48431900	-0.84299600	-2.35620400
H	-5.44362800	-1.41044000	-0.15458600
H	-4.68315700	0.17051600	-0.10887600
H	-4.21072900	-1.91299100	-2.30504100
H	-3.37345500	-0.38168100	-2.16412800
H	-2.01021400	-2.32314900	-1.36768500
H	-3.22067700	-2.69182300	-0.15347800
H	-1.62038800	-0.08043300	-0.29983600
H	-2.80490700	-0.49627500	0.94116000
H	-1.33006800	-2.36130800	1.76853900
H	0.66511900	-0.73297700	0.13409700
H	2.12954000	-1.56623400	2.04136800
H	0.94462300	-2.81463400	2.40187200
H	2.81467300	-3.83013700	1.24593300
H	1.39486100	-3.90422900	0.21631800
H	3.28364700	-3.33588900	-1.16948800
H	2.25425800	-1.92976100	-1.08972700
H	3.80125300	-0.96010100	0.69238900

H	4.79282500	-2.39021000	0.56265400
H	5.80358300	-0.57324000	-0.70193800
H	5.34636900	-1.86927300	-1.78713000
C	3.51541300	1.04692700	-1.21108300
C	2.82866500	2.09210100	-2.09142800
C	2.13156700	3.20013200	-1.27198200
C	1.10424000	2.63243900	-0.33137800
O	1.16706600	2.69297100	0.88086600
O	0.05732400	1.97846400	-0.87695900
H	0.10685800	1.98038800	-1.84293900
H	2.85565600	3.74552200	-0.66740500
H	1.64058300	3.91724600	-1.93888000
H	3.55855400	2.57188300	-2.74822800
H	2.10886600	1.59956500	-2.75798500
H	2.78369600	0.62366000	-0.51308300
H	4.27214300	1.54106400	-0.59009800
H	4.84817000	0.34165100	-2.75006800
H	3.38907200	-0.60865600	-2.58436100
N	-0.32919200	1.16691400	2.60317300
C	-1.69193600	1.73358700	2.80737900
H	-1.60096700	2.71233100	3.27589900
H	-2.17708600	1.82967900	1.83820700
H	-2.26743700	1.06705100	3.44794200
C	0.46874300	1.01737500	3.85312900
H	1.43877800	0.59463500	3.59830500
H	-0.05702300	0.35432100	4.53858000
H	0.60140600	1.99716100	4.30870800
H	-0.40023600	0.24176600	2.14006300
H	0.19678000	1.77274000	1.93080600

conf_60

C	-4.29390700	-1.88979000	-0.06800500
C	-2.85810200	-2.39733100	-0.25875200
C	-2.24649800	-2.99764100	1.01520000
C	-2.11003400	-2.03082000	2.19973600
C	-1.17951000	-0.83532900	1.95718600
C	0.27199200	-1.23876900	1.64234600
C	1.23859100	-0.09158100	1.68870900
C	2.05299400	0.28535200	0.69497300
C	3.08666000	1.37313000	0.79536000
C	4.53043900	0.83115300	0.84190000
C	4.95905100	0.07790500	-0.41952700
C	6.42435800	-0.36185200	-0.38133400
C	6.86390800	-1.11930100	-1.63674400
C	8.33007900	-1.55245700	-1.58957800
H	8.61652900	-2.09014100	-2.49657900
H	8.99335000	-0.68790100	-1.49168400
H	8.51867600	-2.21247800	-0.73763500
H	6.22480100	-2.00073200	-1.76794300
H	6.69620800	-0.48633100	-2.51638300
H	7.06374700	0.51945200	-0.24737700
H	6.58936000	-0.99438500	0.49966200
H	4.79629100	0.71765500	-1.29713000

H	4.32718100	-0.80658000	-0.56126000
H	5.20656600	1.67741500	1.00335000
H	4.63284400	0.17854600	1.71550700
H	2.90092200	1.97240000	1.69357500
H	3.00717900	2.04622400	-0.06864200
H	2.02032100	-0.27468000	-0.23878000
H	1.29654800	0.44497300	2.63791500
H	0.33004300	-1.72914800	0.66702000
H	0.58880800	-1.98304800	2.38425900
H	-1.18649700	-0.20401400	2.85392400
H	-1.57479200	-0.22500500	1.13714800
H	-1.73856300	-2.58912700	3.06583500
H	-3.09833400	-1.65698000	2.48605300
H	-1.26766400	-3.42460800	0.77491700
H	-2.86716700	-3.84213000	1.33344000
H	-2.22255100	-1.59488500	-0.64731000
H	-2.86399900	-3.17547800	-1.03009100
C	-5.08050300	-1.72284900	-1.37429300
C	-4.58819700	-0.62930600	-2.33053400
C	-4.77893300	0.79614900	-1.78484900
C	-3.67611300	1.27174900	-0.87739800
O	-2.49640000	1.04929600	-1.07046500
O	-4.00528200	2.04870800	0.17415800
H	-4.96373400	2.14697800	0.25213000
H	-4.77784600	1.51128600	-2.61690200
H	-5.75198600	0.89257200	-1.29257800
H	-3.53639500	-0.77176000	-2.58945500
H	-5.15152600	-0.70219600	-3.26347800
H	-5.06407600	-2.67653800	-1.91153000
H	-6.13373100	-1.52971100	-1.13881900
H	-4.83778400	-2.60528300	0.55853800
H	-4.29329900	-0.95218400	0.50334500
N	-0.42507400	2.27671800	0.25396500
C	-0.88666200	2.96788000	1.48973700
H	-1.64888000	3.69751000	1.22337400
H	-1.30976700	2.22630200	2.16381800
H	-0.03864500	3.46251200	1.96086800
C	0.18818100	3.17998000	-0.75935400
H	-0.55627000	3.90595800	-1.08254700
H	0.51308300	2.58233300	-1.60920400
H	1.04048800	3.69177900	-0.31631700
H	0.25774500	1.54024200	0.51775800
H	-1.23497700	1.78249300	-0.18677500

conf_125

C	-2.09883200	-2.64458100	1.35516300
C	-3.14844800	-3.14793000	0.35248300
C	-3.24652600	-2.34481900	-0.95198200
C	-2.02308900	-2.47982900	-1.86680100
C	-2.05851900	-1.53723600	-3.07615400
C	-1.76737000	-0.06000400	-2.71602800
C	-0.32728500	0.14416700	-2.33889400
C	0.13710300	0.19309300	-1.08517800

C	1.57792200	0.24835300	-0.67333600
C	1.97483600	-0.98983800	0.15113100
C	3.37032500	-0.90049800	0.77739700
C	4.51523000	-0.82211700	-0.23631600
C	5.89946700	-0.84070900	0.41745900
C	7.04015500	-0.75452400	-0.59759700
H	6.98074500	0.16910100	-1.18157200
H	8.01458100	-0.77294100	-0.10413200
H	7.00725000	-1.59321500	-1.29929800
H	5.97441500	-0.00735900	1.12643900
H	6.00369200	-1.75621000	1.01097600
H	4.43351900	-1.66383200	-0.93576100
H	4.42159300	0.08784500	-0.84081800
H	3.52291700	-1.77666000	1.41705500
H	3.41649900	-0.02629100	1.44166000
H	1.23064200	-1.13702600	0.94344100
H	1.91041500	-1.87640300	-0.48894400
H	1.75675400	1.13821700	-0.05550600
H	2.21413800	0.33803000	-1.55716600
H	-0.58386900	0.11666500	-0.27114200
H	0.38878800	0.16535500	-3.16005900
H	-2.42141800	0.24490600	-1.89175700
H	-2.01875300	0.56699900	-3.57783500
H	-3.04069300	-1.59256700	-3.55651700
H	-1.32969700	-1.86367000	-3.82475500
H	-1.96490800	-3.51366700	-2.22184600
H	-1.10135200	-2.30549100	-1.30254800
H	-3.43036900	-1.29071000	-0.71629200
H	-4.13115700	-2.67912700	-1.50407000
H	-4.12739800	-3.14371900	0.84437900
H	-2.93373000	-4.19486500	0.11296700
C	-2.34398800	-1.20947600	1.83061300
C	-1.41030000	-0.79084300	2.96787100
C	-1.56715400	0.69563900	3.37428000
C	-1.11631900	1.60829200	2.26912400
O	-1.85786100	2.19072300	1.50215700
O	0.21473400	1.74235100	2.07873400
H	0.72042000	1.24204300	2.73364600
H	-2.61239000	0.92154700	3.58505900
H	-0.98408000	0.90301100	4.27709700
H	-1.61178600	-1.39757500	3.85429100
H	-0.36877700	-0.99545200	2.69227400
H	-2.23481700	-0.51890400	0.98878200
H	-3.38230800	-1.10531500	2.16686900
H	-2.09979700	-3.31321500	2.22286400
H	-1.09444000	-2.71486000	0.92209800
N	-0.89768300	3.13201800	-0.90338000
C	-1.99728700	3.67015700	-1.75341100
H	-2.77772500	2.91588900	-1.83274000
H	-2.39717600	4.56931900	-1.28764400
H	-1.60544200	3.90557700	-2.74178700
C	0.24888900	4.06429700	-0.70922700
H	0.96757800	3.59134300	-0.04306700
H	-0.11772900	4.98685400	-0.26194000
H	0.70920700	4.27540300	-1.67317200

H	-1.27644800	2.85870900	0.03327600
H	-0.55095500	2.24397800	-1.30985800

conf_0

C	-3.13148000	1.31679300	-0.35922100
C	-4.12943300	0.26081200	-0.84293300
C	-3.93251900	-1.11125100	-0.18462200
C	-2.67400800	-1.85796200	-0.64273300
C	-2.50925600	-3.22256000	0.03108300
C	-1.32605500	-4.04464800	-0.51127500
C	0.04101000	-3.57092200	-0.09742000
C	0.95284100	-3.04530300	-0.92279100
C	2.37114600	-2.69595400	-0.57827500
C	2.77923400	-1.27835800	-1.00872200
C	4.22574400	-0.93651900	-0.63849100
C	4.62740500	0.50872100	-0.95591100
C	3.94506100	1.55857000	-0.07159900
C	4.39107200	2.98617000	-0.39403500
H	5.47112200	3.10091100	-0.26729300
H	4.15153500	3.24681200	-1.42921400
H	3.90226400	3.71640500	0.25667100
H	2.85800000	1.49216300	-0.18028300
H	4.17113600	1.33662200	0.98001400
H	5.71235600	0.60726800	-0.84483300
H	4.40967700	0.72324100	-2.00937100
H	4.89284400	-1.62618100	-1.16606300
H	4.38992800	-1.12773800	0.43131800
H	2.65295900	-1.18344400	-2.09290800
H	2.09398100	-0.54112100	-0.57742700
H	3.03409800	-3.41125100	-1.08090900
H	2.54686200	-2.84069400	0.49478700
H	0.67164700	-2.89233900	-1.96438800
H	0.32394800	-3.77215500	0.93946400
H	-1.38245800	-4.06902500	-1.60403000
H	-1.43792900	-5.08045600	-0.17302500
H	-3.42672300	-3.79971000	-0.12062800
H	-2.41272000	-3.10142900	1.11795600
H	-1.77738700	-1.24732200	-0.47957300
H	-2.72756000	-2.00385000	-1.72804800
H	-4.80186200	-1.73977800	-0.40185500
H	-3.92232500	-0.99165400	0.90776800
H	-4.04906200	0.15421000	-1.93124600
H	-5.14592600	0.61533500	-0.64456900
C	-3.25559300	2.65549700	-1.09321300
C	-2.39179800	3.79012000	-0.52723300
C	-0.86954800	3.53475700	-0.57898000
C	-0.36620600	2.48395800	0.37343700
O	0.31762200	1.53174600	0.04942400
O	-0.68796900	2.61184900	1.67558500
H	-1.25408000	3.38229300	1.82329000
H	-0.56131500	3.23336500	-1.58024900
H	-0.33557000	4.46194500	-0.33846200
H	-2.58251500	4.70315200	-1.09584000

H	-2.70981200	4.01842600	0.49937100
H	-3.00419400	2.51084000	-2.15014700
H	-4.29980400	2.98347600	-1.07173100
H	-3.27782200	1.47293200	0.71810100
H	-2.11935000	0.92592200	-0.48999400
N	0.38520800	-0.73234400	1.58738900
C	1.63386400	-0.78545700	2.40114400
H	1.66300100	-1.72227600	2.95515900
H	2.49082400	-0.71145700	1.73726700
H	1.63053700	0.05603900	3.09187300
C	-0.86001500	-0.75614100	2.40714800
H	-1.71837100	-0.72491200	1.74144900
H	-0.86273300	0.11805700	3.05478700
H	-0.88101300	-1.66837200	3.00083200
H	0.39662900	0.14551700	1.01569600
H	0.36471000	-1.51406400	0.91233600

conf_143

C	-4.73481600	-2.46097200	-0.04724900
C	-3.28511600	-2.96175400	0.04815500
C	-2.65522600	-2.70327700	1.42216500
C	-1.16583100	-3.05904800	1.51495900
C	-0.22263400	-2.17504800	0.68700400
C	-0.24085800	-0.69290100	1.10470800
C	0.77873300	0.11616700	0.35762100
C	1.82869100	0.73873600	0.90615500
C	2.89963300	1.47513200	0.15380200
C	4.27169900	0.77517000	0.23987200
C	4.29974300	-0.58847900	-0.45518200
C	5.63954100	-1.32432200	-0.33097800
C	6.81698200	-0.62987900	-1.02311200
C	8.10651900	-1.45021800	-0.95568200
H	8.93271800	-0.93735000	-1.45384600
H	8.40403200	-1.63086100	0.08163200
H	7.97936100	-2.42413500	-1.43757200
H	6.55511500	-0.44181700	-2.07171400
H	6.99223500	0.35164200	-0.56996200
H	5.87660900	-1.46813600	0.73091100
H	5.52670700	-2.32906400	-0.75369400
H	4.05751700	-0.45356400	-1.51764600
H	3.50751200	-1.21988400	-0.03929300
H	5.01908600	1.43866700	-0.20257700
H	4.55039700	0.65589600	1.29396900
H	3.00631700	2.48828200	0.56242700
H	2.61036700	1.57984100	-0.89804900
H	1.96791200	0.66709200	1.98615300
H	0.66221100	0.15031000	-0.72563700
H	-0.05936300	-0.61722000	2.18276800
H	-1.24212400	-0.28473300	0.91756300
H	-0.46256800	-2.25117800	-0.37936400
H	0.79912300	-2.55149100	0.79337500
H	-1.02721500	-4.10076700	1.20621900
H	-0.85488200	-3.01106400	2.56455800

H	-3.20185100	-3.29263900	2.16567400
H	-2.80363400	-1.65704000	1.71303700
H	-2.68176300	-2.49358600	-0.73951900
H	-3.25278600	-4.03671500	-0.15519600
C	-4.83711100	-0.93952500	-0.23389100
C	-4.61183300	-0.50546700	-1.68693400
C	-4.37810300	1.01080300	-1.83600600
C	-3.08503700	1.45873100	-1.21054900
O	-2.98734000	2.29051700	-0.32954100
O	-1.94905200	0.88731200	-1.66296300
H	-2.13783700	0.22289900	-2.34006400
H	-4.34995000	1.28920500	-2.89589600
H	-5.18106000	1.57749600	-1.36503400
H	-3.77211900	-1.06361100	-2.12046500
H	-5.48120300	-0.77631200	-2.29081500
H	-5.82030900	-0.58177700	0.08612800
H	-4.11183400	-0.44443000	0.42290700
H	-5.25022400	-2.95702100	-0.87658500
H	-5.27113800	-2.75452300	0.86023500
N	-0.57693800	2.92608800	0.83866900
C	-0.82877200	3.14888200	2.28961200
H	-1.25860700	2.24206900	2.71156600
H	0.11243400	3.37945300	2.78656700
H	-1.52595200	3.97678200	2.40693200
C	0.01543100	4.09496000	0.13033100
H	-0.67344600	4.93572900	0.19435400
H	0.17529100	3.82600800	-0.91235500
H	0.96522100	4.35381500	0.59527700
H	0.05535700	2.10892300	0.73274600
H	-1.47575200	2.66341600	0.37082900

conf_4

C	-3.12725100	1.57138300	-0.46594500
C	-4.15008000	0.60097900	-1.06394100
C	-4.11753100	-0.79294000	-0.42323700
C	-2.88692700	-1.62927600	-0.79346400
C	-2.87513700	-3.00747600	-0.12733300
C	-1.72513600	-3.91536900	-0.60052900
C	-0.35258200	-3.52491100	-0.12210600
C	0.61466200	-3.02567300	-0.89917700
C	2.03002800	-2.73800000	-0.49238100
C	2.49962300	-1.32529800	-0.87501100
C	3.93015600	-1.01915400	-0.42809800
C	4.37090700	0.40728900	-0.76190900
C	5.79744500	0.72675900	-0.30845700
C	6.22338600	2.15704300	-0.64302500
H	7.24422800	2.35739800	-0.30974300
H	6.18460300	2.33746400	-1.72134600
H	5.56660800	2.88780500	-0.16055600
H	5.87842800	0.56261800	0.77294900
H	6.49057300	0.01834300	-0.77602600
H	4.29091900	0.56518500	-1.84438600
H	3.67410900	1.11958300	-0.30046600

H	4.61612800	-1.73570900	-0.89427400
H	4.02462700	-1.18267600	0.65464600
H	2.42640500	-1.21027700	-1.96206100
H	1.82053200	-0.56888200	-0.46332800
H	2.68689100	-3.46323700	-0.98841900
H	2.15925900	-2.91515200	0.58217600
H	0.38208600	-2.84004100	-1.94748500
H	-0.12209900	-3.75993300	0.92056900
H	-1.72794600	-3.94665900	-1.69453800
H	-1.92467900	-4.93726400	-0.26113800
H	-3.82191100	-3.51074500	-0.34707600
H	-2.84303500	-2.90202600	0.96491600
H	-1.96313300	-1.08822800	-0.55421300
H	-2.86698600	-1.76210200	-1.88159600
H	-5.01193500	-1.34665000	-0.72544700
H	-4.18951100	-0.69222800	0.66872700
H	-3.97732000	0.50724000	-2.14263300
H	-5.15146200	1.02838500	-0.95071800
C	-3.09155100	2.92956300	-1.17361700
C	-2.21467500	3.99325700	-0.50031700
C	-0.71372800	3.64124500	-0.41793400
C	-0.36648900	2.53746900	0.54465100
O	0.30316500	1.56247800	0.26098900
O	-0.82036500	2.64189000	1.80883800
H	-1.36230900	3.43515900	1.92268200
H	-0.33067600	3.34562200	-1.39484900
H	-0.14831900	4.52586300	-0.10083900
H	-2.29363700	4.92821400	-1.05974100
H	-2.61337700	4.22160600	0.49785500
H	-2.75022600	2.78866800	-2.20552600
H	-4.10955000	3.32623400	-1.24227600
H	-3.35966800	1.71644900	0.59759300
H	-2.13685200	1.11107700	-0.51569200
N	0.04610700	-0.77007100	1.66974400
C	1.23414300	-0.96577300	2.54950600
H	1.15601600	-1.92826200	3.05244500
H	2.13310700	-0.93166800	1.93942700
H	1.25520300	-0.16255100	3.28413700
C	-1.24498900	-0.72487500	2.41415700
H	-1.37693100	-1.65755900	2.95979700
H	-2.05523700	-0.59182400	1.70222400
H	-1.21651500	0.11694000	3.10278900
H	0.16818500	0.12916200	1.14625400
H	0.00461800	-1.51722800	0.95737600

conf_32

C	-3.12913400	1.56958500	-0.46579300
C	-4.15161400	0.59849100	-1.06327900
C	-4.11770100	-0.79542200	-0.42260200
C	-2.88655200	-1.63077900	-0.79325500
C	-2.87359200	-3.00908800	-0.12736700
C	-1.72279600	-3.91588500	-0.60072600
C	-0.35059000	-3.52463800	-0.12190300

C	0.61655100	-3.02474300	-0.89868300
C	2.03174300	-2.73657400	-0.49156900
C	2.50129100	-1.32398800	-0.87460800
C	3.93169300	-1.01760600	-0.42738500
C	4.37249100	0.40876200	-0.76150100
C	5.79922100	0.72814000	-0.30856400
C	6.22501800	2.15851300	-0.64299100
H	5.56851900	2.88917300	-0.15998000
H	7.24604700	2.35875500	-0.31020200
H	6.18566800	2.33922100	-1.72124900
H	5.88067600	0.56374900	0.77276900
H	6.49214200	0.01983100	-0.77660200
H	4.29215200	0.56657200	-1.84396500
H	3.67592800	1.12117600	-0.29988100
H	4.61783500	-1.73427800	-0.89313300
H	4.02583800	-1.18077900	0.65544200
H	2.42840200	-1.20939700	-1.96172500
H	1.82201000	-0.56746400	-0.46341000
H	2.68888000	-3.46193300	-0.98708500
H	2.16069300	-2.91328100	0.58310100
H	0.38409000	-2.83904200	-1.94701000
H	-0.12022000	-3.75966400	0.92079600
H	-1.72539000	-3.94676100	-1.69474900
H	-1.92160500	-4.93807400	-0.26177100
H	-3.81990600	-3.51316400	-0.34722900
H	-2.84161000	-2.90379300	0.96490400
H	-1.96307600	-1.08913600	-0.55410700
H	-2.86678000	-1.76339100	-1.88142100
H	-5.01176700	-1.34983600	-0.72452300
H	-4.18938700	-0.69476000	0.66938400
H	-3.97941700	0.50490300	-2.14207200
H	-5.15324800	1.02515500	-0.94944700
C	-3.09490300	2.92797000	-1.17314300
C	-2.21848100	3.99216500	-0.50000600
C	-0.71722900	3.64128200	-0.41843600
C	-0.36869600	2.53743300	0.54360400
O	0.30160600	1.56307700	0.25931900
O	-0.82213300	2.64106300	1.80804200
H	-1.36510700	3.43358400	1.92218000
H	-0.33441600	3.34625600	-1.39562200
H	-0.15235200	4.52621500	-0.10128200
H	-2.29841200	4.92721300	-1.05913800
H	-2.61689900	4.21999600	0.49841400
H	-2.75402500	2.78762400	-2.20527700
H	-4.11323600	3.32389300	-1.24114200
H	-3.36099900	1.71419800	0.59793400
H	-2.13841000	1.11005200	-0.51627800
N	0.04672900	-0.76901000	1.66936000
C	1.23475600	-0.96377000	2.54932200
H	2.13381700	-0.92891900	1.93941400
H	1.25503600	-0.16055600	3.28399100
H	1.15734200	-1.92633700	3.05222900
C	-1.24453600	-0.72462300	2.41352400
H	-1.37606000	-1.65745100	2.95902400
H	-2.05471300	-0.59194700	1.70143800

H	-1.21668200	0.11712700	3.10226000
H	0.16827600	0.13017000	1.14565100
H	0.00584500	-1.51630200	0.95711400

conf_54

C	-3.75851300	0.73903600	-0.30129800
C	-4.54070200	-0.37890100	-1.00135600
C	-4.43663900	-1.75603200	-0.32006700
C	-3.27851200	-2.64597700	-0.79350000
C	-1.87352900	-2.07937500	-0.57288700
C	-0.76408600	-3.08945500	-0.90105700
C	0.61396600	-2.50091400	-0.82206300
C	1.64585800	-3.01927900	-0.14562800
C	3.05438400	-2.48215000	-0.12440000
C	3.27085800	-1.14314500	-0.83090400
C	4.67885800	-0.57833600	-0.62265900
C	4.88571100	0.81106400	-1.23771500
C	4.08499400	1.92773200	-0.55735800
C	4.36078700	3.30657400	-1.16037600
H	5.41989200	3.56629300	-1.07956300
H	4.09538300	3.33012200	-2.22127700
H	3.78731700	4.08810100	-0.65524000
H	3.01110400	1.71805400	-0.62420900
H	4.33568300	1.94268500	0.51194400
H	5.94987200	1.06451700	-1.18853400
H	4.63079300	0.77927400	-2.30405900
H	5.40712900	-1.27710800	-1.04707700
H	4.90125000	-0.53160800	0.45232100
H	3.08471400	-1.25946800	-1.90382000
H	2.52763900	-0.41510500	-0.48619900
H	3.71431700	-3.23935300	-0.56613100
H	3.39530800	-2.40906000	0.91852300
H	1.48754400	-3.94603200	0.40603500
H	0.76606400	-1.59470000	-1.40597800
H	-0.92434800	-3.45258600	-1.92458300
H	-0.84178900	-3.96506900	-0.24853400
H	-1.77520500	-1.75477000	0.47114800
H	-1.72647000	-1.18447100	-1.18629100
H	-3.40619700	-2.86059600	-1.86111700
H	-3.35329100	-3.61171800	-0.28066800
H	-5.36513100	-2.30668500	-0.49456600
H	-4.37281900	-1.61879400	0.76725900
H	-4.20857800	-0.46028800	-2.04366100
H	-5.59237000	-0.07840600	-1.04579000
C	-3.86966100	2.08329700	-1.02486700
C	-3.15752500	3.25497300	-0.33874300
C	-1.62159900	3.14115000	-0.30401400
C	-1.03958300	2.10912600	0.62494300
O	-0.11619000	1.37290300	0.33031200
O	-1.53267500	2.03108500	1.87448200
H	-2.29082700	2.62177200	1.98752900
H	-1.22655200	2.93054200	-1.29836700
H	-1.19083900	4.09961700	0.01253800

H	-3.39513700	4.17960400	-0.86995300
H	-3.56414700	3.39897200	0.67293000
H	-3.47703300	1.98130200	-2.04353900
H	-4.92741700	2.34304300	-1.13352600
H	-4.13580500	0.84304800	0.72438200
H	-2.70972300	0.44457800	-0.21590100
N	0.84733900	-0.63196000	1.92158800
C	2.14180400	-0.16345700	2.49389200
H	2.78472600	0.17126700	1.68371500
H	1.94239800	0.66485600	3.17210200
H	2.61373900	-0.98106400	3.03602300
C	-0.13594700	-1.07581400	2.94872800
H	-0.37753300	-0.23089900	3.59072000
H	0.29750100	-1.88407200	3.53581500
H	-1.03606500	-1.42271800	2.44585600
H	0.43336400	0.14715000	1.35450300
H	1.00554200	-1.39912200	1.24376000

conf_10

C	4.44512900	0.58743400	1.07503200
C	3.96769900	1.99178900	1.46959100
C	3.13246500	2.72303900	0.40965400
C	1.86334400	1.98272800	-0.02545600
C	0.97385300	2.82558700	-0.94167800
C	-0.27342600	2.09646000	-1.46047300
C	-1.20953900	1.63800200	-0.37487300
C	-2.01064600	0.56734800	-0.43087700
C	-3.04280300	0.20520900	0.60015300
C	-4.47859000	0.35820600	0.06926300
C	-5.53355200	-0.05741300	1.09830200
C	-6.97922600	0.16082400	0.63598200
C	-7.40135500	-0.69675400	-0.56149800
C	-8.87707100	-0.51769200	-0.92303000
H	-9.52379700	-0.79464400	-0.08534800
H	-9.09547600	0.52334700	-1.17903100
H	-9.15701000	-1.13643500	-1.77905800
H	-6.78604400	-0.45086100	-1.43375300
H	-7.20449500	-1.75179500	-0.33315900
H	-7.65014600	-0.05249500	1.47552700
H	-7.12592000	1.22075300	0.39335200
H	-5.36615500	0.50685000	2.02324100
H	-5.39206300	-1.11574100	1.35422500
H	-4.63654800	1.40247100	-0.22205900
H	-4.58646600	-0.23648500	-0.84356900
H	-2.91093700	0.82960000	1.49013600
H	-2.91318400	-0.83703400	0.92445700
H	-1.99486900	-0.04681100	-1.33421600
H	-1.27527600	2.28157300	0.50332900
H	0.01393800	1.24840300	-2.09298900
H	-0.82498200	2.78431500	-2.11321000
H	1.56151600	3.16725800	-1.79952200
H	0.66218000	3.73042200	-0.40716300
H	1.29265100	1.69953800	0.87015500

H	2.12899600	1.05391300	-0.54273200
H	3.74359800	2.93172400	-0.47497500
H	2.85052400	3.70193700	0.81146200
H	4.83879400	2.60622500	1.72050900
H	3.37971300	1.91399500	2.39197700
C	5.38472600	0.56647500	-0.13710900
C	5.87416800	-0.83188700	-0.54271600
C	4.88390100	-1.62905000	-1.42555600
C	3.55872900	-1.89268900	-0.76918600
O	2.50468000	-1.40780700	-1.13300400
O	3.54056200	-2.70531700	0.30781600
H	4.42868500	-3.01697000	0.53030000
H	4.67982700	-1.08146700	-2.34537800
H	5.33035500	-2.59102100	-1.70061700
H	6.79490700	-0.74519000	-1.12384200
H	6.14097900	-1.40766500	0.35229200
H	4.90323800	1.03693200	-1.00072100
H	6.25700200	1.18490000	0.09573300
H	4.96711800	0.14040400	1.92883000
H	3.57184000	-0.04449100	0.89271500
N	0.28562600	-1.33480800	0.48480800
C	0.72369900	-1.22326400	1.90423600
H	1.26262600	-0.28658400	2.02944700
H	-0.15191800	-1.23931400	2.55120300
H	1.38025400	-2.06049200	2.13355600
C	-0.47401200	-2.58112900	0.18335200
H	0.17708700	-3.43637400	0.35645800
H	-0.78353600	-2.55866800	-0.85987800
H	-1.34754200	-2.63727100	0.82979200
H	-0.29292700	-0.50970200	0.23702800
H	1.12718400	-1.31773700	-0.13534200

conf_79

C	4.12866300	-0.07853700	-0.73734200
C	4.39479700	1.35543000	-1.20438500
C	3.63227600	2.41369400	-0.39616600
C	2.12084600	2.43368000	-0.65287300
C	1.38745000	3.50988400	0.15123800
C	-0.11331200	3.61135900	-0.18174900
C	-0.97405200	2.50673700	0.36911500
C	-1.51401600	1.51817700	-0.35262800
C	-2.49841000	0.49822500	0.14172100
C	-3.86309700	0.61724000	-0.55908400
C	-4.86505000	-0.43789200	-0.08703500
C	-6.22683400	-0.32663900	-0.77581800
C	-7.23301000	-1.37944200	-0.30402600
C	-8.59038100	-1.26072700	-0.99895400
H	-9.28815700	-2.02271800	-0.64365300
H	-9.04352000	-0.28226100	-0.81392800
H	-8.49039200	-1.37990600	-2.08192000
H	-6.81717500	-2.37894500	-0.47883000
H	-7.36674300	-1.28916500	0.78058300
H	-6.64084300	0.67396600	-0.60062300

H	-6.09219900	-0.41511000	-1.86095900
H	-5.00137300	-0.34975600	0.99856400
H	-4.44991500	-1.43835500	-0.26497100
H	-4.26501600	1.62004200	-0.38041400
H	-3.71896400	0.52987100	-1.64186400
H	-2.64466500	0.60819500	1.22295200
H	-2.11440200	-0.51592400	-0.04011300
H	-1.28730100	1.47522700	-1.41745700
H	-1.22824900	2.58039700	1.42936900
H	-0.23121900	3.65220000	-1.26905600
H	-0.49518500	4.56071200	0.20747400
H	1.85273100	4.47871200	-0.05523600
H	1.52112000	3.33946500	1.22747100
H	1.67868000	1.45005500	-0.45211900
H	1.94883700	2.61308800	-1.72075000
H	4.03424200	3.40328500	-0.63518900
H	3.83075000	2.26669100	0.67464700
H	4.12773300	1.44928000	-2.26366600
H	5.46916400	1.55546000	-1.14106500
C	4.79741100	-1.13887100	-1.61731600
C	4.69246100	-2.57806700	-1.09653900
C	3.25180600	-3.11730500	-0.96684500
C	2.43616900	-2.51225100	0.14437800
O	1.31832400	-2.05278300	0.00789200
O	2.97801100	-2.49017700	1.37830100
H	3.87588700	-2.85040000	1.37301000
H	2.69547500	-2.96440300	-1.89186600
H	3.28182500	-4.19778600	-0.78097200
H	5.22423300	-3.24508500	-1.77889200
H	5.22544200	-2.66393300	-0.13929800
H	4.36622400	-1.09366600	-2.62391600
H	5.85899400	-0.89691500	-1.73072400
H	4.47985100	-0.18321300	0.29800500
H	3.04864400	-0.24787300	-0.72592900
N	0.38479700	-0.19766100	1.79236000
C	-0.56169200	-0.82631000	2.75810900
H	-0.01751400	-1.56712700	3.34166500
H	-0.96792200	-0.05969800	3.41600800
H	-1.36449900	-1.30641900	2.20436300
C	1.56363600	0.43907000	2.44672300
H	2.19431400	0.88025800	1.67960800
H	2.11703600	-0.32824800	2.98385100
H	1.21829700	1.20974800	3.13379900
H	0.72104800	-0.93468700	1.12754400
H	-0.10349500	0.50752700	1.21528400

conf_62

C	-4.28912600	-1.03944600	-0.29994000
C	-3.86026300	-2.31302300	-1.04150800
C	-2.50039600	-2.88957300	-0.62430300
C	-1.29556200	-2.01444200	-0.98891200
C	0.03802400	-2.68811400	-0.65749500
C	1.27930500	-1.97004500	-1.20269200

C	1.58738700	-0.63530100	-0.58211800
C	2.18551300	0.38565200	-1.20884800
C	2.73306500	1.61694400	-0.53757900
C	4.27103300	1.57292200	-0.40622100
C	4.77534200	0.47797000	0.53393100
C	6.30196300	0.45782600	0.65439000
C	6.83850400	-0.58426400	1.64412100
C	6.54531400	-2.03383500	1.24703200
H	5.47281300	-2.24701800	1.24088700
H	6.93453000	-2.25243700	0.24745000
H	7.01216300	-2.73366100	1.94424400
H	7.92104100	-0.45143900	1.73647000
H	6.42227400	-0.38437900	2.63885300
H	6.73833600	0.27329600	-0.33548800
H	6.64824600	1.45164900	0.96060800
H	4.41845900	-0.49439900	0.18282400
H	4.33622500	0.62598700	1.52960200
H	4.71136200	1.43741600	-1.40098900
H	4.61400700	2.54974500	-0.04930400
H	2.29309200	1.72697000	0.46104300
H	2.47212100	2.51247800	-1.11406900
H	2.41965100	0.27680700	-2.26894800
H	1.39957700	-0.54850200	0.48924900
H	1.21443000	-1.87033700	-2.29201700
H	2.14691600	-2.61567200	-1.01378200
H	0.02849900	-3.70427700	-1.06399000
H	0.13053100	-2.79798700	0.42943200
H	-1.35736600	-1.05298200	-0.46495400
H	-1.32854000	-1.80022200	-2.06636000
H	-2.37701700	-3.86229300	-1.11151600
H	-2.48691900	-3.09398400	0.45183400
H	-3.83942900	-2.10739400	-2.11808800
H	-4.63073400	-3.07787100	-0.89710000
C	-4.60856200	-1.27349400	1.18247200
C	-5.07099300	-0.02555200	1.94803300
C	-3.92431400	0.89269100	2.43224700
C	-3.08567900	1.45735400	1.32218100
O	-1.91377000	1.18868300	1.14093400
O	-3.67616800	2.30994100	0.45812000
H	-4.61182800	2.43526700	0.66854400
H	-4.33986500	1.72812200	3.00671800
H	-3.25294100	0.33889900	3.08807000
H	-5.78753500	0.54081500	1.33979700
H	-5.61795000	-0.32640400	2.84433400
H	-5.39978600	-2.02701100	1.24354000
H	-3.74225000	-1.70312700	1.69618500
H	-3.50930000	-0.28135800	-0.41286800
H	-5.18223500	-0.63093300	-0.78667800
N	-0.66379300	1.84925700	-1.20352400
C	-1.52893800	1.50502500	-2.36676300
H	-1.02476700	1.79512800	-3.28735700
H	-1.70935500	0.43289600	-2.36216900
H	-2.47106600	2.04154000	-2.26970100
C	-0.32512500	3.29809300	-1.11802700
H	0.21709100	3.59701200	-2.01344600

H	0.29021800	3.46344900	-0.23666900
H	-1.25140900	3.86458100	-1.03525000
H	-1.15158500	1.58060200	-0.31667400
H	0.19740000	1.27292100	-1.23002600

conf_64

C	-4.32194900	0.05313000	-0.56253600
C	-3.06742200	-0.62809700	-1.12335200
C	-2.68968600	-1.91966800	-0.39469600
C	-1.34220500	-2.47813600	-0.85680700
C	-0.90489400	-3.75586600	-0.13795400
C	0.49473000	-4.24162900	-0.56572200
C	1.61992000	-3.32652800	-0.15887600
C	2.24274700	-2.48112300	-0.98607400
C	3.38573300	-1.56906200	-0.65384500
C	3.10714500	-0.11009900	-1.05219900
C	4.25075500	0.84547000	-0.70081300
C	3.94244900	2.31810700	-0.99818000
C	2.86993500	2.93392000	-0.09189000
C	2.61227200	4.41059100	-0.39858400
H	3.52359400	5.00335400	-0.28101700
H	2.26444600	4.54162000	-1.42748000
H	1.85435700	4.83236600	0.26702000
H	1.92895400	2.38216500	-0.18848200
H	3.18930000	2.83234200	0.95409000
H	4.86419700	2.90043400	-0.89631100
H	3.63425700	2.41938600	-2.04599700
H	5.14391800	0.53964700	-1.25540300
H	4.51289900	0.73926600	0.36111600
H	2.92111800	-0.06505400	-2.13102000
H	2.17341700	0.22907200	-0.58954000
H	4.28037700	-1.90971200	-1.18914800
H	3.63350000	-1.63970800	0.41237000
H	1.90631400	-2.44455800	-2.02198800
H	1.97056800	-3.41381600	0.87276400
H	0.50697300	-4.36203500	-1.65341500
H	0.67178700	-5.23376700	-0.13948000
H	-1.62833300	-4.55112000	-0.34232800
H	-0.92562000	-3.60992800	0.94942700
H	-0.57702300	-1.70100300	-0.73835800
H	-1.38407400	-2.67685100	-1.93415600
H	-3.47479600	-2.67089500	-0.53470500
H	-2.65822000	-1.72211700	0.68541000
H	-2.21027500	0.05007900	-1.09446700
H	-3.22688300	-0.84124900	-2.18623200
C	-4.55469200	1.48435200	-1.07812600
C	-3.88807800	2.62167600	-0.28684600
C	-2.34984900	2.67597600	-0.28684800
C	-1.59852300	1.71598200	0.60022000
O	-0.47481700	1.31774200	0.35153900
O	-2.16788300	1.32154600	1.74987100
H	-3.06758600	1.67109200	1.82618400
H	-1.94840800	2.55346700	-1.29373200

H	-2.02487200	3.66938700	0.04955900
H	-4.23059300	3.57085400	-0.70656300
H	-4.27761100	2.62874000	0.74291500
H	-4.23919300	1.54897900	-2.12580800
H	-5.62781000	1.69357300	-1.07726400
H	-5.19068200	-0.56049300	-0.81761000
H	-4.28488300	0.05448800	0.53451600
N	0.81852300	-0.67160600	1.70820400
C	2.02266200	-0.10791700	2.38412400
H	2.64767100	0.38169200	1.64312800
H	1.69339500	0.61860800	3.12504800
H	2.57127400	-0.91248000	2.87108100
C	-0.13629900	-1.32274300	2.64976100
H	-0.99036900	-1.68683700	2.08582500
H	-0.46574800	-0.58223700	3.37569000
H	0.36234400	-2.14965800	3.15267600
H	0.31863300	0.10053200	1.20330500
H	1.10312000	-1.35070400	0.98513500

conf_360

C	-1.87363000	2.91744000	-1.08436900
C	-0.70378100	2.34675800	-0.27752900
C	0.61659000	3.10493600	-0.42342600
C	1.74674200	2.39395100	0.32277600
C	3.14757400	2.96187800	0.09629600
C	4.24911100	2.03122100	0.64403300
C	4.33514100	0.74517500	-0.12544100
C	4.09451200	-0.47748400	0.34686800
C	4.16859400	-1.74226900	-0.46169100
C	2.81874100	-2.47022800	-0.60373400
C	1.74103200	-1.63429700	-1.30015700
C	0.45406200	-2.40547900	-1.61780400
C	-0.69180000	-1.50723500	-2.09227700
C	-1.97879700	-2.27428600	-2.39652600
H	-1.81700100	-3.02260300	-3.17670900
H	-2.35097000	-2.79371800	-1.50814500
H	-2.76717400	-1.60178300	-2.74494100
H	-0.89417000	-0.74263100	-1.33192400
H	-0.36733300	-0.95804300	-2.98244400
H	0.66862700	-3.16224500	-2.37947600
H	0.12318000	-2.98627500	-0.74265400
H	2.14730500	-1.22617900	-2.23180800
H	1.51779000	-0.74237400	-0.69669500
H	2.97347500	-3.39869400	-1.16268000
H	2.46934900	-2.78233200	0.39274300
H	4.88705500	-2.43110600	-0.00326200
H	4.55227600	-1.51108100	-1.46044500
H	3.83468400	-0.59036100	1.40122900
H	4.60554500	0.84644500	-1.17688400
H	4.06776200	1.82380100	1.70483700
H	5.21197100	2.55019700	0.58548300
H	3.23391000	3.94684400	0.56502300
H	3.31045500	3.11438600	-0.97699100

H	1.75184300	1.34202900	0.01312400
H	1.53257100	2.40238500	1.40062500
H	0.50761800	4.13327000	-0.06121800
H	0.87660300	3.17779400	-1.48600200
H	-0.54078600	1.31121900	-0.60286900
H	-0.97152900	2.29026600	0.78387000
C	-3.10322700	1.99958800	-1.06303900
C	-3.82508800	1.95283800	0.29543300
C	-4.53992400	0.62144400	0.53771900
C	-3.57035200	-0.50790700	0.79767800
O	-2.43868500	-0.33747000	1.21037700
O	-3.99100600	-1.76397600	0.59345100
H	-4.89387800	-1.78301000	0.24761200
H	-5.18676400	0.67742800	1.42275900
H	-5.19018300	0.36044300	-0.30479100
H	-3.11771200	2.10832400	1.11170100
H	-4.55691700	2.76140700	0.35722000
H	-3.81441100	2.30831500	-1.83513700
H	-2.77431100	0.99059000	-1.34223800
H	-2.14073700	3.91302400	-0.71151100
H	-1.55412600	3.05525800	-2.12254200
N	-0.04521800	-1.52865800	1.62519500
C	0.66397800	-0.56931400	2.52270600
H	1.65567800	-0.95735100	2.74758000
H	0.74547200	0.38701400	2.01230400
H	0.08459000	-0.45892600	3.43738800
C	-0.21113400	-2.89563000	2.19959400
H	-0.78042400	-2.81534900	3.12388300
H	0.76942100	-3.32433300	2.39867400
H	-0.75307200	-3.51255200	1.48548400
H	-0.99255700	-1.14208700	1.40191200
H	0.46086600	-1.59184800	0.73825500

conf_311

C	0.32047700	3.49963300	0.86709300
C	-0.58425300	2.54041400	0.08666400
C	-2.08038100	2.70587800	0.36328400
C	-2.92198200	1.72654400	-0.45647200
C	-4.40424400	1.69373500	-0.08112800
C	-5.16564600	0.54392800	-0.77527100
C	-4.71640200	-0.80205900	-0.28338000
C	-3.95266600	-1.65479100	-0.96474900
C	-3.40392600	-2.94943300	-0.43665800
C	-1.88475700	-2.87244400	-0.17585800
C	-1.52581900	-1.97763100	1.01233000
C	-0.02257000	-1.74828900	1.17707900
C	0.34810000	-0.87569300	2.37902400
C	1.85698300	-0.70577700	2.56322500
H	2.34449000	-1.67175900	2.73985200
H	2.32086600	-0.24252600	1.68625000
H	2.09030800	-0.07244300	3.42146500
H	-0.12243100	0.10645100	2.26311400
H	-0.08179700	-1.31373100	3.28510700

H	0.47017300	-2.73019500	1.28119300
H	0.35849100	-1.27621000	0.25869800
H	-1.92452100	-2.42097600	1.93172700
H	-2.02118900	-1.00879600	0.90106700
H	-1.49640200	-3.88305800	-0.00199800
H	-1.38870700	-2.49726800	-1.08153900
H	-3.59800000	-3.75366400	-1.15427500
H	-3.91659400	-3.22346700	0.49125700
H	-3.65547400	-1.39181200	-1.98047200
H	-5.00945800	-1.06065900	0.73442500
H	-5.02022900	0.61241200	-1.85871700
H	-6.23769300	0.66600100	-0.59009700
H	-4.87502400	2.64781700	-0.33936900
H	-4.50387400	1.58397900	1.00559000
H	-2.50657400	0.72063600	-0.33433900
H	-2.82536200	1.96454700	-1.52356900
H	-2.38581600	3.73748600	0.15547700
H	-2.26374900	2.54057800	1.43174600
H	-0.30288800	1.50725800	0.33331100
H	-0.42430600	2.66093000	-0.99421400
C	1.80829000	3.14272000	0.75546800
C	2.39594100	3.33539800	-0.65564500
C	3.56748500	2.39471200	-0.95838700
C	3.21848700	0.92738100	-0.94812300
O	4.00331400	0.04590200	-0.64852000
O	1.97731400	0.55535800	-1.31404500
H	1.39876300	1.32044600	-1.45371400
H	3.98526100	2.60052400	-1.95170500
H	4.38467600	2.52400000	-0.24671800
H	1.61917100	3.21820400	-1.42219800
H	2.74056200	4.36431400	-0.77893900
H	2.39270900	3.73490600	1.46481600
H	1.92921600	2.09887100	1.07214700
H	0.15305700	4.52873300	0.53009200
H	0.03430500	3.47453800	1.92335300
N	2.92940000	-2.44969900	-0.37695400
C	2.42226700	-2.90973100	-1.70262800
H	1.90814400	-3.86189000	-1.58234400
H	1.73775100	-2.15629000	-2.08695400
H	3.26781300	-3.02321400	-2.37882500
C	3.91324600	-3.36881000	0.26498700
H	4.77874300	-3.46401900	-0.38822700
H	3.45184800	-4.34326900	0.41798700
H	4.21762700	-2.94247000	1.21906200
H	3.36420600	-1.49817800	-0.49327400
H	2.12930500	-2.31304600	0.24781900

conf_372

C	4.15392400	-0.68160200	0.96014200
C	5.04671900	0.32036000	0.20991900
C	4.28714300	1.14535500	-0.84131900
C	3.53774100	2.34271800	-0.24154200
C	2.55778800	3.00234800	-1.21986000

C	1.21309800	2.24969800	-1.33606100
C	0.36826600	2.42278100	-0.10668400
C	0.10216900	1.47923200	0.79721600
C	-0.66569800	1.67189600	2.07404100
C	-1.95359200	0.83135000	2.14950400
C	-3.03763500	1.30273300	1.17829400
C	-4.26637900	0.38758700	1.13890400
C	-5.28305500	0.75828800	0.05398800
C	-6.48491200	-0.18565000	0.00476900
H	-6.17545500	-1.21820300	-0.18855200
H	-7.02343500	-0.18048700	0.95579900
H	-7.19009800	0.10397400	-0.77723100
H	-5.62359400	1.78521900	0.22037300
H	-4.79255800	0.77937800	-0.93233700
H	-3.95227700	-0.66108700	1.01384500
H	-4.76081400	0.40616400	2.11550100
H	-2.60096700	1.40965100	0.17430000
H	-3.35438000	2.31662600	1.44579200
H	-1.70982200	-0.21993000	1.95274800
H	-2.34698700	0.86677200	3.17009300
H	-0.90754700	2.73187000	2.20151500
H	-0.02219100	1.39700400	2.91798600
H	0.48610100	0.47511500	0.62086100
H	0.00106700	3.43264300	0.07500100
H	1.39763400	1.18499400	-1.51870500
H	0.67383500	2.63526800	-2.20964400
H	3.01896700	3.07101600	-2.21068400
H	2.35265500	4.03042600	-0.90570400
H	4.27703600	3.07901000	0.08837700
H	2.99385100	2.03904500	0.65992200
H	3.58575400	0.49858900	-1.38442300
H	4.98683300	1.51516300	-1.59704900
H	5.87052500	-0.22522100	-0.26212000
H	5.50978700	1.00462100	0.92735400
C	3.89176200	-1.95269900	0.14569000
C	2.95009800	-2.97787100	0.80392900
C	1.55501000	-2.49008600	1.23039600
C	0.55626500	-2.15004200	0.15430700
O	-0.64607200	-2.13199900	0.35355500
O	0.99562700	-1.81005900	-1.06581000
H	1.96550000	-1.83365000	-1.10511900
H	1.06651700	-3.23443300	1.86184700
H	1.63280200	-1.58875400	1.84989200
H	2.84688600	-3.85478100	0.15631300
H	3.43874500	-3.33866800	1.71280100
H	3.56673500	-1.66806700	-0.86906600
H	4.84543400	-2.45822300	-0.03381900
H	4.62342000	-0.96998700	1.90625700
H	3.21151100	-0.18893300	1.22515800
N	-2.39051100	-0.91590800	-1.34360000
C	-1.66624800	-0.06121700	-2.32959900
H	-2.39001200	0.41801000	-2.98710100
H	-1.09798200	0.68687200	-1.78047500
H	-0.99079400	-0.69217200	-2.90332300
C	-3.26580200	-1.95856500	-1.95151600

H	-4.02792700	-1.47846200	-2.56303600
H	-3.73333900	-2.53134600	-1.15274400
H	-2.65101500	-2.61424700	-2.56554300
H	-1.69017700	-1.38532100	-0.71390300
H	-2.95306700	-0.31731900	-0.73504000

conf_351

C	-4.60582600	0.99126500	-0.74731100
C	-4.95666400	-0.40250700	-1.28411200
C	-4.13687100	-1.55394600	-0.68700500
C	-4.27819500	-1.69568200	0.83297500
C	-3.53579900	-2.89849700	1.43437300
C	-2.03749900	-2.66885900	1.70948900
C	-1.20009700	-2.40815900	0.49323300
C	-0.36904000	-1.37953600	0.32816800
C	0.50942200	-1.16864300	-0.87356200
C	1.96212300	-1.61617300	-0.63263200
C	2.91863500	-1.20580400	-1.75663300
C	4.36934900	-1.66121100	-1.55606200
C	5.10539500	-0.95440600	-0.41259300
C	6.55597700	-1.41445800	-0.25172600
H	6.60077400	-2.48503200	-0.03645700
H	7.12415800	-1.23567000	-1.16809200
H	7.05994300	-0.88882400	0.56306300
H	5.09833900	0.12912600	-0.60683900
H	4.57714700	-1.12772200	0.53547900
H	4.38905700	-2.74271200	-1.38022700
H	4.92881100	-1.49289300	-2.48188100
H	2.89022400	-0.11373500	-1.89121100
H	2.54095100	-1.61916500	-2.69715400
H	2.30130300	-1.22917100	0.33748000
H	1.98136500	-2.70419300	-0.51327200
H	0.50462100	-0.11019300	-1.16030400
H	0.10914500	-1.72128500	-1.72910500
H	-0.28665900	-0.64367900	1.12888500
H	-1.28582500	-3.14042500	-0.30995300
H	-1.64880500	-3.56436400	2.21104300
H	-1.91921600	-1.84103300	2.41863600
H	-3.65666600	-3.76859400	0.77845600
H	-4.00421100	-3.16983800	2.38502600
H	-5.34425400	-1.78050800	1.07189500
H	-3.93105100	-0.78301200	1.33368500
H	-3.08376200	-1.43661800	-0.95478200
H	-4.46312000	-2.48903900	-1.15532600
H	-4.83027400	-0.40459400	-2.37275400
H	-6.02094100	-0.58589600	-1.10085200
C	-3.14297200	1.39237800	-0.95786200
C	-2.86907800	2.82962000	-0.50594100
C	-1.42745800	3.29602800	-0.74052500
C	-0.35975500	2.58876600	0.05244800
O	0.78172300	2.43979700	-0.34794700
O	-0.66108800	2.14340000	1.28129500
H	-1.59979800	2.27943100	1.47763800

H	-1.14795600	3.20465700	-1.79129700
H	-1.32462700	4.35871000	-0.48539600
H	-3.52836700	3.51423900	-1.04627200
H	-3.15363600	2.95601000	0.55021700
H	-2.48437700	0.69512700	-0.42653200
H	-2.88735800	1.30021200	-2.02027700
H	-5.24877900	1.72858100	-1.24065000
H	-4.84583300	1.05067900	0.32040300
N	2.91218300	1.37749100	0.93925900
C	2.55746000	0.91682900	2.31341500
H	2.14711100	1.75959600	2.86622200
H	1.80976600	0.13028200	2.23545200
H	3.45116200	0.54033200	2.80842200
C	3.94047100	2.45788500	0.90352000
H	4.11866200	2.73640300	-0.13339600
H	4.86049700	2.09523600	1.35875800
H	3.56183700	3.31625100	1.45551100
H	3.24992600	0.57923700	0.39487500
H	2.04288800	1.72674500	0.45933400

conf_322

C	-1.14479200	2.22362000	-0.86271000
C	-2.60695700	1.81336500	-1.04515900
C	-2.99447800	0.63215900	-0.15336100
C	-4.43273800	0.15120400	-0.36369300
C	-4.86899300	-1.01327700	0.53697100
C	-4.31624500	-2.39921000	0.14807300
C	-2.85924600	-2.62173500	0.43417200
C	-1.94468900	-3.01076000	-0.45282200
C	-0.49391100	-3.25290900	-0.15373400
C	0.44123300	-2.29431700	-0.90786300
C	1.92698100	-2.56421500	-0.65142000
C	2.85960800	-1.50897700	-1.25594900
C	4.33003900	-1.65691000	-0.84459900
C	4.98176700	-2.96135500	-1.30942000
H	4.51404000	-3.83806000	-0.85442600
H	4.90481100	-3.06861700	-2.39539900
H	6.04209900	-2.98438300	-1.04886500
H	4.89201300	-0.80835500	-1.24674100
H	4.41064100	-1.58121700	0.24922900
H	2.78179000	-1.55611500	-2.34798500
H	2.51006900	-0.50512000	-0.98234100
H	2.17908700	-3.55595200	-1.03677400
H	2.11802300	-2.65446100	0.43442500
H	0.24790200	-2.36542000	-1.98315400
H	0.18812800	-1.25754000	-0.64463300
H	-0.22675100	-4.28224400	-0.42240100
H	-0.32398200	-3.16910300	0.92778200
H	-2.24850900	-3.16110100	-1.48813700
H	-2.55409300	-2.47694100	1.47241800
H	-4.51384600	-2.58581100	-0.91282300
H	-4.88751800	-3.15258000	0.70455500
H	-5.96067500	-1.07675100	0.51137700

H	-4.60594100	-0.79249500	1.57960600
H	-4.56636800	-0.13830700	-1.41319100
H	-5.10956000	0.99654700	-0.19587400
H	-2.86820100	0.91817000	0.90063200
H	-2.30474700	-0.19589600	-0.33904900
H	-2.77769700	1.54851800	-2.09501500
H	-3.26095400	2.66785100	-0.83477300
C	-0.72686600	3.42485000	-1.71572300
C	0.76987400	3.75889000	-1.67672500
C	1.29371100	4.15293200	-0.28521600
C	1.46053000	2.98075600	0.64515200
O	1.87169700	1.89136400	0.29550700
O	1.16021800	3.14932900	1.94931300
H	0.83785400	4.04408700	2.12303200
H	2.29159200	4.59819700	-0.37864700
H	0.65077500	4.91357400	0.16983100
H	1.35766100	2.91190300	-2.04059000
H	0.96845300	4.59413500	-2.35216300
H	-1.00077000	3.23062300	-2.75758900
H	-1.30555000	4.30565700	-1.41162100
H	-0.98477300	2.44329200	0.20171000
H	-0.49876900	1.37025900	-1.10229800
N	1.41790700	-0.32677200	1.79660500
C	2.43688000	-0.40300300	2.88349500
H	2.35927600	0.49570500	3.49264300
H	2.25156700	-1.28690600	3.49176500
H	3.42516700	-0.45940700	2.43053500
C	0.00746700	-0.25311800	2.27939400
H	-0.20808100	-1.12599800	2.89311200
H	-0.65864000	-0.22954700	1.41972100
H	-0.10626000	0.65706100	2.86504900
H	1.62103100	0.52196700	1.21153900
H	1.52398000	-1.13530800	1.17754900

conf_427

C	-2.92215600	2.15478100	-0.13265000
C	-4.39511500	1.77717500	-0.32744300
C	-4.79369000	0.47743800	0.38195300
C	-4.05321200	-0.76645700	-0.11620300
C	-4.49171800	-2.05026100	0.58958000
C	-3.72800500	-3.29758500	0.11090400
C	-2.25956300	-3.25284800	0.42148900
C	-1.27889600	-3.31447200	-0.47813600
C	0.19032600	-3.30338100	-0.17030800
C	0.94324100	-2.15867800	-0.86512700
C	2.45843500	-2.22369600	-0.66452300
C	3.22027200	-1.10418600	-1.37950300
C	4.73070900	-1.11126600	-1.11853300
C	5.45222200	0.09025600	-1.73008400
H	5.05489600	1.03488200	-1.34318400
H	6.52444800	0.06319200	-1.52402900
H	5.32417700	0.10862100	-2.81536900
H	4.93373100	-1.14896600	-0.03526200

H	5.15797100	-2.04115000	-1.50657300
H	3.04958300	-1.18962000	-2.45780700
H	2.80009200	-0.12688800	-1.10533400
H	2.83227500	-3.19284100	-1.01220900
H	2.67873700	-2.21196600	0.41596700
H	0.72554600	-2.18374700	-1.93907200
H	0.55850600	-1.19836900	-0.49874100
H	0.63356800	-4.25389000	-0.49477500
H	0.34099700	-3.24748200	0.91425700
H	-1.54133500	-3.39874300	-1.53270000
H	-1.99849700	-3.17575700	1.47823000
H	-3.87154000	-3.42629400	-0.96720400
H	-4.16634100	-4.17949800	0.59255300
H	-5.56369800	-2.20719100	0.43137900
H	-4.35546500	-1.93533800	1.67221800
H	-2.97266300	-0.64930200	0.02041300
H	-4.21087400	-0.87527900	-1.19720400
H	-5.87093500	0.32646400	0.25767900
H	-4.62760900	0.59120500	1.46100600
H	-4.60297900	1.69267100	-1.40034600
H	-5.01730300	2.59771600	0.04439200
C	-2.53940000	3.50889900	-0.73226300
C	-1.09583300	3.95159700	-0.45923000
C	0.00212500	3.03623300	-1.07450800
C	0.72384800	2.16141900	-0.08639500
O	1.93751800	2.15035800	0.04272600
O	0.00786000	1.34239700	0.69807600
H	-0.94597200	1.41436300	0.52159300
H	-0.43855000	2.38014400	-1.83496300
H	0.77127700	3.62470500	-1.57114400
H	-0.95389200	4.95156100	-0.87241200
H	-0.93827300	4.05286800	0.62013600
H	-2.71100800	3.48633900	-1.81439800
H	-3.21102900	4.27454400	-0.33219300
H	-2.71259300	2.16213800	0.94852700
H	-2.31656200	1.36241600	-0.59730500
N	3.19530000	0.36730200	1.64828200
C	4.43335900	1.01762000	2.16786800
H	5.03163200	1.35098000	1.32192600
H	4.14740600	1.87243000	2.77806700
H	4.99496800	0.30210800	2.76657300
C	2.27622400	-0.15355000	2.70137000
H	2.79221000	-0.91176400	3.28831700
H	1.39923500	-0.57599100	2.21580000
H	1.97591000	0.67460000	3.34065200
H	2.67280700	1.05781100	1.04485600
H	3.45798800	-0.39904600	1.02375600

11Z_NMe2H2

conf_5

C	2.84533000	-2.22802500	-1.92887500
C	3.52761100	-1.76409200	-0.63449400
C	3.78556000	-0.24937800	-0.60005100

C	3.92337500	0.29896000	0.82076700
C	2.61310100	0.28287500	1.56836500
O	1.52989800	0.15657000	1.03303300
O	2.64478700	0.44801300	2.90436800
H	3.55216600	0.52601600	3.23013800
H	4.67183500	-0.26028100	1.39441100
H	4.25993900	1.34390500	0.81001000
H	2.97320000	0.28764300	-1.09261000
H	4.69709200	-0.01559100	-1.15545100
H	2.90699500	-2.05528400	0.21948800
H	4.47845300	-2.29133500	-0.50889100
C	1.37076600	-1.81223400	-2.04081600
C	0.43262400	-2.56454700	-1.09041800
C	-1.00165500	-2.03539400	-1.14348900
C	-2.01309500	-2.88290900	-0.37033100
C	-3.42976200	-2.27284600	-0.36205600
C	-3.59330900	-1.13702800	0.60897500
C	-3.78361200	0.16375800	0.34565600
C	-3.88965700	0.84014200	-0.99150700
C	-2.99998600	2.09088700	-1.11700100
C	-1.51227600	1.77045000	-1.26426500
C	-0.60169300	2.99826400	-1.28571100
C	0.87275800	2.63812000	-1.48934300
C	1.80711100	3.84675200	-1.42317800
H	1.55080600	4.58770200	-2.18529600
H	1.74477100	4.34324300	-0.44949000
H	2.84879900	3.55661300	-1.58504800
H	0.98519400	2.13911200	-2.45883900
H	1.16567000	1.90066200	-0.73426000
H	-0.92602800	3.68142800	-2.07899200
H	-0.71514700	3.55826700	-0.34696100
H	-1.19057000	1.10817300	-0.45169500
H	-1.36111600	1.18909100	-2.18098800
H	-3.32545300	2.67110600	-1.98536500
H	-3.16095400	2.73918900	-0.24580900
H	-3.66332300	0.14417800	-1.80239500
H	-4.93535500	1.14280300	-1.12427400
H	-3.95202900	0.82743800	1.19543900
H	-3.61715000	-1.44267600	1.65615700
H	-3.69228000	-1.96127000	-1.37550500
H	-4.14891000	-3.04960900	-0.08179000
H	-2.05870000	-3.88013200	-0.81793900
H	-1.67203400	-3.03670300	0.66278300
H	-1.01436300	-1.00387800	-0.77638900
H	-1.32707100	-1.97570100	-2.18897300
H	0.44138900	-3.63097000	-1.34491200
H	0.80145800	-2.49375500	-0.06025900
H	1.26985500	-0.73546400	-1.86540700
H	1.03261700	-1.98201400	-3.06841400
H	3.40116900	-1.83084200	-2.78531200
H	2.91507600	-3.31838100	-2.00196800
N	-0.97969700	0.12165300	2.09114600
C	-1.21914700	1.50859000	2.58071900
H	-1.22433000	2.18216800	1.72609300
H	-2.17832900	1.54939700	3.09395700

H	-0.41657800	1.78021700	3.26432800
C	-0.96899600	-0.90947600	3.16624200
H	-0.81166500	-1.88527700	2.71046700
H	-1.92225100	-0.89376300	3.69194900
H	-0.15604200	-0.68613200	3.85509600
H	-0.04966000	0.10398600	1.61607500
H	-1.70703300	-0.11872500	1.39448600

conf_6

C	3.07778100	-2.31985000	-1.40267500
C	3.36886400	-1.67812800	-0.03803300
C	3.36555300	-0.14597300	-0.10323200
C	3.27957100	0.51699600	1.27175000
C	1.91335300	0.39401400	1.89635800
O	0.90030500	0.13326500	1.27790700
O	1.80194100	0.62927600	3.21855600
H	2.66283000	0.81290400	3.61923700
H	4.03075900	0.10936900	1.95815600
H	3.47924500	1.59400100	1.19858900
H	2.52739100	0.20577500	-0.70619300
H	4.27596600	0.20023600	-0.59942600
H	2.62339600	-2.02150900	0.68815100
H	4.33992900	-2.01722700	0.33701600
C	1.58864800	-2.29686900	-1.78281900
C	0.76972100	-3.37405600	-1.06049300
C	-0.73973900	-3.29468800	-1.32085900
C	-1.44102400	-2.17067200	-0.55315500
C	-2.94464400	-2.08079100	-0.87205000
C	-3.67643500	-1.03642100	-0.06708300
C	-3.72329800	0.27646600	-0.33813300
C	-3.05449600	0.97131600	-1.48888800
C	-1.87653100	1.86180800	-1.05544600
C	-1.24667500	2.62296500	-2.22556200
C	-0.03148700	3.47213900	-1.83403900
C	1.20462600	2.65168000	-1.45153800
C	2.40506200	3.52290500	-1.07727600
H	2.66930000	4.20097800	-1.89314300
H	2.19016800	4.13889800	-0.19817700
H	3.28987900	2.91798300	-0.85987200
H	1.47174900	2.00363600	-2.29488300
H	0.96497600	1.98233000	-0.61938500
H	0.22790700	4.12766300	-2.67187200
H	-0.30223500	4.13771700	-1.00380500
H	-0.95253500	1.90796700	-3.00353300
H	-2.01036800	3.26627300	-2.67492800
H	-2.22290100	2.58712400	-0.30756100
H	-1.11832300	1.23692800	-0.57021600
H	-2.69745600	0.24355000	-2.22135500
H	-3.79544000	1.59538000	-2.00108300
H	-4.31856500	0.91353200	0.31611600
H	-4.23704300	-1.39663300	0.79455800
H	-3.06215600	-1.87950900	-1.94072100
H	-3.40563500	-3.05593300	-0.69192700

H	-1.30204800	-2.34868900	0.52162600
H	-0.96714000	-1.20834100	-0.77188500
H	-0.91872300	-3.16672000	-2.39515300
H	-1.20721200	-4.24626900	-1.04626200
H	1.13682600	-4.35508700	-1.37907200
H	0.94755600	-3.32289800	0.02092200
H	1.17164500	-1.30439800	-1.57798300
H	1.48712700	-2.45140700	-2.86199500
H	3.66540100	-1.80245300	-2.16872300
H	3.42769900	-3.35707000	-1.40269700
N	-1.66747600	0.13727300	2.21389700
C	-1.91386400	1.50755000	2.74318600
H	-2.94634900	1.58397800	3.08074200
H	-1.23377400	1.68951300	3.57362700
H	-1.72841100	2.22521600	1.94704300
C	-1.88335000	-0.94423900	3.21423700
H	-2.90975200	-0.90157200	3.57554500
H	-1.18979600	-0.80141100	4.04125800
H	-1.69804800	-1.90457300	2.73723600
H	-0.68228000	0.09147500	1.86490800
H	-2.27899600	-0.02887400	1.39263400

conf_2

C	3.71830400	1.95767900	0.34964700
C	3.92197900	0.57691400	-0.29126700
C	3.58758100	-0.56551700	0.67609000
C	3.39787800	-1.91195600	-0.02317600
C	2.09215900	-2.00205100	-0.77158800
O	1.14878100	-1.25918300	-0.58721900
O	1.94690500	-2.99801000	-1.66775900
H	2.75685800	-3.51992800	-1.75153300
H	4.22573800	-2.12518900	-0.70943600
H	3.38342900	-2.73323200	0.70560600
H	2.67707600	-0.33603900	1.23220500
H	4.38868400	-0.66514400	1.41299700
H	3.29674500	0.50119500	-1.18834100
H	4.95715000	0.46515200	-0.62950400
C	2.24670000	2.40018700	0.37401600
C	1.76725400	2.95326100	-0.97387100
C	0.26861900	3.27585600	-1.01665900
C	-0.62589800	2.04282900	-1.17853300
C	-2.12376200	2.38401700	-1.08331100
C	-3.01391000	1.23223400	-1.45020000
C	-3.84114000	0.54622200	-0.64815500
C	-4.11576700	0.78551000	0.81030300
C	-3.49710400	-0.27152700	1.74602300
C	-1.97825200	-0.15325200	1.88488300
C	-1.37228500	-1.19777500	2.82601700
C	0.15926600	-1.15689000	2.90900000
C	0.71761000	0.14932400	3.48059200
H	0.29466200	0.35697600	4.46778300
H	1.80342900	0.09814700	3.59304400
H	0.49690900	1.00482100	2.83766900

H	0.57452800	-1.32305500	1.90846700
H	0.49737900	-1.99286700	3.52995900
H	-1.79483400	-1.06340800	3.82847200
H	-1.68684200	-2.19992800	2.50574400
H	-1.49720400	-0.23005900	0.90192000
H	-1.73717400	0.85249900	2.24347300
H	-3.95040600	-0.16688600	2.73642600
H	-3.76968000	-1.27637500	1.39792600
H	-3.76813000	1.77580800	1.11282700
H	-5.20176900	0.78232000	0.94789300
H	-4.42662400	-0.25648200	-1.09798900
H	-2.99340100	0.95649900	-2.50556000
H	-2.34414000	2.74267600	-0.07603100
H	-2.33654900	3.21603100	-1.76475000
H	-0.41300900	1.58210500	-2.15303100
H	-0.37560000	1.29656000	-0.41736300
H	-0.01321700	3.81081700	-0.10166900
H	0.06405000	3.96226100	-1.84491800
H	2.33686500	3.86194500	-1.19385500
H	2.00470300	2.24709300	-1.77934800
H	1.61618400	1.55903600	0.68241300
H	2.11062900	3.17617400	1.13434900
H	4.11984300	1.93725400	1.36848100
H	4.30553700	2.70626300	-0.19147000
N	-1.39792800	-1.53984800	-1.53547800
C	-1.94967300	-2.75414800	-0.87273100
H	-2.97305500	-2.91416900	-1.20814100
H	-1.33112200	-3.60976000	-1.13854500
H	-1.93076600	-2.60128800	0.20411900
C	-1.35098100	-1.62580900	-3.02150600
H	-2.35682600	-1.78747600	-3.40616400
H	-0.70257600	-2.45358000	-3.30354600
H	-0.94852100	-0.69277200	-3.41141000
H	-0.42642600	-1.38206900	-1.18282100
H	-1.96399500	-0.71609000	-1.26162700

conf_8

C	-1.17449600	0.98658700	1.66078300
C	-2.70454200	1.02406200	1.70692200
C	-3.38655400	1.13748000	0.33707000
C	-3.05841400	2.42370600	-0.43691600
C	-1.66889100	2.44424100	-1.02251700
O	-1.09545600	1.46698200	-1.45887900
O	-1.02820500	3.63153400	-1.08154300
H	-1.56877600	4.34091700	-0.70866600
H	-3.74033000	2.52505100	-1.29009900
H	-3.21466100	3.30538200	0.19391700
H	-4.47000900	1.10248700	0.47331400
H	-3.12128100	0.28140500	-0.28718200
H	-3.06523900	0.10845700	2.18640000
H	-3.03159000	1.85033200	2.34981300
C	-0.52115300	0.70489200	3.01526200
C	1.00940900	0.80657000	2.98331600

C	1.68558900	-0.21029700	2.05894800
C	3.21279200	-0.12778000	2.07500300
C	3.89538100	-1.19242500	1.19328400
C	3.76463300	-0.96518700	-0.28961000
C	2.91116300	-1.56568300	-1.13174400
C	1.82539500	-2.54258600	-0.78575100
C	0.44110500	-2.05820300	-1.24738900
C	-0.70108200	-2.97398300	-0.80670000
C	-2.05953500	-2.51934400	-1.34740800
C	-3.25624400	-3.30077000	-0.79062800
C	-3.52011800	-3.05585700	0.69800700
H	-2.68282900	-3.37847700	1.32221700
H	-3.69216000	-1.99256100	0.89470600
H	-4.40482600	-3.60063500	1.03526000
H	-3.10248400	-4.37164700	-0.96543700
H	-4.15012600	-3.02708500	-1.36033800
H	-2.19103000	-1.45217800	-1.12971300
H	-2.04896800	-2.60420400	-2.43995800
H	-0.50087500	-3.99953000	-1.13796900
H	-0.71999600	-3.00370600	0.28804100
H	0.24718500	-1.05735100	-0.84240500
H	0.43358400	-1.95722200	-2.34017700
H	2.03892200	-3.50372900	-1.26826000
H	1.80328800	-2.73003800	0.28925500
H	3.01553400	-1.35085200	-2.19645700
H	4.49139000	-0.27475700	-0.71780000
H	4.96310600	-1.20983200	1.43124000
H	3.50491100	-2.17653800	1.46350900
H	3.54006900	0.87384300	1.76787100
H	3.56470700	-0.25516000	3.10333600
H	1.37886100	-1.21982400	2.35795500
H	1.32104600	-0.08818500	1.03317500
H	1.29710200	1.82499500	2.68638800
H	1.39918700	0.67373000	3.99761500
H	-0.90986600	1.40931800	3.75840000
H	-0.81436100	-0.29431300	3.35781900
H	-0.86541900	0.23141900	0.93249100
H	-0.78758700	1.94794600	1.29571100
N	1.66062800	1.37499700	-1.54277100
C	2.14741600	2.46599900	-0.65201700
H	1.80954300	2.26178400	0.36166600
H	3.23525000	2.49218800	-0.68191000
H	1.73238300	3.41016800	-0.99799700
C	2.07819500	1.52016800	-2.96592700
H	3.16554400	1.51459400	-3.02363900
H	1.68719400	2.46000500	-3.35169100
H	1.66848900	0.68836200	-3.53583100
H	0.61643700	1.35230400	-1.51010900
H	2.01328100	0.47049800	-1.18362300

conf_27

C	-2.12362900	2.42666800	0.93367800
C	-1.90588000	3.62130300	-0.00330100

C	-1.56284900	3.29467500	-1.46252800
C	-0.23467100	2.53579000	-1.63324100
C	-0.36419700	1.03999700	-1.54393000
O	-1.30498700	0.40650200	-1.98440200
O	0.64630200	0.33818100	-0.99510900
H	1.36557600	0.89893500	-0.66437400
H	0.16663500	2.71835000	-2.63802800
H	0.51991600	2.89657400	-0.92716900
H	-1.48598300	4.23222000	-2.01735700
H	-2.35992000	2.71760000	-1.93664100
H	-2.81140500	4.23660900	0.00050100
H	-1.11031200	4.25410300	0.40721200
C	-3.32084300	1.53765400	0.57199400
C	-3.79796200	0.64343300	1.72811100
C	-2.71634200	-0.22018600	2.38878800
C	-2.03367500	-1.22370900	1.45629200
C	-0.94026200	-2.02864600	2.17994500
C	-0.25083800	-3.04932800	1.31864500
C	1.02305100	-3.00922200	0.90122200
C	2.02926200	-1.93208800	1.20718800
C	3.10253800	-1.76490500	0.12218500
C	4.11951500	-0.65779300	0.43327500
C	3.56840000	0.76618200	0.29858000
C	4.55757900	1.86558300	0.69226000
C	3.97859400	3.26964000	0.51252900
H	3.08265700	3.40951800	1.12670900
H	4.69710600	4.03880700	0.80264300
H	3.70561200	3.45413700	-0.53199400
H	5.46816600	1.76020800	0.09317700
H	4.85689200	1.71898600	1.73531100
H	3.27992200	0.92830100	-0.75236400
H	2.66856700	0.87815800	0.92065300
H	4.50503100	-0.79894400	1.44950300
H	4.97968100	-0.76065200	-0.23541100
H	3.63231700	-2.71573400	0.00418300
H	2.62423600	-1.55817200	-0.84305600
H	1.51714800	-0.98476700	1.38664200
H	2.53317000	-2.18629300	2.14926500
H	1.40221600	-3.86027000	0.33573100
H	-0.84289000	-3.92820800	1.06278000
H	-0.21247000	-1.33404600	2.60504300
H	-1.39578600	-2.54979900	3.02972700
H	-2.78817800	-1.90662600	1.04492200
H	-1.58678700	-0.69344800	0.60861300
H	-1.95011400	0.42496100	2.83282700
H	-3.16543900	-0.76730000	3.22440100
H	-4.24098500	1.27975900	2.50167500
H	-4.60756000	-0.00161100	1.36787200
H	-4.15798500	2.17782000	0.27247600
H	-3.08468300	0.92935000	-0.30520500
H	-1.20870300	1.82116500	0.99264600
H	-2.26856800	2.82202600	1.94520700
N	-0.87158200	-2.30460600	-1.76054700
C	-2.11965200	-3.10191800	-1.91322700
H	-2.82734700	-2.79145000	-1.14751800

H	-2.53907700	-2.91637700	-2.90063600
H	-1.88804200	-4.16008800	-1.80032100
C	0.18587700	-2.62601300	-2.75829500
H	0.46770500	-3.67329200	-2.66052600
H	1.04578200	-1.98891300	-2.56337900
H	-0.20098100	-2.43596900	-3.75810400
H	-0.48332900	-2.45012300	-0.81040900
H	-1.09533400	-1.28341900	-1.82832600

conf_1

C	3.76383200	-1.11053700	-1.21565200
C	4.19117700	-0.89587500	0.25209100
C	4.36195500	0.57889000	0.64863000
C	3.11505200	1.45871200	0.47660000
C	1.91143000	1.06618800	1.29274900
O	0.76632000	1.22514800	0.91278300
O	2.10745600	0.54451000	2.51456700
H	3.05206900	0.44074800	2.69982300
H	3.34679900	2.49257700	0.76202800
H	2.78791400	1.49846400	-0.56180400
H	5.15406300	1.02312900	0.03967600
H	4.73660500	0.64686600	1.68027900
H	3.47296000	-1.38548600	0.92020100
H	5.14673900	-1.39248100	0.43719100
C	2.26488000	-1.38132800	-1.41906000
C	1.83826100	-2.78592000	-0.97419400
C	0.34646400	-3.07613600	-1.17984800
C	-0.57437700	-2.36842100	-0.18107200
C	-2.06405300	-2.57118700	-0.50985200
C	-2.97998900	-1.95124900	0.50433300
C	-3.89399300	-0.99063600	0.30219500
C	-4.25333700	-0.31665300	-0.99551700
C	-3.57573300	1.05569400	-1.19527300
C	-2.09818700	0.94662800	-1.57688300
C	-1.35983300	2.28418000	-1.64708800
C	0.04901100	2.15036700	-2.23154100
C	0.86638700	3.43792300	-2.13015500
H	1.85802100	3.32067700	-2.57575500
H	0.37146300	4.26472300	-2.64726700
H	0.99866900	3.73499300	-1.08490300
H	-0.02804700	1.84193500	-3.28013300
H	0.57063900	1.34237800	-1.70947000
H	-1.93833900	2.99642200	-2.24688800
H	-1.29026000	2.72001700	-0.64157100
H	-1.57223000	0.29192100	-0.87360700
H	-2.02796700	0.44406900	-2.54891400
H	-4.10671900	1.60006300	-1.98173200
H	-3.69540000	1.65901400	-0.28592200
H	-4.00815000	-0.95900900	-1.84490700
H	-5.33864600	-0.18262900	-1.01227100
H	-4.48567200	-0.67596900	1.16238300
H	-2.89790000	-2.36427000	1.51032800
H	-2.26661300	-2.17631000	-1.50713300

H	-2.26721200	-3.64816100	-0.55147500
H	-0.36482000	-2.74655700	0.82824600
H	-0.35065700	-1.29619900	-0.16908100
H	0.06193800	-2.78738700	-2.19878200
H	0.17269700	-4.15480400	-1.10943100
H	2.42359600	-3.51695500	-1.54158900
H	2.09573900	-2.94814700	0.07992600
H	1.66966700	-0.62438500	-0.89861900
H	2.02532800	-1.26863300	-2.48188500
H	4.06387700	-0.23979600	-1.80904300
H	4.32162900	-1.95404100	-1.63261000
N	-1.56817700	0.49319700	2.10259700
C	-2.29427400	1.74527100	2.45316200
H	-1.69862800	2.30868700	3.16952400
H	-2.43480900	2.33151300	1.54766800
H	-3.25997800	1.49294400	2.88852600
C	-1.27070900	-0.38523300	3.26811200
H	-0.75510400	-1.27375200	2.90970100
H	-2.20296000	-0.66477700	3.75652400
H	-0.62905700	0.15529700	3.96133600
H	-0.66412800	0.74669200	1.63895900
H	-2.12385800	-0.04709100	1.41507100

conf_36

C	-5.18572700	-0.91365400	-0.18199900
C	-4.03227200	-1.92173900	-0.08216000
C	-3.25200100	-2.11564300	-1.38532200
C	-2.04491500	-3.05204300	-1.20980100
C	-1.02763400	-2.45313100	-0.27143800
O	-0.61764700	-1.31223600	-0.36779000
O	-0.54934500	-3.21057000	0.73417000
H	-0.94531300	-4.09233700	0.72853600
H	-2.36570400	-4.03678400	-0.85668000
H	-1.53680900	-3.20001100	-2.16853000
H	-2.88759600	-1.15866600	-1.76359900
H	-3.90440800	-2.53458900	-2.15600600
H	-3.34418700	-1.59754400	0.70861700
H	-4.43344800	-2.88824100	0.24356100
C	-4.77593000	0.49841900	-0.62702800
C	-3.63760300	1.11219300	0.19259600
C	-3.34011700	2.56805100	-0.18363400
C	-2.05166500	3.11058800	0.44676300
C	-0.78220100	2.55230100	-0.21012500
C	0.48892900	2.93546100	0.51089900
C	1.72440300	2.85327200	-0.00088800
C	2.06391600	2.38258900	-1.39044700
C	3.46232400	1.76467300	-1.51216900
C	3.64491500	0.51265100	-0.65376000
C	4.99348600	-0.18204400	-0.84896500
C	5.18150800	-1.41330400	0.04111800
C	6.52183500	-2.11612800	-0.18083600
H	7.35693300	-1.44115700	0.02613500
H	6.62953000	-2.98815600	0.46849700

H	6.61941100	-2.45608000	-1.21564000
H	4.36400700	-2.12236800	-0.14618600
H	5.10040900	-1.11315100	1.09407200
H	5.09757700	-0.47558600	-1.90000000
H	5.80031000	0.53331500	-0.65130100
H	3.53905200	0.78845100	0.40420100
H	2.83597900	-0.19709200	-0.88164300
H	3.64933100	1.51600000	-2.56120700
H	4.21692600	2.50905800	-1.23358000
H	1.31365600	1.66651100	-1.74259100
H	1.99595400	3.24417700	-2.06744900
H	2.55653900	3.21518500	0.60090200
H	0.37228700	3.36155900	1.50631000
H	-0.85758900	1.45979200	-0.29553100
H	-0.73701700	2.90700400	-1.24485300
H	-2.03125900	4.20210000	0.37471400
H	-2.04975500	2.88340800	1.52151500
H	-3.26943900	2.65642100	-1.27464200
H	-4.18617400	3.19363200	0.11632100
H	-3.88617800	1.06155300	1.26121400
H	-2.73435900	0.50788500	0.05579700
H	-4.48525300	0.48946200	-1.68363800
H	-5.65534000	1.14801200	-0.56853500
H	-5.94266800	-1.29918300	-0.87328000
H	-5.67169000	-0.85387500	0.79772000
N	0.77527600	-0.03545700	1.62253400
C	1.87469400	-0.86190700	2.19751500
H	2.35504500	-0.30994200	3.00397400
H	1.44557000	-1.78680300	2.57873800
H	2.59671600	-1.08364200	1.41685000
C	-0.29778900	0.29307800	2.60422400
H	0.12648100	0.87768000	3.41875700
H	-0.71163100	-0.63723000	2.98943300
H	-1.07392300	0.86326200	2.10028600
H	0.33623100	-0.55178800	0.82635000
H	1.14186800	0.84829000	1.22635400

conf_154

C	4.73491200	-2.84163600	0.13848600
C	5.30540000	-1.44862600	0.44122000
C	5.56068600	-0.60275600	-0.81563700
C	5.60758300	0.89673200	-0.51634600
C	4.24664700	1.45950600	-0.18584400
O	3.19588900	0.93989800	-0.50384600
O	4.19511300	2.63367000	0.47697900
H	5.08061200	2.95260600	0.69937900
H	6.30218400	1.11741200	0.30229900
H	5.96643400	1.46101400	-1.38705600
H	4.78032900	-0.77353300	-1.55900200
H	6.50542200	-0.89857000	-1.27807900
H	4.61785200	-0.91862200	1.10931900
H	6.24199000	-1.55151700	0.99842000
C	3.30490300	-2.82980300	-0.42306700

C	2.25175200	-2.30934900	0.55945500
C	0.87277900	-2.12900900	-0.07719600
C	-0.17675200	-1.62748600	0.91696300
C	-1.57321500	-1.37569400	0.31690800
C	-1.59668000	-0.34800300	-0.78006100
C	-2.16981600	0.86435800	-0.74065400
C	-2.92877700	1.48589200	0.39962800
C	-4.45255400	1.49605900	0.15446500
C	-5.07855600	0.10052400	0.15003100
C	-6.58775300	0.12235300	-0.10043300
C	-7.22142200	-1.27112700	-0.09831900
C	-8.72949500	-1.24172800	-0.35158600
H	-9.24637300	-0.65670500	0.41494400
H	-8.95740200	-0.79073300	-1.32202400
H	-9.15484300	-2.24813300	-0.34459100
H	-7.02012100	-1.75647600	0.86413300
H	-6.73284200	-1.88967500	-0.86070500
H	-7.07508100	0.74271600	0.66192400
H	-6.78811600	0.60933900	-1.06299300
H	-4.59467300	-0.51803800	-0.61420000
H	-4.87902900	-0.38703000	1.11290800
H	-4.92398800	2.10637300	0.93159300
H	-4.66012900	1.99606400	-0.79887900
H	-2.71826300	0.97060400	1.34102100
H	-2.59787400	2.52506400	0.52590800
H	-2.15513800	1.45367800	-1.65762500
H	-1.13115700	-0.64687000	-1.71672200
H	-1.94772900	-2.31844400	-0.09875800
H	-2.26373400	-1.09860700	1.11486900
H	-0.27616200	-2.35313800	1.73044600
H	0.18286000	-0.70370700	1.39072700
H	0.96683900	-1.42982100	-0.91690300
H	0.53392100	-3.07756700	-0.51005300
H	2.17885500	-2.99395400	1.41294100
H	2.56905100	-1.34332500	0.96702900
H	3.26733600	-2.22700300	-1.33728900
H	3.03723700	-3.84715600	-0.72641800
H	5.39612100	-3.35261200	-0.56988700
H	4.75414000	-3.43863600	1.05639000
N	0.70048600	1.95084600	0.05336000
C	0.57010600	3.13614700	-0.84059900
H	-0.38018100	3.62988900	-0.64541900
H	1.39588400	3.81690700	-0.64122400
H	0.60807700	2.79963200	-1.87508500
C	0.65737900	2.28618700	1.50485200
H	-0.28703600	2.77728100	1.73143900
H	1.49336100	2.94466700	1.73431100
H	0.74341300	1.36591800	2.07925600
H	1.61394800	1.48793000	-0.15431300
H	-0.06018800	1.28305700	-0.17421000

conf_60

C	-1.83347000	-3.11236200	-0.54616900

C	-0.65514700	-2.36771200	0.09186600
C	0.16076100	-3.21423700	1.06982000
C	1.38644700	-2.46218500	1.63747800
C	1.00100900	-1.11344000	2.18314300
O	1.35686800	-0.05721900	1.69529100
O	0.17764100	-1.07559600	3.24478500
H	-0.05125100	-1.96597200	3.54540500
H	1.86223100	-3.05004300	2.42940700
H	2.12469300	-2.28777600	0.85667900
H	0.52558900	-4.12110700	0.58054700
H	-0.48874400	-3.55510600	1.88530000
H	0.00282500	-1.98329700	-0.69608600
H	-1.03600600	-1.48729800	0.62273800
C	-2.83886500	-2.15215000	-1.19976800
C	-3.74596700	-1.46020900	-0.17185200
C	-4.52058200	-0.24569600	-0.70061600
C	-3.64883700	0.91668500	-1.19833300
C	-2.60856200	1.39757400	-0.16666700
C	-1.83507400	2.59815100	-0.64736900
C	-0.76348400	2.56415700	-1.45428100
C	-0.12679600	1.34168700	-2.04998200
C	1.37560300	1.20391800	-1.75482700
C	2.04056300	0.12199400	-2.61264400
C	3.53057000	-0.08466300	-2.31727300
C	3.81565700	-0.78775000	-0.98635100
C	5.31036600	-0.95323800	-0.70744300
H	5.80936000	0.01876500	-0.65170600
H	5.48879300	-1.47248400	0.23789300
H	5.79811700	-1.52811500	-1.49960500
H	3.33753400	-1.77587300	-1.00975200
H	3.34781200	-0.23977800	-0.16197800
H	3.97592700	-0.67346600	-3.12607500
H	4.04239400	0.88600500	-2.33529200
H	1.50780200	-0.82809300	-2.47632500
H	1.91302600	0.39183700	-3.66639500
H	1.87510800	2.16247700	-1.94412800
H	1.51804300	0.97014300	-0.69474500
H	-0.64656500	0.43524400	-1.73132100
H	-0.26031400	1.40209400	-3.13775100
H	-0.32959200	3.51345600	-1.76593000
H	-2.23064400	3.57342400	-0.36969200
H	-3.12399900	1.63962000	0.76946200
H	-1.92214000	0.57537300	0.05517800
H	-3.12251200	0.63618400	-2.11518200
H	-4.29535500	1.75652400	-1.47015500
H	-5.17798800	-0.55935000	-1.51845800
H	-5.17947400	0.12011300	0.09484600
H	-4.46266100	-2.19514700	0.20880200
H	-3.15447500	-1.15511200	0.69902200
H	-2.28481900	-1.41067200	-1.78664500
H	-3.46268000	-2.69442400	-1.91638000
H	-1.45060800	-3.82254800	-1.28530500
H	-2.34964400	-3.70964800	0.21522700
N	0.40176600	2.49560900	1.62391000
C	1.59115300	3.38464000	1.49923100

H	2.18321300	3.31437200	2.41012100
H	2.18190200	3.05395900	0.64720500
H	1.25862100	4.41061300	1.34804700
C	-0.50724200	2.83589700	2.75354200
H	-0.88915200	3.84639000	2.61649900
H	0.04774600	2.76983500	3.68780300
H	-1.33083000	2.12444600	2.76394800
H	0.72833900	1.50907000	1.73112600
H	-0.13666400	2.53224900	0.73664600

conf_0

C	-3.64395800	2.03305500	-0.83451600
C	-3.80912700	0.90894400	0.18771100
C	-3.59441100	-0.46560200	-0.43900500
C	-3.40879400	-1.57784200	0.58120800
C	-2.07670400	-1.52917200	1.26963300
O	-1.13291500	-0.87476900	0.88133100
O	-1.91193100	-2.28723000	2.36002600
H	-2.72500400	-2.75377200	2.58625000
H	-4.20460600	-1.56326200	1.33713900
H	-3.46236000	-2.56416700	0.09934800
H	-2.71975000	-0.44878400	-1.09489600
H	-4.45175000	-0.71418000	-1.07189800
H	-3.09772000	1.06185400	1.00942500
H	-4.80857600	0.94543100	0.63571800
C	-2.18050300	2.38216600	-1.11014600
C	-1.58581200	3.31446900	-0.05771100
C	-0.08286300	3.53535200	-0.20881700
C	0.75891400	2.40533700	0.37252900
C	2.24997800	2.55136100	0.05913700
C	3.10123600	1.59045600	0.82510300
C	3.89616600	0.62592400	0.34040000
C	4.16206500	0.29098800	-1.09513500
C	3.43301000	-0.96870400	-1.57915900
C	1.94199600	-0.74557700	-1.78408800
C	1.15963800	-1.99577100	-2.15816400
C	-0.31198900	-1.70407900	-2.42154500
C	-1.15425200	-2.95695800	-2.60545300
H	-1.12012300	-3.59168300	-1.71230000
H	-2.20161800	-2.71165600	-2.80421600
H	-0.79540100	-3.56027000	-3.44460900
H	-0.39694800	-1.06679800	-3.30982100
H	-0.70461300	-1.11461600	-1.58506600
H	1.61005800	-2.46540600	-3.04132400
H	1.24299900	-2.74265000	-1.35447300
H	1.49946400	-0.30488800	-0.88003300
H	1.80302700	0.01277400	-2.56557800
H	3.88000200	-1.29645700	-2.52359600
H	3.60482100	-1.78979400	-0.86894900
H	3.89531900	1.12988600	-1.74463300
H	5.24145100	0.14428900	-1.20761600
H	4.45910400	0.03233000	1.06370200
H	3.08865000	1.73112800	1.90880500

H	2.40586000	2.44186600	-1.01734300
H	2.56301100	3.57130900	0.31791000
H	0.61164000	2.38871600	1.46232900
H	0.39746700	1.44113300	-0.00672300
H	0.16016900	3.66218300	-1.27182900
H	0.20785500	4.47130200	0.28143000
H	-2.10412300	4.27764200	-0.12170000
H	-1.79395400	2.93017400	0.95045000
H	-1.58705400	1.46067400	-1.17768900
H	-2.09407400	2.86742800	-2.08872600
H	-4.14343300	1.73978200	-1.76528000
H	-4.16386400	2.93212500	-0.48563900
N	1.37871100	-0.87652600	1.92524300
C	1.91252300	-2.25086800	1.82802900
H	2.91402200	-2.28322200	2.25712200
H	1.25220300	-2.92533800	2.37308100
H	1.95065800	-2.53883900	0.77828400
C	1.28575400	-0.36785300	3.30952800
H	2.27596900	-0.36928800	3.76552300
H	0.61387200	-1.01139300	3.87732500
H	0.88983000	0.64685700	3.28589500
H	0.42128300	-0.85655000	1.50083800
H	1.97681800	-0.23796700	1.36855800

conf_45

C	3.75529600	-1.24394600	-1.61070100
C	4.34093200	-0.78613800	-0.26074000
C	4.57196100	0.72614800	-0.11525100
C	3.31690700	1.61487900	-0.20019800
C	2.22927800	1.25318500	0.77536300
O	1.05338600	1.16698000	0.47967000
O	2.58018200	1.01541900	2.05326200
H	3.53819200	1.08555700	2.17157500
H	3.59124400	2.65814000	-0.00130900
H	2.87239400	1.58974100	-1.19333800
H	5.25805600	1.06496500	-0.89627200
H	5.10194900	0.91929400	0.82762300
H	3.71625000	-1.15030400	0.56318000
H	5.31268300	-1.26667600	-0.11912000
C	2.22181400	-1.26681400	-1.72141000
C	1.54373000	-2.31592200	-0.83328900
C	0.01744700	-2.27940800	-0.93625400
C	-0.68164000	-3.43448600	-0.21684600
C	-2.21914100	-3.33899600	-0.26204600
C	-2.79133200	-2.31541800	0.67792200
C	-3.49304200	-1.21275400	0.37901500
C	-3.89713900	-0.70231800	-0.97436600
C	-3.51764300	0.77032200	-1.21617200
C	-2.02264900	0.97866400	-1.47173500
C	-1.60925900	2.43672000	-1.69794800
C	-1.68460600	3.31805200	-0.44743000
C	-1.25677300	4.76265800	-0.71196000
H	-1.30642600	5.37025600	0.19505800

H	-0.23017700	4.80461100	-1.08820100
H	-1.90102700	5.23128300	-1.46083900
H	-1.03442400	2.88598300	0.32350200
H	-2.70421400	3.31366900	-0.04549700
H	-0.58092900	2.45752300	-2.07465900
H	-2.23300600	2.87206500	-2.48774000
H	-1.43648800	0.57304900	-0.64018700
H	-1.73607800	0.38253400	-2.34497400
H	-4.07616800	1.13940600	-2.08181300
H	-3.85306600	1.37179600	-0.36340100
H	-3.47919100	-1.32394000	-1.76931100
H	-4.98721400	-0.79736000	-1.04668800
H	-3.88867700	-0.62899800	1.21198800
H	-2.66162800	-2.55562200	1.73461800
H	-2.53892800	-3.14899700	-1.28884500
H	-2.63934000	-4.31148800	0.01720500
H	-0.37287000	-4.37735600	-0.67806900
H	-0.34912500	-3.49266500	0.82815800
H	-0.34588000	-1.31765000	-0.55848700
H	-0.27331900	-2.30004800	-1.99305700
H	1.90637200	-3.31193000	-1.11319000
H	1.83704900	-2.17636900	0.21521200
H	1.79353500	-0.28459100	-1.50499900
H	1.96005200	-1.48026800	-2.76324500
H	4.16660800	-0.61374400	-2.40728100
H	4.12015700	-2.25499800	-1.81766700
N	-0.88399600	-0.02393900	2.00055600
C	-1.68910700	0.98954000	2.73802600
H	-2.42980500	0.48108500	3.35336600
H	-1.02281200	1.57768200	3.36694800
H	-2.18017500	1.63685300	2.01461600
C	-0.16032700	-0.98577800	2.87808100
H	0.36473000	-1.70084600	2.24798700
H	-0.87642600	-1.50503700	3.51267700
H	0.55516100	-0.43448800	3.48495500
H	-0.17738500	0.46735100	1.40603600
H	-1.50846800	-0.55022900	1.36378400

conf_95

C	4.95488800	-1.62831100	-1.06257300
C	4.60424400	-0.14296000	-1.22477300
C	3.59814100	0.15051100	-2.34186000
C	3.19143900	1.62972900	-2.38337500
C	2.43979200	2.03691800	-1.14005200
O	1.63295000	1.32109000	-0.57772400
O	2.66531300	3.25804000	-0.62386400
H	3.32826800	3.74061900	-1.13600700
H	4.06716600	2.27135600	-2.52426900
H	2.51811200	1.82011000	-3.22752900
H	2.69498800	-0.44931100	-2.21512000
H	4.02453500	-0.11890600	-3.31183400
H	4.20931400	0.23419900	-0.27386000
H	5.52523600	0.41957800	-1.41509900

C	3.75893100	-2.55237000	-0.78817900
C	2.86241900	-2.10412600	0.36994500
C	1.78875800	-3.13718500	0.73245800
C	0.69666600	-2.58360100	1.65589300
C	-0.31776900	-1.69973100	0.91597800
C	-1.29459600	-1.00732300	1.83143600
C	-2.05393100	0.05525800	1.52207600
C	-2.09813800	0.73997500	0.18059100
C	-3.17811700	0.14882700	-0.75203400
C	-4.60466000	0.29006000	-0.21785700
C	-5.66059200	-0.24138100	-1.18927600
C	-7.09163900	-0.10840600	-0.66232600
C	-8.13978700	-0.64165600	-1.64027700
H	-7.97682700	-1.70249400	-1.85291000
H	-9.15036600	-0.53462300	-1.23902300
H	-8.10094700	-0.10328600	-2.59199700
H	-7.29755000	0.94578100	-0.44201600
H	-7.17476900	-0.64185900	0.29201000
H	-5.57571000	0.29099600	-2.14492500
H	-5.45261500	-1.29617500	-1.40863500
H	-4.69698700	-0.24115800	0.73640900
H	-4.80837800	1.34737100	-0.00367400
H	-3.10207900	0.64785600	-1.72413400
H	-2.95373900	-0.90900700	-0.92604800
H	-2.31194000	1.80658300	0.31840900
H	-1.12937600	0.66509100	-0.32393000
H	-2.75219600	0.41333200	2.27433200
H	-1.41761600	-1.44925400	2.81853700
H	0.19917100	-0.96963700	0.28313800
H	-0.87771000	-2.33182100	0.21545400
H	0.15776900	-3.40721300	2.13410600
H	1.16240400	-2.01854000	2.47446700
H	1.32047500	-3.51528300	-0.18446600
H	2.26920700	-3.99966700	1.20374700
H	3.48055500	-1.90590200	1.25625500
H	2.38424500	-1.15479600	0.10607200
H	3.14446500	-2.64962200	-1.69041400
H	4.14049600	-3.55775900	-0.58182500
H	5.47502500	-1.97764300	-1.96083300
H	5.67369900	-1.71988300	-0.24120000
N	0.67820600	1.56264100	1.98001300
C	0.16978900	2.92077400	2.31949900
H	-0.16785200	2.93076100	3.35469800
H	0.97237800	3.64281200	2.17905800
H	-0.65970800	3.15728100	1.65606200
C	1.80825700	1.09970100	2.83242100
H	2.62792700	1.81152200	2.74753800
H	2.12966100	0.12101200	2.48200000
H	1.47704100	1.03578400	3.86778600
H	0.99030400	1.55005800	0.98306900
H	-0.10418600	0.88200500	2.04291900

conf_24

C	-3.06742200	-1.37970300	-0.14150800
C	-3.37011600	-2.85248600	-0.43541500
C	-2.34294600	-3.84724700	0.12185100
C	-0.96671500	-3.79734400	-0.56240000
C	-0.16877100	-2.56265200	-0.22947600
O	-0.09760200	-2.08398100	0.88650700
O	0.51530100	-1.96495100	-1.21925400
H	0.38633700	-2.42048100	-2.06174900
H	-0.35818700	-4.64437900	-0.22459100
H	-1.07622000	-3.90026600	-1.64690300
H	-2.72701200	-4.86298300	0.00187300
H	-2.20632100	-3.68757600	1.19501800
H	-4.34557800	-3.10025400	-0.00504100
H	-3.47379300	-2.99896400	-1.51779300
C	-4.17352300	-0.42348900	-0.59339600
C	-3.97801100	1.01521900	-0.10054400
C	-2.69187800	1.69781700	-0.57837100
C	-2.61777400	3.16631900	-0.15388400
C	-1.30867400	3.86837200	-0.55457200
C	-0.13591400	3.52832900	0.31989700
C	1.06413200	3.05534500	-0.04676300
C	1.51672800	2.67669700	-1.42894400
C	2.06679400	1.24040900	-1.51204200
C	3.39505100	1.02741100	-0.77932600
C	3.82024900	-0.44110900	-0.71478900
C	5.13393000	-0.66590100	0.03725500
C	5.53673900	-2.13950100	0.10883200
H	6.47571400	-2.27136000	0.65102400
H	4.77195400	-2.73475500	0.61823400
H	5.67053200	-2.55905000	-0.89249600
H	5.04570400	-0.25967600	1.05338100
H	5.92961300	-0.08934200	-0.44698800
H	3.02367400	-1.03323500	-0.24422000
H	3.91321600	-0.83555300	-1.73389800
H	4.17612800	1.61434900	-1.27538000
H	3.33825200	1.42785500	0.24034300
H	1.31375400	0.53982400	-1.13120900
H	2.20183400	0.97712000	-2.56617300
H	0.69756700	2.79189300	-2.14029200
H	2.30210100	3.37449000	-1.74520500
H	1.82563300	2.98337700	0.72922400
H	-0.27578100	3.77805900	1.37243800
H	-1.09088400	3.66671000	-1.60561200
H	-1.45928800	4.95167800	-0.47624200
H	-3.45776800	3.70300700	-0.60467200
H	-2.75835900	3.24781400	0.93162900
H	-1.81074200	1.16005600	-0.20596100
H	-2.63136800	1.63678300	-1.67194100
H	-4.83244000	1.61914500	-0.42316000
H	-4.00746400	1.02458800	0.99767000
H	-4.23929000	-0.43133300	-1.68788400
H	-5.13773500	-0.79143500	-0.22722400
H	-2.90682200	-1.26453700	0.93822200
H	-2.13203300	-1.08185000	-0.62568400
N	0.34290300	0.45394400	1.78081100

C	1.63489300	0.38395600	2.52278000
H	1.82224400	1.33966800	3.00966000
H	1.56542000	-0.40778300	3.26651100
H	2.43102200	0.15856300	1.81733100
C	-0.83889300	0.70789700	2.65374000
H	-0.93108700	-0.11243600	3.36300000
H	-1.72840300	0.76187400	2.03070900
H	-0.69690100	1.64874600	3.18267500
H	0.20804600	-0.45880700	1.28945500
H	0.39554600	1.20426100	1.07066500

conf_56

C	-3.69431700	-2.25405200	0.46157900
C	-4.61571300	-2.48626800	-0.74210700
C	-4.66316400	-1.34857400	-1.77356500
C	-5.15299400	-0.00665200	-1.20302400
C	-4.09281900	0.78576000	-0.48057300
O	-2.93225600	0.84933900	-0.83535700
O	-4.46334600	1.51205600	0.59426500
H	-5.39784300	1.37908400	0.80276200
H	-5.48005900	0.64352600	-2.02387800
H	-6.02460400	-0.15569900	-0.55726300
H	-5.34549800	-1.63545600	-2.57676900
H	-3.68714500	-1.19430400	-2.23873000
H	-4.30821500	-3.40099500	-1.25909500
H	-5.63337600	-2.67232700	-0.38058100
C	-2.20685900	-2.15611600	0.11359400
C	-1.34170500	-1.72756800	1.30160500
C	0.08179900	-1.34723900	0.89427500
C	0.98454900	-0.95585600	2.06624200
C	2.31917000	-0.33195900	1.61222400
C	2.18269200	1.08742000	1.13387100
C	2.18395200	1.52983900	-0.13208700
C	2.32881800	0.72215300	-1.39031600
C	3.66923800	1.00175900	-2.10446200
C	4.89964200	0.51657200	-1.32886300
C	5.05312000	-1.00825700	-1.29742900
C	6.26839100	-1.49487400	-0.49695200
C	6.15735300	-1.25539900	1.01159200
H	7.03354000	-1.64354700	1.53615100
H	6.07742900	-0.19207200	1.25250100
H	5.27640200	-1.75875500	1.42309000
H	7.17070700	-1.00560500	-0.88129900
H	6.40347100	-2.56596100	-0.67795200
H	4.15049300	-1.47399300	-0.88253500
H	5.13231500	-1.37111500	-2.32859000
H	5.79595700	0.94483800	-1.78997400
H	4.86342100	0.91610100	-0.31030000
H	3.64428500	0.52519700	-3.08994300
H	3.75171000	2.07934400	-2.28500400
H	2.22635700	-0.34391300	-1.18300800
H	1.51321900	0.98235400	-2.07700000
H	2.15323300	2.60974000	-0.28463300

H	2.11267400	1.83330800	1.92601900
H	3.02020900	-0.33485700	2.45203200
H	2.76474000	-0.95936300	0.83822600
H	0.46328000	-0.25506100	2.73160700
H	1.19041300	-1.84280100	2.67245500
H	0.54407500	-2.18181400	0.35499200
H	0.03419900	-0.52837200	0.16496400
H	-1.81681700	-0.86873200	1.79642000
H	-1.31444700	-2.52308700	2.05456700
H	-1.85802000	-3.12286300	-0.26605400
H	-2.06350500	-1.43847000	-0.69836000
H	-4.00073500	-1.34408600	0.99644700
H	-3.84562100	-3.06613600	1.18014400
N	-0.91866500	2.17051200	0.49507800
C	-1.30526200	2.30666800	1.92801200
H	-2.30074900	2.74306700	1.98026100
H	-1.31239100	1.31786900	2.38104600
H	-0.58207600	2.94316700	2.43507100
C	-0.83513700	3.46858600	-0.23245500
H	-0.09517500	4.10443600	0.25065800
H	-1.81255300	3.94722300	-0.20811800
H	-0.54424800	3.27385100	-1.26301400
H	-1.63006100	1.57725600	0.01149500
H	0.00197200	1.69863500	0.43742100

conf_58

C	4.29958000	2.35408000	0.07976400
C	2.93733200	2.71385000	0.68942600
C	1.91267100	3.22802700	-0.32964500
C	0.45984600	3.19122400	0.20571900
C	-0.00251200	1.76880300	0.36322200
O	-0.09205800	1.18257700	1.42456100
O	-0.27335000	1.08064600	-0.76406200
H	-0.17508000	1.63775300	-1.54805000
H	0.39570000	3.67524300	1.18007500
H	-0.21125900	3.71611300	-0.48173200
H	1.97156800	2.64697200	-1.25593700
H	2.14642900	4.25866400	-0.60690800
H	2.53575300	1.83940800	1.21044000
H	3.07674900	3.47695800	1.46136800
C	4.26934400	1.20096200	-0.93710400
C	3.59331700	-0.07172000	-0.41964900
C	3.69864400	-1.26292100	-1.37822800
C	2.81876100	-2.44900300	-0.96160800
C	1.32872000	-2.21699500	-1.24766500
C	0.41706200	-3.23235300	-0.59721000
C	-0.92347900	-3.21680600	-0.63258900
C	-1.77566300	-2.20301600	-1.34132900
C	-2.66784200	-1.37843200	-0.39934200
C	-3.59271700	-0.41085200	-1.13816800
C	-4.40249700	0.48004100	-0.19458900
C	-5.32197800	1.46428900	-0.92181200
C	-6.11868200	2.35488700	0.03268600

H	-6.75478200	1.75631400	0.69135300
H	-5.45386900	2.95058600	0.66616700
H	-6.76488200	3.04620600	-0.51298900
H	-6.01116600	0.90438400	-1.56383200
H	-4.72131300	2.08922100	-1.59395300
H	-5.00463000	-0.14918500	0.47300400
H	-3.71473700	1.04019800	0.45380000
H	-2.99104400	0.21835100	-1.80548600
H	-4.27242100	-0.97843900	-1.78382900
H	-3.26699000	-2.05300200	0.22466400
H	-2.03448100	-0.79199400	0.27544400
H	-1.15456500	-1.52484500	-1.93053800
H	-2.41937300	-2.73534700	-2.05192300
H	-1.45878200	-4.02654000	-0.13893400
H	0.90524500	-4.06374100	-0.09073600
H	1.03672800	-1.20743900	-0.93712400
H	1.17544600	-2.23337700	-2.33315500
H	3.14054600	-3.35592700	-1.48188600
H	2.96893200	-2.65023600	0.10716600
H	3.42076400	-0.94639700	-2.39108800
H	4.74350700	-1.58156800	-1.43691000
H	4.03664600	-0.34969900	0.54607200
H	2.53629000	0.13888500	-0.22173200
H	3.76952500	1.51827400	-1.85981500
H	5.29905500	0.96968400	-1.22792400
H	4.73494900	3.23946800	-0.39556600
H	4.97683000	2.08593900	0.89751000
N	-0.06386700	-1.48979300	2.05033100
C	-1.14890200	-1.82656100	3.01511800
H	-1.03480000	-2.86131900	3.33485600
H	-1.07631400	-1.16028600	3.87295100
H	-2.10878500	-1.69221000	2.52136800
C	1.31102200	-1.63974700	2.60528100
H	2.02859800	-1.34732700	1.84192300
H	1.47248800	-2.67802400	2.89086200
H	1.41165100	-0.99206700	3.47438200
H	-0.17917400	-0.50315400	1.72804200
H	-0.15677900	-2.09026900	1.20861200

conf_31

C	4.20652500	-1.17261600	-0.61046800
C	4.11134500	-0.08968700	0.47396900
C	3.46619200	1.20955300	-0.04865000
C	2.74212200	2.08558100	0.99726900
C	1.33962600	1.63134400	1.30644600
O	0.36345500	2.35743200	1.31218200
O	1.13917800	0.32930400	1.59073000
H	1.95768900	-0.18408600	1.50580400
H	3.30526400	2.10040600	1.93841400
H	2.65936700	3.11611000	0.65522300
H	2.76862100	0.98224300	-0.85751300
H	4.24916600	1.82065700	-0.50175300
H	3.58239300	-0.50686500	1.34583500

H	5.10527400	0.14152700	0.86677100
C	2.85875200	-1.83099600	-0.95066600
C	2.40814800	-2.88801800	0.06756500
C	0.98829700	-3.41872700	-0.18119200
C	-0.11322300	-2.48161500	0.32417200
C	-1.52713500	-2.93662100	-0.07344200
C	-2.60721500	-2.15204600	0.61411200
C	-3.55746900	-1.39175600	0.05262200
C	-3.78017400	-1.12226600	-1.41086800
C	-3.28771000	0.27331900	-1.84574300
C	-1.76236700	0.37889500	-1.88134800
C	-1.22325500	1.79720800	-2.06755200
C	0.30044100	1.82671000	-2.20378200
C	0.89054400	3.23592000	-2.17949900
H	0.65850900	3.74090300	-1.23745100
H	1.97745400	3.21714800	-2.29607100
H	0.48594400	3.84500200	-2.99218500
H	0.58747900	1.31677200	-3.12996300
H	0.73039500	1.22993800	-1.39076400
H	-1.68080800	2.26004300	-2.94895100
H	-1.51812600	2.42177600	-1.21330900
H	-1.33821600	-0.03994800	-0.96044500
H	-1.38460500	-0.26246400	-2.68624600
H	-3.68932900	0.50276400	-2.83690500
H	-3.70799700	1.03210500	-1.17302200
H	-3.29023600	-1.88108100	-2.02543500
H	-4.85214400	-1.20179400	-1.61430400
H	-4.28795000	-0.93045000	0.71855600
H	-2.62384000	-2.25976400	1.69874900
H	-1.63291900	-2.88355900	-1.15885500
H	-1.64564200	-3.99222000	0.20068000
H	-0.04625400	-2.41606700	1.41771700
H	0.05129300	-1.46836800	-0.05391700
H	0.85502400	-3.59852400	-1.25458900
H	0.86983700	-4.39127500	0.30689100
H	3.11964900	-3.71877900	0.03491600
H	2.46717800	-2.49584100	1.09263600
H	2.08529800	-1.06186400	-1.06514500
H	2.93396300	-2.31400200	-1.92951900
H	4.63412700	-0.72016800	-1.51113900
H	4.91337600	-1.94566800	-0.29404100
N	-2.01983300	1.02744100	1.72537200
C	-3.11678700	2.03138900	1.62450100
H	-4.06729300	1.55108000	1.85035500
H	-2.92283700	2.83171200	2.33644800
H	-3.13183300	2.43294500	0.61383800
C	-1.87716100	0.42115400	3.07982900
H	-1.67449300	1.21443600	3.79736600
H	-1.04480700	-0.27813300	3.05529900
H	-2.80014800	-0.09321400	3.34179200
H	-1.11347100	1.49415900	1.48188000
H	-2.18356700	0.26608400	1.04779600

conf_44

C	4.83091100	1.99220700	-1.19615100
C	4.57518100	0.52227500	-1.56315300
C	5.17093500	-0.45230100	-0.53875400
C	4.58453800	-1.88031600	-0.63946700
C	3.15439600	-1.91317500	-0.17600000
O	2.18895100	-2.00562600	-0.90938400
O	2.92902300	-1.79037600	1.14804600
H	3.75726600	-1.72950600	1.64327600
H	4.60878600	-2.23289000	-1.67051400
H	5.17178000	-2.57410200	-0.02881000
H	5.02692800	-0.06757300	0.47716300
H	6.25193900	-0.52135500	-0.68162000
H	3.49631700	0.35627100	-1.65473000
H	4.99715100	0.29564800	-2.54675200
C	3.87732800	2.50025300	-0.10431600
C	2.46810100	2.81680400	-0.63107200
C	1.38592800	2.80212900	0.45638200
C	0.97195300	1.38239700	0.85382000
C	-0.02815000	1.33443600	2.01858000
C	-0.39003500	-0.07098600	2.40586800
C	-1.62070800	-0.59039000	2.51775900
C	-2.92537100	0.11601600	2.26884200
C	-3.46429500	-0.12913400	0.84910400
C	-4.84074800	0.48553100	0.59438000
C	-5.34283000	0.25618000	-0.83273600
C	-6.72232300	0.86378000	-1.09999200
C	-7.21111800	0.62882900	-2.52990900
H	-7.28868300	-0.44026600	-2.75024000
H	-8.19623500	1.07222900	-2.69125800
H	-6.52531100	1.07038000	-3.25955800
H	-6.68590800	1.93982800	-0.89527100
H	-7.44315000	0.44207400	-0.39041100
H	-4.62103900	0.67914200	-1.54459700
H	-5.38057600	-0.82252500	-1.03615000
H	-5.55991300	0.06703700	1.30774400
H	-4.79777600	1.56182300	0.79831000
H	-2.74834000	0.28630800	0.12789000
H	-3.51565900	-1.21080500	0.66155700
H	-3.66318100	-0.23847000	2.99524200
H	-2.82319700	1.19136800	2.43101900
H	-1.70197100	-1.63132300	2.82876500
H	0.45677000	-0.71682400	2.63752100
H	0.42730800	1.82986200	2.88520000
H	-0.91865500	1.91390400	1.76436700
H	0.53241600	0.89359700	-0.02667300
H	1.85466400	0.79111700	1.11896200
H	1.74525100	3.34018700	1.34151000
H	0.50074900	3.34466500	0.10803600
H	2.48968400	3.79447200	-1.12122900
H	2.18858800	2.09844400	-1.41191600
H	3.81632400	1.75523400	0.69839800
H	4.28939400	3.39967600	0.36220300
H	5.87089200	2.10701700	-0.87271100
H	4.72046400	2.61742500	-2.08758300

N	-0.48378600	-1.87216400	-0.33585300
C	-0.93168600	-3.24853500	0.01713300
H	-0.62922500	-3.93121500	-0.77505400
H	-0.46144900	-3.53826700	0.95517600
H	-2.01477600	-3.25646700	0.12588100
C	-1.05435600	-1.35734500	-1.61308400
H	-0.69509500	-0.34215300	-1.77151600
H	-2.14041300	-1.36274700	-1.55273600
H	-0.71607700	-1.99655500	-2.42669900
H	0.55906400	-1.87407500	-0.42319300
H	-0.71829700	-1.23934900	0.44927600

conf_268

C	-2.42774500	-3.53997000	-0.29659300
C	-2.23331300	-2.51449800	-1.42437700
C	-3.50313500	-1.68983600	-1.67137200
C	-3.25715400	-0.43496200	-2.51080900
C	-2.59598200	0.67118900	-1.72768300
O	-2.63319000	0.76288300	-0.51642900
O	-1.96277100	1.63971100	-2.41686200
H	-1.98614000	1.46871800	-3.36835100
H	-2.66170400	-0.66327000	-3.40206200
H	-4.20557500	-0.01634000	-2.87245200
H	-3.94844500	-1.38622700	-0.72107600
H	-4.24334200	-2.31167500	-2.18098200
H	-1.39888200	-1.85003900	-1.16546300
H	-1.94834700	-3.01535000	-2.35519400
C	-2.21998200	-2.92701300	1.09838900
C	-0.74371400	-2.91531600	1.51707400
C	-0.45876700	-2.07508100	2.77067600
C	-0.32120400	-0.56775700	2.50846100
C	0.95235300	-0.21429500	1.71816300
C	1.29722100	1.24635700	1.72518600
C	1.48786000	2.04133400	0.66114200
C	1.38018800	1.65883800	-0.79016700
C	2.75907800	1.41464500	-1.43942400
C	3.47289800	0.17371900	-0.90161000
C	4.84624500	-0.04888300	-1.54074100
C	5.54552600	-1.33579600	-1.08433100
C	5.87886400	-1.37090900	0.40979300
H	4.97823600	-1.36251200	1.02997700
H	6.44135300	-2.27208100	0.66501400
H	6.48807300	-0.50765500	0.69585600
H	6.46933400	-1.45300400	-1.65944900
H	4.91712200	-2.19742700	-1.34069800
H	4.73350500	-0.07150100	-2.63089600
H	5.49004800	0.81109000	-1.31728100
H	3.58083700	0.26126900	0.18321700
H	2.84547000	-0.70983900	-1.08003400
H	2.62083900	1.31842200	-2.52166400
H	3.39050200	2.29767700	-1.28761500
H	0.76235400	0.76441500	-0.91227500
H	0.87728800	2.46448900	-1.33698600

H	1.82304200	3.06104700	0.84963200
H	1.46196900	1.67720800	2.71249100
H	1.79502200	-0.74708100	2.17739200
H	0.88202700	-0.58936800	0.69710600
H	-1.20190000	-0.20575800	1.96292400
H	-0.30180300	-0.04063200	3.46945700
H	-1.25620900	-2.24447800	3.50212800
H	0.46559600	-2.42187500	3.24458400
H	-0.43265600	-3.94894900	1.69741400
H	-0.12279700	-2.56397500	0.68500200
H	-2.62577700	-1.90806400	1.11244800
H	-2.78813800	-3.48940100	1.84569700
H	-3.43190700	-3.96862200	-0.37854000
H	-1.73253800	-4.37503100	-0.42636100
N	-1.55559400	2.72544100	1.05877700
C	-2.25276300	2.76878000	2.37415800
H	-1.76951000	3.50691800	3.01279800
H	-3.29478900	3.04110500	2.21471100
H	-2.19291600	1.78406300	2.83295700
C	-1.54401200	4.02202800	0.32585000
H	-1.05440700	3.87260400	-0.63420800
H	-1.00446700	4.76359800	0.91291500
H	-2.57048500	4.34897400	0.16751100
H	-1.99177200	1.99690900	0.44864200
H	-0.57602900	2.40735800	1.19589100

conf_115

C	4.87227600	-0.56987000	0.34902600
C	3.88043100	-0.40461100	-0.80973200
C	3.05257900	-1.65435500	-1.11950000
C	2.01620000	-1.42908700	-2.25043400
C	0.97054100	-0.42809600	-1.84407400
O	1.04888000	0.76858900	-2.05545100
O	-0.09684800	-0.87292300	-1.15725200
H	-0.10058900	-1.83785200	-1.06144100
H	2.51672300	-1.03825600	-3.13713500
H	1.53896200	-2.37716500	-2.51491200
H	2.54307800	-2.01918800	-0.22256100
H	3.71295700	-2.46314000	-1.44380500
H	3.21862400	0.44169600	-0.60310200
H	4.43511400	-0.12897300	-1.71305400
C	4.25552600	-0.70566600	1.75222900
C	3.29065000	0.43730100	2.13299100
C	1.81435400	0.06422800	1.95813900
C	0.84674400	1.24621300	2.00601700
C	-0.61625000	0.78322700	1.95930900
C	-1.61900000	1.89356900	1.82457200
C	-2.73956500	1.85179600	1.08668700
C	-3.20912800	0.67037500	0.27417200
C	-3.91989500	-0.38317700	1.14718700
C	-4.46481200	-1.57946000	0.35626000
C	-3.42240000	-2.37821300	-0.43967500
C	-2.26276500	-2.92416000	0.39957000

C	-1.34920000	-3.86456900	-0.39282100
H	-1.02888700	-3.42036300	-1.34711000
H	-0.46100800	-4.15226800	0.17622100
H	-1.87283400	-4.78319000	-0.66864600
H	-2.65937900	-3.46402100	1.26607200
H	-1.67793900	-2.09438100	0.81108600
H	-3.93209100	-3.21557400	-0.92836000
H	-3.01765300	-1.76320600	-1.25324700
H	-4.96497900	-2.25371000	1.05969600
H	-5.24199500	-1.23053400	-0.33281900
H	-3.22447900	-0.72497800	1.91935300
H	-4.74639200	0.09762500	1.67906900
H	-3.90243800	1.00847100	-0.50325700
H	-2.36508000	0.19576900	-0.23555500
H	-3.41198900	2.70547700	1.13658800
H	-1.43168400	2.78322200	2.42361800
H	-0.74155800	0.06076500	1.14923400
H	-0.83169600	0.23576500	2.88595400
H	1.01808000	1.84365100	2.90821200
H	1.06093600	1.90960100	1.15738800
H	1.67218900	-0.45267100	1.00498600
H	1.53877800	-0.66199100	2.73213400
H	3.45275500	0.73411600	3.17323700
H	3.52146700	1.32529700	1.53157100
H	3.73544200	-1.66581900	1.84899100
H	5.07951900	-0.74801600	2.46899300
H	5.51822700	-1.43341200	0.15527900
H	5.53131900	0.30483800	0.35071600
N	-0.68173800	2.71613200	-1.20959000
C	-1.72609300	2.82620800	-2.26674500
H	-2.24274200	1.87205300	-2.34570300
H	-2.43196200	3.60957700	-1.99493100
H	-1.24573400	3.06806300	-3.21329500
C	0.13056000	3.95202600	-1.02849100
H	0.65474900	4.16932000	-1.95746200
H	0.85036400	3.78387800	-0.22969500
H	-0.52729900	4.77950800	-0.76671400
H	-0.04577600	1.92046600	-1.45471800
H	-1.14170000	2.47924100	-0.31023800

conf_72

C	-4.31691300	0.89755700	-0.29629400
C	-5.42584500	-0.16220900	-0.29118000
C	-4.98118300	-1.63048500	-0.32639400
C	-4.06734800	-2.04937800	0.85234600
C	-2.59280200	-1.89591500	0.60370300
O	-1.82376900	-1.27063800	1.30666400
O	-2.07714800	-2.53548800	-0.46961200
H	-2.76810600	-2.97702200	-0.98260400
H	-4.30879700	-1.48970000	1.75555600
H	-4.22503600	-3.11085500	1.07843600
H	-4.49848900	-1.84775100	-1.28669500
H	-5.87348300	-2.25911800	-0.29804400

H	-6.05058400	-0.01451400	0.59624400
H	-6.07986300	0.00508200	-1.15337900
C	-3.35743900	0.82201800	-1.49221100
C	-2.63425200	2.14593100	-1.79104900
C	-1.91797400	2.79845700	-0.60189200
C	-0.83033000	1.93832100	0.04435700
C	-0.07997500	2.68836300	1.16144500
C	0.90374100	1.82537500	1.90221100
C	2.22599900	1.74491700	1.69084900
C	3.01991100	2.51434100	0.67199000
C	3.46066600	1.67637300	-0.54468400
C	4.36739800	0.49165400	-0.20337000
C	4.87244000	-0.25355300	-1.44221700
C	5.81026700	-1.42886700	-1.13994100
C	5.16046000	-2.56639700	-0.34691800
H	5.84428700	-3.41084800	-0.23785600
H	4.88054800	-2.24993500	0.66240000
H	4.26230600	-2.93796900	-0.85378200
H	6.68561200	-1.05979400	-0.59419400
H	6.18836900	-1.82615900	-2.08663200
H	4.01764000	-0.61512600	-2.03106100
H	5.39330200	0.46001000	-2.08927600
H	5.22782700	0.85078600	0.37404400
H	3.83851900	-0.20169400	0.46116000
H	2.56930000	1.32228500	-1.07907300
H	3.98542100	2.33544200	-1.24353200
H	2.44847800	3.37401300	0.31700600
H	3.91533700	2.91396700	1.16077200
H	2.79880300	1.08151700	2.33678600
H	0.48094200	1.21750900	2.70206900
H	0.41736700	3.55977400	0.72945200
H	-0.81462900	3.07192600	1.87678800
H	-1.27761600	1.02827000	0.45664100
H	-0.10754800	1.62763000	-0.72209100
H	-1.47126100	3.73910600	-0.94135300
H	-2.64893900	3.07472200	0.16544400
H	-1.91506100	1.98283100	-2.60220200
H	-3.36881300	2.85978200	-2.17856000
H	-2.61923200	0.02723600	-1.33608900
H	-3.91779100	0.53462800	-2.38931100
H	-4.80640900	1.87706700	-0.29606600
H	-3.75129100	0.85253800	0.64082200
N	0.85364800	-1.09986700	0.61593800
C	1.04787200	-1.36881400	-0.83584000
H	2.10997400	-1.32973600	-1.06627300
H	0.64263300	-2.35187100	-1.06550000
H	0.51203900	-0.61103200	-1.40365200
C	1.56031700	-2.05816300	1.51163500
H	1.16754600	-3.05809100	1.33492500
H	1.37948100	-1.76821600	2.54522700
H	2.62602500	-2.03516200	1.29424600
H	-0.16707600	-1.13414900	0.84033900
H	1.15999100	-0.13645300	0.84160800

conf_90

C	4.54129300	0.28921100	-0.16487600
C	4.84828900	-1.04467500	0.52080000
C	4.92692100	-2.25025600	-0.42464900
C	3.68714500	-2.42231800	-1.33615400
C	2.39119800	-2.31777600	-0.57820900
O	1.56770400	-1.44114500	-0.76068100
O	2.14167600	-3.23248000	0.37639200
H	2.86539000	-3.86977300	0.45235700
H	3.66206900	-1.65523700	-2.10766200
H	3.72591900	-3.39528300	-1.83748200
H	5.09148100	-3.16133300	0.16208700
H	5.79498800	-2.16055100	-1.08293100
H	5.79297500	-0.97202400	1.06852900
H	4.07972300	-1.23100800	1.28278900
C	4.45935900	1.46132500	0.82409900
C	3.62976600	2.63933600	0.29701800
C	2.12025200	2.37214800	0.33381500
C	1.28542400	3.51109300	-0.25344500
C	-0.22725800	3.28636800	-0.15628100
C	-0.75529700	2.18189900	-1.03799400
C	-2.03546400	1.78457100	-1.06671200
C	-3.12883100	2.37463400	-0.20937800
C	-4.48799400	1.68228700	-0.34893000
C	-4.48705900	0.22270400	0.10909800
C	-5.87874700	-0.41667100	0.10206100
C	-5.89420500	-1.89650900	0.50635700
C	-5.27546000	-2.83465000	-0.53413900
H	-5.81050700	-2.76653200	-1.48560800
H	-4.22684100	-2.59601500	-0.73471400
H	-5.31832900	-3.87555000	-0.20454300
H	-6.92951400	-2.20316900	0.68179800
H	-5.38145500	-2.01452200	1.46993700
H	-6.52395800	0.15029600	0.78139200
H	-6.31905400	-0.31071300	-0.89672200
H	-3.81516300	-0.35672600	-0.53442100
H	-4.07755000	0.16759100	1.12889300
H	-5.22806500	2.24069600	0.23239300
H	-4.81738100	1.73421700	-1.39292500
H	-2.82270100	2.37033600	0.84584200
H	-3.23597200	3.43579100	-0.46700200
H	-2.33496300	1.05328600	-1.81603900
H	-0.05736800	1.74294400	-1.74867300
H	-0.50857100	3.10601000	0.89019700
H	-0.74679700	4.21375500	-0.42865700
H	1.53709500	4.44165700	0.26459400
H	1.56068400	3.66324200	-1.30351200
H	1.89508100	1.43871700	-0.19671900
H	1.82420200	2.21733200	1.38102300
H	3.84116700	3.53376100	0.89166000
H	3.93613200	2.87533200	-0.72915700
H	4.01529200	1.11351600	1.76600100
H	5.46975500	1.79671600	1.07529000
H	5.30340800	0.50260800	-0.92310300

H	3.59266900	0.21031800	-0.70182900
N	-0.69626100	-0.71708800	0.58615000
C	-0.38896800	-0.22697100	1.95862600
H	0.11820000	-1.01773000	2.50924600
H	0.25748600	0.64422900	1.87888500
H	-1.31819100	0.03951900	2.45961900
C	-1.57225600	-1.92250100	0.55424500
H	-1.76686000	-2.18559300	-0.48355600
H	-2.50813200	-1.70623500	1.06310000
H	-1.05016200	-2.74091300	1.04752600
H	0.19537400	-0.96422800	0.09937700
H	-1.11142300	0.06038900	0.04187500

conf_171

C	4.65818000	-1.48955500	-0.88058900
C	4.82001300	-0.55627400	0.33806500
C	5.11535600	0.90913400	-0.02046300
C	4.06433700	1.61397400	-0.89084100
C	2.68760000	1.76420300	-0.29482900
O	1.67016400	1.79764100	-0.96231400
O	2.57799900	1.90220000	1.03588700
H	3.44450300	1.84211700	1.46375400
H	4.39906300	2.63379400	-1.11954900
H	3.93550600	1.11610200	-1.85174000
H	6.06321600	0.95712200	-0.56349500
H	5.29649000	1.48717900	0.89751300
H	3.92508400	-0.61813600	0.96888000
H	5.64392900	-0.90482000	0.96564600
C	3.19982600	-1.75372300	-1.28640500
C	2.47674300	-2.71713400	-0.33513100
C	0.95789500	-2.77116000	-0.55220800
C	0.21836000	-1.53286800	-0.02387800
C	0.08974300	-1.50392200	1.51293900
C	-0.28610400	-0.14793200	2.03752000
C	-1.44480300	0.22936500	2.59520800
C	-2.66660300	-0.60306600	2.85883800
C	-3.86031200	-0.21382000	1.96351100
C	-3.73373000	-0.73371800	0.53064800
C	-4.82112100	-0.21705900	-0.41217800
C	-4.74992400	-0.81874200	-1.81821700
C	-5.80121500	-0.24663700	-2.77017900
H	-5.68183200	0.83486600	-2.89107900
H	-5.73323400	-0.70186200	-3.76076700
H	-6.81138100	-0.42476900	-2.39150300
H	-4.87053800	-1.90512700	-1.74582800
H	-3.74806400	-0.65893900	-2.23758100
H	-5.80590100	-0.42912100	0.01944300
H	-4.76234300	0.87929800	-0.47595400
H	-2.74714200	-0.47481100	0.12560600
H	-3.75754400	-1.82958200	0.54707300
H	-4.78115200	-0.60684600	2.40397200
H	-3.97142000	0.87858900	1.95924600
H	-2.45339400	-1.66743100	2.73481200

H	-2.94915700	-0.46102300	3.90700900
H	-1.52364200	1.26321400	2.93306200
H	0.50283600	0.60045200	1.96252300
H	-0.62812200	-2.26459700	1.82589000
H	1.05057600	-1.78318100	1.95819700
H	0.73754200	-0.63092000	-0.36638600
H	-0.78812900	-1.49684200	-0.45459100
H	0.75507500	-2.88620700	-1.62247700
H	0.54634500	-3.66231400	-0.06699100
H	2.89936500	-3.71784900	-0.46746400
H	2.68783100	-2.44496100	0.70527200
H	2.65315500	-0.80704200	-1.34707100
H	3.17415300	-2.17566000	-2.29639400
H	5.21102800	-1.07306700	-1.72970200
H	5.13411400	-2.45042700	-0.66423800
N	-0.99625500	1.85233200	-0.33359700
C	-1.66098900	1.52816400	-1.62731700
H	-2.73822000	1.49314700	-1.48069400
H	-1.40308800	2.29655100	-2.35407800
H	-1.29935000	0.56114400	-1.96989100
C	-1.41972000	3.14906900	0.26410300
H	-2.48902300	3.11803500	0.46693600
H	-1.19551000	3.95145300	-0.43670900
H	-0.86924700	3.30239200	1.19058100
H	0.03890700	1.86238200	-0.48487500
H	-1.16399800	1.10284200	0.35865200

conf_166

C	4.64803500	0.98274600	0.13056000
C	3.84669200	-0.26920800	-0.23829700
C	4.36806100	-1.56204600	0.38982800
C	3.61288300	-2.81099500	-0.11499800
C	2.12235000	-2.69455400	0.06314900
O	1.31640000	-2.72504500	-0.84683100
O	1.65439100	-2.52355000	1.31582000
H	2.37584700	-2.49469600	1.95970500
H	3.79312900	-2.96431500	-1.17860900
H	3.95621100	-3.70662700	0.41472700
H	4.31124000	-1.49251800	1.48381700
H	5.42730200	-1.70093400	0.15836300
H	2.80316700	-0.12523700	0.06124200
H	3.83872200	-0.37792600	-1.32956600
C	3.97668400	2.26527500	-0.38266100
C	2.76351800	2.69298000	0.45888200
C	1.80005900	3.64376600	-0.26846500
C	0.96799700	3.00973300	-1.39666800
C	-0.26159200	2.19873400	-0.93394600
C	0.07582000	0.92732500	-0.21080100
C	-0.33773600	0.53218300	1.00132300
C	-1.26098100	1.25818000	1.93809500
C	-2.63822400	0.57809600	2.06717000
C	-3.48354900	0.65978700	0.79463600
C	-4.83217300	-0.05565000	0.91376400

C	-5.60146100	-0.17315900	-0.40798300
C	-5.97938600	1.17148900	-1.03412400
H	-6.56392100	1.77531700	-0.33388100
H	-5.09842000	1.75311500	-1.31795900
H	-6.58355900	1.03027300	-1.93305800
H	-6.50967700	-0.75867600	-0.23538100
H	-5.00329800	-0.75283700	-1.12591500
H	-4.67333500	-1.05941100	1.32861700
H	-5.45301600	0.47006900	1.64799300
H	-3.64048400	1.71256600	0.54017200
H	-2.92615200	0.24235100	-0.05585700
H	-2.50051800	-0.47202700	2.35706900
H	-3.18677300	1.04533800	2.89062700
H	-0.79162600	1.29082400	2.92703500
H	-1.39944900	2.29502700	1.62455000
H	0.05340700	-0.41147000	1.38364900
H	0.77227100	0.27867500	-0.74294000
H	-0.90751200	2.83299900	-0.32302500
H	-0.84468300	1.94194900	-1.82865300
H	0.60140900	3.80534700	-2.05127000
H	1.60254200	2.37522300	-2.02580400
H	1.11652400	4.09473800	0.45986500
H	2.37935200	4.47356100	-0.68761800
H	2.21278400	1.81328200	0.79847800
H	3.12756200	3.18350100	1.36769300
H	4.70309500	3.08294000	-0.39993600
H	3.68060500	2.10730600	-1.42620500
H	4.76665200	1.04188500	1.21955900
H	5.65691300	0.89511600	-0.28519800
N	-1.32208700	-1.95806500	-0.77532100
C	-2.17587900	-2.81494400	0.09358300
H	-3.17992900	-2.39762000	0.13290600
H	-2.20337800	-3.82111500	-0.32153400
H	-1.74176800	-2.83854600	1.09147300
C	-1.80382900	-1.82903500	-2.17922700
H	-1.82156100	-2.81524000	-2.63985900
H	-1.11971700	-1.18027600	-2.72331900
H	-2.80420100	-1.39903200	-2.17538500
H	-0.34287000	-2.32677900	-0.77494300
H	-1.23833000	-1.01512100	-0.35920900

conf_130

C	-5.28933900	0.95101800	-1.41307100
C	-5.72065800	0.24269400	-0.11114300
C	-5.78509100	-1.29003000	-0.21170200
C	-4.49722100	-1.98725200	-0.67372100
C	-3.27805700	-1.80961400	0.19680700
O	-2.14389500	-1.81904800	-0.24735600
O	-3.44601900	-1.66950200	1.51651600
H	-4.38602200	-1.65931700	1.74738500
H	-4.66521700	-3.07013000	-0.73115700
H	-4.20882700	-1.67272000	-1.67668000
H	-6.56758700	-1.56737100	-0.92348000

H	-6.12223200	-1.71054600	0.74653100
H	-5.04993400	0.53850900	0.70373600
H	-6.71327200	0.58651800	0.18965000
C	-3.80434700	1.34778100	-1.44966000
C	-3.49309900	2.55586600	-0.55656800
C	-1.99858100	2.83139400	-0.35302000
C	-1.30814300	1.82325200	0.57140700
C	0.14913600	2.19412500	0.88122800
C	0.78394200	1.30869900	1.91833500
C	2.05434000	0.88011500	1.96018200
C	3.14890900	1.16986400	0.97165000
C	4.11399800	-0.00556300	0.76883500
C	5.22794400	0.29262500	-0.23526200
C	6.17925700	-0.88926600	-0.44203700
C	7.27376200	-0.64131800	-1.48829900
C	8.26056800	0.46592400	-1.10858200
H	8.72917800	0.25829700	-0.14152300
H	9.05746500	0.55046600	-1.85093800
H	7.77465600	1.44266100	-1.04068400
H	6.80511900	-0.40241200	-2.45071700
H	7.82483200	-1.57446000	-1.64233300
H	6.64774100	-1.14625900	0.51625700
H	5.59391100	-1.76809500	-0.74157800
H	4.78550900	0.57484200	-1.19984600
H	5.78668200	1.16836900	0.10969900
H	4.55389100	-0.28171800	1.73402000
H	3.55783000	-0.89117100	0.43229500
H	2.73481000	1.48724300	0.00942100
H	3.72512700	2.02890400	1.33943700
H	2.35835100	0.29983000	2.83068200
H	0.13977100	1.04874300	2.75771300
H	0.74125800	2.21412700	-0.03897100
H	0.16712300	3.22374400	1.26220100
H	-1.86606000	1.76382200	1.51368000
H	-1.35522500	0.82029300	0.13159600
H	-1.48954900	2.85507100	-1.32469900
H	-1.87752600	3.83123500	0.07715300
H	-3.96715300	3.43916600	-0.99623400
H	-3.95887000	2.42558000	0.42765600
H	-3.18301000	0.49526900	-1.15903600
H	-3.51760700	1.58938200	-2.47874000
H	-5.52684000	0.31222900	-2.27068500
H	-5.88901400	1.85593800	-1.54747200
N	0.48573200	-1.46052400	0.29508200
C	0.89874600	-1.12851700	-1.09899000
H	0.56543700	-1.92464400	-1.76177900
H	0.42445800	-0.19178100	-1.38494200
H	1.98146500	-1.02871100	-1.13946500
C	1.06801100	-2.72692100	0.82155400
H	0.70417800	-2.88393900	1.83562600
H	2.15373400	-2.65044900	0.82444800
H	0.75160500	-3.55067800	0.18399500
H	-0.55462200	-1.53931200	0.30343400
H	0.74914300	-0.67370400	0.91782300

conf_249

C	-5.44630000	0.10215100	-0.58089900
C	-4.34496200	-0.74180900	-1.23800700
C	-4.43354400	-2.23315000	-0.88836800
C	-3.90847400	-2.55385200	0.53600500
C	-2.43635600	-2.26817300	0.63679900
O	-1.95273500	-1.27644100	1.14349700
O	-1.59162700	-3.15779700	0.06175400
H	-2.07111100	-3.91349700	-0.30419600
H	-4.41452300	-1.93984400	1.27971100
H	-4.09688600	-3.60416500	0.77894800
H	-3.88123000	-2.82917600	-1.62293500
H	-5.47194300	-2.57118600	-0.94125000
H	-4.40546300	-0.62270500	-2.32391800
H	-3.36054200	-0.36446300	-0.94748900
C	-5.16073000	1.61007200	-0.55129500
C	-4.00418600	1.99228300	0.39750800
C	-2.66102300	2.28732800	-0.28264900
C	-1.51201200	2.32280700	0.72415400
C	-0.15782200	2.71884900	0.12474700
C	0.98288800	2.50628600	1.08758900
C	2.28061800	2.39220100	0.77309700
C	2.87988300	2.49075500	-0.60592700
C	4.29712500	1.91128800	-0.69883200
C	4.35975900	0.40544500	-0.44160200
C	5.77336800	-0.17633700	-0.46724100
C	5.81045200	-1.68211700	-0.19315200
C	7.22403100	-2.26503400	-0.22892700
H	7.68690500	-2.11074800	-1.20765000
H	7.86411600	-1.78879900	0.51908200
H	7.21923900	-3.33895100	-0.02832600
H	5.18341800	-2.19859600	-0.93130300
H	5.36052000	-1.88004000	0.78880800
H	6.23125600	0.02759500	-1.44214700
H	6.39108200	0.34324000	0.27461300
H	3.90614700	0.18805400	0.53489500
H	3.75116000	-0.11182200	-1.19634700
H	4.71093300	2.12820300	-1.68808000
H	4.94072700	2.42826600	0.02210000
H	2.22657900	2.01533500	-1.34815500
H	2.90867900	3.55063300	-0.88843000
H	2.99119500	2.29747500	1.59230500
H	0.71523900	2.50907800	2.14315000
H	0.02123100	2.17431100	-0.81119700
H	-0.18906000	3.77535300	-0.16991700
H	-1.75499100	3.01857300	1.53517200
H	-1.44058200	1.33585800	1.19741000
H	-2.44722400	1.52973000	-1.04639300
H	-2.72220400	3.24141400	-0.81865800
H	-4.28446700	2.87068300	0.98649500
H	-3.85925500	1.18364700	1.12421100
H	-4.95227700	1.97053400	-1.56554900
H	-6.07436800	2.11973900	-0.23515900

H	-6.38866500	-0.08755900	-1.10518900
H	-5.60429300	-0.22897900	0.45248600
N	0.70219400	-0.66652400	0.58951400
C	0.69217100	-0.85116700	-0.89098400
H	0.02976500	-0.10678000	-1.32805300
H	1.70227400	-0.72427800	-1.27184200
H	0.32272500	-1.85080600	-1.10867700
C	1.55144700	-1.65776800	1.31244600
H	1.53316700	-1.42432400	2.37561500
H	2.56963200	-1.60102500	0.93387600
H	1.13848400	-2.65056900	1.14306300
H	-0.27984200	-0.77579500	0.92104000
H	1.01601400	0.29562400	0.81250700

9Z_NH4

conf_0

C	3.16079100	-0.95905900	-1.49293000
C	3.94420400	-0.65948600	-0.21074500
C	4.46133900	0.78088200	-0.10775200
C	3.34953300	1.85748300	-0.12352600
C	2.28116900	1.58283300	0.89814500
O	1.12328900	1.32619500	0.61547100
O	2.62729000	1.58409100	2.19352500
H	3.56683100	1.78233100	2.31112000
H	2.85942100	1.90203300	-1.09418100
H	3.78416600	2.84284100	0.07552500
H	5.06737600	0.88508300	0.79964300
H	5.13395300	1.00070100	-0.94101000
H	4.80161600	-1.33701200	-0.14655600
H	3.32617800	-0.88331900	0.66727100
C	2.69991200	-2.42004800	-1.60951500
C	1.79809200	-2.91629800	-0.46982000
C	0.52070500	-2.07910500	-0.28500100
C	-0.31507400	-2.57060500	0.86531900
C	-1.59807800	-2.96452400	0.84022900
C	-2.51141100	-3.02450700	-0.35020200
C	-3.91212600	-2.43525400	-0.10106300
C	-3.92450200	-0.96768900	0.34366000
C	-3.15133400	-0.01853900	-0.57619300
C	-3.28727700	1.45903300	-0.19128300
C	-2.27695900	2.37114400	-0.89022500
C	-2.37227100	3.83798400	-0.46445600
C	-1.33477800	4.72416200	-1.15524900
H	-1.46061400	4.70315500	-2.24143200
H	-1.41926100	5.76388200	-0.83157000
H	-0.31784300	4.38528300	-0.93278400
H	-3.37910600	4.21294800	-0.67780500
H	-2.24780700	3.90895900	0.62404200
H	-2.42278700	2.29516300	-1.97391200
H	-1.25750900	2.00709400	-0.70317700
H	-4.30657100	1.79805300	-0.40322100
H	-3.20220700	1.58617800	0.90468000
H	-2.08936900	-0.29489600	-0.59577300

H	-3.49618900	-0.14677200	-1.60778100
H	-3.52515600	-0.89898300	1.36615700
H	-4.96343500	-0.63112900	0.41842500
H	-4.49166800	-2.53628700	-1.02378500
H	-4.42958700	-3.03805100	0.65227800
H	-2.63549900	-4.08106200	-0.61786300
H	-2.05215400	-2.54712700	-1.21697200
H	-2.02631500	-3.33885500	1.76974000
H	0.22214500	-2.67849100	1.80925300
H	-0.05238700	-2.07853200	-1.21489100
H	0.81041700	-1.03729100	-0.10633600
H	1.51611300	-3.95470000	-0.66668500
H	2.35632600	-2.93175500	0.47279300
H	3.57918700	-3.07059800	-1.66320700
H	2.17247100	-2.54293900	-2.56148000
H	2.29182000	-0.29744100	-1.56561100
H	3.79112100	-0.72262800	-2.35711300
N	-0.97347200	0.28262100	1.93894300
H	-0.94190100	0.32770700	2.95685300
H	-1.80476900	0.77590600	1.59805700
H	-0.11037000	0.73094500	1.52298200
H	-1.02857200	-0.71146600	1.63340700

conf_2

C	3.29165000	-2.17898600	-0.16101900
C	4.25384600	-1.35078000	-1.02282000
C	3.84430000	0.11187400	-1.25821600
C	3.73330300	0.95048000	0.02617500
C	2.45892700	0.73833800	0.80037900
O	1.37225200	0.54992300	0.28427600
O	2.50689600	0.80671500	2.14343800
H	3.40933400	0.94667200	2.46076100
H	3.73344500	2.01725300	-0.23104300
H	4.60109500	0.78571000	0.67325700
H	4.59495800	0.58494500	-1.89520900
H	2.89780500	0.17673200	-1.79912500
H	4.36713100	-1.83217000	-1.99953400
H	5.24807600	-1.36610100	-0.56250600
C	1.91238700	-2.39823900	-0.79312700
C	0.84910600	-2.82003300	0.22122600
C	-0.56136200	-2.88448800	-0.38214500
C	-1.65041100	-2.91378300	0.66049700
C	-2.91722600	-2.49804600	0.50459400
C	-3.54304900	-1.97831800	-0.75907700
C	-4.59086200	-0.87392500	-0.54276500
C	-4.05346800	0.40773400	0.10479100
C	-2.91864500	1.07828500	-0.67443500
C	-2.43659000	2.39674200	-0.05946600
C	-1.14074000	2.92050900	-0.68266500
C	-0.55632900	4.13858700	0.03592000
C	0.76346000	4.60879500	-0.57764000
H	1.17094500	5.46885500	-0.04161200
H	1.51261600	3.80999200	-0.55317900

H	0.63048800	4.90079800	-1.62293800
H	-1.28295000	4.95797800	0.02068000
H	-0.40007700	3.89310500	1.09473600
H	-1.32740300	3.16948700	-1.73350800
H	-0.39019800	2.12060100	-0.69519000
H	-3.22701400	3.14975200	-0.14275800
H	-2.29872000	2.28960400	1.03271700
H	-2.06774500	0.39123300	-0.77126900
H	-3.24606500	1.26602600	-1.70252400
H	-3.72298900	0.18201600	1.12968400
H	-4.87788000	1.11839800	0.21979800
H	-5.03547200	-0.62563100	-1.51140900
H	-5.40436300	-1.26948100	0.07414100
H	-4.04211700	-2.82662600	-1.24498800
H	-2.77677900	-1.65133300	-1.46607500
H	-3.59590000	-2.62008400	1.34787500
H	-1.38238400	-3.37305800	1.61172700
H	-0.64103300	-3.77465600	-1.01773600
H	-0.70780700	-2.03345700	-1.05685400
H	1.10449000	-3.78625100	0.66947000
H	0.86363400	-2.09990600	1.04996400
H	1.98903600	-3.14656000	-1.58932800
H	1.57413600	-1.47487900	-1.27050400
H	3.75432900	-3.14714500	0.05347700
H	3.16991300	-1.70252100	0.82130400
N	-0.87868500	-0.00871600	1.69131300
H	-1.33311800	-0.88391100	1.35661100
H	-0.66752800	-0.09655400	2.68507800
H	0.01570400	0.18558700	1.15856500
H	-1.52176700	0.77501500	1.54529400

conf_11

C	2.63167200	-0.37614000	1.73574400
C	4.12791100	-0.62879400	1.50264400
C	4.57332700	-0.79736700	0.04200100
C	4.46816500	0.47920000	-0.80454700
C	3.08041500	0.81312000	-1.28559400
O	2.22678200	-0.01992300	-1.53488900
O	2.78168100	2.10327400	-1.49760900
H	3.51874700	2.68176100	-1.25913200
H	5.06512500	0.36671300	-1.71891100
H	4.89002500	1.33454700	-0.26561600
H	5.62193500	-1.10369700	0.03485200
H	4.01661700	-1.59690300	-0.45159500
H	4.41579800	-1.53341400	2.04788100
H	4.70430100	0.18756600	1.95272800
C	1.73471700	-1.54351100	1.31594800
C	0.24926300	-1.29176000	1.58717100
C	-0.67465600	-2.44495800	1.14701900
C	-0.72203900	-2.63915600	-0.34236500
C	-1.81457200	-2.70897100	-1.11890200
C	-3.25767900	-2.64381100	-0.69551100
C	-3.90635900	-1.26003800	-0.90645200

C	-3.48980400	-0.23331100	0.14811600
C	-4.06420700	1.16475600	-0.09293900
C	-3.75388000	2.16612200	1.03007600
C	-2.26122100	2.38512800	1.31134800
C	-1.48546300	3.00687800	0.14725300
C	0.02286100	3.06890300	0.39358900
H	0.55913500	3.50122700	-0.45406000
H	0.24183000	3.67767700	1.27434900
H	0.43981000	2.07399600	0.58680500
H	-1.70284300	2.45536900	-0.78182300
H	-1.86912400	4.01293200	-0.05114300
H	-1.79228000	1.43645300	1.59784800
H	-2.15533800	3.03863900	2.18322400
H	-4.21957200	3.12753200	0.78855100
H	-4.23490400	1.81838100	1.95032000
H	-3.70642500	1.54918200	-1.05693300
H	-5.15098200	1.08759200	-0.19948800
H	-2.39633800	-0.18673800	0.20785800
H	-3.81293100	-0.58827000	1.13402300
H	-3.66748700	-0.88946100	-1.91296700
H	-4.99425400	-1.37173800	-0.88290600
H	-3.36871200	-2.93342700	0.35225400
H	-3.80802500	-3.38588200	-1.28088500
H	-1.65736500	-2.87920700	-2.18443400
H	0.24259800	-2.77521600	-0.82890000
H	-1.67785500	-2.27700500	1.54059400
H	-0.31036000	-3.37196300	1.60759300
H	0.10232100	-1.11886100	2.65798200
H	-0.06519800	-0.36167200	1.09447400
H	2.04553100	-2.44472700	1.85757400
H	1.88873500	-1.75252200	0.25522200
H	2.48091300	-0.17295200	2.80122700
H	2.31131000	0.53878400	1.22037900
N	-0.43576300	0.19219000	-1.84189500
H	-0.72256600	0.26152100	-2.81814200
H	-0.79719200	-0.69825400	-1.43864900
H	0.62168900	0.19774800	-1.77219600
H	-0.82474400	0.98439900	-1.32230300

conf_19

C	-2.30356100	-1.71243500	-1.67179300
C	-3.27019100	-2.03235600	-0.52274600
C	-4.36801200	-0.98583800	-0.29564100
C	-3.82983600	0.41933600	0.08052100
C	-2.78525200	0.33606300	1.16012700
O	-1.59977200	0.53724800	0.96415100
O	-3.17171700	-0.02996400	2.38994000
H	-4.12754800	-0.17074100	2.43527500
H	-4.65406700	1.05724200	0.41442900
H	-3.36446500	0.90144900	-0.77665100
H	-4.97922000	-0.86816300	-1.19435200
H	-5.04694100	-1.34140700	0.48683000
H	-2.71158600	-2.16258300	0.41107600

H	-3.74579000	-2.99879100	-0.71381600
C	-1.01702100	-2.54951300	-1.63404000
C	-0.07744700	-2.14279800	-0.49429900
C	1.25257500	-2.91127100	-0.47866000
C	2.09361500	-2.58551400	0.72644800
C	3.25816600	-1.91928900	0.74584300
C	4.00055000	-1.36447300	-0.43833100
C	4.73396500	-0.04133300	-0.16007800
C	3.83948400	1.10451000	0.32944500
C	2.63496700	1.39760600	-0.56902700
C	1.82189800	2.62159300	-0.13559900
C	0.48731200	2.75826500	-0.87059600
C	-0.36830200	3.92770800	-0.37941200
C	-1.70683300	4.03036200	-1.11153000
H	-2.29475500	3.11649000	-0.97924900
H	-2.30262800	4.86792800	-0.74176200
H	-1.55949400	4.17600100	-2.18535100
H	-0.54852500	3.81499100	0.69716700
H	0.18889500	4.86337400	-0.49818400
H	-0.08601200	1.82949700	-0.76259600
H	0.68340700	2.87302800	-1.94308600
H	1.63325900	2.60024500	0.95349100
H	2.42304600	3.52612800	-0.27581500
H	2.97780300	1.54819300	-1.59837700
H	1.97647300	0.52057900	-0.61351600
H	4.44686200	2.00997800	0.42573500
H	3.49780900	0.87988100	1.35103100
H	5.52365400	-0.21446500	0.57835200
H	5.23957400	0.26786200	-1.07994700
H	3.33020500	-1.25020800	-1.29240800
H	4.74875200	-2.10686000	-0.74307100
H	3.76728300	-1.81862500	1.70450800
H	1.72782400	-2.99243200	1.67019000
H	1.79962900	-2.71395400	-1.40258900
H	1.04165500	-3.98673200	-0.46917500
H	0.12863200	-1.06953300	-0.58520600
H	-0.58028300	-2.28467400	0.46980200
H	-0.48681300	-2.43553200	-2.58505700
H	-1.26465200	-3.61371900	-1.54555200
H	-2.81988000	-1.87198600	-2.62327500
H	-2.02075400	-0.65487600	-1.65087000
N	0.84070100	0.13718900	2.01607000
H	1.33242000	-0.65867200	1.56332600
H	-0.16372000	0.20643000	1.69586900
H	0.88173100	0.03055000	3.02931700
H	1.31877100	1.00176600	1.74450300

conf_1

C	3.72824300	-1.60562500	0.04492400
C	4.58151800	-0.79616000	-0.94002100
C	3.95743000	0.51985200	-1.43075200
C	3.65239600	1.52834000	-0.30967500
C	2.38433700	1.24590000	0.45297400

O	1.35906100	0.83248400	-0.05696300
O	2.35922600	1.50930500	1.77355200
H	3.21756100	1.82115500	2.09068900
H	3.50544000	2.52504100	-0.74388900
H	4.49946400	1.60944500	0.37927100
H	4.65352000	0.99825800	-2.12323800
H	3.03984900	0.33831500	-1.99438400
H	4.80458000	-1.41339400	-1.81625600
H	5.54808400	-0.57313500	-0.47462100
C	2.42133300	-2.14336000	-0.54910100
C	1.39826900	-2.53685600	0.51645200
C	0.03570300	-2.92101700	-0.07589200
C	-1.06634300	-2.96361800	0.95182200
C	-2.37745300	-2.79369400	0.72109900
C	-3.03303900	-2.58437700	-0.61567300
C	-4.30287800	-1.71977100	-0.56550200
C	-4.08471000	-0.28944700	-0.05963200
C	-3.11429500	0.53944300	-0.90542000
C	-2.92999700	1.97175300	-0.39300300
C	-1.77759500	2.72192000	-1.06832400
C	-1.45722100	4.08593100	-0.44354600
C	-0.87327500	3.99621200	0.97039900
H	-1.59001400	3.58316000	1.68719700
H	0.02552900	3.36923600	0.98431800
H	-0.58986600	4.98232700	1.34441900
H	-0.74376500	4.60695400	-1.08899100
H	-2.36083800	4.70474700	-0.42876500
H	-2.02553500	2.85428800	-2.12623300
H	-0.87147600	2.10148300	-1.05129800
H	-3.86228900	2.53061200	-0.52575200
H	-2.79193500	1.96547100	0.70212500
H	-2.13647800	0.04240200	-0.95690100
H	-3.46739200	0.57221600	-1.94150500
H	-3.73143200	-0.32515900	0.98155900
H	-5.05056000	0.22378900	-0.01845000
H	-4.74038100	-1.68464900	-1.56798200
H	-5.04283000	-2.21263500	0.07354800
H	-3.30980800	-3.57350800	-1.00263900
H	-2.31895000	-2.18287900	-1.33952800
H	-3.05619300	-2.89622000	1.56671500
H	-0.75919900	-3.21701500	1.96610100
H	0.11758400	-3.90084400	-0.56197900
H	-0.22287800	-2.22157100	-0.87914600
H	1.77906000	-3.35876600	1.13234000
H	1.27573200	-1.68859200	1.20300500
H	2.63786500	-3.00046800	-1.19575300
H	1.96562500	-1.38333900	-1.18917400
H	4.32567800	-2.43781100	0.42948900
H	3.50099000	-0.99266600	0.92786100
N	-0.80351200	0.17736700	1.45750200
H	-1.56529400	0.81827600	1.21593700
H	0.04350600	0.41772700	0.87057000
H	-0.56628000	0.29067400	2.44283500
H	-1.11292300	-0.80136200	1.28650400

conf_37

C	4.62149300	-0.90820800	-0.44054300
C	4.21492200	0.51595400	-0.03715100
C	3.49814100	1.29747200	-1.14584100
C	2.81082300	2.56070200	-0.61907800
C	1.62686300	2.23428700	0.25742200
O	0.96825200	1.21606700	0.13775400
O	1.26556900	3.11371100	1.20022600
H	1.86013400	3.87622300	1.22193200
H	2.42070200	3.16713100	-1.44638100
H	3.51429900	3.19621200	-0.07028100
H	4.21265600	1.58320000	-1.92190200
H	2.74011300	0.67762000	-1.62737100
H	5.10899700	1.06953800	0.26836700
H	3.57553200	0.46678700	0.85149100
C	3.45184600	-1.84600800	-0.77523600
C	2.45019200	-2.04143000	0.36690800
C	1.30048700	-2.98316200	-0.01919400
C	0.33310200	-3.24689900	1.09888900
C	-1.00660900	-3.27422000	1.01888100
C	-1.84137300	-2.99657600	-0.20506100
C	-3.25440600	-2.48498600	0.12745600
C	-3.94560800	-1.78953100	-1.05386600
C	-3.34163200	-0.42395800	-1.41761000
C	-3.63673200	0.68582000	-0.40185900
C	-2.81280900	1.95713100	-0.62295200
C	-3.03933800	3.03247500	0.44251700
C	-2.20482200	4.29248500	0.20879800
H	-2.43682000	4.74079100	-0.76120300
H	-2.39668200	5.04540200	0.97658500
H	-1.13511900	4.06310000	0.22643000
H	-4.10246000	3.29350900	0.47065300
H	-2.80831500	2.62107900	1.43544900
H	-3.05256300	2.36723100	-1.61017200
H	-1.74331100	1.70635300	-0.66709500
H	-4.70373900	0.93090000	-0.43016100
H	-3.48622800	0.31825600	0.62791500
H	-2.25610200	-0.51810500	-1.55022700
H	-3.72290900	-0.10856900	-2.39315000
H	-5.00852000	-1.66053400	-0.82604400
H	-3.89972600	-2.44919200	-1.92645200
H	-3.86471400	-3.32616600	0.46793000
H	-3.21591700	-1.80237000	0.98660500
H	-1.93317100	-3.91506600	-0.79707900
H	-1.32219100	-2.28859500	-0.85835700
H	-1.55937100	-3.56666000	1.91068700
H	0.78579200	-3.50313600	2.05569200
H	1.72486700	-3.94853600	-0.32557200
H	0.78377700	-2.59422000	-0.90145700
H	2.96921400	-2.43852500	1.24751200
H	2.03411800	-1.07230100	0.65929300
H	3.86074800	-2.82153900	-1.05859100
H	2.91778900	-1.48126400	-1.65976700

H	5.29365400	-0.85981300	-1.30397600
H	5.20697400	-1.34566800	0.37505500
N	-0.87061500	-0.14095400	1.54273000
H	-0.89742400	0.10957100	2.53082600
H	-1.79603800	0.02099100	1.13345800
H	-0.15976800	0.45839600	1.03935400
H	-0.63177500	-1.15166600	1.45217700

conf_21

C	4.18660100	-0.78264000	0.08350700
C	4.84688100	0.12337100	-0.96359000
C	3.95461200	1.23919000	-1.52972400
C	3.43595600	2.22800900	-0.47122800
C	2.24969300	1.73323700	0.31523200
O	1.33553300	1.08668200	-0.16247300
O	2.16448400	2.06208600	1.61880900
H	2.94008300	2.55925400	1.91165300
H	3.08613500	3.14260800	-0.96572100
H	4.24221500	2.52807700	0.20601900
H	4.53273000	1.81076500	-2.25912100
H	3.09922900	0.82840600	-2.07026300
H	5.19816200	-0.48843600	-1.80075000
H	5.74249800	0.57902700	-0.52671500
C	3.02643300	-1.62712400	-0.45618400
C	2.10662000	-2.14987000	0.64712800
C	0.86331600	-2.86654900	0.10223400
C	-0.21556200	-3.04289100	1.13872700
C	-1.53428200	-3.13865200	0.90896100
C	-2.21372800	-3.17094500	-0.43256900
C	-3.64763000	-2.62075400	-0.42355900
C	-3.76506800	-1.10589000	-0.21559300
C	-3.16554300	-0.27299700	-1.35820400
C	-3.54870200	1.21284100	-1.32847000
C	-2.91802000	2.01350400	-0.18685500
C	-3.35458200	3.48001100	-0.13153800
C	-2.68189500	4.25801100	0.99992300
H	-3.00547400	5.30075600	1.01448200
H	-2.92432300	3.82636500	1.97665000
H	-1.59214800	4.25290200	0.89011600
H	-3.12809700	3.95275800	-1.09310700
H	-4.44245500	3.52403500	-0.01654600
H	-1.82384600	1.98830100	-0.30026900
H	-3.17605300	1.54995300	0.77936400
H	-3.25786100	1.67692200	-2.27627300
H	-4.63937900	1.29908100	-1.26797400
H	-2.07106700	-0.36794000	-1.37870100
H	-3.51112800	-0.70129200	-2.30487700
H	-3.32056100	-0.83650500	0.75233800
H	-4.82614200	-0.85176100	-0.12279700
H	-4.12723800	-2.87962800	-1.37252300
H	-4.21747700	-3.13726800	0.35616000
H	-2.25359500	-4.22205600	-0.74652600
H	-1.60856400	-2.66769800	-1.19198000

H	-2.18508900	-3.30204200	1.76709200
H	0.12847500	-3.15096100	2.16694400
H	1.15487200	-3.84862200	-0.28978000
H	0.47186900	-2.31578100	-0.76054500
H	2.65337400	-2.81829800	1.32101600
H	1.79757400	-1.29758300	1.26664600
H	3.42283000	-2.46316000	-1.04233700
H	2.42187600	-1.02963300	-1.14376400
H	4.94838700	-1.43946900	0.51429200
H	3.83171800	-0.17485600	0.92710700
N	-0.66617900	0.08219700	1.38657800
H	-1.53090900	0.55070200	1.09918500
H	-0.77062100	-0.94889000	1.27714800
H	0.12808300	0.45031300	0.79316700
H	-0.47555800	0.29695400	2.36526900

conf_5

C	3.44556000	0.31864700	-1.52871300
C	4.15473500	-0.16025000	-0.25783200
C	4.38267600	-1.67569000	-0.19333000
C	3.08381800	-2.51568400	-0.24044400
C	2.07843000	-2.06601600	0.78327900
O	0.98854400	-1.59704800	0.50331500
O	2.41194700	-2.15228000	2.07953100
H	3.29901200	-2.52047500	2.19544000
H	3.31768500	-3.57113400	-0.06457100
H	2.60298800	-2.44178700	-1.21393700
H	5.00587900	-1.99965600	-1.03100400
H	4.95120500	-1.91914400	0.71175800
H	3.59025200	0.15607100	0.62771900
H	5.12694400	0.33665900	-0.17896800
C	3.27393300	1.84362000	-1.60413100
C	2.49176500	2.47316700	-0.44208000
C	1.08014400	1.88890700	-0.25955100
C	0.36852200	2.49323400	0.91978300
C	-0.80885700	3.13769100	0.92756900
C	-1.70055900	3.42441400	-0.24626500
C	-3.19208400	3.14648600	0.01495400
C	-3.51725300	1.70482700	0.42351300
C	-2.99796600	0.63652900	-0.54309400
C	-3.45950200	-0.78227800	-0.19221400
C	-2.72240400	-1.87697100	-0.96897800
C	-3.10185200	-3.30674400	-0.56295500
C	-4.56319100	-3.66941400	-0.83887200
H	-4.81002400	-3.51862300	-1.89405800
H	-4.75789700	-4.71718000	-0.59992500
H	-5.25519700	-3.06644600	-0.24552400
H	-2.45106500	-4.00390900	-1.09998300
H	-2.88385500	-3.45083800	0.50381400
H	-1.63984200	-1.74386600	-0.84314900
H	-2.91639600	-1.73908200	-2.03910200
H	-3.35635100	-0.97043000	0.89379300
H	-4.53765500	-0.85623200	-0.35849700

H	-3.32958200	0.87169900	-1.56015000
H	-1.90159500	0.66748100	-0.58850900
H	-4.60280500	1.60360500	0.52147300
H	-3.11852600	1.51783700	1.43137300
H	-3.55513000	3.82554200	0.79329100
H	-3.74906500	3.39710300	-0.89306400
H	-1.36599600	2.88093000	-1.13115800
H	-1.59542700	4.48915500	-0.48862900
H	-1.14541800	3.55616300	1.87578200
H	0.92321700	2.45487200	1.85890200
H	1.16764800	0.80602500	-0.11622700
H	0.50624100	2.02765500	-1.17851500
H	3.05065700	2.35867500	0.49326100
H	2.41040500	3.55084100	-0.61087200
H	2.77440300	2.09072500	-2.54694300
H	4.26160800	2.31426300	-1.65109900
H	4.01948000	-0.01116200	-2.40155100
H	2.46558500	-0.16126000	-1.61541500
N	-0.86295600	-0.21040700	1.87983500
H	-0.71614700	0.78655500	1.61691700
H	-0.11049400	-0.80558500	1.43367500
H	-0.83321200	-0.30499400	2.89444100
H	-1.78025800	-0.50944400	1.53376600

conf_9

C	2.69451800	0.20398500	1.82835800
C	4.21304400	0.24330900	1.61457000
C	4.71028800	-0.04161300	0.18945300
C	4.13436300	0.90457700	-0.89571700
C	2.78635800	0.49528700	-1.42040500
O	1.76610600	1.14915100	-1.29929000
O	2.70123700	-0.67818600	-2.07403900
H	3.55967100	-1.12106000	-2.12505500
H	4.81583800	0.93126500	-1.75370500
H	4.04597200	1.92212600	-0.51577200
H	5.79638000	0.07074000	0.17581400
H	4.51911800	-1.08709500	-0.07750200
H	4.69296200	-0.47814700	2.28399400
H	4.58294200	1.22882900	1.91623800
C	2.03085100	-1.13137800	1.48022700
C	0.51958700	-1.11711800	1.72625200
C	-0.20735200	-2.39788900	1.27322400
C	-0.27268000	-2.55138700	-0.22025800
C	-1.36396400	-2.79804700	-0.96102900
C	-2.78002600	-2.98774500	-0.48614400
C	-3.65084300	-1.72597700	-0.65811700
C	-3.34628400	-0.63565400	0.37184100
C	-4.09434700	0.67589400	0.11712300
C	-3.75948500	1.78519800	1.12477400
C	-2.33545000	2.35094500	1.02211500
C	-2.07580400	3.18171700	-0.23977500
C	-0.65143400	3.73546200	-0.31818800
H	-0.49674400	4.32010600	-1.22821700

H	-0.45021700	4.39110500	0.53283200
H	0.10244800	2.94230600	-0.29700200
H	-2.30617800	2.59931500	-1.14681500
H	-2.79331900	4.00805600	-0.27195600
H	-1.59703700	1.54073200	1.09753200
H	-2.14419200	2.98848800	1.89107700
H	-4.47113700	2.60812300	1.00175300
H	-3.91765000	1.39658800	2.13630300
H	-3.90039500	1.02294600	-0.90710900
H	-5.17018600	0.47772200	0.15041200
H	-2.26658700	-0.45390600	0.41453300
H	-3.60728800	-1.00614000	1.36968000
H	-3.52478000	-1.33106400	-1.67617200
H	-4.70533800	-2.00596700	-0.58087600
H	-2.80314100	-3.29968400	0.56058000
H	-3.22155700	-3.80549900	-1.06268200
H	-1.22196500	-2.91147400	-2.03638600
H	0.67959500	-2.48671100	-0.74450200
H	-1.21081400	-2.41580600	1.69964000
H	0.32175200	-3.26403100	1.69089900
H	0.33454000	-0.96764500	2.79434500
H	0.07682500	-0.24655300	1.22470200
H	2.48387300	-1.93319500	2.07508300
H	2.22723100	-1.39018800	0.43529900
H	2.49529500	0.43346000	2.88013400
H	2.21713000	1.00675800	1.25663700
N	-0.74729600	0.31058800	-1.77105000
H	-0.97810400	0.25801400	-2.76287200
H	-0.86693400	-0.62842100	-1.34052200
H	0.25861900	0.62429900	-1.64921500
H	-1.37232500	0.98237600	-1.31695900

conf_69

C	-5.00904200	-0.06881500	0.40823900
C	-5.68399000	-1.00194700	-0.60494200
C	-4.73488200	-1.91361900	-1.39846800
C	-3.90250700	-2.86866800	-0.52493800
C	-2.69573800	-2.23499600	0.11683500
O	-1.97740400	-1.42135700	-0.43570900
O	-2.35524700	-2.62050900	1.36138400
H	-2.98801800	-3.25569300	1.72297600
H	-3.49930900	-3.67576800	-1.14891300
H	-4.53076900	-3.34489500	0.23477800
H	-5.32915800	-2.52570700	-2.08040800
H	-4.05419300	-1.33147100	-2.02319200
H	-6.25784900	-0.40419500	-1.32039000
H	-6.41465100	-1.62810800	-0.08093300
C	-4.09549400	0.98896200	-0.22127400
C	-3.11165500	1.59717400	0.77832700
C	-2.08640300	2.52916400	0.11666700
C	-0.93395300	2.87331400	1.02164400
C	0.33632800	3.09742400	0.65061800
C	0.88216900	3.09854200	-0.75358400

C	2.38049400	2.77654900	-0.84183600
C	2.73901300	1.35946000	-0.39045500
C	4.21909700	1.00416600	-0.53600700
C	4.54236800	-0.41573100	-0.06582200
C	6.02059300	-0.78566700	-0.20497000
C	6.33994500	-2.20596300	0.26796900
C	7.82010100	-2.56396300	0.12456400
H	8.44776300	-1.88523500	0.70942200
H	8.01959100	-3.58136800	0.46919300
H	8.14219400	-2.49660200	-0.91870100
H	6.03693000	-2.31323000	1.31667500
H	5.73307000	-2.92042900	-0.30148400
H	6.62647400	-0.06944300	0.36314400
H	6.32249200	-0.67608100	-1.25358400
H	4.24381800	-0.52637000	0.98607000
H	3.93854500	-1.13392000	-0.63797400
H	4.51568900	1.11994100	-1.58438400
H	4.81792700	1.72361400	0.03377100
H	2.14827400	0.64203200	-0.98440200
H	2.45711900	1.23618700	0.66535800
H	2.71159200	2.91691700	-1.87486200
H	2.94232700	3.49831600	-0.23865300
H	0.71308400	4.09653100	-1.17684000
H	0.31405400	2.41463100	-1.39505100
H	1.04350400	3.38277000	1.42804700
H	-1.18214500	2.99804200	2.07468200
H	-2.58832700	3.45455800	-0.19189900
H	-1.72230600	2.07493100	-0.81146700
H	-3.64694500	2.13687400	1.56694400
H	-2.58862700	0.77683700	1.28798300
H	-4.70541700	1.77785000	-0.67432000
H	-3.51790100	0.54250500	-1.03491500
H	-5.78211800	0.42366100	1.00588000
H	-4.43253300	-0.66270700	1.13064900
N	0.05130100	-0.18695800	0.87959400
H	-0.11837300	0.83787900	0.96838300
H	0.13212200	-0.59642300	1.81052200
H	-0.73929100	-0.66582900	0.36044800
H	0.93478400	-0.30926800	0.37948400

conf_59

C	2.62425700	2.35094700	-0.44426300
C	1.89052700	2.94367800	-1.65568200
C	0.39160600	2.63640700	-1.77639700
C	-0.45873800	3.24455500	-0.64974000
C	-0.41867300	2.45227100	0.62979300
O	-0.36124200	1.23522800	0.64570800
O	-0.49493800	3.09996800	1.79687200
H	-0.52505100	4.05800400	1.66948200
H	-1.51534700	3.25641000	-0.94544300
H	-0.17412100	4.28555000	-0.46560200
H	0.02823500	3.04472500	-2.72217900
H	0.20990700	1.55965600	-1.81160400

H	2.38412700	2.59141700	-2.56727100
H	2.02026400	4.03193600	-1.64822800
C	2.76589000	0.82476600	-0.45713500
C	3.70021000	0.31825000	0.64690500
C	4.01172800	-1.19172000	0.56795800
C	2.97563900	-2.10002300	1.17345600
C	2.04002800	-2.82216900	0.53778500
C	1.74237200	-2.82966800	-0.93427400
C	0.32051400	-2.33304500	-1.26716900
C	-0.80433300	-3.25601300	-0.78169800
C	-2.21170000	-2.69459900	-1.02304500
C	-2.57373400	-1.52746800	-0.10039900
C	-3.94728900	-0.91110100	-0.37055600
C	-4.27302500	0.26101200	0.55890400
C	-5.63577700	0.89401000	0.27392600
H	-5.68556300	1.27774900	-0.74928600
H	-6.44007100	0.16256400	0.39082600
H	-5.84196400	1.72459800	0.95328400
H	-4.24167800	-0.08424700	1.60014200
H	-3.48576900	1.02041300	0.46515500
H	-4.72098300	-1.68128000	-0.27465400
H	-3.98400900	-0.56999900	-1.41200300
H	-2.56262600	-1.89052900	0.94285300
H	-1.82149200	-0.73542000	-0.18272300
H	-2.29739400	-2.36928100	-2.06623400
H	-2.94874700	-3.49270000	-0.89120900
H	-0.69089100	-3.47915600	0.28852500
H	-0.70316400	-4.22070400	-1.28885900
H	0.19493000	-1.32156800	-0.86425600
H	0.23549700	-2.22965600	-2.35359500
H	1.85479300	-3.85290000	-1.31205200
H	2.46786300	-2.21518700	-1.46902000
H	1.44493900	-3.50746100	1.14083200
H	3.05795400	-2.22919300	2.25323400
H	4.94907600	-1.37937500	1.10000600
H	4.19467400	-1.46027500	-0.47530700
H	4.64631900	0.86329100	0.57371600
H	3.28975700	0.56572700	1.63476600
H	3.17523300	0.52022600	-1.42787200
H	1.78310700	0.35122000	-0.37796700
H	3.62775200	2.78865700	-0.41914800
H	2.15243100	2.67166000	0.49377300
N	-0.00486900	-0.93708200	2.10744000
H	-0.14026600	0.02564600	1.69064000
H	0.84581100	-1.37058100	1.69201000
H	0.08327600	-0.89241900	3.12190200
H	-0.82029400	-1.50004600	1.85043000

conf_85

C	-4.64899000	0.02759700	0.91931300
C	-5.66819200	-0.66602000	0.00558200
C	-5.08786000	-1.54588700	-1.11142300
C	-4.27877900	-2.75202500	-0.60364100

C	-2.88481900	-2.40451600	-0.15273900
O	-2.17117700	-1.60751400	-0.73675300
O	-2.38060800	-3.02492600	0.92520700
H	-3.02354200	-3.62903500	1.32070900
H	-4.14511900	-3.47320000	-1.41901600
H	-4.81998700	-3.27382400	0.19213300
H	-5.91300100	-1.93410300	-1.71258400
H	-4.45958200	-0.96409600	-1.78941000
H	-6.30365100	0.09545100	-0.45797700
H	-6.33592400	-1.27925200	0.62113800
C	-3.78837000	1.08744100	0.22495700
C	-2.72300900	1.67464600	1.15491300
C	-1.90000400	2.82740800	0.55012900
C	-1.12166800	2.47681200	-0.68842100
C	0.17144800	2.74620500	-0.93000600
C	1.14878500	3.43858200	-0.01618600
C	2.60800100	3.01460000	-0.23993700
C	2.84737900	1.51773400	-0.03510900
C	4.31448400	1.09248000	-0.09397300
C	4.50035100	-0.41904200	0.05681300
C	5.96237600	-0.86826700	0.01405700
C	6.13889200	-2.38163900	0.16000000
C	7.60409300	-2.81868500	0.11751800
H	8.17731100	-2.35270800	0.92430000
H	7.70055600	-3.90177900	0.22361400
H	8.07264600	-2.53372100	-0.82899900
H	5.68645400	-2.70779800	1.10450600
H	5.58235400	-2.88806000	-0.63821700
H	6.51834800	-0.35961600	0.81077400
H	6.41316800	-0.53957700	-0.93009100
H	4.05314600	-0.74674400	1.00576900
H	3.94733100	-0.93310500	-0.74300800
H	4.74846300	1.42115100	-1.04510200
H	4.87263700	1.61074800	0.69362200
H	2.30014000	0.96521700	-0.81185700
H	2.42803800	1.21676300	0.93698000
H	2.91903200	3.29371500	-1.25279900
H	3.24688800	3.58083300	0.44407900
H	0.87640200	3.28026400	1.03238300
H	1.06681100	4.51957700	-0.18387900
H	0.55494400	2.50127700	-1.92032900
H	-1.69592700	2.03099900	-1.49807500
H	-1.23852000	3.24136800	1.31407100
H	-2.59357300	3.63597200	0.28488300
H	-3.20568700	2.04462100	2.06517200
H	-2.05149300	0.87081400	1.48960500
H	-4.43700800	1.89473900	-0.13471400
H	-3.31386000	0.65488900	-0.65897600
H	-5.19154200	0.49840800	1.74556200
H	-3.99790700	-0.71646600	1.39778200
N	0.07570400	-0.39808300	0.08417200
H	0.19160900	-0.43726900	1.09644300
H	0.92514300	-0.75420200	-0.35490200
H	-0.77187900	-0.96629300	-0.20678700
H	-0.06356800	0.59559800	-0.20474700

conf_83

C	-3.14715400	0.47414700	-0.92609900
C	-2.87329600	-0.47634500	-2.09902800
C	-1.81828800	-1.56598600	-1.86377300
C	-2.20223600	-2.57238300	-0.76846500
C	-1.93882300	-2.08412000	0.63311200
O	-1.00960900	-1.34634300	0.90612900
O	-2.71833100	-2.52255900	1.62507400
H	-3.42801400	-3.08952000	1.29367300
H	-1.59487500	-3.48100000	-0.86863600
H	-3.24663900	-2.88372600	-0.87430300
H	-1.68011000	-2.12334100	-2.79315100
H	-0.84804200	-1.12966500	-1.61667000
H	-2.56636200	0.11569900	-2.96752700
H	-3.81253800	-0.96247900	-2.38612000
C	-2.00868200	1.44711800	-0.60232500
C	-2.38153100	2.41222700	0.52757000
C	-1.37433600	3.55932400	0.73812100
C	-0.10957900	3.18102300	1.45883700
C	1.12201300	3.07362300	0.93938800
C	1.53139500	3.28012100	-0.49257100
C	2.60165800	2.28822300	-0.97183000
C	2.10235600	0.85057500	-1.13191500
C	3.22796400	-0.12382900	-1.48690000
C	2.78211800	-1.57633200	-1.69849800
C	2.07107700	-2.22605700	-0.50329900
C	2.86475100	-2.19983100	0.80696500
C	2.20165800	-3.01083400	1.92439100
H	2.16901400	-4.07057800	1.65998200
H	2.74646000	-2.92865400	2.86868000
H	1.16402800	-2.70205200	2.09419800
H	3.87582400	-2.58322900	0.63440000
H	3.02304700	-1.15680400	1.12659300
H	1.85641700	-3.27126800	-0.75397100
H	1.09659400	-1.75445200	-0.34287400
H	2.11740800	-1.62459500	-2.56852400
H	3.66215800	-2.17492900	-1.95645000
H	3.71964400	0.22582400	-2.40062800
H	3.99619100	-0.08131400	-0.70607900
H	1.32314700	0.81721400	-1.90322300
H	1.60897400	0.52702900	-0.20756300
H	2.99989700	2.63342300	-1.93042100
H	3.44642200	2.30578500	-0.27178100
H	1.93738300	4.29592100	-0.57793300
H	0.66300800	3.23877900	-1.15388600
H	1.95173500	2.89546600	1.62667200
H	-0.21624300	3.07308400	2.53968000
H	-1.85944100	4.34078900	1.33221200
H	-1.14302900	4.01197100	-0.22870700
H	-3.35506700	2.85707700	0.29861300
H	-2.51957300	1.85713100	1.46470600
H	-1.77941100	2.02730000	-1.50425000

H	-1.09631900	0.89807200	-0.35153800
H	-4.03933300	1.06044700	-1.16956400
H	-3.41752800	-0.09033000	-0.02428700
N	0.65228300	0.13705100	2.32029900
H	-0.10217700	-0.48265600	1.92401500
H	0.58253500	1.07507100	1.87532700
H	0.55744600	0.22336800	3.33171100
H	1.55852400	-0.28099000	2.09382100

conf_4

C	4.02084600	-0.89326100	0.58975500
C	4.93045800	-0.04073000	-0.30301900
C	4.22230000	1.02165800	-1.16080900
C	3.27960800	1.96707700	-0.37083700
C	1.88417300	1.43845600	-0.16809000
O	1.37131700	1.22896800	0.91636200
O	1.14589000	1.19554400	-1.26583800
H	1.63867800	1.38907600	-2.07534400
H	3.17877800	2.91396200	-0.91397100
H	3.69129300	2.19785000	0.61131000
H	4.98373200	1.64065200	-1.63960100
H	3.67627400	0.54316800	-1.98158900
H	5.50536400	-0.68595800	-0.97525800
H	5.66402300	0.46549300	0.33280900
C	2.95271400	-1.69776400	-0.15986800
C	1.85595700	-2.22553800	0.76666000
C	0.65867500	-2.81745200	0.01163200
C	-0.52585800	-3.08434900	0.90332700
C	-1.81634300	-3.08953700	0.53521700
C	-2.35495100	-2.87879000	-0.85370200
C	-3.77889600	-2.30209000	-0.89178600
C	-3.92850400	-0.92030900	-0.24566400
C	-3.09849200	0.18207800	-0.90875000
C	-3.26413300	1.55152400	-0.24141100
C	-2.27384300	2.60582400	-0.74664700
C	-2.25701800	3.90266900	0.07303000
C	-1.68620800	3.73213800	1.48495000
H	-1.62544700	4.69026500	2.00514800
H	-2.30987000	3.07965000	2.10486700
H	-0.67384900	3.31350200	1.45170000
H	-1.66005800	4.64782400	-0.46139500
H	-3.27078900	4.31306600	0.13263300
H	-2.51582700	2.83439100	-1.78961500
H	-1.26193200	2.18046600	-0.76379300
H	-4.28935600	1.90806700	-0.38537500
H	-3.18351200	1.44765000	0.85423200
H	-2.03530400	-0.09333300	-0.91454300
H	-3.37402500	0.26205900	-1.96571500
H	-3.66689700	-0.98984800	0.82045700
H	-4.98405900	-0.63172300	-0.26438200
H	-4.10795900	-2.25066900	-1.93427200
H	-4.45675000	-3.00192500	-0.39226200
H	-2.36800700	-3.85501900	-1.35498800

H	-1.67123000	-2.26289500	-1.44476500
H	-2.55463500	-3.35265900	1.29145000
H	-0.29106100	-3.36027700	1.93091400
H	0.96325800	-3.75418700	-0.47175900
H	0.37293800	-2.14716600	-0.80698800
H	2.26693600	-2.97321700	1.45329300
H	1.51879000	-1.39573300	1.39968900
H	3.41986900	-2.52645300	-0.70306800
H	2.47769100	-1.07640200	-0.92622000
H	4.64617200	-1.57627900	1.17245600
H	3.53064500	-0.24578100	1.32452200
N	-0.93653700	0.02108900	1.65356800
H	-1.75480500	0.53497500	1.31308800
H	-1.00970500	-0.97367300	1.35773500
H	-0.05925000	0.47267600	1.27304400
H	-0.91491800	0.06507100	2.67246200

conf_164

C	2.94380400	1.02988300	1.85628600
C	4.11431100	0.37752600	1.11322200
C	4.83079200	1.30340300	0.12214400
C	3.92238100	1.85285300	-1.00304300
C	3.16891700	0.76323300	-1.71560700
O	1.95285000	0.67402900	-1.72076100
O	3.87320100	-0.18039500	-2.35526800
H	4.82531500	-0.02706200	-2.27823700
H	4.52751800	2.39702200	-1.73620500
H	3.18228500	2.54596300	-0.60782400
H	5.25084000	2.16570000	0.64659700
H	5.68692000	0.77522700	-0.31365900
H	3.76773200	-0.51681700	0.58151500
H	4.84883200	0.01952800	1.84168200
C	2.26811600	0.10420100	2.87996500
C	1.72364700	-1.21377600	2.31021100
C	0.70198400	-1.02391000	1.17456100
C	0.28277200	-2.33774100	0.57481100
C	-0.95210600	-2.86024000	0.51718000
C	-2.22597900	-2.26492700	1.04518000
C	-3.41552300	-2.38607900	0.07683800
C	-3.20484800	-1.70271200	-1.28126100
C	-2.82512100	-0.21659700	-1.20897900
C	-3.88092400	0.67990300	-0.55645500
C	-3.48221600	2.15822200	-0.55602300
C	-4.53060300	3.09190200	0.06247400
C	-4.77248200	2.85677600	1.55590100
H	-3.83943000	2.94143500	2.12269700
H	-5.47151800	3.59277000	1.95929300
H	-5.19449200	1.86775500	1.75253300
H	-4.20764300	4.12661100	-0.08955900
H	-5.47431800	2.98495400	-0.48474600
H	-3.28877500	2.47361400	-1.58846400
H	-2.53327700	2.27538700	-0.01519100
H	-4.83393200	0.55662300	-1.08433200

H	-4.05226600	0.34366500	0.46977100
H	-1.88496600	-0.08816500	-0.65565300
H	-2.65074700	0.15913200	-2.23004600
H	-4.11981500	-1.80509900	-1.87295700
H	-2.43773600	-2.26037300	-1.83640700
H	-3.63195600	-3.44524700	-0.09618300
H	-4.30212500	-1.97375000	0.56477800
H	-2.07918900	-1.22371300	1.33821500
H	-2.48538300	-2.80264600	1.96546900
H	-1.05418100	-3.85722600	0.08925600
H	1.10424800	-2.95786400	0.21250100
H	1.15799300	-0.40022300	0.39747200
H	-0.15989100	-0.46616600	1.54830800
H	2.54987300	-1.83465000	1.94724900
H	1.25412000	-1.78467200	3.11642800
H	1.45083400	0.65334300	3.35961000
H	2.98231400	-0.13348000	3.67532200
H	3.30640600	1.92186500	2.37861600
H	2.19624700	1.38571000	1.14025600
N	0.09165400	-1.16830700	-2.31517600
H	-0.76386500	-0.63874900	-2.49914400
H	0.89708300	-0.49648800	-2.17376600
H	-0.05668300	-1.71752400	-1.44188100
H	0.28211700	-1.79883600	-3.09326800

conf_93

C	2.81495500	1.98249600	-0.48686400
C	2.04004900	2.77009400	-1.55285600
C	0.50897600	2.67375100	-1.50541000
C	-0.11299700	3.29747000	-0.24706100
C	-0.05270700	2.40870100	0.96549600
O	-0.13912400	1.19471700	0.90060300
O	0.05247000	2.97187600	2.17437700
H	0.12621300	3.93400100	2.11196800
H	-1.18195100	3.47880500	-0.41230300
H	0.34066400	4.26992400	-0.02939400
H	0.09993400	3.19964900	-2.37104000
H	0.17530300	1.63670600	-1.58608200
H	2.37153500	2.43491400	-2.54103800
H	2.32238000	3.82697600	-1.48642400
C	2.72426000	0.45891400	-0.62403800
C	3.69558700	-0.27300000	0.30772000
C	3.75182600	-1.79980300	0.08370500
C	2.66831100	-2.59648900	0.76095000
C	1.53201000	-3.06511600	0.22229300
C	1.02264000	-2.83248400	-1.17126800
C	-0.34201500	-2.11370900	-1.20572800
C	-1.50791600	-2.94611100	-0.65720000
C	-2.83572600	-2.18058400	-0.59137100
C	-2.90114100	-1.16210800	0.55187300
C	-4.21828000	-0.37711100	0.63345200
C	-4.49071500	0.54621800	-0.56272300
C	-3.42777500	1.63056100	-0.76068300

H	-3.31548300	2.22939900	0.15027200
H	-2.45140500	1.20289800	-1.00411200
H	-3.70466100	2.30660700	-1.57322300
H	-4.58526400	-0.04439800	-1.47916200
H	-5.46451700	1.02161400	-0.41306000
H	-4.21439400	0.22640500	1.54893900
H	-5.04776500	-1.08462700	0.73679900
H	-2.77703700	-1.70851700	1.50193900
H	-2.07174800	-0.45290100	0.46769000
H	-3.00253700	-1.67583700	-1.54841900
H	-3.66023500	-2.89082900	-0.47345300
H	-1.27313000	-3.33503100	0.34329500
H	-1.62678300	-3.83093000	-1.29045900
H	-0.25940600	-1.15987900	-0.67238800
H	-0.56721800	-1.84993100	-2.24401200
H	0.92047800	-3.80091800	-1.67570700
H	1.74675500	-2.25331500	-1.74557900
H	0.91815200	-3.71608300	0.84424600
H	2.88348000	-2.87659700	1.79257400
H	4.70714300	-2.17114500	0.46536200
H	3.75552100	-1.99783100	-0.99113400
H	4.69968800	0.13087500	0.14579600
H	3.45285600	-0.05590800	1.35639200
H	2.96408100	0.18758900	-1.65921900
H	1.69831200	0.12352300	-0.44969300
H	3.86814000	2.27359200	-0.55966600
H	2.50637300	2.28377700	0.52284500
N	0.10378800	-1.06749100	2.24823900
H	0.03877400	-0.07911600	1.87416300
H	0.79995800	-1.59481400	1.68056900
H	0.37290300	-1.07480200	3.23134600
H	-0.81443900	-1.50428000	2.13212000

conf_209

C	-3.93909900	2.07320700	0.07336200
C	-5.44519000	1.95381400	-0.19529500
C	-6.02248100	0.53098700	-0.20110000
C	-5.91215400	-0.19476600	1.15098900
C	-4.54451600	-0.75735300	1.43157400
O	-3.85541700	-1.29633100	0.58211900
O	-4.06649800	-0.70284400	2.68377200
H	-4.67987700	-0.25125000	3.27932100
H	-6.58479900	-1.06106000	1.15828200
H	-6.23253800	0.45890500	1.96857600
H	-7.08265500	0.58650400	-0.45778600
H	-5.55096100	-0.08613500	-0.96907000
H	-5.66894700	2.41452300	-1.16286300
H	-5.98640000	2.54644600	0.55087500
C	-3.04699000	1.47651000	-1.01922800
C	-1.55839000	1.57180900	-0.67305600
C	-0.60462700	1.08931700	-1.78324200
C	-0.74020100	-0.36743000	-2.12550300
C	0.22883200	-1.29757700	-2.11973100

C	1.67721000	-1.11833200	-1.76357300
C	2.07155700	-1.84723300	-0.46455900
C	3.58072500	-1.86646700	-0.19435600
C	4.20001900	-0.48133100	0.01215500
C	5.68411500	-0.53820700	0.38186900
C	6.31188800	0.84288200	0.57973300
C	7.79651700	0.78950800	0.94878600
C	8.41408900	2.17528400	1.14299100
H	7.90816000	2.72395900	1.94335700
H	9.47272700	2.10733000	1.40476500
H	8.33511000	2.77339600	0.23007700
H	7.91864800	0.20089100	1.86564000
H	8.34237700	0.25059900	0.16561100
H	5.76431000	1.38226500	1.36346500
H	6.18931600	1.43211300	-0.33803100
H	5.80706800	-1.12850200	1.29830400
H	6.23064500	-1.07612500	-0.40237700
H	4.08525400	0.12092000	-0.89602700
H	3.65028000	0.04709200	0.80298400
H	4.08436100	-2.37325200	-1.02580800
H	3.77055000	-2.47844800	0.69432100
H	1.57030600	-1.35861700	0.38410600
H	1.70289500	-2.88052900	-0.50630800
H	1.93254300	-0.06165900	-1.68581900
H	2.28219000	-1.52495000	-2.58238700
H	-0.04363900	-2.30311400	-2.44254500
H	-1.72835700	-0.68234300	-2.45621600
H	0.42260300	1.32227400	-1.49819600
H	-0.81291000	1.67129000	-2.68976700
H	-1.31082200	2.61295600	-0.44256000
H	-1.36030800	1.01144900	0.25204500
H	-3.23160300	2.00867900	-1.95978100
H	-3.32473400	0.43424200	-1.19442200
H	-3.69421900	3.13419200	0.18809500
H	-3.68590700	1.61965200	1.04130700
N	-1.27378100	-1.98435000	0.63008800
H	-0.89335600	-1.59069900	-0.26019700
H	-0.75757100	-1.59068100	1.41661800
H	-2.30598300	-1.73920300	0.70655100
H	-1.14979000	-2.99699100	0.62722900

conf_95

C	-3.48571900	0.45441700	0.90815400
C	-4.80038500	0.08023200	0.21864900
C	-5.00860500	-1.42763900	0.02197800
C	-4.14042000	-2.05782000	-1.09115400
C	-2.65931900	-2.06131200	-0.82781800
O	-1.83559800	-1.55426600	-1.57104200
O	-2.21585400	-2.66563100	0.28425700
H	-2.94712000	-3.00793900	0.81726300
H	-4.29497700	-1.53493500	-2.03486300
H	-4.44264100	-3.10117700	-1.24078800
H	-4.85818500	-1.95158200	0.97525700

H	-6.04838200	-1.61826500	-0.25311400
H	-4.85826900	0.57710800	-0.75708700
H	-5.63571400	0.47100300	0.80742100
C	-3.24619100	1.96292900	1.00014500
C	-1.93211600	2.33210900	1.70363600
C	-0.66285600	1.68700500	1.10290200
C	-0.45473900	2.02390300	-0.34902900
C	0.55017600	2.73746000	-0.88117600
C	1.70695900	3.36894100	-0.16187000
C	3.06583000	3.14545600	-0.85121400
C	3.43473900	1.67484300	-1.07820200
C	3.38924300	0.80195800	0.17845800
C	3.85985800	-0.63613500	-0.06888600
C	3.47030000	-1.60898200	1.04815100
C	3.85626400	-3.07053900	0.78770400
C	5.36657600	-3.30857000	0.71284700
H	5.82243900	-2.78752800	-0.13246100
H	5.86158600	-2.96303300	1.62521500
H	5.58768900	-4.37183700	0.59859300
H	3.43394500	-3.69086900	1.58434800
H	3.38672600	-3.41104200	-0.14497800
H	2.38220600	-1.55246900	1.22455100
H	3.92004100	-1.27360100	1.98973900
H	3.46228900	-1.00309100	-1.02790800
H	4.94452500	-0.63876500	-0.20261400
H	4.00609800	1.25302900	0.96320600
H	2.37060500	0.79296000	0.58749400
H	4.43697600	1.62555800	-1.51573100
H	2.76499800	1.24970300	-1.83971400
H	3.07102000	3.66362700	-1.81565100
H	3.83977800	3.62358700	-0.24311500
H	1.75121600	3.03324900	0.87540800
H	1.52333900	4.44955600	-0.12010700
H	0.51384800	2.93009400	-1.95320700
H	-1.24806300	1.70587400	-1.02440100
H	-0.73981400	0.59768000	1.22240800
H	0.19814600	1.99585300	1.69736000
H	-1.81125400	3.41909400	1.68919500
H	-1.99165700	2.03840500	2.75694900
H	-4.07424000	2.43294300	1.53997300
H	-3.26424600	2.39206100	-0.00784000
H	-3.46490800	0.01739700	1.91471400
H	-2.64838900	0.00966800	0.36695600
N	0.69301800	-0.74851300	-1.16918900
H	-0.27301400	-1.17299800	-1.26959300
H	1.23155100	-0.90334500	-2.02104400
H	0.59587300	0.27559800	-1.00962500
H	1.19889800	-1.16235200	-0.38325000

conf_207

C	-1.89486400	-2.48350200	-1.19054300
C	-0.56168300	-2.78754300	-1.88693500
C	0.71025600	-2.22252300	-1.24073700

C	0.98641700	-2.73396800	0.20348100
C	0.35563800	-1.84932600	1.23503000
O	-0.68337300	-2.09078200	1.82730600
O	0.94813800	-0.66838000	1.48971800
H	1.78855400	-0.55888800	1.01277000
H	2.06461900	-2.75924400	0.38513300
H	0.59773900	-3.74344300	0.33583400
H	1.56485600	-2.51067400	-1.85665400
H	0.69037400	-1.12840000	-1.25098200
H	-0.60263800	-2.41102900	-2.91478100
H	-0.45074400	-3.87401200	-1.96817600
C	-2.25080000	-0.99622500	-1.08893400
C	-3.68860900	-0.77469700	-0.60811400
C	-4.13234600	0.70363900	-0.59021700
C	-3.65611100	1.49739600	0.59654000
C	-2.64485000	2.37824100	0.64411800
C	-1.70457600	2.73982900	-0.46755900
C	-0.26274500	2.27754300	-0.19826500
C	0.70115600	2.65336300	-1.32944300
C	2.03470900	1.89970100	-1.28562000
C	2.89638100	2.18160100	-0.04948400
C	4.20209000	1.37535200	-0.01975200
C	4.01094300	-0.11153600	0.30170500
C	5.30068100	-0.92986200	0.21177100
H	6.05937400	-0.53415900	0.89156500
H	5.13537400	-1.97862400	0.47157700
H	5.71081100	-0.89587200	-0.80063700
H	3.61630700	-0.19470100	1.32630400
H	3.27306400	-0.54332500	-0.38970700
H	4.88201100	1.80374100	0.72332700
H	4.70806300	1.46997900	-0.98733100
H	3.13091000	3.25089900	-0.02520700
H	2.32875900	1.98038700	0.86841800
H	1.82706800	0.82549700	-1.36634500
H	2.61624200	2.15506200	-2.17768700
H	0.88273600	3.73379700	-1.31142200
H	0.21540000	2.44216200	-2.28843200
H	0.08681200	2.69933700	0.75124600
H	-0.26024400	1.18892900	-0.07362200
H	-1.71100800	3.82836400	-0.59582600
H	-2.04346600	2.31067600	-1.41249900
H	-2.48918500	2.92310500	1.57668400
H	-4.25991700	1.37595600	1.49705600
H	-5.22585500	0.73641600	-0.58370500
H	-3.81752200	1.17874700	-1.52238600
H	-4.36177500	-1.32336900	-1.27363900
H	-3.82479000	-1.22083900	0.38556800
H	-2.13107100	-0.52610700	-2.07282300
H	-1.55098000	-0.47727300	-0.42436600
H	-2.68239200	-2.98752900	-1.76018800
H	-1.91464600	-2.93324300	-0.19353600
N	-1.67468400	0.17088600	2.82812400
H	-2.12682100	0.67037000	2.03488000
H	-2.34035900	0.06954700	3.59410500
H	-1.32196500	-0.78373600	2.49917000

H	-0.86915600	0.71368400	3.14100500

conf_118

C	3.15595800	0.50389400	1.75107900
C	4.65800700	0.43443900	1.44777200
C	5.04638800	0.04674000	0.01296200
C	4.44838500	0.96276700	-1.08625900
C	3.04867700	0.59994400	-1.49710300
O	2.07200300	1.31215100	-1.35185100
O	2.86002900	-0.60460300	-2.07088000
H	3.68851300	-1.09804600	-2.14638800
H	5.07070100	0.90414700	-1.98653100
H	4.43854700	2.00302700	-0.76169700
H	6.13351600	0.09911300	-0.07535800
H	4.78547500	-0.99974400	-0.18124100
H	5.13483200	-0.28053500	2.12633500
H	5.10433300	1.40883700	1.67214100
C	2.39248200	-0.80466000	1.52767600
C	0.89332800	-0.66599600	1.80765900
C	0.07786800	-1.93697200	1.51594500
C	-0.05474900	-2.25253600	0.04902400
C	-1.12211200	-2.79083900	-0.56046000
C	-2.38801500	-3.29061700	0.07823200
C	-3.70835600	-2.81339500	-0.56537800
C	-4.25592500	-1.49518200	-0.00370700
C	-3.34236700	-0.29086100	-0.22666500
C	-3.90706500	1.03930100	0.27336700
C	-2.95613000	2.21569500	0.03681600
C	-3.46912700	3.56083100	0.55813700
C	-2.49599600	4.71170500	0.30102100
H	-2.31261900	4.84429600	-0.77001500
H	-2.88737300	5.65550300	0.68607700
H	-1.53182800	4.53165700	0.78710400
H	-4.43411900	3.77824700	0.08834100
H	-3.66339400	3.47188500	1.63224900
H	-2.77002700	2.31896200	-1.04537300
H	-1.98958400	2.00428600	0.52238500
H	-4.86438400	1.24209700	-0.21866500
H	-4.12220400	0.96114500	1.34477300
H	-2.38466600	-0.47560900	0.27284300
H	-3.13716800	-0.21374300	-1.30514500
H	-4.44025700	-1.61207100	1.07066200
H	-5.23101000	-1.29375600	-0.45977300
H	-3.57922500	-2.72986700	-1.65177600
H	-4.46432500	-3.58764500	-0.41259500
H	-2.40084500	-3.07402700	1.14929200
H	-2.34790000	-4.38419400	-0.00133200
H	-1.04838500	-2.98539600	-1.63094400
H	0.83300000	-2.07772300	-0.55825700
H	-0.91223800	-1.84454500	1.96791500
H	0.55938100	-2.78400700	2.02182500
H	0.75359200	-0.39150500	2.85771900
H	0.49024800	0.17556000	1.22731200

H	2.80685400	-1.58662300	2.17479200
H	2.54041200	-1.16066700	0.50340300
H	3.03489000	0.80849700	2.79557400
H	2.69444100	1.29991900	1.15761900
N	-0.46712100	0.45650600	-1.63687400
H	-1.08932800	1.13812200	-1.19264500
H	-0.73653500	0.35021300	-2.61465700
H	0.54132300	0.77872100	-1.57265100
H	-0.57656900	-0.45599200	-1.15404300

conf_196

C	4.55139100	0.14830900	-1.21828500
C	5.68265200	-0.79723000	-0.79437400
C	5.42096700	-1.67036500	0.44011400
C	4.25052300	-2.66629200	0.27603300
C	2.87420800	-2.09198100	0.46131000
O	1.95579600	-2.22382300	-0.32900200
O	2.62787900	-1.41551200	1.59795100
H	3.41866400	-1.36026300	2.15282100
H	4.34506600	-3.46539200	1.02187800
H	4.27647200	-3.13851500	-0.70597700
H	6.31786500	-2.25886400	0.64491300
H	5.28007200	-1.04283000	1.32916100
H	6.58778100	-0.21028800	-0.60757200
H	5.91988700	-1.46043600	-1.63299600
C	4.25084800	1.27903000	-0.22668400
C	3.19917700	2.27208400	-0.74279500
C	1.75641600	1.72090100	-0.79748300
C	1.13112700	1.62246500	0.56823100
C	0.01349800	2.23147200	0.99584400
C	-0.90236100	3.13468200	0.21824000
C	-2.39455700	2.85458700	0.46431900
C	-2.81014200	1.42750900	0.10331900
C	-4.31590100	1.16939800	0.15455200
C	-4.67939300	-0.28583800	-0.14923500
C	-6.18317000	-0.56614200	-0.11785600
C	-6.53725300	-2.02528700	-0.41519800
C	-8.04290900	-2.29277300	-0.38310400
H	-8.46392700	-2.05659300	0.59854100
H	-8.56685500	-1.68183700	-1.12389500
H	-8.26657600	-3.34035800	-0.59786500
H	-6.13717600	-2.30011300	-1.39878700
H	-6.03465900	-2.67387600	0.31303700
H	-6.68511700	0.08418000	-0.84420400
H	-6.58131600	-0.28893700	0.86570300
H	-4.28503300	-0.55970700	-1.13783800
H	-4.18041500	-0.94277300	0.57852100
H	-4.69687500	1.44188100	1.14545400
H	-4.82001900	1.82938600	-0.56003800
H	-2.31989200	0.73320400	0.79957700
H	-2.44049200	1.19035900	-0.90563100
H	-2.63778900	3.04716900	1.51518000
H	-2.98377500	3.56517700	-0.12267100

H	-0.69242600	3.07548800	-0.85272200
H	-0.68796600	4.16872600	0.51521900
H	-0.25556400	2.10001800	2.04398200
H	1.69049100	1.04182300	1.29883200
H	1.77907200	0.73249900	-1.27658300
H	1.15595400	2.36104700	-1.44500300
H	3.20269100	3.17065400	-0.11924300
H	3.48586200	2.59296800	-1.74931400
H	5.17925600	1.82546500	-0.03083300
H	3.93384000	0.87548300	0.74133900
H	4.83641600	0.60081500	-2.17382300
H	3.64600900	-0.42890000	-1.43326400
N	-0.41900400	-0.99345600	-0.00304000
H	-0.99674800	-1.43031800	0.71484500
H	0.49443400	-1.52378300	-0.11709600
H	-0.94311500	-0.97717000	-0.87801000
H	-0.20119700	-0.01368800	0.28235300

conf_247

C	3.78903500	-0.74424400	-0.11589400
C	3.31750300	-0.87758600	1.33796400
C	2.72157200	-2.25630700	1.67402600
C	1.19086500	-2.36542700	1.47178700
C	0.72446900	-2.05273300	0.07713700
O	0.07707600	-1.05677800	-0.20070900
O	1.03849400	-2.90001900	-0.90901100
H	1.56827700	-3.64203900	-0.58563000
H	0.68004100	-1.66984500	2.13731500
H	0.85929900	-3.37784600	1.72760100
H	3.23895800	-3.03376200	1.09954900
H	2.90062800	-2.49644400	2.72421400
H	2.58554000	-0.09939800	1.58045000
H	4.17429200	-0.69575300	1.99325500
C	4.36448600	0.63400600	-0.47228400
C	3.37118600	1.80246000	-0.39944400
C	2.11626100	1.57856800	-1.26492700
C	1.20730800	2.77957200	-1.31392500
C	0.25844600	3.09226600	-0.41745400
C	-0.12610100	2.30458900	0.80293100
C	-1.64713800	2.12573500	0.92866700
C	-2.05511700	1.14576400	2.02991700
C	-3.56600800	0.86451700	2.08523600
C	-4.18669800	0.42213300	0.75066500
C	-3.52695200	-0.81008400	0.12319400
C	-3.98093300	-1.07888600	-1.31403300
C	-3.30695100	-2.29735200	-1.94830600
H	-3.55781300	-3.20670300	-1.39629500
H	-3.62340000	-2.44365200	-2.98370100
H	-2.21392000	-2.21261900	-1.93472500
H	-5.06774200	-1.20597900	-1.34017100
H	-3.80151800	-0.17982100	-1.93000300
H	-3.73961500	-1.69283900	0.73745500
H	-2.43683100	-0.70419200	0.14045400

H	-5.25165400	0.22179300	0.90375400
H	-4.14869200	1.25496500	0.03804800
H	-3.75036300	0.09654700	2.84398700
H	-4.08825000	1.76316400	2.42976900
H	-1.72953700	1.53665800	2.99959600
H	-1.50830800	0.20818100	1.88250300
H	-2.12208100	3.09902800	1.09473100
H	-2.04399000	1.77282900	-0.02992200
H	0.24433100	2.83088600	1.69130600
H	0.35880100	1.32528800	0.80325200
H	-0.28272400	4.02590200	-0.56012700
H	1.39310600	3.48836100	-2.11758400
H	1.58349000	0.71369900	-0.86584000
H	2.43378000	1.31929500	-2.28180300
H	3.05567800	1.98061800	0.63318500
H	3.87559500	2.71758600	-0.72263900
H	4.77545200	0.58471900	-1.48652000
H	5.21106000	0.85059800	0.18779500
H	4.56323000	-1.49824400	-0.29500000
H	2.97670500	-0.98893000	-0.80823200
N	-0.91375300	0.49298800	-2.15362600
H	-0.64556700	0.27534300	-3.11321200
H	-1.93387700	0.48390900	-2.07542300
H	-0.51398700	-0.22576400	-1.49126700
H	-0.53807100	1.42597200	-1.88852900

9E_NH4

conf_19

C	4.76982500	0.13437700	-1.16408600
C	4.59785600	1.66131200	-1.15982200
C	3.46405900	2.19108300	-0.26926500
C	2.08381000	1.60125900	-0.60507900
C	1.00848000	2.10137700	0.32011800
C	-0.10136800	2.75046200	-0.05392300
C	-1.12661400	3.34428700	0.86973300
C	-2.57953500	3.04647800	0.46173800
C	-2.88789900	1.55176400	0.37647300
C	-4.35172500	1.20759000	0.10456400
C	-4.58701200	-0.29934500	-0.02507700
C	-6.04530400	-0.67860500	-0.29064100
C	-6.26807400	-2.18711000	-0.42350000
C	-7.72933600	-2.55367600	-0.68797500
H	-8.08832100	-2.09624400	-1.61449000
H	-7.85918700	-3.63464500	-0.77855400
H	-8.37506400	-2.20754900	0.12427900
H	-5.92583400	-2.68416200	0.49253800
H	-5.64077800	-2.57397000	-1.23608100
H	-6.67120200	-0.28917800	0.52114300
H	-6.38620800	-0.17908600	-1.20535800
H	-4.25053800	-0.79931200	0.89456300
H	-3.96453100	-0.69299000	-0.84268100
H	-4.67758300	1.70857800	-0.81389700
H	-4.97548600	1.60789600	0.91136600

H	-2.57979300	1.07113500	1.31769400
H	-2.27792900	1.11745000	-0.42776100
H	-2.79023900	3.51345900	-0.50672500
H	-3.25158900	3.51943900	1.18381600
H	-0.94429000	3.00262400	1.89507500
H	-0.98581900	4.43181800	0.88016600
H	-0.26900500	2.91350200	-1.11939400
H	1.20294700	1.98258300	1.38888800
H	2.14897600	0.50888400	-0.54401400
H	1.81980100	1.83558200	-1.64167200
H	3.41665700	3.27980500	-0.36392800
H	3.69055100	1.99214400	0.78422000
H	4.42961200	1.99446400	-2.18937100
H	5.53534900	2.12746200	-0.83890800
C	5.02456400	-0.46228700	0.22391700
C	5.24630000	-1.98019400	0.22936000
C	4.05587400	-2.79781500	-0.32512700
C	2.75295700	-2.43303600	0.33093900
O	1.79747800	-1.97024400	-0.26893700
H	0.43048100	-1.26057100	0.27228400
N	-0.42294000	-0.68013300	0.51570000
H	-0.68185100	-0.82685600	1.49120900
H	-0.21089900	0.32984200	0.37197600
H	-1.21666300	-0.93630600	-0.07279400
O	2.64335100	-2.59606400	1.65755600
H	3.45781200	-2.95092000	2.04073800
H	4.23605800	-3.86732100	-0.17105800
H	3.93026700	-2.63645700	-1.39398800
H	6.12102700	-2.23596500	-0.37429700
H	5.48352700	-2.30924300	1.24803500
H	5.90526500	0.01507300	0.66529800
H	4.19144100	-0.21874300	0.89440300
H	3.88648000	-0.32825600	-1.61560500
H	5.60937600	-0.12010300	-1.82006000

conf_9

C	4.05922500	-0.75355100	0.05377300
C	2.85237000	-1.66632500	0.28992200
C	2.36443900	-2.33353900	-0.99913700
C	1.21950000	-3.34449200	-0.80409000
C	-0.05079500	-2.75636700	-0.25683600
C	-1.24737300	-2.84071700	-0.85332600
C	-2.56314000	-2.34052300	-0.31709500
C	-2.52043200	-1.63797000	1.04086500
C	-3.90741700	-1.24411800	1.57530600
C	-4.76877100	-0.29646600	0.72269600
C	-4.36950000	1.18673600	0.71141400
C	-3.12885300	1.55306400	-0.11179300
C	-2.92259000	3.06188000	-0.27018900
C	-1.67202500	3.42019900	-1.07549700
H	-0.76617900	3.03446300	-0.59625200
H	-1.72486300	3.01595300	-2.09395900
H	-1.55406700	4.50140600	-1.17204800

H	-2.85918500	3.51640200	0.72397200
H	-3.80581800	3.49595900	-0.74940000
H	-2.23712800	1.12474600	0.36048000
H	-3.23643900	1.09933000	-1.11277500
H	-4.22532200	1.53268500	1.74160800
H	-5.21275900	1.76267800	0.31533500
H	-5.78810200	-0.35394900	1.11573200
H	-4.83859400	-0.66310000	-0.30839900
H	-3.78348000	-0.80355400	2.57062100
H	-4.47546100	-2.16839600	1.72820000
H	-1.86483500	-0.76186600	0.99545300
H	-2.05888800	-2.31177200	1.76977800
H	-3.02682500	-1.68909000	-1.07076400
H	-3.24396800	-3.19951200	-0.25606400
H	-1.30541600	-3.36257400	-1.80915400
H	0.02070300	-2.27306300	0.71474300
H	1.56433100	-4.12197900	-0.11089200
H	1.00891300	-3.84750000	-1.75245300
H	3.20258500	-2.85444100	-1.47328400
H	2.05504700	-1.55965200	-1.71619800
H	2.03928600	-1.09396900	0.74340000
H	3.12820700	-2.44148100	1.01460100
C	4.54550200	-0.00061600	1.29917800
C	3.58558600	1.05265900	1.87139000
C	3.29362900	2.21865600	0.91155000
C	2.27971400	1.89243100	-0.15300100
O	1.28562600	1.21862300	0.05316200
H	-1.39296500	0.54468600	-1.48389300
N	-0.47282700	0.13312800	-1.66377600
H	0.24470200	0.63001300	-1.06453000
H	-0.49051500	-0.87527100	-1.39805800
H	-0.23789500	0.23301500	-2.65040500
O	2.44964400	2.39957500	-1.38385700
H	3.26911900	2.90846500	-1.44818200
H	4.21913900	2.58843500	0.45855400
H	2.86098100	3.05675300	1.47147500
H	2.63720100	0.60273500	2.17334100
H	4.02985500	1.47390900	2.77594500
H	4.76340800	-0.72658100	2.08917900
H	5.49960000	0.48678600	1.06873000
H	4.88870300	-1.35750300	-0.32834600
H	3.83014300	-0.04628800	-0.75488100

conf_10

C	3.78255500	-0.31352600	-0.58004100
C	2.85507100	0.82215300	-1.02052800
C	3.00984100	2.08165100	-0.16268600
C	2.12717300	3.26394900	-0.60743500
C	0.65501700	2.98199200	-0.52387500
C	-0.23876500	3.71237300	0.15596900
C	-1.72257200	3.45216200	0.22711800
C	-2.20053400	2.14806500	-0.41530400
C	-3.65737700	1.80728400	-0.09093400

C	-4.11662700	0.46448000	-0.67308100
C	-3.38357300	-0.75188600	-0.09661700
C	-3.91100300	-2.08416900	-0.63777000
C	-3.21618000	-3.31997400	-0.05201200
C	-1.73414800	-3.43617300	-0.42337100
H	-1.15618100	-2.58922500	-0.04490400
H	-1.30204800	-4.35420600	-0.01396600
H	-1.60461600	-3.46546800	-1.50993800
H	-3.74037500	-4.21599300	-0.39771600
H	-3.32105900	-3.30952800	1.03975300
H	-4.98507500	-2.14898800	-0.43317200
H	-3.80734700	-2.09465400	-1.72992500
H	-3.48530000	-0.74718500	0.99864900
H	-2.31252500	-0.67646400	-0.31038500
H	-3.99234500	0.47994700	-1.76255600
H	-5.19029400	0.35054400	-0.49202500
H	-4.30518200	2.60771300	-0.46278800
H	-3.79472400	1.79096800	0.99942500
H	-1.55956900	1.31818400	-0.09583800
H	-2.07394700	2.20543400	-1.50163000
H	-2.03662200	3.48405100	1.28182700
H	-2.24560900	4.30212900	-0.22780200
H	0.11159700	4.59760600	0.68663200
H	0.30277900	2.12541900	-1.09306800
H	2.37631300	3.50658700	-1.64804400
H	2.36837600	4.15094800	-0.01467000
H	4.05547700	2.40495500	-0.18059700
H	2.79298500	1.83778400	0.88737900
H	1.81861000	0.47768200	-0.99068400
H	3.06863300	1.07171600	-2.06659200
C	3.61643300	-1.61261100	-1.38044300
C	2.27230000	-2.33900500	-1.22369400
C	1.99925700	-2.85072600	0.20046200
C	1.51753000	-1.79009700	1.15132300
O	0.73910700	-0.90661500	0.83213300
H	0.15482300	2.03880900	1.46776400
N	-0.01464500	1.27494800	2.15995600
H	-1.01200400	1.23387200	2.37027100
H	0.29734100	0.35450700	1.72319400
H	0.50766400	1.45560700	3.01690500
O	1.92150300	-1.83449200	2.42957100
H	2.54011000	-2.56184600	2.58191200
H	2.88071000	-3.35686900	0.60696500
H	1.19102800	-3.59147500	0.17342600
H	1.43826800	-1.70559100	-1.53337000
H	2.26464100	-3.20491500	-1.88932800
H	3.76098400	-1.39277900	-2.44314600
H	4.42047200	-2.30469100	-1.10582000
H	4.82151800	0.01859800	-0.67486400
H	3.64374600	-0.50460600	0.49291400

conf_23

C	4.79809300	-0.99616600	0.99976000

C	4.08893600	-2.15983400	0.28797300
C	2.64808700	-2.43671300	0.76024200
C	1.55627300	-1.75070400	-0.08474300
C	0.18450800	-1.98180400	0.48706800
C	-0.76705700	-2.74768400	-0.06303600
C	-2.06414800	-3.13390800	0.58991600
C	-3.27617200	-3.15677100	-0.35581300
C	-3.62467200	-1.81262100	-1.00454700
C	-3.94091100	-0.69260200	-0.00984800
C	-4.45052200	0.58901200	-0.68065400
C	-4.60142800	1.78351000	0.26988500
C	-3.27411800	2.39301500	0.73546300
C	-3.45092500	3.56841000	1.69919000
H	-4.03673000	4.36572400	1.23488800
H	-3.97728100	3.25183500	2.60319300
H	-2.48926900	3.99002100	2.00068100
H	-2.71800500	2.74608400	-0.14726400
H	-2.66418700	1.62566400	1.23398500
H	-5.18561500	2.56632400	-0.22393200
H	-5.18261600	1.47664500	1.14684100
H	-5.41578600	0.37330500	-1.14824700
H	-3.79011400	0.87155000	-1.51521800
H	-3.05910200	-0.48119600	0.60670300
H	-4.70176800	-1.04361500	0.69624600
H	-4.48615300	-1.95544600	-1.66477800
H	-2.80279100	-1.50444000	-1.66723700
H	-4.14423800	-3.51651500	0.20526800
H	-3.09655300	-3.89544700	-1.14412300
H	-1.93885600	-4.14649500	0.99337000
H	-2.25175800	-2.49069600	1.45499800
H	-0.56514200	-3.20451100	-1.03339400
H	0.00210400	-1.56469100	1.47958200
H	1.60135400	-2.13496800	-1.10959500
H	1.76789300	-0.67959800	-0.14458600
H	2.54152500	-2.13259900	1.80846000
H	2.45415900	-3.51266900	0.73995400
H	4.68944600	-3.05636000	0.46368400
H	4.09922000	-2.01043400	-0.79767900
C	4.21196900	0.40585800	0.78469700
C	4.35519800	0.92170800	-0.65033500
C	3.78874000	2.33818800	-0.83160100
C	2.28709800	2.36463000	-0.69586200
O	1.56751800	1.52342400	-1.20501100
H	0.00761600	1.02528000	-1.07880400
N	-0.92512500	0.53537900	-1.02935900
H	-1.32250700	0.44526000	-1.96401500
H	-0.79703400	-0.41564800	-0.62395100
H	-1.57789700	1.06873200	-0.44823000
O	1.71470500	3.35106500	0.00398500
H	2.37651400	3.95240100	0.37146400
H	4.00990500	2.70793100	-1.83935800
H	4.25236100	3.03458900	-0.12608100
H	5.41273400	0.94039500	-0.92714200
H	3.85566600	0.25604100	-1.35728300
H	3.15748200	0.41885600	1.08691200

H	4.72472800	1.10130000	1.45929400
H	4.80657200	-1.20601700	2.07460500
H	5.84851600	-0.98329800	0.68799000

conf_153

C	1.67773500	-1.78642800	1.62899400
C	1.03190000	-0.41975800	1.88601500
C	-0.21482000	-0.48379900	2.78130600
C	-1.38853000	-1.31469900	2.21870400
C	-1.90817200	-0.74908000	0.93053200
C	-3.14464500	-0.28629000	0.70679200
C	-3.56715500	0.42276100	-0.55023800
C	-3.65235000	1.95567500	-0.35195200
C	-2.34911900	2.60204000	0.13337100
C	-1.15534700	2.41211700	-0.80632700
C	0.14593400	2.99066100	-0.24648200
C	1.36125600	2.71146400	-1.13384700
C	2.67222000	3.34305400	-0.64932100
C	3.15799900	2.82181000	0.70570600
H	2.45085900	3.04747100	1.50732200
H	3.30044600	1.73644900	0.68424700
H	4.11470900	3.27330800	0.97702900
H	2.53971600	4.42923100	-0.59742000
H	3.44798600	3.16922200	-1.40216800
H	1.50901200	1.61995800	-1.20262900
H	1.15012800	3.05369800	-2.15292900
H	0.31493400	2.58087300	0.75503200
H	0.04102800	4.07418900	-0.11828700
H	-1.37572700	2.86962700	-1.77858700
H	-1.00683700	1.34504800	-1.00225400
H	-2.09227900	2.20255600	1.12040500
H	-2.52563000	3.67271700	0.27761500
H	-4.44995000	2.17502900	0.36490200
H	-3.95975000	2.40337100	-1.30265800
H	-2.85915500	0.20540400	-1.35730400
H	-4.55135100	0.06673500	-0.87507800
H	-3.88041800	-0.33770200	1.50897300
H	-1.17934900	-0.63669400	0.13132500
H	-2.19240900	-1.35278100	2.95876600
H	-1.04985000	-2.34804200	2.07028000
H	-0.57079400	0.53291600	2.97398800
H	0.06789800	-0.90393900	3.75184100
H	0.78566400	0.07655300	0.94171200
H	1.76682400	0.22930700	2.37284400
C	3.04474800	-1.72540200	0.93448100
C	3.05982400	-1.21995700	-0.51415700
C	2.35495200	-2.15061000	-1.52712200
C	0.85450200	-2.08339100	-1.54279300
O	0.11976400	-3.05114600	-1.42306000
H	-1.49204100	-3.00359500	-1.29596200
N	-2.53981000	-2.98675100	-1.09367200
H	-2.84489000	-3.89770400	-0.75034900
H	-2.70928700	-2.25516300	-0.36864500

H	-3.05922400	-2.75321300	-1.93982500
O	0.27912900	-0.88606100	-1.73314500
H	0.92763400	-0.16695500	-1.79126500
H	2.63141600	-3.19085200	-1.35549300
H	2.67961700	-1.89035300	-2.54210900
H	2.65213000	-0.20491500	-0.56997000
H	4.09905400	-1.13022400	-0.83832100
H	3.70882300	-1.08254100	1.52142500
H	3.49714900	-2.72269800	0.95190200
H	1.81584300	-2.28869700	2.59264800
H	1.00000200	-2.43368600	1.06236900

conf_76

C	-4.01392000	0.68171200	1.30893800
C	-3.43309800	2.05965700	1.66149600
C	-2.27011500	2.53137300	0.77559000
C	-1.10409100	1.53082900	0.69573900
C	0.03432400	2.05244300	-0.14006700
C	1.28981400	2.22632700	0.29312500
C	2.40541200	2.90965000	-0.44549900
C	3.72695300	2.12120800	-0.44978700
C	3.73116900	0.95803000	-1.44454300
C	4.91330700	-0.01022700	-1.30260400
C	5.00870800	-0.72994400	0.05200900
C	3.72251200	-1.44774600	0.47055400
C	3.85681500	-2.32479300	1.71628800
C	2.53321700	-2.97886200	2.11642400
H	2.15080000	-3.62242900	1.31579700
H	2.64333900	-3.60308700	3.00539300
H	1.77039500	-2.22494700	2.34320200
H	4.23329700	-1.71542600	2.54466800
H	4.61179200	-3.09624900	1.53466300
H	2.94546000	-0.69488500	0.66530300
H	3.37943900	-2.07548500	-0.36892200
H	5.29002100	-0.02354200	0.83958000
H	5.82260000	-1.45986900	0.00102100
H	5.84618200	0.53402000	-1.48158100
H	4.84590300	-0.76158100	-2.09812200
H	3.72033200	1.36286700	-2.46217700
H	2.79438700	0.39871700	-1.34251200
H	3.92143600	1.75782700	0.56484400
H	4.55203300	2.79724400	-0.69163000
H	2.09548100	3.14579000	-1.46902800
H	2.57636800	3.87104900	0.05411100
H	1.52661800	1.91959600	1.31349100
H	-0.22227000	2.39484700	-1.14424200
H	-1.48862100	0.59389200	0.27812600
H	-0.74535200	1.29494100	1.70360500
H	-1.89517600	3.48366900	1.16156500
H	-2.62917100	2.73855700	-0.23859300
H	-3.10051200	2.03721600	2.70466400
H	-4.22915800	2.80998900	1.61248000
C	-4.50610600	0.56680800	-0.13737500

C	-5.15870900	-0.77963900	-0.47769800
C	-4.24778200	-2.00810700	-0.24193000
C	-2.89940600	-1.85714300	-0.89035000
O	-1.84881800	-1.83623900	-0.27296800
H	-0.34757100	-1.35885100	-0.73193500
N	0.61400400	-0.96477400	-0.93241400
H	0.65399200	0.03848500	-0.65848700
H	1.33418900	-1.46790500	-0.40677800
H	0.82068100	-1.04281000	-1.92808200
O	-2.85028600	-1.70080800	-2.22227100
H	-3.73442600	-1.71579800	-2.61473100
H	-4.72893800	-2.90840500	-0.63914200
H	-4.07284300	-2.16997800	0.81990900
H	-6.05672000	-0.92654800	0.12808000
H	-5.50303500	-0.76320200	-1.51844900
H	-5.23356100	1.36008900	-0.33692200
H	-3.67548900	0.74888100	-0.83023300
H	-3.26282300	-0.08843500	1.51082100
H	-4.84828800	0.47209900	1.98718300

conf_0

C	-3.88078800	-0.77565500	-1.24487900
C	-3.46529300	-2.25150400	-1.34413500
C	-2.31941600	-2.67584500	-0.41336900
C	-1.03394900	-1.85219000	-0.59767200
C	0.06370600	-2.28258600	0.33689200
C	1.27482700	-2.71206900	-0.03934700
C	2.33780200	-3.25778000	0.87040400
C	3.78143400	-2.82542500	0.55464500
C	4.07153200	-1.32480800	0.70987800
C	3.68669700	-0.46728000	-0.50067300
C	3.93314300	1.03024500	-0.29286800
C	3.32766100	1.90888400	-1.39415800
C	3.38762900	3.41502300	-1.10929900
C	2.47351500	3.86198700	0.03628300
H	2.76972200	3.42489000	0.99593200
H	2.50298500	4.94567800	0.16658900
H	1.43057700	3.59013300	-0.16651900
H	3.10553200	3.95597900	-2.01700500
H	4.42099100	3.70418200	-0.89012600
H	3.84391300	1.69129500	-2.33411100
H	2.27897000	1.62186500	-1.56639500
H	5.00924400	1.22108400	-0.22608700
H	3.55497700	1.33349300	0.69674600
H	2.63549100	-0.63317200	-0.76439700
H	4.25162300	-0.80602700	-1.37537900
H	5.13930300	-1.18423800	0.90463500
H	3.56136600	-0.95195200	1.61087700
H	4.04215100	-3.14297100	-0.46144200
H	4.44091800	-3.38562400	1.22250100
H	2.09513500	-3.01861900	1.91245300
H	2.29854200	-4.35213500	0.79686000
H	1.50175000	-2.73646800	-1.10505400

H	-0.19221200	-2.31540500	1.39902500
H	-1.27173400	-0.79464300	-0.43548900
H	-0.68672100	-1.93083200	-1.63322000
H	-2.09212300	-3.73124900	-0.58973100
H	-2.64017100	-2.60977700	0.63241600
H	-3.17951900	-2.46216900	-2.38013500
H	-4.33411500	-2.88414900	-1.13393100
C	-4.32992700	-0.35355400	0.15771600
C	-4.79678400	1.10416200	0.26046700
C	-3.71826700	2.14513400	-0.12277500
C	-2.42525900	1.92503000	0.61227900
O	-1.36793000	1.66413400	0.06517600
H	0.01671600	1.09472800	0.73639400
N	0.90253300	0.62339700	1.07364800
H	0.93935800	0.65145800	2.09217000
H	0.90952200	-0.36769900	0.75842000
H	1.72481500	1.10303500	0.69822900
O	-2.44070800	1.98451700	1.95338300
H	-3.32657400	2.17800500	2.29065600
H	-4.08032100	3.15467500	0.09997100
H	-3.49076500	2.10420300	-1.18621100
H	-5.65447800	1.27271800	-0.39598800
H	-5.15922300	1.29892200	1.27678800
H	-5.15072000	-1.00066200	0.48311900
H	-3.51941000	-0.52296200	0.87706000
H	-3.05322700	-0.14296200	-1.58089100
H	-4.70049200	-0.59623800	-1.94908000

conf_14

C	-4.38392600	-0.34317300	-0.97061400
C	-4.21062200	0.95990700	-0.18061400
C	-3.53422900	2.07793000	-0.98856400
C	-2.03930100	1.84160700	-1.28847300
C	-1.16922000	2.00761600	-0.07295300
C	-0.07743200	2.77869400	0.00468100
C	0.74434600	2.96809900	1.25270700
C	2.03347500	2.12454200	1.29635500
C	3.02199200	2.41061200	0.16074600
C	4.28899100	1.54591800	0.20689000
C	4.06168500	0.07418700	-0.15313100
C	5.32151300	-0.79136400	-0.08240600
C	5.07473800	-2.25231200	-0.46654900
C	6.33707500	-3.11273100	-0.39148500
H	7.11138600	-2.73349400	-1.06419400
H	6.75003500	-3.11656800	0.62116000
H	6.13082400	-4.14829400	-0.67122900
H	4.66583600	-2.29349600	-1.48439000
H	4.30593900	-2.67406200	0.19367700
H	6.08658100	-0.36422500	-0.74068000
H	5.73031600	-0.74457900	0.93334000
H	3.66645500	0.02119800	-1.18041500
H	3.31173900	-0.36407300	0.52347000
H	4.73324700	1.60725400	1.20662700

H	5.03148100	1.95912500	-0.48269000
H	3.30462800	3.46726500	0.20759600
H	2.53565300	2.28300400	-0.81662800
H	1.75348100	1.06366800	1.31514700
H	2.53564700	2.30783400	2.25156200
H	1.01062900	4.02651400	1.34291200
H	0.13413700	2.72094400	2.12584000
H	0.23422600	3.33122900	-0.88123800
H	-1.48245500	1.47448400	0.82291500
H	-1.70685400	2.53268600	-2.06849700
H	-1.92874500	0.82908500	-1.70213700
H	-3.63594800	3.02892400	-0.45771900
H	-4.06533000	2.19566200	-1.93843800
H	-3.65007700	0.78135600	0.74371300
H	-5.19907400	1.30909300	0.13526900
C	-5.15322200	-1.44571300	-0.23120400
C	-4.46859900	-2.05361000	1.00073100
C	-3.14758300	-2.79549100	0.69408100
C	-1.93479100	-1.92401300	0.51650000
O	-1.18185100	-1.97526700	-0.43995400
H	0.07499300	-0.96058400	-0.76756900
N	0.77171800	-0.23015900	-1.08734300
H	1.70613700	-0.40378400	-0.70583300
H	0.82313100	-0.23127400	-2.10578700
H	0.43467200	0.69769100	-0.75581000
O	-1.64668400	-1.05240100	1.49872900
H	-2.30581800	-1.09313400	2.20582400
H	-3.24446100	-3.40508000	-0.20425900
H	-2.91168800	-3.47527600	1.52225600
H	-4.31784600	-1.28719300	1.77135500
H	-5.14835400	-2.78085400	1.45001100
H	-6.12610800	-1.05318100	0.08197300
H	-5.36691000	-2.25813200	-0.93387600
H	-4.93111300	-0.11387000	-1.89105700
H	-3.41347000	-0.72762900	-1.30117500

conf_11

C	-3.82314200	-0.77341100	-0.10245000
C	-3.20049500	0.60970100	0.11543800
C	-2.90566600	1.32389900	-1.20610300
C	-2.43290200	2.78419800	-1.06926100
C	-1.26477900	3.01477000	-0.15073200
C	-0.10112100	3.58058900	-0.49750500
C	1.00936700	3.95469500	0.45113900
C	2.17360200	2.94435300	0.53631400
C	1.86342100	1.74379300	1.43905300
C	2.99503400	0.71090300	1.53655700
C	3.14565900	-0.18399200	0.30347900
C	4.28407300	-1.20197900	0.40963800
C	4.41389200	-2.12076900	-0.81182300
C	3.21383600	-3.04842100	-1.02873700
H	3.03199800	-3.66241600	-0.14088200
H	2.29298200	-2.49628700	-1.24111900

H	3.38705900	-3.72469100	-1.86905900
H	5.31508800	-2.72993700	-0.69679200
H	4.57719600	-1.51112000	-1.70947400
H	5.22638700	-0.66576500	0.56190300
H	4.12960900	-1.81217300	1.30774400
H	3.33402800	0.43568800	-0.58958100
H	2.20281900	-0.72148700	0.15345400
H	2.81354200	0.06655300	2.40270000
H	3.94154800	1.22645300	1.73419000
H	0.93778600	1.24346300	1.12471600
H	1.65186400	2.13251500	2.44038600
H	3.05233800	3.45670500	0.93894400
H	2.46447900	2.62817800	-0.47440500
H	1.40449700	4.92511500	0.13769100
H	0.59792700	4.09629800	1.45510400
H	0.04184200	3.87087400	-1.54072400
H	-1.41871500	2.76499000	0.89766700
H	-3.27275200	3.37464000	-0.68251800
H	-2.20747900	3.19050000	-2.06058400
H	-3.80468500	1.31875400	-1.83089400
H	-2.16235500	0.73917500	-1.76520200
H	-2.27787300	0.51485600	0.69359000
H	-3.88433100	1.21929000	0.71763400
C	-3.94843100	-1.62690800	1.16649000
C	-2.62891100	-2.09823900	1.79615400
C	-1.79398800	-3.01656000	0.88794400
C	-1.01555800	-2.28180000	-0.17166500
O	-0.49523200	-1.19567000	0.01415100
H	0.33882000	0.60137900	-2.59070300
N	0.56889100	0.65229100	-1.59932400
H	0.20144700	1.53692700	-1.19258400
H	0.14270800	-0.15977800	-1.06933300
H	1.58313200	0.62052400	-1.46854900
O	-0.84950600	-2.86112500	-1.36912300
H	-1.30307600	-3.71354100	-1.41836400
H	-2.42272000	-3.78852600	0.43238500
H	-1.03909700	-3.54069900	1.48699400
H	-2.00945200	-1.25122700	2.09927200
H	-2.85803600	-2.65515100	2.70742100
H	-4.50331300	-1.06164200	1.92232700
H	-4.56064100	-2.50749300	0.94170400
H	-4.82072700	-0.64711600	-0.53520000
H	-3.25153900	-1.31444800	-0.86838600

conf_13

C	-4.30406900	-0.11989700	-0.40930300
C	-4.38465400	1.24168900	0.29890800
C	-3.02631600	1.84923800	0.68404600
C	-2.21944200	2.34423900	-0.52785200
C	-0.98509900	3.12582100	-0.17290500
C	0.21331600	2.97838600	-0.75249600
C	1.42578500	3.82937500	-0.48227400
C	2.54173300	3.10089100	0.29159300

C	3.06480300	1.83117400	-0.38893100
C	3.99518000	1.00367600	0.50595500
C	4.35895900	-0.37545200	-0.05938400
C	3.18843600	-1.36662100	-0.06753400
C	3.52412500	-2.74719200	-0.64867800
C	4.56994700	-3.52669100	0.15193600
H	4.25962200	-3.64538400	1.19514300
H	4.71535900	-4.52526600	-0.26603500
H	5.54207200	-3.02875300	0.14816700
H	3.86730100	-2.62237600	-1.68186900
H	2.60136400	-3.33502500	-0.70311700
H	2.83115200	-1.50323900	0.96580600
H	2.35378300	-0.95154100	-0.64508700
H	5.17869700	-0.79068700	0.53193100
H	4.74333900	-0.26073300	-1.07962800
H	3.53868200	0.87028500	1.50196500
H	4.90637100	1.57861400	0.69674500
H	3.59725600	2.10297000	-1.30640100
H	2.22620100	1.21201800	-0.72624500
H	2.16906600	2.85921900	1.29743900
H	3.37299100	3.79309900	0.45442400
H	1.82732200	4.17616500	-1.44119800
H	1.12915200	4.72186700	0.07513400
H	0.31566600	2.21043000	-1.51916700
H	-1.11355900	3.91875900	0.56511900
H	-2.87216800	3.00362300	-1.11500400
H	-1.95112700	1.51178900	-1.18438100
H	-2.43839400	1.11676300	1.25361900
H	-3.19679900	2.69208600	1.36191600
H	-5.00290700	1.13966800	1.19702500
H	-4.90577600	1.95548000	-0.34703000
C	-4.04076800	-1.28051300	0.55714100
C	-3.78871200	-2.63259800	-0.12115500
C	-2.50794000	-2.67684400	-0.99577900
C	-1.32806300	-2.16497400	-0.22098600
O	-0.83139700	-1.06535800	-0.39360600
H	0.13108900	0.49452400	2.24042100
N	0.49008500	0.53703300	1.28742600
H	0.04525000	-0.21571100	0.68928300
H	0.25141500	1.45507300	0.85887800
H	1.50548100	0.41800900	1.29463100
O	-0.83887400	-2.92708900	0.76935300
H	-1.30149500	-3.77422000	0.83027500
H	-2.61504200	-2.04787900	-1.87748700
H	-2.32136100	-3.70113800	-1.33210400
H	-3.73670000	-3.41628700	0.64210900
H	-4.63105200	-2.89415700	-0.76696700
H	-3.19429400	-1.03881500	1.21186800
H	-4.90276400	-1.38728700	1.22267500
H	-5.24623700	-0.31157100	-0.93248500
H	-3.53072400	-0.08227900	-1.18212400

conf_24

C	5.21932300	0.24355900	-0.08388000
C	4.07389600	1.18430000	-0.46983600
C	3.56591200	2.00212900	0.72065400
C	2.49683700	3.05516700	0.37312600
C	1.26030600	2.52423900	-0.29756100
C	0.00602800	2.69143100	0.14349500
C	-1.23949900	2.27972200	-0.59073000
C	-2.07747400	1.20579800	0.12553800
C	-3.46683500	1.01111700	-0.48495700
C	-4.27015000	-0.10322400	0.18835200
C	-5.66750600	-0.28904700	-0.40741600
C	-6.46370700	-1.40790100	0.27021400
C	-7.84365500	-1.66419600	-0.34932300
C	-8.81800600	-0.49094000	-0.21318800
H	-8.94431700	-0.20647200	0.83639000
H	-8.47406300	0.39180700	-0.75833600
H	-9.80342400	-0.75241200	-0.60609500
H	-8.28039000	-2.54844200	0.12614300
H	-7.72049700	-1.91842500	-1.40892200
H	-5.87765300	-2.33403100	0.22806800
H	-6.58569000	-1.17013200	1.33496000
H	-5.57829600	-0.50711700	-1.47912200
H	-6.21356500	0.65709000	-0.33170600
H	-4.36191800	0.11208100	1.26092700
H	-3.71669200	-1.04870700	0.10995500
H	-3.36317400	0.79318400	-1.55438500
H	-4.02005900	1.95465400	-0.41644200
H	-2.18504900	1.46654100	1.18731400
H	-1.54558700	0.24436100	0.07653900
H	-1.86464200	3.17142300	-0.71837900
H	-0.98313100	1.93382000	-1.59645100
H	-0.14800400	3.22554200	1.08326200
H	1.40276100	2.02367600	-1.25444000
H	2.95277100	3.78890500	-0.30329600
H	2.21619300	3.60554800	1.27645500
H	4.40882100	2.51791900	1.19172500
H	3.17988200	1.31688300	1.48890500
H	3.25122700	0.60747500	-0.89966800
H	4.41987600	1.86516700	-1.25635500
C	5.68167500	-0.69403200	-1.20718300
C	4.66892000	-1.75492400	-1.66252400
C	4.28749600	-2.76801500	-0.56905500
C	3.28672400	-2.24368600	0.42624000
O	2.33475000	-1.54835100	0.11547800
H	1.09310900	0.10792100	2.57266000
N	0.72393300	-0.04929000	1.63512000
H	1.35069200	-0.72591100	1.10641100
H	-0.22614900	-0.41702300	1.69881400
H	0.69450000	0.85260900	1.11009100
O	3.41981100	-2.58282700	1.71738100
H	4.20616500	-3.12607500	1.86344400
H	5.18070800	-3.13776700	-0.05538700
H	3.80518200	-3.63923000	-1.02864000
H	3.75579500	-1.29424500	-2.04590700
H	5.10291100	-2.31643200	-2.49277500

H	5.95882200	-0.09280500	-2.07913300
H	6.59933400	-1.20179800	-0.88961200
H	6.07606400	0.84340900	0.23974800
H	4.93174400	-0.33752700	0.80273500

conf_40

C	-4.53130700	-0.37353500	0.17659000
C	-4.73488600	1.10298400	-0.19994100
C	-3.48563400	1.82055200	-0.73238400
C	-2.27506900	1.75191900	0.21369800
C	-1.03999500	2.35405400	-0.39474500
C	-0.26105300	3.28952600	0.16253500
C	0.95461300	3.91065000	-0.47074500
C	2.27606400	3.32486900	0.07205000
C	2.61042200	1.95644100	-0.53265400
C	3.65373800	1.16044800	0.25661400
C	3.84519200	-0.27175300	-0.24968200
C	4.87460400	-1.06892100	0.55617400
C	5.01597400	-2.53378600	0.12322600
C	5.54555300	-2.71537300	-1.30173300
H	5.69574500	-3.77273500	-1.53130500
H	4.85713900	-2.31381700	-2.05004900
H	6.50744600	-2.20946900	-1.42879800
H	4.04453300	-3.03492000	0.22141600
H	5.68925700	-3.03890900	0.82264000
H	5.84945200	-0.57289500	0.47973200
H	4.60151400	-1.03557900	1.61882100
H	4.13725600	-0.23620100	-1.30343100
H	2.88209000	-0.80733900	-0.23378700
H	3.38453600	1.14366400	1.32672100
H	4.61247500	1.68880700	0.23241800
H	2.96355000	2.09428100	-1.55958100
H	1.69366000	1.36040100	-0.63698500
H	2.21427600	3.25163200	1.16658000
H	3.09538300	4.01997800	-0.12944000
H	0.91949700	3.77893100	-1.55646400
H	0.93268200	4.98764900	-0.28226700
H	-0.52489800	3.66281800	1.15377400
H	-0.79323300	2.00982900	-1.39970200
H	-2.08667400	0.69801900	0.44965500
H	-2.51032400	2.24690100	1.16263600
H	-3.72917300	2.86937500	-0.92427400
H	-3.19907000	1.39750800	-1.70131200
H	-5.11155100	1.63661400	0.67905400
H	-5.52069500	1.17563500	-0.95892900
C	-3.95498100	-1.22848600	-0.95706600
C	-3.80973100	-2.71791800	-0.61835700
C	-2.92142500	-3.00871000	0.61413200
C	-1.58269800	-2.32502900	0.54238400
O	-1.20320900	-1.50034500	1.35665400
H	-0.00523400	-0.38755100	1.46321200
N	0.60713400	0.45375100	1.65280200
H	1.58144500	0.26192200	1.40981500

H	0.27588200	1.25678200	1.07991200
H	0.54746500	0.69568800	2.64199900
O	-0.77558400	-2.61644000	-0.48552200
H	-1.17703000	-3.26775200	-1.07768500
H	-2.75600600	-4.08783400	0.70499300
H	-3.40025800	-2.67224100	1.53166500
H	-4.79056300	-3.15630600	-0.41630100
H	-3.42187000	-3.25200700	-1.49380000
H	-4.59581400	-1.13868500	-1.84003400
H	-2.97787600	-0.83478800	-1.26188000
H	-3.88455300	-0.43632700	1.05750600
H	-5.49634300	-0.79065200	0.48422100

conf_17

C	4.58984200	-0.51152900	-1.40300800
C	3.90445200	0.76107800	-0.89639500
C	2.80221600	1.24915600	-1.84140700
C	2.04792700	2.49568900	-1.34678700
C	1.22956500	2.29917500	-0.09787800
C	-0.01447100	2.76183400	0.08626200
C	-0.76822300	2.74450000	1.38944100
C	-2.28596900	2.55252000	1.25348400
C	-2.69335400	1.21401800	0.63780400
C	-4.20139300	0.96715600	0.59253900
C	-4.56539600	-0.37345400	-0.04950200
C	-6.07066100	-0.64362700	-0.09861900
C	-6.42751200	-1.98521500	-0.74337900
C	-7.93427100	-2.24479100	-0.78574400
H	-8.16006300	-3.20748300	-1.25029900
H	-8.36077500	-2.25297600	0.22157000
H	-8.45154000	-1.46928400	-1.35819100
H	-5.93173500	-2.79336300	-0.19190400
H	-6.02178400	-2.01488800	-1.76203300
H	-6.47575400	-0.61140700	0.91997900
H	-6.56444700	0.16637900	-0.64891800
H	-4.07511800	-1.18631500	0.50483500
H	-4.16344700	-0.40672800	-1.07210700
H	-4.68291500	1.78105500	0.03883700
H	-4.60443400	1.00790300	1.61055100
H	-2.22303000	0.40299100	1.21787800
H	-2.30395300	1.16089100	-0.38872100
H	-2.70109900	3.36663700	0.64884300
H	-2.74062400	2.64580900	2.24413600
H	-0.34467000	1.98408300	2.05651500
H	-0.59082400	3.70776700	1.88372300
H	-0.50285500	3.27866100	-0.74124000
H	1.72908600	1.82568500	0.74782600
H	1.40469400	2.88249700	-2.14300000
H	2.78767700	3.28200400	-1.14743900
H	2.09616900	0.42800000	-2.02664600
H	3.24520800	1.47600600	-2.81601400
H	4.64958000	1.55578000	-0.77304600
H	3.48295900	0.59323500	0.09944700

C	5.67596600	-1.07318500	-0.47669400
C	5.23213100	-1.45851800	0.94281600
C	4.07015400	-2.48514900	1.00135200
C	2.70430900	-1.86897400	0.88041500
O	1.93055900	-2.06453400	-0.03930000
H	0.61618400	-1.14755200	-0.33989800
N	-0.18252800	-0.51925700	-0.64610600
H	-1.02677800	-0.70749500	-0.10306600
H	0.07872400	0.47873500	-0.49927100
H	-0.38139300	-0.67389600	-1.63454800
O	2.30989700	-1.03315800	1.85984800
H	2.98501200	-0.94678700	2.54707300
H	4.10101100	-3.01466300	1.96027200
H	4.16947800	-3.22980200	0.21195500
H	6.08511300	-1.90227000	1.46035400
H	4.97394300	-0.56132100	1.51654900
H	6.11342100	-1.95785600	-0.95090700
H	6.48931200	-0.34588800	-0.38453800
H	3.83349700	-1.27981900	-1.59371600
H	5.04835600	-0.30208700	-2.37467900

conf_54

C	4.98748900	0.06424200	-0.07122500
C	3.91547400	1.07351500	-0.49320100
C	3.46836500	1.96711800	0.66681500
C	2.48138700	3.08436400	0.27927600
C	1.20914200	2.62408900	-0.37674600
C	-0.02960700	2.90348800	0.05123600
C	-1.30114700	2.55955700	-0.67316400
C	-2.22380900	1.58755800	0.08313500
C	-3.62000700	1.47199600	-0.53178800
C	-4.51257600	0.45767400	0.18561300
C	-5.90771900	0.34453800	-0.43491900
C	-6.85439000	-0.60197600	0.31564200
C	-6.37966400	-2.05866300	0.42256500
C	-6.14026500	-2.73660800	-0.92896900
H	-7.03521600	-2.68689100	-1.55657200
H	-5.32411600	-2.26468200	-1.48377800
H	-5.88377700	-3.79128800	-0.80098400
H	-7.13326900	-2.62445300	0.97955200
H	-5.46565700	-2.10999600	1.02570500
H	-7.02380100	-0.20867600	1.32499500
H	-7.82911500	-0.58533900	-0.18454500
H	-5.81127700	0.02574400	-1.47865000
H	-6.36285300	1.34079000	-0.46641500
H	-4.60919600	0.73937900	1.24252200
H	-4.02182100	-0.52311500	0.17155900
H	-3.52613600	1.19346300	-1.58808700
H	-4.09806600	2.45798800	-0.51622400
H	-2.31709400	1.90410300	1.13091100
H	-1.76775400	0.58672300	0.08307100
H	-1.85345900	3.49130200	-0.84370700
H	-1.06925700	2.15099400	-1.66119600

H	-0.14453200	3.48368100	0.96899000
H	1.31463300	2.07746400	-1.31300900
H	2.99153000	3.75783100	-0.42074300
H	2.24196500	3.68558300	1.16183400
H	4.34673700	2.43443700	1.12344700
H	3.03112700	1.33919100	1.45632600
H	3.05253100	0.54520200	-0.90603800
H	4.31187800	1.69953500	-1.30120100
C	5.37926900	-0.94384000	-1.15975300
C	4.29026100	-1.94114200	-1.58172400
C	3.83186700	-2.88319100	-0.45483300
C	2.87101800	-2.25030500	0.51626000
O	1.97431200	-1.49743800	0.17653500
H	0.86141500	0.32934500	2.57319200
N	0.48000300	0.16850900	1.64117100
H	0.51727100	1.05180300	1.08589500
H	1.05384500	-0.57048700	1.13634500
H	-0.49471200	-0.12503700	1.71648500
O	2.97564300	-2.55188900	1.81935400
H	3.71889900	-3.14668000	1.98845100
H	4.69381600	-3.30000800	0.07592000
H	3.28670700	-3.73182600	-0.88551300
H	3.41502300	-1.42700100	-1.98498200
H	4.68209400	-2.56274700	-2.38982100
H	5.70145900	-0.39575100	-2.05102200
H	6.25601700	-1.50750800	-0.82156100
H	5.88636900	0.60981500	0.23362800
H	4.65759000	-0.46231100	0.83459700

conf_6

C	-3.38536200	-1.36346400	-1.39349800
C	-2.31855800	-2.46732800	-1.38090500
C	-1.09240500	-2.10444700	-0.53573000
C	0.04039000	-3.13730700	-0.61230200
C	1.17736600	-2.84572300	0.32931600
C	2.43131200	-2.56323300	-0.04428700
C	3.61737600	-2.42360700	0.86926400
C	4.67632100	-1.38574800	0.45767900
C	4.24330300	0.08314000	0.55487600
C	3.37260400	0.58513700	-0.60243700
C	2.91192500	2.03668000	-0.43091500
C	1.84984600	2.46716000	-1.44630000
C	1.34222400	3.90274000	-1.26197000
C	0.56796800	4.12357400	0.04134900
H	1.20271900	3.99662200	0.92362900
H	0.16042200	5.13580200	0.08886100
H	-0.27064500	3.42362000	0.11856300
H	0.69485000	4.15390200	-2.10747200
H	2.18887100	4.59622400	-1.31074400
H	2.26758500	2.35485600	-2.45213300
H	0.99437900	1.77806400	-1.39959200
H	3.77822900	2.70368600	-0.49716000
H	2.54139100	2.20102200	0.59584500

H	2.50175300	-0.06656400	-0.74008200
H	3.93490000	0.49843700	-1.53770700
H	5.13516200	0.71514100	0.61097700
H	3.72470300	0.23653700	1.51411800
H	5.01518800	-1.59943200	-0.56252900
H	5.54746800	-1.53607500	1.10074700
H	3.28414800	-2.23542000	1.89760400
H	4.11361900	-3.40215700	0.89667600
H	2.64788500	-2.51652300	-1.11137300
H	0.96067200	-2.95628700	1.39495800
H	0.41753500	-3.19199000	-1.63791100
H	-0.36534700	-4.12821700	-0.37555600
H	-1.38956400	-1.98055300	0.51320500
H	-0.71527300	-1.13174400	-0.87378100
H	-1.99253300	-2.65913200	-2.40825300
H	-2.75250100	-3.40550400	-1.01585600
C	-4.11112400	-1.20156900	-0.05008600
C	-4.87428100	0.11879900	0.11412300
C	-3.97353400	1.37953300	0.03974700
C	-2.73539300	1.22309900	0.87896500
O	-1.61363000	1.12032100	0.41489700
H	-0.21486600	0.55163700	1.05277800
N	0.73898800	0.20797100	1.35842100
H	0.75350900	0.08530100	2.37042900
H	0.97326700	-0.69292600	0.90133900
H	1.44343200	0.90320200	1.09191600
O	-2.87738600	1.14161900	2.21092500
H	-3.80391900	1.22476700	2.47589700
H	-4.53344900	2.25807600	0.37577200
H	-3.64388800	1.56752100	-0.97998000
H	-5.63886600	0.22102000	-0.66039300
H	-5.41265200	0.10633900	1.06815100
H	-4.81476100	-2.02895900	0.08004900
H	-3.39591900	-1.29639600	0.77483600
H	-2.89766700	-0.42618900	-1.68024400
H	-4.12451000	-1.57364100	-2.17244800

conf_88

C	-5.01703600	1.33006800	0.12364500
C	-3.56425200	1.75339300	0.36117300
C	-2.86151200	2.17511000	-0.93204600
C	-1.43666100	2.72856100	-0.74268500
C	-0.47035600	1.80656100	-0.05176300
C	0.71692500	1.42051800	-0.53920200
C	1.74715600	0.61883800	0.20493400
C	2.08875100	-0.73809400	-0.43837600
C	3.31975100	-1.42335000	0.17062600
C	4.64660200	-0.72534900	-0.14092400
C	5.85287500	-1.46508700	0.44663400
C	7.20789200	-0.84312300	0.08881400
C	7.43672100	0.55994600	0.66053900
C	8.83778700	1.09714100	0.36212800
H	9.60717300	0.44703800	0.78887600

H	9.01557900	1.15515500	-0.71603800
H	8.97975900	2.09803200	0.77725700
H	7.27604800	0.53453300	1.74539600
H	6.69251500	1.25504400	0.25650200
H	8.00275400	-1.50382700	0.45249000
H	7.31669200	-0.81197100	-1.00294000
H	5.83398200	-2.50391200	0.09710700
H	5.75156500	-1.50821400	1.53836900
H	4.62859200	0.29964300	0.24079600
H	4.76668300	-0.64730300	-1.22950800
H	3.36716300	-2.45317200	-0.19979000
H	3.18899600	-1.49700000	1.25700900
H	2.24774400	-0.60590500	-1.51720800
H	1.22795700	-1.41029500	-0.31445200
H	2.66003300	1.22159000	0.25323000
H	1.42397200	0.46110200	1.23846500
H	1.00758200	1.76455100	-1.53385600
H	-0.73567200	1.49036300	0.95623700
H	-1.50752100	3.64487400	-0.14326900
H	-1.02708600	3.02922800	-1.71203100
H	-3.45880000	2.94225900	-1.43520000
H	-2.84276400	1.32200900	-1.62532300
H	-3.01328600	0.93477500	0.83069900
H	-3.54645200	2.58761900	1.07223900
C	-5.72840800	0.76675800	1.36122800
C	-5.19373700	-0.56866400	1.89906300
C	-5.33225600	-1.74221800	0.91373600
C	-4.28005200	-1.76210300	-0.16297400
O	-3.10959200	-1.48736800	0.03958000
H	-0.71612200	-0.06471800	-1.26567900
N	-1.14835000	-0.91763400	-1.68614800
H	-1.95378400	-1.22844600	-1.06544400
H	-1.49203400	-0.70480600	-2.62242600
H	-0.43740400	-1.64778500	-1.74159600
O	-4.63052500	-2.13470600	-1.40312700
H	-5.57689500	-2.32314600	-1.46407400
H	-6.33335100	-1.76017700	0.47113400
H	-5.20903100	-2.68966800	1.45228500
H	-4.14669100	-0.48591800	2.19874100
H	-5.75261600	-0.82933800	2.80053000
H	-5.67334300	1.50438800	2.16834500
H	-6.79409600	0.64974600	1.13458100
H	-5.58332000	2.19499200	-0.23657700
H	-5.05628300	0.60525600	-0.70077600

conf_31

C	-4.07293200	-0.50913600	0.50115100
C	-4.35085500	0.93426100	0.95135400
C	-3.21329100	1.93322200	0.68880000
C	-1.84284100	1.46675700	1.21674300
C	-0.75580400	2.48285600	0.97598600
C	-0.03681000	2.56929000	-0.15164000
C	0.91080800	3.68722900	-0.48728900

C	2.16713800	3.30288600	-1.28655900
C	3.10030700	2.28908500	-0.60058300
C	2.83439900	0.83164700	-0.99202200
C	3.72858700	-0.17492100	-0.26370800
C	3.37453600	-1.63719400	-0.55872400
C	4.09818400	-2.65634700	0.33076700
C	3.63818400	-2.63664100	1.79215500
H	3.86471700	-1.68427100	2.28371300
H	2.55996800	-2.82223100	1.86515600
H	4.13803700	-3.41254800	2.37540000
H	5.17845200	-2.48223100	0.28297400
H	3.93336600	-3.65828900	-0.07604500
H	2.28997500	-1.78490100	-0.46502200
H	3.60446400	-1.83965700	-1.60942500
H	3.70623800	0.02113900	0.82200300
H	4.77533600	0.00691100	-0.52923400
H	2.98226200	0.71971900	-2.07146900
H	1.77933200	0.57872500	-0.82889600
H	3.02648300	2.40562900	0.49009700
H	4.13990000	2.52420400	-0.84713700
H	2.71687000	4.22611500	-1.48522200
H	1.87000300	2.91580900	-2.26809400
H	0.35158800	4.42135300	-1.08177000
H	1.19950200	4.20544800	0.43347300
H	-0.21026600	1.82883600	-0.93458300
H	-0.62395200	3.25269800	1.73558200
H	-1.58500200	0.52818200	0.72135200
H	-1.92911300	1.24616300	2.28754700
H	-3.47044700	2.89446300	1.14352900
H	-3.11525100	2.12576400	-0.38417200
H	-4.58001500	0.92213400	2.02216500
H	-5.25280300	1.30297100	0.45191600
C	-3.67931500	-0.63889300	-0.97407400
C	-3.43127000	-2.08071100	-1.44020900
C	-2.37328500	-2.85101700	-0.61651600
C	-1.08609200	-2.09152600	-0.43508600
O	-0.60026000	-1.83774900	0.65293400
H	0.50265200	-0.75187500	1.23159400
N	1.07929600	-0.00466500	1.70265300
H	2.06550900	-0.12295700	1.45480500
H	0.74869200	0.92537200	1.37587600
H	0.96892000	-0.07626800	2.71365600
O	-0.45197500	-1.64988500	-1.53126000
H	-0.93032500	-1.89370400	-2.33618900
H	-2.14151900	-3.80173800	-1.10978900
H	-2.74232100	-3.08509900	0.38020500
H	-4.35787400	-2.65827600	-1.38702100
H	-3.15131200	-2.07479400	-2.50070600
H	-4.46626300	-0.20647400	-1.60000600
H	-2.78326900	-0.03986600	-1.17400900
H	-3.28923600	-0.93598400	1.13507500
H	-4.96838400	-1.11184400	0.68742800

conf_121

C	5.06803400	1.32927900	0.02926800
C	4.23764400	2.46268700	0.65121200
C	2.73372300	2.16994100	0.77081900
C	2.00243100	2.18216000	-0.58184300
C	0.50478600	2.11956900	-0.46701200
C	-0.30796200	1.39288000	-1.24575000
C	-1.80971200	1.41974200	-1.21202400
C	-2.45077500	0.06696300	-0.85708600
C	-3.97019500	0.04718900	-1.03300900
C	-4.59915900	-1.28627000	-0.62228600
C	-6.11214300	-1.36903600	-0.87044900
C	-6.95373200	-0.30655300	-0.15008600
C	-6.81490700	-0.31191000	1.37513400
C	-7.74879600	0.69016600	2.05653700
H	-7.54844600	1.71044300	1.71494800
H	-8.79547900	0.46735900	1.83064400
H	-7.63219700	0.67324500	3.14308500
H	-5.77938200	-0.08674900	1.65672200
H	-7.01985000	-1.32153200	1.75143500
H	-6.70751100	0.69141800	-0.53178500
H	-8.00567200	-0.46891300	-0.40922600
H	-6.45913800	-2.36297000	-0.56678500
H	-6.29738300	-1.29808700	-1.94833800
H	-4.38858300	-1.47228000	0.43781900
H	-4.11103400	-2.09690800	-1.17730600
H	-4.21182800	0.25992600	-2.08096700
H	-4.40478900	0.86290800	-0.44617500
H	-2.22235000	-0.17603200	0.19116400
H	-2.00792900	-0.72381700	-1.47928200
H	-2.15475200	2.18555900	-0.51104000
H	-2.16879700	1.71970600	-2.20378600
H	0.14133900	0.75853600	-2.01306100
H	0.05394500	2.78324400	0.27182900
H	2.36037600	1.37733900	-1.23053400
H	2.25550200	3.12224500	-1.08966400
H	2.27958200	2.92529000	1.42051400
H	2.57720300	1.20623700	1.27407500
H	4.36161000	3.37225400	0.05478600
H	4.63997400	2.69467000	1.64291000
C	5.34119900	0.18503400	1.01226000
C	6.03227100	-1.03685400	0.39558300
C	5.19320100	-1.75748400	-0.69151000
C	3.80672600	-2.03781100	-0.18820600
O	2.81660500	-1.42837100	-0.55654600
H	1.38374800	-1.30791700	0.19666700
N	0.45892300	-0.98910100	0.60970600
H	-0.30301700	-1.59619200	0.30548600
H	0.50476100	-0.98872000	1.62846200
H	0.26970300	-0.02340200	0.25902400
O	3.65342400	-2.96236900	0.77111500
H	4.49287700	-3.38576400	0.99836700
H	5.68072900	-2.69364600	-0.98022100
H	5.09567000	-1.14147300	-1.58330900
H	6.97834000	-0.74361500	-0.06690500

H	6.29045700	-1.74597800	1.18939300
H	5.97077100	0.56095300	1.82443800
H	4.40519300	-0.13008000	1.49008900
H	4.56020700	0.95450600	-0.86445100
H	6.02763300	1.72969500	-0.31282500

conf_111

C	3.76477100	-2.36701900	-0.12488100
C	2.87793800	-2.44803300	-1.37737500
C	2.11128000	-1.15558700	-1.69992400
C	0.92103500	-0.90694100	-0.75932400
C	0.08116500	0.27874300	-1.14158600
C	-0.43920100	1.17222900	-0.28950800
C	-1.38659600	2.28030900	-0.65532000
C	-2.80025800	2.05686200	-0.07941500
C	-3.50726500	0.82913800	-0.65534600
C	-4.92462800	0.64410700	-0.10904400
C	-5.62795600	-0.59009700	-0.68168800
C	-7.08081400	-0.75966600	-0.22165700
C	-7.24799800	-1.01579000	1.27966600
C	-8.70322000	-1.27265900	1.67592200
H	-8.80054900	-1.45195700	2.74963300
H	-9.33635800	-0.41691800	1.42260600
H	-9.10499400	-2.14626000	1.15395300
H	-6.86584300	-0.16189200	1.84914100
H	-6.63140500	-1.87688900	1.56634500
H	-7.65634300	0.13146700	-0.50295700
H	-7.52872000	-1.59481200	-0.77222500
H	-5.05676700	-1.48816700	-0.41272400
H	-5.60658900	-0.53124000	-1.77637800
H	-5.52136900	1.53666800	-0.33530300
H	-4.87926600	0.57534200	0.98317500
H	-2.91839600	-0.07051000	-0.44261400
H	-3.54722700	0.91587700	-1.74882300
H	-3.39786300	2.95180300	-0.28015700
H	-2.73568700	1.96886800	1.01157700
H	-1.01066600	3.23779700	-0.26949500
H	-1.44765700	2.37295700	-1.74513100
H	-0.23196500	1.05163400	0.77576900
H	-0.16945200	0.36787100	-2.19961000
H	1.25371700	-0.80609700	0.27782100
H	0.27757700	-1.79610500	-0.79216500
H	1.73826100	-1.20980800	-2.72790500
H	2.79677300	-0.29803300	-1.67075600
H	2.15127900	-3.25801900	-1.25834900
H	3.50005500	-2.72417700	-2.23506500
C	5.08411800	-1.62937200	-0.38233900
C	5.92759900	-1.36790700	0.87098300
C	5.24937200	-0.43435700	1.90639500
C	4.75015700	0.81888800	1.24670100
O	3.56978000	1.05632500	1.05106100
H	2.89722000	2.12514800	0.03661100
N	2.26816400	2.63942600	-0.64859500

H	1.40770800	2.05390100	-0.77447500
H	2.01018800	3.55410300	-0.27853600
H	2.74102300	2.75920000	-1.54422400
O	5.65170000	1.69925100	0.78925000
H	6.55679500	1.42066300	0.98640300
H	5.95901800	-0.17754400	2.69895100
H	4.39219600	-0.91939100	2.36924200
H	6.15141800	-2.30873100	1.38060100
H	6.89466500	-0.94698300	0.57528200
H	5.68479700	-2.21638100	-1.08363800
H	4.88726900	-0.67780200	-0.89182300
H	3.20725700	-1.88491600	0.68395400
H	3.99393200	-3.37834100	0.22543000

conf_171

C	-4.64404200	-0.41935300	-0.05710600
C	-4.59927900	-0.10166500	1.44495000
C	-3.22373600	0.39156400	1.91621700
C	-2.87646900	1.81045400	1.41361500
C	-1.39900000	2.05173800	1.29534300
C	-0.78771000	2.56901200	0.22134000
C	0.68429100	2.84692100	0.09615400
C	1.24833400	2.47367600	-1.28470300
C	2.77138200	2.62599200	-1.39499400
C	3.58392100	1.65971200	-0.52069400
C	3.38899900	0.18391500	-0.88827800
C	4.24435100	-0.77845300	-0.05505900
C	3.87408300	-0.83333000	1.43293600
C	4.58683500	-1.95385600	2.19276100
H	4.35386400	-2.94354100	1.77979800
H	5.67173300	-1.83752600	2.13339900
H	4.31251800	-1.96199200	3.24989700
H	2.78346600	-0.93034600	1.53984900
H	4.11651800	0.12495300	1.89879800
H	4.18983200	-1.79116500	-0.49394000
H	5.30296600	-0.51401800	-0.15164900
H	3.62774600	0.05128600	-1.94948600
H	2.33240500	-0.08554600	-0.77101300
H	3.33781100	1.81798900	0.53362500
H	4.64619300	1.90749900	-0.62038200
H	3.04507900	3.65435700	-1.13614200
H	3.06409300	2.48766400	-2.44144800
H	0.77237700	3.10871700	-2.03900300
H	0.96216800	1.44657000	-1.53563900
H	0.85905300	3.91771400	0.26089400
H	1.22057200	2.32556700	0.89481900
H	-1.40148900	2.85470900	-0.63411300
H	-0.79561500	1.79862800	2.16850000
H	-3.30789300	2.54358300	2.10468700
H	-3.34395900	2.00318000	0.44379800
H	-2.45687300	-0.31576400	1.58242500
H	-3.17796600	0.37628500	3.00877300
H	-4.87710300	-0.99716200	2.01076400

H	-5.35423400	0.65530500	1.68137200
C	-3.83654700	-1.67122700	-0.46190100
C	-3.11808100	-1.53271100	-1.81039900
C	-1.90553700	-0.58226200	-1.74653400
C	-0.76813300	-1.17534900	-0.96045700
O	-0.31928400	-2.29396900	-1.20459800
H	1.62584400	-3.17268600	1.08138300
N	1.77063000	-3.18755000	0.07164700
H	2.59064200	-2.61125500	-0.14301100
H	1.94454900	-4.14711000	-0.22874400
H	0.88733900	-2.78807100	-0.43890500
O	-0.20633000	-0.48587200	0.02237100
H	-0.61103400	0.40676200	0.16619700
H	-2.18053600	0.38973400	-1.33469800
H	-1.51661000	-0.41040000	-2.75572600
H	-2.77333800	-2.50593900	-2.16603300
H	-3.81077400	-1.14659200	-2.56304100
H	-3.09813600	-1.91420400	0.31194800
H	-4.50439800	-2.53515000	-0.50380500
H	-5.68306900	-0.55018100	-0.36996000
H	-4.28522100	0.45105200	-0.61704500

conf_100

C	-4.96660600	1.03183600	1.60416100
C	-4.16349100	1.75025900	0.50939300
C	-2.64415700	1.67235200	0.69213900
C	-1.87655600	2.48278000	-0.36267900
C	-0.38936100	2.48776400	-0.15025100
C	0.54431800	2.34261600	-1.09822400
C	2.02997100	2.46443800	-0.89172500
C	2.78960900	1.12539400	-0.98623600
C	2.66077400	0.27267900	0.28118300
C	3.15106800	-1.17390500	0.12642400
C	4.62255000	-1.32177500	-0.28797800
C	5.61345100	-0.69958700	0.69944200
C	7.07422900	-0.96151900	0.32222900
C	8.06455100	-0.32885800	1.30090800
H	7.93986500	0.75748800	1.34239800
H	7.92172100	-0.71823300	2.31329700
H	9.09724200	-0.53333700	1.00928800
H	7.25922300	-0.57783900	-0.68810500
H	7.24427000	-2.04313400	0.27375700
H	5.45431900	0.38340400	0.76252200
H	5.42177500	-1.09528000	1.70496600
H	4.77577600	-0.88592200	-1.28160900
H	4.84530900	-2.38900000	-0.39089600
H	2.54547300	-1.70335700	-0.63125400
H	2.98685600	-1.70864700	1.06802500
H	1.62129700	0.27396500	0.63478600
H	3.22046800	0.75708500	1.08613200
H	3.84585500	1.32909600	-1.17610600
H	2.44145500	0.56647100	-1.86756200
H	2.42274800	3.15066200	-1.64850200

H	2.23130300	2.92315200	0.08106700
H	0.21881200	2.18810800	-2.12998600
H	-0.05903900	2.68966300	0.86865700
H	-2.22795500	3.52175400	-0.32088000
H	-2.11637500	2.12661400	-1.37197800
H	-2.31131300	0.62902100	0.66641400
H	-2.38197900	2.04540700	1.68893900
H	-4.46080100	2.80388100	0.49482500
H	-4.42904800	1.35734500	-0.48032000
C	-4.76513600	-0.48958400	1.64686100
C	-5.39359100	-1.22459700	0.44712300
C	-4.57953900	-2.46170200	-0.00979800
C	-3.27329500	-2.02266200	-0.60229800
O	-2.20380500	-2.04286500	-0.02024400
H	-0.92561500	-1.27817800	-0.70622800
N	-0.12053000	-0.77469600	-1.18079200
H	0.77318400	-1.20395400	-0.92389300
H	-0.24563400	-0.83563900	-2.19119700
H	-0.08830900	0.22622400	-0.90504100
O	-3.29130800	-1.49966800	-1.84647600
H	-4.17761000	-1.52237600	-2.23246400
H	-5.14369300	-3.03642100	-0.75096800
H	-4.36282700	-3.11292800	0.83674400
H	-6.40084300	-1.56355500	0.69686900
H	-5.51158700	-0.54106200	-0.40009400
H	-3.69589800	-0.70994900	1.70600800
H	-5.19623600	-0.88745100	2.56908700
H	-4.68905600	1.45169000	2.57638200
H	-6.03124000	1.25280000	1.47218400

11E_NH4

conf_3

C	-4.23301000	-0.25072900	0.26734800
C	-4.62816700	-1.73184400	0.31424600
C	-3.51585200	-2.74279300	0.00820700
C	-2.36192100	-2.71934200	1.02856400
C	-1.29761500	-1.69797200	0.73082000
O	-0.86174400	-1.49870700	-0.38960400
O	-0.75704200	-1.00866700	1.74857100
H	-1.18218200	-1.23199100	2.58760700
H	-1.83544300	-3.68117000	1.00700900
H	-2.74643000	-2.59424500	2.04564200
H	-3.94562500	-3.74671600	0.01760000
H	-3.10365500	-2.59069400	-0.99192800
H	-5.44412800	-1.89962800	-0.39604400
H	-5.04020400	-1.95946400	1.30422900
C	-3.84245700	0.26353400	-1.12404300
C	-3.82169600	1.79708100	-1.22508200
C	-2.86555800	2.51070300	-0.26072300
C	-1.38156400	2.25471700	-0.53896500
C	-0.46738000	2.88483900	0.52630400
C	0.98720700	2.93485000	0.15394500
C	1.98551200	2.35111700	0.82986900

C	3.45063400	2.50790200	0.52216500
C	4.14861800	1.23610900	0.00217600
C	4.04305500	0.02380800	0.93464400
C	4.73935900	-1.23592600	0.40332300
C	4.03708900	-1.89310000	-0.79034700
C	4.74827400	-3.15094700	-1.29399700
H	4.22021100	-3.60337900	-2.13649700
H	4.82007500	-3.89977900	-0.50103500
H	5.76379300	-2.91605600	-1.62240900
H	3.01244900	-2.16363300	-0.49091800
H	3.96923200	-1.17729400	-1.62243800
H	4.80955300	-1.97342700	1.20924000
H	5.77027300	-0.99193300	0.12322400
H	4.47917500	0.29564000	1.90153100
H	2.99099800	-0.21230100	1.14624300
H	5.20700700	1.46457700	-0.15706600
H	3.75631700	0.99987000	-0.99506600
H	3.58519300	3.31238200	-0.20627000
H	3.95703300	2.82979600	1.43962500
H	1.73069100	1.76148700	1.71000400
H	1.23704500	3.55399700	-0.70937000
H	-0.59026700	2.35379700	1.47540200
H	-0.80247000	3.91471000	0.70059500
H	-1.20327100	1.17515000	-0.57613500
H	-1.13006100	2.65466000	-1.52983300
H	-3.09305500	2.22318700	0.77175600
H	-3.04702200	3.58903300	-0.31595600
H	-3.57547300	2.08527400	-2.25355100
H	-4.83588600	2.16804600	-1.04282800
H	-4.56765400	-0.11499900	-1.85275200
H	-2.87547800	-0.15308000	-1.42252000
H	-5.08512000	0.33472000	0.62997800
H	-3.42573800	-0.05125500	0.98420800
N	1.16786500	0.02244400	-1.27792200
H	0.93378200	0.29484000	-2.23243700
H	1.30508800	0.88040200	-0.70411300
H	0.37740900	-0.55380800	-0.87217900
H	2.03191200	-0.52686900	-1.27955400

conf_118

C	4.35817100	0.12252400	0.97298500
C	3.66139600	1.32440800	0.32903300
C	2.97828600	2.23864300	1.34859700
C	2.27081700	3.43264800	0.70018800
C	1.10869500	3.03402400	-0.17377100
O	0.48500300	1.99561900	-0.03952800
O	0.72363900	3.87850600	-1.14033400
H	1.28722100	4.66375200	-1.17403200
H	2.97650500	4.03086500	0.11307200
H	1.85981800	4.10285600	1.46622400
H	2.24994200	1.66842300	1.93148400
H	3.71547200	2.62608400	2.05719300
H	2.91390400	0.97140600	-0.38800400

H	4.39651200	1.90083400	-0.24655100
C	4.94673100	-0.86281200	-0.04796800
C	3.90574100	-1.61200800	-0.89355900
C	3.08330000	-2.63262600	-0.09887400
C	1.97283600	-3.31047000	-0.91130300
C	0.79769600	-2.38722700	-1.30706800
C	0.07276000	-1.83092200	-0.11097900
C	-1.11310000	-2.26541600	0.33635000
C	-1.77203100	-1.83618900	1.61783600
C	-3.29387200	-1.64696300	1.52091100
C	-3.72067900	-0.57180300	0.52064300
C	-5.21904500	-0.26929000	0.51302000
C	-5.60752300	0.79107100	-0.52093000
C	-7.10422600	1.10614900	-0.53113000
H	-7.34815900	1.86433900	-1.27865900
H	-7.43208600	1.47972400	0.44279200
H	-7.69091500	0.21195000	-0.75865800
H	-5.04453500	1.71277600	-0.31977400
H	-5.30312200	0.44830300	-1.51941800
H	-5.52579500	0.06411600	1.51077500
H	-5.77405600	-1.19312000	0.31405700
H	-3.17298700	0.35670000	0.74612600
H	-3.42918300	-0.89477800	-0.48919300
H	-3.67787800	-1.38977800	2.51254900
H	-3.76312900	-2.59869800	1.24792200
H	-1.57091300	-2.60840900	2.37068200
H	-1.29768700	-0.92041700	1.98917600
H	-1.62649000	-3.04502500	-0.22882300
H	0.59987500	-1.07884200	0.47650900
H	0.09866900	-2.95835000	-1.92611700
H	1.18295900	-1.57027100	-1.93056800
H	2.39898000	-3.73658700	-1.82582900
H	1.56292000	-4.14704400	-0.33736000
H	2.64969200	-2.16408500	0.79044300
H	3.76044300	-3.40662000	0.27683900
H	3.24313900	-0.88944200	-1.38303900
H	4.42000200	-2.13676400	-1.70586600
H	5.56542200	-1.59549900	0.48087100
H	5.62502400	-0.31742600	-0.71362200
H	5.16105900	0.48340100	1.62428100
H	3.65052300	-0.39615600	1.62952000
N	-1.30187500	0.59591200	-1.45168000
H	-1.11340600	-0.34414700	-1.03663300
H	-1.13498700	0.57582800	-2.45708000
H	-2.27539900	0.84054800	-1.26602900
H	-0.64908200	1.28272300	-0.97847500

conf_2

C	-3.77148400	0.48947600	0.63605100
C	-3.92687400	-1.01410900	0.87810000
C	-4.50483300	-1.79728700	-0.30748700
C	-3.70842500	-1.62717400	-1.62633100
C	-2.23262200	-1.81319100	-1.41303100

O	-1.41299500	-0.91669600	-1.51569400
O	-1.79308100	-3.02722200	-1.04399900
H	-2.51883300	-3.66309500	-0.97591100
H	-3.84725300	-0.63060200	-2.04049000
H	-4.05945100	-2.34981600	-2.37011000
H	-4.56820500	-2.85924300	-0.04489500
H	-5.53022200	-1.47773200	-0.51050600
H	-4.56730900	-1.18360500	1.74893400
H	-2.94825600	-1.43410600	1.14770700
C	-3.10602200	1.20638800	1.81372300
C	-2.83847100	2.69694100	1.56538800
C	-1.91773900	3.01037800	0.37701000
C	-0.51153200	2.41286200	0.48709000
C	0.39630700	2.83468600	-0.67928700
C	1.84081400	2.46447700	-0.49982400
C	2.62843700	1.88541000	-1.41492500
C	4.10623400	1.63679300	-1.26760600
C	4.47382500	0.15495200	-1.04934700
C	4.20441400	-0.32301200	0.38121300
C	4.29712100	-1.84212800	0.55670700
C	3.92290300	-2.33919500	1.95935100
C	2.43787300	-2.18378200	2.30371200
H	2.21745800	-2.56551500	3.30253100
H	1.81307800	-2.75843000	1.60652000
H	2.12254200	-1.13384800	2.29781700
H	4.19444800	-3.39482800	2.04723700
H	4.52451000	-1.80621800	2.70295400
H	5.31647100	-2.16175100	0.31971300
H	3.66000900	-2.34899700	-0.18515300
H	4.92425100	0.15610300	1.05345400
H	3.22740400	0.04428700	0.71544700
H	5.53123000	0.00486300	-1.28385500
H	3.92734900	-0.46646900	-1.77394600
H	4.60396700	1.99824300	-2.17263100
H	4.49960000	2.22952000	-0.43618000
H	2.19385700	1.61982700	-2.38135300
H	2.29006700	2.76509800	0.44712200
H	0.34526100	3.92735600	-0.77034900
H	0.01323900	2.43496700	-1.62491800
H	-0.05671100	2.72943800	1.43349900
H	-0.57514100	1.31817200	0.52792600
H	-1.82509100	4.09827300	0.29099500
H	-2.37878900	2.67657300	-0.55901800
H	-3.79387200	3.20945700	1.40944200
H	-2.40612400	3.13230800	2.47303300
H	-2.16503800	0.69708400	2.05620000
H	-3.73575000	1.10386100	2.70368800
H	-3.17442500	0.65490100	-0.26418400
H	-4.75414800	0.93551100	0.44112900
N	1.12559100	-0.82422600	-0.64150800
H	1.18765200	-1.16057100	0.32212700
H	1.76080100	-1.38490200	-1.21008700
H	0.13347300	-0.91375400	-1.00265900
H	1.44786600	0.16365300	-0.67617100

conf_117

C	1.52670000	-2.14794900	-1.54500900
C	0.75847000	-2.50627600	-0.26964600
C	-0.75645500	-2.36442800	-0.42870100
C	-1.52914400	-2.63802400	0.86294700
C	-1.34746700	-1.57622200	1.91863700
O	-0.82843500	-0.49343300	1.70997400
O	-1.81206800	-1.82772300	3.14764900
H	-2.19946500	-2.71195800	3.21001900
H	-1.24734300	-3.60772500	1.29055300
H	-2.60753300	-2.69316700	0.66607500
H	-1.00337000	-1.36004200	-0.78128500
H	-1.11590500	-3.05932400	-1.19244400
H	1.09292100	-1.86803900	0.55294000
H	0.99913600	-3.53676400	0.01942700
C	3.04917000	-2.29740200	-1.40555000
C	3.69694900	-1.31500800	-0.41990400
C	3.76167400	0.13125700	-0.93157600
C	4.02634400	1.14907300	0.18440300
C	2.75612200	1.48189400	1.00450200
C	1.81996700	2.36576100	0.22829700
C	0.67638300	1.98141200	-0.35529800
C	-0.17669500	2.86177700	-1.22410800
C	-1.55589800	3.20891800	-0.62885000
C	-2.44766000	2.01145400	-0.27478400
C	-2.75971700	1.07968500	-1.44860400
C	-3.75284400	-0.02534500	-1.08016500
C	-3.99230800	-1.02306500	-2.21375800
H	-4.70889600	-1.79555700	-1.92383100
H	-3.06267200	-1.51940800	-2.50802300
H	-4.39047800	-0.52113300	-3.09964400
H	-3.38301900	-0.55431100	-0.19278700
H	-4.70365100	0.43001200	-0.78207700
H	-1.83510100	0.62083400	-1.81794500
H	-3.16110900	1.66497400	-2.28431400
H	-1.98864800	1.40943900	0.51819500
H	-3.38972600	2.39169300	0.13603900
H	-2.08516500	3.84512100	-1.34509200
H	-1.40604900	3.83217100	0.26318500
H	0.35945800	3.79080100	-1.43565200
H	-0.32048400	2.36186800	-2.18797700
H	0.34756700	0.95073800	-0.22353000
H	2.16384500	3.38725600	0.06446600
H	3.06326500	1.98790300	1.92803300
H	2.25360300	0.55056700	1.28998900
H	4.79491700	0.76272400	0.86121700
H	4.42311000	2.07903500	-0.23429500
H	2.83244000	0.39922300	-1.44497300
H	4.55009600	0.20260300	-1.68664500
H	3.16154000	-1.35085200	0.53549900
H	4.71465000	-1.64921900	-0.19479500
H	3.51268700	-2.17312600	-2.39004100
H	3.27257800	-3.32325400	-1.09260200

H	1.17974600	-2.78957300	-2.36206700
H	1.27517000	-1.12234100	-1.84032600
N	-0.25372600	1.93750000	2.64233800
H	-1.07688100	2.52544900	2.77310000
H	0.25852500	2.24036000	1.78215000
H	0.35446800	2.00969500	3.45761700
H	-0.54566600	0.93499100	2.47748100

conf_36

C	4.67197400	-0.61240100	-0.27382600
C	3.74989400	-1.54604900	-1.06685700
C	3.47519900	-2.88923000	-0.37675200
C	2.65957900	-2.78349500	0.92190900
C	1.28079300	-2.19491100	0.77450400
O	0.75316500	-1.49632600	1.62369300
O	0.57615000	-2.47878700	-0.32998400
H	1.09313100	-3.01833500	-0.94585200
H	3.17385700	-2.19067900	1.67748200
H	2.51627000	-3.78083000	1.35667300
H	2.98197900	-3.57739700	-1.07805000
H	4.42160700	-3.37853500	-0.13109400
H	4.20588600	-1.74586900	-2.04158600
H	2.80038300	-1.04120500	-1.28059500
C	4.92683400	0.72877500	-0.97930000
C	3.70859100	1.66290000	-1.02065000
C	3.37859100	2.30368200	0.33477600
C	1.98929300	2.95162500	0.38966100
C	0.86288200	1.90763200	0.48959400
C	-0.50779000	2.51821200	0.54401000
C	-1.47798800	2.30658700	-0.35470700
C	-2.85323300	2.91457300	-0.31447200
C	-3.94336800	1.92500000	0.14496900
C	-4.08389100	0.67399200	-0.72814500
C	-5.04010300	-0.36941500	-0.14498800
C	-5.17654100	-1.62793900	-1.00575300
C	-6.12521100	-2.66581200	-0.40442900
H	-5.78149300	-2.99635500	0.58096100
H	-6.20401000	-3.54901900	-1.04195500
H	-7.13073300	-2.25415200	-0.28186800
H	-5.52906500	-1.34020100	-2.00203300
H	-4.18558500	-2.07671400	-1.15634200
H	-6.02731300	0.08346800	-0.00053100
H	-4.70849700	-0.65631500	0.86651200
H	-4.43056300	0.96804400	-1.72472800
H	-3.10264700	0.20893600	-0.89846900
H	-4.90425800	2.44692900	0.17689300
H	-3.73831600	1.63428400	1.18528300
H	-2.85223500	3.77899900	0.35487300
H	-3.10804100	3.28631800	-1.31281700
H	-1.25973800	1.64613800	-1.19395500
H	-0.69663400	3.21200200	1.36435700
H	1.04332000	1.31334100	1.39622700
H	0.91552500	1.21397900	-0.35592900

H	1.92685000	3.61941000	1.25489000
H	1.82912700	3.57498000	-0.49653800
H	4.14058800	3.05606700	0.55968600
H	3.44685700	1.55822200	1.13601500
H	3.89101200	2.46089300	-1.74717900
H	2.84043300	1.11383500	-1.40125700
H	5.26814600	0.52919900	-2.00074800
H	5.75300700	1.24421700	-0.47854800
H	4.25832900	-0.42701500	0.72326100
H	5.62852100	-1.12124100	-0.11389100
N	-1.64646900	-0.29484300	1.55779500
H	-1.94169900	-0.17378000	2.52666600
H	-1.50411500	0.64166600	1.12092800
H	-0.73165100	-0.83606000	1.52749100
H	-2.38050500	-0.78476800	1.04425700

conf_55

C	4.38972300	1.30941900	-0.83572600
C	5.62777800	0.42226000	-1.01565900
C	5.54408200	-0.99218900	-0.42836300
C	4.47655200	-1.87851800	-1.09913500
C	3.09599900	-1.72566000	-0.52196000
O	2.87620800	-1.69109000	0.67732300
O	2.04446200	-1.67888500	-1.35433800
H	2.32735300	-1.70283400	-2.27849700
H	4.72715100	-2.93404200	-0.93954100
H	4.46511300	-1.71724800	-2.18151400
H	6.51058100	-1.48249200	-0.56276900
H	5.35592800	-0.96725000	0.64745800
H	6.48739000	0.92652900	-0.56267400
H	5.85681700	0.34074300	-2.08456700
C	4.07909300	1.68167900	0.61949200
C	3.10722000	2.86414000	0.75586800
C	1.73527400	2.67544200	0.09585200
C	0.88633000	1.56473000	0.71899200
C	-0.46840900	1.38510500	0.01236700
C	-1.40473500	0.45653200	0.72960900
C	-1.88540200	-0.69612200	0.24585400
C	-2.88728400	-1.58245300	0.93029500
C	-4.24460000	-1.61198000	0.19712100
C	-4.96586900	-0.26346300	0.19341100
C	-6.32839200	-0.31541300	-0.50107200
C	-7.05440500	1.03218900	-0.51029300
C	-8.41530900	0.97251900	-1.20586800
H	-8.31141300	0.66052000	-2.24943500
H	-8.91031800	1.94651600	-1.19774900
H	-9.07953200	0.25766400	-0.71119100
H	-7.18456600	1.37779700	0.52208900
H	-6.42141300	1.77869100	-1.00492700
H	-6.96055700	-1.06382300	-0.00748200
H	-6.19631700	-0.66208400	-1.53373400
H	-5.09890900	0.07682900	1.22851800
H	-4.33880800	0.48995100	-0.29666000

H	-4.87754500	-2.36548200	0.67661900
H	-4.08933900	-1.95190300	-0.83354700
H	-2.50348900	-2.61074700	0.97534400
H	-3.04142400	-1.24566400	1.96201000
H	-1.58424800	-1.00101200	-0.75724900
H	-1.74043600	0.78435600	1.71536600
H	-0.30569400	1.03240900	-1.01071300
H	-0.95148500	2.36639900	-0.06708700
H	1.44017100	0.62058400	0.67948600
H	0.72159700	1.79463000	1.78015900
H	1.85814700	2.47714500	-0.97470300
H	1.18048800	3.61703600	0.16330400
H	2.96467300	3.08817100	1.81926500
H	3.57959000	3.75156300	0.32140700
H	5.01581400	1.95367800	1.11826900
H	3.69738100	0.80931600	1.15931200
H	4.55331200	2.23070000	-1.40558300
H	3.51343700	0.83920100	-1.30109200
N	0.56676300	-1.67066600	2.03208700
H	0.26168600	-2.61836300	2.25306200
H	0.74895100	-1.16580300	2.89977100
H	1.45457600	-1.69461400	1.44688300
H	-0.18324400	-1.17189500	1.49917500

conf_18

C	-4.12004500	-0.26190100	-0.16866300
C	-4.55494200	-1.53760200	-0.90120400
C	-3.42905700	-2.50665800	-1.28745500
C	-2.71094400	-3.13725800	-0.08009200
C	-1.65629700	-2.26362200	0.54309000
O	-0.84307400	-1.62182300	-0.09796800
O	-1.56067000	-2.22486700	1.88539900
H	-2.25305000	-2.75243600	2.30574400
H	-2.17273200	-4.03588100	-0.40487000
H	-3.43716500	-3.45992800	0.67265200
H	-3.85593800	-3.32249500	-1.87493000
H	-2.68657500	-2.02142700	-1.92490800
H	-5.08843500	-1.25521800	-1.81450400
H	-5.28199700	-2.07759400	-0.28370900
C	-3.29839200	0.70187100	-1.03092600
C	-2.92504900	2.01263600	-0.32702200
C	-1.99217300	1.82087900	0.87434000
C	-1.43365700	3.12540300	1.45728600
C	-0.39443000	3.82861000	0.56255100
C	0.91832000	3.10407200	0.46756400
C	1.47141500	2.63952800	-0.65903000
C	2.82434000	1.99447500	-0.77302600
C	2.83001000	0.74598700	-1.67749500
C	4.08978000	-0.11584700	-1.52779900
C	4.13313800	-0.92667000	-0.22806200
C	5.40675500	-1.75819900	-0.05106100
C	5.41189300	-2.57398500	1.24195100
H	6.33089400	-3.15509000	1.34121000

H	4.57356600	-3.27765200	1.27003700
H	5.33746600	-1.92512300	2.12067800
H	5.51643000	-2.42603500	-0.91194900
H	6.27232600	-1.08776200	-0.07001400
H	3.27041500	-1.61250700	-0.21008300
H	4.04616700	-0.25421600	0.63930700
H	4.15496200	-0.80920300	-2.37160900
H	4.97821700	0.52276800	-1.58704700
H	2.72639200	1.07263400	-2.71603400
H	1.94322700	0.12607000	-1.48552400
H	3.53061500	2.72009100	-1.19330200
H	3.21037300	1.76582200	0.22746000
H	0.92212500	2.76091000	-1.59219300
H	1.48263500	3.02084300	1.40036500
H	-0.20186400	4.82490500	0.97596100
H	-0.79896700	3.98577700	-0.44062600
H	-2.26216500	3.81533900	1.64483600
H	-0.98104900	2.92908000	2.43701000
H	-1.16819900	1.17313500	0.55514600
H	-2.51909700	1.27874200	1.66538300
H	-2.44794000	2.67152400	-1.05875900
H	-3.83357900	2.53015700	0.00214800
H	-3.87484100	0.93474700	-1.93269700
H	-2.38033600	0.21093400	-1.37191400
H	-5.01866400	0.25819700	0.18063300
H	-3.56751500	-0.52075800	0.74306000
N	1.03421100	-0.10800600	1.10733000
H	0.30050400	-0.72772500	0.65234100
H	1.97170500	-0.47235300	0.91520700
H	0.95719800	0.85267800	0.72131900
H	0.87137900	-0.08171400	2.11373300

conf_0

C	3.19891500	-0.68283600	-0.45043300
C	3.70854900	-2.12504400	-0.44292900
C	2.70948100	-3.14885200	-0.99821200
C	1.46435600	-3.37545700	-0.11237900
C	0.50563900	-2.21937300	-0.02031600
O	0.10361300	-1.75219100	1.03206200
O	0.04349200	-1.68762900	-1.16280400
H	0.45276600	-2.10432700	-1.93432300
H	1.75997200	-3.62482300	0.90674800
H	0.89070200	-4.22550000	-0.50182300
H	2.41511700	-2.86611100	-2.01848200
H	3.20167700	-4.11872000	-1.09972600
H	3.97919000	-2.41277700	0.57968700
H	4.62962400	-2.18716500	-1.03055000
C	4.17077500	0.33578000	0.15224700
C	3.57373500	1.74913500	0.20969000
C	2.43380100	1.89248400	1.22738500
C	1.68930600	3.23322400	1.16871700
C	1.00667100	3.54593200	-0.17903900
C	0.09146800	2.46535400	-0.68074700

C	-1.21419100	2.61384400	-0.93897800
C	-2.11212500	1.55108400	-1.50988900
C	-3.51516000	1.52953600	-0.87496400
C	-4.30508500	0.25320000	-1.19611200
C	-3.82354800	-0.99281100	-0.43667200
C	-4.21451300	-1.01272500	1.04594000
C	-3.62560600	-2.19900200	1.81213700
H	-3.97040900	-3.14213000	1.38053900
H	-3.92490200	-2.18980900	2.86272700
H	-2.53112600	-2.21110100	1.77008100
H	-3.93297200	-0.06605600	1.53705700
H	-5.30609900	-1.02946300	1.12543900
H	-2.73584500	-1.10053700	-0.53845400
H	-4.24518800	-1.88630600	-0.90764100
H	-5.36425700	0.41583200	-0.97298400
H	-4.24723300	0.06824900	-2.27377700
H	-4.07033900	2.40481400	-1.22400800
H	-3.44420200	1.66031600	0.21286900
H	-2.23200600	1.72805000	-2.58574600
H	-1.62423500	0.57387900	-1.42629900
H	-1.67321500	3.58561100	-0.75718200
H	0.55271300	1.50331000	-0.90880100
H	1.77788100	3.72007300	-0.93745300
H	0.45066500	4.48301300	-0.08960000
H	0.93088300	3.25824300	1.96188700
H	2.38560900	4.04570600	1.39838200
H	2.84250000	1.75855100	2.23472000
H	1.71679500	1.07463500	1.08797200
H	4.35784300	2.47119000	0.45832300
H	3.22178000	2.01763600	-0.79254000
H	5.09153900	0.35214600	-0.43914500
H	4.45746400	0.01559600	1.16112900
H	2.96195300	-0.38552600	-1.48033300
H	2.26379900	-0.62705100	0.11001600
N	-1.15244700	0.55486200	1.59513400
H	-0.93274700	1.30890100	0.91286300
H	-2.16483800	0.40419100	1.61765400
H	-0.67853200	-0.34860600	1.30654100
H	-0.82683600	0.82976500	2.52155900

conf_64

C	-4.55913400	-0.28596100	0.09648600
C	-5.01976400	-1.72820700	-0.15310400
C	-3.97041700	-2.69882200	-0.70977700
C	-2.83725400	-3.04096900	0.27761900
C	-1.76955300	-2.00112900	0.47100500
O	-1.33114400	-1.67089200	1.56081700
O	-1.21998500	-1.44886300	-0.62072000
H	-1.66616600	-1.74994300	-1.42519200
H	-2.30786200	-3.93704400	-0.07155600
H	-3.23994000	-3.27455200	1.26336200
H	-4.46425400	-3.63985300	-0.96229600
H	-3.56964900	-2.32618400	-1.66192400

H	-5.86296200	-1.70734300	-0.85085600
H	-5.40984500	-2.14788000	0.78053900
C	-4.16230700	0.47999700	-1.17208700
C	-4.12619400	2.00389600	-0.98879100
C	-3.15604300	2.52371900	0.07952000
C	-1.68434400	2.20213000	-0.19903000
C	-0.74415100	2.93498200	0.75917900
C	0.73098300	2.70306100	0.56292000
C	1.30244900	1.86840900	-0.31541400
C	2.78153800	1.71767900	-0.53109900
C	3.32318900	0.32649600	-0.15777900
C	4.76855500	0.09704700	-0.60346400
C	5.31660500	-1.26674600	-0.17424800
C	6.73674200	-1.56075700	-0.67542500
C	7.80523600	-0.61978600	-0.11272600
H	7.80299600	-0.63286600	0.98199500
H	7.65244200	0.41344700	-0.43477200
H	8.80212700	-0.91868800	-0.44429600
H	6.99180800	-2.59191900	-0.41126600
H	6.74658300	-1.51631400	-1.77072700
H	4.64158100	-2.05072500	-0.53932000
H	5.30375700	-1.33265100	0.92227100
H	4.82227200	0.18382100	-1.69517000
H	5.39575700	0.89832900	-0.20000900
H	2.68028900	-0.44994100	-0.59324700
H	3.27809000	0.20570100	0.93498700
H	2.99323200	1.89457700	-1.59224800
H	3.32431300	2.48628900	0.02715200
H	0.66732200	1.25942000	-0.95717700
H	1.38532300	3.32265100	1.17712200
H	-1.01596700	2.69836800	1.79964500
H	-0.91949900	4.01487100	0.67889200
H	-1.52027500	1.12106700	-0.13851300
H	-1.43894900	2.48931200	-1.22799000
H	-3.43576700	2.13311300	1.06488300
H	-3.27317900	3.61071100	0.14523200
H	-3.87682100	2.46927500	-1.94877300
H	-5.13590500	2.34632900	-0.73826200
H	-4.88430800	0.24973400	-1.96338600
H	-3.19072700	0.13546200	-1.54278600
H	-5.38803000	0.24453400	0.57725800
H	-3.74358500	-0.26568300	0.82890200
N	0.71352000	-0.05459200	2.16139000
H	1.58096900	-0.59158800	2.13206000
H	0.81347200	0.75539100	1.50838800
H	-0.10040800	-0.67248700	1.86244600
H	0.55935300	0.28320800	3.11141700

conf_27

C	-3.89631500	-0.88993000	-0.02011900
C	-4.26482700	-2.09776200	-0.88364100
C	-3.49671400	-3.38145100	-0.54119100
C	-1.96952400	-3.29597000	-0.75524200

C	-1.22507900	-2.41648400	0.21248300
O	-0.41516400	-1.56555900	-0.11695200
O	-1.44982400	-2.58695000	1.52529900
H	-2.13049100	-3.25630000	1.68371200
H	-1.73808900	-2.93492900	-1.75761300
H	-1.53197400	-4.29763800	-0.66334800
H	-3.72737900	-3.68815300	0.48867800
H	-3.86059300	-4.19800100	-1.16883300
H	-4.09431900	-1.85362400	-1.93853500
H	-5.33559600	-2.30307900	-0.78760300
C	-4.70261500	0.37745000	-0.31966200
C	-4.13461300	1.60666300	0.40499800
C	-2.83143400	2.13030000	-0.21278500
C	-2.10512900	3.14476300	0.67404200
C	-0.89854000	3.83346900	0.00800400
C	0.12725000	2.90981700	-0.59014200
C	1.42175400	2.86225600	-0.24648300
C	2.48057000	2.04987000	-0.93708100
C	3.22417300	1.06042300	-0.02343900
C	4.46476700	0.45063100	-0.67984900
C	5.17117800	-0.57886200	0.20394500
C	6.42174200	-1.17931600	-0.44362100
C	7.11775900	-2.21050000	0.44583100
H	8.00589800	-2.62034600	-0.04046000
H	6.45074100	-3.04655000	0.67800100
H	7.43425000	-1.76475900	1.39363200
H	6.14532900	-1.64250600	-1.39789200
H	7.12169600	-0.37214100	-0.68757900
H	4.47089500	-1.38823800	0.45086600
H	5.44853700	-0.11117100	1.15745100
H	4.17726400	-0.01784800	-1.62862000
H	5.16378400	1.25539000	-0.93483300
H	2.54878000	0.24004800	0.25555800
H	3.51807100	1.56585300	0.90569100
H	2.04797600	1.51607100	-1.78890600
H	3.21763500	2.74863300	-1.35166700
H	1.76844300	3.50990900	0.56073200
H	-0.20378600	2.28820300	-1.42167700
H	-0.41622500	4.50117700	0.72771600
H	-1.27784800	4.47191900	-0.79942900
H	-2.80946000	3.92056700	0.98997000
H	-1.78357500	2.65508400	1.60532100
H	-3.05592500	2.59094200	-1.18191000
H	-2.16117700	1.29154000	-0.43580700
H	-4.87354600	2.41330400	0.40501800
H	-3.96793500	1.35260700	1.46068400
H	-5.74499000	0.22121200	-0.02631400
H	-4.71101300	0.56314500	-1.40023700
H	-4.02027000	-1.14317100	1.04077600
H	-2.83822800	-0.65774400	-0.16550700
N	0.20526700	0.46936500	1.52480300
H	1.03302900	0.30713000	2.09864400
H	0.37611600	1.29476500	0.90787900
H	0.00786900	-0.38601000	0.92366000
H	-0.59728800	0.65702200	2.12609100

conf_44

C	-4.34894200	-0.11803200	-0.50281100
C	-4.91072000	-1.27875600	-1.32549100
C	-4.71428600	-2.66088800	-0.68853000
C	-3.23952600	-3.07851000	-0.50218400
C	-2.47613300	-2.33212300	0.55766000
O	-1.36400900	-1.85641700	0.39642200
O	-3.03888400	-2.19900100	1.77021600
H	-3.93131900	-2.57292700	1.78855200
H	-2.68362800	-2.96707200	-1.43348700
H	-3.19264500	-4.13866900	-0.22390200
H	-5.25608800	-2.71069900	0.26665000
H	-5.18295600	-3.41950600	-1.31932900
H	-4.44416500	-1.27739800	-2.31735500
H	-5.98156400	-1.12597900	-1.49220400
C	-4.57566700	1.26583300	-1.11869300
C	-3.80098800	2.36560100	-0.37793000
C	-2.28922000	2.32601900	-0.63446500
C	-1.49248500	3.25291300	0.28708200
C	0.00446800	3.36296500	-0.05980200
C	0.74568300	2.05595400	-0.06743600
C	1.78550100	1.75163700	0.72113500
C	2.59553500	0.48858200	0.63861300
C	4.02219100	0.74534900	0.11963500
C	4.87577300	-0.52341600	0.07802900
C	6.29199000	-0.27758800	-0.44670700
C	7.15040000	-1.54438800	-0.49016100
C	8.56369200	-1.28908000	-1.01638700
H	9.15351800	-2.20855600	-1.03679500
H	9.09391400	-0.56743900	-0.38781000
H	8.53878500	-0.88690000	-2.03356500
H	7.20570900	-1.97669000	0.51600300
H	6.65440700	-2.29397000	-1.11818000
H	6.78661000	0.47339100	0.18173200
H	6.23568300	0.15607300	-1.45279400
H	4.93258800	-0.95638000	1.08491400
H	4.38104800	-1.27373600	-0.55145000
H	4.50732100	1.49504600	0.75500600
H	3.95683900	1.18452500	-0.88139700
H	2.67912500	0.02688700	1.63334900
H	2.09740100	-0.23719000	-0.01462600
H	2.13048600	2.49782200	1.43765800
H	0.43779600	1.32418000	-0.81481500
H	0.49029100	4.05385500	0.63487300
H	0.09293300	3.80979500	-1.05774500
H	-1.92690900	4.25700200	0.25416200
H	-1.60245100	2.91959000	1.32920100
H	-2.09768200	2.60085200	-1.67851100
H	-1.92070500	1.29897400	-0.52807300
H	-4.18244100	3.34803600	-0.67178500
H	-3.99558100	2.27723500	0.69943200
H	-5.64554500	1.49502200	-1.10978800

H	-4.27181400	1.25380800	-2.17216100
H	-4.78676300	-0.13263400	0.50382200
H	-3.27217400	-0.25821300	-0.37843200
N	-0.44492500	-0.00796900	2.11427900
H	0.16565400	-0.39623200	2.83308800
H	0.08264600	0.70596300	1.56166700
H	-0.78624600	-0.78599600	1.47088800
H	-1.24805400	0.43484200	2.56080300

conf_68

C	4.06237900	0.36347900	-0.89529700
C	3.32553700	1.66076600	-1.25143000
C	3.20788300	2.66008400	-0.08758700
C	1.98427100	3.57056300	-0.21941400
C	0.69085500	2.82370400	0.00906200
O	0.61504600	1.80218500	0.66740100
O	-0.43401200	3.32655100	-0.51664100
H	-0.25671600	4.12741200	-1.02887900
H	1.95723000	4.06098100	-1.19892300
H	2.00909800	4.37264600	0.52948100
H	3.13281000	2.13441700	0.86525300
H	4.10494900	3.28116600	-0.03426500
H	2.32201900	1.38575400	-1.60169600
H	3.81651600	2.15068800	-2.09798900
C	3.30273900	-0.47952500	0.13619400
C	3.76991600	-1.93351500	0.22233700
C	3.07049800	-2.73825900	1.33053600
C	1.53709400	-2.64650900	1.31985100
C	0.89391500	-3.11453800	0.00545200
C	-0.60597500	-3.01295800	-0.01981200
C	-1.32145800	-2.47198300	-1.01530300
C	-2.82013200	-2.52571000	-1.15816800
C	-3.42305900	-1.35019200	-1.94355600
C	-3.27693700	0.02713700	-1.28699000
C	-4.06800600	0.19563600	0.01349900
C	-3.96487700	1.60269300	0.61627700
C	-4.64019000	1.72705000	1.98323900
H	-4.56918800	2.74428400	2.37389000
H	-4.18711500	1.05636800	2.72296100
H	-5.69993000	1.46706200	1.92177600
H	-2.91253700	1.91396200	0.68611200
H	-4.41484400	2.31418200	-0.08303500
H	-3.76116200	-0.55914000	0.75483000
H	-5.12253700	-0.03265100	-0.17486200
H	-2.21307800	0.24633900	-1.11690800
H	-3.61298800	0.79136400	-1.99495500
H	-4.48498400	-1.55169000	-2.11495000
H	-2.95693100	-1.32227500	-2.93418500
H	-3.06308800	-3.44691700	-1.70193300
H	-3.29340100	-2.63570200	-0.17660700
H	-0.77971300	-2.03699200	-1.85658700
H	-1.13758000	-3.49359100	0.80435700
H	1.17087400	-4.16455900	-0.15439100

H	1.30526400	-2.55911500	-0.84176800
H	1.13708900	-3.24114500	2.14872000
H	1.25303700	-1.60583100	1.51861800
H	3.42736500	-2.38957700	2.30562300
H	3.36762100	-3.78939900	1.25452900
H	3.61516000	-2.41749200	-0.74849600
H	4.84982600	-1.96328500	0.39980300
H	2.23884200	-0.45397300	-0.12679700
H	3.37533000	-0.02003800	1.12838700
H	4.19243900	-0.22287400	-1.81120600
H	5.07125300	0.58659200	-0.53047100
N	-1.06241600	-0.17278900	1.37957200
H	-2.04103400	0.11732400	1.42870700
H	-0.98474600	-0.97418100	0.71750200
H	-0.46946400	0.63754700	1.05206300
H	-0.74375800	-0.47557600	2.29988100

conf_50

C	-2.20456000	-1.13663200	-0.97397300
C	-3.69017500	-0.83513600	-0.77177900
C	-3.97464200	0.45303500	0.01024400
C	-3.55722100	0.40513400	1.49398700
C	-2.08120700	0.38680000	1.77703000
O	-1.55012800	-0.37327600	2.57293400
O	-1.29447000	1.28441900	1.16727400
H	-1.76274600	1.79022300	0.48560300
H	-3.98420900	-0.46830700	1.98748400
H	-3.94911700	1.29018500	2.01093100
H	-3.51062400	1.30842600	-0.49798300
H	-5.04756000	0.65772500	-0.00691700
H	-4.16678200	-1.67652300	-0.25530600
H	-4.17919200	-0.76094400	-1.74800800
C	-1.93593300	-2.44862800	-1.71408900
C	-0.44567300	-2.70600400	-1.96946500
C	0.41233800	-2.73223500	-0.70020000
C	1.86397900	-3.14108100	-0.96762900
C	2.77602000	-3.03587200	0.25802500
C	3.16162600	-1.64746500	0.70024500
C	2.91794800	-0.49456800	0.06435000
C	3.45412600	0.84800800	0.48377100
C	2.47276300	2.02156000	0.30641200
C	2.18879200	2.36252300	-1.16354000
C	1.50019400	3.71961200	-1.36658000
C	0.07880800	3.83575700	-0.79965300
C	-0.94047400	2.95096800	-1.52315900
H	-1.95390400	3.10601100	-1.13105500
H	-0.69203100	1.88815900	-1.44912800
H	-0.98653200	3.19341400	-2.58812100
H	0.08535200	3.60555300	0.27185300
H	-0.24246300	4.87835800	-0.87867600
H	1.46895600	3.94119200	-2.43886700
H	2.12558400	4.49714800	-0.91460500
H	3.14239900	2.38107200	-1.70221700

H	1.59782100	1.56672800	-1.62970300
H	2.90341600	2.90356600	0.79258400
H	1.52765100	1.81359700	0.82461900
H	3.81223400	0.79578400	1.51934600
H	4.34564100	1.06480000	-0.11827400
H	2.34360300	-0.51305700	-0.85778500
H	3.78350300	-1.61084600	1.59787000
H	3.71143700	-3.57596000	0.06524000
H	2.31921100	-3.56975400	1.10295900
H	1.87855300	-4.17600000	-1.32155300
H	2.27629700	-2.54152600	-1.78677000
H	-0.02833100	-3.42930200	0.02436300
H	0.39168700	-1.74442400	-0.22796200
H	-0.33486500	-3.65993600	-2.49546900
H	-0.05517700	-1.93779300	-2.64880800
H	-2.46930800	-2.44348100	-2.67033600
H	-2.35452900	-3.27907700	-1.13293600
H	-1.73565400	-0.30890600	-1.52214600
H	-1.70549900	-1.18973600	-0.00320600
N	1.11200500	-0.35784600	2.77855300
H	1.37863900	-0.96828400	3.55131100
H	1.58603100	-0.68096000	1.90862100
H	0.04627000	-0.35803500	2.67006700
H	1.42673900	0.59203900	2.97746200

conf_4

C	-3.57291600	-0.02535000	0.27316000
C	-4.48522500	-0.89430600	-0.59562300
C	-4.11309900	-2.38300400	-0.62408800
C	-2.72629300	-2.69391100	-1.22891700
C	-1.54762000	-2.25332500	-0.40350900
O	-0.61022000	-1.60428900	-0.83463900
O	-1.51780800	-2.60515500	0.89357100
H	-2.31828500	-3.08551500	1.14786300
H	-2.61903600	-2.23193900	-2.21059300
H	-2.62405300	-3.77708400	-1.36759800
H	-4.19773600	-2.80474600	0.38721100
H	-4.84916000	-2.92620500	-1.22100700
H	-4.48295200	-0.50790300	-1.62131200
H	-5.51670900	-0.80758100	-0.23984400
C	-4.00536500	1.44045100	0.37666300
C	-2.95461100	2.30733400	1.08588900
C	-1.72046500	2.60232000	0.22357400
C	-0.55835100	3.20640200	1.01602400
C	0.63150900	3.67930700	0.15763200
C	1.16578500	2.67300900	-0.82496800
C	2.41306700	2.18360300	-0.83819500
C	2.97598900	1.29207900	-1.91264200
C	3.35341900	-0.13168600	-1.46040100
C	4.39433200	-0.19179200	-0.33732200
C	4.75247300	-1.62260400	0.09177600
C	3.64098600	-2.36615900	0.84817200
C	3.39309200	-1.82555000	2.26182000

H	2.54511000	-2.31954100	2.74591400
H	3.22543100	-0.73975100	2.28018900
H	4.26635100	-1.98921000	2.89793300
H	2.71529800	-2.35248100	0.25897200
H	3.90959400	-3.42289200	0.92871700
H	5.64685100	-1.59562400	0.72300400
H	5.02551200	-2.19793900	-0.79880800
H	5.30284600	0.31458600	-0.67930700
H	4.05555600	0.38309700	0.53330500
H	3.74427000	-0.67186800	-2.32851900
H	2.44219600	-0.67171800	-1.17806200
H	2.26078400	1.22664400	-2.73751300
H	3.87380100	1.77610700	-2.31527300
H	3.10037600	2.50281000	-0.05501400
H	0.49518900	2.38280200	-1.63340200
H	1.43908500	4.01962200	0.81209000
H	0.30648500	4.55689500	-0.41448500
H	-0.92113200	4.05963900	1.59739900
H	-0.20642700	2.48269500	1.76626400
H	-2.00425500	3.28799100	-0.58319900
H	-1.38788300	1.68549800	-0.27829900
H	-3.40281200	3.25626600	1.39465900
H	-2.64709900	1.80632300	2.01427100
H	-4.95688800	1.49818800	0.91368900
H	-4.19281900	1.84204700	-0.62617900
H	-3.51731300	-0.44687400	1.28530800
H	-2.55943900	-0.05263500	-0.13563100
N	0.80626300	-0.07200500	0.87647200
H	1.59176000	-0.54153900	1.33451900
H	1.15735400	0.74228500	0.33003000
H	0.30797100	-0.74505500	0.22784200
H	0.15231600	0.26328000	1.58384000

conf_7

C	4.24999600	0.60364000	-0.38585600
C	5.03048900	-0.26800100	0.60627300
C	4.18858800	-1.26623400	1.41670400
C	3.42055400	-2.28695000	0.55743000
C	2.13638500	-1.76445000	-0.03239000
O	1.34795700	-1.05603300	0.56577000
O	1.80735200	-2.13925400	-1.28560700
H	2.49712000	-2.68564600	-1.68561800
H	3.11824200	-3.13513500	1.18366600
H	4.06484500	-2.69198400	-0.22954100
H	4.85408400	-1.82716900	2.07650900
H	3.47639200	-0.75165400	2.06522100
H	5.56348500	0.37688400	1.31232500
H	5.80326000	-0.82237700	0.06200900
C	3.23965100	1.55063900	0.26876100
C	2.23356800	2.14633300	-0.72090800
C	1.01847400	2.75840400	-0.02190400
C	-0.06875000	3.25295500	-0.97921100
C	-1.39702500	3.62588700	-0.29375000

C	-2.02156700	2.53644500	0.54006600
C	-3.21713500	1.97914500	0.30704600
C	-3.94952200	1.05172900	1.23987300
C	-4.85705500	0.02578000	0.54409900
C	-4.14145300	-1.08721900	-0.23072500
C	-3.31802900	-2.03559600	0.65453600
C	-2.94506100	-3.36366800	-0.01980100
C	-1.96849300	-3.24142300	-1.19275900
H	-0.98213400	-2.89874500	-0.85880500
H	-2.34594700	-2.56780600	-1.97204400
H	-1.80795900	-4.20682600	-1.67737400
H	-2.51363500	-4.03479500	0.72875900
H	-3.86385600	-3.84657000	-0.36813500
H	-3.90841800	-2.26049400	1.54880100
H	-2.41104600	-1.54037800	1.02908700
H	-4.89915700	-1.67880700	-0.75524500
H	-3.52950300	-0.64090300	-1.02684800
H	-5.52872200	0.56046400	-0.13601800
H	-5.49870800	-0.43805200	1.29962100
H	-3.24741900	0.56378200	1.92414800
H	-4.58849700	1.67902900	1.87404000
H	-3.76994500	2.29452000	-0.57916100
H	-1.49699700	2.25841000	1.45448000
H	-2.11238200	3.95776800	-1.05127200
H	-1.21727300	4.48728200	0.36086600
H	0.29864700	4.12284900	-1.53153900
H	-0.26634300	2.49430700	-1.75081200
H	1.33692000	3.58721900	0.61984100
H	0.60840500	2.00542200	0.66145900
H	2.71747100	2.88795200	-1.36542300
H	1.89250900	1.34812700	-1.39746500
H	3.77349300	2.35458600	0.78637100
H	2.67973400	1.01255300	1.03763600
H	4.96117800	1.18038400	-0.98538900
H	3.72752700	-0.03738800	-1.10945500
N	-0.76864900	-0.07656700	-0.84680800
H	0.02179300	-0.42204600	-0.23653800
H	-1.35945300	0.63146600	-0.36711000
H	-0.36830400	0.35467000	-1.68002200
H	-1.34301700	-0.87713200	-1.11956500

conf_161

C	3.14229800	0.64913000	-1.39532100
C	4.51813900	0.08910300	-1.76229600
C	4.63015100	-1.43806500	-1.67293700
C	4.55861300	-2.00346300	-0.23914000
C	3.22480600	-1.89305800	0.44780800
O	3.08115000	-1.45423000	1.57802800
O	2.13695600	-2.33181000	-0.19926500
H	2.35521800	-2.62049000	-1.09683000
H	5.28897100	-1.51286000	0.40446900
H	4.80786700	-3.07181200	-0.25512000
H	3.87024300	-1.90531100	-2.31513400

H	5.58907500	-1.75620800	-2.08832500
H	5.28157700	0.53939900	-1.11704000
H	4.76723200	0.39183100	-2.78405000
C	3.05307600	2.17451400	-1.48989600
C	1.64485800	2.71765200	-1.21797600
C	1.10177600	2.40883200	0.18173500
C	-0.29709600	2.99128200	0.41016600
C	-0.92133500	2.64316100	1.76466700
C	-1.40266600	1.22788800	1.95350400
C	-1.41230300	0.24203900	1.04758700
C	-2.01708000	-1.11945500	1.25012600
C	-3.21548600	-1.37324700	0.31375000
C	-4.40195400	-0.44547100	0.58773800
C	-5.63521400	-0.74228400	-0.27365200
C	-5.43731400	-0.49282100	-1.77260900
C	-6.71967600	-0.70464600	-2.57946600
H	-6.55684700	-0.52257000	-3.64450400
H	-7.51206100	-0.02973800	-2.24302600
H	-7.08892400	-1.72859000	-2.46927700
H	-5.07571800	0.53265800	-1.91943200
H	-4.65505200	-1.15336400	-2.16237900
H	-6.46510900	-0.12024600	0.07932200
H	-5.94838600	-1.78210300	-0.11660100
H	-4.67581500	-0.53027900	1.64651100
H	-4.09694900	0.59615500	0.43439500
H	-3.53236400	-2.41511000	0.42976500
H	-2.88034700	-1.26620800	-0.72288400
H	-1.26171000	-1.89263800	1.05042700
H	-2.34233200	-1.23470600	2.29154700
H	-1.01056500	0.42951900	0.05400600
H	-1.85574900	1.02586900	2.92614500
H	-1.78581800	3.29461700	1.94325500
H	-0.21869400	2.89524200	2.57161500
H	-0.23895300	4.08076200	0.32945300
H	-0.96751500	2.66889400	-0.39436800
H	1.78941000	2.81236900	0.93643700
H	1.07891600	1.32470800	0.33312200
H	1.64604100	3.80257800	-1.36552700
H	0.95299200	2.31081800	-1.96618400
H	3.37094100	2.49336400	-2.48801300
H	3.76319400	2.62239200	-0.78422500
H	2.38155300	0.20307400	-2.04994800
H	2.88432300	0.35580300	-0.37483100
N	0.91886700	-0.73960100	2.96221700
H	1.29150400	-0.16019100	3.71475200
H	0.23180300	-0.18142500	2.40615100
H	1.72418300	-1.07615000	2.35030100
H	0.43538700	-1.54107600	3.36766700

conf_172

C	-4.82945400	0.37603400	0.32958400
C	-3.62227000	1.07611200	-0.30651800
C	-3.47014600	2.54181900	0.10356200

C	-2.32429300	3.25031400	-0.62818500
C	-0.97097400	2.65213400	-0.34276800
O	-0.68160000	2.07369400	0.68888700
O	-0.01290500	2.77772500	-1.27810000
H	-0.33782300	3.24438600	-2.06017500
H	-2.26300700	4.30249800	-0.32195000
H	-2.49935200	3.25162300	-1.70966000
H	-4.39202100	3.09058500	-0.10861200
H	-3.30295400	2.60911400	1.18165800
H	-3.70715500	1.00406200	-1.39832600
H	-2.70973400	0.53742000	-0.03180700
C	-4.72274100	-1.15149100	0.22053000
C	-3.75574000	-1.75491400	1.25783800
C	-3.03516100	-3.02650100	0.78935000
C	-1.96081900	-2.78495500	-0.28140200
C	-0.74534100	-1.99360400	0.23691500
C	0.24376700	-1.68835500	-0.85001300
C	1.52024900	-2.09074000	-0.88672500
C	2.48275700	-1.78901700	-2.00705400
C	3.37720500	-0.56394700	-1.73428200
C	4.26212900	-0.68751800	-0.49010200
C	4.98501000	0.61602100	-0.13453500
C	5.77904200	0.56588900	1.17659500
C	4.90394100	0.47024000	2.43014500
H	4.30210400	-0.44434100	2.44317300
H	4.23385600	1.33624200	2.50666100
H	5.51090300	0.46146100	3.33748300
H	6.46877500	-0.28414200	1.14661800
H	6.40230000	1.46163700	1.24832200
H	4.26040400	1.44744900	-0.08288800
H	5.65381800	0.88368600	-0.95846100
H	3.66532100	-1.03062400	0.36327300
H	5.00435700	-1.47701900	-0.64908000
H	2.73302600	0.32289900	-1.64984500
H	4.00956000	-0.38142200	-2.60836800
H	3.11541700	-2.66504100	-2.18191000
H	1.92168400	-1.61591700	-2.92937300
H	1.89485400	-2.69466400	-0.06046300
H	-0.14307400	-1.11933800	-1.69698500
H	-0.25912000	-2.55569800	1.04248200
H	-1.10363200	-1.05222600	0.67409300
H	-1.60798700	-3.74460600	-0.67000300
H	-2.39408600	-2.25210200	-1.13506400
H	-3.77259200	-3.73304900	0.39436900
H	-2.57176000	-3.52230600	1.64977300
H	-3.01297200	-1.00341600	1.54894300
H	-4.31306700	-1.97418900	2.17317800
H	-4.40104200	-1.40683800	-0.79570500
H	-5.70894100	-1.60704200	0.34300700
H	-4.90729000	0.65847300	1.38634400
H	-5.74600000	0.73108000	-0.15136100
N	1.53217000	0.57484400	1.01869200
H	1.58289100	0.26133800	1.98787400
H	1.38514700	-0.24541500	0.39381900
H	0.71972200	1.24380300	0.89328100

H	2.41735400	1.02126000	0.77093600

conf_15

C	3.55488100	-0.45377600	0.03173300
C	4.19651600	-1.72665300	-0.52753600
C	3.25420000	-2.62695800	-1.33590400
C	2.02325500	-3.13244300	-0.55814900
C	0.94366400	-2.11799400	-0.29278100
O	0.36361300	-2.00319600	0.77379400
O	0.56129300	-1.31869700	-1.30146600
H	1.11052400	-1.46085900	-2.08547900
H	2.31368100	-3.54962300	0.40652600
H	1.53941900	-3.93997900	-1.12211400
H	2.94753300	-2.11026500	-2.25720900
H	3.80389200	-3.50637400	-1.67899900
H	4.61969000	-2.30865800	0.29896400
H	5.04018000	-1.45494000	-1.17004200
C	4.57340400	0.49809500	0.66566300
C	3.96642700	1.69942500	1.40461100
C	3.06108500	2.60559300	0.54782900
C	1.56949200	2.25306100	0.61076800
C	0.71293900	3.09176000	-0.35222000
C	-0.75791500	2.82405300	-0.21408300
C	-1.53031400	2.24863600	-1.14288500
C	-3.00240200	1.97770900	-1.01599100
C	-3.39994400	0.57645500	-1.52048800
C	-4.79970700	0.14313400	-1.06227800
C	-4.86687600	-0.28790900	0.41056600
C	-4.24791700	-1.66519000	0.68523800
C	-4.19975600	-2.02190600	2.17315500
H	-3.75851500	-3.00707400	2.33819900
H	-5.20343400	-2.02813900	2.60488000
H	-3.61708500	-1.29575700	2.75555300
H	-4.82905700	-2.42294700	0.15084300
H	-3.24127900	-1.73304100	0.24885200
H	-5.91042500	-0.30831100	0.73826600
H	-4.39027200	0.47514400	1.04502900
H	-5.49898900	0.96736600	-1.23535200
H	-5.14727200	-0.68623100	-1.68629600
H	-2.65728200	-0.16626900	-1.20235100
H	-3.35096000	0.57448100	-2.61320000
H	-3.31586800	2.12715100	0.02369700
H	-3.55864500	2.72160100	-1.59838000
H	-1.07082200	1.95497100	-2.08650200
H	-1.22104500	3.15803600	0.71748300
H	1.02739600	2.89676000	-1.38220200
H	0.90322200	4.15437300	-0.16175000
H	1.42451200	1.19264500	0.37825100
H	1.21830300	2.40215700	1.64088100
H	3.39729200	2.57868900	-0.49588200
H	3.17424300	3.64540700	0.86938500
H	3.40387800	1.34417600	2.27746500
H	4.79136000	2.29406400	1.80588100

H	5.24984600	0.86277700	-0.11624800
H	5.19687000	-0.06551700	1.36848700
H	3.02109600	0.06837900	-0.77120800
H	2.80805000	-0.71871600	0.78911700
N	-1.26848900	0.05657700	1.30942600
H	-1.13617700	0.81276200	0.60828000
H	-2.24740400	-0.23640000	1.31541400
H	-0.64883300	-0.77266000	1.06212700
H	-1.01364200	0.40938100	2.23153700

conf_192

C	5.30999900	0.57377500	-0.73360900
C	4.29608200	1.69298000	-0.45924200
C	3.86238800	1.81295100	1.00516600
C	2.75431300	2.85502000	1.19962500
C	1.47032100	2.46390500	0.51083500
O	1.10803000	1.30760200	0.37313900
O	0.67630700	3.43236700	0.03773200
H	1.05945300	4.30860800	0.18134900
H	3.07812500	3.84012100	0.84743900
H	2.50981800	2.96663200	2.26335100
H	3.50311800	0.85326800	1.38126400
H	4.71536600	2.09655200	1.62751100
H	3.41178100	1.52935400	-1.08558500
H	4.72591200	2.64703800	-0.78438100
C	4.85558900	-0.83442900	-0.31914000
C	3.46024300	-1.22592600	-0.81549600
C	3.12713200	-2.70454200	-0.57706900
C	1.62358400	-3.00322500	-0.65038400
C	0.88684800	-2.64191900	0.65133900
C	-0.60785100	-2.77012200	0.55831300
C	-1.49407600	-1.93110700	1.11019200
C	-2.98779900	-2.07062300	1.05626800
C	-3.67092100	-0.93438500	0.27499800
C	-5.19869600	-0.96823300	0.37260600
C	-5.90647200	0.06786500	-0.50804500
C	-5.60975400	1.52661900	-0.14448500
C	-6.41796600	2.52043200	-0.98089400
H	-6.20768500	2.40203700	-2.04835700
H	-6.18966400	3.55346200	-0.70754900
H	-7.49150700	2.36880200	-0.83898900
H	-5.82409400	1.68109600	0.91975100
H	-4.54040700	1.73687700	-0.27148700
H	-6.98686800	-0.09722600	-0.43978900
H	-5.64318800	-0.10663900	-1.55983300
H	-5.54565200	-1.96999000	0.09695700
H	-5.49013400	-0.81959200	1.41895900
H	-3.38800200	-1.00996600	-0.78581100
H	-3.29974100	0.03037000	0.64309200
H	-3.25923900	-3.03487200	0.61686400
H	-3.37783000	-2.07048600	2.08088600
H	-1.11531700	-1.07703700	1.67655900
H	-0.98392100	-3.64506200	0.02753400

H	1.15468300	-1.63314400	0.98519700
H	1.24527000	-3.32095600	1.43505100
H	1.18853800	-2.45904800	-1.50183800
H	1.45634300	-4.06339000	-0.86303600
H	3.49870000	-3.01128800	0.40787800
H	3.66565000	-3.31618900	-1.30644000
H	2.71443700	-0.59856300	-0.31648700
H	3.37629400	-1.01114900	-1.88937800
H	4.87682800	-0.93023800	0.77252500
H	5.58950000	-1.55632300	-0.69234400
H	5.53254900	0.57614600	-1.80599100
H	6.25344800	0.80522200	-0.22799600
N	-0.63791200	-0.01260900	-1.17327700
H	-0.00963900	0.61162200	-0.59136900
H	-0.16755000	-0.26531500	-2.04238400
H	-0.84436100	-0.87606900	-0.62169500
H	-1.51811400	0.45963800	-1.38133600

conf_12

C	-2.44580800	-0.47438600	1.30759300
C	-3.75619300	0.24761100	0.96929800
C	-4.53894800	-0.36173500	-0.20339700
C	-4.06571000	0.10486900	-1.60142700
C	-2.63515400	-0.23347400	-1.89961200
O	-1.74835200	0.59353900	-2.02724900
O	-2.29905600	-1.53106500	-2.01496500
H	-3.05872200	-2.11161700	-1.86845100
H	-4.16167300	1.18693500	-1.68683100
H	-4.69532100	-0.35018400	-2.37402700
H	-4.52005400	-1.45667300	-0.13651200
H	-5.59153600	-0.07933600	-0.13208100
H	-3.56548300	1.30469500	0.75680900
H	-4.39402600	0.22971600	1.85816800
C	-1.67414300	0.15621700	2.47418200
C	-1.15039100	1.57722400	2.22219700
C	-0.18139000	1.68974700	1.04011500
C	0.50324600	3.05686700	0.95562700
C	1.57157700	3.16100000	-0.14611100
C	2.67231200	2.15257800	0.03282700
C	3.34914100	1.50542800	-0.92120800
C	4.43861700	0.49808700	-0.65985000
C	3.96150600	-0.96125100	-0.82500500
C	2.95454700	-1.38784500	0.24899400
C	2.21688600	-2.69843500	-0.05582400
C	0.94809200	-2.90684800	0.78406700
C	1.20357700	-2.95980000	2.29170700
H	0.28073400	-3.16621500	2.83829100
H	1.60635500	-2.01669500	2.66909600
H	1.91876800	-3.74960800	2.53818600
H	0.23199900	-2.09843900	0.57415800
H	0.46034000	-3.83329300	0.46519800
H	2.89520300	-3.54616700	0.08579900
H	1.95261300	-2.73888900	-1.12540800

H	3.47529600	-1.47261700	1.20670500
H	2.21940200	-0.58851300	0.40419900
H	4.82769100	-1.62846000	-0.80167100
H	3.52757100	-1.08833600	-1.82761000
H	5.27436000	0.68007600	-1.34161000
H	4.82453700	0.63306900	0.35507800
H	3.13096000	1.72714100	-1.96883900
H	2.95394200	1.95719400	1.06790000
H	2.00796500	4.16625300	-0.11019100
H	1.11712200	3.06775000	-1.14039400
H	-0.24989200	3.83519500	0.80040300
H	0.97681500	3.27796600	1.91904100
H	-0.71479700	1.49990100	0.10194900
H	0.58139300	0.90539400	1.14064600
H	-1.98405700	2.27214500	2.07545900
H	-0.63825800	1.91662700	3.12862700
H	-0.82561800	-0.49193300	2.72101200
H	-2.31659000	0.16542100	3.36109000
H	-2.67627400	-1.51603700	1.55874400
H	-1.79004400	-0.51702200	0.43343900
N	0.82379000	-0.16838300	-1.93559700
H	-0.18995200	0.14814600	-1.98519700
H	0.87368000	-1.09769100	-1.51168000
H	1.21319400	-0.21814700	-2.87686200
H	1.40449300	0.47601900	-1.37078000

conf_92

C	3.34891300	-0.91881000	-0.51287300
C	3.76144200	-2.38343500	-0.35235400
C	2.64084500	-3.39031700	-0.64547700
C	1.51319300	-3.42015700	0.41120700
C	0.63525100	-2.19893700	0.47082500
O	0.41019200	-1.57348600	1.49363000
O	0.04685500	-1.79004900	-0.66367600
H	0.31786900	-2.33838800	-1.41344100
H	1.92857300	-3.56287200	1.40877400
H	0.85046400	-4.27084700	0.21043100
H	2.23071000	-3.21090200	-1.64890200
H	3.05971700	-4.39844700	-0.68207600
H	4.12688900	-2.55027300	0.66765900
H	4.60437600	-2.59837900	-1.01608700
C	4.42426600	0.09001600	-0.09553500
C	3.90670100	1.53631200	-0.09497300
C	2.88031600	1.80487000	1.01402700
C	2.17979600	3.16737100	0.93692400
C	1.39615900	3.43473100	-0.36670900
C	0.50917100	2.31151000	-0.82878900
C	-0.82287900	2.36890900	-0.96266600
C	-1.67975800	1.29304600	-1.56879000
C	-2.75840800	0.72467000	-0.63196200
C	-3.75997600	-0.17589900	-1.36130200
C	-4.74488700	-0.89698300	-0.43441100
C	-5.68267900	0.02965800	0.34631900

C	-6.71038400	-0.73707700	1.18064400
H	-7.33967500	-1.36675100	0.54546300
H	-7.36679500	-0.05784000	1.72950400
H	-6.22033000	-1.38987100	1.90977800
H	-5.10122800	0.68524900	1.00538900
H	-6.20051200	0.69052600	-0.35882000
H	-4.18588600	-1.52699400	0.27092100
H	-5.35055200	-1.58439100	-1.03469200
H	-4.31542600	0.42655300	-2.08945700
H	-3.20250400	-0.92050100	-1.94111500
H	-3.28656200	1.54816000	-0.13846800
H	-2.28049800	0.12601300	0.15495800
H	-2.18100000	1.72028900	-2.44602600
H	-1.04815900	0.47841400	-1.93441500
H	-1.33698000	3.28665000	-0.67293500
H	1.01127400	1.39941700	-1.15081900
H	2.11159800	3.64001700	-1.17052300
H	0.80479000	4.34652200	-0.24709000
H	1.49380900	3.26766300	1.78937000
H	2.91621000	3.96617000	1.06540500
H	3.38107100	1.71769500	1.98440400
H	2.12520300	1.01066700	0.99605600
H	4.74531500	2.22917100	0.02439400
H	3.47103300	1.75408400	-1.07633000
H	5.28230300	0.00737900	-0.76948400
H	4.79321000	-0.16460900	0.90530300
H	3.06265400	-0.73206400	-1.55585900
H	2.45744100	-0.72918200	0.08746300
N	-0.47796000	0.92688900	1.90678500
H	-0.38613400	1.47154200	1.02115700
H	-1.44438600	0.97212500	2.23022200
H	-0.20391100	-0.08065700	1.71454700
H	0.14289500	1.32184000	2.61357100

conf_35

C	4.90480000	0.18985000	-0.67730700
C	4.77712800	-0.70991100	0.56940800
C	4.40913600	-2.16985400	0.26203500
C	3.08495400	-2.37770000	-0.48961200
C	1.85065600	-1.88285100	0.21397400
O	0.88805500	-1.40844500	-0.36369400
O	1.78649100	-1.99398800	1.55079400
H	2.61008200	-2.35293500	1.91192900
H	2.92254700	-3.44878600	-0.66529000
H	3.09783000	-1.90447600	-1.47080800
H	5.19728900	-2.61668600	-0.34994600
H	4.41436100	-2.75835600	1.19102300
H	4.05017900	-0.27544600	1.26573300
H	5.72595800	-0.72588300	1.11132600
C	3.65316800	1.01563900	-1.01547300
C	3.42388300	2.18915000	-0.05491900
C	2.14438500	2.98410400	-0.34358600
C	0.85762800	2.31179400	0.15011600

C	-0.40250700	3.07282500	-0.29804100
C	-1.66225500	2.70044300	0.43113400
C	-2.77645600	2.21613600	-0.13368300
C	-4.08781700	2.00287100	0.57352300
C	-4.56882200	0.54136400	0.65691700
C	-4.69343500	-0.17020300	-0.69476700
C	-5.12003300	-1.64115500	-0.57969400
C	-4.03830300	-2.58597200	-0.03425100
C	-2.86243500	-2.80396500	-0.99459900
H	-2.09905000	-3.45524500	-0.55941400
H	-2.37740000	-1.86996600	-1.29759000
H	-3.20856500	-3.28037500	-1.91483900
H	-3.68939400	-2.22299700	0.94559300
H	-4.49125700	-3.55651200	0.18574800
H	-5.42596200	-2.00388500	-1.56631300
H	-6.00813600	-1.70465000	0.05752800
H	-5.42848500	0.36905600	-1.30084400
H	-3.75246400	-0.10347900	-1.25269200
H	-5.54423900	0.52870900	1.15314600
H	-3.90469900	-0.01519500	1.33020600
H	-4.02975900	2.42118800	1.58268300
H	-4.85007200	2.58428200	0.04106600
H	-2.76543200	2.03120700	-1.20706700
H	-1.67793400	2.92622200	1.49930900
H	-0.54410200	2.94642800	-1.37572900
H	-0.23259500	4.14419700	-0.13322700
H	0.81089500	1.28179700	-0.22093400
H	0.88760700	2.25604500	1.24654600
H	2.06749100	3.16599000	-1.42229300
H	2.21492100	3.97057400	0.12593100
H	3.40126800	1.83667500	0.98386100
H	4.28602500	2.86040000	-0.12378700
H	2.76639800	0.37497000	-1.04349900
H	3.75999600	1.41810100	-2.02838900
H	5.73900300	0.88271700	-0.53307700
H	5.18296600	-0.42777300	-1.53851200
N	-1.26120000	-0.48638200	0.96665600
H	-1.05495800	-0.44759600	1.96469000
H	-1.52621200	0.46579100	0.64021800
H	-0.40655200	-0.84434200	0.44830800
H	-2.04756000	-1.12101800	0.80896800

conf_224

C	-4.24972200	1.47414600	0.05080000
C	-5.28761800	0.49560700	-0.50681200
C	-5.96244500	-0.39768600	0.54238600
C	-4.97239800	-1.22169200	1.40813100
C	-3.90499400	-1.86854800	0.56860400
O	-2.73570800	-1.52201700	0.58618200
O	-4.26482900	-2.83947600	-0.28095100
H	-5.21082600	-3.03238800	-0.22099100
H	-5.51653500	-1.99112100	1.96482700
H	-4.46535700	-0.58577900	2.13103000

H	-6.56154100	0.20481400	1.22993100
H	-6.66595800	-1.07129700	0.04141100
H	-4.80401500	-0.14089200	-1.26006900
H	-6.06479600	1.04516700	-1.04637300
C	-3.48735600	2.21944300	-1.05233400
C	-2.21098200	2.91291000	-0.55995500
C	-1.09161500	1.93691000	-0.17961400
C	0.18804000	2.63043500	0.29083800
C	1.23942600	1.67339300	0.87158000
C	1.75542400	0.64247800	-0.09163800
C	2.02568300	-0.63439000	0.21041000
C	2.71774200	-1.61673200	-0.69557800
C	4.16346100	-1.91402900	-0.24381700
C	5.09289400	-0.70351500	-0.33742500
C	6.52537800	-1.02282300	0.10031500
C	7.49998400	0.15352000	-0.04039400
C	7.17073400	1.34986500	0.85691600
H	7.93525600	2.12573900	0.77201500
H	7.11946200	1.04860000	1.90813200
H	6.21277700	1.80703000	0.59411600
H	8.50871200	-0.20111200	0.19338200
H	7.52696800	0.47603700	-1.08814700
H	6.89796600	-1.86741100	-0.49055400
H	6.51562600	-1.36012000	1.14458000
H	5.10481000	-0.33579600	-1.37207000
H	4.69132600	0.11051600	0.27296900
H	4.55651100	-2.72838800	-0.86103600
H	4.14696200	-2.28881400	0.78622300
H	2.17105300	-2.57022300	-0.70961000
H	2.73389800	-1.23267400	-1.72240700
H	1.83064800	-0.97749400	1.22776700
H	2.00169700	1.00134000	-1.09266300
H	0.84690100	1.17829000	1.76640400
H	2.09788200	2.27010400	1.20511700
H	0.62530900	3.19578700	-0.54008000
H	-0.06605300	3.36742700	1.05883600
H	-0.86297200	1.31716200	-1.05951600
H	-1.43689200	1.25341700	0.60479200
H	-1.84156500	3.58813100	-1.33877200
H	-2.44633200	3.54464400	0.30441200
H	-4.14960600	2.95388900	-1.52068800
H	-3.21907800	1.50992800	-1.84798900
H	-4.73954900	2.19854000	0.71120400
H	-3.53443900	0.92987200	0.67097100
N	-0.65502400	-1.50235200	-1.09316500
H	0.10891200	-0.95379200	-0.63605400
H	-0.29595800	-2.42381100	-1.34236700
H	-1.48634200	-1.59741100	-0.44199900
H	-0.94521800	-1.01525700	-1.94090400

conf_a

C	-3.49045000	1.15558200	0.58256500
C	-2.67140200	2.44422200	0.48698600

C	-1.60770800	2.58054900	1.58189700
C	-0.52087300	1.48172000	1.53434100
C	0.24965600	1.52763900	0.24557200
O	0.87150000	2.53122800	-0.10022100
O	0.27239800	0.47978200	-0.56807200
H	-0.12721400	-0.33918600	-0.19117200
H	0.20163900	1.66066600	2.33551900
H	-0.94673500	0.48978200	1.68678900
H	-2.07536300	2.53236900	2.56922800
H	-1.11460800	3.55235000	1.50939900
H	-2.18812800	2.49399500	-0.49819100
H	-3.33632200	3.31172200	0.53288300
C	-4.37212400	0.89762600	-0.64134600
C	-5.01924600	-0.49685400	-0.65531800
C	-4.03486600	-1.65628200	-0.43011200
C	-2.83662800	-1.65853500	-1.38574700
C	-1.77122400	-2.71560400	-1.04697700
C	-1.00030000	-2.45456800	0.21929500
C	0.33673900	-2.46897500	0.30777700
C	1.14223800	-2.38924200	1.57609000
C	2.14378100	-1.22055400	1.65440400
C	3.11432700	-1.12302900	0.47469200
C	3.99334200	0.12950700	0.53582600
C	4.92396900	0.29734900	-0.67413100
C	6.05717700	-0.73067800	-0.74278300
H	5.67218900	-1.74834000	-0.83266200
H	6.67035700	-0.68173400	0.16077300
H	6.70886300	-0.54576800	-1.59921300
H	4.33469100	0.20654500	-1.60401900
H	5.36277000	1.30281800	-0.65824600
H	3.34273600	1.00942800	0.62965500
H	4.59983900	0.11702900	1.44808500
H	2.55475500	-1.10634200	-0.46725600
H	3.73721300	-2.02249200	0.44518600
H	1.59274100	-0.27905700	1.74643300
H	2.71430200	-1.31680800	2.58361200
H	1.70134200	-3.32864400	1.67059700
H	0.46919100	-2.34578400	2.43745200
H	0.89985500	-2.64308500	-0.60837400
H	-1.57980100	-2.33001600	1.13352400
H	-1.06603200	-2.81277300	-1.87829500
H	-2.26501200	-3.69093500	-0.94730200
H	-3.19460700	-1.83425400	-2.40556500
H	-2.35962300	-0.67260400	-1.40316300
H	-3.68486900	-1.63290500	0.60686400
H	-4.57222800	-2.60439400	-0.53591500
H	-5.79544400	-0.54691800	0.11550400
H	-5.53185400	-0.63466700	-1.61317400
H	-3.76555500	1.02605200	-1.54521100
H	-5.15614500	1.65929900	-0.69971600
H	-2.80952900	0.30892500	0.70430100
H	-4.10454600	1.17494400	1.49059900
N	2.56171600	2.41746800	-2.06890600
H	1.81328700	2.44650200	-1.26489300
H	2.12304300	2.11530000	-2.93908900

H	3.30510200	1.75529600	-1.82207000
H	2.96804500	3.34409100	-2.20237700

conf_194

C	4.97236000	0.40036000	-0.87089300
C	5.71337900	-0.89107500	-0.48990500
C	4.93622800	-2.21475300	-0.56467700
C	3.88702600	-2.47355200	0.52809300
C	2.51665800	-1.86626400	0.38521600
O	1.77907200	-1.69461000	1.34314900
O	2.05850400	-1.56245800	-0.83283900
H	2.74310900	-1.71025600	-1.50215000
H	4.25865600	-2.17615300	1.51033400
H	3.69179700	-3.55171100	0.59900900
H	4.50231400	-2.33543800	-1.56960800
H	5.66042400	-3.02990100	-0.49232900
H	6.13597900	-0.78583300	0.51615900
H	6.56992200	-0.99861200	-1.16118300
C	4.10344800	1.03922800	0.21811600
C	3.56053100	2.40676300	-0.20941200
C	2.55250900	3.02333000	0.76837500
C	1.20869900	2.28516600	0.87272000
C	0.46747000	2.16824300	-0.47254600
C	-0.99247500	1.83742200	-0.34649000
C	-1.61933300	0.82330300	-0.95784000
C	-3.09902300	0.56595500	-0.93179100
C	-3.48827000	-0.80694400	-0.35141000
C	-4.94577500	-1.20374000	-0.61511700
C	-5.97731200	-0.26866700	0.02186300
C	-7.41930000	-0.74334300	-0.17753500
C	-8.44849700	0.19481000	0.45437900
H	-9.46657500	-0.16859200	0.29739900
H	-8.38434800	1.19876900	0.02412600
H	-8.29084100	0.28501000	1.53349700
H	-7.61959500	-0.83971600	-1.25082000
H	-7.52877400	-1.74892800	0.24479800
H	-5.87685500	0.74080600	-0.39281300
H	-5.77206700	-0.18043500	1.09756700
H	-5.11025300	-1.24770800	-1.69819200
H	-5.10654900	-2.22099700	-0.24157500
H	-3.32712300	-0.79611900	0.73707500
H	-2.82642100	-1.57562600	-0.77052100
H	-3.60465200	1.36600100	-0.38572100
H	-3.46762800	0.61316000	-1.96424800
H	-1.02623600	0.14528600	-1.57447900
H	-1.58885100	2.53205000	0.24707700
H	0.95635500	1.43408100	-1.11873200
H	0.54396300	3.13434100	-0.98811400
H	1.36949000	1.28464600	1.29340400
H	0.57557800	2.82439500	1.58666400
H	2.35072300	4.05525000	0.46213500
H	3.00357300	3.08368500	1.76479100
H	4.40274000	3.09627500	-0.32579600

H	3.10831600	2.32335800	-1.20419400
H	4.69639800	1.15545400	1.13290800
H	3.26768500	0.38893300	0.48458400
H	5.72429900	1.13848300	-1.16772800
H	4.36815600	0.23011600	-1.77291400
N	-0.65642600	-0.66140100	1.67539500
H	0.29790700	-1.08425200	1.47109500
H	-1.37423100	-1.38674200	1.67423900
H	-0.63833100	-0.20439800	2.58727600
H	-0.89361300	0.04022600	0.93985500

conf_238

C	-4.02176600	0.30560500	-1.33533800
C	-2.70715500	-0.27341300	-1.87364300
C	-2.75262400	-1.78084400	-2.15363200
C	-3.03607300	-2.64777600	-0.90268800
C	-2.10295900	-2.30453300	0.22313000
O	-2.45810500	-1.78440000	1.26631700
O	-0.79133500	-2.53791100	0.04930600
H	-0.61656000	-2.94840200	-0.80899800
H	-2.92700200	-3.70876300	-1.15036500
H	-4.04996400	-2.49367700	-0.53882700
H	-3.53276200	-2.00422100	-2.88604200
H	-1.80943600	-2.09361500	-2.61626000
H	-1.88360700	-0.05714000	-1.18458400
H	-2.45211500	0.23739800	-2.80737500
C	-4.00872800	1.83075500	-1.11406300
C	-3.59911800	2.29244400	0.29397100
C	-2.16364500	1.97617500	0.73742700
C	-1.07764800	2.61372300	-0.13293600
C	0.34044300	2.48693600	0.44312900
C	0.80033900	1.07138700	0.65358700
C	1.57829500	0.64576700	1.65732800
C	2.16073600	-0.73651000	1.77727400
C	3.67071200	-0.76807200	1.45999300
C	3.99231900	-0.44313300	0.00087300
C	5.49337300	-0.48480400	-0.29983000
C	5.84487800	-0.23295800	-1.77165000
C	5.47439700	1.16542000	-2.27377500
H	5.93984700	1.93949000	-1.65542600
H	4.39379500	1.33282600	-2.25878900
H	5.81284300	1.31561100	-3.30169100
H	6.92055600	-0.38590100	-1.90328500
H	5.35283200	-0.98990800	-2.39457300
H	5.88938800	-1.46193300	-0.00050700
H	6.00551400	0.25738000	0.32533300
H	3.47390000	-1.15961500	-0.65102800
H	3.59158100	0.54377900	-0.24813800
H	4.05449000	-1.76286200	1.70849800
H	4.19247700	-0.06417100	2.11858900
H	2.02313700	-1.11431500	2.79951800
H	1.63280200	-1.42204100	1.10538600
H	1.90190200	1.37144300	2.40555700

H	0.52372800	0.35383800	-0.12032000
H	0.42287000	3.04598600	1.38172300
H	1.03784300	2.96601900	-0.25544300
H	-1.09603000	2.17042300	-1.13337000
H	-1.30457400	3.67562800	-0.26909300
H	-2.02275700	0.88952500	0.76495500
H	-2.03991700	2.33862900	1.76814700
H	-3.74506000	3.37629900	0.35659600
H	-4.29559000	1.85145800	1.01653900
H	-5.01682400	2.21444100	-1.29250900
H	-3.37595200	2.30753400	-1.87042700
H	-4.81202900	0.05653700	-2.05156600
H	-4.29868300	-0.18592800	-0.39615500
N	-1.01872400	-0.37386400	3.02277100
H	-1.66274800	0.31565600	3.40999800
H	-0.59061000	-0.90765900	3.77854000
H	-1.54747500	-1.01804800	2.36403400
H	-0.27787800	0.11681700	2.47203900

conf_133

C	-4.25142800	1.09812400	-0.55536200
C	-2.89673800	1.63777900	-0.09810000
C	-2.42343400	2.87108600	-0.86839100
C	-1.09426300	3.44102400	-0.31035300
C	-0.07285600	2.34705500	-0.17602700
O	0.24783500	1.84673000	0.88723700
O	0.46366600	1.84356700	-1.30021500
H	0.14114500	2.30771600	-2.08509200
H	-1.24985600	3.85896200	0.68366300
H	-0.71263500	4.23442500	-0.96031900
H	-2.31283100	2.62433200	-1.93082200
H	-3.16990400	3.66763200	-0.81720200
H	-2.14702300	0.84865000	-0.20957800
H	-2.93686500	1.87110800	0.97253400
C	-4.74845100	-0.11667700	0.24259100
C	-3.87231700	-1.38164400	0.14515800
C	-2.76047000	-1.50030600	1.19875200
C	-2.03259800	-2.85258000	1.17998600
C	-1.36122300	-3.22819800	-0.15521500
C	-0.33058900	-2.24934900	-0.64041200
C	0.94090100	-2.55466600	-0.93093500
C	1.96491400	-1.60617000	-1.49014700
C	3.36844800	-1.80286300	-0.88662800
C	4.33050000	-0.64734100	-1.19485500
C	4.04516100	0.63838300	-0.40352500
C	4.44349900	0.56639300	1.07550100
C	4.03056800	1.80450800	1.87415400
H	4.50363500	2.70009900	1.46356100
H	4.32771700	1.72715300	2.92255400
H	2.94866000	1.97296600	1.83731300
H	4.03449500	-0.34340200	1.54653000
H	5.52693700	0.42763300	1.14667000
H	2.98588600	0.90966500	-0.49306600

H	4.59271200	1.46948100	-0.85896200
H	5.35752500	-0.96732400	-0.99236800
H	4.28606600	-0.43199800	-2.26754400
H	3.78391900	-2.73988200	-1.26828500
H	3.29950600	-1.95092600	0.19910100
H	2.03917900	-1.76428700	-2.57313900
H	1.62042800	-0.57388900	-1.36906700
H	1.27024300	-3.58363000	-0.78625700
H	-0.67303400	-1.23029800	-0.82885100
H	-2.13467000	-3.32390700	-0.92551000
H	-0.90255700	-4.21628500	-0.06049100
H	-1.27689600	-2.87046900	1.97643900
H	-2.74707000	-3.64247400	1.43250300
H	-3.20393500	-1.36294300	2.19078200
H	-2.03780400	-0.68542100	1.08456100
H	-4.51572800	-2.26207000	0.24829500
H	-3.44810000	-1.44237700	-0.86410800
H	-5.75063000	-0.36000200	-0.12059800
H	-4.87134200	0.16329900	1.29575000
H	-4.99788100	1.89652400	-0.48146400
H	-4.19457200	0.82964200	-1.61760200
N	1.20024100	-0.55400900	1.61902500
H	0.87943700	-1.29290600	0.96030200
H	2.22335200	-0.55353200	1.65766600
H	0.86995300	0.39149200	1.27730900
H	0.82112100	-0.73442500	2.54828300

conf_b

C	-3.42819700	-0.46505000	-0.10388700
C	-2.31512800	-1.47175300	0.19440300
C	-0.93454800	-1.00129300	-0.26825200
H	-2.55253600	-2.42820100	-0.28775000
H	-2.28702500	-1.67201200	1.27268700
C	0.17912400	-2.00667000	0.03171200
H	-0.69766100	-0.04415700	0.21289000
H	-0.96204800	-0.80193000	-1.34683400
C	1.56034300	-1.53550500	-0.42687400
H	-0.05630800	-2.96357300	-0.45092600
H	0.20463900	-2.20767100	1.11010700
C	2.67487500	-2.54774100	-0.12027900
C	4.02031300	-2.10198500	-0.61450600
C	5.08046500	-1.86042100	0.15527200
C	6.41899300	-1.38941000	-0.33149900
C	6.81467900	-0.01789000	0.23802100
H	6.41953500	-1.34730400	-1.42611100
H	7.18751400	-2.12005500	-0.04638400
C	8.20085500	0.44319000	-0.21580900
H	6.78549600	-0.05911800	1.33397700
H	6.06278700	0.72196100	-0.06064600
C	8.60160200	1.80920000	0.34482300
H	8.22950300	0.47910000	-1.31221400
H	8.94645100	-0.30427800	0.08318500
C	9.98836300	2.27185000	-0.10880200

H	8.57377700	1.77365600	1.44146400
H	7.85579400	2.55713400	0.04618300
C	10.37978300	3.63816700	0.45717000
H	10.01538100	2.30690300	-1.20442100
H	10.73251900	1.52413800	0.19003800
H	9.66961200	4.41097800	0.14669400
H	11.37302000	3.94338500	0.11766900
H	10.39247800	3.62189900	1.55141200
C	-4.80753200	-0.93905100	0.35907300
H	-3.45716300	-0.26426400	-1.18188700
H	-3.19243600	0.49082600	0.37979200
C	-5.91395700	0.07504900	0.05926200
H	-4.77834000	-1.14102500	1.43694000
H	-5.04446400	-1.89389900	-0.12626900
C	-7.28190900	-0.41889600	0.53031100
H	-5.95429700	0.27662700	-1.01499100
H	-5.68829600	1.02824700	0.54574600
C	-8.41225500	0.53171700	0.26681100
H	-7.27093700	-0.62289200	1.60941000
H	-7.53694100	-1.37345100	0.05095300
O	-9.64815500	0.16881600	0.64137600
H	-9.66020500	-0.70609300	1.05520000
O	-8.28693900	1.62384800	-0.26889700
N	-10.21757000	3.32163100	-0.75101300
H	-11.12731200	2.97438800	-0.44221300
H	-10.01088700	4.19664200	-0.26581900
H	-10.25096100	3.50019900	-1.75638300
H	-9.44583900	2.58231300	-0.52987700
H	2.72062300	-2.72680600	0.95952700
H	2.41258800	-3.50744200	-0.58560300
H	1.80460300	-0.58156200	0.05449000
H	1.53822500	-1.33754300	-1.50569000
H	4.11189000	-1.96022300	-1.69175100
H	4.98576500	-1.99866300	1.23294800

conf_d

C	3.05846700	-1.13161100	-0.92014400
C	3.07613300	-2.66499800	-0.87952900
C	1.65405400	-3.24048400	-0.86700600
C	1.00344000	-3.07852800	0.53155800
C	-0.49126700	-3.09906200	0.43788700
O	-1.19346900	-4.03461500	0.20476800
O	-1.05659400	-1.82570900	0.60535400
H	-2.01372900	-1.92991900	0.45639600
H	1.33255900	-2.14755100	0.99670300
H	1.31174700	-3.90426000	1.17555700
H	1.05119400	-2.72465800	-1.62212600
H	1.64617600	-4.29838900	-1.13331000
H	3.61513400	-3.01210700	0.00968900
H	3.62483700	-3.05598700	-1.74014200
C	4.32468800	-0.44493200	-0.40030000
C	4.13203400	1.06704900	-0.21427700
C	3.22019400	1.40485000	0.97341500

C	2.76915700	2.86735900	1.05867500
C	1.97623400	3.38655300	-0.15769000
C	0.77673800	2.56464500	-0.54635300
C	-0.48984000	2.99801200	-0.47602800
C	-1.71745500	2.28413600	-0.97073300
C	-2.93075800	2.45997300	-0.04243700
C	-4.24589700	1.92124600	-0.61977000
C	-4.30187200	0.40396700	-0.83663900
C	-4.20658600	-0.41764100	0.45195100
C	-4.36849600	-1.92151700	0.21357200
H	-5.36157200	-2.14272300	-0.18414000
H	-4.25262200	-2.50279600	1.13226100
H	-3.65838100	-2.30376100	-0.53075700
H	-3.24666100	-0.21482700	0.94954800
H	-4.97229000	-0.07871500	1.15782100
H	-3.51504400	0.08792400	-1.53237500
H	-5.24685700	0.16056400	-1.33244600
H	-5.06472900	2.21324500	0.04605500
H	-4.43569500	2.42560400	-1.57309800
H	-3.05295500	3.52784500	0.16471100
H	-2.73533700	1.99537400	0.93405100
H	-1.98032100	2.70657600	-1.94920300
H	-1.49992600	1.22623400	-1.15749300
H	-0.66255000	3.99750300	-0.07617100
H	0.97269600	1.58733000	-0.99159600
H	2.64315000	3.44014100	-1.02444800
H	1.65733500	4.41224600	0.04495800
H	2.15876800	3.00629000	1.96064500
H	3.64574100	3.50817600	1.19360000
H	3.73740200	1.13730000	1.90116600
H	2.33552600	0.75631100	0.92540300
H	5.09986200	1.55459500	-0.06366400
H	3.72091600	1.48471100	-1.13959200
H	5.15225600	-0.63120300	-1.09089900
H	4.61847400	-0.89354000	0.55668900
H	2.85811300	-0.80034100	-1.94555300
H	2.20295400	-0.78178300	-0.32993200
N	-0.28414900	0.62790400	1.72016700
H	-1.05390500	0.90200100	2.33287100
H	0.56758200	0.51701300	2.27313900
H	-0.52201100	-0.27859200	1.25136000
H	-0.12211300	1.38733400	1.01527000

conf_c

C	3.02705300	-1.99188600	-1.00773900
C	4.13009700	-0.99494700	-1.38501800
C	4.17338100	0.29784400	-0.55630000
C	4.47524200	0.08184700	0.93218600
C	3.28348300	-0.30990500	1.77071500
O	2.14229800	0.05599600	1.60434900
O	3.63590300	-1.10796800	2.80534200
H	2.83997300	-1.26533800	3.33527800
H	5.27064400	-0.65181700	1.07975300

H	4.83552300	1.01781300	1.37600500
H	4.95644800	0.94050800	-0.96757900
H	3.23797400	0.85171000	-0.65257500
H	5.10231100	-1.49405600	-1.30351200
H	4.01637600	-0.72347900	-2.44074500
C	1.60858200	-1.46988400	-1.24528800
C	0.52553500	-2.44924600	-0.78968900
C	-0.88201900	-1.86558300	-0.91901400
C	-1.97621400	-2.79989900	-0.40251600
C	-3.39130300	-2.19112700	-0.49273100
C	-3.50662800	-0.93625700	0.32500600
C	-3.80757500	0.28642500	-0.14224000
C	-3.79474400	1.55498200	0.66353000
C	-2.81339700	2.60005000	0.09368400
C	-1.37180400	2.09829200	-0.00402700
C	-0.38662800	3.19038700	-0.42536200
C	1.03382600	2.66104000	-0.63686000
C	2.03374600	3.76332900	-0.98807500
H	3.03580700	3.35668500	-1.14556400
H	1.74186000	4.28903300	-1.90232800
H	2.09930500	4.50519000	-0.18652000
H	1.01700800	1.91213100	-1.43802800
H	1.36105700	2.13137700	0.26320100
H	-0.74029200	3.66576500	-1.34919900
H	-0.37280200	3.97891600	0.33716100
H	-1.31960000	1.27271100	-0.72239700
H	-1.05785700	1.67985700	0.96027400
H	-3.16007900	2.91619500	-0.89704400
H	-2.85682800	3.48838100	0.73153800
H	-3.52941000	1.32761900	1.70175400
H	-4.79746500	2.00753300	0.67462200
H	-4.00747300	0.39638800	-1.20987000
H	-3.23809100	-1.02975200	1.37940400
H	-4.10726800	-2.94365700	-0.13295200
H	-3.64096500	-1.97921600	-1.53779300
H	-1.76867700	-3.06486100	0.64101700
H	-1.96730000	-3.73803100	-0.96572000
H	-1.08315700	-1.61377900	-1.96834100
H	-0.91639600	-0.92161000	-0.36577500
H	0.70492100	-2.71305200	0.25988800
H	0.59701300	-3.38346900	-1.35987400
H	1.47923700	-1.24763900	-2.31224500
H	1.47039700	-0.52969100	-0.70668900
H	3.13351000	-2.28822600	0.04295900
H	3.17542000	-2.91100500	-1.58575200
N	-6.54064500	-0.72586400	0.75992700
H	-6.74866200	-1.72266200	0.85074300
H	-6.73656700	-0.25576800	1.64650700
H	-7.12909400	-0.32294800	0.02707500
H	-5.50168100	-0.59369800	0.50473400

conf_e

C	-3.38519500	0.13865900	-0.41632100

C	-2.37550000	-0.94462600	-0.03146900
C	-0.94304100	-0.61241700	-0.45484200
H	-2.67516600	-1.89802500	-0.48480100
H	-2.40507300	-1.09929900	1.05457000
C	0.06885800	-1.69396200	-0.07101200
H	-0.64375000	0.34083900	-0.00204300
H	-0.91315000	-0.45669500	-1.54033000
C	1.50132200	-1.35691300	-0.48881400
H	-0.22736900	-2.64701600	-0.52735500
H	0.03604100	-1.85228800	1.01442600
C	2.51579900	-2.44331900	-0.09946800
C	3.91109400	-2.12470800	-0.55221100
C	4.95431800	-1.92897000	0.25286000
C	6.34213800	-1.57455900	-0.19308100
C	6.80562800	-0.20670500	0.33250400
H	6.39193500	-1.58432500	-1.28728600
H	7.04549400	-2.34061600	0.15918400
C	8.23662300	0.14335900	-0.07979300
H	6.72903300	-0.19486900	1.42690800
H	6.11739600	0.56543000	-0.03079600
C	8.70112700	1.50593900	0.43818400
H	8.31249600	0.12680400	-1.17438300
H	8.91912100	-0.63503000	0.28402600
C	10.13165600	1.86118400	0.02525800
H	8.62687300	1.52224400	1.53313100
H	8.01707500	2.28408500	0.07583400
C	10.58528100	3.22525800	0.54848600
H	10.20493200	1.84471800	-1.06866900
H	10.81440700	1.08354700	0.38767200
H	9.93835000	4.02509300	0.17479900
H	11.60866100	3.45336700	0.23932400
H	10.55268200	3.25800100	1.64194600
C	-4.81729900	-0.19429000	0.00848500
H	-3.35730300	0.29344300	-1.50150100
H	-3.08678800	1.09175800	0.03633000
C	-5.82001600	0.89405400	-0.37981800
H	-4.84436700	-0.34771000	1.09520100
H	-5.11587100	-1.14782500	-0.44624500
C	-7.24342000	0.54280800	0.05578100
H	-5.80607700	1.04966600	-1.46195500
H	-5.53366700	1.84772300	0.07185400
C	-8.25524300	1.58950000	-0.31060100
H	-7.28832800	0.40041400	1.14346100
H	-7.56236900	-0.40368500	-0.40009800
O	-9.56060500	1.22483700	0.11861000
H	-10.15255500	1.94589000	-0.15418900
O	-8.10132400	2.61883600	-0.88034300
N	-10.43678800	-0.96136000	1.43635400
H	-9.65556800	-1.59685300	1.61549300
H	-10.08986200	-0.09817300	0.91480000
H	-10.85272600	-0.68623700	2.32968100
H	-11.14001700	-1.44936500	0.87594600
H	2.50464000	-2.58290100	0.98713100
H	2.19439000	-3.39627100	-0.54104000
H	1.80550900	-0.40763800	-0.03329300

H	1.53697200	-1.20098500	-1.57404100
H	4.05653700	-2.03640400	-1.62918700
H	4.80561500	-2.01241900	1.33010600

11Z_NH4

conf_0

C	3.95559700	0.84482600	-0.52104200
C	5.06353800	-0.19478500	-0.73642400
C	4.78752500	-1.60870500	-0.20769900
C	3.62198500	-2.33395400	-0.91203100
C	2.23869100	-1.88644900	-0.53217800
O	1.35098800	-1.62598800	-1.32293700
O	1.94298700	-1.81011100	0.78306000
H	2.71897800	-1.99968200	1.32873400
H	3.66400600	-3.40522900	-0.67817200
H	3.70345400	-2.23939700	-1.99501700
H	4.64095100	-1.59042600	0.88024900
H	5.68055800	-2.21884400	-0.36030900
H	5.98541000	0.16756100	-0.27020000
H	5.27556700	-0.26966500	-1.80827400
C	3.71039800	1.22629700	0.94359400
C	2.67810500	2.34689500	1.12161800
C	1.23177900	1.94676800	0.80662000
C	0.26555000	3.13146200	0.89633500
C	-1.20150400	2.78451600	0.61822300
C	-1.46116200	2.31347100	-0.79467700
C	-2.63533200	1.87128100	-1.26830600
C	-3.91963800	1.77546300	-0.49043300
C	-4.89550400	0.71557000	-1.02332700
C	-4.38277100	-0.72721800	-0.94542700
C	-4.14996800	-1.23832500	0.47949400
C	-3.67112700	-2.69330000	0.53800800
C	-3.32134900	-3.16342700	1.95034800
H	-2.51884000	-2.56025300	2.39027600
H	-4.18671700	-3.07952500	2.61239400
H	-2.99692900	-4.20605700	1.95761100
H	-2.80586600	-2.84415300	-0.13050500
H	-4.44600300	-3.33869900	0.11306900
H	-3.43652000	-0.59124400	1.00918100
H	-5.08055300	-1.15124400	1.05046000
H	-3.45907800	-0.81849300	-1.53544700
H	-5.10362700	-1.38556500	-1.44006700
H	-5.83590400	0.79224900	-0.46899200
H	-5.13533300	0.95143100	-2.06522100
H	-4.41262700	2.75491800	-0.54450700
H	-3.71608800	1.61812700	0.57333800
H	-2.69948800	1.63039500	-2.32824800
H	-0.63784200	2.42036500	-1.49868200
H	-1.81800700	3.67003600	0.81043100
H	-1.55169300	2.03901200	1.34676900
H	0.34025600	3.57881400	1.89212000
H	0.58619500	3.90587800	0.19044300
H	1.18146800	1.50453700	-0.19549000

H	0.92193700	1.16584000	1.51769800
H	2.71990000	2.71564900	2.15155300
H	2.95925600	3.19351400	0.48447000
H	3.39175800	0.35485700	1.52763100
H	4.66097300	1.54962100	1.38067200
H	4.23905100	1.75185900	-1.06537300
H	3.02750300	0.50735300	-0.99408700
N	-0.88491200	-0.70508200	-0.09736500
H	-0.06085100	-1.06910100	-0.65185500
H	-1.26037900	0.17357000	-0.51199900
H	-1.62655000	-1.40805000	-0.05747900
H	-0.55461200	-0.50791600	0.84722100

conf_6

C	-4.11720200	-0.03393100	0.88628200
C	-4.94630100	-1.32406600	0.94473800
C	-4.52716900	-2.45073000	-0.00921700
C	-3.13276100	-3.04364800	0.28128000
C	-1.95789000	-2.20263400	-0.13086900
O	-0.99680400	-1.95966700	0.57595600
O	-1.94290000	-1.71663700	-1.38942100
H	-2.76096600	-1.93151200	-1.85899700
H	-3.02185400	-3.99502000	-0.25414900
H	-3.01592200	-3.25860900	1.34361200
H	-4.60543700	-2.11999900	-1.05316900
H	-5.24504600	-3.26908300	0.08036100
H	-5.99439700	-1.08113300	0.74239600
H	-4.91854800	-1.71745500	1.96646200
C	-4.24754800	0.75164800	-0.42370700
C	-3.50844400	2.09565300	-0.41272600
C	-1.97875500	1.99137600	-0.40905400
C	-1.30070000	3.36217100	-0.32943000
C	0.23169500	3.31763600	-0.33693200
C	0.82843900	2.62999400	0.86759500
C	2.10904500	2.25557200	1.00523400
C	3.20195100	2.44871300	-0.01377800
C	4.40204800	1.50994000	0.16737200
C	4.07650900	0.03103300	-0.05319000
C	5.28100300	-0.90227200	0.07711300
C	4.92964300	-2.37473000	-0.15064800
C	6.13551400	-3.30631700	-0.01871400
H	6.57760600	-3.23694300	0.97909500
H	5.85452200	-4.34841400	-0.18682400
H	6.91188000	-3.04885900	-0.74455700
H	4.48892900	-2.48939000	-1.14970000
H	4.15584700	-2.67603000	0.56721600
H	6.05317100	-0.59574100	-0.63762300
H	5.72086200	-0.78212800	1.07379300
H	3.65082300	-0.08914900	-1.06341900
H	3.31372700	-0.28611000	0.67251000
H	5.19293100	1.80826500	-0.52687500
H	4.81297300	1.64081800	1.17455900
H	3.55082200	3.48659800	0.06014600

H	2.80318300	2.35658300	-1.03232900
H	2.41660600	1.83752800	1.96218900
H	0.16813700	2.50234300	1.72329300
H	0.61610900	4.34410100	-0.37368000
H	0.58911100	2.85454600	-1.26732000
H	-1.64116700	3.97962100	-1.16611100
H	-1.63529900	3.87246400	0.58081000
H	-1.65267600	1.37067500	0.43430700
H	-1.65941600	1.47669600	-1.32765800
H	-3.81430200	2.68062000	-1.28619800
H	-3.82765000	2.67045500	0.46449700
H	-3.88964600	0.15838900	-1.27335400
H	-5.31037300	0.93595500	-0.61259100
H	-4.44873300	0.61303600	1.70540600
H	-3.06656500	-0.25275400	1.10374800
N	0.75414600	-0.23119100	-0.54285600
H	0.29740700	0.05065400	-1.40988000
H	0.90800700	0.60977200	0.05395600
H	1.66608700	-0.64350200	-0.74740500
H	0.12341100	-0.93096800	-0.05689900

conf_174

C	4.62859400	0.07422100	-1.17484300
C	3.92107700	1.14927700	-0.34566300
C	3.33283300	2.27405000	-1.19981600
C	2.61010000	3.33582900	-0.36242400
C	1.39749600	2.79937300	0.35339700
O	0.68862200	1.90640200	-0.07402500
O	1.05666200	3.35531400	1.53088500
H	1.68323100	4.04258200	1.79549500
H	2.24750000	4.15030500	-1.00234100
H	3.29486900	3.78928200	0.36214000
H	2.63115000	1.85944600	-1.92838400
H	4.12311200	2.77199100	-1.76809000
H	4.62470000	1.56807400	0.38513100
H	3.11209800	0.68910100	0.22983600
C	5.11717100	-1.12656900	-0.34926200
C	4.01655200	-1.86670400	0.42587500
C	2.93958500	-2.50159300	-0.46384600
C	1.67387600	-2.92125800	0.29381700
C	0.79577600	-1.71362700	0.68629200
C	-0.45012800	-2.12862600	1.42112100
C	-1.65010600	-2.37726700	0.87191600
C	-2.01147000	-2.29435700	-0.58744500
C	-3.48247600	-1.93948800	-0.84836700
C	-3.88357000	-0.54607300	-0.36006800
C	-5.32448800	-0.15460500	-0.68952400
C	-5.70052900	1.23899100	-0.17940400
C	-7.14092000	1.63240900	-0.51098500
H	-7.85134200	0.92968200	-0.06691200
H	-7.37908100	2.62976100	-0.13454500
H	-7.30766500	1.63493100	-1.59168300
H	-5.01308700	1.97825800	-0.61164300

H	-5.55530200	1.27575900	0.90850100
H	-5.47025300	-0.19780600	-1.77477200
H	-6.00620400	-0.89685100	-0.25884100
H	-3.20586900	0.19484700	-0.81444200
H	-3.75347800	-0.49777900	0.73109300
H	-3.67635100	-2.01086500	-1.92258600
H	-4.12576100	-2.68637800	-0.37007000
H	-1.80666800	-3.27430900	-1.03626700
H	-1.35657400	-1.59092900	-1.11352100
H	-2.43871200	-2.74280000	1.52808500
H	-0.33312200	-2.32754200	2.48503300
H	0.54935600	-1.16257900	-0.22712800
H	1.38083600	-1.03482200	1.31607400
H	1.07340200	-3.59090800	-0.32881400
H	1.94027600	-3.48909600	1.19201200
H	2.64700000	-1.80910000	-1.26068700
H	3.36688600	-3.37450300	-0.96720500
H	4.47830700	-2.65160000	1.03356800
H	3.55492400	-1.17823900	1.14152800
H	5.62110500	-1.83030400	-1.02030300
H	5.87885200	-0.78537800	0.36057300
H	3.95238600	-0.26685700	-1.96599200
H	5.48578200	0.52213400	-1.68836400
N	-1.37007900	0.88080100	1.35160400
H	-2.25600100	1.06602000	0.87706000
H	-1.25897900	-0.15555300	1.41583500
H	-1.39226900	1.28552900	2.28744100
H	-0.56703100	1.30326700	0.80244800

conf_177

C	4.62859500	0.07404100	-1.17485300
C	3.92116500	1.14919800	-0.34573000
C	3.33294200	2.27393600	-1.19993700
C	2.61028000	3.33581000	-0.36261100
C	1.39780900	2.79936900	0.35346400
O	0.68866700	1.90663200	-0.07402800
O	1.05745300	3.35498500	1.53121900
H	1.68422600	4.04205500	1.79586500
H	2.24752000	4.15013800	-1.00262500
H	3.29513500	3.78944600	0.36175600
H	2.63122600	1.85930500	-1.92845900
H	4.12321800	2.77180700	-1.76828200
H	4.62483700	1.56800500	0.38501600
H	3.11219100	0.68910800	0.22985000
C	5.11708800	-1.12673400	-0.34921200
C	4.01641000	-1.86678700	0.42591000
C	2.93947800	-2.50169900	-0.46384200
C	1.67370100	-2.92124800	0.29375800
C	0.79556100	-1.71358000	0.68600100
C	-0.45026100	-2.12847700	1.42102600
C	-1.65029200	-2.37726300	0.87200000
C	-2.01184900	-2.29464100	-0.58732300
C	-3.48283200	-1.93961400	-0.84815100

C	-3.88368700	-0.54605800	-0.36006100
C	-5.32457000	-0.15442700	-0.68945500
C	-5.70037600	1.23928900	-0.17949000
C	-7.14071700	1.63289700	-0.51103500
H	-7.30753300	1.63532600	-1.59172400
H	-7.85123700	0.93033800	-0.06684800
H	-7.37870600	2.63032900	-0.13469700
H	-5.01283800	1.97839900	-0.61185000
H	-5.55507600	1.27617200	0.90840400
H	-5.47043600	-0.19775600	-1.77468600
H	-6.00636400	-0.89652300	-0.25862900
H	-3.20590700	0.19468600	-0.81460800
H	-3.75347800	-0.49759600	0.73107900
H	-3.67682500	-2.01116400	-1.92233800
H	-4.12617900	-2.68633200	-0.36966500
H	-1.80726400	-3.27474100	-1.03592800
H	-1.35690700	-1.59145600	-1.11366300
H	-2.43879900	-2.74270800	1.52833800
H	-0.33314000	-2.32722400	2.48495600
H	0.54903700	-1.16274700	-0.22752300
H	1.38063100	-1.03457800	1.31556600
H	1.07328300	-3.59099800	-0.32882200
H	1.94002200	-3.48897100	1.19205400
H	2.64699900	-1.80925300	-1.26076700
H	3.36677600	-3.37466500	-0.96710800
H	4.47811200	-2.65165000	1.03368800
H	3.55474500	-1.17827500	1.14149900
H	5.62100600	-1.83052300	-1.02021100
H	5.87877500	-0.78556100	0.36062800
H	3.95235700	-0.26702600	-1.96598400
H	5.48581100	0.52186200	-1.68841200
N	-1.37007800	0.88088300	1.35158600
H	-1.39230100	1.28547000	2.28748600
H	-2.25601400	1.06613000	0.87707400
H	-1.25893400	-0.15546700	1.41567800
H	-0.56706200	1.30345400	0.80250400

conf_53

C	-1.46198400	-1.66326900	1.83252700
C	-2.93165400	-1.49720200	1.42687400
C	-3.18161400	-1.14438700	-0.04543700
C	-2.73861100	-2.24148300	-1.03082700
C	-1.27467600	-2.21180400	-1.37737500
O	-0.65841800	-1.18750000	-1.61584700
O	-0.61294000	-3.37690600	-1.49011400
H	-1.17958400	-4.12960700	-1.27323600
H	-3.25354000	-2.10060400	-1.98890900
H	-3.02643200	-3.23091900	-0.66177400
H	-2.69032600	-0.20883300	-0.31901400
H	-4.25226900	-0.98468000	-0.19052600
H	-3.38410300	-0.71602200	2.04617800
H	-3.47648900	-2.41899200	1.66041900
C	-0.66217300	-0.35669000	1.82136300

C	0.81259000	-0.52254600	2.21071800
C	1.63114100	-1.30249400	1.17548600
C	3.13865200	-1.35312400	1.45393700
C	3.86927400	-0.00982800	1.24850100
C	3.98038200	0.40665300	-0.19323800
C	3.30051600	1.38248100	-0.81591600
C	2.22960100	2.25808500	-0.23332000
C	0.87905200	2.10522300	-0.95225300
C	-0.23759600	2.92921800	-0.30955500
C	-1.57982200	2.76953000	-1.02852400
C	-2.75044100	3.48649800	-0.34355700
C	-3.13442500	2.89582100	1.01647400
H	-3.99677600	3.41566000	1.43994500
H	-3.39890000	1.83760600	0.92378500
H	-2.32036700	2.97213600	1.74169800
H	-3.62078500	3.44975100	-1.00653700
H	-2.50441300	4.54792600	-0.22599900
H	-1.47816900	3.14493700	-2.05326200
H	-1.81304600	1.70084600	-1.11973500
H	0.04621800	3.98791600	-0.29651800
H	-0.33460900	2.62478600	0.73769400
H	0.98800500	2.38874700	-2.00699000
H	0.57456900	1.05288000	-0.93880100
H	2.55057400	3.30377700	-0.30567500
H	2.09491600	2.04695700	0.82881400
H	3.56483900	1.60169700	-1.85179600
H	4.74201900	-0.12212000	-0.76731200
H	4.88493800	-0.10620400	1.64456800
H	3.38549300	0.76903100	1.83930000
H	3.30143000	-1.68710600	2.48328500
H	3.60744300	-2.11269500	0.81603300
H	1.25287800	-2.32709100	1.10719300
H	1.44939000	-0.84193800	0.19951800
H	1.24844600	0.47141200	2.34704300
H	0.88679600	-1.02230800	3.18343300
H	-0.71758500	0.10932300	0.83274700
H	-1.13977300	0.34419100	2.51372200
H	-1.42595400	-2.08739300	2.84157800
H	-0.97813200	-2.41104100	1.19187600
N	1.93421400	-1.12123900	-2.33519000
H	2.01581800	-0.78992600	-3.29670300
H	2.44355300	-0.46845100	-1.70121700
H	2.34843100	-2.05090900	-2.26159800
H	0.90759500	-1.17291300	-2.05447700

conf_4

C	3.53693900	-0.06039900	1.20206300
C	4.45180300	0.17614900	-0.00664900
C	4.79965300	-1.08749700	-0.80790500
C	3.72033600	-1.52931300	-1.82447800
C	2.39531400	-1.86051200	-1.19890600
O	1.37899400	-1.21273200	-1.38176900
O	2.33080100	-2.91639100	-0.37437700

H	3.19148500	-3.34731500	-0.27938300
H	4.07228000	-2.41032500	-2.37251200
H	3.54165900	-0.73576000	-2.54964500
H	5.03859500	-1.90827200	-0.12027400
H	5.70881500	-0.91419300	-1.38794400
H	5.38486600	0.61886000	0.35569100
H	4.00970100	0.91576500	-0.68279900
C	3.28890100	1.19594700	2.04801100
C	2.53121000	2.33004700	1.34507200
C	1.11355400	1.96787300	0.88694700
C	0.33375400	3.18285200	0.37473800
C	-1.12118300	2.89464100	-0.01308200
C	-1.27704500	1.99899500	-1.21835300
C	-2.42353200	1.44248200	-1.63765900
C	-3.77281700	1.60623200	-0.99133300
C	-4.74734500	0.45432300	-1.28014500
C	-4.28354500	-0.92149000	-0.78695000
C	-4.08779700	-1.02029400	0.72850300
C	-3.68274600	-2.42026200	1.20212200
C	-3.34660600	-2.48669500	2.69213400
H	-4.20201000	-2.17520000	3.29669600
H	-3.07381500	-3.49866600	2.99842900
H	-2.51264900	-1.82312300	2.94868100
H	-2.83065100	-2.79715700	0.60978400
H	-4.49130900	-3.12109600	0.97262700
H	-3.34478300	-0.28576700	1.06892600
H	-5.01659000	-0.73302200	1.23314600
H	-3.35383700	-1.19948300	-1.30445500
H	-5.01756000	-1.67210700	-1.09637500
H	-5.71444700	0.69109000	-0.82609700
H	-4.92174100	0.40197800	-2.35980300
H	-4.21623000	2.53563700	-1.37130400
H	-3.66559600	1.76227600	0.08650600
H	-2.40507500	0.88291700	-2.57186900
H	-0.39035600	1.86314400	-1.83576500
H	-1.62349900	3.84590200	-0.22624100
H	-1.65973100	2.47925400	0.84922600
H	0.34181100	3.95693700	1.14779900
H	0.85799600	3.60955500	-0.48808100
H	1.16691300	1.20588300	0.10002300
H	0.56951500	1.52644800	1.73748000
H	2.46721300	3.17703000	2.03580200
H	3.10219500	2.69227300	0.48346000
H	2.73493800	0.90776800	2.94902400
H	4.25257800	1.58085500	2.39791300
H	2.57514800	-0.47249100	0.88320100
H	3.99420100	-0.82457700	1.84070100
N	-0.80466900	-0.81284300	0.11606700
H	-1.23631700	-0.01325600	-0.39779700
H	-1.49419200	-1.55617000	0.24630000
H	-0.48623900	-0.47841500	1.02521500
H	0.02794000	-1.14275500	-0.44436100

conf_15

C	3.74631300	0.51639200	0.43039300
C	4.83059700	-0.30387300	-0.27632700
C	4.71241500	-1.82265800	-0.09154800
C	3.60652100	-2.48422000	-0.94663800
C	2.20392800	-2.07637300	-0.59670500
O	1.41920900	-1.55854800	-1.36958100
O	1.77748100	-2.30681200	0.66430600
H	2.47945900	-2.68496900	1.21192900
H	3.66409800	-3.57382500	-0.83884900
H	3.75239500	-2.25059000	-2.00108300
H	4.57822400	-2.06212600	0.97175800
H	5.65091500	-2.30049600	-0.38126800
H	5.81104600	0.01630300	0.08838400
H	4.81838200	-0.07747900	-1.34897500
C	3.77623900	1.99921500	0.05461200
C	2.68718000	2.83936400	0.73245900
C	1.25760200	2.33701300	0.48283700
C	0.19146100	3.38679700	0.80603000
C	-1.25137700	2.89053700	0.65518100
C	-1.60806600	2.45879700	-0.74912200
C	-2.77866200	1.92423700	-1.12653400
C	-3.96087500	1.67357000	-0.22894500
C	-4.92655800	0.60045900	-0.75310300
C	-4.33259600	-0.81038000	-0.84035800
C	-3.97842900	-1.42938000	0.51539600
C	-3.38714800	-2.83997500	0.41064100
C	-2.93478600	-3.41188700	1.75464300
H	-2.15876300	-2.79134600	2.21707400
H	-3.77061700	-3.46034400	2.45698500
H	-2.53284700	-4.42133600	1.64570500
H	-2.54529100	-2.85634000	-0.30254000
H	-4.13066100	-3.50203300	-0.04371500
H	-3.28835900	-0.77676800	1.06934900
H	-4.87841400	-1.47031000	1.13804600
H	-3.44742100	-0.79404800	-1.49314500
H	-5.04775500	-1.46582100	-1.34678300
H	-5.81224200	0.57719000	-0.11081100
H	-5.27753800	0.90094400	-1.74561600
H	-4.51220100	2.61833500	-0.13577300
H	-3.63106100	1.43865000	0.78851900
H	-2.93045600	1.72432200	-2.18592700
H	-0.87627000	2.67586800	-1.52446400
H	-1.93769500	3.68783100	0.96281500
H	-1.43805700	2.07488700	1.36950100
H	0.33634000	3.74579000	1.82968600
H	0.33959500	4.25549200	0.15497600
H	1.16649800	2.02575400	-0.56604700
H	1.07651100	1.44079400	1.09358300
H	2.86784800	2.88595900	1.81244200
H	2.76670000	3.86851400	0.36736200
H	4.75811300	2.41874900	0.29681000
H	3.66612700	2.08685100	-1.03342200
H	2.75854700	0.12505600	0.17878000
H	3.85368900	0.40465200	1.51696400

N	-0.80389900	-0.56684700	-0.17230800
H	-1.53561500	-1.28107500	-0.14381100
H	-0.49030100	-0.37850500	0.77941500
H	-1.19567200	0.30977100	-0.57491300
H	0.02396000	-0.92930300	-0.72204900

conf_102

C	0.14541800	3.40208900	-1.14072200
C	1.12917100	2.36615800	-0.57611100
C	1.58129500	2.72780500	0.84414900
C	2.33718000	1.60891200	1.56204500
C	1.44777200	0.48662300	2.02528600
O	0.22968100	0.53732600	2.03120700
O	2.03068100	-0.62592600	2.49557500
H	2.99396000	-0.58277000	2.41741200
H	2.83596000	1.99221800	2.46189400
H	3.12892300	1.18971800	0.93027600
H	0.71944400	3.01033400	1.45343000
H	2.23169600	3.60526400	0.79861900
H	2.01088200	2.28619800	-1.22053400
H	0.65264400	1.37957900	-0.57984900
C	-1.29425400	3.19048400	-0.64283200
C	-2.08293800	2.19079400	-1.49891300
C	-3.43317200	1.79316500	-0.87606900
C	-3.35324600	0.55444400	0.03020200
C	-3.29648000	-0.75253600	-0.78268200
C	-3.27164200	-1.99488600	0.05863200
C	-2.36597800	-2.98380100	-0.00445100
C	-1.17967200	-3.03121500	-0.93456400
C	0.03990000	-2.25016700	-0.41399900
C	1.22380900	-2.27507200	-1.38318700
C	2.42369800	-1.47308900	-0.87852200
C	3.60963300	-1.44359400	-1.84403300
C	4.77347200	-0.60165100	-1.31846500
H	4.47037400	0.44009700	-1.16777200
H	5.14237900	-0.98892200	-0.36253900
H	5.61446300	-0.59831900	-2.01490100
H	3.27826000	-1.04849800	-2.81060000
H	3.95017300	-2.46744200	-2.03215400
H	2.10214000	-0.44284000	-0.68411200
H	2.75258300	-1.88788300	0.08329100
H	0.90147600	-1.87442300	-2.35139100
H	1.52504900	-3.31260800	-1.56601800
H	-0.25318200	-1.20902800	-0.23195500
H	0.35800200	-2.66274800	0.55314700
H	-1.45802900	-2.63378100	-1.91440800
H	-0.89410800	-4.07408100	-1.09608000
H	-2.50918400	-3.84732600	0.64370200
H	-4.10904500	-2.11114500	0.74750300
H	-2.44134900	-0.73075200	-1.46046300
H	-4.19208000	-0.79136500	-1.41541700
H	-2.46612500	0.63607500	0.67289600
H	-4.22535900	0.52269500	0.69293700

H	-3.82401100	2.64077500	-0.30448700
H	-4.16890900	1.59593300	-1.66219100
H	-2.24495400	2.63651200	-2.48488200
H	-1.47681900	1.29365300	-1.67602700
H	-1.27757800	2.85308600	0.40079000
H	-1.83383000	4.14247400	-0.64264300
H	0.49520200	4.40406300	-0.87123500
H	0.15393000	3.36505200	-2.23451700
N	-1.51285600	-1.37526700	2.67194400
H	-1.09769700	-2.14549400	3.19629600
H	-2.25098800	-0.94567800	3.23020300
H	-1.92444700	-1.74126200	1.78461400
H	-0.76654800	-0.64847000	2.44472400

conf_30

C	-4.27405300	0.06832900	0.84257300
C	-5.17327900	-1.17374200	0.90385800
C	-4.80335200	-2.33295300	-0.03137300
C	-3.44809100	-2.99833500	0.28655900
C	-2.22271100	-2.22811800	-0.11692900
O	-1.26040100	-2.02967000	0.60221500
O	-2.16197900	-1.75909400	-1.38027100
H	-2.98350400	-1.93438400	-1.86017400
H	-3.38218100	-3.96057500	-0.23674000
H	-3.35870000	-3.20696600	1.35281100
H	-4.84806400	-2.01044100	-1.07986400
H	-5.56653800	-3.10943300	0.05622600
H	-6.20359300	-0.87638200	0.68367200
H	-5.18117800	-1.55571600	1.93024200
C	-4.34318000	0.84326500	-0.47837400
C	-3.53341400	2.14593700	-0.47285200
C	-2.01190700	1.96043200	-0.43966900
C	-1.26261000	3.29449800	-0.37786500
C	0.26499000	3.16670800	-0.35167100
C	0.79890000	2.47792500	0.88044300
C	2.04279000	2.00159300	1.03825800
C	3.14863800	2.06089900	0.01523500
C	4.30631900	1.09028200	0.28235600
C	3.92120000	-0.38908800	0.18039100
C	5.06440800	-1.36857100	0.47678100
C	6.22064100	-1.31711600	-0.52706500
C	7.28982100	-2.37350500	-0.24254300
H	6.87008500	-3.38280500	-0.28683900
H	8.10430500	-2.31903300	-0.96817800
H	7.72190600	-2.23733100	0.75303100
H	6.68307800	-0.32523700	-0.51447900
H	5.82388600	-1.45880700	-1.54033900
H	5.44695400	-1.17237200	1.48543100
H	4.66127600	-2.38756200	0.49943000
H	3.54808000	-0.58697300	-0.83908700
H	3.10293200	-0.59459900	0.88420000
H	5.11135700	1.30738300	-0.42291800
H	4.71294200	1.28278500	1.28178600

H	3.54211900	3.08507600	0.00196500
H	2.75144000	1.90194100	-0.99652100
H	2.31230000	1.59687800	2.01206200
H	0.12770200	2.43830000	1.73617700
H	0.70573200	4.16991300	-0.40222400
H	0.61648100	2.66369900	-1.26325300
H	-1.55240300	3.90886300	-1.23561100
H	-1.58673100	3.84401900	0.51311900
H	-1.73348200	1.34280400	0.42285600
H	-1.70539200	1.40871300	-1.34094600
H	-3.79224800	2.73270500	-1.36023700
H	-3.83674600	2.75044800	0.38991400
H	-4.00536300	0.22093000	-1.31537300
H	-5.39173700	1.08174300	-0.68553600
H	-4.58077200	0.74250900	1.64933000
H	-3.24013400	-0.20510100	1.07759400
N	0.59950900	-0.40256000	-0.48895000
H	0.18580300	-0.10983900	-1.37371900
H	0.77249000	0.43693500	0.10512300
H	1.49822300	-0.85772100	-0.65798700
H	-0.07850000	-1.06697500	-0.01654000

conf_132

C	3.70447400	0.00419100	-1.34380200
C	3.28400800	1.02984500	-0.28677800
C	2.50351800	2.20441400	-0.88082600
C	2.00711700	3.19779700	0.17182100
C	0.90303100	2.66446200	1.04831000
O	0.31285100	1.61764500	0.85302800
O	0.52110800	3.41309200	2.09581600
H	1.04955200	4.21967600	2.17253400
H	1.60838800	4.10323200	-0.30420900
H	2.83040500	3.53093300	0.81560400
H	1.64544900	1.83093000	-1.44699700
H	3.13349600	2.74907400	-1.58963200
H	4.17579800	1.40607800	0.22966000
H	2.66695000	0.54307100	0.47273600
C	4.38610500	-1.24176500	-0.75823400
C	3.46502800	-2.11652800	0.10421000
C	2.39175800	-2.87815300	-0.69099100
C	1.17428200	-3.26065200	0.16089500
C	0.21844500	-2.06739300	0.36595500
C	-0.81913900	-2.33522200	1.41670400
C	-2.11501300	-2.62875700	1.22720300
C	-2.84670200	-2.77531900	-0.07944100
C	-3.81154800	-1.60723800	-0.37323200
C	-3.12799900	-0.40358200	-1.03294100
C	-4.00872600	0.85025900	-1.05006600
C	-3.42735400	2.03655200	-1.82783600
C	-2.15565900	2.62476200	-1.21216200
H	-1.79945900	3.47929800	-1.79238500
H	-2.34431000	2.98734500	-0.19372800
H	-1.34290000	1.89625100	-1.16969700

H	-4.18804600	2.81937400	-1.89840200
H	-3.22130100	1.71843500	-2.85546800
H	-4.98233800	0.58488900	-1.47459900
H	-4.23510200	1.16893300	-0.01702300
H	-2.86680400	-0.66791900	-2.06355900
H	-2.16628600	-0.19826400	-0.54851500
H	-4.61616800	-1.95317100	-1.02773000
H	-4.30437400	-1.30149000	0.55983100
H	-3.42029700	-3.70564400	-0.02719500
H	-2.14717600	-2.89031200	-0.91092300
H	-2.71801600	-2.83263700	2.11188100
H	-0.44696000	-2.35318100	2.44153900
H	-0.24712000	-1.81847000	-0.59103300
H	0.80183300	-1.18809600	0.66183200
H	0.61425400	-4.07480000	-0.30827000
H	1.50911400	-3.63901200	1.13351000
H	2.04818800	-2.27851000	-1.54144900
H	2.83945100	-3.77849000	-1.12143600
H	4.06977900	-2.84153500	0.65758400
H	2.98756600	-1.49408400	0.86906700
H	4.79017400	-1.84608300	-1.57742300
H	5.24817100	-0.92584100	-0.16037000
H	2.82244100	-0.29184300	-1.92338400
H	4.38436300	0.48300900	-2.05641300
N	-1.93081500	0.59511700	1.92527300
H	-2.72814500	0.92796500	1.37880400
H	-1.83153500	-0.43436300	1.78029700
H	-2.08143500	0.79688300	2.91332900
H	-1.04732600	1.07061700	1.58123600

conf_25

C	-3.14564500	0.25384000	-0.51065100
C	-3.89682000	-0.90087800	-1.17517900
C	-3.13918700	-2.23405400	-1.18195400
C	-2.85159600	-2.79421900	0.22045400
C	-1.67682000	-2.16043700	0.91642700
O	-0.65769800	-1.80378100	0.35402300
O	-1.72478800	-2.03141300	2.25690500
H	-2.57380100	-2.32715400	2.61237000
H	-2.59490100	-3.85875800	0.14822200
H	-3.74415100	-2.73153300	0.85184500
H	-2.19180800	-2.13250400	-1.71833700
H	-3.72840200	-2.97955900	-1.72069900
H	-4.11818200	-0.63112200	-2.21267800
H	-4.86892900	-1.03780200	-0.68552700
C	-3.88305700	1.59350500	-0.58316600
C	-3.07820600	2.74353800	0.03613100
C	-1.85618600	3.17779800	-0.80540600
C	-0.56483700	3.35899400	0.00095700
C	0.03493100	2.01787100	0.47018300
C	1.27584300	2.20792800	1.30043300
C	2.54043700	2.22265000	0.84925700
C	2.99385000	2.06544700	-0.57514100

C	4.33194100	1.32403100	-0.72714000
C	4.32845600	-0.12570800	-0.22924600
C	3.34371500	-1.04413500	-0.95908200
C	3.43537600	-2.51100400	-0.52485800
C	2.36575400	-3.40007900	-1.16061600
H	1.35744400	-3.05839200	-0.90723900
H	2.45213700	-3.38426900	-2.25024800
H	2.46526900	-4.43841500	-0.83641200
H	3.38161200	-2.59495900	0.57517700
H	4.43220400	-2.89192300	-0.76789000
H	2.31312800	-0.68576400	-0.83474900
H	3.53174000	-0.98746200	-2.03687300
H	4.12783200	-0.13640400	0.85252300
H	5.33771300	-0.53569300	-0.33576700
H	4.61962700	1.33828800	-1.78291400
H	5.10781300	1.88112200	-0.19182300
H	3.11792700	3.07356200	-0.99053500
H	2.21764300	1.59389200	-1.18293700
H	3.32963600	2.44121400	1.56754100
H	1.11408700	2.44780900	2.35068900
H	0.24561200	1.40730800	-0.41379400
H	-0.71681600	1.47510700	1.05367600
H	0.18458500	3.87545000	-0.60615500
H	-0.75333200	3.99960300	0.86960300
H	-1.66842500	2.44611000	-1.59984500
H	-2.08580000	4.11523300	-1.31828500
H	-3.73430600	3.60404600	0.18912800
H	-2.75480800	2.43966000	1.03957800
H	-4.11412100	1.82955500	-1.62856500
H	-4.84643600	1.50004400	-0.07035700
H	-2.96783900	0.03030500	0.55034500
H	-2.15591800	0.34413400	-0.97037200
N	1.45514200	-0.86507100	1.76120400
H	1.63461100	0.14672400	1.58127300
H	1.30573400	-1.01158800	2.75920500
H	2.26664000	-1.40554500	1.45061500
H	0.60119500	-1.19380100	1.22593500

conf_40

C	2.36852100	-2.11082000	-0.91173300
C	3.77284100	-1.50043700	-0.99147400
C	3.99145900	-0.19420700	-0.21462100
C	3.67607200	-0.29631300	1.30134700
C	2.25268500	0.01813300	1.65763300
O	1.46829000	-0.73964200	2.19624800
O	1.80314500	1.26159700	1.36791500
H	2.46182300	1.79236400	0.89793300
H	4.29885200	0.41640900	1.85400700
H	3.90208000	-1.29157400	1.68279500
H	3.41281800	0.61654300	-0.67202700
H	5.03872200	0.09690500	-0.31680900
H	4.02727900	-1.31119100	-2.03961300
H	4.49788700	-2.23783700	-0.63135700

C	1.25013600	-1.20009600	-1.42880700
C	-0.10635800	-1.89836200	-1.58402100
C	-0.63917600	-2.53135300	-0.29434900
C	-2.04142200	-3.13477000	-0.43510000
C	-3.17403900	-2.10151500	-0.54583900
C	-3.41473500	-1.34833500	0.73261800
C	-3.78379300	-0.06674100	0.87215200
C	-4.03070200	0.95043100	-0.20751400
C	-3.04782300	2.13662900	-0.16575900
C	-1.69098800	1.83319200	-0.80988200
C	-0.65023000	2.93063100	-0.57127600
C	0.63736300	2.74505000	-1.37677300
C	1.71472300	3.77712200	-1.03922900
H	1.96893800	3.76441400	0.02835500
H	2.63041100	3.60591300	-1.61048800
H	1.37542300	4.79206000	-1.26154500
H	0.40029600	2.80355100	-2.44410400
H	1.02157100	1.73341700	-1.21405100
H	-1.09093900	3.90497800	-0.81040400
H	-0.39969200	2.98679600	0.50042800
H	-1.83736900	1.70417300	-1.88776100
H	-1.30217900	0.86652400	-0.46205000
H	-3.49223600	2.99368000	-0.67932500
H	-2.91401200	2.46134500	0.87634900
H	-5.04881200	1.33064000	-0.07314500
H	-4.00405900	0.48845600	-1.19682700
H	-4.00912300	0.28398500	1.88091200
H	-3.35502800	-1.95549500	1.63683600
H	-4.10172900	-2.63333000	-0.79197700
H	-2.99265700	-1.41570400	-1.37612200
H	-2.06349500	-3.78158500	-1.31754800
H	-2.24577800	-3.78524200	0.42216800
H	0.04730600	-3.31505700	0.03560300
H	-0.63603800	-1.78383400	0.50996700
H	-0.82954600	-1.17275300	-1.97102500
H	-0.02316900	-2.67954300	-2.34836800
H	1.12510900	-0.34173700	-0.75914100
H	1.54500800	-0.78121300	-2.39775700
H	2.37062900	-3.03521200	-1.49918400
H	2.15665200	-2.41489800	0.11772900
N	-0.96789000	0.45846600	2.26378700
H	-0.75015000	1.35009700	1.81227400
H	-1.72286000	-0.01084100	1.72384600
H	-1.29215300	0.63098400	3.21537200
H	-0.07323400	-0.11052600	2.28200600

conf_57

C	-0.70745300	-1.41342100	-1.44573600
C	-2.10146800	-2.01917800	-1.24305900
C	-2.29910700	-2.85808700	0.02680700
C	-2.16021300	-2.06288800	1.34159500
C	-0.75999800	-1.70760300	1.75836900
O	-0.42084000	-0.60037700	2.14346400

O	0.16749900	-2.67644400	1.75042100
H	-0.19090700	-3.50291400	1.39704800
H	-2.57557100	-2.65128000	2.16950500
H	-2.72724900	-1.13295600	1.29548800
H	-1.63114300	-3.72952700	0.02110900
H	-3.30700100	-3.27878500	0.01418000
H	-2.34844200	-2.64465000	-2.10684100
H	-2.84009700	-1.21123400	-1.23278400
C	0.40230400	-2.42322000	-1.75157100
C	1.78115000	-1.77120000	-1.91420600
C	2.40644400	-1.27755700	-0.60232000
C	3.72602800	-0.52096900	-0.82531000
C	3.51618600	0.96321600	-1.19618200
C	3.37166200	1.85520100	0.00546800
C	2.30717200	2.58036400	0.38210900
C	0.95926800	2.66348600	-0.27762400
C	-0.18255000	2.76576500	0.74769500
C	-1.56871100	2.99173800	0.13296100
C	-2.06030900	1.82249800	-0.72498800
C	-3.50112500	1.96679000	-1.23117300
C	-4.55943000	1.87416700	-0.12903300
H	-4.48042800	0.92458500	0.41211300
H	-4.46199000	2.67965100	0.60259600
H	-5.56760100	1.93297000	-0.54523200
H	-3.69222100	1.18641700	-1.97600200
H	-3.60415200	2.91954000	-1.76255100
H	-1.39602500	1.69759000	-1.58605000
H	-1.97854200	0.90008800	-0.13708100
H	-1.55652500	3.90762600	-0.47004400
H	-2.27619400	3.17136900	0.94739000
H	-0.21635400	1.85330700	1.35523200
H	0.04100400	3.59051700	1.43377300
H	0.80460800	1.81443900	-0.94672800
H	0.93873100	3.56090100	-0.91031500
H	2.43370600	3.23626300	1.24449600
H	4.28016800	1.95897000	0.59981000
H	2.65966100	1.06361100	-1.86613000
H	4.38675100	1.31835600	-1.75733100
H	4.36365600	-0.57796600	0.06553300
H	4.28885500	-1.02157300	-1.61807500
H	1.69277200	-0.62592100	-0.08375700
H	2.57009800	-2.14446400	0.04791200
H	2.47052100	-2.48863700	-2.37011700
H	1.69938800	-0.93748200	-2.62144000
H	0.46386000	-3.18987000	-0.97003000
H	0.14561200	-2.95575900	-2.67287900
H	-0.76526300	-0.70817000	-2.28098600
H	-0.43856400	-0.80054100	-0.58047700
N	2.13173800	0.13917400	2.49537800
H	2.26679700	0.58383200	3.40353400
H	2.75836500	-0.66147900	2.41090200
H	2.35125600	0.82226300	1.73666100
H	1.12391800	-0.19333800	2.39601700

conf_96

C	4.40554900	1.07132700	-0.27938400
C	5.73658900	0.31366400	-0.18180900
C	5.70439100	-1.03560500	0.54841000
C	4.85799900	-2.11812700	-0.15360800
C	3.36818000	-1.97153300	-0.03168300
O	2.58582500	-2.04433200	-0.96267600
O	2.85158500	-1.78937600	1.20023100
H	3.54884000	-1.71036500	1.86632100
H	5.09786900	-3.10125200	0.27069400
H	5.08996600	-2.16303400	-1.21780900
H	5.38679000	-0.90123600	1.59065900
H	6.72386100	-1.42228100	0.61286300
H	6.47136100	0.95203800	0.31921400
H	6.12272000	0.14321400	-1.19238500
C	3.85478800	1.57111200	1.06123900
C	2.59837500	2.44036400	0.92709100
C	1.33775400	1.69429800	0.47380100
C	0.14054700	2.63296300	0.29920600
C	-1.15872900	1.94144400	-0.13207000
C	-1.08065800	1.30118100	-1.49567800
C	-1.87046100	0.32648300	-1.97239200
C	-3.01673500	-0.32367500	-1.24847500
C	-4.37219000	0.26883000	-1.68960700
C	-5.58231500	-0.41569800	-1.04516600
C	-5.66516000	-0.26010700	0.47605900
C	-6.94930500	-0.84374300	1.07162900
C	-7.02311000	-0.69840100	2.59221500
H	-6.19016500	-1.21491900	3.07996800
H	-6.98185700	0.35359300	2.89034700
H	-7.95033700	-1.11884800	2.98824300
H	-7.02399100	-1.90286300	0.79849400
H	-7.81290100	-0.34966500	0.61263600
H	-4.80522600	-0.74560800	0.95483900
H	-5.59851300	0.80484400	0.73450600
H	-5.58031600	-1.48166900	-1.30545900
H	-6.49007700	0.00211800	-1.49305400
H	-4.37939200	1.33779700	-1.45066400
H	-4.44788900	0.19517100	-2.77909600
H	-2.91204200	-0.20678100	-0.16625500
H	-3.03349500	-1.40088300	-1.46239800
H	-1.73298500	0.02680100	-3.00962300
H	-0.34389700	1.72867300	-2.17338200
H	-1.96171800	2.68814400	-0.15197200
H	-1.46882000	1.21423800	0.62871100
H	-0.03769000	3.16563200	1.23821200
H	0.39591900	3.39937500	-0.44128300
H	1.53455000	1.17116800	-0.47004400
H	1.09567800	0.92519200	1.22295100
H	2.38990500	2.91964500	1.88915300
H	2.80226300	3.25372600	0.22092500
H	3.63429500	0.73193700	1.73135300
H	4.63496700	2.15632300	1.55941500
H	4.56326400	1.93974400	-0.92776100

H	3.66184600	0.45889200	-0.79982700
N	0.03228300	-1.50612100	-0.30886700
H	0.07690900	-1.19170700	0.66058100
H	-0.36721300	-0.73929800	-0.89524400
H	-0.57112000	-2.32559800	-0.37174100
H	1.02031700	-1.74839900	-0.61912000

conf_184

C	4.05435400	0.54544400	-0.04287500
C	5.30066200	-0.30769600	-0.28449400
C	5.26966500	-1.67645900	0.40894100
C	4.23435100	-2.66671200	-0.16853700
C	2.78848600	-2.31798600	0.06296700
O	1.94805200	-2.28817600	-0.82129000
O	2.39354300	-2.04862300	1.31595200
H	3.14008600	-2.06811300	1.93131200
H	4.38993500	-3.65658000	0.27788000
H	4.36625800	-2.77492400	-1.24518700
H	5.12022300	-1.54277900	1.48941500
H	6.24716900	-2.15424400	0.31216000
H	6.18517900	0.23543100	0.06213100
H	5.43805800	-0.45890900	-1.36153900
C	4.05437000	1.89065200	-0.77476800
C	2.74886600	2.67206400	-0.56730000
C	1.53068400	2.01439800	-1.22902500
C	0.18768500	2.68376500	-0.90983600
C	-0.19426500	2.71114300	0.58653200
C	-0.12353600	1.37112700	1.26426900
C	-1.14777500	0.62822700	1.71291000
C	-2.61563200	0.93330200	1.62114800
C	-3.36189900	-0.00384600	0.65384500
C	-4.87886700	0.19288500	0.66360500
C	-5.61076700	-0.71637200	-0.32538900
C	-7.13005300	-0.52712200	-0.31566100
C	-7.85095900	-1.43904200	-1.30936200
H	-8.93160400	-1.28275200	-1.28006200
H	-7.51619200	-1.25005800	-2.33393900
H	-7.66214500	-2.49358100	-1.08657700
H	-7.36280400	0.51994000	-0.54088000
H	-7.50797800	-0.71341700	0.69600700
H	-5.23284500	-0.53029400	-1.33972500
H	-5.37785800	-1.76484100	-0.09725700
H	-5.10677200	1.24052700	0.43489900
H	-5.25571900	0.01114400	1.67655800
H	-2.99203300	0.16877400	-0.36712800
H	-3.13594800	-1.04897400	0.90917700
H	-2.78544900	1.96832800	1.31838100
H	-3.05499900	0.82265100	2.61876100
H	-0.90187100	-0.30263900	2.22555600
H	0.87959200	0.98590100	1.44045100
H	-1.19240200	3.14080800	0.68284100
H	0.48209800	3.39083500	1.11457100
H	0.19638500	3.71303700	-1.28131800

H	-0.61093400	2.17456400	-1.46520300
H	1.67750100	2.01363000	-2.31473500
H	1.48762800	0.96023600	-0.93135500
H	2.57770900	2.78510000	0.50903600
H	2.85972400	3.68588500	-0.96473500
H	4.89833400	2.49384800	-0.42602100
H	4.21666300	1.72354400	-1.84636100
H	3.17070500	-0.01078000	-0.36097900
H	3.93788600	0.72337500	1.03422300
N	-0.44796300	-1.08688600	-0.88589200
H	-0.47645000	-0.62814500	-1.79676500
H	-1.24244400	-1.72218700	-0.80591100
H	-0.52279500	-0.36290300	-0.13865400
H	0.46887600	-1.61548100	-0.78691200

conf_81

C	3.57003500	-0.85301400	0.61231100
C	3.58387300	-1.55650500	-0.75074500
C	2.89671600	-2.93072800	-0.77009900
C	1.35590500	-2.88293500	-0.92501400
C	0.66315400	-2.15327200	0.18809900
O	0.11078800	-1.07411700	0.05479200
O	0.67493100	-2.70253500	1.41308900
H	1.14886500	-3.54566000	1.42307900
H	0.96093500	-3.90356200	-0.97164300
H	1.08938000	-2.37813900	-1.85325700
H	3.17343600	-3.49878800	0.12639400
H	3.26567000	-3.51384100	-1.61670700
H	4.62616700	-1.68796600	-1.05717600
H	3.12370500	-0.91979000	-1.51358400
C	4.27360700	0.51181700	0.62299100
C	3.68707600	1.56108900	-0.33170700
C	2.19032300	1.83268300	-0.14730000
C	1.69128100	2.99377000	-1.01312100
C	0.16455900	3.13948500	-1.05625900
C	-0.46409900	3.45489500	0.27695100
C	-1.73325800	3.20412000	0.63458500
C	-2.78698900	2.54331000	-0.21350200
C	-3.08150400	1.08718400	0.19000500
C	-4.24955400	0.47118800	-0.58500700
C	-4.56762200	-0.97451500	-0.18469600
C	-3.45628200	-1.98166200	-0.50101300
C	-3.84650600	-3.42041000	-0.15688600
H	-3.04347400	-4.12356700	-0.39487600
H	-4.07638100	-3.52399000	0.90796200
H	-4.73312400	-3.73199700	-0.71571000
H	-2.54238900	-1.71575400	0.04137800
H	-3.20565200	-1.91204600	-1.56652600
H	-4.80018700	-1.01327100	0.88765200
H	-5.48155800	-1.28731300	-0.70046000
H	-4.02601600	0.51124200	-1.65786500
H	-5.13898500	1.09175300	-0.43352400
H	-2.18340400	0.48537400	0.01747100

H	-3.30988700	1.04266300	1.26514200
H	-2.50379300	2.56986400	-1.26803800
H	-3.71036600	3.12578800	-0.12597800
H	-2.06202100	3.54743200	1.61535700
H	0.16361000	4.00222000	0.97995100
H	-0.27751100	2.23272400	-1.48117000
H	-0.08826900	3.95064500	-1.75059200
H	2.14008100	3.93022500	-0.66309500
H	2.05051700	2.84977800	-2.03694700
H	1.61580500	0.93341500	-0.39488300
H	1.99738000	2.05533400	0.91303900
H	4.23931200	2.49690500	-0.19647400
H	3.86238600	1.26207600	-1.37069600
H	4.24830300	0.90614900	1.64574400
H	5.33236400	0.36968600	0.38245000
H	2.54251000	-0.72149100	0.96278400
H	4.05654700	-1.50401700	1.34731900
N	-0.37009000	0.68664100	2.02058100
H	-0.55585300	1.58931100	1.52774900
H	-1.18146000	0.44906300	2.59107500
H	0.45342300	0.78000300	2.61448500
H	-0.20948100	-0.07272300	1.29550000

conf_92

C	3.80390000	1.38372500	-0.92951900
C	5.30031100	1.04682700	-0.88023300
C	5.73963700	0.03578200	0.18729300
C	5.17450100	-1.38613400	-0.01340900
C	3.72808800	-1.57555500	0.34424900
O	2.90411300	-2.12441700	-0.36563400
O	3.31708900	-1.14656400	1.55452500
H	4.02961100	-0.69604400	2.02930900
H	5.73339500	-2.09288300	0.61283500
H	5.29456300	-1.70941400	-1.04753000
H	5.50925800	0.41297600	1.19216700
H	6.82828300	-0.04884500	0.16129500
H	5.86633200	1.97083400	-0.72443700
H	5.60997100	0.66337300	-1.85823600
C	3.27636900	2.13413000	0.29891200
C	1.81427700	2.57884700	0.16942800
C	0.78525500	1.44218300	0.18904300
C	-0.64481400	1.95177900	-0.01022200
C	-1.72646100	0.86533200	0.02088200
C	-1.61346200	-0.13601100	-1.10065200
C	-2.13119200	-1.37322400	-1.13786100
C	-2.95306800	-2.04860500	-0.07439400
C	-4.44214700	-2.15412300	-0.47886900
C	-5.17260000	-0.80885100	-0.55504400
C	-5.40112000	-0.14497200	0.80790100
C	-6.23101100	1.14442900	0.74067300
C	-5.53234200	2.30625700	0.02926200
H	-5.29747900	2.06830700	-1.01163300
H	-6.16227400	3.19870400	0.02417600

H	-4.59750900	2.57051100	0.53544100
H	-6.48545200	1.45269800	1.75967800
H	-7.18276500	0.92959100	0.24180800
H	-5.91164200	-0.86147200	1.46177800
H	-4.44124500	0.07703600	1.29096700
H	-6.14707800	-0.97448700	-1.02661200
H	-4.62593400	-0.13588300	-1.22350900
H	-4.50137000	-2.65942500	-1.44832800
H	-4.94856300	-2.80504200	0.24121900
H	-2.87261500	-1.52565100	0.88264700
H	-2.58139900	-3.07046600	0.07886100
H	-2.01508700	-1.93774600	-2.06169700
H	-1.10579900	0.22021100	-1.99546100
H	-2.70652900	1.34823200	-0.05489400
H	-1.73234700	0.36617200	0.99789500
H	-0.87091900	2.69598300	0.75947700
H	-0.70260300	2.47972500	-0.96885600
H	1.02570400	0.70960500	-0.59118200
H	0.85798300	0.91905500	1.15470200
H	1.57779700	3.27382700	0.98173300
H	1.69639500	3.14764400	-0.76027800
H	3.38207600	1.52564200	1.20468300
H	3.90012200	3.02015200	0.45772300
H	3.63242500	2.01058500	-1.81103900
H	3.22000800	0.47493600	-1.10853000
N	0.38967000	-2.04094000	0.58812400
H	-0.27849900	-1.63187000	-0.10293000
H	0.05177800	-2.95716000	0.88101600
H	0.44348500	-1.42437700	1.39906200
H	1.36588500	-2.12150900	0.17404300

conf_8

C	-3.88908900	0.21817600	-0.97919000
C	-4.60972400	1.31005600	-0.17786900
C	-3.92964300	1.76727100	1.12009600
C	-2.55887500	2.45248700	0.91401600
C	-1.40141900	1.52502300	0.66126100
O	-0.63900800	1.61718500	-0.28521800
O	-1.18258100	0.55072700	1.55675500
H	-1.85708800	0.55488300	2.25023400
H	-2.30027700	3.02477500	1.81339500
H	-2.60005000	3.15694100	0.08335000
H	-3.84571700	0.92964700	1.82390400
H	-4.57884800	2.49264000	1.61535100
H	-5.61800500	0.96351000	0.07129000
H	-4.74229000	2.18980500	-0.81671800
C	-3.88166700	-1.16972200	-0.32575900
C	-3.04444000	-2.18802800	-1.11723700
C	-1.53329700	-2.06469200	-0.85469700
C	-1.09062500	-2.79883500	0.41711100
C	0.32931100	-2.46792400	0.89084800
C	1.41313400	-2.76126000	-0.11718400
C	2.69187900	-2.36703100	-0.01480900

C	3.28074200	-1.58585500	1.13074500
C	4.58786400	-0.85804800	0.78481800
C	4.45101200	0.24139800	-0.27490200
C	3.59238100	1.43413700	0.15757200
C	3.48121500	2.52845300	-0.90938500
C	2.53655300	3.66550900	-0.51828100
H	2.49838900	4.43639000	-1.29090400
H	1.51738700	3.30130800	-0.35558200
H	2.87005500	4.14093300	0.40797800
H	3.16054200	2.09775600	-1.87425900
H	4.47993700	2.92548000	-1.11630400
H	2.58772400	1.10414400	0.45432600
H	4.02131700	1.87203100	1.06539800
H	4.05466500	-0.19633000	-1.20339100
H	5.44876500	0.60610100	-0.53770800
H	5.00262400	-0.42315800	1.69939200
H	5.31908500	-1.59626900	0.43977900
H	3.48359100	-2.29278100	1.94563200
H	2.54308100	-0.88797100	1.54089300
H	3.39367500	-2.70662700	-0.77486800
H	1.14562400	-3.40787400	-0.95075800
H	0.54095500	-3.04284700	1.80102400
H	0.37112600	-1.41794400	1.20106900
H	-1.78413600	-2.56360700	1.23021200
H	-1.17928700	-3.87769900	0.24861600
H	-0.97813300	-2.45982600	-1.71311100
H	-1.27424900	-1.00328500	-0.76990400
H	-3.36118600	-3.20564400	-0.86879700
H	-3.25255500	-2.05883900	-2.18435900
H	-3.50601600	-1.11397100	0.70271600
H	-4.91459200	-1.52227000	-0.24689300
H	-4.38427800	0.13062800	-1.95173900
H	-2.86655800	0.53693400	-1.20792900
N	1.14850900	0.06130500	-1.58536700
H	0.69005800	-0.17176800	-2.46582800
H	1.96569400	0.64958800	-1.76724600
H	1.45608400	-0.82049200	-1.11992600
H	0.47416000	0.59659200	-0.97598900

conf_27

C	4.16481000	-0.40360100	-1.02599200
C	4.64083600	0.54004700	0.08964600
C	4.23676300	2.01143800	-0.11911600
C	2.96858300	2.42370200	0.65625000
C	1.70597900	1.67228300	0.32240200
O	1.00994200	1.12489400	1.15990400
O	1.30207600	1.64576500	-0.95476300
H	1.94640300	2.07715600	-1.53391600
H	2.75458700	3.48525300	0.47838300
H	3.12927200	2.30105100	1.72709800
H	4.12779000	2.22387500	-1.19073500
H	5.03018800	2.67822200	0.22498200
H	5.72924900	0.46936000	0.16002900

H	4.25927500	0.20208800	1.05911000
C	4.20596100	-1.88619200	-0.62895300
C	3.05101700	-2.30049200	0.29549000
C	1.69803800	-2.35390900	-0.42049800
C	0.51298600	-2.54589500	0.52435700
C	-0.82693900	-2.59958900	-0.22054700
C	-2.02345300	-2.63031700	0.69411100
C	-3.26322100	-2.20680800	0.40189400
C	-3.73607900	-1.65388600	-0.91423000
C	-4.88459000	-0.64059100	-0.79158800
C	-4.53819000	0.63710600	-0.01855200
C	-3.42277800	1.47587400	-0.64991500
C	-3.15148000	2.79158200	0.08788800
C	-1.96323900	3.56967400	-0.47846000
H	-1.79183700	4.49627300	0.07382300
H	-1.04470700	2.97540700	-0.44541800
H	-2.14024700	3.83400600	-1.52425400
H	-2.99423400	2.60925100	1.16554400
H	-4.05330700	3.41064900	0.05580600
H	-2.49321800	0.89466500	-0.72236800
H	-3.68987400	1.70540800	-1.68716200
H	-4.27620200	0.37410000	1.01715500
H	-5.43843400	1.25409000	0.06354700
H	-5.22046200	-0.36944600	-1.79719700
H	-5.73646100	-1.12838300	-0.30667400
H	-4.09099700	-2.49999900	-1.51652700
H	-2.90212000	-1.22969600	-1.48010500
H	-4.03505400	-2.34474500	1.15802900
H	-1.86616000	-3.10501100	1.66224500
H	-0.84189200	-3.49563500	-0.85381900
H	-0.89671900	-1.75518800	-0.91629500
H	0.64075800	-3.46312600	1.10997900
H	0.51149700	-1.72299700	1.25144000
H	1.53653400	-1.43113600	-0.98884400
H	1.71213900	-3.16430400	-1.15870000
H	3.26551000	-3.28236000	0.72912300
H	2.98594300	-1.60626600	1.14232800
H	4.17811200	-2.50632800	-1.53095400
H	5.16347100	-2.09992800	-0.14252900
H	3.14593400	-0.14558600	-1.33151400
H	4.78943400	-0.23820100	-1.90957600
N	-1.46761800	0.27434400	1.77121600
H	-1.83768500	-0.61587500	1.37261400
H	-0.52220500	0.52099100	1.37697300
H	-1.38255500	0.17376700	2.78284500
H	-2.12456900	1.03037200	1.56076500

conf_46

C	-3.53377900	0.16716400	0.48231200
C	-3.50456000	1.01783400	-0.79391200
C	-3.09017200	2.48204500	-0.58116600
C	-1.56130200	2.72076400	-0.50106400
C	-0.90435100	2.00323300	0.64097800

O	-0.17627000	1.03417400	0.50371000
O	-1.15752600	2.42575200	1.88835400
H	-1.75203200	3.18870500	1.89330400
H	-1.36127700	3.79260800	-0.40233500
H	-1.08050400	2.37461100	-1.41552600
H	-3.58608300	2.88403600	0.31051900
H	-3.44474500	3.08810800	-1.41781500
H	-4.50530500	1.00479200	-1.23675300
H	-2.84007400	0.56647800	-1.53817700
C	-3.98006700	-1.28517100	0.25964100
C	-3.11352100	-2.09991500	-0.71060100
C	-1.62255100	-2.15194800	-0.35955700
C	-0.84203300	-3.11174000	-1.26403500
C	0.68355100	-2.99879700	-1.14745200
C	1.23297000	-3.34018600	0.21371500
C	2.39800100	-2.90612000	0.71968800
C	3.36008000	-1.97222700	0.02863800
C	3.08746900	-0.48575000	0.31950700
C	4.09540700	0.46376400	-0.33534100
C	3.70174100	1.94066500	-0.19569000
C	2.56420900	2.36555800	-1.13240000
C	2.04846300	3.77559900	-0.83973400
H	1.26710900	4.07275400	-1.54473800
H	1.63750800	3.84162600	0.17405500
H	2.85073200	4.51514100	-0.91050000
H	1.73470100	1.65624300	-1.05636600
H	2.92089300	2.31284800	-2.16697200
H	3.41392400	2.14064000	0.84539900
H	4.57479800	2.57132100	-0.39099000
H	4.19252100	0.21228100	-1.39818000
H	5.08102100	0.29575300	0.11020600
H	2.07888100	-0.23669500	-0.02474700
H	3.10972100	-0.31354600	1.40527900
H	3.32238200	-2.12939400	-1.05260200
H	4.37880100	-2.21816000	0.34206600
H	2.70526500	-3.28680300	1.69293500
H	0.65925200	-4.06325100	0.79319500
H	0.99970300	-1.99388300	-1.44395700
H	1.13856600	-3.68278100	-1.87535000
H	-1.15142800	-4.14198700	-1.05502000
H	-1.11952300	-2.91803100	-2.30495600
H	-1.18583400	-1.15012200	-0.43884900
H	-1.51507800	-2.46709700	0.68937000
H	-3.50640600	-3.12135800	-0.74785900
H	-3.21836100	-1.70599100	-1.72718900
H	-4.00284600	-1.79321200	1.23107400
H	-5.01265300	-1.28919700	-0.10478000
H	-2.55028300	0.16396600	0.95995200
H	-4.21536400	0.63619100	1.20072500
N	0.41145000	-0.90930300	2.24506000
H	-0.45115100	-1.25861900	2.66187000
H	0.84668300	-1.66021700	1.66331600
H	1.06175700	-0.63738500	2.98200900
H	0.19761500	-0.07329000	1.62529300

conf_153

C	-3.62664700	-0.00671100	0.24347200
C	-4.40171600	1.01412600	1.08366300
C	-3.61230400	2.25653000	1.51168100
C	-3.06680000	3.08722400	0.33862400
C	-1.80756700	2.52314000	-0.26352100
O	-0.92750300	1.99500400	0.39223800
O	-1.61749400	2.64216500	-1.58833100
H	-2.37454100	3.06262800	-2.01822900
H	-2.79234000	4.08948500	0.69018100
H	-3.83198600	3.22410800	-0.43251400
H	-2.77455900	1.97594700	2.15537500
H	-4.26217100	2.90355700	2.10510900
H	-4.76882400	0.52048800	1.98927100
H	-5.29538700	1.32697500	0.52998400
C	-4.43844700	-1.28102700	-0.00674700
C	-3.79054300	-2.29823900	-0.95431600
C	-2.42376300	-2.84424900	-0.49309500
C	-1.21807700	-2.17786700	-1.16871400
C	0.13095800	-2.71807200	-0.66457000
C	0.50840400	-2.18712200	0.69727700
C	1.74328900	-2.12243300	1.21619500
C	3.01153800	-2.60641400	0.56427100
C	3.91236700	-1.48167400	0.01332100
C	4.34878200	-0.45300600	1.06258300
C	5.24440900	0.66460400	0.51372900
C	4.52691700	1.66306100	-0.40187500
C	5.43844000	2.78323700	-0.90747400
H	4.89920900	3.48515800	-1.54787300
H	5.86149500	3.34704400	-0.07230100
H	6.26938400	2.37253700	-1.48657000
H	3.69411400	2.11725400	0.16091700
H	4.10346900	1.13752300	-1.26839800
H	5.67856600	1.21781500	1.35248200
H	6.08777600	0.22355300	-0.02964200
H	4.88211000	-0.98122100	1.85967700
H	3.47249000	-0.00247800	1.54823900
H	4.80482200	-1.93786900	-0.42623700
H	3.39927700	-0.99116200	-0.82346400
H	2.78143500	-3.30653800	-0.24158400
H	3.58068200	-3.17055900	1.31107800
H	1.84351300	-1.71828200	2.22060900
H	-0.31740700	-1.84906800	1.32046400
H	0.92286100	-2.50013100	-1.38958400
H	0.07475300	-3.81322600	-0.62320000
H	-1.29004900	-2.34643300	-2.24778600
H	-1.26511200	-1.09012000	-1.02526400
H	-2.34372300	-2.75669100	0.59684100
H	-2.36682700	-3.91609400	-0.70713100
H	-4.48716700	-3.13223000	-1.07305600
H	-3.68701800	-1.85390300	-1.95210300
H	-4.63541800	-1.76583200	0.95689100
H	-5.41836800	-1.00247300	-0.41032100

H	-3.35359700	0.42914800	-0.72742100
H	-2.68601900	-0.25284300	0.74822900
N	1.19180100	0.63552600	-0.52667400
H	2.08486700	1.12100500	-0.40388400
H	1.19959900	-0.24597600	0.02961300
H	1.07177300	0.40221200	-1.51182900
H	0.38024400	1.24000600	-0.20813900
